# Catalytic Enantioselective Hydrogen Atom Abstraction Enables the Asymmetric Oxidation of *Meso* Diols

**DOI:** 10.1021/jacs.4c13919

**Published:** 2024-11-26

**Authors:** Nelson Y. S. Lam, Jyoti Dhankhar, Antti S. K. Lahdenperä, Robert J. Phipps

**Affiliations:** Yusuf Hamied Department of Chemistry, Lensfield Road, Cambridge CB2 1EW, United Kingdom

## Abstract

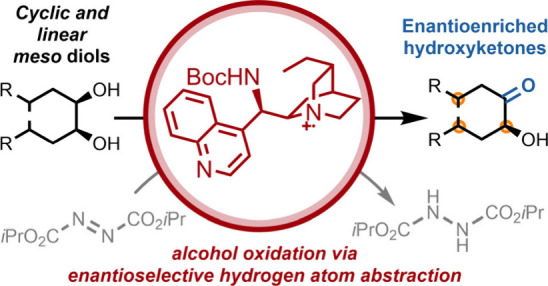

Desymmetrization of *meso* diols is an important strategy for the synthesis of chiral oxygen-containing building blocks. Oxidative desymmetrization is an important subclass, but existing methods are often constrained by the need for activated alcohol substrates. We disclose a conceptually distinct strategy toward oxidative diol desymmetrization that is enabled by catalytic enantioselective hydrogen atom abstraction. Following single electron oxidation of a cinchona alkaloid-derived catalyst, enantiodetermining hydrogen atom abstraction generates a desymmetrized ketyl radical intermediate which reacts with either DIAD or O_2_ before *in situ* elimination to form valuable hydroxyketone products. A range of cyclic and acyclic *meso* diols are competent, defining the absolute configuration of up to four stereocenters in a single operation. As well as providing rapid access to complex hydroxyketones, this work emphasizes the broad synthetic potential of harnessing hydrogen atom abstraction in an enantioselective manner.

Alcohol oxidation is among the most commonly used transformations in organic synthesis.^1^ Classically carried out with stoichiometric oxidative reagents, recent developments have led to a suite of catalytic methods that permit the use of less activated oxidants.^2^ The inclusion of a catalyst provides opportunities to perform enantioselective oxidation in a practical manner without requiring stoichiometric chiral oxidants,^3^ and has been widely applied to the kinetic resolution of racemic chiral secondary alcohols.^4,5^ Alternatively, catalytic enantioselective alcohol oxidation can be used to desymmetrize *meso* diols. This requires a chiral catalyst to selectively oxidize one of two enantiotopic hydroxyl groups, generating a single chiral product. Several important advances have been made toward the enantioselective oxidation of *meso* primary diols, spanning a range of catalytic approaches (Figure 1A, left).^6^ In contrast, enantioselective oxidation protocols for *meso* secondary diols^7^ are largely limited to activated (*e*.*g*. benzylic) alcohols (Figure 1A, right).^3c,8^ To the best of our knowledge, there is only a single example demonstrated on nonactivated *meso* secondary alcohols: Hua and co-workers in 2016 utilized chiral Pd/Au nanoclusters as catalysts to give excellent enantioselectivities in the oxidation of simple carbocyclic *meso* diols.^9^ However, this method has not been extended to acyclic and more complex diol substrates, in which other functional groups or prochiral stereocenters were present.

**Figure 1:**
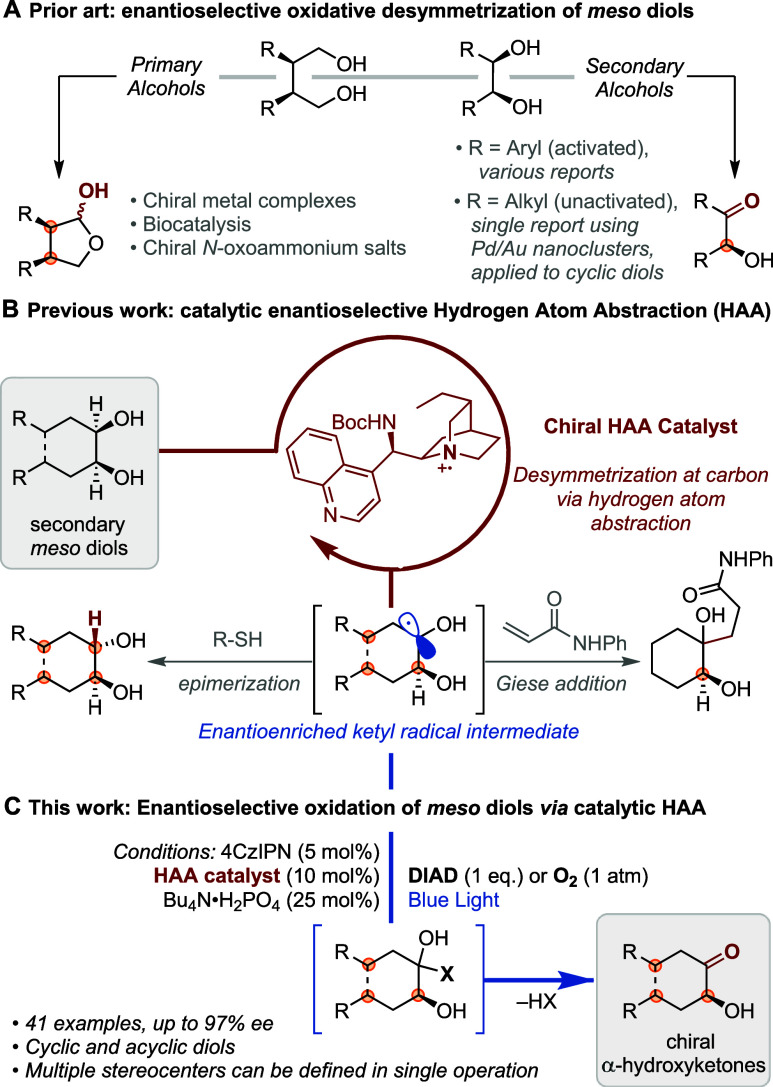
Prior art relating to enantioselective oxidative desymmetrization of *meso* diols and enantioselective hydrogen atom abstraction.

By taking advantage of structural parallels between cinchona alkaloids and the established hydrogen atom abstraction (HAA) catalyst quinuclidine,^10^ we recently developed a series of chiral catalysts capable of performing enantioselective HAA from *meso* diols (Figure 1B).^11−14^ After single electron oxidation of a modified cinchona alkaloid, the resulting chiral quinuclidinium radical cation selectively abstracts one of the two enantiotopic hydrogen atoms from the achiral diol substrate, setting the adjacent hydroxyl stereocenter in the process. Inspired by the prior work of Wendlandt,^15^ we demonstrated that the resulting ketyl radical can be intercepted with a thiol hydrogen atom donor to afford enantioenriched *trans* diols or trapped with electron deficient olefins in Giese addition. In addition to introducing a chiral catalyst for HAA, our work represented a rare example of an asymmetric diol desymmetrization which takes place at carbon rather than oxygen, an outcome achieved by harnessing a radical mechanism involving hydrogen atom transfer.^16,17^

Leveraging the known nucleophilic character of the enantioenriched ketyl radical intermediate,^18^ we speculated whether this may be trapped with a suitable oxidant which, upon *in situ* elimination, would deliver enantioenriched hydroxyketone-containing products. These are prevalent in bioactive molecules and synthetic intermediates^19^ and extensive literature precedent exists for their elaboration.^20^ Most approaches for asymmetric oxidation are initiated by enantiodetermining *O*-functionalization.^21^ In contrast, this process would offer a conceptually distinct strategy, where enantioselection is initiated by desymmetrization at carbon rather than oxygen. Herein we report the realization of this approach using either diisopropyl azodicarboxylate (DIAD) or oxygen as mild oxidants to intercept the desymmetrized ketyl radical (Figure 1C). Enantioselective oxidation of a range of cyclic and linear *meso* secondary diols is possible, in many cases allowing the absolute configuration of multiple stereocenters to be defined in a single operation.

We commenced our optimization using *meso* cyclohexane-1,2-diol (**1a**) as the model substrate, using the optimal chiral HAA catalyst from our previous work^11^ (*epi-*NHBoc-DHCN, 10 mol %), 4CzIPN as photocatalyst (5 mol %) and the additive Bu_4_N·H_2_PO_4_ (25 mol %) at +10 °C (Table 1). An evaluation of oxidants (entries 1–4) identified diisopropyl azodicarboxylate (DIAD) as the best performer and acetonitrile as the optimal solvent (entry 4, 54% yield, 82% ee; see SI for full details).^22,23^ Interestingly, omission of the Bu_4_N·H_2_PO_4_ additive resulted in negligible reactivity (entry 5),^10^ its importance here contrasting with our reported enantioselective epimerization reaction where its impact was minimal on the same substrate.^11^ Yield and enantioselectivity markedly improved when the reaction was conducted at −35 °C (entry 6, 91% ee), and a minor yield improvement was obtained on addition of 4 Å molecular sieves (entry 7).^24^ Control experiments varying the conditions from entry 7 were conducted to establish the importance of each component. Omission of blue light (entry 8), photocatalyst (entry 9) or HAA catalyst (entry 10) resulted in no product formation. Finally, conducting the reaction in the absence of DIAD gave minimal product formation (entry 11), indicating its key role.

**Table 1:**
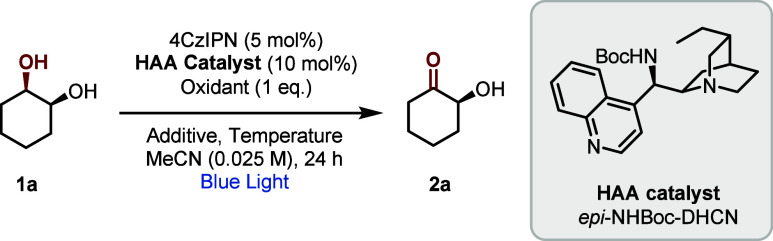
Selected Reaction Optimization and Controls

	Oxidant	Conditions	Yield^a^	ee^b^
1.	TCCA	Bu_4_N·H_2_PO_4_ (25 mol %), +10 °C	45%	8%
2.	BzOO*t*Bu	Bu_4_N·H_2_PO_4_ (25 mol %), +10 °C	18%	76%
3.	O_2_ (1 atm)	Bu_4_N·H_2_PO_4_ (25 mol %), +10 °C	20%	82%
4.	**DIAD**	Bu_4_N·H_2_PO_4_ (25 mol %), +10 °C	54%	82%
5.	**DIAD**	No Bu_4_N·H_2_PO_4_, +10 °C	2%	n.d.
6.	**DIAD**	Bu_4_N·H_2_PO_4_ (25 mol %), **–35 °C**	78%	91%
7.	**DIAD**	Bu_4_N·H_2_PO_4_ (25 mol %), **4 Å MS**, **–35 °C**	**92%**^c^	**91%**
8.		No Light	0%	n.d.
9.		No 4CzIPN (Photocatalyst)	0%	n.d.
10.		No HAA catalyst	0%	n.d.
11.		No DIAD	8%	n.d.

a Yields determined by ^1^H NMR using CH_2_Br_2_ as internal standard.

b Enantiomeric excess (ee) determined by chiral SFC analysis from the benzoate ester derivative of **2a**.

c Isolated yield of **2a** prior to derivatization. TCCA: Trichloroisocyanuric Acid.

With optimized conditions, we proceeded to examine the scope of *meso* cyclic diols (Scheme 1A; see SI for ineffective examples). Six, seven and eight-membered carbocyclic *meso* diols were competent (**2a**–**c**). Reduced yield due to product volatility and lower enantioselectivities was observed for five-membered diol **2d**, which required a telescoped derivatization to the corresponding benzoate ester for isolation. A fused benzene ring (**2e**), cyclic protected amine (**2f**), esters (**2g**–**h**) and acetals (**2i**–**j**) were effective, showing no cross-reactivity at other C–H bonds potentially liable to HAA. Remarkably, alkenes (**2k**–**l**), motifs which may interfere in radical processes, were highly effective; no evidence of alkene isomerization, radical addition or competitive HAA at allylic sites was observed. We evaluated the corresponding *meso*-1,3 isomer of **1a** which underwent oxidation but gave low ee (see SI). We next explored a series of more elaborate *meso* cyclic diols and functionalized 5,6-fused bicycles were first examined (**2m**–**2s**). Notably, cyclic ethers (**2m**), protected amines (**2n**), acetonides (**2o**), esters (**2p**–**s**) and nitriles (**2q**–**s**) were tolerated. Extension to 7,6-bicycles (**2t**–**u**), as well as more strained 4,6- (**2v**) and 3,6-fused bicycles (**2w**–**x**), and monocyclic tetrasubstituted substrate **2y** was also amenable. In the case of **2w**, in which the starting diol contains an epoxide, the hydroxyketone product was unstable. This underwent *in situ* elimination to afford the dihydroxyenone **2w′** as a single diastereomer in good yield and excellent enantioselectivity. While diminished enantioselectivity was observed for substrates containing bulky substituents (*e*.*g*. **2x** and **2z**), the success of **2x** attests to the mildness of this method as it contains both cyclopropyl and alkyl chloride motifs. Finally, 3,8-fused (**2z-2aa**) and 5,8-fused (**2ab-2ac**) bicycles were also highly effective. In the above cases, our enantioselective oxidation can set the absolute configuration of up to four stereocenters in a single operation, generating stereodefined hydroxyketones that would be challenging to access using conventional methods.^19b^

**Scheme 1:**
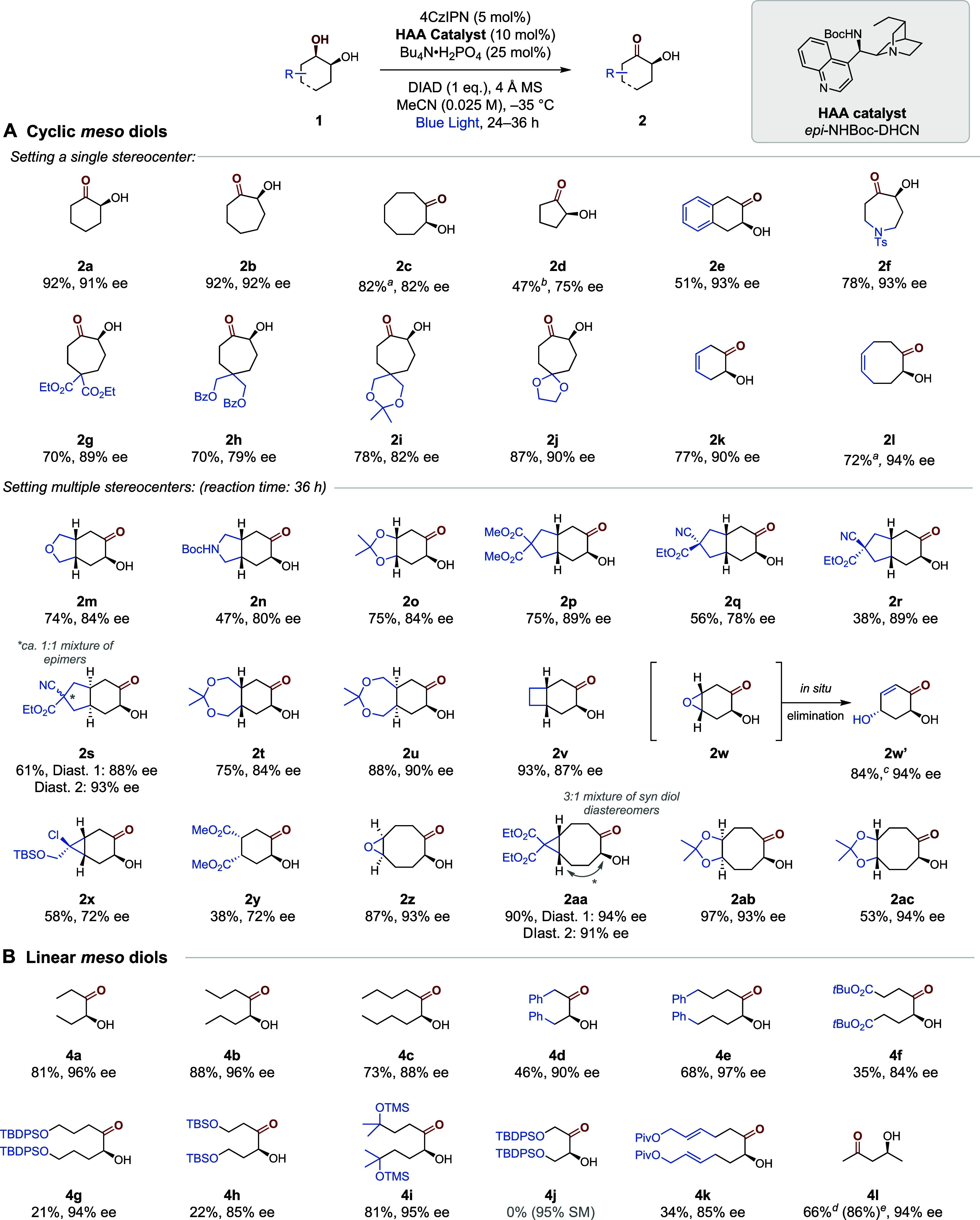
Scope of Enantioselective Oxidation for (A) Cyclic and (B) Linear *Meso* Diols Isolated yields of hydroxyketone. Enantiomeric excess (ee) determined after *O*-benzoylation for products without a chromophore. 36 h reaction time, Isolated as *O***-**Bz ester, Fifteen mol % HAA catalyst loading, Isolated as TBDPS ether, NMR yield in parentheses.

A series of linear *meso* alcohols were next examined (Scheme 1B). Simple diols bearing alkyl chains were highly effective, giving the corresponding hydroxyketone products (**4a**–**c**) with high ee values. The reaction was tolerant of phenyl substituents (**4d**–**4e**), affording the products in high ee with no evidence of deleterious HAA at the benzylic position. Diesters (**4f**) and silyl ethers (**4g**–**h**) proved amenable. In these cases, we attribute the low yields to electronic deactivation toward HAA resulting in moderate conversions; an effect that could apparently be counteracted by inclusion of extra alkyl groups in **4i**. As may be expected, moving the ether motif even closer to the site of HAA shut down the reaction completely (**4j**). In another example of tolerance to alkene functionality, a pivalate-protected diallylic alcohol (**4k**) gave modest yield of the product but in good enantioselectivity. We also demonstrate that enantioselective oxidation of an acyclic *meso* 1,3-diol could also be achieved; *meso*-pentane-2,4-diol underwent oxidation with high yield and enantioselectivity (**4l**). A reduced yield was obtained due to product volatility, and preparative isolation required telescoping to the corresponding TBDPS ether (a known intermediate for the total synthesis of altohyrtin A).^25^ This result indicates potential applications toward accessing chiral β-hydroxyketones distinct from a classical aldol-type disconnection. Comparison with literature optical rotation values for cyclic hydroxyketones **2a**–**d**, linear α-hydroxyketones **4a**–**4d** and TBDPS-protected β-hydroxyketone **4l′** indicated that the major enantiomer for newly set hydroxyl stereocenter was *S*-configured across all substrate classes. This observation was consistent with our enantioselective diol epimerization, in line with a common mechanism involving enantiodetermining HAA by the cinchona alkaloid derived catalyst. For modest-yielding reactions that gave nonvolatile products, the remaining mass balance typically consists of recovered starting material; overoxidation was not observed, indicating that enantioselectivity was not enhanced by downstream resolution processes. It is desirable that both product enantiomers can be readily accessed.

Gratifyingly, subjecting a sample of substrates with pseudoenantiomeric dihydrocinchonidine-derived HAA catalyst (*epi*-NHBoc-DHCD) afforded the antipodal cyclic (*ent***-2b** and *ent***-2l**) and linear (*ent-***4b**) hydroxyketones with excellent yields and ees (Scheme 2A). During reaction optimization, we observed that oxygen gas could be used as the terminal oxidant, maintaining good enantioselectivity albeit with reduced product conversion (Table 1, entry 3, see SI for further optimization).^24^ To explore this further, we subjected diols **1a**, **1b**, **1l** and **3b** to the enantioselective oxidation under a static atmosphere of O_2_ in the absence of DIAD. In all cases, the desired product was obtained in high enantioselectivities, albeit with modestly reduced yields (Scheme 2B). This demonstrates that use of molecular oxygen in this protocol is viable and may find applications in reaction scale up with further development, potentially in a flow setting.^26^ We also investigated a modestly scaled up reaction, which was successfully achieved on 2.5 mmol of **3b** (corresponding to a 25-fold increase in reaction scale compared to Scheme 1), employing slightly modified conditions to conserve photocatalyst, HAA catalyst and additives (Scheme 2C). This afforded the target hydroxyketone **4b** in 71% yield and 97% ee.

**Scheme 2:**
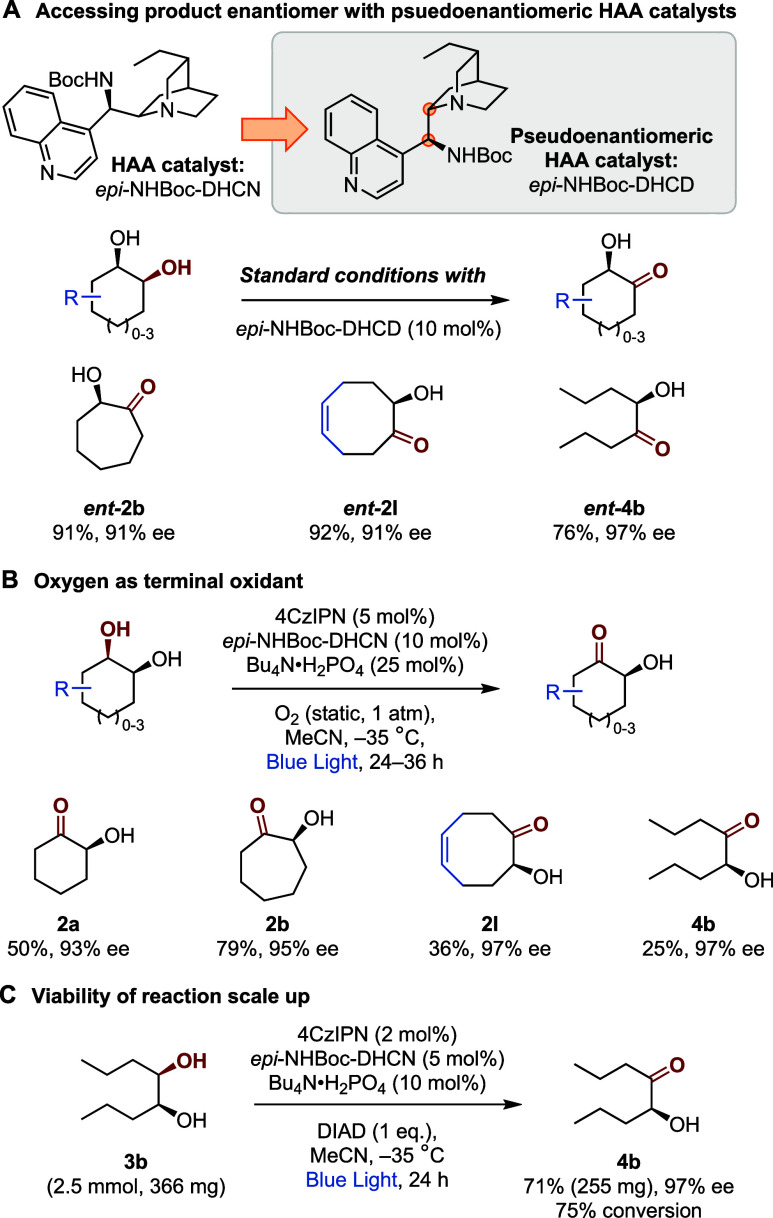
Further Experiments to Expand Method

Regarding the photoredox aspect of the mechanism, one possibility involves reductive quenching of the photocatalyst by *epi*-NHBoc-DHCN (typically proposed with quinuclidine^10^), HAA from the diol, followed by quenching of the intermediate ketyl radical with DIAD prior to reduction and elimination (see SI for complete depiction). However, Stern Volmer fluorescence quenching experiments revealed that DIAD is a faster (1.5x) quencher of the excited state photocatalyst than *epi*-NHBoc-DHCN, presumably through oxidative quenching to form 4CzIPN^·+^ and DIAD^·–^ (Figure 2B, see SI for discussion). The 10-fold greater concentration of DIAD at the start of the reaction would suggest that photocatalytic oxidation of the HAA catalyst may be minimal under these conditions.^27^ Studies suggest that initially formed DIAD^•–^ radical anion may be protonated by catalytic amounts of Bu_4_N·H_2_PO_4_ to afford the neutral DIAD^•^ radical and it is plausible that the photocatalytic cycle (blue) could close through single electron oxidation of the HAA catalyst (Figure 2A). Enantiodetermining HAA from *meso* diol ***I*** would ensue to generate an enantioenriched ketyl radical in ***II***. This could potentially combine with DIAD^•^ to afford ***III***, which eliminates *in situ* to afford H_2_DIAD ***IV*** and hydroxyketone ***V*** (*shown*). Alternatively, electron transfer between DIAD^•^ and ***II*** followed by proton transfer (*not shown*) is also a possibility at this point.

**Figure 2:**
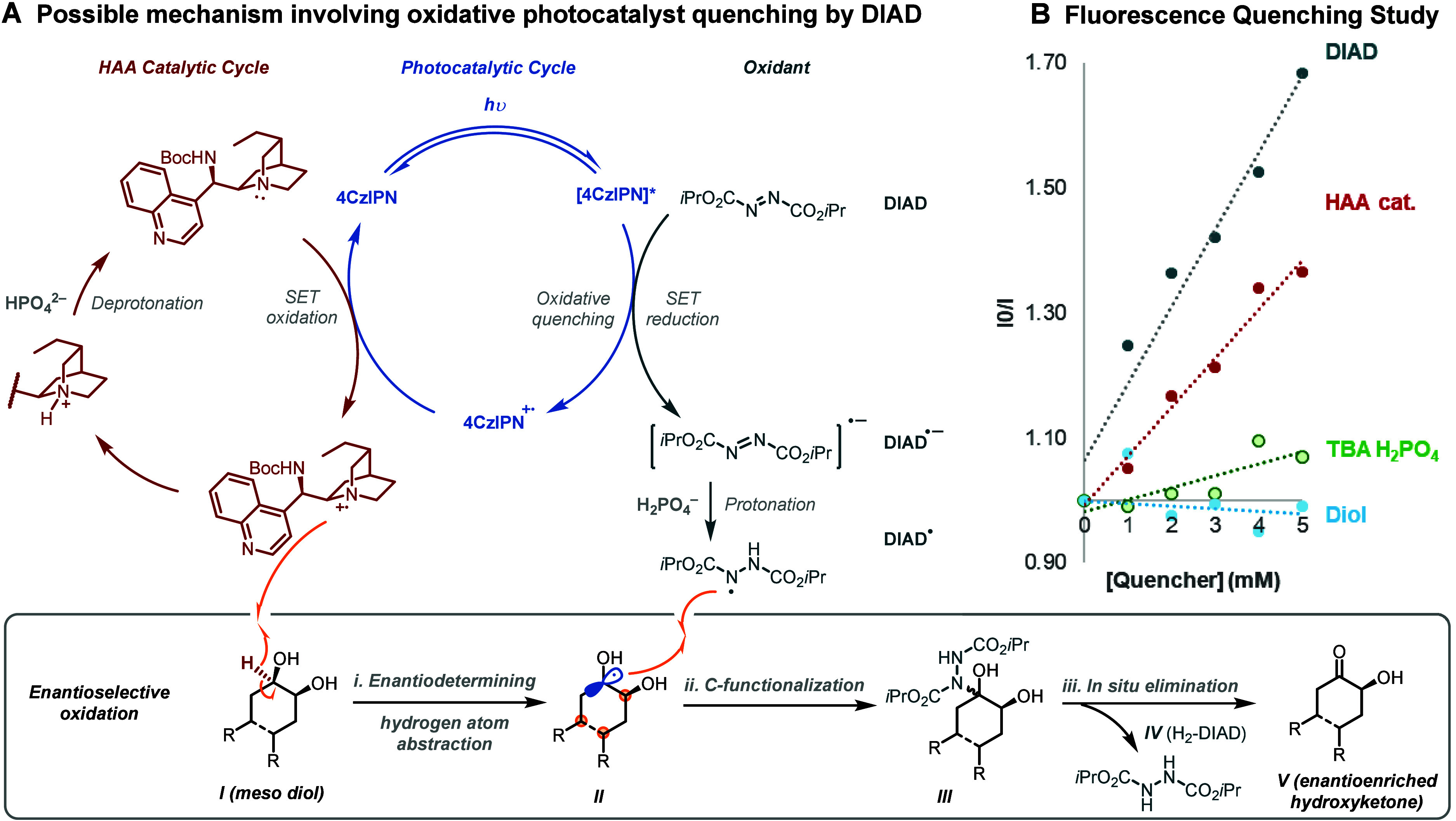
(a) Possible reaction mechanism; (b) Fluorescence quenching study indicates competitive quenching of the excited state photocatalyst by DIAD.

In summary, we report a conceptually distinct strategy to achieve the enantioselective oxidation of *meso* secondary diols through catalytic hydrogen atom abstraction. A desymmetrized ketyl radical intermediate could be trapped by either DIAD or molecular oxygen to furnish hydroxyketones with very high levels of enantioenrichment following *in situ* elimination. The mildness of this method permits a range of substrates bearing potentially sensitive functionalities, including alkenes, to be competent in this reaction. Given the broad utility of alcohol oxidation, we envisage that our method can serve as a powerful complement to established oxidations capable of being deployed as a strategic transformation in asymmetric synthesis.^28^ Additionally, this method demonstrates the potential offered by chiral catalysts capable of enantioselective HAA, facilitating new asymmetric approaches to abundant motifs.

## Supporting Information Available

Reaction optimization, procedures, characterization data and mechanistic discussion (PDF)
