# Supplementary material for: Catalytic Enantioselective Hydrogen Atom Abstraction Enables the Asymmetric Oxidation of Meso Diols
Source: J Am Chem Soc. 2024 Nov 26;146(49):33302–8. doi: 10.1021/jacs.4c13919 (PMC11638968; doi:10.1021/jacs.4c13919)

Catalytic Enantioselective Hydrogen Atom Abstraction Enables the Asymmetric Oxidation of *Meso* Diols

Nelson Y. S. Lam, Jyoti Dhankhar, Antti S. K. Lahdenperä, Robert J. Phipps *

Yusuf Hamied Department of Chemistry, Lensfield Road, Cambridge,
CB2 1EW, United Kingdom

Supporting information

TABLE OF CONTENTS

1. GENERAL CONSIDERATIONS 3

2. GENERAL PROCEDURES 4

2.1. Photoreactor set up 4

2.2. Substrate synthesis 5

2.3. Enantioselective oxidation 5

2.4. Racemate synthesis 7

3. REACTION OPTIMIZATION 8

4. SYNTHESIS AND CHARACTERIZATION OF STARTING MATERIALS 12

4.1. List of known substrates 12

4.2. Synthesis and characterization of new *meso* diols 13

PRODUCT CHARACTERIZATION 29

4.1. Enantioenriched hydroxyketones 29

4.2. Absolute stereochemistry assignment 110

4.3. Enantioselective oxidation with O_2 111

4.4. Reaction scale up 117

5. INEFFECTIVE SUBSTRATES 119

5.1. Substrates that give poor reactivity 119

5.2. Substrates with sub-optimal reactivity and/or enantioselectivity 119

5.3 Cyclic 1,3 diol substrate 120

6. MECHANISTIC ANALYSIS 121

6.1. Initial Mechanistic Hypothesis 121

6.2. Stern Volmer Fluorescence Quenching Study 121

6.3. Role of TBA·H_2PO_4 in reaction 129

6.4. Proposed reaction mechanism deduced from mechanistic experiments 135

7. REFERENCES 136

8. SPECTROSCOPIC DATA FOR NOVEL COMPOUNDS 137

1. General considerations

Reagents: All reagents were used as supplied from commercial sources without further purification. Acetonitrile (MeCN) was purified by distillation under inert atmosphere from CaH_2. Synthesis of 4CzIPN[1] and HAA catalysts (*epi*-NHBoc-dihydrocinchonine and -dihydrocinchonidine) were conducted according to literature procedures.[2] 4 Å molecular sieves was powdered and used directly from the bottle.

NMR spectra: ^{1}H NMR spectra were recorded on a 700 MHz Bruker TXO spectrometer, 500 MHz Bruker DCH Cryoprobe or 400 MHz Bruker Avance NEO prodigy N_2 Cryoprobe. Chemical shifts are reported in parts per million (ppm) to two decimal places, and the spectra are calibrated to the resonance resulting from incomplete deuteration of the solvent ($CDCl_3$: 7.26 ppm; d_6-DMSO: 2.50 ppm; d_3-MeOD: 3.31 ppm). Data are reported as follows: chemical shift δ/ppm, integration, multiplicity (s = singlet, d = doublet, t = triplet, q = quartet, qn = quintet, sext = sextet, sept = septet, br = broad, m = multiplet or combinations thereof. Coupling constants *J in* Hz ^{13}C NMR spectra were recorded on the same spectrometers with complete proton decoupling. Chemical shifts are reported in ppm to one decimal place, with the solvent resonance as the internal standard ($CDCl_3$: 77.2 ppm, d_6-DMSO: 39.5 ppm; d_3-MeOD: 49.0 ppm). ^{1}H-^{1}H COSY, ^{1}H-^{1}H NOESY and ^{1}H-^{13}C HSQC were used where appropriate to facilitate structural determination of stereoisomers.

High Resolution Mass Spectrometry (HRMS): Recorded on a Waters Micromass LCT Premier spectrometer or on a Waters Xevo G2-S benchtop QTOF using an electrospray ionization (ESI). Measured values are reported to 4 decimal places are within ± 5 ppm of the calculated value. The calculated values are based on the most abundant isotope.

Chromatography: Analytical thin layer chromatography was performed using precoated Merck glass backed silica gel plates (Silicagel 60 F254). Visualisation was by ultraviolet fluorescence (λ = 254 nm) and/or staining with cerium ammonium molybdate (CAM) or potassium permanganate ($KMnO_4$). Flash column chromatography was performed using silica gel 60 (0.040-0.063 µm) from Material Harvest Ltd.

Optical rotations: Measured in spectrophotometric grade $CHCl_3$ on a Perkin Elmer 43 Polarimeter using a sodium lamp (λ = 589 nm, D-line). All $[\alpha]_D$ values are reported at 25 °C with concentration in g/100mL.

Chiral SFC analysis: Performed on a Waters ACQUITY UPC2 system with DAICEL CHIRALPAK IA, C, IE, IG or IK columns (4.6 x 250 mm, 3 µm) in a mixed solvent system of supercritical CO_2 and MeOH. A system backpressure of 138 bar was used in all cases

Chiral HPLC analysis: Performed on a Waters ARC system with DAICEL CHIRALPAK IG column (4.6 x 250 mm, 3 µm) in a mixed solvent system of *n*-hexane and *i*PrOH.

2. General procedures

2.1. Photoreactor set up

All enantioselective oxidation reactions are carried out in the following reaction set up, which is depicted in the photo below (**Figure S1**) and described below. An immersion chiller and associated thermoprobe was placed in a glass dish filled with EtOH as the cooling bath. The cooling bath was placed in a larger glass dish and insulated with a layer of cotton wool. The combined set up was placed in a large Styrofoam box, with a cut out at the base to accommodate a Kessil® Tuna Blue lamp. The Styrofoam box is further insulated with cotton wool, and a flow of dry nitrogen was provided to ensure the air around the chiller remained dry to mitigate significant formation of ice around the reactor. A stirrer hotplate was positioned on top such that stirring was conducted from above. A Styrofoam lid was affixed and the lamp switched on (**Figure S2**).

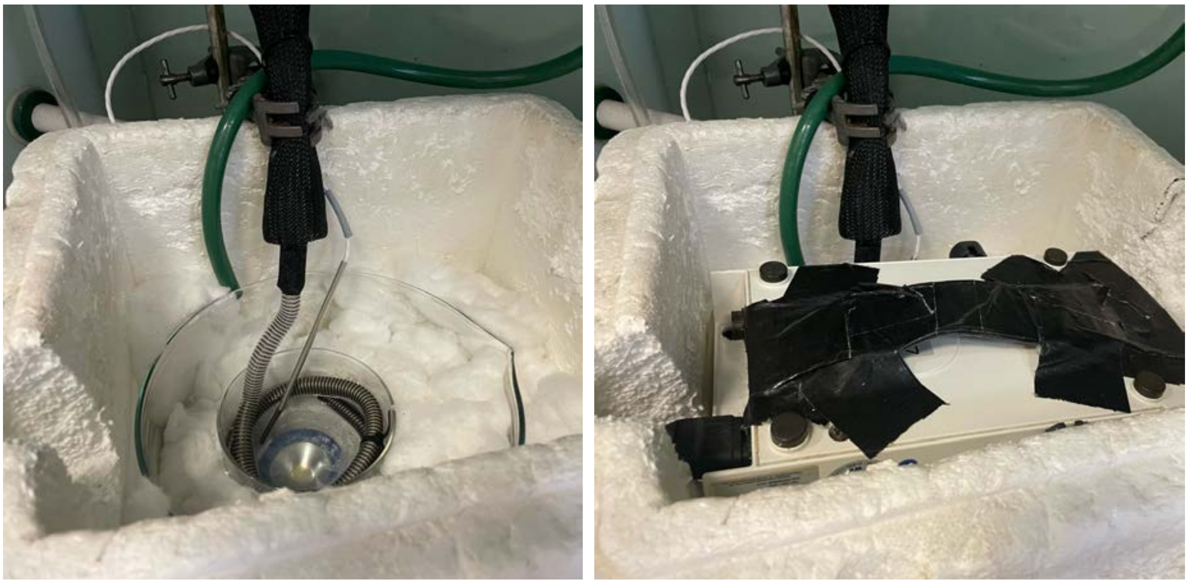

Figure S1. *(Left) Photoreactor set up from above.*
(Right) Photoreactor with stirrer hot plate affixed from above

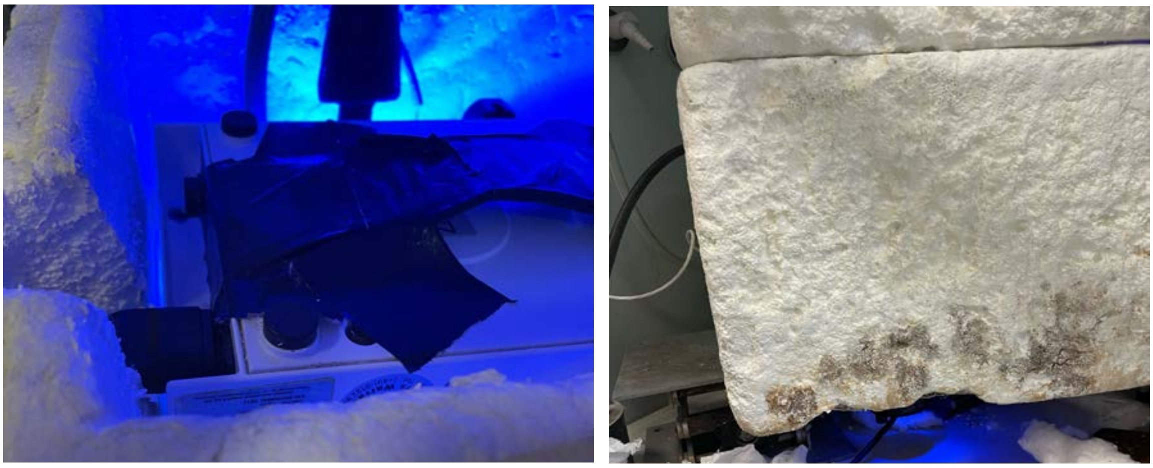

Figure S2. *(Left) Set up with Kessil® lamp switched on (top view; open lid);*
(Right) Exterior view of set up with lid affixed (side vide)

2.2. Substrate synthesis

General Procedure A: *meso* syn diol synthesis *via* Upjohn dihydroxylation

A solution of *N*-methylmopholine-*N*-oxide (NMO, 50% w/w in water, 1.2 eq.) was added to a stirred solution of the alkene substrate (1 eq.) and $K_2OsO_4{\cdot}2H_2O$ (0.02 eq.) in acetone/H_2O (2:1 v/v, 0.3 M). The solution was stirred for 16 h at r.t., after which the reaction mixture was evaporated to dryness *in vacuo* and adsorbed onto SiO_2. Column chromatography of the SiO_2-adsorbed crude mixture (typically acetone/hexanes) affords the meso *syn*-diol product.

General Procedure A': resolution *via* acetonide formation, chromatography and deprotection
In some cases, diastereomeric enrichment of *syn*-diols could be achieved by protecting the above *syn*-diol as the corresponding dimethyl acetonide. This was conducted by dissolving the product in a mixture of CH_2Cl_2/2,2-dimethoxypropane (0.2 M; 3:1 v/v) and *p*-TsOH·H_2O (10 mol%) was next added. The reaction was then stirred for 16 h at r.t. before quenching the reaction with a few drops of Et_3N, and the mixture was evaporated to dryness under reduced pressure. Purification by column chromatography then enables partial chromatographic separation of the mixture. The acetonide protected product was then dissolved in MeOH (0.1 M) and a few drops of HCl (2 M in MeOH) was added. The reaction was stirred for 16 h before evaporating to dryness. The crude product was re-dissolved in CH_2Cl_2, and solid $NaHCO_3$ was added until no effervescence was observed. The mixture was filtered over $MgSO_4$ in a plugged glass pipette, and the solvent removed under reduced pressure to afford the diastereomerically enriched *syn* diol.

2.3. Enantioselective oxidation

General Procedure B: Catalytic enantioselective oxidation: DIAD as oxidant
Enantioselective reactions were carried out in 4 mL (15 x 45mm) crimp-top vials

Meso diol substrate (0.1 mmol), HAA catalyst (0.01 mmol, 10 mol%), 4CzIPN (3.9 mg, 0.005 mmol, 5 mol%), TBA·H_2PO_4 (8.5 mg, 0.025 mmol, 25 mol%), 4 Å molecular sieves (20 mg) and diisopropyl diazocarboxylate (DIAD, 20 mg, 0.1 mmol) was added in a 4 mL reaction vial equipped with a stirrer bar. Dry MeCN (4 mL) was next added, and the vial was sealed with a Teflon-lined metal cap with a vial crimper, and further sealed with electrical tape around the perimeter (**Figure S3**)
N.B. no precautions were taken to remove air or moisture during reaction set up.

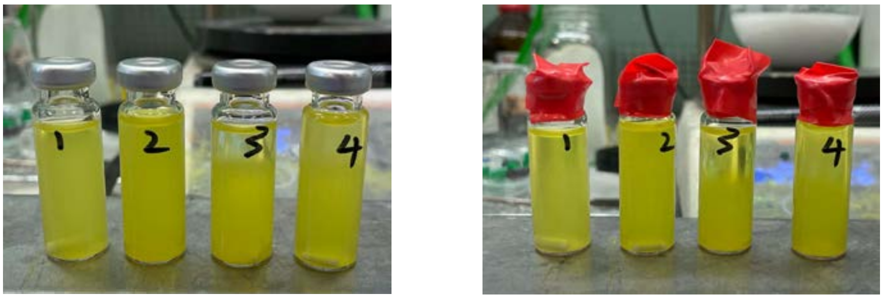

Figure S3. *(Left) Reaction set up (0.1 mmol scale) in a 4 mL reaction vial after sealing with a Teflon-lined metal cap; (Right) Reaction vials sealed with electrical tape*

The sealed reaction vials were placed in a beaker and metal beads were poured to fix the vials in place. The beaker is then placed in the indicated photoreactor set up (*vide supra*) and stirred with a stirrer hotplate affixed overhead for 30 min at –35 °C. The lamp was next switched on, and the reaction was stirred with photoirradiation for 24-36 h at –35 °C (**Figure S4**).

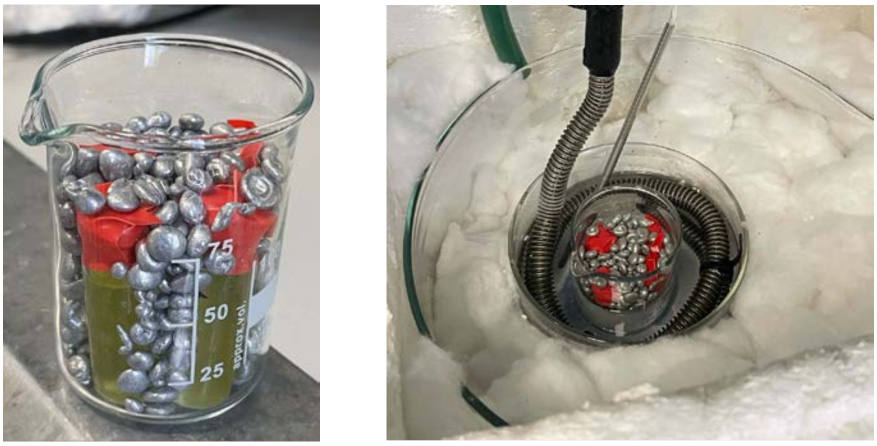

Figure S4. *(Left) vials in beaker with metal beads (Right) beaker in photoreactor set up*

Upon reaction completion, the reaction mixture was filtered through a layer of $MgSO_4$ in a cotton wool-plugged glass pipette. The $MgSO_4$ was rinsed with *i*Pr_2O, and the combined filtrate evaporated to dryness under reduced pressure. Purification by column chromatography (SiO_2, 1:1 CH_2Cl_2:hexanes, first to remove 4CzIPN, then CH_2Cl_2, or EtOAc/hexanes, or acetone/CH_2Cl_2) then affords the enantioenriched hydroxyketone.

N.B. iPr_2O forms a negative azeotrope with MeCN, depressing the boiling point of MeCN to ca. 67.5 °C, and is particularly important to minimize evaporation of volatile hydroxyketone products

General Procedure C1: Enantiomeric excess (ee) determination: benzoylation *via* BzCl

A small sample (*ca.* 1 mg) of the purified hydroxyketone was taken up in a glass pipette and washed into a 2 mL vial with CH_2Cl_2 (1 mL). Et_3N (*ca.* 20 µL), DMAP (tip of a spatula) and BzCl (*ca.* 10 µL) was successively added, and the mixture was reacted for 16 h. Purification *via* preparative thin layer chromatography (pTLC; SiO_2) affords an analytically pure sample of the benzoylated hydroxyketone for spectroscopic characterization and enantiomeric excess determination.

General Procedure C2: Enantiomeric excess (ee) determination: Keck esterification
For non-polar hydroxyketone products, it was necessary to use an alternative esterification method to avoid excess BzCl from coeluting with the benzoate ester derivative of the product.

A small sample (*ca.* 1 mg) of the purified hydroxyketone was taken up in a glass pipette and washed into a 2 mL vial with CH_2Cl_2 (1 mL). BzOH (5.0 mg, 0.04 mmol), DCC (8.3 mg, 0.04 mmol), DMAP (5.0 mg, 0.04 mg) and DMAP·HCl (6.3 mg, 0.04 mg) was successively added to the reaction vial, and the mixture was stirred for 16 h, during which a white precipitate formed. The reaction mixture was filtered over a thin pad of SiO_2 in a glass pipette and the combined filtrate was concentrated under a stream of air. Purification *via* preparative thin layer chromatography (pTLC;

SiO_2) affords an analytically pure sample of the benzoylated hydroxyketone for spectroscopic characterization and enantiomeric excess determination.

2.4. Racemate synthesis

Unless otherwise stated, racemic hydroxyketone synthesis was prepared using the General Procedures outlined below.

General Procedure D1: *mono*-benzoylation *via* BzCl then Dess-Martin oxidation

To a solution of the *meso* diol (0.1 mmol) in CH_2Cl_2 (1 mL) was added BzCl (14 µL, 0.12 mmol), Et_3N (20 µL, 0.15 mmol) and DMAP (tip of spatula). The reaction was stirred for 16 h, after which the mixture was adsorbed onto SiO_2 and the solvent evaporated under reduced pressure. Purification by column chromatography (SiO_2, 10–30% EtOAc/hexanes) afforded the mono-benzoate ester, which was next dissolved in CH_2Cl_2 (1 mL). Dess-Martin Periodinane (2 eq.) and $NaHCO_3$ (3 eq.) was added successively, and the reaction was stirred at r.t. for 2 h. The reaction was diluted with hexanes (1 mL), filtered over a pad of SiO_2 (eluted with CH_2Cl_2) and the solvent removed under a stream of air to afford the racemic benzoylated hydroxyketone.

General Procedure D2: *mono*-benzoylation *via* Keck esterification then Dess-Martin oxidation
For a selection of linear meso diols, the following general procedure was used as it was found that these substrates were poorly reactive with BzCl

To a solution of the *meso* diol (0.1 mmol) in CH_2Cl_2 (1 mL) was added BzOH (18.3 mg, 0.15 mmol), DCC (30.9 mg, 0.15 mmol), DMAP (18.3 mg, 0.15 mmol) and DMAP·HCl (23.8 mg, 0.15 mmol). The reaction was stirred for 16 h, after which the mixture was adsorbed onto SiO_2 and the solvent evaporated under reduced pressure. Purification by column chromatography (SiO_2, 10–30% EtOAc/hexanes) afforded the mono-benzoate ester, which was next dissolved in CH_2Cl_2 (1 mL). Dess-Martin Periodinane (2 eq.) and $NaHCO_3$ (3 eq.) was added successively, and the reaction was stirred at r.t. for 2 h. The reaction was diluted with hexanes (1 mL), filtered over a pad of SiO_2 (eluted with CH_2Cl_2) and the solvent removed under a stream of air to afford the racemic benzoylated hydroxyketone.

General Procedure E: Racemate synthesis (TESCl; Dess-Martin oxidation; deprotection)

To a stirred solution of the *meso* diol (0.1 mmol) in CH_2Cl_2 (1 mL) was added TESCl (14 µL, 0.12 mmol) and imidazole (20 µL, 0.15 mmol). The reaction was stirred for 16 h, after which the reaction mixture was adsorbed onto SiO_2 and the solvent evaporated under reduced pressure. Purification by column chromatography (SiO_2, 2–10% EtOAc/hexanes) affords the mono-TES ether, which was next dissolved in CH_2Cl_2 (1 mL). Dess-Martin Periodinane (2 eq.) and $NaHCO_3$ (3 eq.) was added successively, and the reaction was stirred at r.t. for 2 h. The reaction was next diluted with hexanes (1 mL), filtered over a pad of SiO_2 (eluted with CH_2Cl_2) and the solvent removed under a stream of air to afford the racemic TES-protected hydroxyketone. The resulting compound was dissolved in MeOH (1 mL) and 3 drops of HCl (2 M in MeOH) was added). The reaction was stirred for 16 h before evaporating the mixture to dryness *in vacuo* to obtain the racemic hydroxyketone.

3. Reaction optimization

Reaction optimization commenced by taking conditions competent in the enantioselective diol epimerization (5 mol% of 4CzIPN photocatalyst, 10 mol% of HAA catalyst, acetone as reaction solvent). For all reaction optimization studies, reaction yields quoted are determined by ^{1}H NMR with CH_2Br_2 as an internal standard. All enantiomeric excess (ee) values are quoted after benzoylating the crude product (obtained after filtering the crude material over a SiO_2 pipette column, first eluting with 1:1 CH_2Cl_2/hexanes to remove any photocatalyst, next with 10% EtOAc/CH_2Cl_2 to afford the crude product) *via* General Procedure **C1**.

Initial oxidant screen

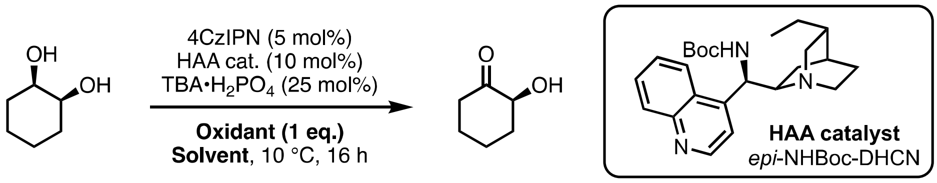

Entry	Oxidant	Solvent (0.025 M)	Product % (SM %)	ee (%)
1	Dichlorohydantoin	Acetone	15% (31%; 12%*)	-
2.	NCS	Acetone	15% (31%; 25%*)	-
3.	Ethyl trichloroacetate	Acetone	4% (75%)	-
4.	SelectFluor®	Acetone	0% (25%; 40%*)	-
5.	Trichlorocyanuric acid (TCCA)	Acetone	45% (10%)	8%
6.	Trichlorocyanuric acid (TCCA) *omitting* HAA cat.	Acetone	40% (12%)	0%
7.	Ethyl trichloroacetate	MeCN	2% (84%)	-
8.	TEMPO	MeCN	10% (70%)	-
9.	$(BzO)_2$	MeCN	14% (60%)	-
10.	Dicumyl peroxide	MeCN	11% (78%)	-
11.	BzOO*t*Bu	MeCN	18% (43%)	78%
12.	$K_2S_2O_8$	MeCN	6% (69%)	-
**13.**	**DIAD**	**MeCN**	**54% (20%)**	**81%**
14.	DIAD	Acetone	44% (47%)	76%
**15.**	**Air**	**MeCN**	**32% (56%)**	**80%**
16.	Air	Acetone	22% (62%)	76%

Note: for all reactions, N_2 was purged through the system after addition of all reagents in the reaction vial. For Entries 15 and 16, a needle was pierced through the top of the vial and a fan was positioned directly above to force air into the reaction vial.
* formation of the dimethyl acetonide (i.e. acetal formation with acetone) derivative of starting material observed

Initial screen revealed that DIAD and air are competent oxidants, and that the reaction performs better in acetonitrile over acetone (optimal solvent in enantioselective epimerization). Of note, TCCA performs well, however this is driven by a HAA catalyst independent background reaction (entry 5 vs. entry 6).

Reaction time

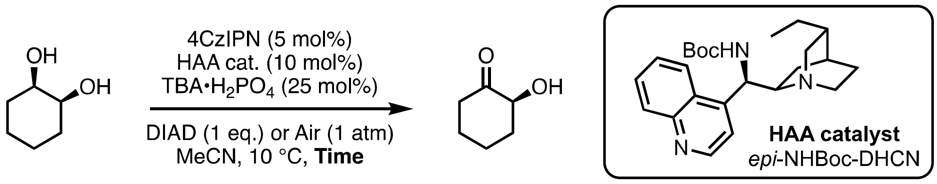

Entry	Oxidant	Time	Product % (SM %)	ee (%)
1	DIAD	16 h	54% (20%)	82%
**2.**	**DIAD**	**24 h**	**62% (27%)**	**81%**
3.	DIAD	36 h	58% (28%)	82%
4.	DIAD	48 h	59% (34%)	-
5.	Air	16 h	32% (56%)	80%
6.	Air	24 h	18% (63%)	-
7.	Air	36 h	28% (41%)	77%
8.	Air	48 h	28% (42%)	-

For all reactions, N_2 was purged through the system after addition of all reagents in the reaction vial. For Entries 5–8, a needle was pierced through the top of the vial and a fan was positioned directly above to force air into the reaction vial.

These results indicated that the reaction was complete by 24 h for our model substrate, and that enantioselectivity did not change/erode over time, providing evidence against further reaction of the hydroxyketone. These results also indicated that air was a capricious oxidant for the reaction.

Further oxidant screen

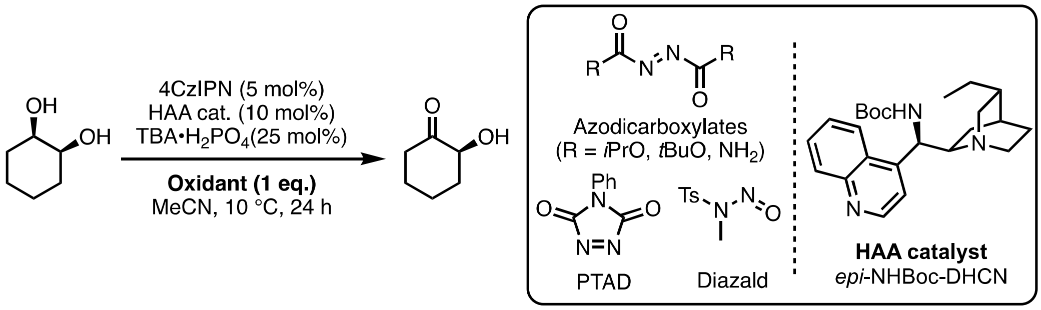

Entry	Oxidant	Product % (SM %)	ee (%)
**1**	**R = *i*PrO (DIAD)**	**62% (27%)**	**81%**
2.	R = *t*BuO (DTAD)	46% (39%)	81%
3.	R = NH_2 (ACDA)	28% (42%)	80%
4.	PTAD	0% (75%)	-
5.	Diazald	3% (64%)	-
7.	O_2 (1 atm, static)	20% (77%)	81%

For all reactions, N_2 was purged through the system after addition of all reagents in the reaction vial.

DIAD remains the best oxidant. DEAD (R = EtO) was not screened due to poor availability of the azodicarboxylate reagent in our laboratory.

Further screen of reaction parameters: additives and photocatalyst (DIAD as oxidant)

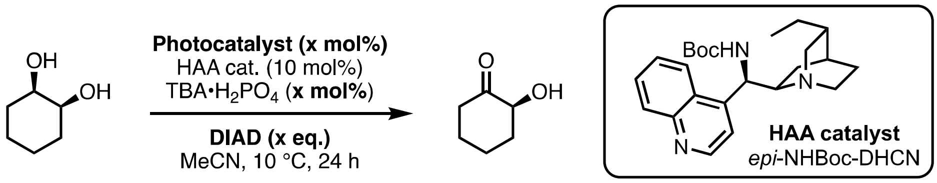

Entry	Oxidant	Changes to condition	Product % (SM %)	ee (%)
Additives				
1	DIAD	No TBA H_2PO_4	2% (71%)	-
2.	DIAD	TBA H_2PO_4 (15 mol%)	42% (32%)	-
**3.**	**DIAD**	**TBA H_2PO_4 (25 mol%)**	**62% (27%)**	**81%**
4.	DIAD	TBA H_2PO_4 (40 mol%)	44% (25%)	70%
Photocatalyst				
**5.**	**DIAD**	**4CzIPN (5 mol%)**	**62% (27%)**	**81%**
6.	DIAD	4CzIPN (7.5 mol%)	60% (13%)	80%
7.	DIAD	Ir(dF(CF$_3$)ppy$_2$)dtbppy PF_6 (1 mol%)	23% (73%)	-
8.	DIAD	3CzClIPN (5 mol%)	40% (26%)	77%
9.	DIAD	T-Ph-perylium (5 mol%)	16% (67%)	-
Equivalents				
10.	DIAD	1.2 eq DIAD	44% (38%)	72%

TBA H_2PO_4 is an essential additive for reactivity at +10 °C. Higher loadings of TBA H_2PO_4 results in eroded enantioselectivity. No beneficial increase in yield and ee on higher 4CzIPN loadings, and it appears that 4CzIPN is the best performing photocatalyst for this reaction

Further screen of reaction parameters: temperature (DIAD as oxidant)

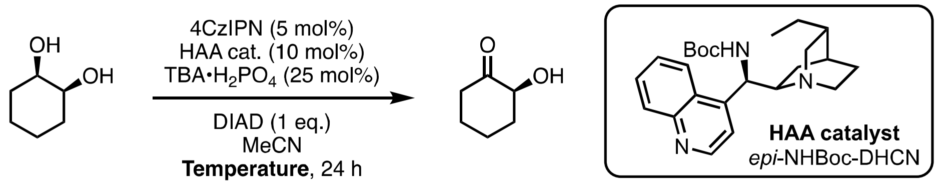

Entry	Oxidant	Changes to condition	Product % (SM %)	ee (%)
Temperature				
1.	DIAD	T = +10 °C	62% (27%)	81%
**2.**	**DIAD**	**T = –35 °C**	**78% (1%)**	**91%**

*0.05 mmol scale in 4 mL vial.

Note: for all reactions, N_2 was purged through the system after addition of all reagents in the reaction vial.

Marked increase in reaction yield, enantioselectivity and a much cleaner reaction profile was observed when reducing the reaction temperature to –35 °C, in line with our previous report. Note that we could not carry reactions at temperatures lower than –35 °C as MeCN freezes at –45 °C.

Further screen of reaction parameters: additives (DIAD as oxidant)

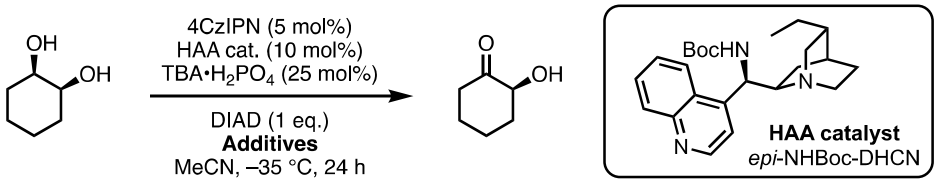

Entry	Oxidant	Changes to condition	Product % (SM %)	ee (%)
1.	DIAD	T = –35 °C<br>No exclusion of air	71% (0%)	91%
2.	DIAD	T = –35 °C<br>No exclusion of air<br>HAA cat (15 mol%)	72% (9%)	-
3.	DIAD	T = –35 °C<br>No exclusion of air<br>+ $MgSO_4$ (20 mg)	80% (2%)	90%
**4.**	**DIAD**	**T = –35 °C<br>No exclusion of air<br>+ 4 Å MS (20 mg)**	**96% (0%)<br>92% isolated**	**91%**
5.	DIAD	T = –35 °C<br>No exclusion of air<br>+ 4 Å MS (20 mg)<br>Acetone as solvent	57% (38%)	72%

Reaction yield and enantioselectivity was not impaired when the reaction is set up open to air (i.e. no N_2 purge). Increasing HAA catalyst loading did not benefit reaction, and yield improvements was observed on addition of drying agents in the reaction, with the best performing drying agent being 4 Å molecular sieves. Acetone performs poorer than acetonitrile in this reaction.

Control reactions at –35 °C (DIAD as oxidant)

Optimized conditions:

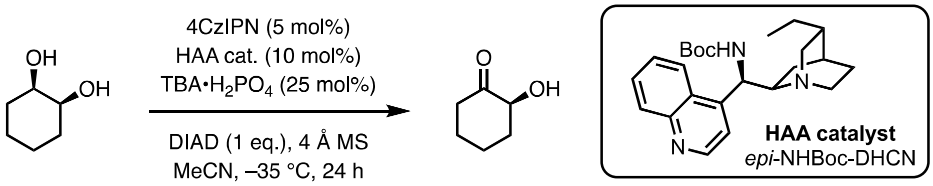

Entry	Oxidant	Changes to condition	Product % (SM %)	ee (%)
**1.**	**DIAD**	**None**	**96%, 92% isolated (0%)**	**91%**
2.	DIAD	No Blue LED irradiation	0% (71%)	-
3.	DIAD	No 4CzIPN	0% (88%)	-
4.	DIAD	No HAA catalyst	0% (78%)	-
5.	DIAD	No DIAD	8% (78%)	-
6.	DIAD	No TBA H_2PO_4	*ca.* 48% (44%)<br>*messy reaction profile*	74%

4. Synthesis and characterization of starting materials

4.1. List of known substrates

Aside from commercially-available substrates, all known substrates are synthesized according to General Procedure **A** (stereospecific dihydroxylation) from the corresponding cyclic or acyclic *cis* alkenes starting material. References for known *meso* diols are provided below. Procedures for the synthesis known diols that differ substantially from literature reports are detailed in the section below. Characterization data are provided for published diol substrates that have incomplete data associated with the published structure.

		Reference
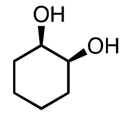	**1a**	Commercially available
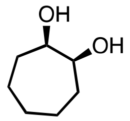	**1b**	*J. Am. Chem. Soc.*, **1999**, *121*, 10702–10710
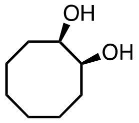	**1c**	*J. Org. Chem.*, **1990**, *55*, 766–768
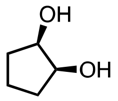	**1d**	Commercially available
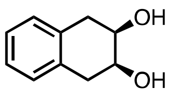	**1e**	*J. Am. Chem. Soc.*, **2022**, *144*, 93–98
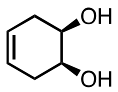	**1k**	*Org. Biomol. Chem.*, **2003**, *1*, 2173–2186
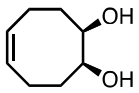	**1l**	*J. Org. Chem.*, **1994**, *59*, 2848–2876
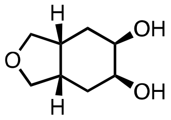	**1m**	*Heterocycles*, **2000**, *52*, 261–272
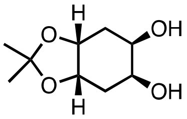	**1o**	*J. Org. Chem.*, **1994**, *59*, 2848–2876
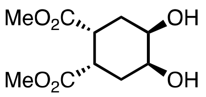	**1y**	*Synthesis*, **2017**, *49*, 1206–1213

		Reference
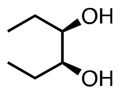	**3a**	*J. Am. Chem. Soc*, **2022**, *144*, 93–98
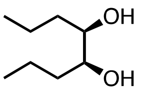	**3b**	*Angew. Chem. Int. Ed.*, **2021**, *60*, 13819 - 13823
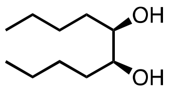	**3c**	*J. Org. Chem.*, **1978**, *43*, 3255 - 3266
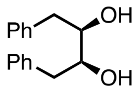	**3d**	*J. Org. Chem.*, **1984**, *49*, 4706–4711
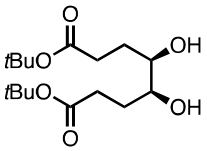	**3f**	*Molecules*, **2021**, *26*, 3974
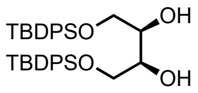	**3j**	*Eur. J. Org. Chem.* **2020**, *30*, 4775–4786
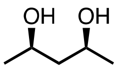	**3l**	Commercially available; separated by chromatography

4.2. Synthesis and characterization of new *meso* diols

Unless otherwise stated, General Procedure **A** (stereospecific dihydroxylation) was used to synthesize all novel diols from their corresponding cyclic or acyclic *cis*-alkenes.

(4*S*,5*R*)-1-tosylazepane-4,5-diol (1f)

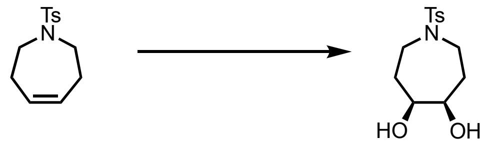

S-1f 2 steps (known) **1f**

S-1f (338 mg, 1.34 mmol, prepared in two steps according to literature procedures[3]) was subjected to General Procedure **A** using $K_2OsO_4{\cdot}2H_2O$ (10.0 mg, 0.027 mmol) and NMO (0.34 mL, 1.61 mmol). After reaction completion, the reaction was quenched with sat. Na_2SO_3 (100 µL), and the reaction mixture concentrated to dryness over SiO_2 under reduced pressure. Purification of the crude product (adsorbed onto SiO_2) by column chromatography (SiO_2, 10–20% acetone/hexanes) gave **1f** as a white solid (302 mg, 1.05 mmol, 79%).

^{1}H NMR (400 MHz, $CDCl_3$) δ 7.65 (d, *J* = 8.4 Hz, 2H), 7.30 (d, *J* = 7.9 Hz, 2H), 3.95 (d, *J* = 6.0 Hz, 2H), 3.42 (ddd, *J* = 12.6, 8.0, 4.1 Hz, 2H), 3.19 (ddd, *J* = 13.6, 7.8, 3.7 Hz, 2H), 2.42 (s, 3H), 2.21 (br s, 2H), 2.06 (dtd, *J* = 15.5, 7.8, 3.7 Hz, 2H), 1.87 – 1.71 (m, 2H).
^{13}C NMR (126 MHz, $CDCl_3$) δ 143.5, 135.8, 129.9 127.2, 72.5, 42.2, 31.1, 21.7.

NMR in agreement with the previously reported data.[4]

Diethyl (4R,5S)-4,5-dihydroxycycloheptane-1,1-dicarboxylate (1g)

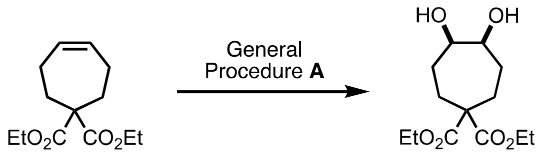

S-1g **1g**

Prepared according to General Procedure **A** using alkene **S-1g** (800 mg, 3.33 mmol), NMO (0.83 mL, 4.0 mmol) and $K_2OsO_4{\cdot}2H_2O$ (24.5 mg, 0.067 mmol). Purification by column chromatography (SiO_2, 40% acetone/hexanes) afforded **1g** (739 mg, 2.69 mmol, 80%) as a white solid.

^{1}H NMR (700 MHz, $CDCl_3$) δ 4.15 (qd, *J* = 7.1, 4.9 Hz, 4H), 3.78 (m, *J* = 7.2 Hz, 2H), 2.41 (bs, 2H), 2.39 – 2.35 (m, 2H), 1.93 – 1.84 (m, 4H), 1.64 (t, *J* = 13.7, Hz, 2H), 1.22 (td, *J* = 7.1, 4.7 Hz, 6H).
^{13}C NMR (101 MHz, $CDCl_3$) δ 172.8, 172.7, 73.0, 61.5, 61.4, 56.7, 26.7, 25.7, 14.1.

NMR in agreement with the previously reported data.[5]

((4R,5S)-4,5-dihydroxycycloheptane-1,1-diyl)bis(methylene) dibenzoate (1h)

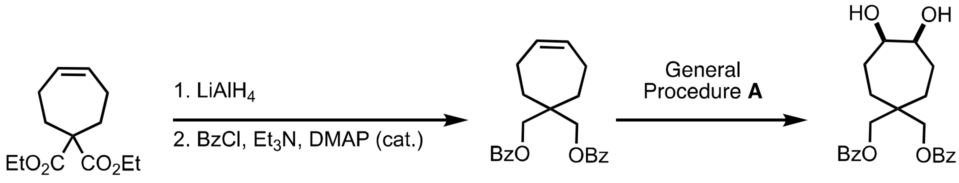

S-1g **S-1h** **1h**

Crude diol (200 mg, 1.28 mmol, obtained from the $LiAlH_4$ reduction of **S-1g**; *vide supra*) was dissolved in CH_2Cl_2 (10 mL). Et_3N (0.54 mL, 3.84 mmol) and BzCl (0.37 mL, 3.20 mmol) was successively added, and the reaction mixture was stirred at r.t. for 20 h. The reaction mixture was quenched with H_2O, the phases separated and the aqueous phase extracted with CH_2Cl_2. The combined organic extracts were dried ($MgSO_4$), filtered, and the solvent was removed under reduced pressure. Purification of the crude product by column chromatography (SiO_2, 10% EtOAc/hexanes) gave **S-1h** as a white solid (337 mg, 0.92 mmol, 72%) in good spectroscopic agreement with previously reported data.[6]

^{1}**H NMR** (400 MHz, CDCl3) δ 8.00 (dd, J = 8.4, 1.4 Hz, 4H), 7.52–7.47 (m, 2H), 7.37 (t, J = 7.7 Hz, 4H), 5.65 (t, J = 2.9 Hz, 2H), 4.35 (s, 4H), 2.24–2.21 (m, 4H), 1.81–1.72 (m, 4H).

S-1h (337 mg, 0.92 mmol) was subjected to General Procedure **A** with NMO (0.23 mL, 1.10 mmol) and $K_2OsO_4 \cdot 2H_2O$ (6.8 mg, 0.018 mmol). Purification by column chromatography (SiO_2, 20% acetone/hexanes) afforded **1h** (226 mg, 0.57 mmol, 61% yield) as a white solid.

^{1}**H NMR** (400 MHz, $CDCl_3$) δ 8.02–7.99 (m, 4H), 7.55 (tdd, *J* = 7.0, 2.9, 1.5 Hz, 2H), 7.42 (t, *J* = 7.8 Hz, 4H), 4.28 (s, 2H), 4.24 (s, 2H), 3.84 (d, *J* = 7.9 Hz, 2H), 2.17 (br s, 2H), 2.01–1.94 (m, 4H), 1.65–1.61 (m, 2H), 1.39 (dd, *J* = 15.4, 9.8 Hz, 2H).
^{13}**C NMR** (176 MHz, $CDCl_3$) δ 166.7, 166.6, 133.3, 133.2, 130.2, 130.1, 129.7, 129.7, 128.6, 128.6, 73.3, 68.0, 67.95, 40.2, 25.3, 24.1.
HRMS (ESI+) $[M+Na]^+$ m/z calc'd for $[C_{23}H_{26}O_6Na]^+$, expect 421.1622, found 421.1617.

((4*R*,5*S*)-4,5-dihydroxycycloheptane-1,1-diyl)bis(methylene) dibenzoate (1i)

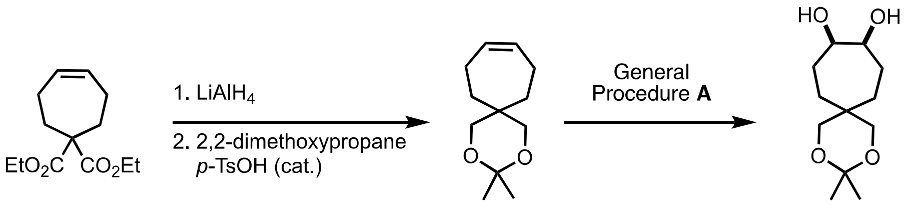

S-1g **S-1i** **1i**

*Alkene **S-1i** synthesis from **S-1g***
$LiAlH_4$ (2.29 mL, 9.16 mmol, 4.0 M in THF) was added dropwise to a stirred solution of **S-1g** (1.00 g, 4.16 mmol) in THF (10 mL) at 0 °C under an atmosphere of N_2. The reaction was stirred at 0 °C for 1 h and then allowed to warm to r.t. over 16 h. The reaction was cooled to 0 °C, and carefully

quenched with sat. NH_4Cl and diluted with H_2O (30 mL). The phases were separated and the aqueous phase extracted with EtOAc (3 x 20 mL). The combined organic extracts were dried ($MgSO_4$), filtered and the solvent removed under reduced pressure to afford crude diol (543 mg, 3.5 mmol).

Crude diol (300 mg, 1.92 mmol), which next dissolved in DMF (7 mL). 2,2-dimethoxypropane (1.2 mL, 9.60 mmol) and *p*TsOH·H_2O (37 mg, 0.19 mmol) was added successively, and the reaction was stirred at r.t. for 24 h. The mixture was diluted with H_2O (50 mL) and extracted with EtOAc (3 x 20 mL). The combined organic extracts were dried ($MgSO_4$), filtered and the solvent removed under reduced pressure to afford the crude acetonide **S-1i** (*ca.* 320 mg, 1.63 mmol).

Crude **S-1i** (320 mg, 1.63 mmol) was subjected to General Procedure **A** with NMO (0.41 mL, 1.96 mmol) and $K_2OsO_4 \cdot 2H_2O$ (12 mg, 0.032 mmol). Purification by column chromatography (SiO_2, 30% acetone/hexanes) afforded **1i** (286 mg, 1.24 mmol, 76%) as a white solid.

^{1}H NMR (700 MHz, $CDCl_3$) δ 3.76 (d, *J* = 7.0 Hz, 2H), 3.50 (s, 4H), 2.32 (br s, 2H), 1.84 (dd, *J* = 15.2, 10.2 Hz, 2H), 1.80–1.71 (m, 2H), 1.51–1.44 (m, 2H), 1.39 (s, 6H), 1.19 (dd, *J* = 15.1, 9.8 Hz, 2H).
^{13}C NMR (176 MHz, $CDCl_3$) δ 98.20, 73.4, 70.4, 68.9, 35.1, 25.5, 25.4, 23.9.
HRMS (ESI+) $[M+Na]^+$ m/z calc'd for $[C_{12}H_{22}O_4Na]^+$, expect 253.1410, found: 253.1415.

(8*R*,9*S*)-1,4-dioxaspiro[4.6]undecane-8,9-diol (1j)

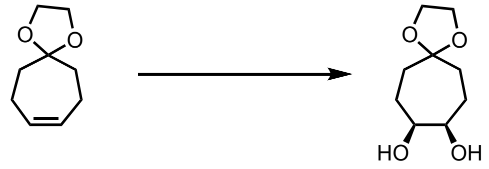

S-1j
4 steps (known)

1j

S-1j (265 mg, 1.72 mmol; prepared in four steps according to literature procedures[7]) was subjected to General Procedure **A** using $K_2OsO_4 \cdot 2H_2O$ (12.0 mg, 0.034 mmol) and NMO (0.43 mL, 2.00 mmol). After reaction completion, the reaction was quenched with sat. Na_2SO_3 (100 µL), and the reaction mixture concentrated to dryness over SiO_2 under reduced pressure. Purification of the crude product (adsorbed onto SiO_2) by column chromatography (SiO_2, 10–20% acetone/hexanes) gave **1j** as a white solid (148 mg, 0.78 mmol, 48%).

^{1}H NMR (700 MHz, $CDCl_3$) δ 3.95 – 3.88 (m, 4H), 3.83 (dq, *J* = 6.1, 2.9 Hz, 2H), 2.38 (d, *J* = 5.0 Hz, 2H), 2.02 (ddd, *J* = 15.4, 9.5, 2.4 Hz, 2H), 1.95 – 1.85 (m, 2H), 1.71 – 1.63 (m, 4H).
^{13}C NMR (176 MHz, $CDCl_3$) δ 112.1, 73.6, 64.3, 64.2, 31.9, 26.3.

NMR in agreement with the previously reported data.[8]

tert-Butyl (3a*R*,5*R*,6*S*,7a*S*)-5,6-dihydroxyoctahydro-2*H*-isoindole-2-carboxylate (1n)

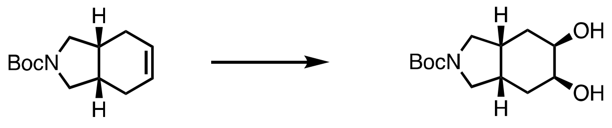

S-1n
2 steps (known)

1n

Synthesis of **1n** reported in our previous publication (**Ref 2**). In brief, **S-1n** (1.37 g, 6.13 mmol; prepared in two steps according to literature procedures[9]) was subjected to General Procedure A using K_2OsO_4·$2H_2O$ (45.0 mg, 0.12 mmol) and NMO (1.5 mL, 7.36 mmol). After reaction completion, the reaction was quenched with Na_2SO_3, extracted with EtOAc (4 x 20 mL) and the combined organic phases dried ($MgSO_4$) and filtered over a pad of SiO_2. At this point, crude NMR analysis was taken, which indicated a single product diastereomer. The combined filtrate was concentrated under reduced pressure until precipitation of a white solid was observed. The precipitate was collected by filtration and washed with hexanes to afford **1n** as a white solid (510 mg, 1.98 mmol, 32%) as a single diastereomer.

^{1}H NMR (400 MHz, d_3-MeOD) δ 3.81 (dd, *J* = 6.1, 1.8 Hz, 2H), 3.41–3.33 (m, 2H), 3.16 (dt, *J* = 10.3, 4.5 Hz, 2H), 2.48–2.38 (m, 2H), 1.93–1.84 (m, 2H), 1.60–1.51 (m, 2H), 1.45 (s, 9H).
^{13}C NMR (101 MHz, d_3-MeOD) δ 155.5, 79.4, 67.8, (49.5, 49.0)*, (34.5, 33.8)*, 29.0, 27.3.
Apparent splitting of ^{13}C NMR signals arising from hindered rotation/rotamerism of the amide bond denoted by (*)
HRMS (ESI+) $[M+Na]^+$ m/z calc'd for $[C_{13}H_{23}NO_4Na]^+$ expect 280.1525; found 280.1522

Dimethyl (3a*R*,5*R*,6*S*,7a*S*)-5,6-dihydroxyoctahydro-2*H*-indene-2,2-dicarboxylate (1p)

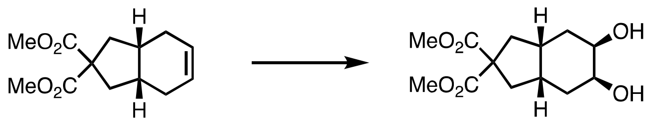

S-1p
4 steps (known)

1p

Synthesis of **1p** reported in our previous publication.[2] In brief, alkene **S-1p** (1.19 g, 5.00 mmol; prepared in four steps according to literature procedures[10]) was subjected to General Procedure A using K_2OsO_4·$2H_2O$ (36.8 mg, 0.10 mmol) and NMO (1.2 mL, 6.00 mmol) to afford a single *syn*-diol diastereomer. Purification by column chromatography (SiO_2, 20% acetone/hexane) afforded the product **1p** (402 mg, 1.47 mmol, 29%) as a white solid.

^{1}H NMR (700 MHz, $CDCl_3$) δ 3.86 (dd, *J* = 6.1, 2.7 Hz, 2H), 3.72 (s, 3H), 3.72 (s, 3H), 2.37 (dd, *J* = 13.7, 7.4 Hz, 2H), 2.34–2.27 (m, 2H), 2.11 (d, *J* = 6.5 Hz, 2H), 1.82 (m, 4H).
^{13}C NMR (176 MHz, $CDCl_3$) δ 173.6, 173.4, 68.6, 58.9, 52.8, 52.8, 37.9, 35.7, 30.5.
HRMS (ESI+) $[M+H]^+$ m/z calc'd for $[C_{13}H_{20}O_6H]^+$ expect 273.1338; found 273.1335

Ethyl (2*r*,3a*R*,5*R*,6*S*,7a*S*)-2-cyano-5,6-dihydroxyoctahydro-1*H*-indene-2-carboxylate (1q)
Ethyl (2*s*,3a*R*,5*R*,6*S*,7a*S*)-2-cyano-5,6-dihydroxyoctahydro-1*H*-indene-2-carboxylate (1r)
Ethyl (3a*R*,5*S*,6*R*,7a*S*)-2-cyano-5,6-dihydroxyoctahydro-1*H*-indene-2-carboxylate (1s)

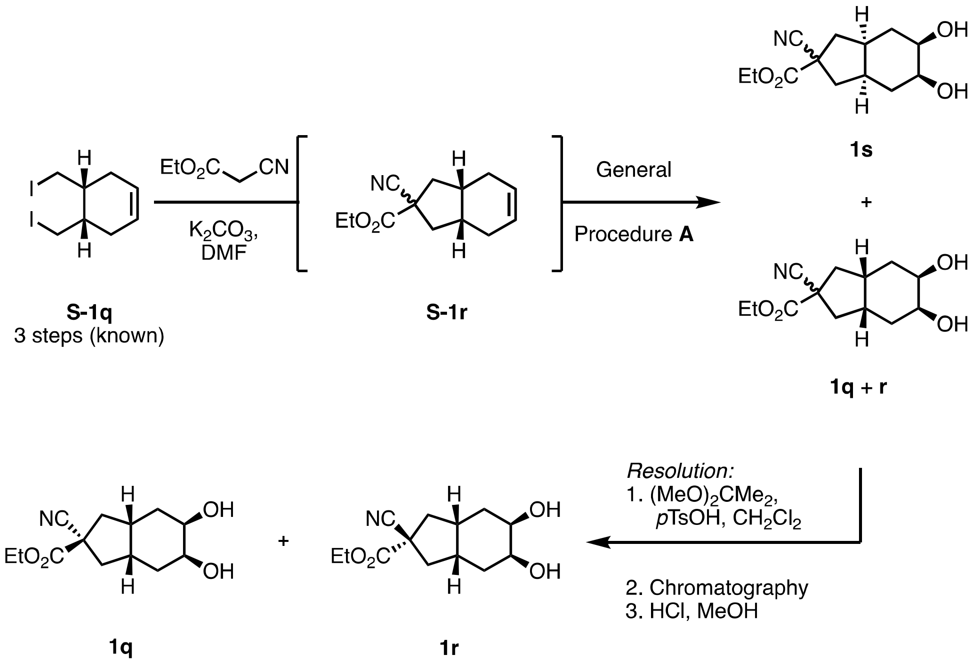

K_2CO_3 was added to a stirred solution of diiodide **S-1q** (2.36 g, 6.52 mmol, prepared in three steps according literature procedures[11]) and ethyl cyanoacetate (1.30 mL, 13.3 mmol) in DMF (15 mL). The mixture was stirred at 60 °C for 16 h before diluting with water (60 mL) and extracted with Et_2O (4 x 20 mL). The combined organic extracts were successively washed with sat. NH_4Cl (2 x 20 mL) and brine (20 mL), dried ($MgSO_4$) and filtered. The filtrate was concentrated under reduced pressure to afford the crude alkene **S-1r** as a clear oil (*ca.* 1.03 g, 4.70 mmol, 72%) as an inseparable mixture of diastereomers. Crude **S-1r** was used in the next step without further purification.

Crude **S-1r** (1.03 g, 4.70 mmol) was subjected to General Procedure **A** using $K_2OsO_4{\cdot}2H_2O$ (34.0 mg, 0.09 mmol) and NMO (1.16 mL, 5.60 mmol). Purification by column chromatography (SiO_2, 20–30% acetone/hexane) afforded two products: **1s** (100 mg, 0.39 mmol, 8%); diol resultant from concave face dihydroxylation, and a mixture of **1q** and **1r** (470 mg, 1.86 mmol, 40%); diol resultant from convex face dihydroxylation (*dr* = 5:1 relative to fused ring system).

Resolution of 1q and 1r

The mixture of **1q** and **1r** (in 262 mg, 1.00 mmol batches) was next subjected to modified General Procedure **A'**, using *p*-TsOH (17 mg, 0.1 mmol), 2,2-dimethoxypropane (612 μL, 5.00 mmol) in CH_2Cl_2 (1.5 mL), to resolve the diastereomeric mixture at the quaternary -CO_2Et/-CN bearing stereocenter. Purification by column chromatography (SiO_2, 10% EtOAc/hexane) resolved the diastereomerically pure acetonide-protected product **1q'** (111 mg, 0.378 mmol, 38%) and **1r'** (126

mg, 0.430 mmol, 43%). Resolution of **1s** was not attempted due to the limited amount of material obtained.

The combined chromatographically-separated acetonide **1q'** and **1r'** was next subjected to acidic methanolysis conditions as outlined in General Procedure **A'**, to afford the diastereomerically enriched diols **1q** (217 mg, 0.856 mmol, 18% relative to **S-1r**) and **1r** (224 mg, 0.884 mmol, 19% relative to **S-1r**, contains 7% **1q** as minor diastereomer) as off white solids.

Characterization data for **1q**
^{1}H NMR (700 MHz, $CDCl_3$) δ 4.26 (q, *J* = 7.1 Hz, 2H), 3.91 (dd, *J* = 6.0, 3.0 Hz, 2H), 2.47 (d, *J* = 9.1 Hz, 4H), 2.21 (q, *J* = 8.9 Hz, 2H), 2.00–1.92 (m, 2H), 1.87 (br s, 2H), 1.33 (t, *J* = 7.1 Hz, 3H).
^{13}C NMR (176 MHz, $CDCl_3$) δ 170.2, 122.3, 68.2, 63.0, 44.7, 41.5, 36.5, 30.0, 14.0.
HRMS (ESI+) $[M+Na]^+$ m/z calc'd for $[C_{13}H_{19}NO_4Na]^+$ expect 276.1212; found 276.1209

Characterization data for **1r**
^{1}H NMR (700 MHz, $CDCl_3$) δ 4.30 (q, *J* = 7.1 Hz, 2H), 3.89 (dd, *J* = 6.0, 3.0 Hz, 2H), 2.55 (h, *J* = 6.6 Hz, 2H), 2.43 (dd, *J* = 13.8, 7.2 Hz, 2H), 2.23 (dd, *J* = 13.8, 7.1 Hz, 2H), 1.95 (dt, *J* = 13.7, 7.2 Hz, 2H), 1.67 (dq, *J* = 10.6, 3.9, 3.4 Hz, 3H), 1.36 (t, *J* = 7.1 Hz, 3H).
^{13}C NMR (176 MHz, $CDCl_3$) δ 170.1, 121.8, 68.4, 63.1, 46.5, 41.4, 35.8, 30.3, 14.0.
HRMS (ESI+) $[M+Na]^+$ m/z calc'd for $[C_{13}H_{19}NO_4Na]^+$ expect 276.1212; found 276.1210

Characterization data for **1s**
^{1}H NMR (700 MHz, $CDCl_3$) δ 4.28–4.23 (m, 2H), 3.82 (m, 2H), 2.58 (dd, *J* = 13.9, 6.5 Hz, 1.2H), 2.52* (dd, *J* = 13.9, 7.1 Hz, 0.9H), 2.47 (dd, *J* = 13.8, 7.3 Hz, 1.2H), 2.42 (dd, *J* = 13.8, 7.1 Hz, 0.9H), 2.29* (m, 0.9H), 2.20 (m, 1.2H), 2.04 (br s, 1.2H), 1.98 (br s, 0.9H), 1.97–1.89 (m, 2H), 1.74 – 1.60 (m, 2H), 1.34–1.30 (m, 3H).
Signals marked * denote resonances arising from epimeric diastereomer at the quaternary center in **1s**, which can be visually distinguished based on relative intensity (*dr = ca.* 1:1.3 by ^{1}H NMR)
^{13}C NMR (176 MHz, $CDCl_3$) δ 170.6, 169.9*, 122.2, 122.1*, 69.9, 69.8*, 62.9*, 62.9, 46.7, 44.5*, 42.5, 42.5*, 37.9, 37.3*, 30.2*, 29.9, 14.0*, 14.0.
Signals marked * denote resonances arising from epimeric diastereomer at the quaternary center in **1s**, which can be visually distinguished based on relative intensity
HRMS (ESI+) $[M+Na]^+$ m/z calc'd for $[C_{13}H_{19}NO_4Na]^+$ expect 276.1212; found 276.1210

Stereochemical assignment of 1q and 1r

The relative configuration between the diol and 6,5-ring fusion was unambiguously confirmed through NOE analysis of **1q** and **1r**, which both exhibited NOE correlation between the carbinol methine proton with protons on the cyclopentyl motif (**Figure S5** and **Figure S6**). This is only possible if the diol and cyclopentyl motif were anti disposed to each other. By method of elimination, this leaves **1s** as the alternative *syn* diol diastereomer containing an epimeric mixture at the quaternary stereocenter.

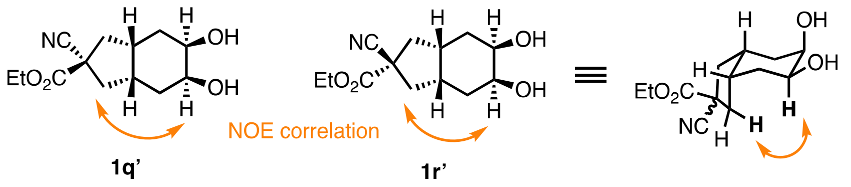

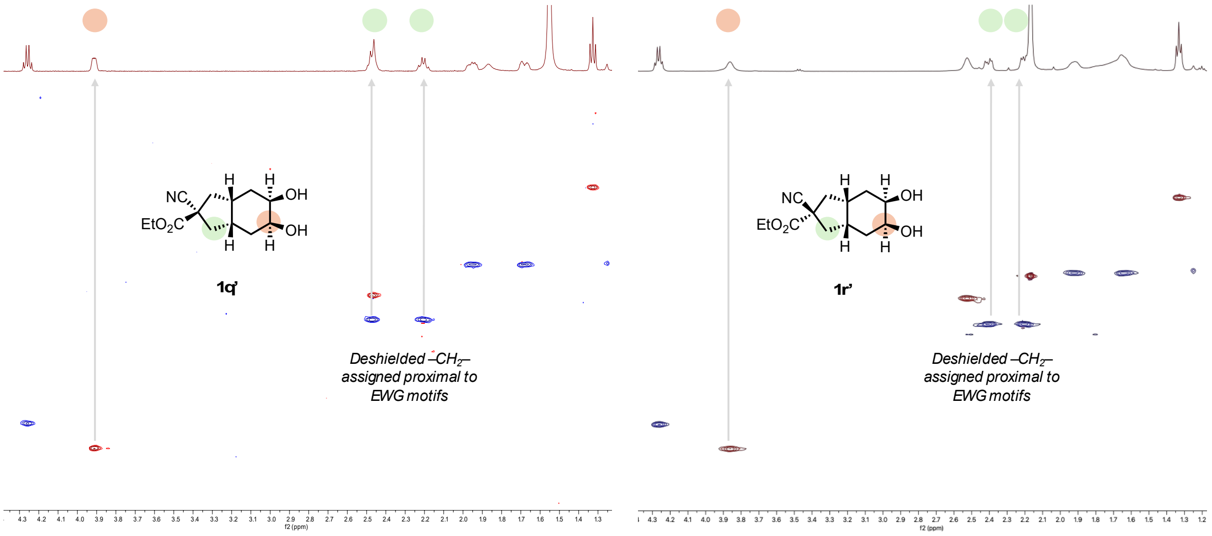

Figure S5. ^{1}H-^{13}C *HSQC assignment of carbinol methine and cyclopentyl protons for* ***1q'*** *(left) and* ***1r'*** *(right)*

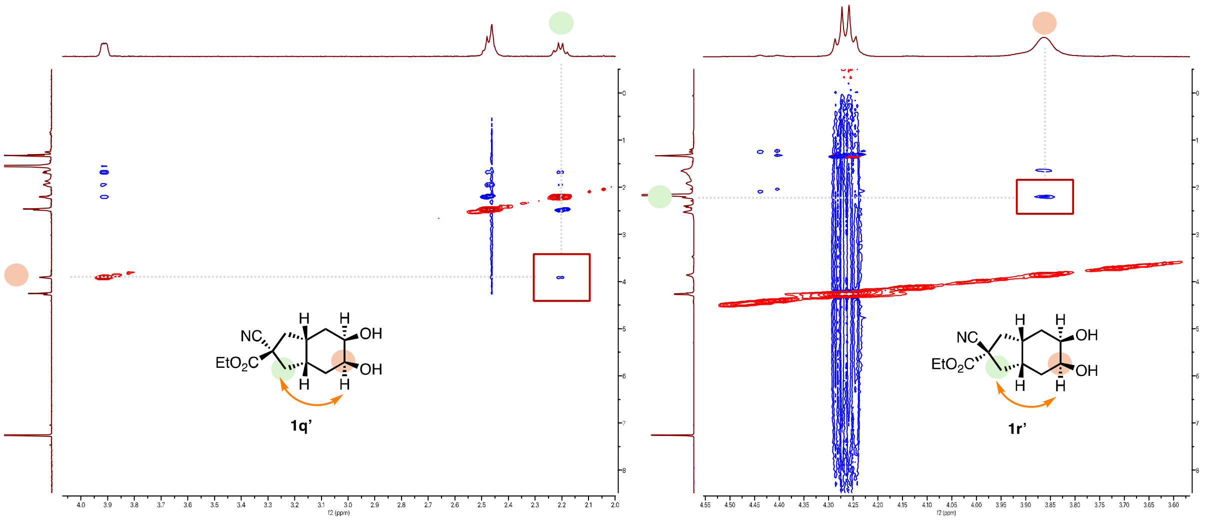

Figure S6. Key NOE correlations for ***1q'*** *(left) and* ***1r'*** *(right) enabling unambiguous assignment of syn OH relative to cyclopentyl motif*

To establish the relative configuration of **1q'** and **1r'** (and hence **1q** and **1r**), acetonides **1q'** and **1r'** (containing ethyl esters) was reduced to the corresponding primary alcohol to facilitate NOE analysis. A small sample of acetonides **1q'** or **1r'** (5 mg, 0.017 mmol) was dissolved in THF (1 mL) at 0 °C. A solution of $LiBH_4$ (20 μL, 0.080 mmol, 4 M in THF) was next added and the reaction was stirred at 0 °C for 3 h. MeOH (100 μL) was next added, and the reaction was concentrated to dryness under a stream of air. The crude reaction mixtures were then spectroscopically analyzed to deduce the relative configuration of **1q"** and **1r"**. Strong NOE correlations were observed for **1q"**; the corresponding signals in **1r"** saw no NOE correlation, giving conclusive evidence of the relative configuration in **1q** and **1r** (**Figure S7** and **Figure S8**).

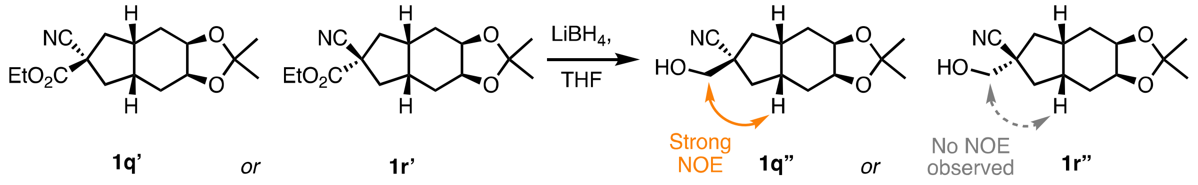

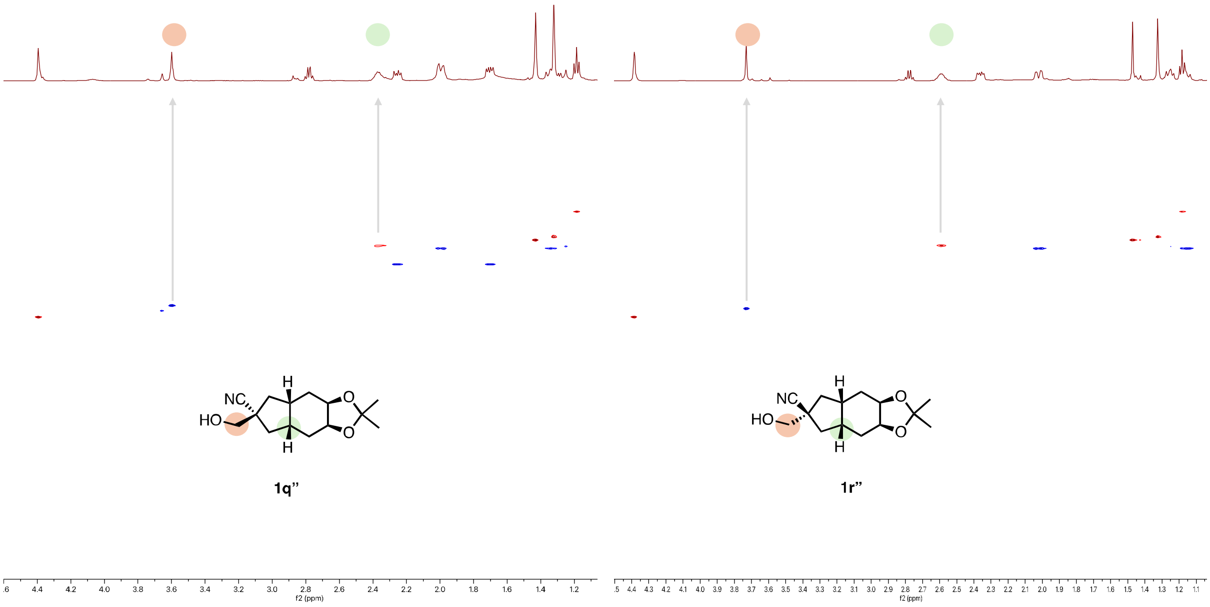

Figure S7. ^1H-^{13}C HSQC assignment of –C$\underline{H}_2$OH and bridging protons for **1q"** (left) and **1r"** (right)

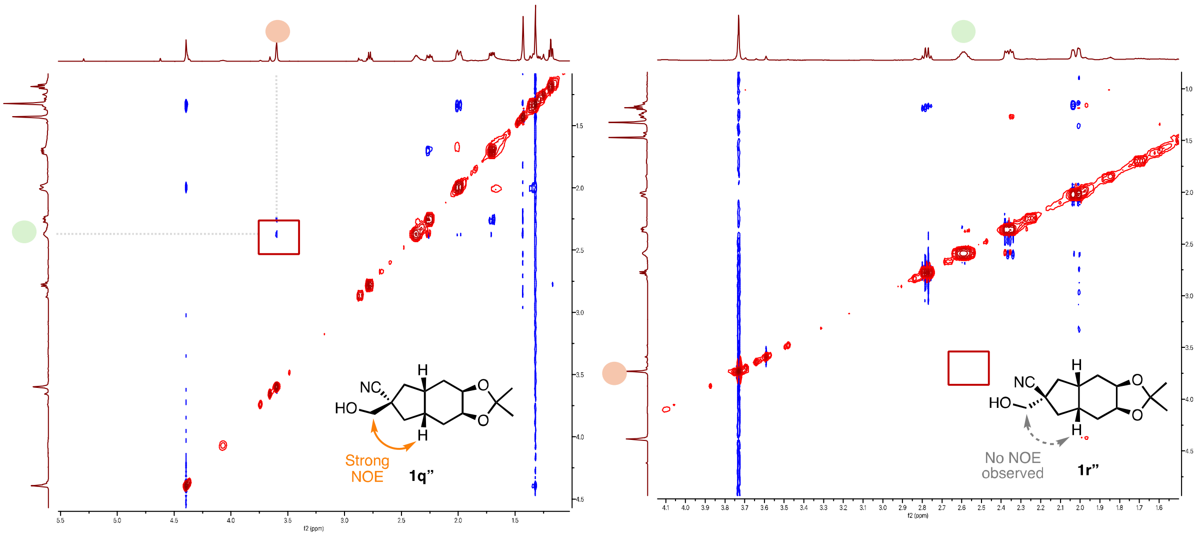

Figure S8. Key NOE correlations for **1q"** (left) and the absence of which in **1r"** (right) for unambiguous assignment

(5a*R*,7*R*,8*S*,9a*S*)-3,3-dimethyloctahydrobenzo[e][1,3]dioxepine-7,8-diol (1t) and (5a*R*,7*S*,8*R*,9a*S*)-3,3-dimethyloctahydrobenzo[e][1,3]dioxepine-7,8-diol (1u)

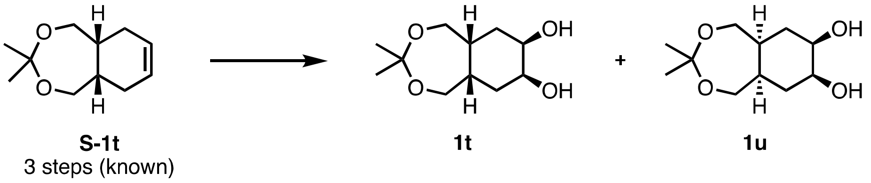

Synthesis of **1t** and **1u** reported in our previous publication.[2] In brief, alkene **S-1t** (1.00 g, 5.49 mmol; prepared in three steps according to literature procedures[3]), was subjected to General Procedure A using $K_2OsO_4 \cdot 2H_2O$ (40.0 mg, 0.11 mmol) and NMO (1.4 mL, 6.48 mmol) to obtain a 2:1 mixture of *syn*-diol diastereomers. Purification by column chromatography (SiO_2, 20–30% acetone/hexane) afforded **1t** (290 mg, 1.34 mmol, 24%) and **1u** (109 mg, 0.50 mmol, 9%) as clear viscous oils that solidifies into an amorphous solid upon standing.

A comprehensive structure and stereochemical assignment of **1t** and **1u** can be found in our previous publication.[2]

Characterization data for **1t**
^{1}**H NMR** (400 MHz, d_3-MeOD) δ 4.58 (br m, 2H), 3.71 (br m, 4H), 1.97 (br m, 2H), 1.76 (br m, 4H), 1.28 (s, 6H).
HRMS (ESI+) $[M+H]^+$ m/z calc'd for $[C_{11}H_{20}O_4H]^+$ expect 217.1440; found 217.1433.

Characterization data for **1u**
^{1}**H NMR** (400 MHz, d_3-MeOD) δ 4.57 (br m, 2H), 3.94–3.48 (br m, 4H), 1.92 (br m, 2H), 1.73 (br m, 2H), 1.55 (br m, 2H), 1.29 (s, 3H), 1.28 (s, 3H).
HRMS (ESI+) $[M+H]^+$ m/z calc'd for $[C_{11}H_{20}O_4H]^+$ expect 217.1440; found 217.1439

Extensive ^{1}H NMR signal broadening observed across a range of NMR solvents for **1t** and **1u** indicates slow conformational interconversion on the NMR time scale, to the extent where it precludes the ability to obtain ^{13}C NMR spectra for both diol diastereomers.

(1*R*,3*R*,4*S*,6*S*)-bicyclo[4.2.0]octane-3,4-diol (1v)

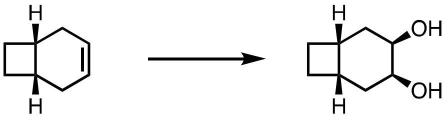

S-1v
4 steps (known)

1v

Synthesis of **1v** reported in our previous publication.[2] In brief, alkene **S-1v** (541 mg, 5.00 mmol; prepared in four steps according to literature procedures[11]) was subjected to General Procedure A using K_2OsO_4·$2H_2O$ (36.8 mg, 0.10 mmol) and NMO (1.2 mL, 6.00 mmol) to afford a 5:1 mixture of *syn*-diol diastereomers. Purification by column chromatography (SiO_2, 20% acetone/hexane) afforded the product **1v** (223 mg, 1.57 mmol, 31%) as a white solid. The minor diastereomer could not be cleanly isolated.

^{1}H NMR (700 MHz, $CDCl_3$) δ 3.97–3.88 (m, 2H), 2.47 (s, 2H), 1.99–1.88 (m, 4H), 1.70 (s, 2H), 1.68–1.58 (m, 4H).
^{13}C NMR (176 MHz, $CDCl_3$) δ 69.5, 31.7, 30.6, 24.7.
HRMS (ESI+) $[M+Na]^+$ m/z calc'd for $[C_8H_{14}O_2Na]^+$ expect 165.0891; found 165.0892.

(1*R*,3*R*,4*S*,6*S*)-7-oxabicyclo[4.1.0]heptane-3,4-diol (1w)

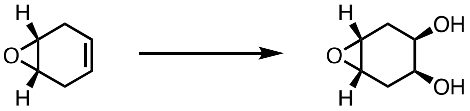

S-1w

1w

Alkene **S-1w** (2.85 g, 25.0 mmol; prepared in one step according to literature procedures[12]) was subjected to General Procedure **A** using K_2OsO_4·$2H_2O$ (184 mg, 0.50 mmol) and NMO (5.2 mL, 25.0 mmol) to afford a 6:1 mixture of *syn*-diol diastereomers. Purification by column chromatography (SiO_2, 20–40% acetone/hexane) afforded product **1w** (204 mg, 1.57 mmol, 6.3%) as a white solid. The minor diastereomer could not be cleanly isolated.

^{1}H NMR (700 MHz, $CDCl_3$) δ 3.71–3.61 (m, 2H), 3.30–3.24 (br s, 2H), 2.49 (dd, *J* = 10.4, 4.0 Hz, 2H), 2.27–2.22 (m, 4H).
^{13}C NMR (176 MHz, $CDCl_3$) δ 68.5, 52.6, 29.7.

NMR in agreement with the previously reported data[13]

(1*R*,3*R*,4*S*,6*S*,7*r*)-7-(((*tert*-butyldimethylsilyl)oxy)methyl)-7-chlorobicyclo[4.1.0]heptane-3,4-diol (1x)

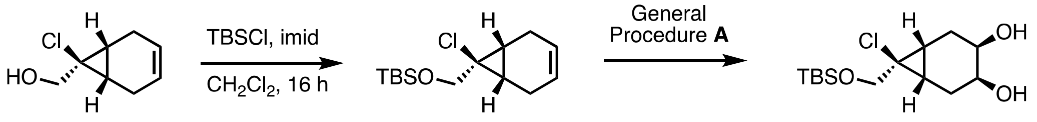

S-1x
(two steps, known)
S-1x'
1x

To a solution of alcohol (440 mg, 2.77 mmol, **S-1x**; prepared in two steps according to literature procedures[14]) in CH_2Cl_2 (20 mL) was added TBSCl (452 mg, 3.00 mmol) and imidazole (245 mg, 3.60 mmol). The reaction was stirred at r.t. for 16 h before quenching with MeOH (1 mL) and the resulting mixture stirred for 1 h at r.t.. Sat. NH_4Cl was next added, the phases separated, and the aqueous phase was extracted with CH_2Cl_2 (2 x 20 mL). The combined organic phases were dried ($MgSO_4$), filtered and the solvent removed under reduced pressure and dried *in vacuo* to afford crude alkene **S-1x'** (*ca.* 750 mg, 2.76 mmol, 99%) as a clear oil, which was carried to the next step without further purification. **S-1x'** (750 mg, 2.76 mmol) was subjected to General Procedure **A** with NMO (0.69 mL, 3.31 mmol) and $K_2OsO_4 \cdot 2H_2O$ (20 mg, 0.055 mmol). Purification by column chromatography (SiO_2, 20% acetone/hexanes) afforded **1x** (500 mg, 1.63 mmol, 59% yield) as a white solid.

^{1}H NMR (700 MHz, $CDCl_3$) δ 3.81–3.78 (m, 2H), 3.78 (s, 2H), 2.17–2.05 (m, 2H), 1.90 (dd, J = 14.2, 4.7 Hz, 2H), 1.83 (d, J = 4.1 Hz, 2H), 1.38–1.32 (m, 2H), 0.91 (s, 9H), 0.08 (s, 6H).
^{13}C NMR (176 MHz, $CDCl_3$) δ 68.8, 68.4, 54.6, 25.9, 25.1, 18.4, 15.7, -5.3.ppm.
HRMS (ESI+) $[M+H]^+$ m/z calc'd for $[C_{14}H_{27}ClO_3SiH]^+$, expect 307.1489; found = 307.1291

(1*R*,4*S*,5*R*,8*S*)-9-oxabicyclo[6.1.0]nonane-4,5-diol (1z)

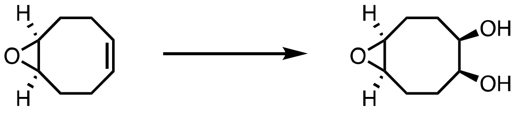

S-1z
1 step (known)
1z

Alkene **S-1z** (1.90 g, 15.0 mmol; prepared in one step according to literature procedures[15]) was subjected to General Procedure **A** using $K_2OsO_4 \cdot 2H_2O$ (110 mg, 0.30 mmol) and NMO (3.1 mL, 15.0 mmol). Purification by column chromatography (SiO_2, 0–5% MeOH/CH_2Cl_2) afforded the product **1z** (581 mg, 3.67 mmol, 24%) as a white solid. Note that the diastereomer arising from convex face dihydroxylation spontaneously cyclizes; observation and stereochemical assignment of **1z** can be found in a previous publication by Hodgson *et al.*[16]

^{1}H NMR (700 MHz, $CDCl_3$) δ 4.05 (dd, *J* = 8.6, 4.2 Hz, 2H), 2.94 (dt, *J* = 7.8, 3.8 Hz, 2H), 2.05 (dq, *J* = 13.5, 4.3 Hz, 2H), 1.92 (ddt, *J* = 13.6, 8.7, 4.3 Hz, 2H), 1.80 (ddd, *J* = 18.1, 9.1, 4.0 Hz, 2H), 1.74–1.62 (m, 4H).
^{13}C NMR (176 MHz, $CDCl_3$) δ 75.0, 55.0, 29.6, 22.5.
HRMS (ESI+) $[M+H]^+$ m/z calc'd for $[C_8H_{14}O_3H]^+$, expect 159.1016; found = 159.1017

(3a*R*,6*R*,7*S*,9a*S*)-2,2-dimethyloctahydrocycloocta[*d*][1,3]dioxole-6,7-diol (1aa)

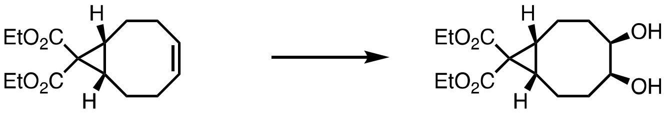

S-1aa
2 steps (known)

1aa
3:1 mixture of
syn diol diastereomers

Synthesis of **1aa** reported in our previous publication.[2] In brief, alkene **S-1aa** (1.28 g, 4.00 mmol; prepared in two steps according to literature procedures[17]) was subjected to General Procedure A using $K_2OsO_4 \cdot 2H_2O$ (29.4 mg, 0.08 mmol) and NMO (1.75 mL, 4.80 mmol). Purification by column chromatography (SiO_2, 20% acetone/hexane) afforded **1m** as a viscous oil (499 mg, 1.67 mmol, 42%) as an inseparable mixture of *syn*-diol diastereomers (3:1 *dr*). Combined signals for both diastereomers are denoted ('). Discernible signals for minor *meso* diastereomer are denoted with (*). Major diastereomer assumed to arise from convex face dihydroxylation.

^{1}**H NMR** (700 MHz, $CDCl_3$) δ 4.18–4.14' (m, 3H), 4.14–4.09' (m, 3H), 4.08* (dd, *J* = 8.1, 4.0 Hz, 0.6H), 3.87 (dd, *J* = 6.6, 4.3 Hz, 2H), 2.09–1.99 (m, 2H), 1.93–1.87' (m, 3H), 1.85 (dq, *J* = 14.4, 4.2 Hz, 2H), 1.80 (qd, *J* = 9.0, 3.1 Hz, 2H), 1.72–1.67* (m, 0.6H), 1.64–1.60* (m, 0.6H), 1.43–1.37* (m, 0.6H), 1.27' (t, *J* = 7.1 Hz, 4H), 1.23–1.19' (m, 4H), 1.18–1.07 (m, 2H).
^{13}**C NMR** (176 MHz, $CDCl_3$) δ 170.9*, 170.8, 167.1, 166.8*, 76.0, 75.4, 61.6*, 61.6, 61.0*, 61.0, 38.0, 37.2*, 33.1*, 32.5, 31.0, 30.5*, 21.0, 19.3*, 14.1, 14.1*, 14.1'.
HRMS (ESI+) $[M+H]^+$ m/z calc'd for $[C_{15}H_{24}O_6H]^+$ expect 301.1651; found 301.1653

(3a*R*,6*S*,7*R*,9a*S*)-2,2-dimethyloctahydrocycloocta[*d*][1,3]dioxole-6,7-diol (1ab) and
(3a*R*,6*R*,7*S*,9a*S*)-2,2-dimethyloctahydrocycloocta[*d*][1,3]dioxole-6,7-diol (1ac)

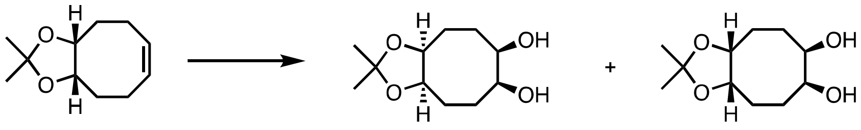

S-1ab
3 steps (known)

1ab

1ac

Synthesis of **1ab** and **1ac** was reported in our previous publication.[2] In brief, alkene **S-1ab** (1.28 g, 7.00 mmol; prepared in three steps according to literature procedures[18]) was subjected to General Procedure **A** using $K_2OsO_4 \cdot 2H_2O$ (51.5 mg, 0.14 mmol) and NMO (1.75 mL, 8.40 mmol) to obtain a 1:1 mixture of *syn*-diol diastereomers. Purification by column chromatography (SiO_2, 10–20% acetone/hexane) afforded **1ab** (130 mg, 0.60 mmol, 8.6%) and **1ac** (106 mg, 0.49 mmol, 7.0%) as white solids. A comprehensive structure and stereochemical assignment of **1ab** and **1ac** can be found in our previous publication.[2]

Characterization data for **1ab**
^{1}**H NMR** (700 MHz, $CDCl_3$) δ 4.28 (d, *J* = 8.4 Hz, 2H), 3.95 (d, *J* = 6.5 Hz, 2H), 1.86 (d, *J* = 11.3 Hz, 4H), 1.72 – 1.62 (m, 4H), 1.40 (s, 3H), 1.32 (s, 3H).
^{13}**C NMR** (176 MHz, $CDCl_3$) δ 106.7, 78.3, 73.9, 28.9, 28.1, 25.5, 25.2.

HRMS (ESI+) [M+Na]$^+$ m/z calc'd for $[C_{11}H_{20}O_4Na]^+$ expect 239.1259; found 239.1258

Characterization data for **1ac**

^{1}**H NMR** (700 MHz, $CDCl_3$) δ 4.11 (dd, *J* = 8.3, 6.3 Hz, 2H), 3.84 (dd, *J* = 8.5, 4.1 Hz, 2H), 2.19 – 2.06 (m, 2H), 2.01 – 1.91 (m, 2H), 1.70 (tt, *J* = 13.1, 4.4 Hz, 5H), 1.41 (s, 3H), 1.32 (s, 3H).

^{13}**C NMR** (176 MHz, $CDCl_3$) δ 106.3, 78.2, 74.3, 30.4, 28.0, 25.2, 24.9.ppm

HRMS (ESI+) [M+H]$^+$ m/z calc'd for $[C_{11}H_{20}O_4H]^+$ expect 217.1440; found 217.1439.

(4*R*,5*S*)-1,8-diphenyloctane-4,5-diol (3e)

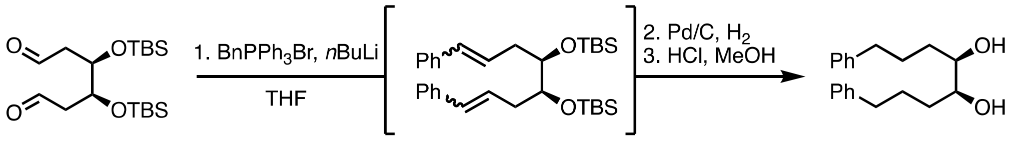

Synthesis of **3e** is reported in our previous publication.[2] In brief, **S-3e** (750 mg, 2.0 mmol, prepared in four steps according to literature procedures[19]) was subjected to Wittig olefination conditions to obtain crude alkene **S-3e'** (*ca.* 658 mg, 1.26 mmol, 63%), which was carried forward to the next step without further purification.

Crude alkene **S-3e'** (*ca.* 658 mg, 1.26 mmol) dissolved in EtOAc (10 mL) was next hydrogenated with Pd/C (67 mg, 0.06 mmol) under an atmosphere of H_2 to obtain the crude product (*ca.* 600 mg, 1.13 mmol, 90%). The crude product was dissolved in MeOH (10 mL), desilylated using HCl (5 drops, 3 M aq.) and worked up to obtain diol **3e** (321 mg, 1.08 mmol, 54% over three steps) as a white solid.

^{1}**H NMR** (700 MHz, $CDCl_3$) δ 7.28 (t, *J* = 7.4 Hz, 4H), 7.20–7.16 (m, 6H), 3.63–3.59 (m, 2H), 2.64 (hept, *J* = 6.5 Hz, 4H), 1.86 (dp, *J* = 15.4, 7.9 Hz, 2H), 1.76 (d, *J* = 3.9 Hz, 2H), 1.64 (dp, *J* = 15.4, 7.9 Hz, 2H), 1.46 (q, *J* = 7.9, 6.5 Hz, 4H).

^{13}**C NMR** (176 MHz, $CDCl_3$) δ 142.2, 128.4, 128.4, 125.8, 74.5, 35.8, 30.8, 27.8.

HRMS (ESI+) [M+Na]$^+$ m/z calc'd for $[C_{11}H_{20}O_4Na]^+$ expect 239.1259; found 239.1258

(7*R*,8*S*)-2,2,3,3,12,12,13,13-octamethyl-4,11-dioxa-3,12-disilatetradecane-7,8-diol (3h)

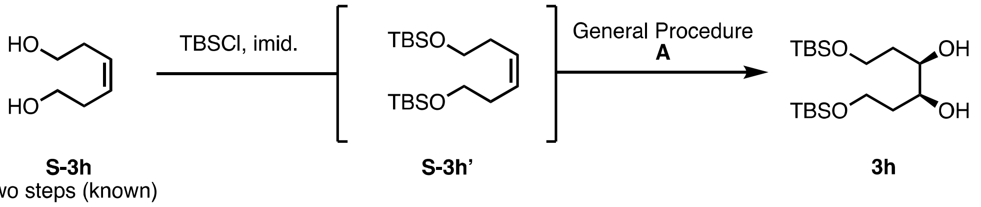

To a solution of diol (406 mg, 3.50 mmol, **S-3h**; prepared in two steps according to literature procedures[20]) in CH_2Cl_2 (20 mL) was added TBSCl (1.16 g, 7.69 mmol) and imidazole (600 mg, 8.73 mmol). The reaction was stirred at r.t. for 2 h before quenching with MeOH (1 mL) and the resulting mixture stirred at r.t. for 1 h. Sat. NH_4Cl was next added, the phases separated, and the aqueous phase was extracted with CH_2Cl_2 (2 x 20 mL). The combined organic phases were dried ($MgSO_4$), filtered and the solvent removed under reduced pressure and dried *in vacuo* to afford crude alkene **S-3h'** (*ca.* 1.15 g, 3.33 mmol, 95%) as a clear oil, which was carried to the next step without further purification. **S-3h'** (1.15 g, 3.33 mmol) was subjected to General Procedure A with NMO (0.83 mL, 3.99 mmol) and $K_2OsO_4{\cdot}2H_2O$ (24 mg, 0.067 mmol). Purification by column chromatography (SiO_2, 5% acetone/hexanes) afforded **3h** (608 mg, 1.61 mmol, 48% yield) as a white solid.

^{1}H NMR (700 MHz, $CDCl_3$) δ 3.93 (ddd, *J* = 9.9, 5.5, 4.3 Hz, 1H), 3.85 (ddd, *J* = 10.2, 8.8, 3.7 Hz, 2H), 3.72 (m, 2H), 3.68 (d, *J* = 2.0 Hz, 2H), 1.83 (m, 2H), 1.73 (m, 2H), 0.90 (s, 18H), 0.08 (d, *J* = 1.5 Hz, 12H).
^{13}C NMR (176 MHz, $CDCl_3$) δ 74.6, 62.6, 33.8, 25.9, 18.1, -5.5, -5.6.
HRMS (ESI+) $[M+H]^+$ m/z calc'd for $[C_{18}H_{42}O_4Si_2H]^+$ expect 376.2694; found 379.2686

(8*R*,9*S*)-2,2,15,15-tetramethyl-3,3,14,14-tetraphenyl-4,13-dioxa-3,14-disilahexadecane-8,9-diol (3g)

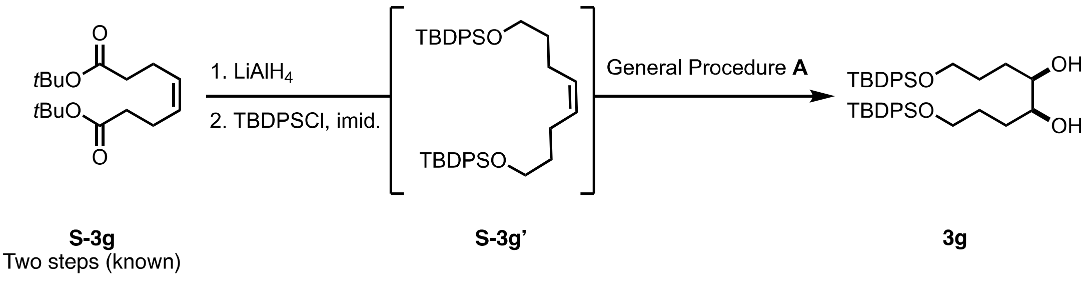

$LiAlH_4$ (4.4 mmol, 3.0 eq, 2.4 M solution in THF) was added dropwise to a stirred solution of **S-3g** (1.00 g, 3.52 mmol, prepared in two steps according to literature procedures[21]) in THF (5 mL) at 0 °C. The reaction mixture was stirred at 0 °C for 2 h, allowed to warm to r.t. over 2 h before cooling to 0 °C before carefully quenching with sat. NH_4Cl solution. The mixture was diluted with H_2O (30 mL) and extracted with EtOAc. The combined organic extracts were dried over $MgSO_4$, filtered and the solvent evaporated under reduced pressure to afford the yield crude diol. The crude diol (533 mg, 3.70 mmol) was next dissolved in CH_2Cl_2 (40 mL). TBDPSCl (2.4 mL, 9.24 mmol, 2.5 eq) and imidazole (754.8 mg, 11.09 mL, 3.0 eq) were successively added and the reaction was stirred at r.t. for 15 h. The reaction was quenched with sat. NH_4Cl solution, extracted with CH_2Cl_2 (2 x 20 mL).

The combined organic extracts were dried over $MgSO_4$, filtered, and the solvent was removed under reduced pressure to provide crude *bis*-TBDPS ether (**S-3g'**), which was carried forward without further purification.

Crude **S-3g'** (*ca.* 1.5 g, 2.42 mmol) was subjected to modified General Procedure **A** with NMO (0.60 mL, 2.90 mmol) and $K_2OsO_4{\cdot}2H_2O$ (89 mg, 0.241 mmol). After reaction completion, the reaction was quenched with sat. Na_2SO_3 (100 µL), and the reaction mixture concentrated to dryness over SiO_2 under reduced pressure. Purification of the crude product (adsorbed onto SiO_2) by column chromatography (SiO_2, 10% EtOAc/hexanes then 40% acetone/hexanes) gave **3g** as a white solid (791 mg, 1.20 mmol, 50%).

^{1}H NMR (700 MHz, $CDCl_3$) δ 7.70 – 7.66 (m, 8H), 7.43 (t, *J* = 7.3 Hz, 4H), 7.39 (t, *J* = 7.2 Hz, 8H), 3.72 (t, J = 5.8 Hz, 4H), 3.63 (d, J = 7.7 Hz, 2H), 2.80 (br s, 2H), 1.80 – 1.72 (m, 2H), 1.72 – 1.63 (m, 4H), 1.56 – 1.48 (m, 2H), 1.06 (s, 18H).
^{13}C NMR (176 MHz, $CDCl_3$) δ 135.7, 133.7, 129.8, 127.8, 74.4, 64.5, 29.2, 28.6, 27.0, 19.3.
HRMS (ESI$^+$) $[M+H]^+$m/z calc'd for $[C_{40}H_{54}O_4Si_2H]^+$ expect 655.3633, found = 655.3630

(7*R*,8*S*)-2,2,4,4,11,11,13,13-octamethyl-3,12-dioxa-2,13-disilatetradecane-7,8-diol (3i)

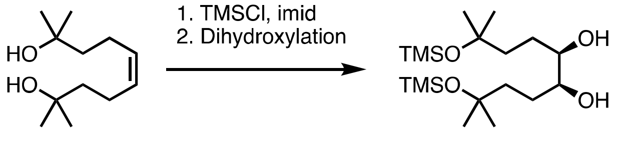

S-3i
6 steps (known)

3i

Synthesis of **3i** is reported in our previous publication.[2] In brief, **S-3i** (1.87 g, 9.35 mmol, prepared in six steps according to literature procedures)[22] was silylated with TMSCl (6.0 mL, 47.5 mmol), imidazole (3.90 g, 57 mmol) in dichloromethane (30 mL) to afford the crude silyl ether (*ca.* 2.76 g, 8.0 mmol), which was carried forward without further purification.

Crude silyl ether (*ca.* 2.76 g, 8.0 mmol) was subjected to General Procedure **A** with $K_2OsO_4{\cdot}2H_2O$ (59.0 mg, 0.16 mmol) and NMO (1.70 mL, 8.00 mmol). Purification by column chromatography (SiO_2, 5–20% EtOAc/hexane) afforded **3i** (2.28 g, 6.02 mmol, 64% over 2 steps from **S-3i**) as a viscous clear oil.

^{1}H NMR (700 MHz, $CDCl_3$) δ 3.56 (d, *J* = 5.8 Hz, 2H), 2.97 (s, 2H), 1.70–1.60 (m, 4H), 1.58–1.51 (m, 4H), 1.26 (s, 3H), 1.25 (s, 3H), 0.13 (s, 18H).
^{13}C NMR (176 MHz, $CDCl_3$) δ 74.8, 74.4, 41.3, 30.0, 29.8, 26.1, 2.5.
HRMS (ESI+) $[M+NH_4]^+$ m/z calc'd for $[C_{18}H_{42}O_4Si_2NH_4]^+$ expect 396.2965; found 396.2931

(2*E*,6*R*,7*S*,10*E*)-6,7-dihydroxydodeca-2,10-diene-1,12-diyl bis(2,2-dimethylpropanoate) (3j)

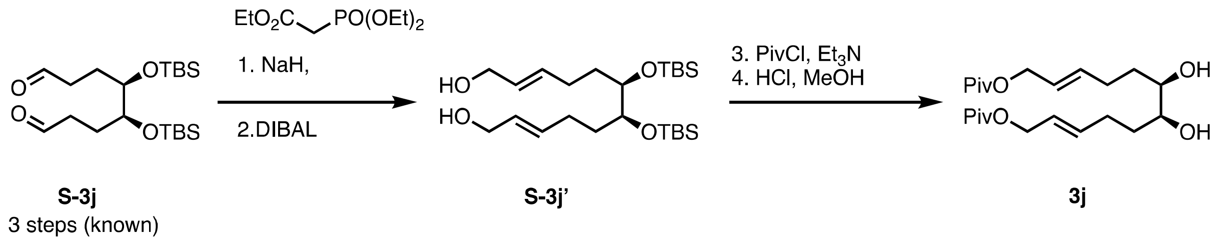

NaH (368 mg, 9.2 mmol, 60% w/w) was added to a stirred solution of triethyl phosphonoacetate (2.0 g, 9.2 mmol) in THF at 0 °C. A solution of **S-3j** (930 mg, 2.3 mmol, prepared in three steps according to literature procedures[23]) in THF (10 mL) was next added dropwise *via* cannula into the reaction mixture, and the reaction was allowed to warm to r.t. over 1 h. The reaction mixture was diluted with Et_2O/hexanes (1:1 40 mL), filtered over a pad of SiO_2 (eluting with Et_2O) to afford the crude dienoate (*ca.* 899 mg, 1.66 mmol, 72%). This was next dissolved in CH_2Cl_2 (10 mL) and cooled to –78 °C. DIBAL (3.65 mL, 3.65 mmol, 1.0 M in PhMe) was next added dropwise to the reaction mixture and the reaction was allowed to warm to –40 °C over 2 h. The reaction was quenched with Na/K tartrate (10 mL), allowed to warm to r.t. and vigorously stirred for 1.5 h. The layers were separated and the aqueous phase extracted with EtOAc (2 x 10 mL). The combined organic phase was dried ($MgSO_4$) and concentrated *in vacuo* to afford the crude diol **S-3j'** (*ca.* 716 mg, 1.56 mmol, 68% from **S-3j**), which was carried forward without further purification.

PivCl (480 µL, 3.92 mmol) was added dropwise to a stirred solution of crude diol **S-3j'** (716 mg, 1.56 mmol) and Et_3N (660 µL, 4.74 mmol) in CH_2Cl_2 (15 mL). The reaction was stirred for 16 h at r.t. before quenching with NH_4Cl (15 mL) and the layers were separated. The aqueous phase was extracted with CH_2Cl_2 (2 x 10 mL); the combined organic phase was dried ($MgSO_4$) and concentrated *in vacuo*. Purification by column chromatography (SiO_2, 5% EtOAc/hexane) afforded the intermediary pivaloyl ester. This was dissolved in MeOH (10 mL) and HCl (70 µL, 0.21 mmol, 3.0 M in water) was added. The reaction mixture was evaporated to dryness *in vacuo* at 50 °C for 16 h, before redissolving in CH_2Cl_2. Solid $NaHCO_3$ was next added, and the mixture was filtered through a pad of $MgSO_4$ and evaporated to afford **3j** as a viscous oil (276 mg, 0.69 mmol, 44% over two steps from **S-3j'**)

^{1}H NMR (700 MHz, $CDCl_3$) δ 5.77 (dt, *J* = 13.7, 6.7 Hz, 2H), 5.66 – 5.53 (m, 2H), 4.50 (d, *J* = 6.0 Hz, 4H), 3.60 (dq, *J* = 7.7, 3.4 Hz, 2H), 2.29 (dq, *J* = 13.8, 6.8 Hz, 2H), 2.14 (dq, *J* = 15.0, 7.6 Hz, 2H), 1.20 (s, 18H).
^{13}C NMR (176 MHz, $CDCl_3$) δ 178.4, 134.7, 125.0, 74.1, 64.8, 38.8, 30.5, 28.8, 27.2.ppm
HRMS (ESI+) $[M+Na]^+$ m/z calc'd for $[C_{22}H_{38}O_6Na]^+$ expect 421.2566; found 421.2558.

Product characterization

4.1. Enantioenriched hydroxyketones

(*S*)-2-hydroxycyclohexan-1-one (2a)

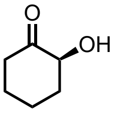

Cyclohexane-1,2-diol **1a** (11.6 mg, 0.10 mmol) was subjected to General Procedure **B**, with 4CzIPN (3.9 mg, 0.005 mmol), *epi*-NHBoc-DHCN (4.0 mg, 0.01 mmol), TBA·H_2PO_4 (8.5 mg, 0.025 mmol), DIAD (20 mg, 0.1 mmol) in MeCN (4 mL). Purification by column chromatography (SiO_2, 50–100% CH_2Cl_2/hexane) afforded **2a** (10.5 mg, 0.092 mmol, 92%) as a clear oil. Note: product **2a** is volatile and care must be taken on solvent evaporation to avoid product loss.

^{1}H NMR (500 MHz, $CDCl_3$) δ 4.22–4.05 (m, 1H), 3.66 (d, *J* = 3.2 Hz, 1H), 2.59 (ddt, *J* = 13.9, 4.3, 2.2 Hz, 1H), 2.49 (ddq, *J* = 12.5, 6.2, 3.1 Hz, 1H), 2.38 (tdd, *J* = 13.9, 6.4, 1.4 Hz, 1H), 2.14 (ddt, *J* = 12.5, 6.1, 3.0 Hz, 1H), 1.92 (dq, *J* = 13.4, 3.0 Hz, 1H), 1.74 (qt, *J* = 13.0, 3.2 Hz, 1H), 1.70–1.57 (m, 1H; overlaps with H_2O), 1.51 (qd, *J* = 12.8, 3.7 Hz, 1H).
^{13}C NMR (126 MHz, $CDCl_3$) δ 211.4, 75.4, 39.5, 36.7, 27.6, 23.4.
$[\alpha]_D$: –13.2 (c = 0.9, T = 25 °C, $CHCl_3$).
*Literature $[\alpha]_D$ values for **S**-2a:* –18.8 (c = 0.65; T = 22 °C $CHCl_3$)[24]
*Absolute configuration of **2a** therefore assigned as **S***

Data in agreement with literature reported for ***S*-2a**.[24]

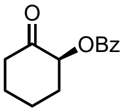

Derivatization for enantiomeric excess (ee) determination (**Bz-2a**)
Bz-2a was prepared via General Procedure **C2**. Purified by preparative thin layer chromatography (SiO_2, 20% EtOAc/hexanes) afforded an analytical sample of **Bz-2a** for ee determination. Chiral HPLC analysis indicated that product **2a** was formed in 91% ee. A sample of racemic **2a** was synthesized *via* General Procedure **D1**

^{1}H NMR (700 MHz, $CDCl_3$) δ 8.17 – 8.07 (m, 2H), 7.64 – 7.54 (m, 1H), 7.51 – 7.43 (m, 2H), 5.44 (ddd, *J* = 12.3, 6.3, 0.9 Hz, 1H), 2.60 (ddt, *J* = 13.7, 4.6, 2.7 Hz, 1H), 2.50 (ddd, *J* = 13.6, 6.1, 1.0 Hz, 1H), 2.49 – 2.43 (m, 1H), 2.15 (ddq, *J* = 12.3, 6.1, 3.0 Hz, 1H), 2.07 (ddq, *J* = 12.6, 6.9, 3.5 Hz, 1H), 1.96 (qd, *J* = 12.5, 3.6 Hz, 1H), 1.87 (qt, *J* = 13.0, 3.5 Hz, 1H), 1.72 (qt, *J* = 13.5, 4.1 Hz, 1H).
^{13}C NMR (176 MHz, $CDCl_3$) δ 204.4, 165.6, 133.2, 129.9, 129.7, 128.4, 77.0, 40.8, 33.2, 27.2, 23.8.

Data in agreement with literature reported for **Bz-2a**[24]

Chiral HPLC Analysis: CHIRALPAK IG (*n*-hexane: *i*PrOH, 95:05, 1.25 mL min^{-1}, 40 °C, 226 nm) t_R = 13.05 (minor), 16.10 (major) minutes. 91% ee

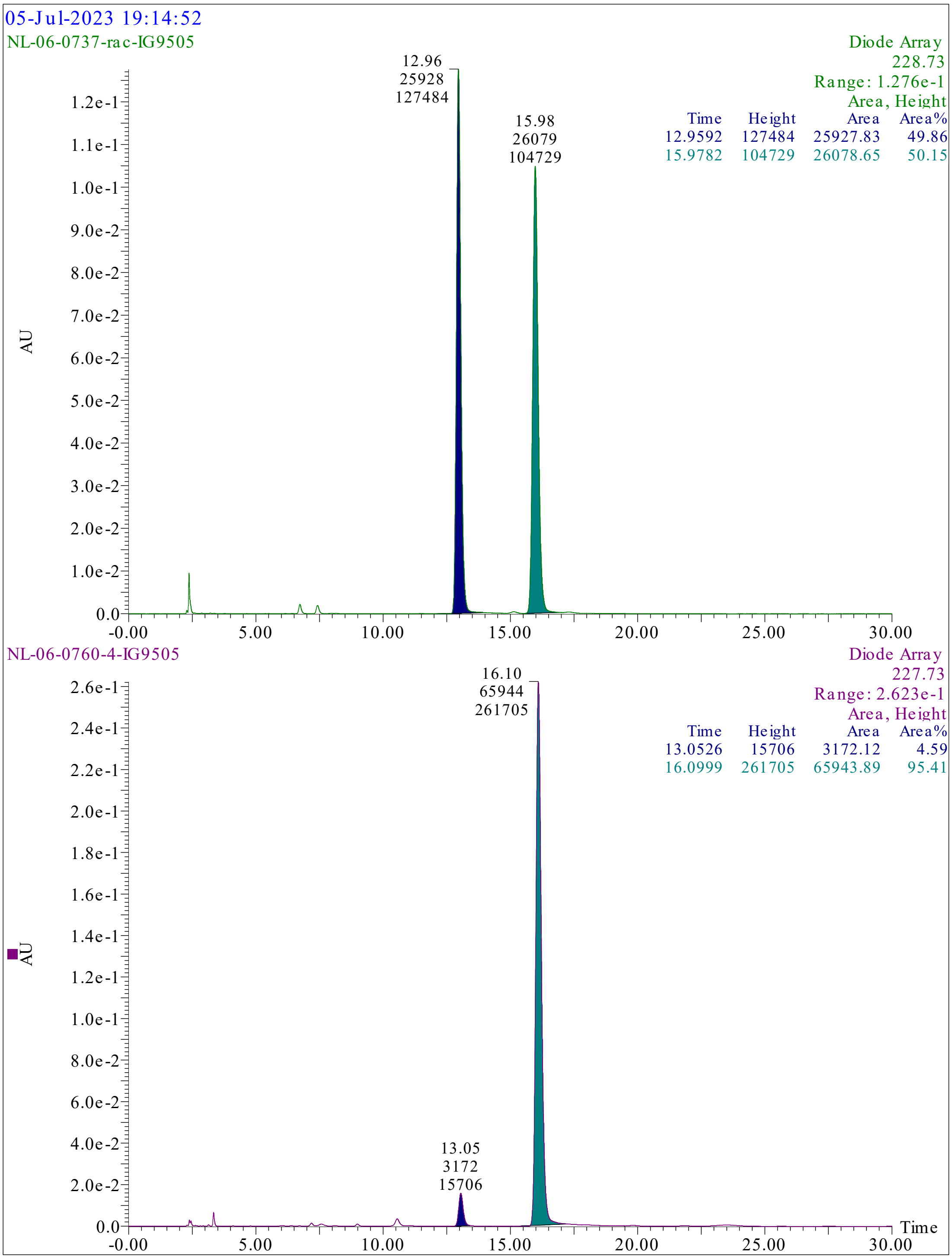

(*S*)-2-hydroxycycloheptan-1-one (2b)

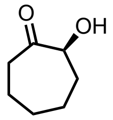

Cycloheptane-1,2-diol **1b** (13.0 mg, 0.10 mmol) was subjected to General Procedure **B**, with 4CzIPN (3.9 mg, 0.005 mmol), *epi*-NHBoc-DHCN (4.0 mg, 0.01 mmol), TBA·H_2PO_4 (8.5 mg, 0.025 mmol), DIAD (20 mg, 0.1 mmol) in MeCN (4 mL) and reacted for 24 h. Purification by column chromatography (SiO_2, 50–100% CH_2Cl_2/hexane) afforded **2b** (10.5 mg, 0.092 mmol, 92%) as a clear oil.

Accessing product enantiomer (*ent*-**2b**)
1b (11.6 mg, 0.10 mmol) was subjected to General Procedure **B**, with 4CzIPN (3.9 mg, 0.005 mmol), *epi*-NHBoc-DHCD (4.0 mg, 0.01 mmol), TBA·H_2PO_4 (8.5 mg, 0.025 mmol) in MeCN (4 mL) and reacted for 24 h. Purification by column chromatography (SiO_2, 50–100% CH_2Cl_2/hexane) afforded *ent*-**2b** (11.7 mg, 0.091 mmol, 91%) as a clear oil.

^{1}H NMR (700 MHz, $CDCl_3$) δ 4.29 (d, *J* = 9.3 Hz, 1H), 3.96–3.74 (m, 1H), 2.69 (dddd, *J* = 17.4, 6.9, 2.9, 1.0 Hz, 1H), 2.46 (ddd, *J* = 17.4, 11.3, 3.4 Hz, 1H), 2.10–1.96 (m, 1H), 1.93 – 1.84 (m, 1H), 1.85–1.77 (m, 2H), 1.77–1.49 (m, 4H), 1.41 – 1.29 (m, 1H).
^{13}C NMR (176 MHz, $CDCl_3$) δ 213.9, 77.1, 40.1, 33.8, 29.6, 26.7, 23.5.
$[\alpha]_D$: +93.9 (**2b**, c = 0.54, T = 25 °C, $CHCl_3$); **$[\alpha]_D$**: –88.5 (*ent*-**2b**, c = 0.33, T = 25 °C, $CHCl_3$). *Literature $[\alpha]_D$ values for **R**-***2b***:* –86.4 (c = 0.65; T = 28 °C $CHCl_3$).[25]
*Absolute configuration of **2b** therefore assigned as **S**.*
*Absolute configuration of ent-**2b** therefore assigned as **R**.*

Data in agreement with literature reported for ***S*-2b**.[25]

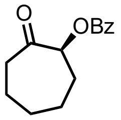

Derivatization for enantiomeric excess (ee) determination (**Bz-2b**)
Bz-2b and **Bz-*ent*-2b** was prepared via General Procedure **C2**. Purified by preparative thin layer chromatography (SiO_2, 20% EtOAc/hexanes) afforded an analytical sample of **Bz-2b** and **Bz-*ent*-2b** for ee determination. A sample of racemic **Bz-2b** was synthesized *via* General Procedure **D1**

^{1}H NMR (700 MHz, $CDCl_3$) δ 8.21 – 8.04 (m, 2H), 7.66 – 7.55 (m, 1H), 7.51 – 7.42 (m, 2H), 5.49 (dd, *J* = 9.6, 3.4 Hz, 1H), 2.73 (dddd, *J* = 16.5, 6.5, 4.5, 1.0 Hz, 1H), 2.54 (ddd, *J* = 16.5, 10.0, 4.9 Hz, 1H), 2.22 – 2.11 (m, 1H), 1.99–1.90 (m, 3H), 1.91–1.79 (m, 2H), 1.81 – 1.71 (m, 1H), 1.52 – 1.44 (m, 1H).
^{13}C NMR (176 MHz, $CDCl_3$) δ 207.4, 133.2, 129.9, 129.7, 128.4, 79.0, 40.7, 30.4, 28.4, 26.5, 23.0.

Data in agreement with literature reported for **Bz-2b**[24]

Chiral SFC Analysis: CHIRALPAK IC (CO_2: MeOH, 91:09, 2.5 mL min^{-1}, 40 °C, 226 nm)
Bz-2b (middle): t_R = 4.76 (minor), 5.33 (major) minutes, 92% ee
Bz-*ent*-2b *via epi-NHBoc-DHCD* (bottom): t_R = 4.74 (major), 5.57 (minor) minutes, –91% ee

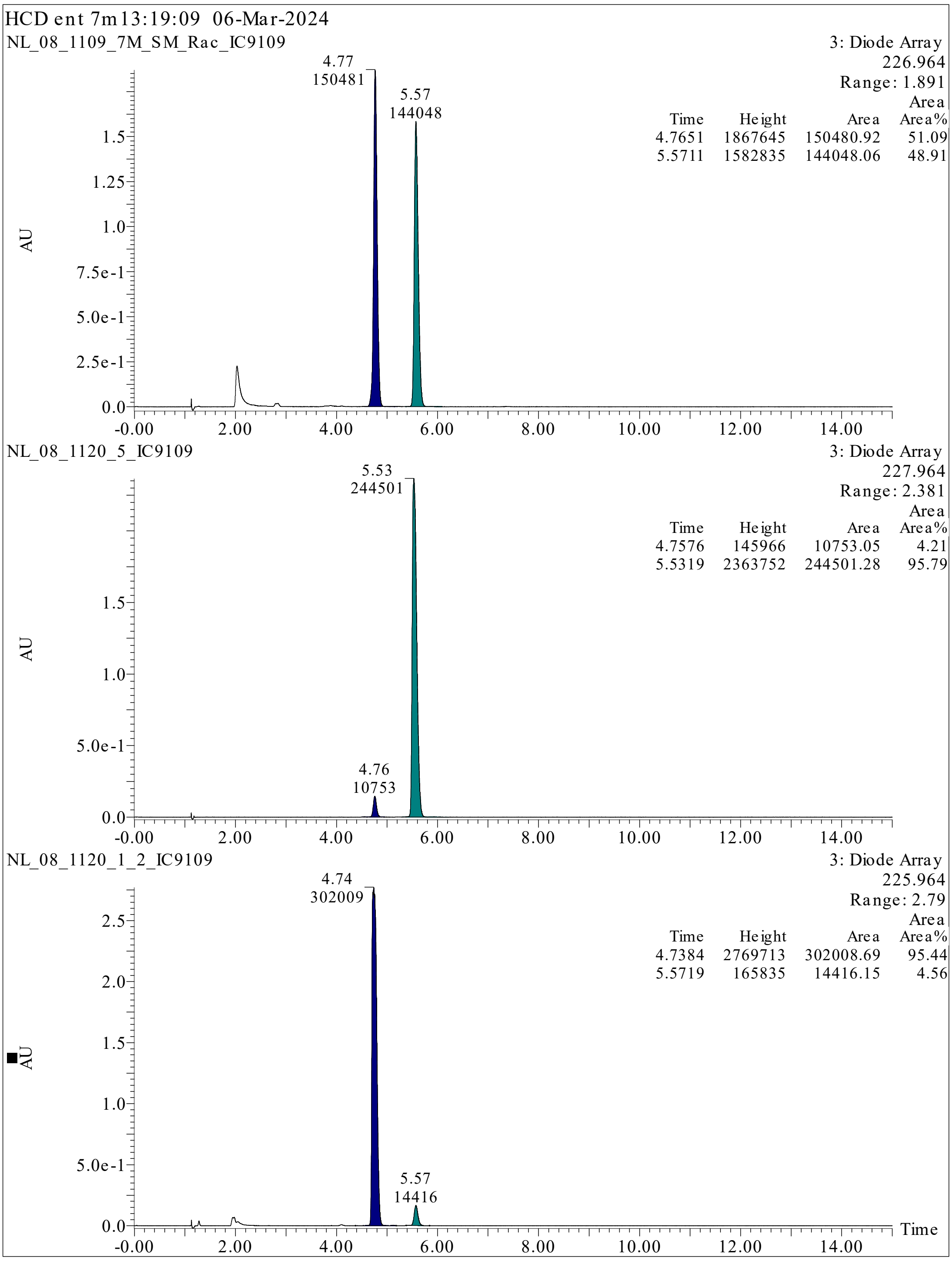

(*S*)-2-hydroxycyclooctan-1-one (2c)

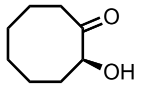

Cyclooctane-1,2-diol **1c** (14.4 mg, 0.10 mmol) was subjected to General Procedure **B**, with 4CzIPN (3.9 mg, 0.005 mmol), *epi*-NHBoc-DHCN (4.0 mg, 0.01 mmol), TBA·H_2PO_4 (8.5 mg, 0.025 mmol), DIAD (20 mg, 0.1 mmol) in MeCN (4 mL) and reacted for 36 h. Purification by column chromatography (SiO_2, 50–100% CH_2Cl_2/hexane) afforded **2b** (11.6 mg, 0.082 mmol, 82%) as a clear oil.

^{1}H NMR (400 MHz, $CDCl_3$) δ 4.18 (dt, *J* = 6.0, 2.8 Hz, 1H), 3.79 – 3.49 (m, 1H), 2.71 (td, *J* = 12.2, 3.9 Hz, 1H), 2.36 (tdd, *J* = 16.2, 8.3, 3.6 Hz, 2H), 2.15 – 1.89 (m, 2H), 1.89 – 1.57 (m, 4H), 1.38 (dtt, *J* = 16.0, 6.9, 3.6 Hz, 2H), 1.01–0.72 (m, 1H).
^{13}C NMR (101 MHz, $CDCl_3$) δ 217.5, 76.2, 37.2, 29.3, 28.6, 25.5, 24.5, 22.1.
$[\alpha]_D$: +50.4 (c = 0.59, T = 25 °C, $CHCl_3$).
*Literature $[\alpha]_D$ values for **R**-2c:* –36.5 (c = 1.0; T = 28 °C $CHCl_3$).[25]
*Absolute configuration of **2c** therefore assigned as **S***

Data in agreement with literature reported for ***S*-2c**.[25]

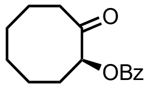

Derivatization for enantiomeric excess (ee) determination (**Bz-2c**)
Bz-2c was prepared via General Procedure **C1**. Purified by preparative thin layer chromatography (SiO_2, 20% CH_2Cl_2/hexanes) afforded an analytical sample of **Bz-2c** for ee determination. Chiral SFC analysis indicated that **2c** was formed in 82% ee. A sample of racemic **Bz-2c** was synthesized *via* General Procedure **D1**.

^{1}H NMR (700 MHz, $CDCl_3$) δ 8.10 (dd, *J* = 8.3, 1.2 Hz, 2H), 7.69 – 7.54 (m, 1H), 7.47 (t, *J* = 7.8 Hz, 2H), 5.45 (dd, *J* = 8.7, 3.7 Hz, 1H), 2.77 (ddd, *J* = 13.3, 9.7, 3.3 Hz, 1H), 2.46 (ddd, *J* = 14.1, 9.2, 3.4 Hz, 1H), 2.36 (ddt, *J* = 13.3, 9.6, 3.5 Hz, 1H), 2.16 – 2.03 (m, 2H), 2.03 – 1.87 (m, 2H), 1.74 – 1.66 (m, 2H), 1.63 – 1.55 (m, 2H), 1.39 – 1.25 (m, 2H).
^{13}C NMR (176 MHz, $CDCl_3$) δ 211.7, 166.0, 133.2, 129.8, 129.6, 128.4, 77.4, 40.5, 31.3, 27.5, 24.6, 24.5, 21.9.

Data in agreement with literature reported for **Bz-2c**[26]

Chiral SFC Analysis: CHIRALPAK IG (CO_2: MeOH, 90:10, 2.5 mL min^{-1}, 40 °C, 226 nm) t_R = 10.16 (minor), 10.81 (major) minutes. 82% ee

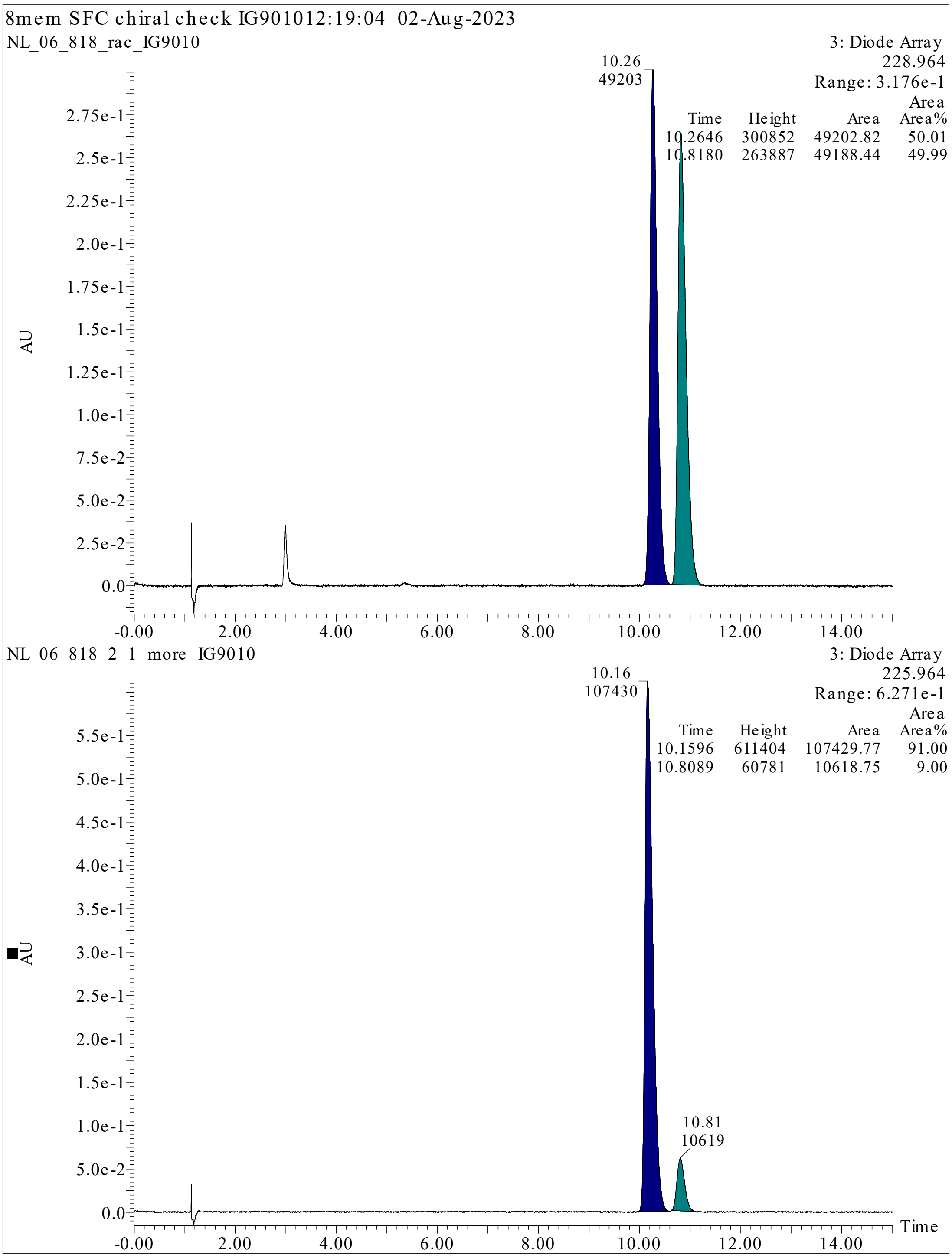

(S)-2-hydroxycyclopentan-1-one (2d; isolated as **Bz-2d**)

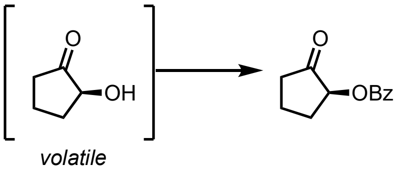

Cyclopentane-1,2-diol **1d** (10.2 mg, 0.10 mmol) was subjected to General Procedure **B**, with 4CzIPN (3.9 mg, 0.005 mmol), *epi*-NHBoc-DHCN (4.0 mg, 0.01 mmol), TBA·H_2PO_4 (8.5 mg, 0.025 mmol), DIAD (20 mg, 0.1 mmol) in MeCN (4 mL) and reacted for 24 h. Purification by column chromatography (SiO_2, 50–100% CH_2Cl_2/hexane then 10% EtOAc/hexane) afforded crude **2d** (mixed with H_2-DIAD). **2d** is very volatile (at no point should solvent be allowed to fully evaporate to dryness to minimize product loss), and was telescoped to the corresponding benzoate ester for preparative isolation.

The crude mixture was then dissolved in CH_2Cl_2 (1 mL), and BzOH (11.2 mg, 0.1 mmol), EDC·HCl (19.2 mg, 0.1 mmol) and DMAP (12.2 mg, 0.1 mmol) were added successively. The reaction was stirred for 2 h at r.t. and monitored by TLC analysis. Upon completion, the sample was concentrated under a stream of air and then adsorbed onto SiO_2. Purification of the adsorbed crude mixture by column chromatography (SiO_2, 10–100% CH_2Cl_2/hexane) afforded **Bz-2d** (9.6 mg, 0.082 mmol, 47% over two steps) as a white solid. Chiral HPLC analysis indicated that **Bz-2d** was formed in 68% ee. A sample of racemic **Bz-2d** was synthesized *via* General Procedure **D1.**

Characterization data for **Bz-2d**
^{1}**H NMR** (700 MHz, $CDCl_3$) δ .12 – 8.01 (m, 2H), 7.62 – 7.57 (m, 1H), 7.50 – 7.45 (m, 2H), 5.33 (dd, J = 10.7, 8.6 Hz, 1H), 2.65 – 2.52 (m, 1H), 2.53 – 2.42 (m, 1H), 2.37 (dt, J = 19.8, 9.9 Hz, 1H), 2.26 – 2.15 (m, 1H), 2.04 (qd, J = 11.8, 6.8 Hz, 1H), 2.02 – 1.93 (m, 1H).
^{13}**C NMR** (176 MHz, $CDCl_3$) δ 212.3, 165.8, 133.3, 129.9, 129.4, 128.4, 76.1, 35.0, 28.6, 17.3.
[α]$_D$: +50.2 (c = 0.9, T = 25 °C, $CHCl_3$).
Literature [α]$_D$ values for ***S*-Bz-2d***:* +38.4 (c = 1.0; T = 22 °C, $CHCl_3$).[24]
Absolute configuration of ***Bz-2d*** *(and therefore* ***2d****) therefore assigned as* ***S***

Data in agreement with literature reported for **Bz-2d**[24]

Chiral SFC Analysis: CHIRALPAK IC (CO_2: MeOH, 93:7, 2.5 mL min^{-1}, 40 °C, 227 nm). t_R = 4.39 (major), 5.22 (minor) minutes, 75% ee

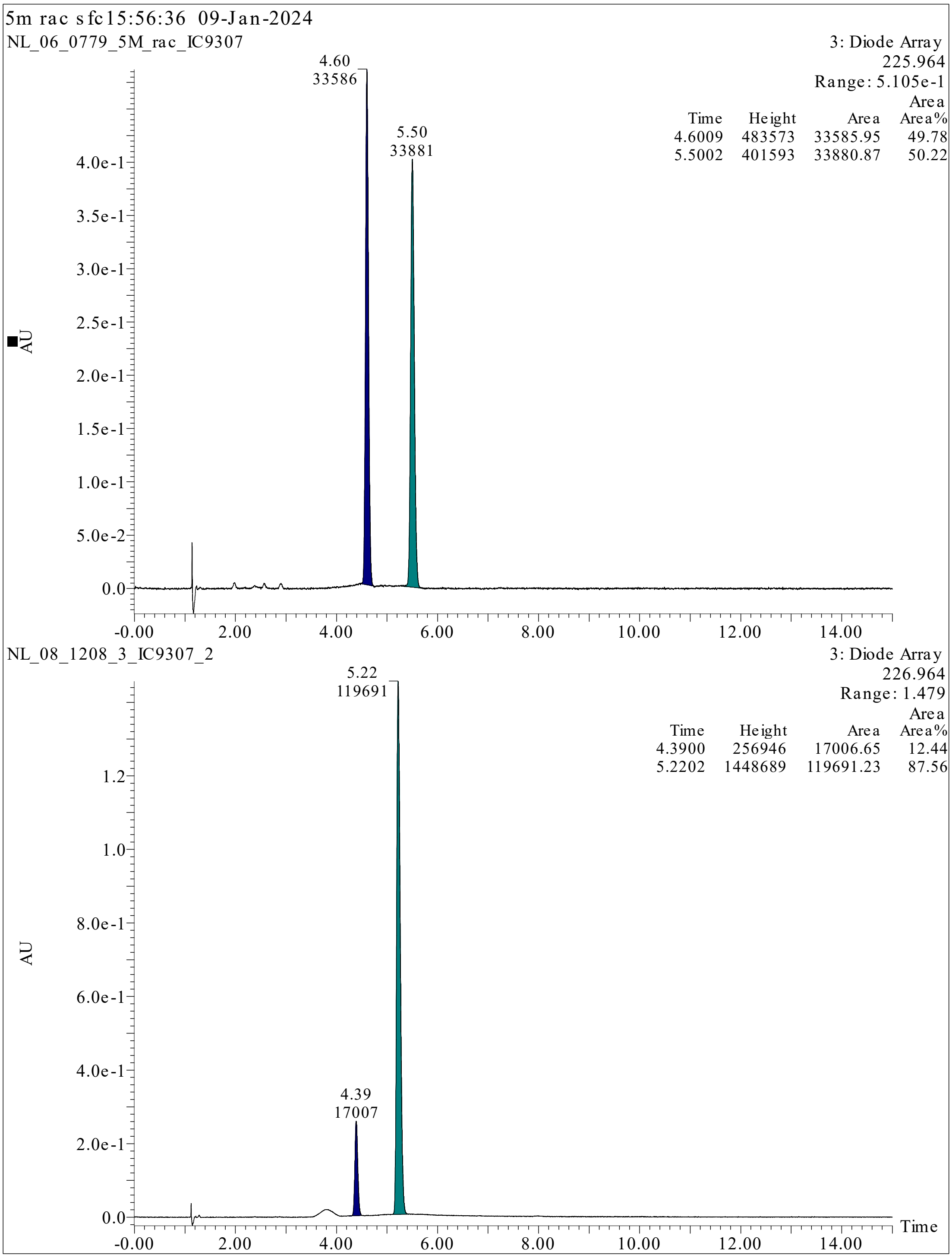

(*S*)-3-hydroxy-3,4-dihydronaphthalen-2(1*H*)-one (2e)

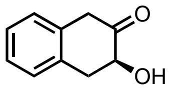

(2*R*,3*S*)-1,2,3,4-tetrahydronaphthalene-2,3-diol **1e** (16.4 mg, 0.10 mmol) was subjected to General Procedure **B**, with 4CzIPN (3.9 mg, 0.005 mmol), *epi*-NHBoc-DHCN (4.0 mg, 0.01 mmol), TBA·H_2PO_4 (8.5 mg, 0.025 mmol), DIAD (20 mg, 0.1 mmol) in MeCN (4 mL) and reacted for 24 h. Purification by column chromatography (SiO_2, 50–100% CH_2Cl_2/hexane) afforded **2e** as a white solid (8.2 mg, 0.051 mmol, 51%). Chiral HPLC analysis of **2e** indicated that it was formed in 93% ee. A sample of racemic **2e** was synthesized *via* General Procedure **E**.

^{1}H NMR (400 MHz, $CDCl_3$) δ 7.24 (q, *J* = 3.1 Hz, 3H), 7.17 – 7.09 (m, 1H), 4.43 (dd, *J* = 12.7, 7.0 Hz, 1H), 3.81 (s, 2H), 3.73 (br s, 1H), 3.39 (dd, *J* = 15.2, 7.0 Hz, 1H), 3.00 (dd, *J* = 15.0, 12.9 Hz, 1H)
^{13}C NMR (101 MHz, $CDCl_3$) δ 209.7, 133.1, 132.1, 128.9, 128.4, 127.3, 127.2, 73.3, 41.6, 37.4.
HRMS (ESI+) $[M+H]^+$ m/z calc'd for $[C_{10}H_8O_2H]^+$ expect 161.0603; found 161.0600.
$[\alpha]_D$: +133.4 (c = 0.47, T = 25 °C, $CHCl_3$)

Chiral HPLC Analysis: CHIRALPAK IG (*n*-hexane: *i*PrOH, 95:05, 1.25 mL min^{-1}, 40 °C, 206 nm) t_R = 9.98 (major), 10.32 (minor) minutes. 93% ee

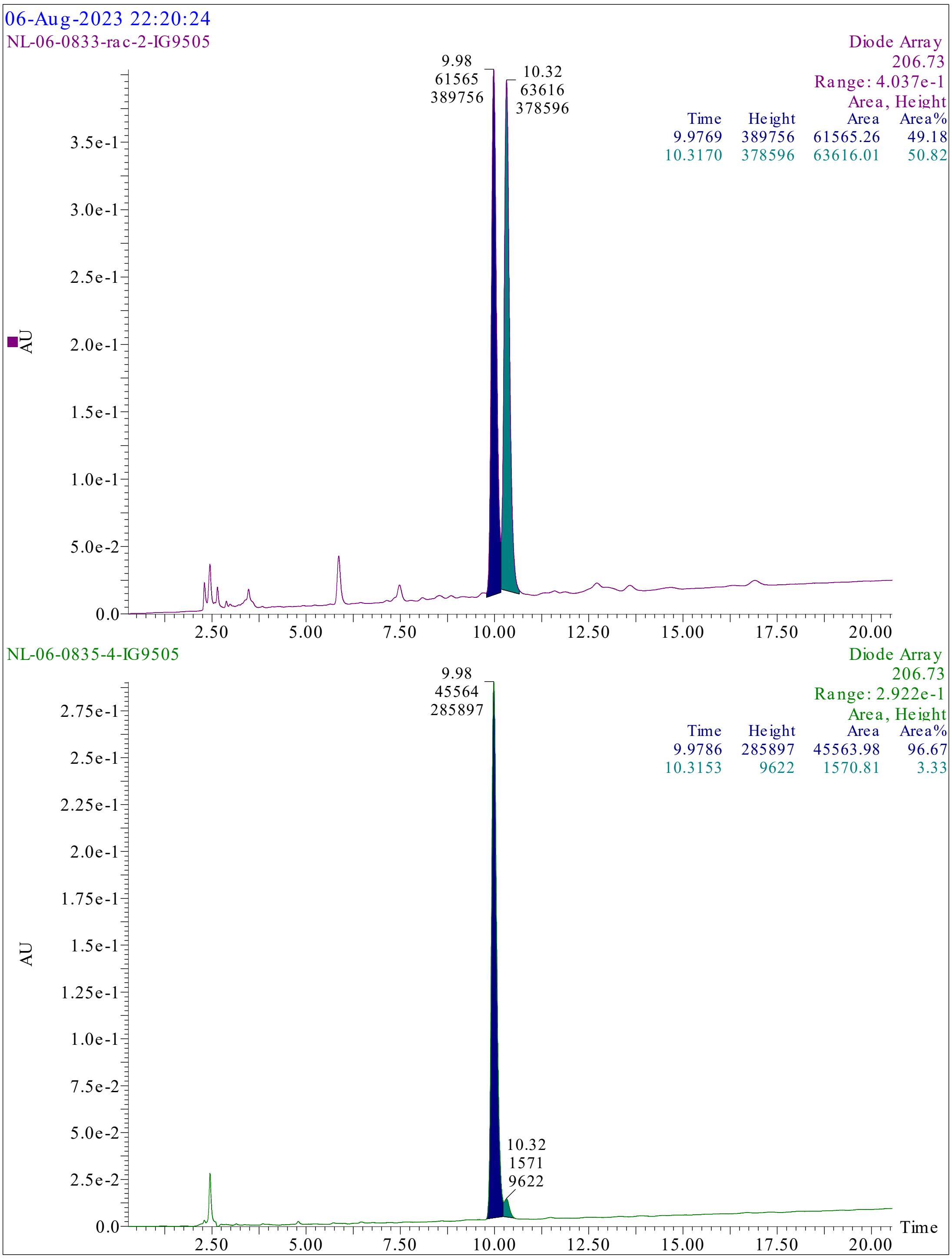

(*S*)-5-hydroxy-1-tosylazepan-4-one (2f)

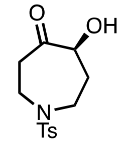

(4*R*,5*S*)-1-tosylazepane-4,5-diol **1f** (28.5 mg, 0.10 mmol) was subjected to General Procedure **B**, with 4CzIPN (3.9 mg, 0.005 mmol), *epi*-NHBoc-DHCN (4.0 mg, 0.01 mmol), TBA·H_2PO_4 (8.5 mg, 0.025 mmol), DIAD (20 mg, 0.1 mmol) in MeCN (4 mL) and reacted for 24 h. Purification by column chromatography (SiO_2, CH_2Cl_2 then 20–40% EtOAc/hexanes) afforded **2f** as a white solid (22.0 mg, 0.078 mmol, 78%). Chiral SFC analysis of **2e** indicated that it was formed in 93% ee. A sample of racemic **2e** was synthesized *via* General Procedure **E.**

^{1}H NMR (700 MHz, $CDCl_3$) δ 7.70 (d, *J* = 8.3 Hz, 2H), 7.36 (d, *J* = 7.9 Hz, 2H), 4.34 (dt, *J* = 11.0, 3.8 Hz, 1H), 3.96 – 3.82 (m, 2H), 3.79 (d, *J* = 4.2 Hz, 1H), 3.05 (dddd, *J* = 22.3, 13.8, 11.3, 2.4 Hz, 2H), 2.93 (ddd, *J* = 18.1, 11.0, 3.9 Hz, 1H), 2.75 (ddd, *J* = 18.2, 5.0, 2.6 Hz, 1H), 2.47 (s, 3H), 2.17 (ddd, *J* = 14.6, 6.0, 4.4 Hz, 1H), 1.87 (dtd, *J* = 14.7, 11.5, 3.5 Hz, 1H).
^{13}C NMR (176 MHz, $CDCl_3$) δ 210.0, 143.9, 135.6, 130.0, 127.1, 76.2, 47.5, 43.5, 41.2, 34.3, 21.5.
$[\alpha]_D$: +43.2 (c = 1.41, T = 25 °C, $CHCl_3$).

Data in agreement with literature reported for racemic **2f**[5]

Chiral SFC Analysis: CHIRALPAK IG (CO_2: MeOH, 65:35, 2.5 mL min^{-1}, 40 °C, 226 nm) t_R = 9.71 (minor), 10.21 (major) minutes. 93% ee

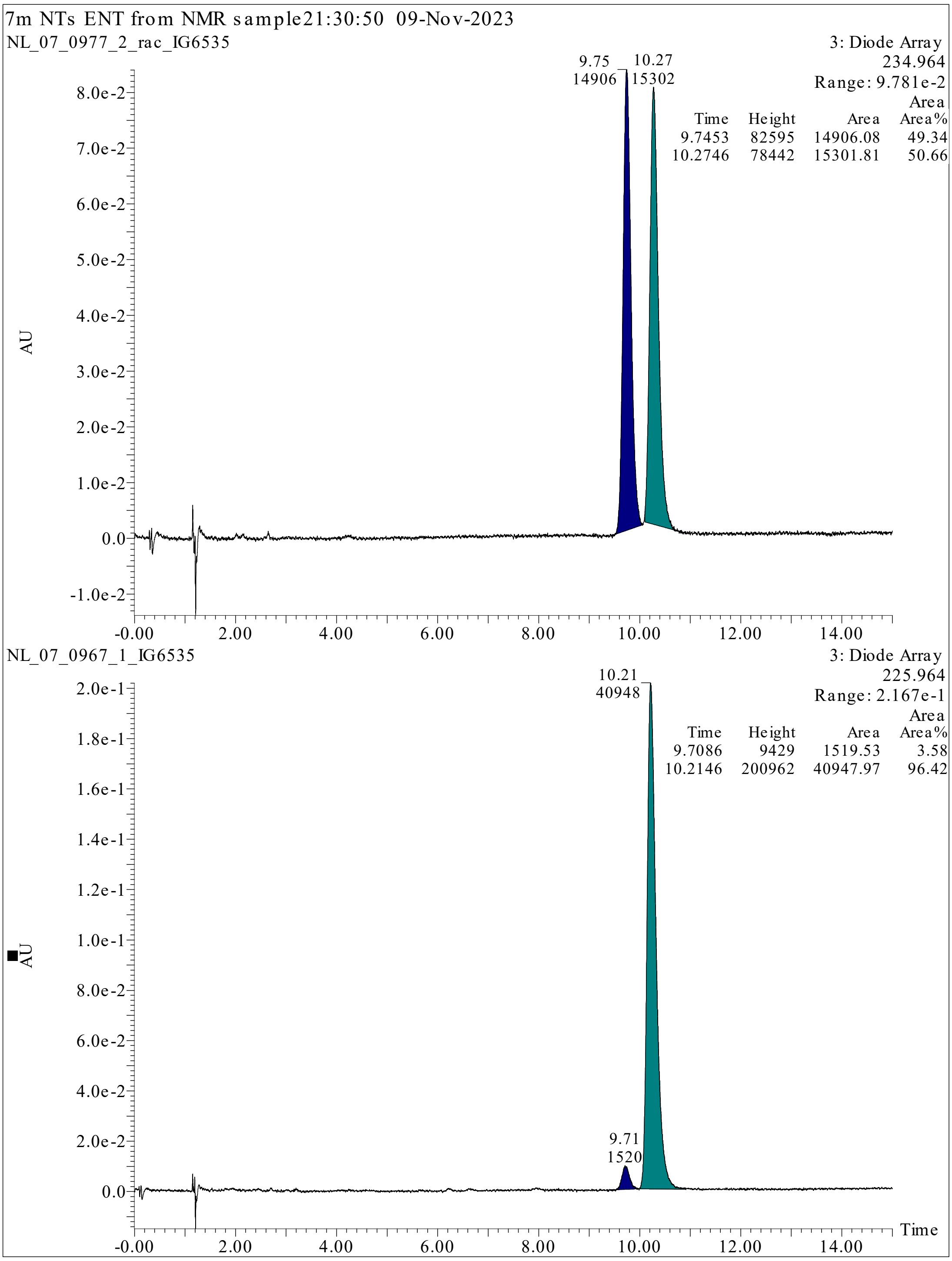

Diethyl (*S*)-4-hydroxy-5-oxocycloheptane-1,1-dicarboxylate (2g)

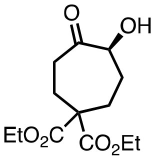

Diethyl (4*R*,5*S*)-4,5-dihydroxycycloheptane-1,1-dicarboxylate **1g** (27.4 mg, 0.10 mmol) was subjected to General Procedure **B**, with 4CzIPN (3.9 mg, 0.005 mmol), *epi*-NHBoc-DHCN (4.0 mg, 0.01 mmol), TBA·H_2PO_4 (8.5 mg, 0.025 mmol), DIAD (20 mg, 0.1 mmol) in MeCN (4 mL) and reacted for 24 h. Purification by column chromatography (SiO_2, CH_2Cl_2 then 5–10% acetone/CH_2Cl_2) afforded **2g** as a white solid (18.7 mg, 0.070 mmol, 70%).

^{1}H NMR (700 MHz, $CDCl_3$) δ δ 4.31 (dt, *J* = 8.8, 3.9 Hz, 1H), 4.28–4.18 (m, 4H), 3.77 (d, *J* = 3.4 Hz, 1H), 2.80 (ddd, *J* = 17.0, 8.5, 2.5 Hz, 1H), 2.56 (ddd, *J* = 17.0, 11.1, 2.7 Hz, 1H), 2.41 – 2.33 (m, 2H), 2.26 – 2.17 (m, 1H), 2.13 – 2.06 (m, 2H), 1.97 – 1.89 (m, 1H), 1.31–1.25 (m, 6H).
^{13}C NMR (176 MHz, $CDCl_3$) δ 211.8, 171.5, 170.4, 76.4, 61.8, 61.6, 57.0, 35.3, 30.7, 28.9, 28.2, 14.0.
$[\alpha]_D$: +32.5 (c = 1.21, T = 25 °C, $CHCl_3$)

Data in agreement with literature reported for racemic **2g**[5]

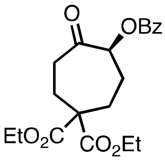

Derivatization for enantiomeric excess (ee) determination (**Bz-2g**)
Bz-2g was prepared via General Procedure **C1**. Purified by preparative thin layer chromatography (SiO_2, 20% EtOAc/CH_2Cl_2) afforded an analytical sample of **Bz-2g** for ee determination. Chiral SFC analysis indicated that **2f** was formed in 89% ee. A sample of racemic **Bz-2g** was synthesized *via* General Procedure **D1.**

^{1}H NMR (700 MHz, $CDCl_3$) δ δ 8.10 (dd, *J* = 8.4, 1.3 Hz, 2H), 7.67 – 7.54 (m, 1H), 7.48 (t, *J* = 7.9 Hz, 2H), 5.57 (t, *J* = 6.6 Hz, 1H), 4.33 – 4.05 (m, 4H), 2.81 (ddd, *J* = 16.6, 8.0, 3.2 Hz, 1H), 2.58 (ddd, *J* = 16.6, 10.7, 3.3 Hz, 1H), 2.53 – 2.40 (m, 2H), 2.36 (ddd, *J* = 15.0, 7.9, 3.2 Hz, 1H), 2.27 – 2.21 (m, 3H), 1.30 (dt, *J* = 14.1, 7.1 Hz, 6H).
^{13}C NMR (176 MHz, $CDCl_3$) δ 204.8, 171.2, 170.5, 165.6, 133.3, 129.9, 129.5, 128.4, 77.8, 61.9, 61.8, 56.9, 36.4, 30.7, 28.0, 26.0, 14.0.
HRMS (ESI+) $[M+Na]^+$ m/z calc'd for $[C_{20}H_{24}O_7Na]^+$ expect 399.1420; found 399.1409.

Chiral SFC Analysis: CHIRALPAK IC (CO_2: MeOH, 90:10, 2.5 mL min^{-1}, 40 °C, 220 nm) t_R = 5.19 (minor), 6.77 (major) minutes. 89% ee

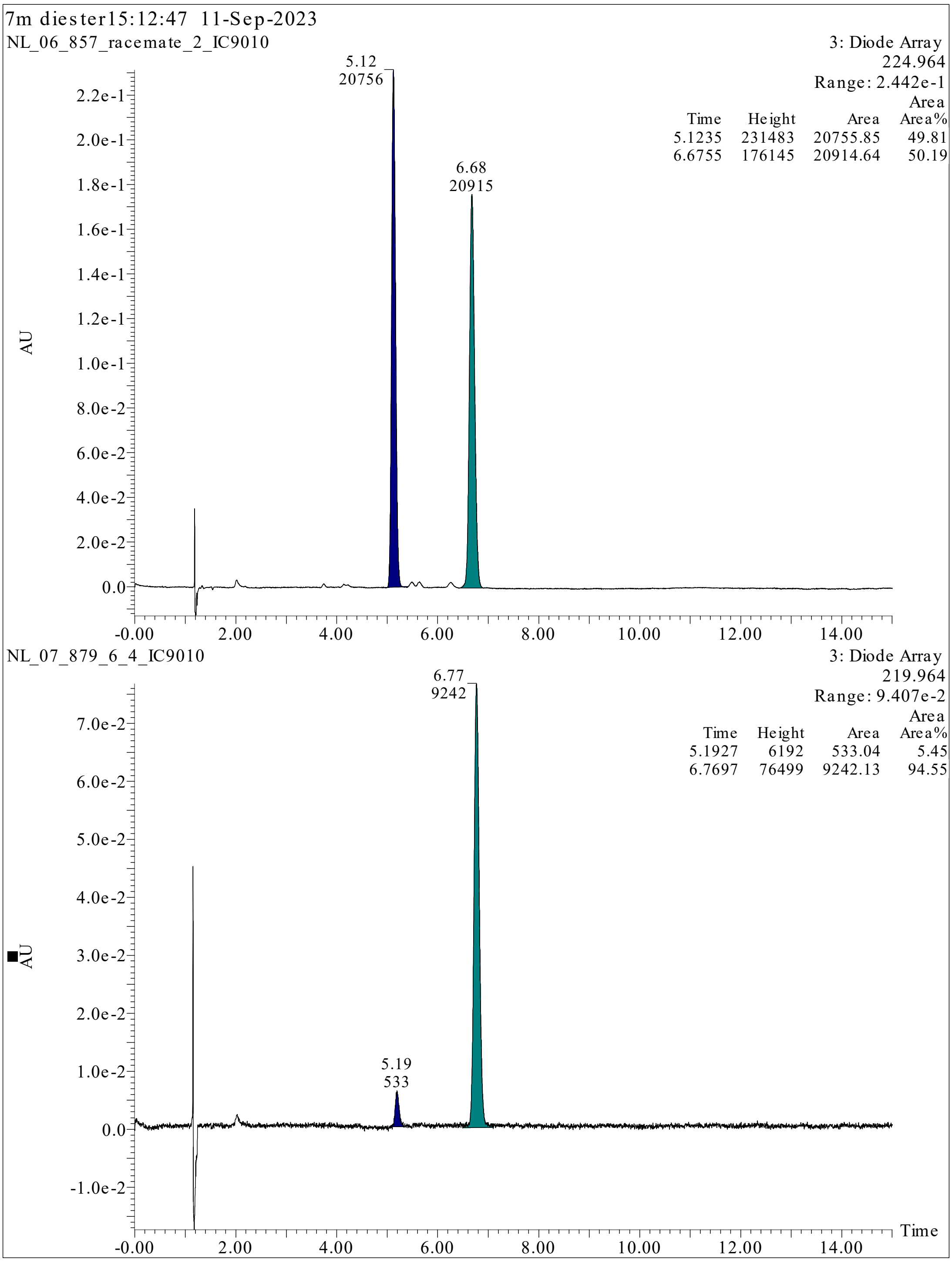

(*S*)-(4-hydroxy-5-oxocycloheptane-1,1-diyl)bis(methylene) dibenzoate (2h)

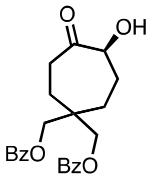

((4*R*,5*S*)-4,5-Dihydroxycycloheptane-1,1-diyl)bis(methylene) dibenzoate **1h** (19.9 mg, 0.05 mmol) was subjected to General Procedure **B**, with 4CzIPN (2.0 mg, 0.0025 mmol), *epi*-NHBoc-DHCN (2.0 mg, 0.005 mmol), TBA·H_2PO_4 (4.8 mg, 0.0125 mmol), DIAD (10 mg, 0.05 mmol) in MeCN (2 mL) and reacted for 24 h. Purification by column chromatography (SiO_2, CH_2Cl_2 then 20% EtOAc/hexanes) afforded **2h** as a white solid (13.8 mg, 0.070 mmol, 70%). Chiral SFC analysis indicated that **2h** was formed in 79% ee. A sample of racemic **2h** was synthesized *via* General Procedure **E.**

^{1}H NMR (700 MHz, $CDCl_3$) δ 8.12 – 7.92 (m, 4H), 7.65 – 7.55 (m, 2H), 7.47 (td, *J* = 7.8, 2.5 Hz, 4H), 4.50 – 4.23 (m, 5H), 3.84 (br s, 1H), 2.84 – 2.55 (m, 2H), 2.24 – 2.14 (m, 1H), 2.00 (dd, *J* = 12.4, 8.5 Hz, 2H), 1.93 (d, *J* = 3.7 Hz, 1H), 1.88 – 1.72 (m, 2H).
^{13}C NMR (176 MHz, $CDCl_3$) δ 211.7, 166.3, 166.2, 133.3, 129.6, 128.6, 76.8, 68.3, 65.2, 40.0, 34.4, 29.6, 28.1, 26.8.
[α]$_D$: +12.9 (c = 0.81, T = 25 °C, $CHCl_3$)
HRMS (ESI+) $[M+Na]^+$ m/z calc'd for $[C_{23}H_{24}O_6Na]^+$ expect 419.1471; found 419.1459.

Chiral SFC Analysis: CHIRALPAK IG (CO_2: MeOH, 70:30, 2.5 mL min^{-1}, 40 °C, 226 nm) t_R = 8.93 (minor), 9.87 (major) minutes. 79% ee

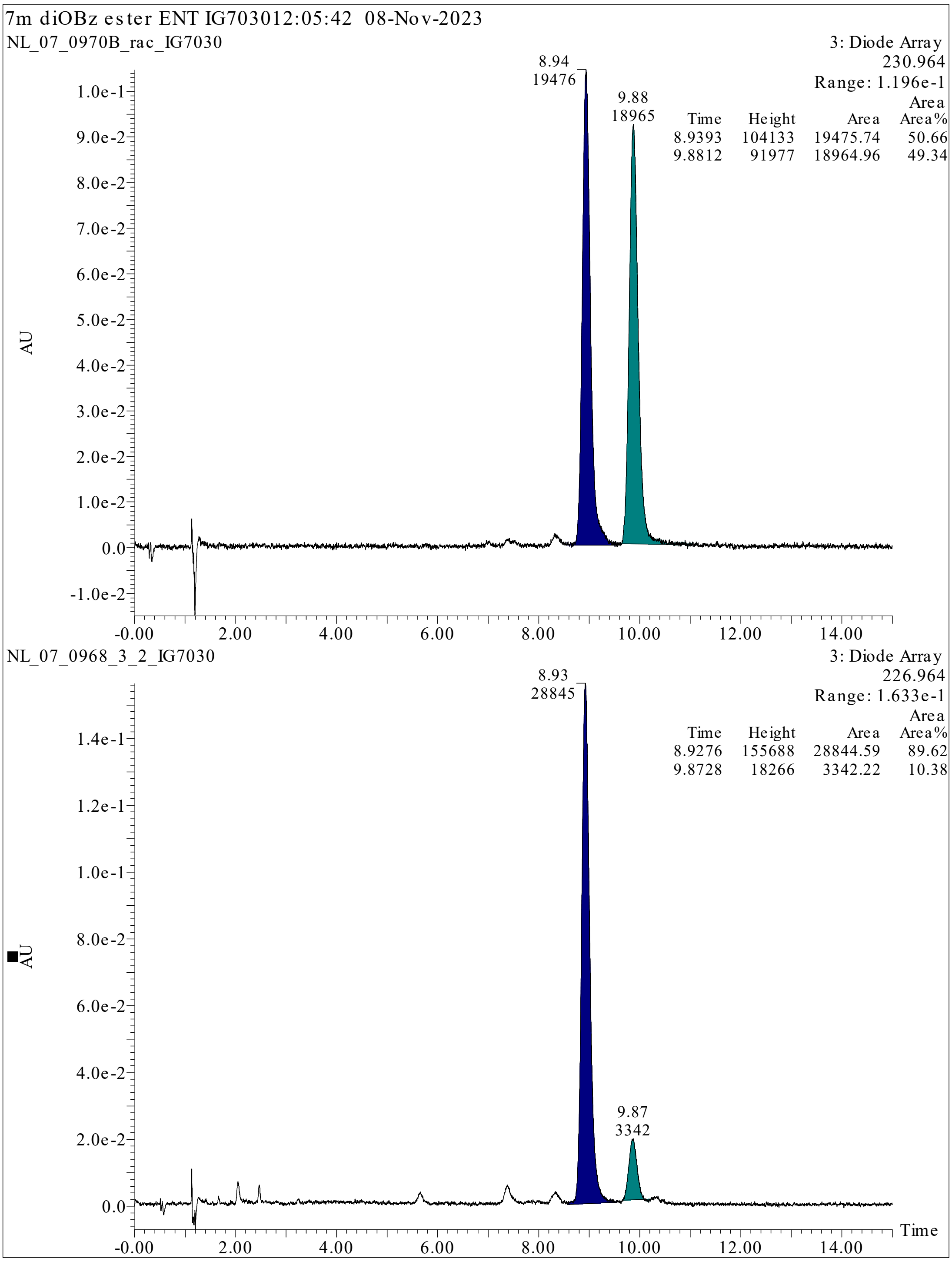

(*S*)-10-hydroxy-3,3-dimethyl-2,4-dioxaspiro[5.6]dodecan-9-one (2i)

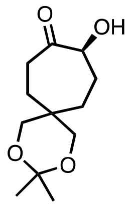

(9*R*,10*S*)-3,3-Dimethyl-2,4-dioxaspiro[5.6]dodecane-9,10-diol **1i** (23.0 mg, 0.10 mmol) was subjected to General Procedure **B**, with 4CzIPN (3.9 mg, 0.005 mmol), *epi*-NHBoc-DHCN (4.0 mg, 0.01 mmol), TBA·H_2PO_4 (8.5 mg, 0.025 mmol), DIAD (20 mg, 0.10 mmol) in MeCN (4 mL) and reacted for 24 h. Purification by column chromatography (SiO_2, CH_2Cl_2 then 20–30% EtOAc/hexanes) afforded **2h** as a white solid (17.8 mg, 0.078 mmol, 78%).

^{1}H NMR (700 MHz, $CDCl_3$) δ 4.29 (dt, *J* = 10.1, 4.1 Hz, 1H), 3.81 (d, *J* = 4.0 Hz, 1H), 3.64 (d, *J* = 11.6 Hz, 1H), 3.63–3.56 (m, 2H), 3.53 (dd, *J* = 11.6, 1.4 Hz, 1H), 2.66–2.50 (m, 2H), 2.09–2.00 (m, 1H), 2.01–1.89 (m, 1H), 1.85–1.71 (m, 2H), 1.60-1.54 (m, 1H), 1.50–1.45 (m, 1H), 1.44 (s, 3H), 1.43 (s, 3H).
^{13}C NMR (176 MHz, $CDCl_3$) δ 212.7, 98.5, 76.8, 69.6, 65.9, 34.7, 34.6, 31.1, 28.0, 27.0, 25.4, 22.0.
$[\alpha]_D$: +26.4 (c = 1.07, T = 25 °C, $CHCl_3$)
HRMS (ESI+) $[M+Na]^+$ m/z calc'd for $[C_{12}H_{20}O_4Na]^+$ expect 251.1259; found 251.1257.

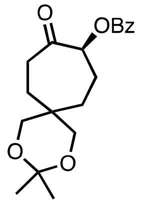

Derivatization for enantiomeric excess (ee) determination (**Bz-2i**)
Bz-2i was prepared via General Procedure **C1**. Purified by preparative thin layer chromatography (SiO_2, 20% EtOAc/CH_2Cl_2) afforded an analytical sample of **Bz-2i** for ee determination. Chiral SFC analysis indicated that **2i** was formed in 82% ee. A sample of racemic **Bz-2i** was synthesized *via* General Procedure **D1.**

^{1}H NMR (700 MHz, $CDCl_3$) δ δ 8.12 – 7.96 (m, 2H), 7.66 – 7.51 (m, 1H), 7.48 – 7.42 (m, 2H), 5.47 (dd, *J* = 10.0, 4.0 Hz, 1H), 3.68 – 3.48 (m, 4H), 2.66 (ddd, *J* = 17.4, 6.3, 4.2 Hz, 1H), 2.54 (ddd, *J* = 17.4, 11.5, 4.3 Hz, 1H), 2.18 – 2.08 (m, 1H), 2.01 (dt, *J* = 14.9, 5.1 Hz, 1H), 1.98 – 1.87 (m, 2H), 1.83 (ddd, *J* = 15.4, 11.5, 4.2 Hz, 1H), 1.60 (ddd, *J* = 14.4, 11.3, 3.3 Hz, 1H), 1.42 (s, 3H), 1.42 (s, 3H).
^{13}C NMR (176 MHz, $CDCl_3$) δ 206.2, 165.7, 133.3, 129.9, 129.5, 128.4, 98.4, 78.3, 69.3, 65.9, 36.0, 35.0, 30.8, 27.0, 25.0.
HRMS (ESI+) $[M+H]^+$ m/z calc'd for $[C_{19}H_{24}O_5H]^+$ expect 333.1699; found 333.1697

Chiral SFC Analysis: CHIRALPAK IG (CO_2: MeOH, 65:35, 2.5 mL min^{-1}, 40 °C, 220 nm) t_R = 9.24 (minor), 11.79 (major) minutes. 82% ee

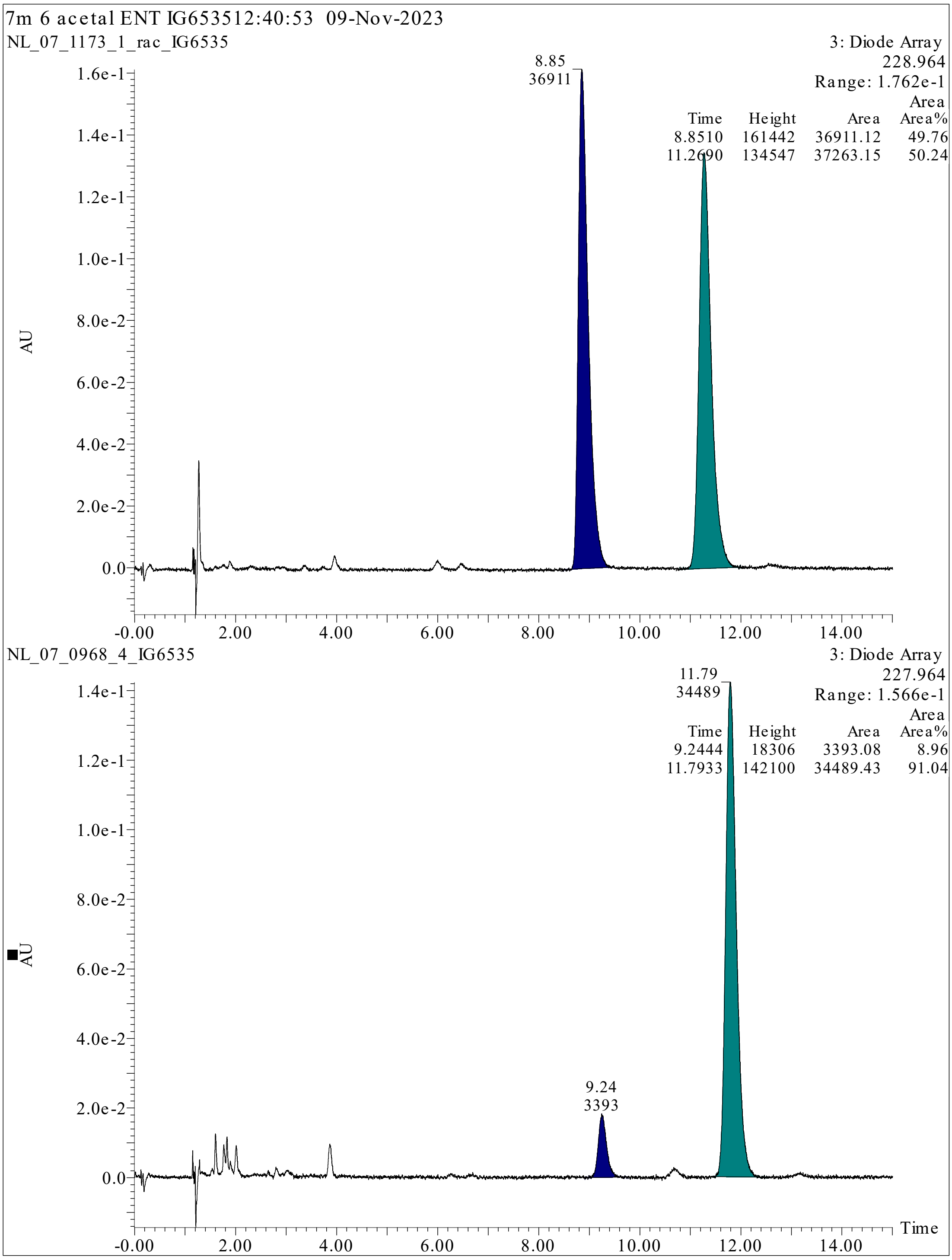

(*S*)-9-hydroxy-1,4-dioxaspiro[4.6]undecan-8-one (2j)

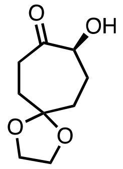

(8*R*,9*S*)-1,4-Dioxaspiro[4.6]undecane-8,9-diol **1j** (18.8 mg, 0.10 mmol) was subjected to General Procedure **B**, with 4CzIPN (3.9 mg, 0.005 mmol), *epi*-NHBoc-DHCN (4.0 mg, 0.01 mmol), TBA·H_2PO_4 (8.5 mg, 0.025 mmol), DIAD (20 mg, 0.10 mmol) in MeCN (4 mL) and reacted for 24 h. Purification by column chromatography (SiO_2, CH_2Cl_2 then 30% EtOAc/hexanes) afforded **2j** as a white solid (18.6 mg, 0.087 mmol, 87%).

^{1}H NMR (700 MHz, $CDCl_3$) δ 4.29 (dt, *J* = 9.3, 3.5 Hz, 1H), 4.07 – 3.85 (m, 4H), 3.82 (d, *J* = 3.4 Hz, 1H), 2.78 – 2.53 (m, 2H), 2.15 – 1.95 (m, 2H), 1.93 – 1.71 (m, 4H).
^{13}C NMR (176 MHz, $CDCl_3$) δ 212.6, 109.3, 76.9, 64.7, 64.4, 35.3, 34.4, 32.5, 28.4.
$[\alpha]_D$: +31.4 (c = 0.95, T = 25 °C, $CHCl_3$)

Data in agreement with literature reported for racemic **2j**[8]

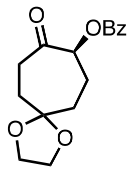

Derivatization for enantiomeric excess (ee) determination (**Bz-2j**)
Bz-2j was prepared via General Procedure **C1**. Purified by preparative thin layer chromatography (SiO_2, CH_2Cl_2) afforded an analytical sample of **Bz-2j** for ee determination. Chiral SFC analysis indicated that **2j** was formed in 90% ee. A sample of racemic **Bz-2j** was synthesized *via* General Procedure **D1**.

^{1}H NMR (700 MHz, $CDCl_3$) δ 8.08 (dd, *J* = 8.3, 1.2 Hz, 2H), 7.65 – 7.52 (m, 1H), 7.50 – 7.36 (m, 2H), 5.52 (dd, *J* = 9.1, 3.5 Hz, 1H), 4.05 – 3.84 (m, 4H), 2.74 – 2.55 (m, 2H), 2.24 (ddt, *J* = 14.6, 7.3, 3.4 Hz, 1H), 2.16 – 2.07 (m, 2H), 2.05 – 1.93 (m, 3H).
^{13}C NMR (176 MHz, $CDCl_3$) δ 205.5, 165.7, 133.3, 129.9, 129.6, 128.4, 109.4, 78.4, 64.7, 64.5, 35.5, 35.1, 32.5, 25.6.
HRMS (ESI+) $[M+Na]^+$ m/z calc'd for $[C_{16}H_{18}O_5Na]^+$ expect 313.1052; found 313.1051

Chiral SFC Analysis: CHIRALPAK IC (CO_2: MeOH, 85:15, 2.5 mL min^{-1}, 40 °C, 220 nm) t_R = 4.47 (minor), 6.02 (major) minutes. 90% ee

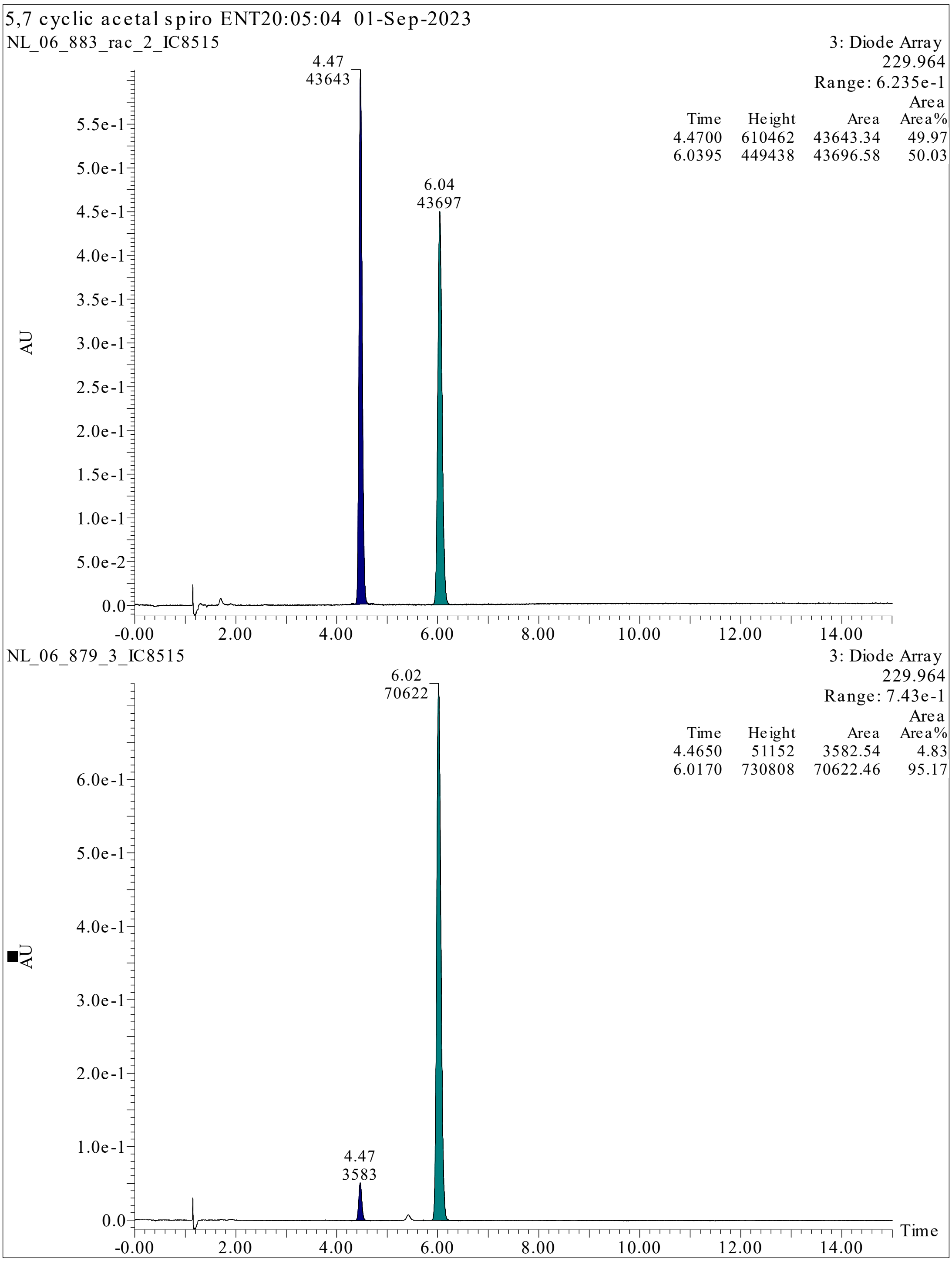

(*S*)-6-hydroxycyclohex-3-en-1-one (2k)

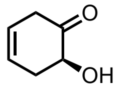

(1*R*,2*S*)-Cyclohex-4-ene-1,2-diol **1k** (11.4 mg, 0.10 mmol) was subjected to General Procedure **B**, with 4CzIPN (3.9 mg, 0.005 mmol), *epi*-NHBoc-DHCN (4.0 mg, 0.01 mmol), TBA·H_2PO_4 (8.5 mg, 0.025 mmol), DIAD (20 mg, 0.10 mmol) in MeCN (4 mL) and reacted for 24 h. Purification by column chromatography (SiO_2, 50–100% CH_2Cl_2/hexanes) afforded **2k** as a white solid (8.5 mg, 0.077 mmol, 77%). Note: **2k** is unstable over time and must be analyzed immediately after synthesis.

^{1}H NMR (700 MHz, $CDCl_3$) δ 5.81 – 5.65 (m, 2H), 4.45 (t, *J* = 8.9 Hz, 1H), 3.70 (d, *J* = 3.6 Hz, 1H), 3.18 – 3.07 (m, 1H), 3.06 – 2.98 (m, 1H), 2.92 (ddd, *J* = 16.9, 8.1, 5.7 Hz, 1H), 2.39 – 2.21 (m, 1H).
^{13}C NMR (176 MHz, $CDCl_3$) δ 209.7, 124.6, 123.9, 73.0, 38.9, 34.8.
$[\alpha]_D$: +2.8 (c = 0.54, T = 25 °C, $CHCl_3$)
HRMS (ESI+) $[M+Na]^+$ m/z calc'd for $[C_6H_8O_2Na]^+$ expect 135.0420; found 135.0420

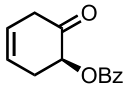

Derivatization for enantiomeric excess (ee) determination (**Bz-2k**)
Bz-2j was prepared via General Procedure **C1**. Purified by preparative thin layer chromatography (SiO_2, 20% CH_2Cl_2/hexanes) afforded an analytical sample of **Bz-2k** for ee determination. Chiral HPLC analysis indicated that **2k** was formed in 90% ee. A sample of racemic **Bz-2k** was synthesized *via* General Procedure **D1**. Note: **Bz-2k** is unstable over time and must be analyzed immediately after synthesis

^{1}H NMR (700 MHz, $CDCl_3$) δ 8.17 – 8.04 (m, 2H), 7.64 – 7.52 (m, 1H), 7.54 – 7.41 (m, 2H), 5.88 – 5.74 (m, 2H), 5.69 (dd, *J* = 11.4, 7.7 Hz, 1H), 3.24 (dt, *J* = 21.1, 3.0 Hz, 1H), 3.10 – 3.00 (m, 1H), 2.94 (ddd, *J* = 16.7, 7.7, 5.6 Hz, 1H), 2.76 (dddq, *J* = 16.7, 11.4, 5.7, 2.7 Hz, 1H).
^{13}C NMR (176 MHz, $CDCl_3$) δ 202.4, 165.6, 133.3, 129.9, 129.5, 128.4, 124.8, 124.0, 74.6, 40.4, 32.0.
HRMS (ESI+) $[M+NH_4]^+$ m/z calc'd for $[C_{13}H_{12}O_3NH_4]^+$ expect 234.1130; found 234.1128

Chiral HPLC Analysis: CHIRALPAK IG (*n*-hexane: *i*PrOH, 95:05, 2.5 mL min^{-1}, 40 °C, 236 nm) t_R = 13.30 (minor), 13.97 (minor) minutes. 90% ee

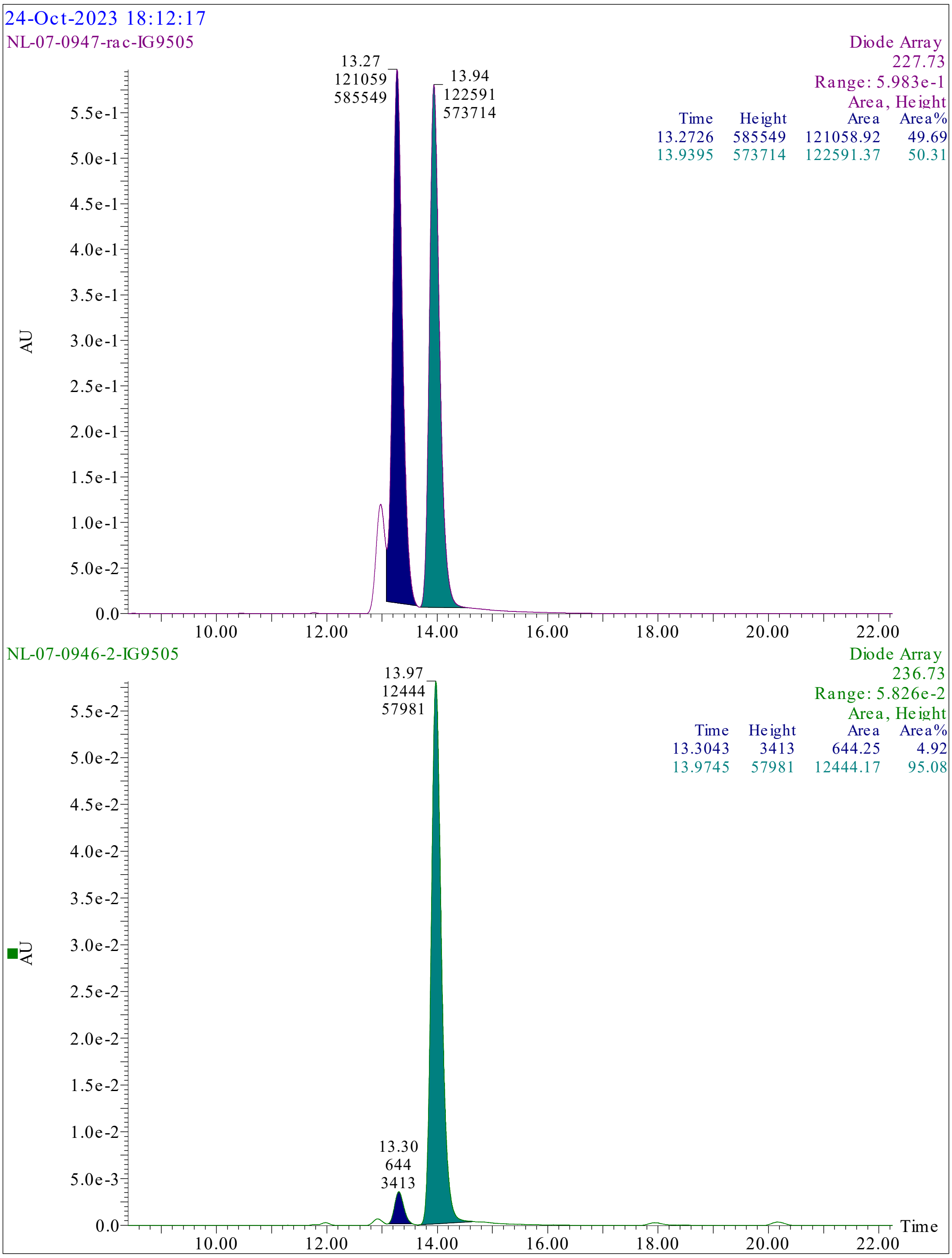

(*S*,*Z*)-8-hydroxycyclooct-4-en-1-one (2l)

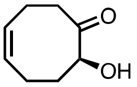

(1*R*,2*S*,*Z*)-Cyclooct-5-ene-1,2-diol **1l** (14.2 mg, 0.10 mmol) was subjected to General Procedure **B**, with 4CzIPN (3.9 mg, 0.005 mmol), *epi*-NHBoc-DHCN (4.0 mg, 0.01 mmol), TBA·H_2PO_4 (8.5 mg, 0.025 mmol), DIAD (20 mg, 0.1 mmol) in MeCN (4 mL) and reacted for 36 h. Purification by column chromatography (SiO_2, 50–100% CH_2Cl_2/hexane) afforded **2l** (10.1 mg, 0.072 mmol, 72%) as a clear oil.

Accessing product enantiomer (*ent*-**2l**)
1l (14.2 mg, 0.10 mmol) was subjected to General Procedure **B**, with 4CzIPN (3.9 mg, 0.005 mmol), *epi*-NHBoc-DHCD (4.0 mg, 0.01 mmol), TBA·H_2PO_4 (8.5 mg, 0.025 mmol) in MeCN (4 mL) and reacted for 36 h. Purification by column chromatography (SiO_2, 50–100% CH_2Cl_2/hexane) afforded *ent*-**2l** (12.9 mg, 0.092 mmol, 92%) as a clear oil.

^{1}H NMR (400 MHz, $CDCl_3$) δ 5.80 – 5.55 (m, 2H), 4.33 (td, *J* = 8.5, 4.1 Hz, 1H), 2.87 (d, *J* = 7.6 Hz, 1H), 2.72 (ddd, *J* = 14.4, 11.0, 4.7 Hz, 1H), 2.67 – 2.51 (m, 2H), 2.52 – 2.33 (m, 1H), 2.30–2.22 (m, 2H), 2.12–1.96 (m, 1H), 1.58–1.45 (m, 1H).
^{13}C NMR (101 MHz, $CDCl_3$) δ 215.3, 130.6, 129.9, 75.9, 43.1, 34.0, 21.9, 20.9.
[α]$_D$: +27.5 (**2l**, c = 0.47, T = 25 °C, $CHCl_3$); **[α]$_D$**: –29.4 (*ent*-**2l**, c = 0.84, T = 25 °C, $CHCl_3$).

Data in agreement with literature reported for racemic **2l**[27]

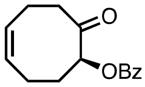

Derivatization for enantiomeric excess (ee) determination (**Bz-2l**)
Bz-2l and **Bz-*ent*-2l** was prepared via General Procedure **C1**. Purified by preparative thin layer chromatography (SiO_2, 20% CH_2Cl_2/hexanes) afforded an analytical sample of **Bz-2l** and **Bz-*ent*-2l** for ee determination. A sample of racemic **Bz-2l** was synthesized *via* General Procedure **D1**.

^{1}H NMR (400 MHz, $CDCl_3$) δ 8.10 (dd, *J* = 8.2, 1.1 Hz, 2H), 7.65 – 7.54 (m, 1H), 7.52 – 7.43 (m, 2H), 5.84 – 5.70 (m, 2H), 5.32 (dd, *J* = 7.6, 4.0 Hz, 1H), 2.74 (m, 3H), 2.51 (dddd, *J* = 13.9, 10.4, 7.3, 3.8 Hz, 1H), 2.44 – 2.31 (m, 1H), 2.20 (dtd, *J* = 14.0, 6.9, 3.6 Hz, 1H), 2.06 – 1.89 (m, 2H).
^{13}C NMR (101 MHz, $CDCl_3$) δ 210.0, 166.0, 133.5, 130.7, 130.3, 129.8, 129.4, 128.6, 78.5, 44.0, 29.3, 21.3, 21.2.
HRMS (ESI+) $[M+H]^+$ m/z calc'd for $[C_{15}H_{16}O_3H]^+$ expect 245.1178; found 245.1171

Chiral SFC Analysis: CHIRALPAK IG (CO_2: MeOH, 90:10, 2.5 mL min^{-1}, 40 °C, 228 nm)

Bz-2l (middle): t_R = 7.96 (major), 9.47 (minor) minutes, 94% ee

Bz-*ent*-2l *via epi-NHBoc-DHCN (bottom):* t_R = 7.93 (minor), 9.36 (major) minutes, –91% ee

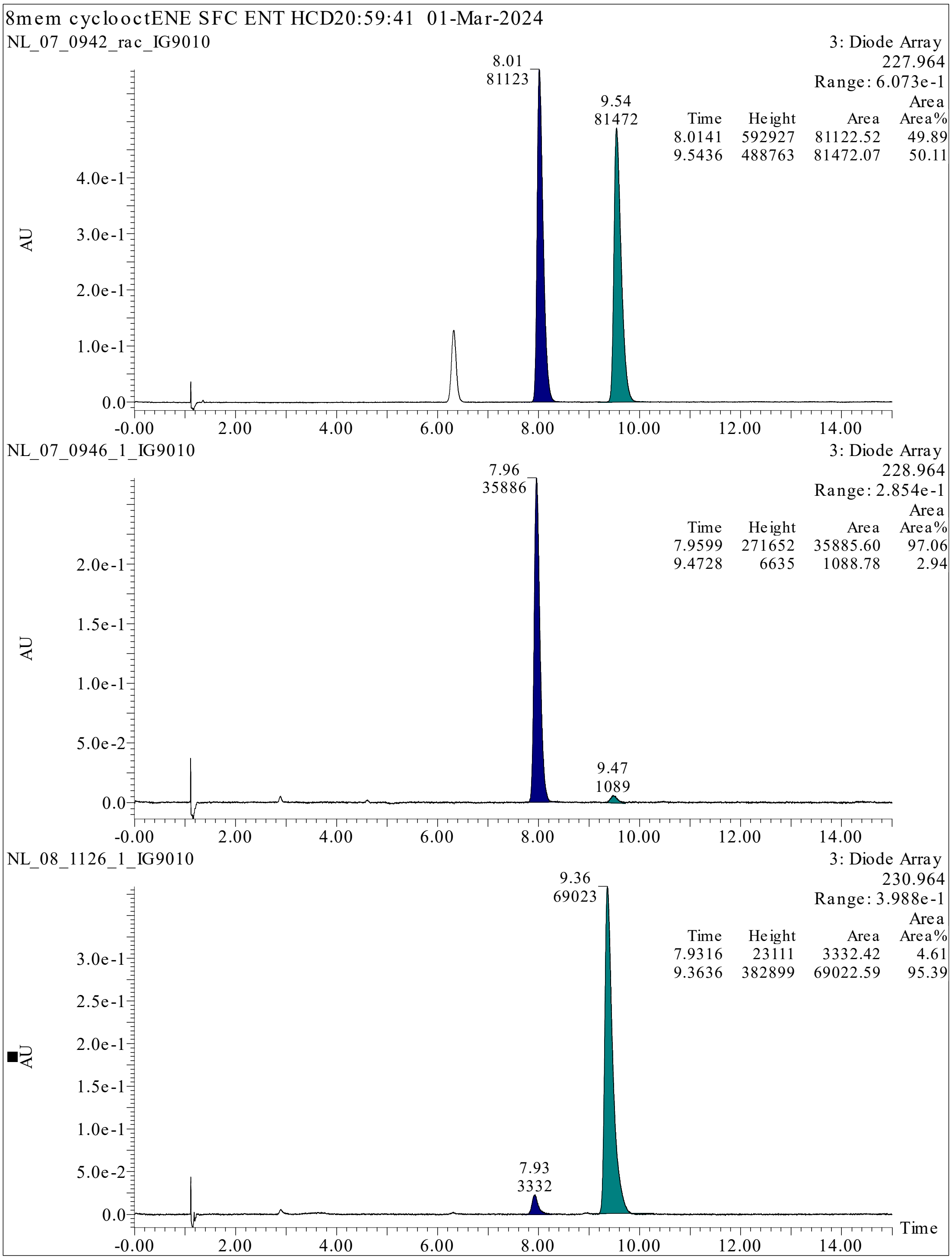

(3a*R*,6*S*,7a*S*)-6-hydroxyhexahydroisobenzofuran-5(3*H*)-one (2m)

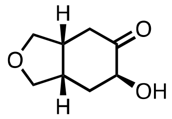

(3a*R*,5*R*,6*S*,7a*S*)-Octahydroisobenzofuran-5,6-diol **1m** (15.8 mg, 0.10 mmol) was subjected to General Procedure **B**, with 4CzIPN (3.9 mg, 0.005 mmol), *epi*-NHBoc-DHCN (4.0 mg, 0.01 mmol), TBA·H_2PO_4 (8.5 mg, 0.025 mmol), DIAD (20 mg, 0.10 mmol) in MeCN (4 mL) and reacted for 36 h. Purification by column chromatography (SiO_2, CH_2Cl_2 then 20–40% EtOAc/hexanes) afforded **2m** as a clear oil (11.5 mg, 0.074 mmol, 74%).

^{1}H NMR (700 MHz, $CDCl_3$) δ 4.34 (ddd, *J* = 10.6, 6.8, 3.4 Hz, 1H), 4.07 (t, *J* = 8.8 Hz, 1H), 3.93 (t, *J* = 8.8 Hz, 2H), 3.90 (dd, *J* = 8.7, 4.5 Hz, 1H), 3.67 (d, *J* = 9.7 Hz, 1H), 3.39 (d, *J* = 3.5 Hz, 1H), 2.72 – 2.62 (m, 2H), 2.64 – 2.58 (m, 1H), 2.54 (dd, *J* = 13.0, 10.7 Hz, 1H), 2.48 (ddd, *J* = 14.0, 6.9, 2.6 Hz, 1H), 1.91 (ddd, *J* = 14.0, 11.6, 5.5 Hz, 1H).
^{13}C NMR (176 MHz, $CDCl_3$) δ 211.1, 74.2, 70.5, 70.0, 41.8, 39.6, 37.3, 34.1.
$[\alpha]_D$: –11.7 (c = 0.92, T = 25 °C, $CHCl_3$)
HRMS (ESI+) $[M+H]^+$ m/z calc'd for $[C_8H_{12}O_3H]^+$ expect 157.0865; found 157.0860

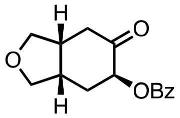

Derivatization for enantiomeric excess (ee) determination (**Bz-2m**)
Bz-2m was prepared via General Procedure **C1**. Purified by preparative thin layer chromatography (SiO_2, 40% EtOAc/hexanes) afforded an analytical sample of **Bz-2m** for ee determination. Chiral SFC analysis indicated that **2m** was formed in 84% ee. A sample of racemic **Bz-2m** was synthesized *via* General Procedure **D1**.

^{1}H NMR (700 MHz, $CDCl_3$) δ 8.15 – 7.99 (m, 2H), 7.58 (t, *J* = 7.4 Hz, 1H), 7.46 (t, *J* = 7.8 Hz, 2H), 5.54 (dd, *J* = 11.0, 6.1 Hz, 1H), 4.09 (t, *J* = 8.6 Hz, 1H), 3.98 (t, *J* = 8.6 Hz, 1H), 3.94 (dd, *J* = 8.8, 5.3 Hz, 1H), 3.69 (dd, *J* = 8.8, 2.6 Hz, 1H), 2.80 (s, 1H), 2.76 – 2.69 (m, 1H), 2.70 – 2.58 (m, 2H), 2.42 (ddd, *J* = 13.9, 6.2, 4.1 Hz, 1H), 2.36 (ddd, *J* = 13.9, 11.0, 5.6 Hz, 1H).
^{13}C NMR (176 MHz, $CDCl_3$) δ 204.4, 165.6, 133.4, 129.9, 129.4, 128.4, 73.7, 72.9, 70.5, 41.1, 40.4, 36.7, 31.0.
HRMS (ESI+) $[M+H]^+$ m/z calc'd for $[C_{15}H_{16}O_4H]^+$ expect 261.1127; found 261.1121

Chiral SFC Analysis: CHIRALPAK IC (CO_2: MeOH, 85:15, 2.5 mL min^{-1}, 40 °C, 225 nm) t_R = 6.59 (minor), 7.58 (major) minutes. 84% ee

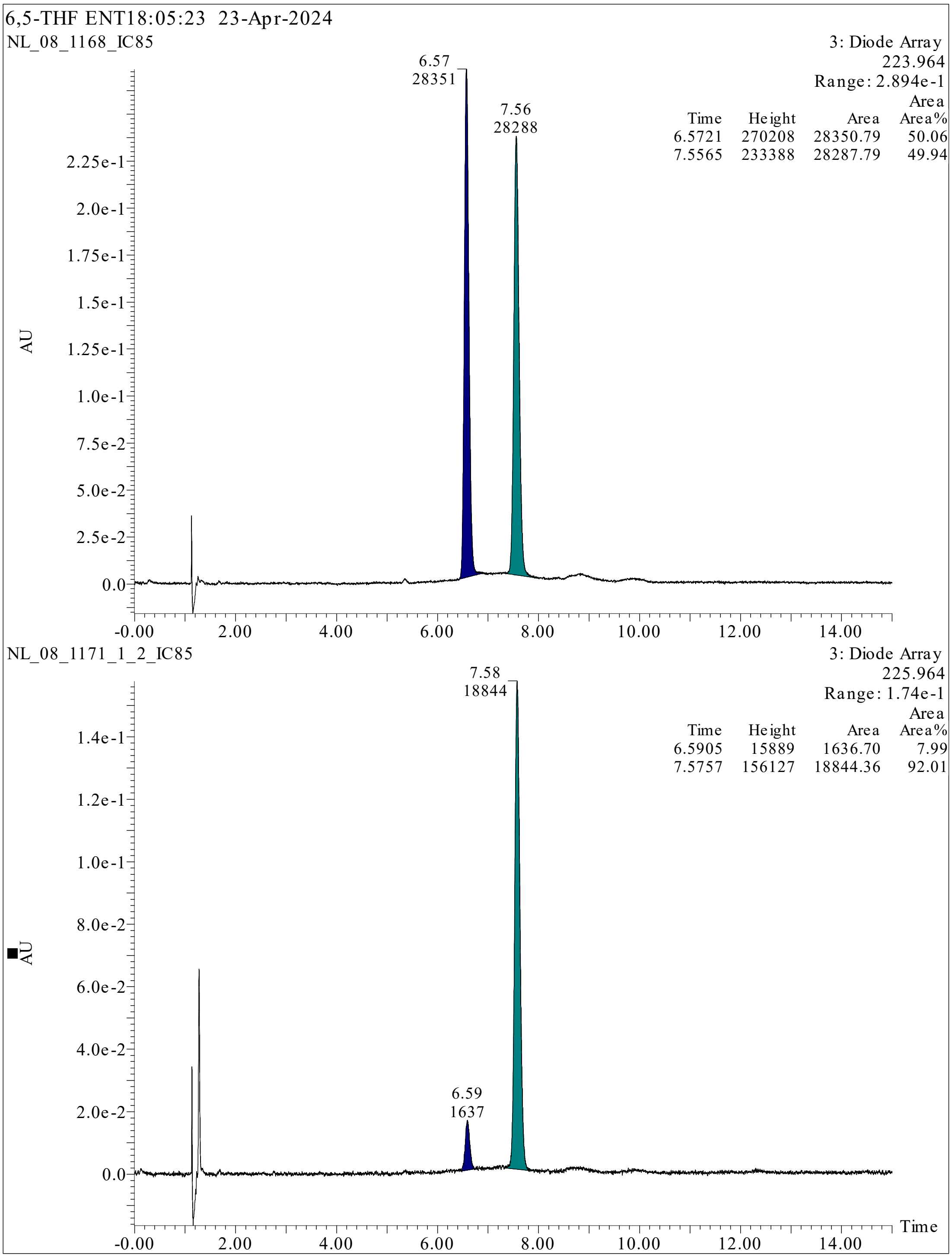

tert-butyl (3a*S*,5*S*,7a*R*)-5-hydroxy-6-oxooctahydro-1*H*-2λ4-isoindole-2-carboxylate (2n)

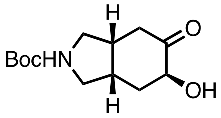

tert-Butyl (3a*R*,5*R*,6*S*,7a*S*)-5,6-dihydroxyoctahydro-1*H*-2λ4-isoindole-2-carboxylate **1n** (25.8 mg, 0.10 mmol) was subjected to General Procedure **B**, with 4CzIPN (3.9 mg, 0.005 mmol), *epi*-NHBoc-DHCN (4.0 mg, 0.01 mmol), TBA·H_2PO_4 (8.5 mg, 0.025 mmol), DIAD (20 mg, 0.10 mmol) in MeCN (4 mL) and reacted for 36 h. Purification by column chromatography (SiO_2, CH_2Cl_2 then 10–40% EtOAc/hexanes) afforded **2n** as a white solid (11.7 mg, 0.047 mmol, 47%).

^{1}H NMR (700 MHz, $CDCl_3$) δ 4.41 – 4.31 (m, 1H), 3.63 (m, 1H), 3.56 – 3.38 (m, 3H), 3.30 (m, 1H), 2.58 (m, 4H), 2.47 – 2.37 (m, 1H), 1.82 (td, *J* = 13.1, 4.9 Hz, 1H), 1.50 – 1.44 (m, 9H).
Combined ^{1}H NMR signals for both rotamers reported
^{13}C NMR (176 MHz, $CDCl_3$) δ 210.4, 210.3*, 154.9, 79.9, 79.8*, 70.6, 70.6*, 52.5, 52.0*, 47.1, 46.8*, 41.6, 40.8*, 40.2, 40.1*, 37.1, 36.3*, 35.0, 35.0*, 28.5, 28.5*.
Discernible ^{13}C signals due to rotamerism of carbamate C–N bond denoted by (*)
[α]$_D$: –6.7 (c = 0.53, T = 25 °C, $CHCl_3$)
HRMS (ESI+) $[M+H]^+$ m/z calc'd for $[C_{13}H_{21}NO_4H]^+$ expect 256.1549; found 256.1550

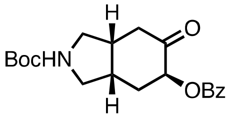

Derivatization for enantiomeric excess (ee) determination (**Bz-2n**)
Bz-2n was prepared via General Procedure **C1**. Purified by preparative thin layer chromatography (SiO_2, 10% EtOAc/CH_2Cl_2) afforded an analytical sample of **Bz-2n** for ee determination. Chiral SFC analysis indicated that **2n** was formed in 80% ee. A sample of racemic **Bz-2n** was synthesized *via* General Procedure **D1**.

^{1}H NMR (700 MHz, $CDCl_3$) δ 8.11 (d, *J* = 7.7 Hz, 2H), 7.61 (t, *J* = 7.3 Hz, 1H), 7.48 (t, *J* = 7.6 Hz, 2H), 5.61 + 5.53 (ddd, *J* = 11.5, 6.0 Hz, 1H), 3.75 – 3.56 (m, 2H), 3.52 (dt, *J* = 11.0, 5.6 Hz, 1H), 3.39 + 3.27 (dd, *J* = 11.3 Hz, 1H), 2.78 (s, 1H), 2.74 – 2.60 (m, 2H), 2.59 – 2.51 (m, 1H), 2.47 (d, *J* = 7.9 Hz, 1H), 2.36 – 2.29 (m, 1H), 1.50 (s, 9H). Combined signals (+) for carbamate rotamers reported
^{13}C NMR (176 MHz, $CDCl_3$) δ 203.9 + 203.4, 165.6 + 165.5, 154.8, 133.4, 129.9, 129.4, 128.4, 79.8, 73.3 + 72.9, 51.6, 47.5 + 47.4, 41.2 + 40.9, 40.6 + 40.4, 36.7, 35.6, 31.8 + 31.6, 28.5 + 28.5. Combined signals (+) for carbamate rotamers reported
HRMS (ESI+) $[M+Na]^+$ m/z calc'd for $[C_{20}H_{25}NO_5Na]^+$ expect 382.1630; found 382.1623.

Chiral SFC Analysis: CHIRALPAK IG (CO_2: MeOH, 85:15, 2.5 mL min^{-1}, 40 °C, 225 nm) t_R = 10.01 (major), 11.48 (minor) minutes. 80% ee

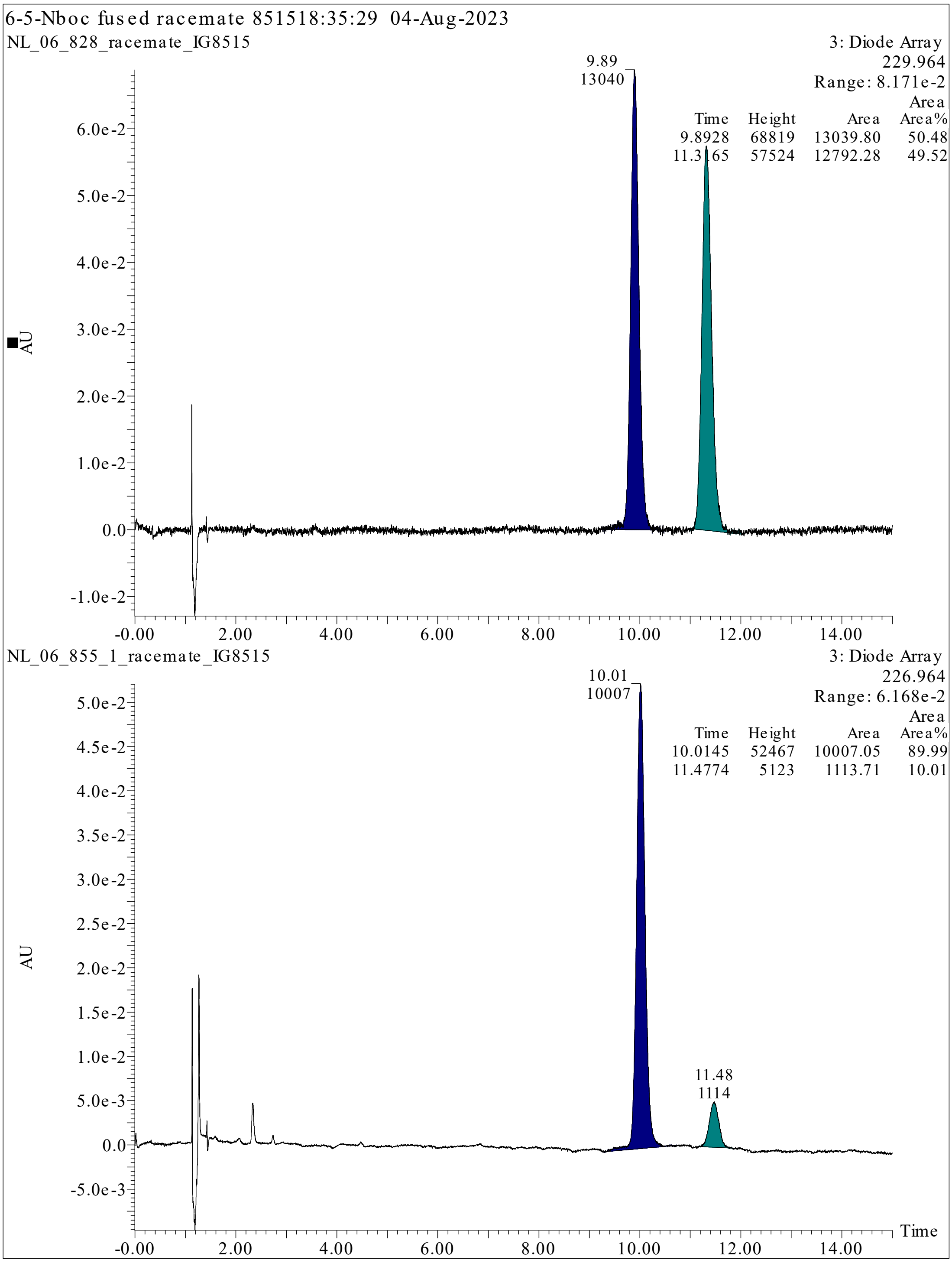

(3a*R*,6*S*,7a*S*)-6-hydroxy-2,2-dimethyltetrahydrobenzo[*d*][1,3]dioxol-5(4*H*)-one (2o)

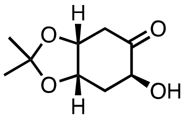

(3a*R*,5*R*,6*S*,7a*S*)-2,2-Dimethylhexahydrobenzo[*d*][1,3]dioxole-5,6-diol **1o** (18.8 mg, 0.10 mmol) was subjected to General Procedure **B**, with 4CzIPN (3.9 mg, 0.005 mmol), *epi*-NHBoc-DHCN (4.0 mg, 0.01 mmol), TBA·H_2PO_4 (8.5 mg, 0.025 mmol), DIAD (20 mg, 0.10 mmol) in MeCN (4 mL) and reacted for 36 h. Purification by column chromatography (SiO_2, CH_2Cl_2 then 10–40% EtOAc/hexanes) afforded **2o** as a clear oil (14.0 mg, 0.075 mmol, 75%).

^{1}H NMR (700 MHz, $CDCl_3$) δ 4.60 (ddd, *J* = 7.1, 4.2, 2.9 Hz, 1H), 4.58 – 4.49 (m, 1H), 4.40 (ddd, *J* = 13.3, 5.3, 2.2 Hz, 1H), 3.28 (d, *J* = 2.5 Hz, 1H), 2.87 (dd, *J* = 18.6, 2.6 Hz, 1H), 2.63 (dd, *J* = 18.6, 4.3 Hz, 1H), 2.44 (ddd, *J* = 14.3, 5.4, 1.6 Hz, 1H), 1.79 – 1.68 (m, 1H), 1.50 (s, 3H), 1.36 (s, 3H).
^{13}C NMR (176 MHz, $CDCl_3$) δ 209.9, 108.1, 72.1, 72.1, 69.7, 39.3, 34.5, 26.3, 24.0.
$[\alpha]_D$: +19.8 (c = 0.87, T = 25 °C, $CHCl_3$)
HRMS (ESI+) $[M+H]^+$ m/z calc'd for $[C_9H_{14}O_4H]^+$ expect 187.0970; found 187.0966

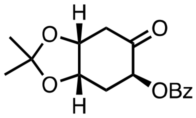

Derivatization for enantiomeric excess (ee) determination (**Bz-2o**)
Bz-2o was prepared via General Procedure **C1**. Purified by preparative thin layer chromatography (SiO_2, 10% EtOAc/CH_2Cl_2) afforded an analytical sample of **Bz-2o** for ee determination. Chiral SFC analysis indicated that **2o** was formed in 84% ee. A sample of racemic **Bz-2o** was synthesized *via* General Procedure **D1**.

^{1}H NMR (400 MHz, $CDCl_3$) δ 8.17 – 7.96 (m, 2H), 7.65 – 7.55 (m, 1H), 7.47 (t, *J* = 7.8 Hz, 2H), 5.59 (dd, *J* = 13.3, 4.9 Hz, 1H), 4.69 (s, 2H), 3.02 – 2.86 (m, 1H), 2.79 (dd, *J* = 18.8, 2.5 Hz, 1H), 2.44 (dd, *J* = 13.9, 4.9 Hz, 1H), 2.30 (td, *J* = 13.9, 2.4 Hz, 1H), 1.60 (s, 3H), 1.42 (s, 3H).
^{13}C NMR (101 MHz, $CDCl_3$) δ 202.9, 165.5, 133.3, 129.9, 129.5, 128.4, 108.4, 72.1, 72.1, 71.4, 40.8, 32.2, 26.4, 24.0.
HRMS (ESI+) $[M+H]^+$ m/z calc'd for $[C_{16}H_{18}O_5H]^+$ expect 291.1232; found 291.1228.

Chiral SFC Analysis: CHIRALPAK IG (CO_2: MeOH, 88:12, 2.5 mL min^{-1}, 40 °C, 227 nm) t_R = 7.54 (major), 9.32 (minor) minutes. 84% ee

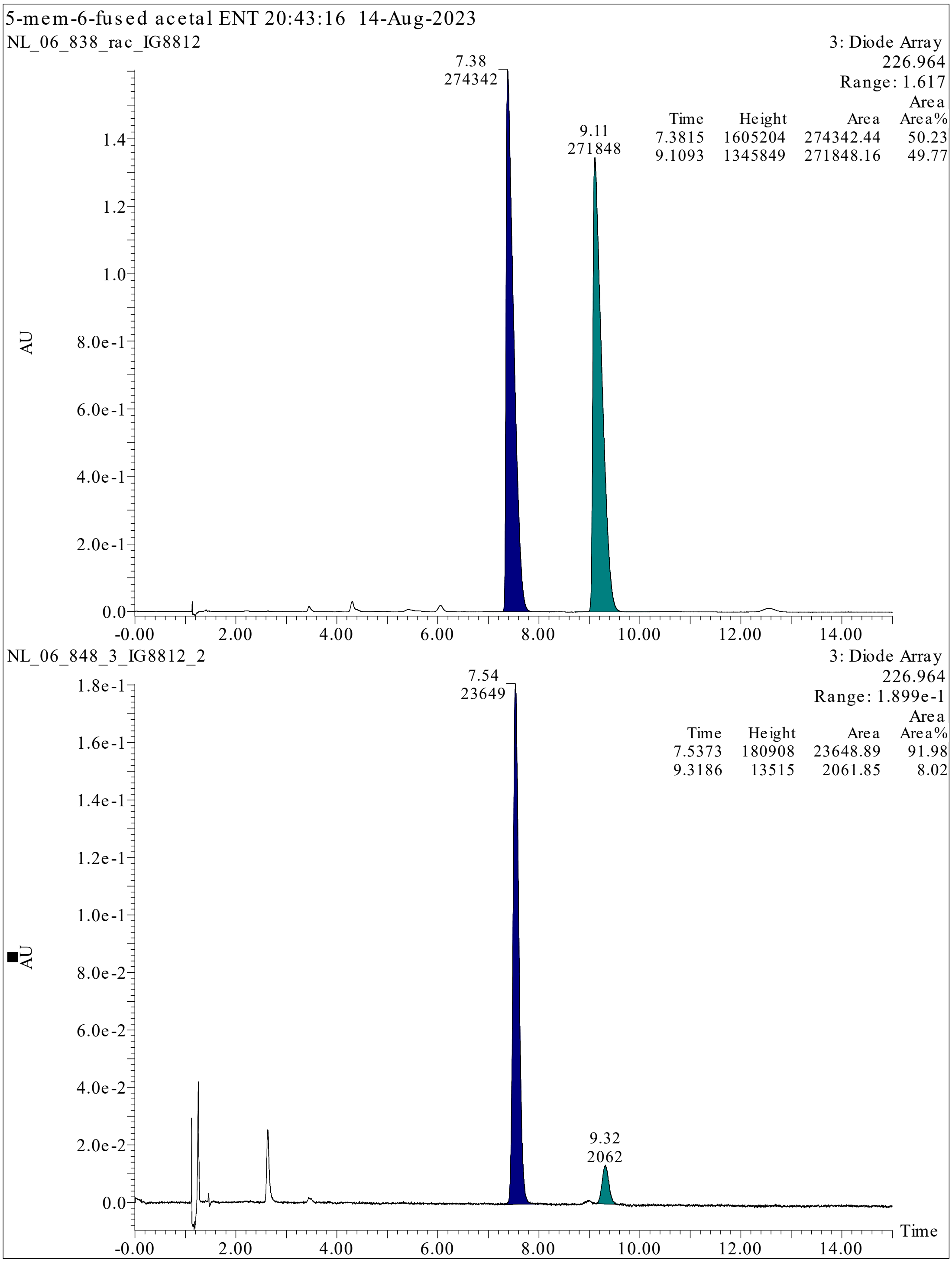

dimethyl (3a*S*,5*S*,7a*R*)-5-hydroxy-6-oxooctahydro-2*H*-indene-2,2-dicarboxylate (2p)

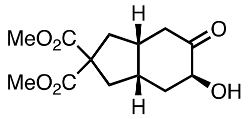

Dimethyl (3a*R*,5*R*,6*S*,7a*S*)-5,6-dihydroxyoctahydro-2*H*-indene-2,2-dicarboxylate **1p** (27.2 mg, 0.10 mmol) was subjected to General Procedure **B**, with 4CzIPN (3.9 mg, 0.005 mmol), *epi*-NHBoc-DHCN (4.0 mg, 0.01 mmol), TBA·H_2PO_4 (8.5 mg, 0.025 mmol), DIAD (20 mg, 0.10 mmol) in MeCN (4 mL) and reacted for 36 h. Purification by column chromatography (SiO_2, CH_2Cl_2 then 20% EtOAc/hexanes) afforded **2p** as a clear oil (20.3 mg, 0.075 mmol, 75%).

^{1}H NMR (700 MHz, $CDCl_3$) δ 4.35 (dd, *J* = 11.8, 7.0 Hz, 1H), 3.78 (s, 3H), 3.78 (s, 3H), 3.41 (s, 1H), 2.64 – 2.40 (m, 8H), 2.23 (d, *J* = 13.4 Hz, 1H), 1.82 (td, *J* = 13.8, 4.4 Hz, 1H).
^{13}C NMR (176 MHz, $CDCl_3$) δ 211.0, 173.2, 172.7, 70.8, 59.2, 53.1, 53.0, 42.1, 41.2, 40.4, 38.3, 36.9, 35.8.
$[\alpha]_D$: –7.7 (c = 0.67, T = 25 °C, $CHCl_3$)
HRMS (ESI+) $[M+Na]^+$ m/z calc'd for $[C_{13}H_{18}O_6Na]^+$ expect 293.1001; found 293.0997

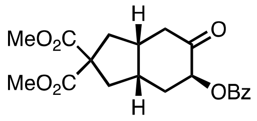

Derivatization for enantiomeric excess (ee) determination (**Bz-2p**)
Bz-2p was prepared via General Procedure **C1**. Purified by preparative thin layer chromatography (SiO_2, 20% EtOAc/CH_2Cl_2) afforded an analytical sample of **Bz-2p** for ee determination. Chiral SFC analysis indicated that **2p** was formed in 89% ee. A sample of racemic **Bz-2p** was synthesized *via* General Procedure **D1**.

^{1}H NMR (700 MHz, $CDCl_3$) δ 8.11 – 8.04 (m, 2H), 7.61 – 7.53 (m, 1H), 7.47 – 7.40 (m, 2H), 5.51 (dd, *J* = 11.4, 6.2 Hz, 1H), 3.78 (s, 5H), 3.76 (s, 4H), 2.67 – 2.43 (m, 7H), 2.40 (ddd, *J* = 13.7, 6.2, 2.8 Hz, 1H), 2.31 – 2.19 (m, 2H).
^{13}C NMR (176 MHz, $CDCl_3$) δ 204.3, 172.8, 172.8, 165.6, 133.3, 129.9, 129.5, 128.4, 73.3, 59.1, 53.1, 53.0, 42.0, 41.8, 40.0, 37.7, 37.1, 32.6.
HRMS (ESI+) $[M+Na]^+$ m/z calc'd for $[C_{20}H_{22}O_7Na]^+$ expect 397.1263; found 397.1259

Chiral SFC Analysis: CHIRALPAK IC (CO_2: MeOH, 85:15, 2.5 mL min^{-1}, 40 °C, 227 nm) t_R = 5.16 (major), 5.72 (minor) minutes. 89% ee

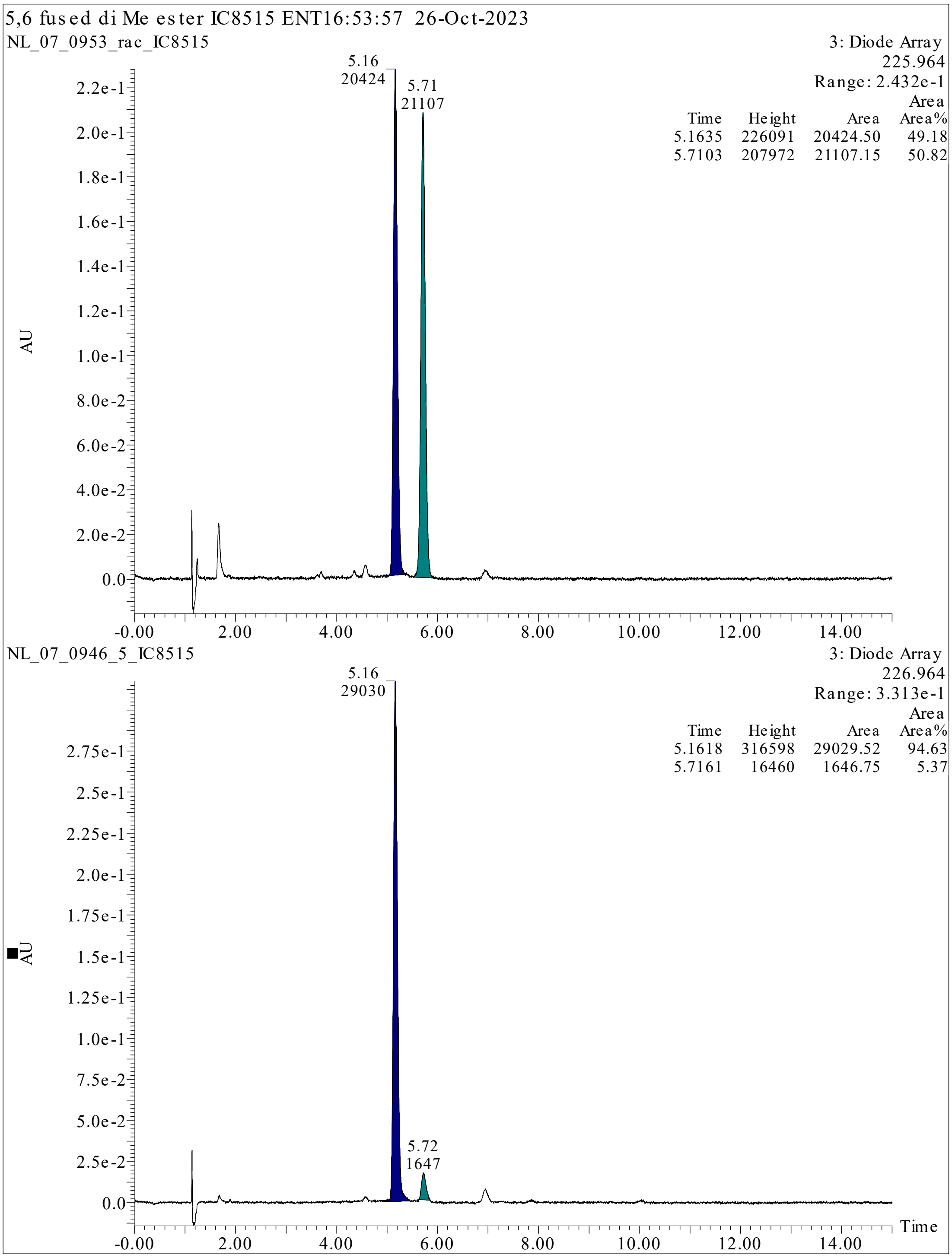

Ethyl (2*R*,3a*S*,5*S*,7a*R*)-2-cyano-5-hydroxy-6-oxooctahydro-1*H*-indene-2-carboxylate (2q)

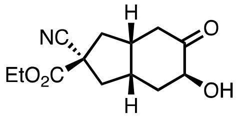

Ethyl (2*r*,3a*R*,5*R*,6*S*,7a*S*)-2-cyano-5,6-dihydroxyoctahydro-1*H*-indene-2-carboxylate **1q** (25.3 mg, 0.10 mmol) was subjected to General Procedure **B**, with 4CzIPN (3.9 mg, 0.005 mmol), *epi*-NHBoc-DHCN (4.0 mg, 0.01 mmol), TBA·H_2PO_4 (8.5 mg, 0.025 mmol), DIAD (20 mg, 0.10 mmol) in MeCN (4 mL) and reacted for 36 h. Purification by column chromatography (SiO_2, CH_2Cl_2 then 20–30% EtOAc/hexanes) afforded **2q** as a clear oil (14.0 mg, 0.056 mmol, 56%).

^{1}H NMR (700 MHz, $CDCl_3$) δ 4.38 – 4.32 (m, 1H), 4.29 (q, *J* = 7.1 Hz, 2H), 3.37 (d, *J* = 3.2 Hz, 1H), 2.69 (dd, *J* = 12.2, 6.3 Hz, 1H), 2.67–2.61 (m, 3H), 2.59–2.53 (m, 3H), 2.53–2.47 (m, 1H), 2.27 (d, *J* = 12.7 Hz, 1H), 1.89 – 1.82 (m, 1H), 1.35 (t, *J* = 7.1 Hz, 3H).
^{13}C NMR (176 MHz, $CDCl_3$) δ 209.8, 169.5, 121.6, 70.6, 63.4, 45.0, 43.9, 42.7, 40.8, 39.9, 38.4, 35.3, 14.0.
$[\alpha]_D$: +6.1 (c = 0.87, T = 25 °C, $CHCl_3$)
HRMS (ESI+) $[M+H]^+$ m/z calc'd for $[C_{13}H_{17}NO_4H]^+$ expect 252.1236; found 252.1229

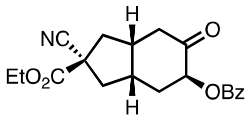

Derivatization for enantiomeric excess (ee) determination (**Bz-2q**)
Bz-2p was prepared via General Procedure **C1**. Purified by preparative thin layer chromatography (SiO_2, 20% EtOAc/CH_2Cl_2) afforded an analytical sample of **Bz-2q** for ee determination. Chiral SFC analysis indicated that **2p** was formed in 78% ee. A sample of racemic **Bz-2q** was synthesized *via* General Procedure **D1**.

^{1}H NMR (700 MHz, $CDCl_3$) δ 8.10 (d, *J* = 8.0 Hz, 2H), 7.61 (t, *J* = 7.4 Hz, 1H), 7.48 (t, *J* = 7.6 Hz, 2H), 5.53 (dd, *J* = 10.8, 6.0 Hz, 1H), 4.33 (q, *J* = 7.1 Hz, 2H), 2.79 – 2.59 (m, 7H), 2.46 (dt, *J* = 14.3, 4.9 Hz, 1H), 2.39 – 2.32 (m, 1H), 2.30 (dd, *J* = 14.2, 2.3 Hz, 1H), 1.38 (t, *J* = 7.1 Hz, 3H).
^{13}C NMR (176 MHz, $CDCl_3$) δ 203.3, 169.6, 165.5, 133.4, 129.9, 129.3, 128.5, 121.3, 72.9, 63.4, 44.9, 43.3, 42.2, 41.4, 40.3, 37.7, 32.3, 29.7, 14.0.
HRMS (ESI+) $[M+H]^+$ m/z calc'd for $[C_{20}H_{21}NO_5H]^+$ expect 356.1498; found 356.1497

Chiral SFC Analysis: CHIRALPAK IC (CO_2: MeOH, 78:22, 2.5 mL min^{-1}, 40 °C, 227 nm) t_R = 4.84 (minor), 10.81 (major) minutes. 78% ee

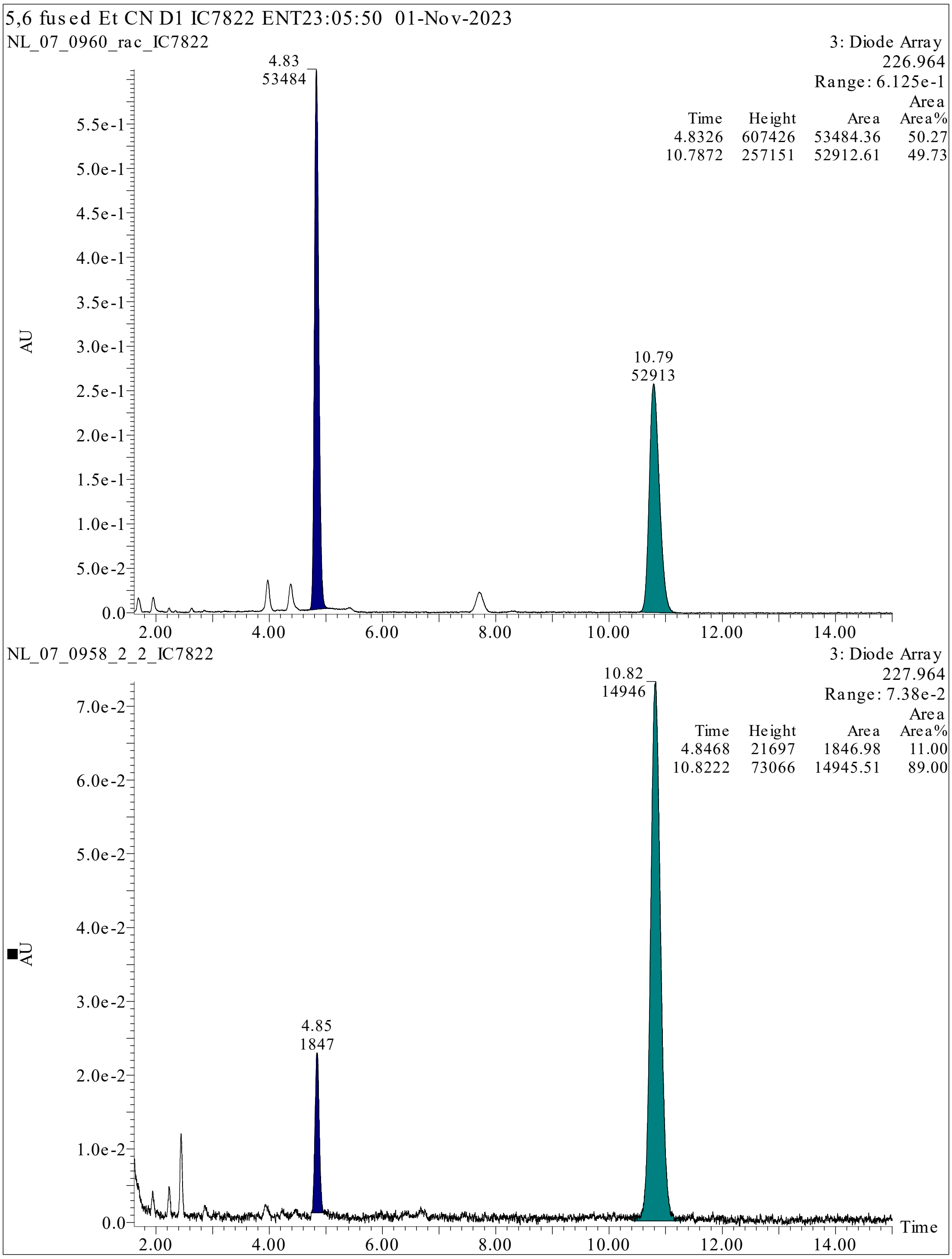

Ethyl (2*S*,3a*S*,5*S*,7a*R*)-2-cyano-5-hydroxy-6-oxooctahydro-1*H*-indene-2-carboxylate (2r)

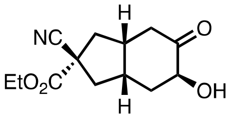

Ethyl (2*s*,3a*R*,5*R*,6*S*,7a*S*)-2-cyano-5,6-dihydroxyoctahydro-1*H*-indene-2-carboxylate **1r** (25.3 mg, 0.10 mmol) was subjected to General Procedure **B**, with 4CzIPN (3.9 mg, 0.005 mmol), *epi*-NHBoc-DHCN (4.0 mg, 0.01 mmol), TBA·H_2PO_4 (8.5 mg, 0.025 mmol), DIAD (20 mg, 0.10 mmol) in MeCN (4 mL) and reacted for 36 h. Purification by column chromatography (SiO_2, CH_2Cl_2 then 20–30% EtOAc/hexanes) afforded **2r** as a clear oil (9.6 mg, 0.038 mmol, 38%).

^{1}H NMR (400 MHz, $CDCl_3$) δ 4.33 – 4.25 (m, 3H), 3.38 (s, 1H), 2.69 (dt, *J* = 15.4, 8.5 Hz, 2H), 2.66 – 2.51 (m, 5H), 2.48 (ddd, *J* = 14.1, 6.8, 2.2 Hz, 1H), 2.21 (dd, *J* = 14.1, 2.2 Hz, 1H), 1.89 (ddd, *J* = 14.0, 11.8, 5.0 Hz, 1H), 1.36 (t, *J* = 7.1 Hz, 3H).
^{13}C NMR (101 MHz, $CDCl_3$) δ 210.1, 169.7, 120.8, 70.7, 63.5, 46.9, 43.8, 41.1, 40.8, 40.5, 38.3, 35.0, 14.0.
$[\alpha]_D$: –3.9 (c = 0.43, T = 25 °C, $CHCl_3$)
HRMS (ESI+) $[M+H]^+$ m/z calc'd for $[C_{13}H_{17}NO_4H]^+$ expect 252.1236; found 252.1228

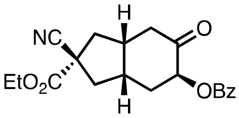

Derivatization for enantiomeric excess (ee) determination (**Bz-2r**)
Bz-2r was prepared via General Procedure **C1**. Purified by preparative thin layer chromatography (SiO_2, 20% EtOAc/CH_2Cl_2) afforded an analytical sample of **Bz-2r** for ee determination. Chiral SFC analysis indicated that **2r** was formed in 89% ee. A sample of racemic **Bz-2r** was synthesized *via* General Procedure **D1**.

^{1}H NMR (500 MHz, $CDCl_3$) δ 8.10 (dd, *J* = 8.3, 1.3 Hz, 2H), 7.66 – 7.54 (m, 1H), 7.48 (t, *J* = 7.8 Hz, 2H), 5.50 (dd, *J* = 11.0, 6.0 Hz, 1H), 4.35 (q, *J* = 7.1 Hz, 2H), 2.87 (td, *J* = 10.9, 5.5 Hz, 1H), 2.80 (tt, *J* = 11.4, 5.4 Hz, 1H), 2.74 – 2.57 (m, 5H), 2.46 (ddd, *J* = 14.0, 6.0, 4.0 Hz, 1H), 2.37 (ddd, *J* = 14.0, 11.0, 5.4 Hz, 1H), 2.26 (dd, *J* = 14.3, 4.2 Hz, 1H), 1.40 (t, *J* = 7.1 Hz, 3H).
^{13}C NMR (126 MHz, $CDCl_3$) δ 203.5, 169.4, 165.6, 133.4, 129.9, 129.3, 128.4, 120.9, 72.9, 63.5, 46.9, 43.3, 41.6, 40.8, 40.6, 37.5, 32.1, 14.0.
HRMS (ESI+) $[M+H]^+$ m/z calc'd for $[C_{20}H_{21}NO_5H]^+$ expect 356.1498; found 356.1494

Chiral SFC Analysis: CHIRALPAK IC (CO_2: MeOH, 78:22, 2.5 mL min^{-1}, 40 °C, 233 nm) t_R = 3.97 (major), 4.39 (minor) minutes. 89% ee

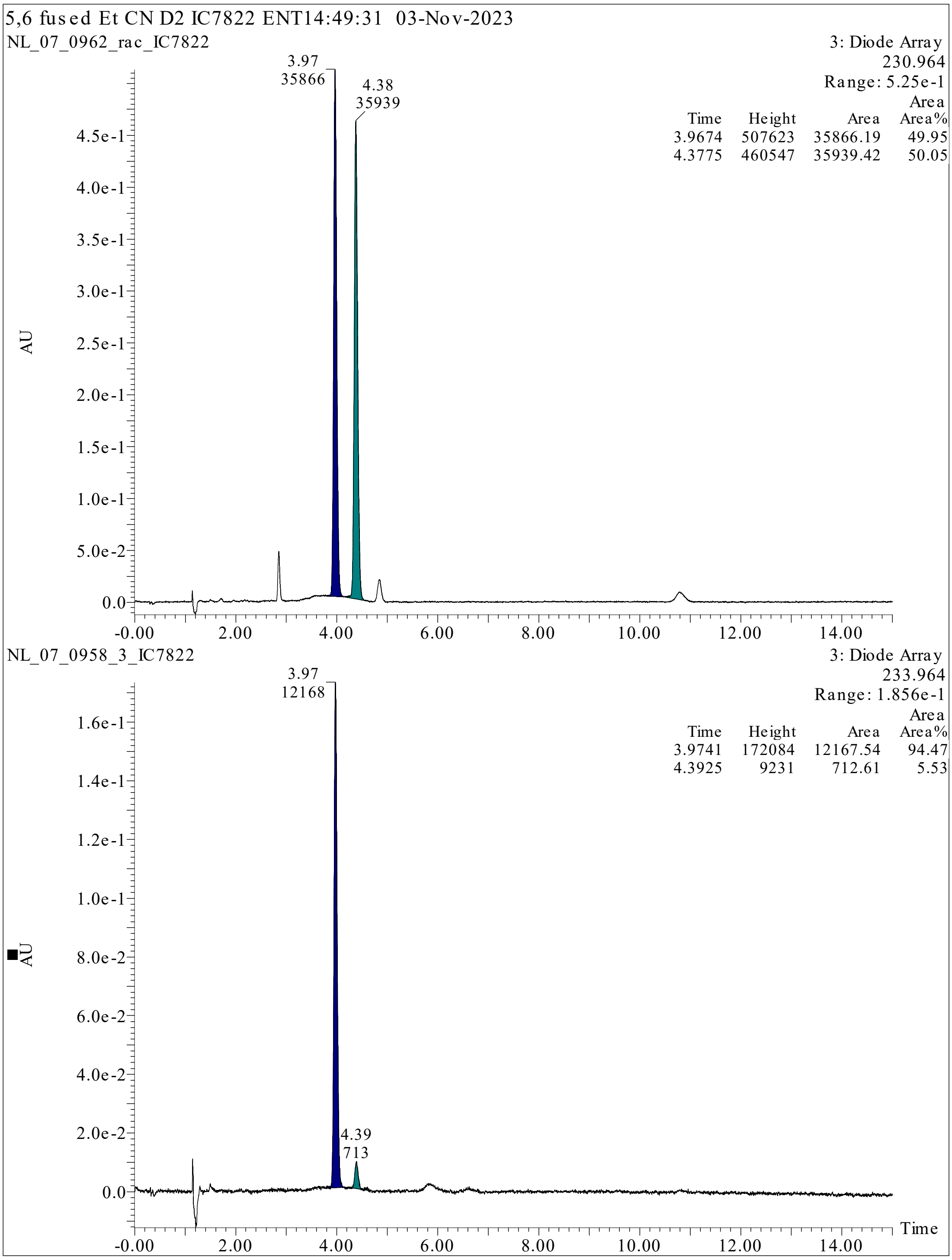

Ethyl (3a*R*,5*S*,7a*S*)-2-cyano-5-hydroxy-6-oxooctahydro-1*H*-indene-2-carboxylate (2s)

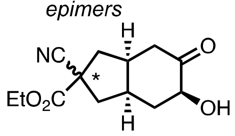

Ethyl (3a*R*,5*S*,6*R*,7a*S*)-2-cyano-5,6-dihydroxyoctahydro-1*H*-indene-2-carboxylate **1s** (25.3 mg, 0.10 mmol, 1.3:1 mixture of epimers at marked stereocenter) was subjected to General Procedure **B**, with 4CzIPN (3.9 mg, 0.005 mmol), *epi*-NHBoc-DHCN (4.0 mg, 0.01 mmol), TBA·H_2PO_4 (8.5 mg, 0.025 mmol), DIAD (20 mg, 0.10 mmol) in MeCN (4 mL) and reacted for 36 h. Purification by column chromatography (SiO_2, CH_2Cl_2 then 15% EtOAc/hexanes) afforded **2s** as a clear oil (15.4 mg, 0.61 mmol, 61%) as an inseparable epimeric mixture at the marked (*) stereocenter.

^{1}H NMR (700 MHz, $CDCl_3$) δ 4.27 (q, *J* = 7.1 Hz, 2H), 4.22 – 4.15 (m, 1H), 3.49* (d, *J* = 3.5 Hz, 0.6H), 3.45' (d, *J* = 3.5 Hz, 0.4H), 2.94–2.89' (m, 0.4H), 2.83–2.68 (m, 2.6H), 2.62* (dd, *J* = 13.9, 6.8 Hz, 1H), 2.58 – 2.50 (m, 2H), 2.41 (td, *J* = 13.6, 6.4 Hz, 1H), 2.31–2.22 (m, 2H), 2.17 – 2.07 (m, 1.6H), 1.80–1.64 (m, 1.4H), 1.36–1.31 (m, 3H). Reported as a combined mixture of epimers. Discernible signals arising from the major epimer (<u>1.3</u>:1 ratio, assigned by relative integration) denoted by (*); minor epimer denoted by (')
^{13}C NMR (176 MHz, $CDCl_3$) δ 210.2*, 210.1, 169.7, 168.9*, 121.1, 120.9*, 73.2, 73.1*, 63.4*, 63.3, 46.7*, 44.5, 43.2, 42.6*, 41.3*, 41.3, 40.7, 40.7*, 38.8, 38.8*, 37.7, 37.4, 37.1*, 36.4*, 14.0'. Discernible signals arising from the minor epimer (1.3:<u>1</u> ratio, assigned by relative peak intensity observed by ^{13}C NMR) denoted by (*). ^{13}C signal from both epimers denoted by (')
[α]$_D$: +6.6 (c = 1.07, T = 25 °C, $CHCl_3$)
HRMS (ESI+) $[M+H]^+$ m/z calc'd for $[C_{13}H_{17}NO_4H]^+$ expect 252.1236; found 252.1226

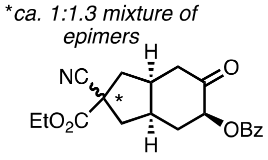

Derivatization for enantiomeric excess (ee) determination (**Bz-2s**)

Bz-2s was prepared via General Procedure **C1**. Purified by preparative thin layer chromatography (SiO_2, 20% EtOAc/CH_2Cl_2) afforded an analytical sample of **Bz-2s** for ee determination as an inseparable epimeric mixture at the marked (*) stereocenter. Chiral SFC analysis indicated that **2s** was formed in 88% ee and 93% ee for each respective diastereomer. A sample of racemic **Bz-2s** was synthesized *via* General Procedure **D1**.

^{1}H NMR (700 MHz, $CDCl_3$) δ 8.17–8.09 (m, 2H), 7.70 – 7.55 (m, 1H), 7.51–7.48 (m, 2H), 5.48 + 5.45 (dd, *J* = 13.2, 5.8 Hz, 1H), 4.38 – 4.29 (m, 2H), 3.02 – 2.82 (m, 3H), 2.74 – 2.67 (m, 1H), 2.64 – 2.47 (m, 2H), 2.47 – 2.26 (m, 2H), 2.26 – 2.05 (m, 2H), 1.37 + 1.37 (t, *J* = 7.2, 3H). Reported as a combined mixture of epimers

^{13}C NMR (176 MHz, $CDCl_3$) δ 203.1*, 202.9, 169.6, 169.2*, 165.5, 165.5*, 133.4, 133.4*, 130.0, 129.9*, 129.3*, 129.2, 128.5*, 128.4, 121.3, 120.9*, 74.5*, 74.5, 63.5*, 63.4, 46.7*, 44.6, 43.2, 42.5*, 41.5*, 41.2, 40.8*, 40.7, 40.0, 40.0*, 38.3, 37.0*, 33.9, 33.7*, 14.0*, 14.0. Discernible signals arising from the minor epimer (1.3:1 ratio, assigned by relative peak intensity observed by ^{13}C NMR) denoted by (*).

HRMS (ESI+) $[M+Na]^+$ m/z calc'd for $[C_{20}H_{21}NO_5Na]^+$ expect 378.1317; found 378.1317

Chiral SFC Analysis: CHIRALPAK IC (CO_2: MeOH, 78:22, 2.5 mL min^{-1}, 40 °C, 233 nm)

Diastereomeric ratio: 1.3:1 (major:minor) as indicated by NMR and relative integration in the SFC trace for racemic **2s** (top). Retention times arising from each racemic pair of diastereomers deduced by their relative integrations from racemic **2s**.

Diastereomer 1: t_R = 3.95 (minor), 4.47 (major) minutes. 88% ee (middle)

Diastereomer 2: t_R = 4.24 (minor), 6.68 (major) minutes. 93% ee (bottom)

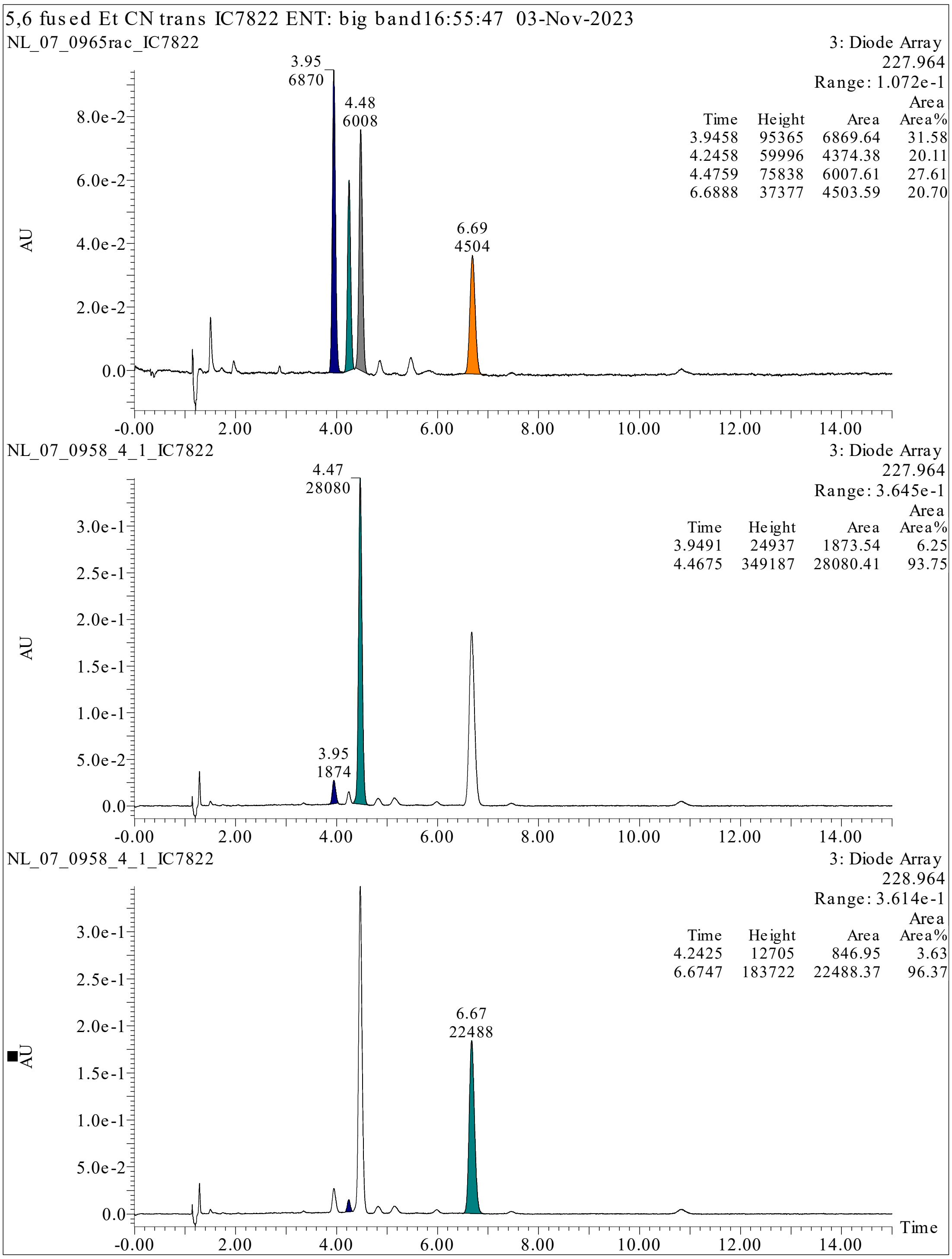

5,6 fused Et CN trans IC7822 ENT: big band16:55:47 03-Nov-2023
NL_07_0965rac_IC7822
3: Diode Array
227.964
Range: 1.072e-1
Time Height Area Area%
3.9458 95365 6869.64 31.58
4.2458 59996 4374.38 20.11
4.4759 75838 6007.61 27.61
6.6888 37377 4503.59 20.70
3.95 6870
4.48 6008
6.69 4504
AU
NL_07_0958_4_1_IC7822
3: Diode Array
227.964
Range: 3.645e-1
Time Height Area Area%
3.9491 24937 1873.54 6.25
4.4675 349187 28080.41 93.75
4.47 28080
3.95 1874
NL_07_0958_4_1_IC7822
3: Diode Array
228.964
Range: 3.614e-1
Time Height Area Area%
4.2425 12705 846.95 3.63
6.6747 183722 22488.37 96.37
6.67 22488
Time

(5a*R*,8*S*,9a*S*)-8-hydroxy-3,3-dimethylhexahydrobenzo[*e*][1,3]dioxepin-7(5*H*)-one (2t)

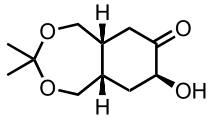

(5a*R*,7*R*,8*S*,9a*S*)-3,3-dimethyloctahydrobenzo[*e*][1,3]dioxepine-7,8-diol **1t** (21.6 mg, 0.10 mmol) was subjected to General Procedure **B**, with 4CzIPN (3.9 mg, 0.005 mmol), *epi*-NHBoc-DHCN (4.0 mg, 0.01 mmol), TBA·H_2PO_4 (8.5 mg, 0.025 mmol), DIAD (20 mg, 0.10 mmol) in MeCN (4 mL) and reacted for 36 h. Purification by column chromatography (SiO_2, CH_2Cl_2 then 15–50% EtOAc/CH_2Cl_2) afforded **2t** as a clear oil (16.0 mg, 0.075 mmol, 75%).

^{1}H NMR (700 MHz, $CDCl_3$) δ 4.10 (m, 2H), 4.00 (dd, *J* = 12.5, 1.4 Hz, 1H), 3.56 (ddd, *J* = 12.2, 3.2, 1.1 Hz, 1H), 3.53 (d, *J* = 3.6 Hz, 1H), 3.36 (dd, *J* = 12.5, 2.6 Hz, 1H), 3.01 – 2.94 (m, 1H), 2.39 (ddd, *J* = 14.4, 4.4, 1.3 Hz, 1H), 2.29 (ddd, *J* = 13.4, 6.8, 2.3 Hz, 1H), 2.22 (d, *J* = 4.5 Hz, 1H), 2.17 – 2.08 (m, 1H), 1.73 (td, *J* = 13.2, 5.4 Hz, 1H), 1.37 (s, 3H), 1.36 (s, 3H).
^{13}C NMR (176 MHz, $CDCl_3$) δ 211.4, 102.0, 71.4, 63.7, 62.4, 41.8, 39.7, 38.4, 37.4, 24.9, 24.8.
$[\alpha]_D$: +20.2 (c = 1.0, T = 25 °C, $CHCl_3$)
HRMS (ESI+) $[M+H]^+$ m/z calc'd for $[C_{11}H_{18}O_4H]^+$ expect 215.1282; found 215.1283

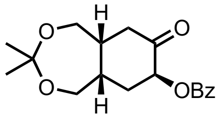

Derivatization for enantiomeric excess (ee) determination (**Bz-2t**)
Bz-2t was prepared via General Procedure **C1**. Purified by preparative thin layer chromatography (SiO_2, 20% EtOAc/CH_2Cl_2) afforded an analytical sample of **Bz-2t** for ee determination. Chiral SFC analysis indicated that **2t** was formed in 84% ee. A sample of racemic **Bz-2t** was synthesized *via* General Procedure **D1**.

^{1}H NMR (700 MHz, $CDCl_3$) δ 8.08 (d, *J* = 8.0 Hz, 2H), 7.61–7.53 (m, 1H), 7.45 (t, *J* = 8.0 Hz, 2H), 5.36 (dd, *J* = 12.9, 6.2 Hz, 1H), 4.20 (t, *J* = 11.4 Hz, 1H), 3.99 (d, *J* = 12.7 Hz, 1H), 3.63 (dd, *J* = 12.1, 2.6 Hz, 1H), 3.40 (dd, *J* = 12.4, 3.0 Hz, 1H), 3.03 (t, *J* = 14.0 Hz, 1H), 2.51–2.39 (m, 1H), 2.35 (s, 1H), 2.29 (ddd, *J* = 13.4, 6.2, 2.8 Hz, 1H), 2.24 – 2.14 (m, 2H), 1.40 (s, 3H), 1.38 (s, 3H).
^{13}C NMR (176 MHz, $CDCl_3$) δ 204.4, 165.6, 133.3, 129.9, 129.5, 128.4, 102.0, 73.6, 63.6, 62.6, 41.5, 39.7, 39.2, 33.7, 24.9, 24.8.
HRMS (ESI+) $[M+Na]^+$ m/z calc'd for $[C_{18}H_{22}O_5Na]^+$ expect 341.1363; found 341.1362

Chiral SFC Analysis: CHIRALPAK IG (CO_2: MeOH, 88:12, 2.5 mL min^{-1}, 40 °C, 226 nm) t_R = 7.52 (major), 9.30 (minor) minutes. 84% ee

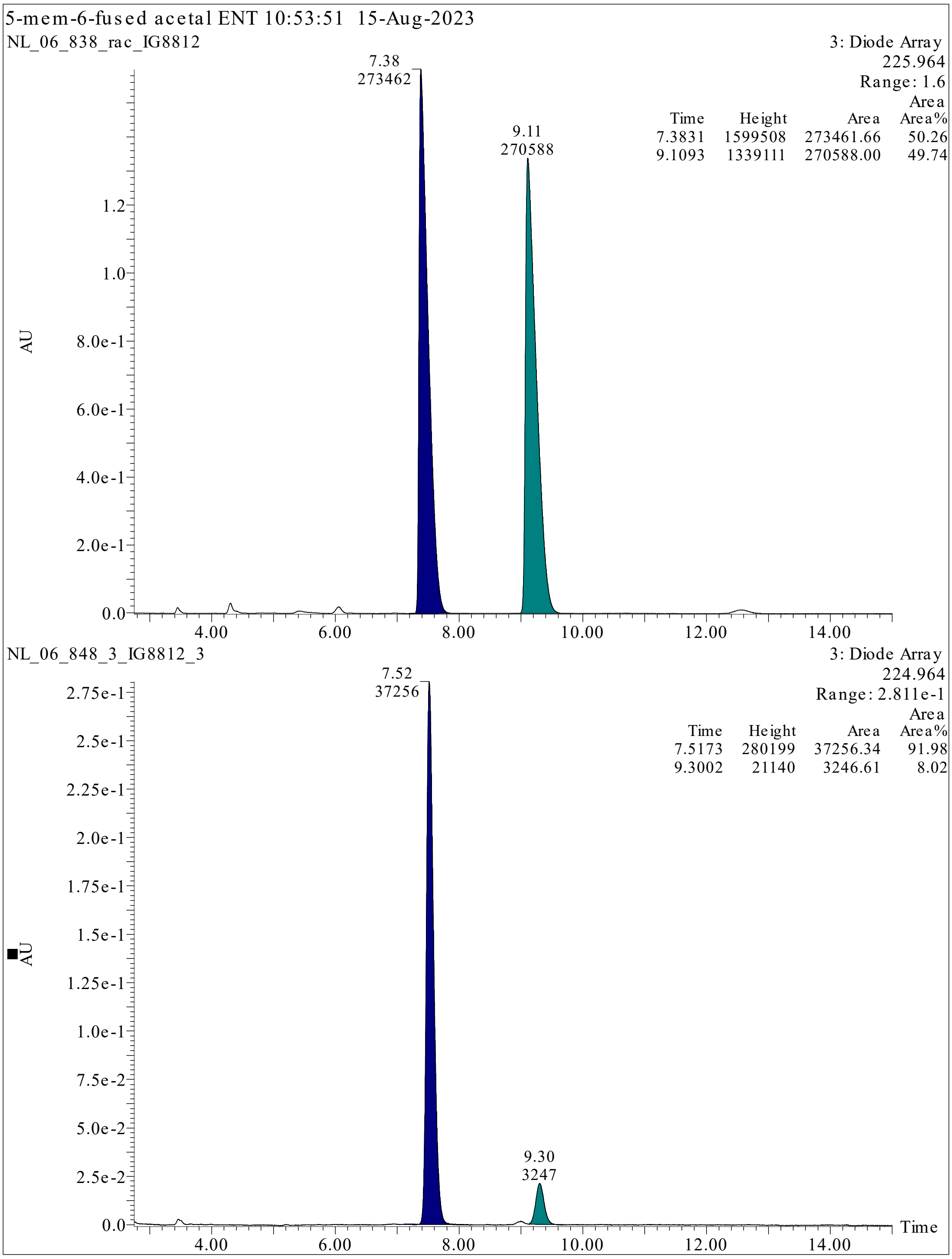

(5a*S*,8*S*,9a*R*)-8-hydroxy-3,3-dimethylhexahydrobenzo[*e*][1,3]dioxepin-7(5*H*)-one (2u)

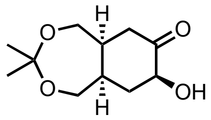

(5a*R*,7*S*,8*R*,9a*S*)-3,3-dimethyloctahydrobenzo[*e*][1,3]dioxepine-7,8-diol **1u** (21.6 mg, 0.10 mmol) was subjected to General Procedure **B**, with 4CzIPN (3.9 mg, 0.005 mmol), *epi*-NHBoc-DHCN (4.0 mg, 0.01 mmol), TBA·H_2PO_4 (8.5 mg, 0.025 mmol), DIAD (20 mg, 0.10 mmol) in MeCN (4 mL) and reacted for 36 h. Purification by column chromatography (SiO_2, CH_2Cl_2 then 15–30% EtOAc/CH_2Cl_2) afforded **2u** as a clear oil (18.9 mg, 0.088 mmol, 88%).

^{1}H NMR (700 MHz, $CDCl_3$) δ 4.33 – 4.16 (m, 1H), 3.99 – 3.84 (m, 1H), 3.68 – 3.55 (m, 1H), 3.53 – 3.46 (m, 2H), 3.35 (dd, *J* = 12.4, 2.4 Hz, 1H), 2.68 (dd, *J* = 15.0, 7.1 Hz, 1H), 2.42 (s, 1H), 2.35 (dd, *J* = 14.5, 2.0 Hz, 1H), 2.28 – 2.19 (m, 2H), 2.03 (q, *J* = 12.3 Hz, 1H), 1.32 (s, 3H), 1.28 (s, 3H).
^{13}C NMR (176 MHz, $CDCl_3$) δ 210.5, 101.5, 74.1, 63.7, 62.5, 42.7, 40.2, 36.7, 34.9, 24.7.
[α]$_D$: +14.1 (c = 1.2, T = 25 °C, $CHCl_3$)
HRMS (ESI+) $[M+H]^+$ m/z calc'd for $[C_{11}H_{18}O_4H]^+$ expect 215.1283; found 215.1281

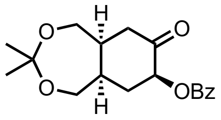

Derivatization for enantiomeric excess (ee) determination (**Bz-2u**)
Bz-2u was prepared via General Procedure **C1**. Purified by preparative thin layer chromatography (SiO_2, 20% EtOAc/CH_2Cl_2) afforded an analytical sample of **Bz-2u** for ee determination. Chiral SFC analysis indicated that **2u** was formed in 90% ee. A sample of racemic **Bz-2u** was synthesized *via* General Procedure **D1**.

^{1}H NMR (700 MHz, $CDCl_3$) δ 8.10 (dd, *J* = 8.3, 1.3 Hz, 2H), 7.61–7.54 (m, 1H), 7.49–7.42 (m, 2H), 5.51 (dd, *J* = 12.6, 6.9 Hz, 1H), 3.99 (d, *J* = 12.4 Hz, 1H), 3.80–3.65 (m, 1H), 3.55 (dd, *J* = 12.4, 2.9 Hz, 1H), 3.41 (dd, *J* = 12.5, 2.6 Hz, 1H), 2.78 (dd, *J* = 14.2, 7.2 Hz, 1H), 2.51–2.48 (m, 2H), 2.38–2.34 (m, 2H), 2.29–2.21 (m, 1H), 1.34 (s, 3H), 1.33 (s, 3H)
^{13}C NMR (176 MHz, $CDCl_3$) δ 203.6, 165.6, 133.3, 129.9, 129.5, 128.4, 101.6, 75.8, 63.5, 62.5, 42.5, 41.3, 37.4, 31.5, 24.8 (2C)
HRMS (ESI+) $[M+Na]^+$ m/z calc'd for $[C_{18}H_{22}O_5Na]^+$ expect 341.1365; found 341.1361

Chiral SFC Analysis: CHIRALPAK IG (CO_2: MeOH, 90:10, 2.5 mL min^{-1}, 40 °C, 230 nm) t_R = 5.60 (minor), 9.47 (major) minutes. 90% ee

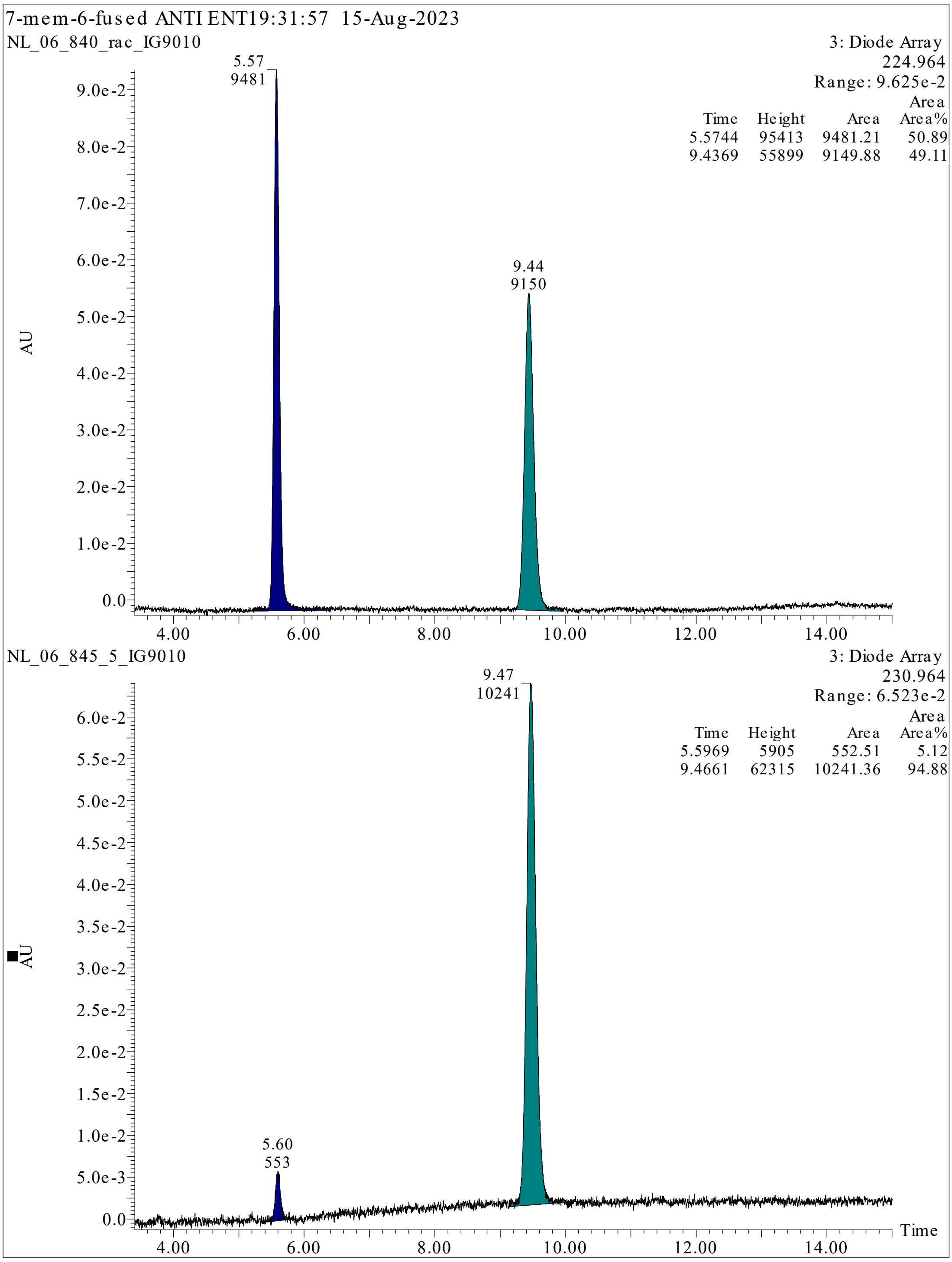

(1*R*,4*S*,6*S*)-4-hydroxybicyclo[4.2.0]octan-3-one (2v)

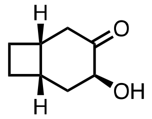

(1*R*,3*R*,4*S*,6*S*)-bicyclo[4.2.0]octane-3,4-diol **1v** (14.2 mg, 0.10 mmol) was subjected to General Procedure **B**, with 4CzIPN (3.9 mg, 0.005 mmol), *epi*-NHBoc-DHCN (4.0 mg, 0.01 mmol), TBA·H_2PO_4 (8.5 mg, 0.025 mmol), DIAD (20 mg, 0.10 mmol) in MeCN (4 mL) and reacted for 36 h. Purification by column chromatography (SiO_2, CH_2Cl_2) afforded **2v** as a clear oil (13.0 mg, 0.093 mmol, 93%).

^{1}H NMR (700 MHz, $CDCl_3$) δ 4.59 (dd, *J* = 12.6, 6.2 Hz, 1H), 2.96 – 2.84 (m, 1H), 2.83 – 2.74 (m, 1H), 2.67 (dd, *J* = 17.8, 7.3 Hz, 1H), 2.53 (dd, *J* = 17.8, 4.4 Hz, 1H), 2.37 – 2.28 (m, 2H), 2.30 – 2.23 (m, 1H), 2.12 – 2.05 (m, 1H), 1.83 (td, *J* = 13.3, 6.1 Hz, 1H), 1.73 – 1.66 (m, 1H).
^{13}C NMR (176 MHz, $CDCl_3$) δ 213.4, 72.0, 41.4, 36.3, 32.7, 31.4, 26.9, 23.6.
$[\alpha]_D$: +13.4 (c = 0.25, T = 25 °C, $CHCl_3$)
HRMS (ESI+) $[M+H]^+$ m/z calc'd for $[C_8H_{12}O_2H]^+$ expect 141.0916; found 141.0914

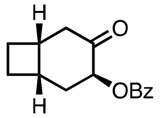

Derivatization for enantiomeric excess (ee) determination (**Bz-2v**)
Bz-2v was prepared via General Procedure **C1**. Purified by preparative thin layer chromatography (SiO_2, CH_2Cl_2) afforded an analytical sample of **Bz-2v** for ee determination. Chiral SFC analysis indicated that **2v** was formed in 87% ee. A sample of racemic **Bz-2v** was synthesized *via* General Procedure **D1**.

^{1}H NMR (700 MHz, $CDCl_3$) δ 8.17 – 8.02 (m, 2H), 7.67 – 7.55 (m, 1H), 7.48 (t, *J* = 7.8 Hz, 2H), 5.76 (dd, *J* = 11.4, 6.1 Hz, 1H), 3.01 – 2.81 (m, 2H), 2.74 (dd, *J* = 16.6, 7.1 Hz, 1H), 2.61 (dd, *J* = 16.6, 4.9 Hz, 1H), 2.41 – 2.21 (m, 4H), 2.22 – 2.08 (m, 1H), 1.89 – 1.74 (m, 1H).
^{13}C NMR (176 MHz, $CDCl_3$) δ 206.6, 165.8, 133.2, 129.9, 129.7, 128.4, 73.9, 42.6, 33.6, 33.3, 31.3, 26.5, 23.5.
HRMS (ESI+) $[M+H]^+$ m/z calc'd for $[C_{15}H_{16}O_3H]^+$ expect 245.1178; found 245.1177

Chiral SFC Analysis: CHIRALPAK IG (CO_2: MeOH, 87:13, 2.5 mL min^{-1}, 40 °C, 230 nm) t_R = 8.38 (major), 11.59 (minor) minutes. 87% ee

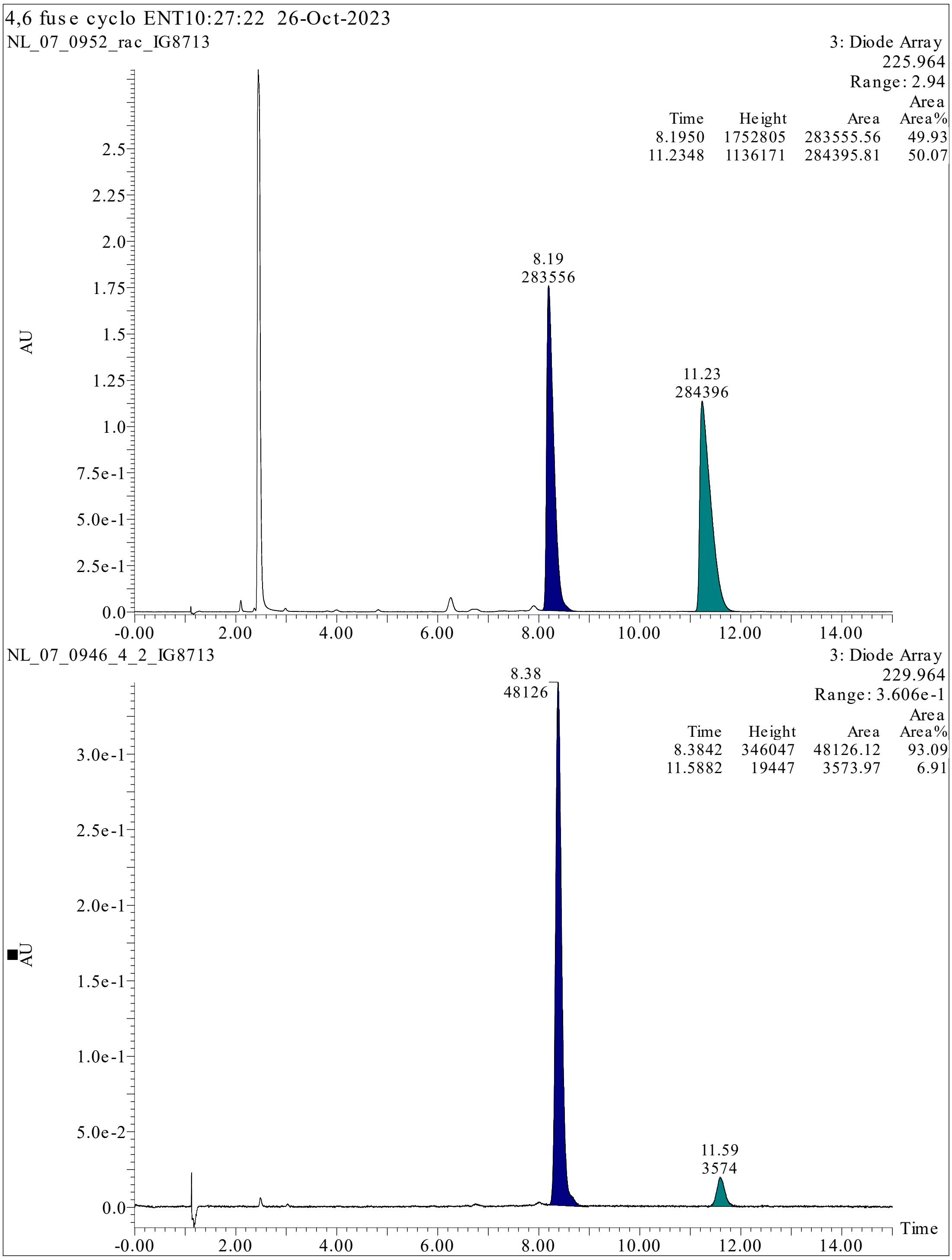

(4*S*,6*S*)-4,6-dihydroxycyclohex-2-en-1-one (2w')

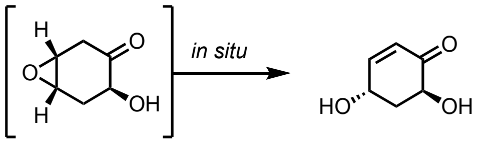

(1*R*,3*R*,4*S*,6*S*)-7-oxabicyclo[4.1.0]heptane-3,4-diol **1w** (13.0 mg, 0.10 mmol) was subjected to General Procedure **B**, with 4CzIPN (3.9 mg, 0.005 mmol), *epi*-NHBoc-DHCN (5.9 mg, 0.015 mmol), TBA·H_2PO_4 (8.5 mg, 0.025 mmol), DIAD (20 mg, 0.10 mmol) in MeCN (4 mL) and reacted for 36 h. Purification by column chromatography (SiO_2, CH_2Cl_2 then EtOAc/hexanes 20–40%) afforded **2w'** as a white solid (10.7 mg, 0.084 mmol, 84%).

Chiral SFC analysis indicated that **2w'** was formed in 94% ee. A racemic sample of **2w'** was synthesized using General Procedure **B**, using quinuclidine (15 mol%) as the HAA catalyst rather than *epi*-NHBoc-DHCN.

^{1}H NMR (700 MHz, $CDCl_3$) δ δ 7.01 (dt, *J* = 10.2, 2.0 Hz, 1H), 6.12 (dd, *J* = 10.2, 2.5 Hz, 1H), 4.72 (s, 1H), 4.15 (dd, *J* = 14.3, 4.5 Hz, 1H), 3.56 (s, 1H), 2.82 (dtd, *J* = 12.4, 5.2, 2.1 Hz, 1H), 1.90 (ddd, *J* = 13.5, 11.8, 10.6 Hz, 2H).
^{13}C NMR (176 MHz, $CDCl_3$) δ 199.4, 154.6, 126.0, 70.9, 66.7, 41.9.
$[\alpha]_D$: +40.4 (c = 0.68, T = 25 °C, $CHCl_3$)
HRMS (ESI+) $[M+Na]^+$ m/z calc'd for $[C_6H_8O_3Na]^+$ expect 151.0371; found 151.0367

Chiral SFC Analysis: CHIRALPAK IG (CO_2: MeOH, 75:25, 2.5 mL min^{-1}, 40 °C, 217 nm) t_R = 3.42 (major), 3.87 (minor) minutes. 94% ee

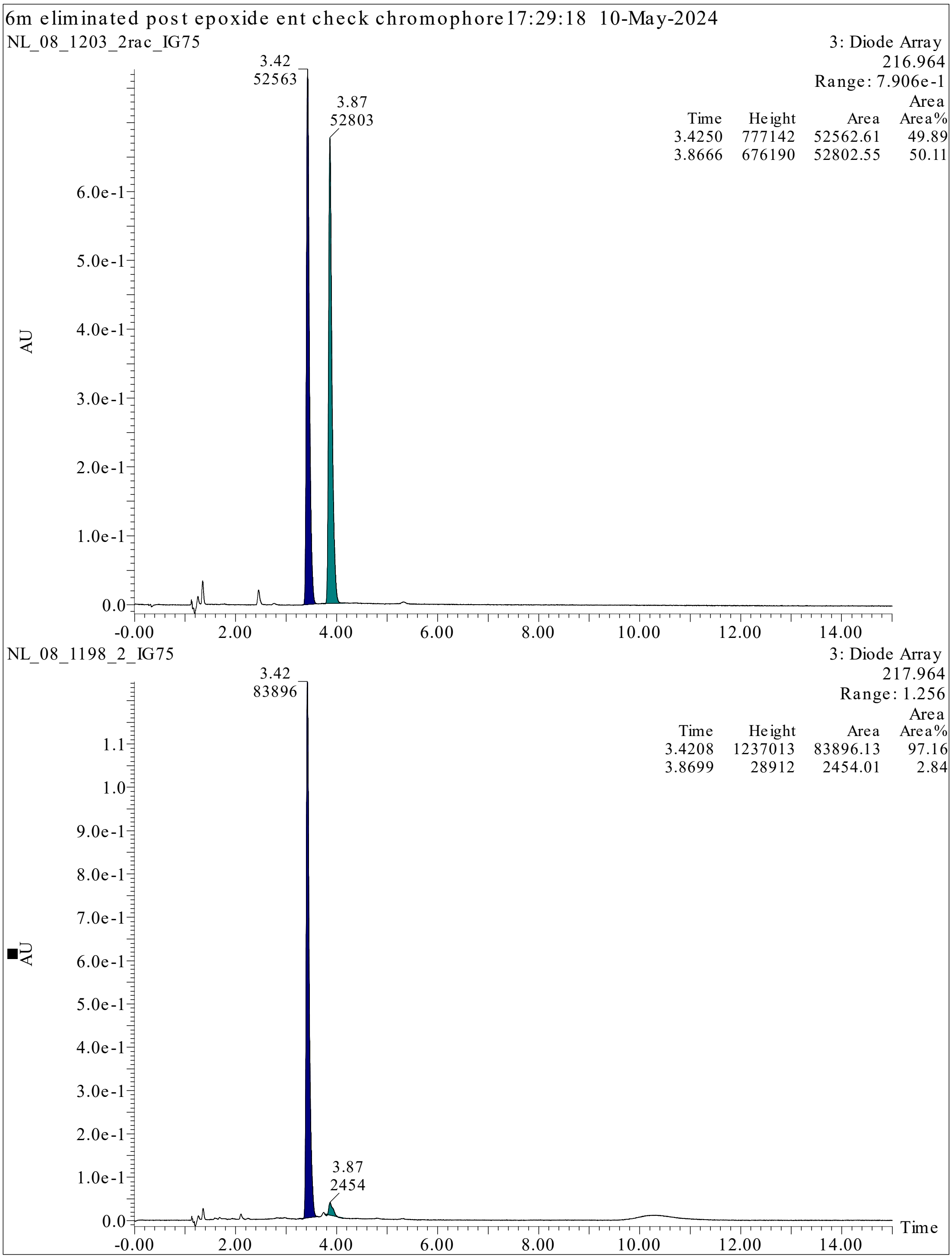

(1*R*,4*S*,6*S*,7*R*)-7-(((*tert*-butyldimethylsilyl)oxy)methyl)-7-chloro-4-hydroxybicyclo[4.1.0]heptan-3-one (2x)

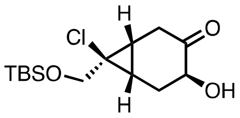

(1*R*,3*R*,4*S*,6*S*,7*R*)-7-(((*tert*-butyldimethylsilyl)oxy)methyl)-7-chlorobicyclo[4.1.0]heptane-3,4-diol **1x** (30.7 mg, 0.10 mmol) was subjected to General Procedure **B**, with 4CzIPN (3.9 mg, 0.005 mmol), *epi*-NHBoc-DHCN (4.0 mg, 0.010 mmol), TBA·H_2PO_4 (8.5 mg, 0.025 mmol), DIAD (20 mg, 0.10 mmol) in MeCN (4 mL) and reacted for 36 h. Purification by column chromatography (SiO_2, CH_2Cl_2/hexanes 50%) afforded **2x** as a clear oil (17.7 mg, 0.058 mmol, 58%).

^{1}H NMR (700 MHz, $CDCl_3$) δ 4.41 (td, *J* = 8.9, 3.6 Hz, 1H), 3.86 (s, 2H), 3.35 (d, *J* = 3.6 Hz, 1H), 2.92 (dd, *J* = 17.0, 9.3 Hz, 1H), 2.81 (dd, *J* = 14.4, 8.4 Hz, 1H), 2.61 (dd, *J* = 16.9, 1.9 Hz, 1H), 2.24 (ddd, *J* = 14.5, 9.2, 7.3 Hz, 1H), 1.62 (td, *J* = 9.8, 2.2 Hz, 1H), 1.49 – 1.44 (m, 1H), 0.90 (s, 9H), 0.09 (s, 6H).
^{13}C NMR (176 MHz, $CDCl_3$) δ 211.4, 70.5, 67.6, 53.7, 33.5, 32.4, 25.8, 18.6, 18.3, 16.7, -5.3.
[α]$_D$: –6.2 (c = 0.73, T = 25 °C, $CHCl_3$)
HRMS (ESI+) $[M+H]^+$ m/z calc'd for $[C_{14}H_{25}ClO_3SiH]^+$ expect 305.1340; found 305.1335

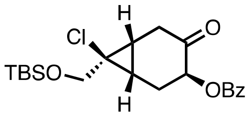

Derivatization for enantiomeric excess (ee) determination (**Bz-2x**)
Bz-2x was prepared via General Procedure **C2**. Purified by preparative thin layer chromatography (SiO_2, 20% EtOAc/hexanes) afforded an analytical sample of **Bz-2x** for ee determination. Chiral SFC analysis indicated that **2x** was formed in 72% ee. A sample of racemic **Bz-2x** was synthesized *via* General Procedure **D1**.

^{1}H NMR (700 MHz, $CDCl_3$) δ 8.15 – 8.01 (m, 2H), 7.61 (t, *J* = 7.4 Hz, 1H), 7.48 (t, *J* = 7.8 Hz, 2H), 5.39 (t, *J* = 6.5 Hz, 1H), 3.94 – 3.76 (m, 2H), 2.91 (dd, *J* = 16.9, 9.2 Hz, 1H), 2.78 – 2.64 (m, 2H), 2.60 (ddd, *J* = 15.1, 6.0, 2.9 Hz, 1H), 1.78 (td, *J* = 9.7, 2.2 Hz, 1H), 1.59 – 1.53 (m, 1H), 0.91 (s, 9H), 0.10 (s, 6H).
^{13}C NMR (176 MHz, $CDCl_3$) δ 205.1, 165.4, 133.3, 129.8, 129.6, 128.5, 73.1, 67.6, 54.2, 34.3, 28.6, 25.8, 19.4, 18.3, 15.1, –5.3.
HRMS (ESI+) $[M+H]^+$ m/z calc'd for $[C_{15}H_{16}O_3H]^+$ expect 245.1178; found 245.1177

Chiral SFC Analysis: CHIRALPAK IG (CO_2: *i*PrOH, 90:10, 2.5 mL min^{-1}, 40 °C, 226 nm) t_R = 4.01 (minor), 4.72 (major) minutes. 72% ee

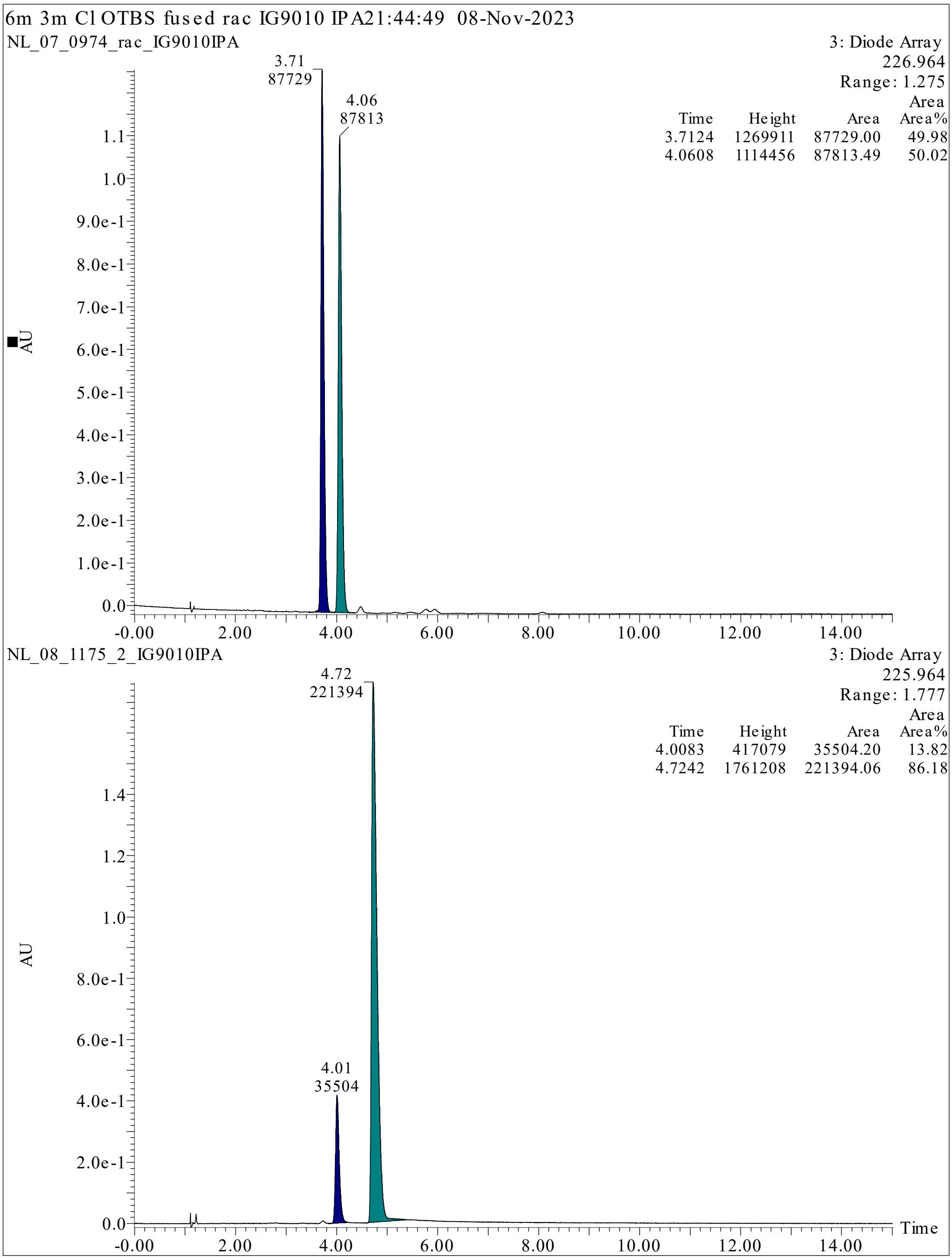

Dimethyl (1*R*,2*S*,4*S*)-4-hydroxy-5-oxocyclohexane-1,2-dicarboxylate (2y)

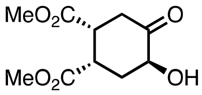

Dimethyl (1*R*,2*S*,4*S*,5*R*)-4,5-dihydroxycyclohexane-1,2-dicarboxylate **1y** (23.2 mg, 0.10 mmol) was subjected to General Procedure **B**, with 4CzIPN (3.9 mg, 0.005 mmol), *epi*-NHBoc-DHCN (4.0 mg, 0.010 mmol), TBA·H_2PO_4 (8.5 mg, 0.025 mmol), DIAD (20 mg, 0.10 mmol) in MeCN (4 mL) and reacted for 36 h. Purification by column chromatography (SiO_2, CH_2Cl_2 *then* EtOAc/hexanes 15–40%) afforded **2y** as a white solid (8.7 mg, 0.038 mmol, 38%).

^{1}H NMR (500 MHz, $CDCl_3$) δ 4.21 (dd, *J* = 12.7, 6.7 Hz, 1H), 3.77 (s, 3H), 3.74 (s, 3H), 3.72 – 3.65 (m, 1H), 3.53 (s, 1H), 3.52 – 3.47 (m, 1H), 3.17 – 3.08 (m, 1H), 2.95 – 2.84 (m, 2H), 2.87 – 2.78 (m, 1H), 1.76 (td, *J* = 13.1, 5.0 Hz, 1H).
^{13}C NMR (126 MHz, $CDCl_3$) δ 208.7, 172.7, 171.5, 71.9, 52.5, 52.4, 44.1, 40.8, 37.9, 35.7.
$[\alpha]_D$: +2.7 (c = 0.23, T = 25 °C, $CHCl_3$)
HRMS (ESI+) $[M+H]^+$ m/z calc'd for $[C_{10}H_{14}O_6H]^+$ expect 231.0869; found 231.0860

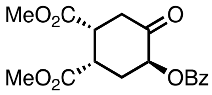

Derivatization for enantiomeric excess (ee) determination (**Bz-2y**)
Bz-2y was prepared via General Procedure **C1**. Purified by preparative thin layer chromatography (SiO_2, 20% EtOAc/hexanes) afforded an analytical sample of **Bz-2y** for ee determination. Chiral SFC analysis indicated that **2x** was formed in 72% ee. A sample of racemic **Bz-2y** was synthesized *via* General Procedure **D1**.

^{1}H NMR (700 MHz, $CDCl_3$) δ 8.09 (d, *J* = 7.7 Hz, 2H), 7.61 (t, *J* = 7.5 Hz, 1H), 7.48 (t, *J* = 7.8 Hz, 2H), 5.54 (dd, *J* = 12.7, 6.3 Hz, 1H), 3.83 (s, 3H), 3.78 (s, 3H), 3.62 (d, *J* = 4.2 Hz, 1H), 3.18 (t, *J* = 13.7 Hz, 1H), 3.00 (dt, *J* = 12.9, 4.5 Hz, 1H), 2.94 (dd, *J* = 14.3, 3.9 Hz, 1H), 2.85 (ddd, *J* = 13.4, 6.2, 3.3 Hz, 1H), 2.25 (td, *J* = 13.1, 5.1 Hz, 1H).
^{13}C NMR (176 MHz, $CDCl_3$) δ 201.6, 172.3, 171.4, 165.3, 133.4, 129.9, 129.3, 128.4, 73.7, 52.6, 52.5, 43.9, 40.8, 39.2, 32.5.
HRMS (ESI+) $[M+Na]^+$ m/z calc'd for $[C_{17}H_{18}O_7Na]^+$ expect 357.0950; found 357.0938

Chiral SFC Analysis: CHIRALPAK IG (CO_2: MeOH, 75:25, 2.5 mL min^{-1}, 40 °C, 227 nm) t_R = 5.94 (minor), 9.46 (major) minutes. 72% ee

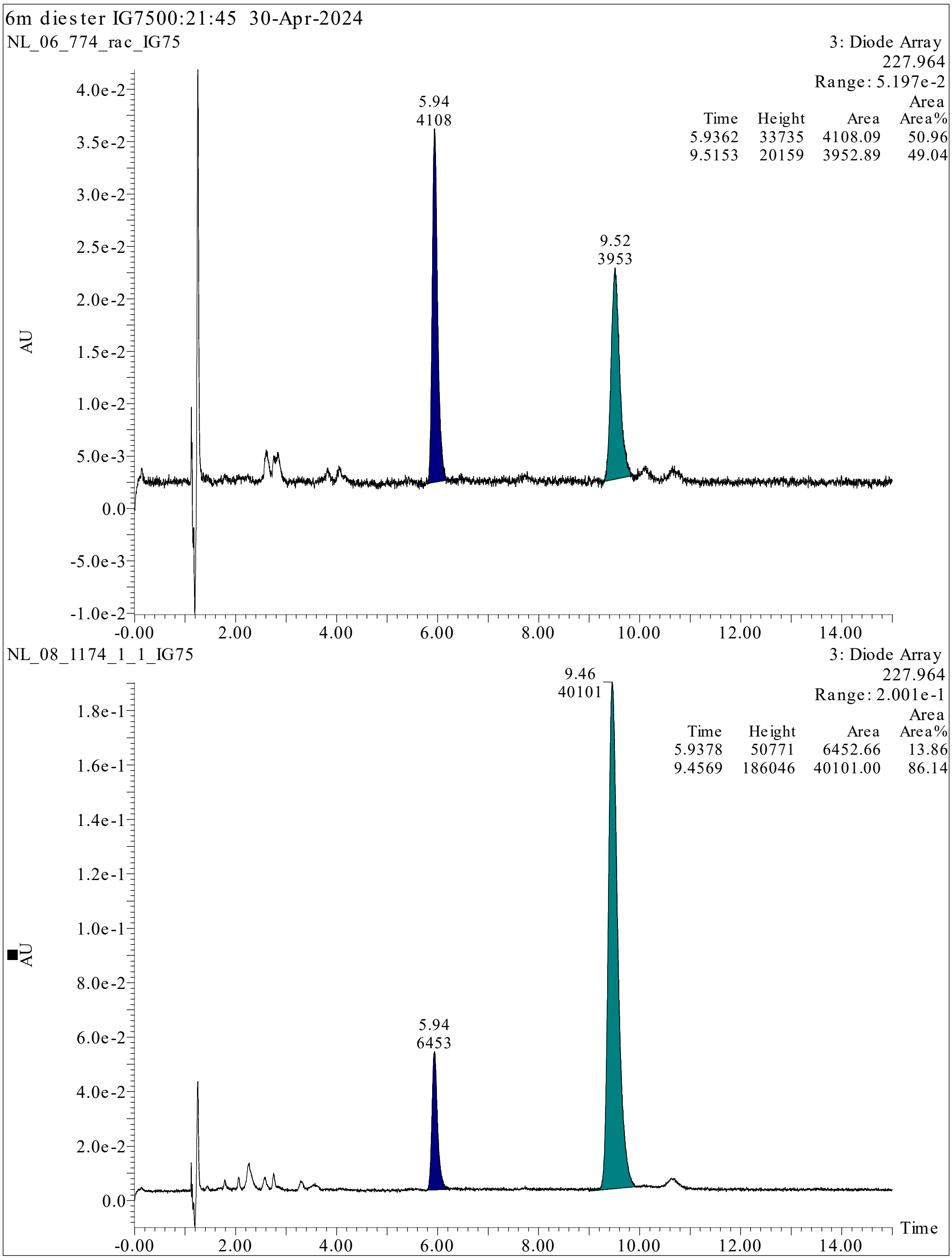

(1*S*,5*S*,8*R*)-5-hydroxy-9-oxabicyclo[6.1.0]nonan-4-one (2z)

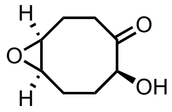

(1*R*,4*S*,5*R*,8*S*)-9-oxabicyclo[6.1.0]nonane-4,5-diol **1z** (15.8 mg, 0.10 mmol) was subjected to General Procedure **B**, with 4CzIPN (3.9 mg, 0.005 mmol), *epi*-NHBoc-DHCN (4.0 mg, 0.010 mmol), TBA·H_2PO_4 (8.5 mg, 0.025 mmol), DIAD (20 mg, 0.10 mmol) in MeCN (4 mL) and reacted for 36 h. Purification by column chromatography (SiO_2, CH_2Cl_2 *then* 20% EtOAc/hexanes) afforded **2z** as a white solid (13.6 mg, 0.087 mmol, 87%).

^{1}H NMR (700 MHz, $CDCl_3$) δ 4.37 (td, *J* = 7.0, 5.9, 2.6 Hz, 1H), 3.06 (ddd, *J* = 12.7, 5.1, 3.5 Hz, 1H), 2.94 (dt, *J* = 10.8, 4.1 Hz, 1H), 2.86 (dq, *J* = 9.5, 5.8, 4.4 Hz, 1H), 2.39 (td, *J* = 13.1, 5.2 Hz, 1H), 2.23 (dq, *J* = 13.0, 4.1 Hz, 1H), 2.15 – 2.07 (m, 2H), 2.06 – 2.00 (m, 2H), 1.96 – 1.86 (m, 1H), 1.73 – 1.60 (m, 1H).
^{13}C NMR (176 MHz, $CDCl_3$) δ 216.6, 76.5, 55.2, 37.8, 31.5, 22.0, 21.7.
$[\alpha]_D$: +50.4 (c = 0.77, T = 25 °C, $CHCl_3$)
HRMS (ESI+) $[M+H]^+$ m/z calc'd for $[C_8H_{12}O_3H]^+$ expect 157.0860; found 157.0859

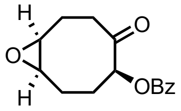

Derivatization for enantiomeric excess (ee) determination (**Bz-2z**)
Bz-2z was prepared via General Procedure **C2**. Purified by preparative thin layer chromatography (SiO_2, 20% EtOAc/hexanes) afforded an analytical sample of **Bz-2z** for ee determination. Chiral SFC analysis indicated that **2z** was formed in 93% ee. A sample of racemic **Bz-2z** was synthesized *via* General Procedure **D1**.

^{1}H NMR (700 MHz, $CDCl_3$) δ 8.09 (d, *J* = 7.7 Hz, 2H), 7.66 (t, *J* = 7.4 Hz, 1H), 7.52 (t, *J* = 7.7 Hz, 2H), 5.52 – 5.39 (m, 1H), 3.01 (dt, *J* = 10.9, 4.0 Hz, 1H), 2.95 (dt, *J* = 9.4, 4.2 Hz, 1H), 2.87 (ddd, *J* = 13.3, 5.1, 3.2 Hz, 1H), 2.55 (td, *J* = 13.7, 5.3 Hz, 1H), 2.32 (dt, *J* = 12.7, 4.2 Hz, 1H), 2.30 – 2.16 (m, 3H), 2.12 – 2.04 (m, 1H), 1.78 – 1.70 (m, 1H).
^{13}C NMR (176 MHz, $CDCl_3$) δ 211.6, 165.8, 133.8, 129.7, 129.1, 128.8, 78.3, 54.9, 54.9, 37.8, 28.4, 22.6, 22.1.
HRMS (ESI+) $[M+Na]^+$ m/z calc'd for $[C_{15}H_{16}O_4Na]^+$ expect 283.0946; found 283.0941

Chiral SFC Analysis: CHIRALPAK IG (CO_2: MeOH, 85:15, 2.5 mL min^{-1}, 40 °C, 228 nm) t_R = 6.66 (minor), 9.46 (major) minutes. 93% ee

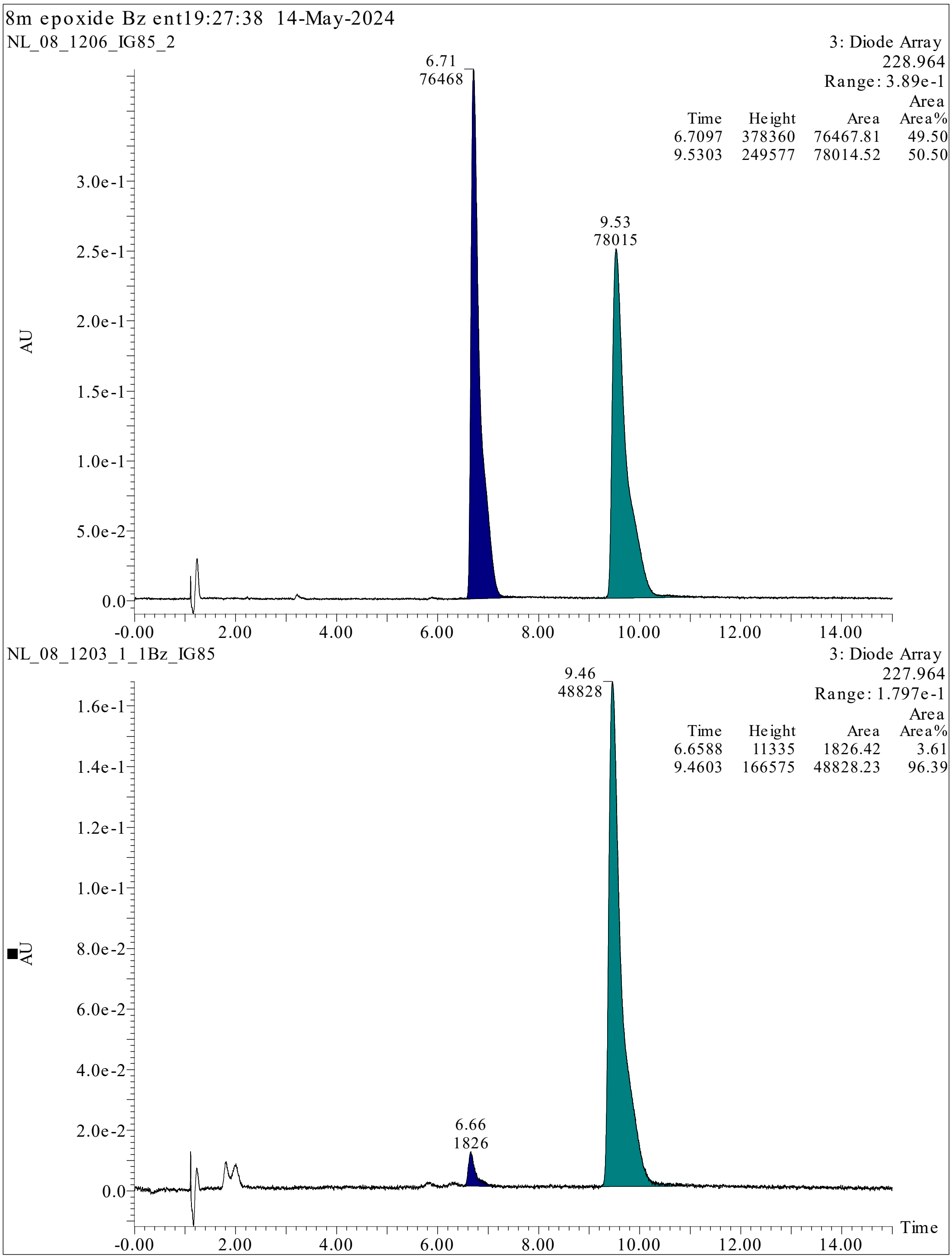

Diethyl (1*R*,4*S*,8*S*)-4-hydroxy-5-oxobicyclo[6.1.0]nonane-9,9-dicarboxylate (2aa)

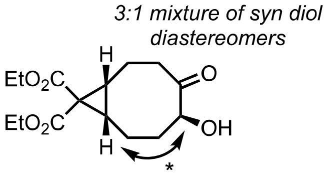

Diethyl (1*R*,4*S*,5*R*,8*S*)-4,5-dihydroxybicyclo[6.1.0]nonane-9,9-dicarboxylate **1aa** (30.0 mg, 0.10 mmol, 3:1 mixture of *syn* diol diastereomers) was subjected to General Procedure **B**, with 4CzIPN (3.9 mg, 0.005 mmol), *epi*-NHBoc-DHCN (4.0 mg, 0.010 mmol), TBA·H_2PO_4 (8.5 mg, 0.025 mmol), DIAD (20 mg, 0.10 mmol) in MeCN (4 mL) and reacted for 36 h. Purification by column chromatography (SiO_2, CH_2Cl_2 *then* 20% EtOAc/hexanes) afforded **2aa** as a white solid (26.9 mg, 0.090 mmol, 90%) as an inseparable 3:1 mixture of diastereomers relative to the cyclopropyl motif.

^{1}H NMR (700 MHz, $CDCl_3$) δ 4.47 – 4.04 (m, 5H), 3.11 – 2.77 (m, 1H), 2.75 – 2.30 (m, 2H), 2.30 – 2.18 (m, 1H), 2.17 – 1.86 (m, 3H), 1.82 – 1.68 (m, 1H), 1.67 – 1.61 (m, 2H), 1.32 – 1.27 (m, 3H), 1.26 – 1.22 (m, 3H). Reported as a combined mixture of diastereomers.
^{13}C NMR (176 MHz, $CDCl_3$) δ 217.0*, 215.8, 170.2*, 170.0, 166.7, 76.6*, 75.0, 61.8, 61.7*, 61.3, 61.2*, 42.9, 41.2*, 37.6*, 37.3, 37.0, 35.1*, 30.1*, 30.1*, 30.1, 29.6, 20.5, 19.8*, 18.4*, 18.3, 14.2, 14.1*, 14.1*, 14.0. Discernible signals from the minor (3:1) diastereomer denoted by (*)
$[\alpha]_D$: +19.1 (c = 1.4, T = 25 °C, $CHCl_3$)
HRMS (ESI+) $[M+H]^+$ m/z calc'd for $[C_{15}H_{22}O_6H]^+$ expect 299.1489; found 299.1488

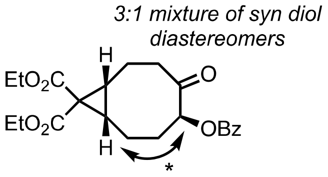

Derivatization for enantiomeric excess (ee) determination (**Bz-2aa**)
Bz-2aa was prepared via General Procedure **C2**. Purified by preparative thin layer chromatography (SiO_2, 20% EtOAc/hexanes) afforded an analytical sample of **Bz-2aa** for ee determination as an inseparable 3:1 diastereomeric mixture. Chiral SFC analysis indicated that **2aa** was formed in 94% ee and 91% ee for each respective diastereomer. A sample of racemic **Bz-2aa** was synthesized *via* General Procedure **D1**.

^{1}H NMR (700 MHz, $CDCl_3$) δ ^{1}H NMR (700 MHz, Chloroform-*d*) δ 8.08 – 8.04 (m, 2H), 7.66 – 7.59 (m, 1H), 7.51 – 7.47 (m, 2H), 5.47 + 5.18 (m, 1H), 4.33 – 4.22 (m, 2H), 4.21 – 4.17 (m, 2H), 3.01 + 2.86 (m, 1H), 2.65 + 2.54 (m, 1H), 2.39 – 2.21 (m, 3H), 2.18 – 2.06 (m, 1H), 1.85 – 1.68 (m, 3H), 1.34 – 1.31 (m, 3H), 1.29 – 1.27 (m, 3H). Reported as a combined mixture of diastereomers.
^{13}C NMR (176 MHz, $CDCl_3$) δ 211.9*, 210.7, 170.0, 170.0*, 166.7*, 166.6, 166.2, 165.8*, 133.7*, 133.5, 129.8, 129.7*, 129.3*, 129.1, 128.7*, 128.5, 78.5*, 77.4, 61.8, 61.3, 61.2*, 42.0, 41.2, 37.6*, 37.4, 31.8*, 31.7, 30.1, 29.9*, 29.8*, 29.7, 20.7, 19.9*, 19.8, 19.3, 14.2*, 14.2, 14.1. Discernible signals from the minor (3:1) diastereomer denoted by (*)
HRMS (ESI+) $[M+H]^+$ m/z calc'd for $[C_{22}H_{26}O_7H]^+$ expect 403.1751; found 403.1753

Chiral SFC Analysis: CHIRALPAK IC (CO_2: MeOH, 90:10, 2.5 mL min^{-1}, 40 °C, 228 nm)
Diastereomeric ratio: 3:1 (major:minor) as indicated by NMR and relative integration in the SFC trace for racemic **2aa** (top two traces). Retention times arising from each racemic pair of diastereomers deduced by relative integrations.
Diastereomer 1 (major): t_R = 6.02 (minor), 6.45 (major) minutes. 94% ee (middle)
Diastereomer 2 (minor): t_R = 5.12 (minor), 5.57 (major) minutes. 91% ee (bottom)

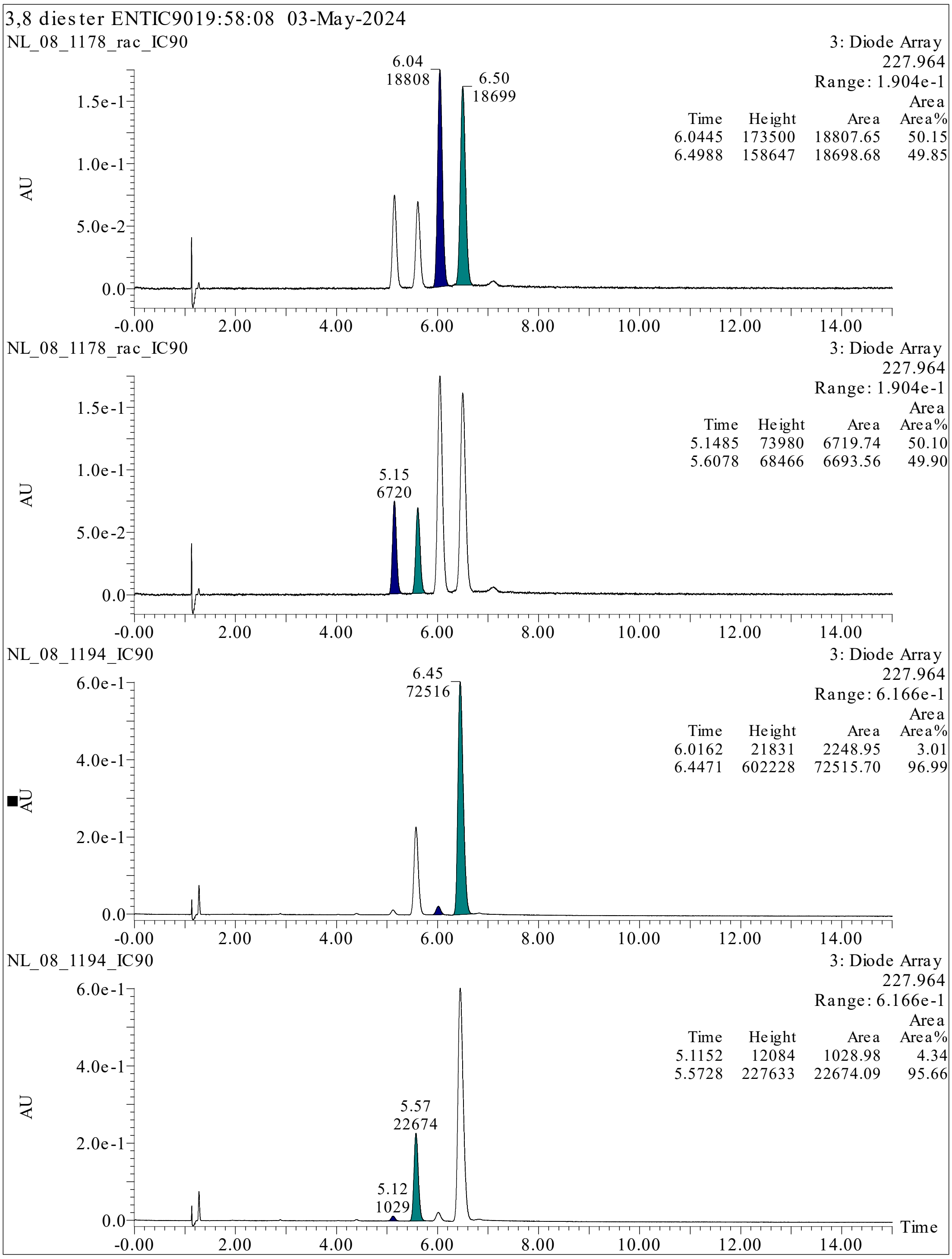

(3a*S*,7*S*,9a*R*)-7-hydroxy-2,2-dimethylhexahydrocycloocta[*d*][1,3]dioxol-6(3a*H*)-one (2ab)

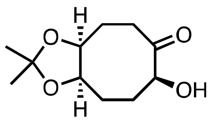

(3a*R*,6*S*,7*R*,9a*S*)-2,2-dimethyloctahydrocycloocta[*d*][1,3]dioxole-6,7-diol **1ab** (21.6 mg, 0.10 mmol) was subjected to General Procedure **B**, with 4CzIPN (3.9 mg, 0.005 mmol), *epi*-NHBoc-DHCN (4.0 mg, 0.010 mmol), TBA·H_2PO_4 (8.5 mg, 0.025 mmol), DIAD (20 mg, 0.10 mmol) in MeCN (4 mL) and reacted for 36 h. Purification by column chromatography (SiO_2, CH_2Cl_2 *then* 20% EtOAc/hexanes) afforded **2ab** as a clear oil (20.8 mg, 0.097 mmol, 97%).

^{1}H NMR (700 MHz, $CDCl_3$) δ 4.33 – 4.26 (m, 1H), 4.21 – 4.13 (m, 2H), 3.14 (d, *J* = 4.1 Hz, 1H), 2.69 (ddd, *J* = 13.0, 8.6, 2.3 Hz, 1H), 2.40 (ddd, *J* = 13.0, 11.9, 2.7 Hz, 1H), 2.16 – 2.08 (m, 1H), 2.07 – 1.90 (m, 3H), 1.80 (ddt, *J* = 15.3, 7.6, 3.6 Hz, 1H), 1.49 – 1.42 (m, 1H), 1.39 (s, 3H), 1.31 (s, 3H).
^{13}C NMR (176 MHz, $CDCl_3$) δ 214.6, 107.5, 78.7, 77.4, 76.9, 36.7, 28.4, 28.2, 26.9, 25.5, 23.4.
$[\alpha]_D$: +4.1 (c = 1.1, T = 25 °C, $CHCl_3$)
HRMS (ESI+) $[M+H]^+$ m/z calc'd for $[C_{11}H_{18}O_4H]^+$ expect 215.1283; found 215.1286

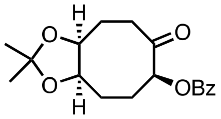

Derivatization for enantiomeric excess (ee) determination (**Bz-2ab**)
Bz-2ab was prepared via General Procedure **C1**. Purified by preparative thin layer chromatography (SiO_2, 10% EtOAc/CH_2Cl_2) afforded an analytical sample of **Bz-2ab** for ee determination. Chiral SFC analysis indicated that **2ab** was formed in 93% ee. A sample of racemic **Bz-2ab** was synthesized *via* General Procedure **D1**.

^{1}H NMR (700 MHz, $CDCl_3$) δ 8.09–7.99 (m, 2H), 7.65–7.55 (m, 1H), 7.50–7.44 (m, 2H), 5.26 (dd, *J* = 11, 4.3 Hz, 1H), 4.23–4.06 (m, 1H), 3.93 (dd, *J* = 10.2, 5.5 Hz, 1H), 3.11 (ddd, *J* = 14.3, 5.5, 3.4 Hz, 1H), 2.55–2.45 (m, 1H), 2.36–2.22 (m, 3H), 2.10–1.93 (m, 2H), 1.84 (dd, *J* = 14.5, 4.6 Hz, 1H), 1.44 (s, 3H), 1.34 (s, 3H).
^{13}C NMR (176 MHz, $CDCl_3$) δ 209.7, 166.2, 133.5, 129.8, 128.9, 128.5, 107.0, 78.1, 76.1, 39.8, 29.7, 28.1, 26.8, 25.4, 23.5.
HRMS (ESI+) $[M+H]^+$ m/z calc'd for $[C_{18}H_{22}O_5H]^+$ expect 319.1545; found 319.1537

Chiral HPLC Analysis: CHIRALPAK IG (*n*-hexane: *i*PrOH, 90:10, 1.25 mL min^{-1}, 40 °C, 230 nm) t_R = 9.04 (minor), 13.41 (major) minutes. 93% ee

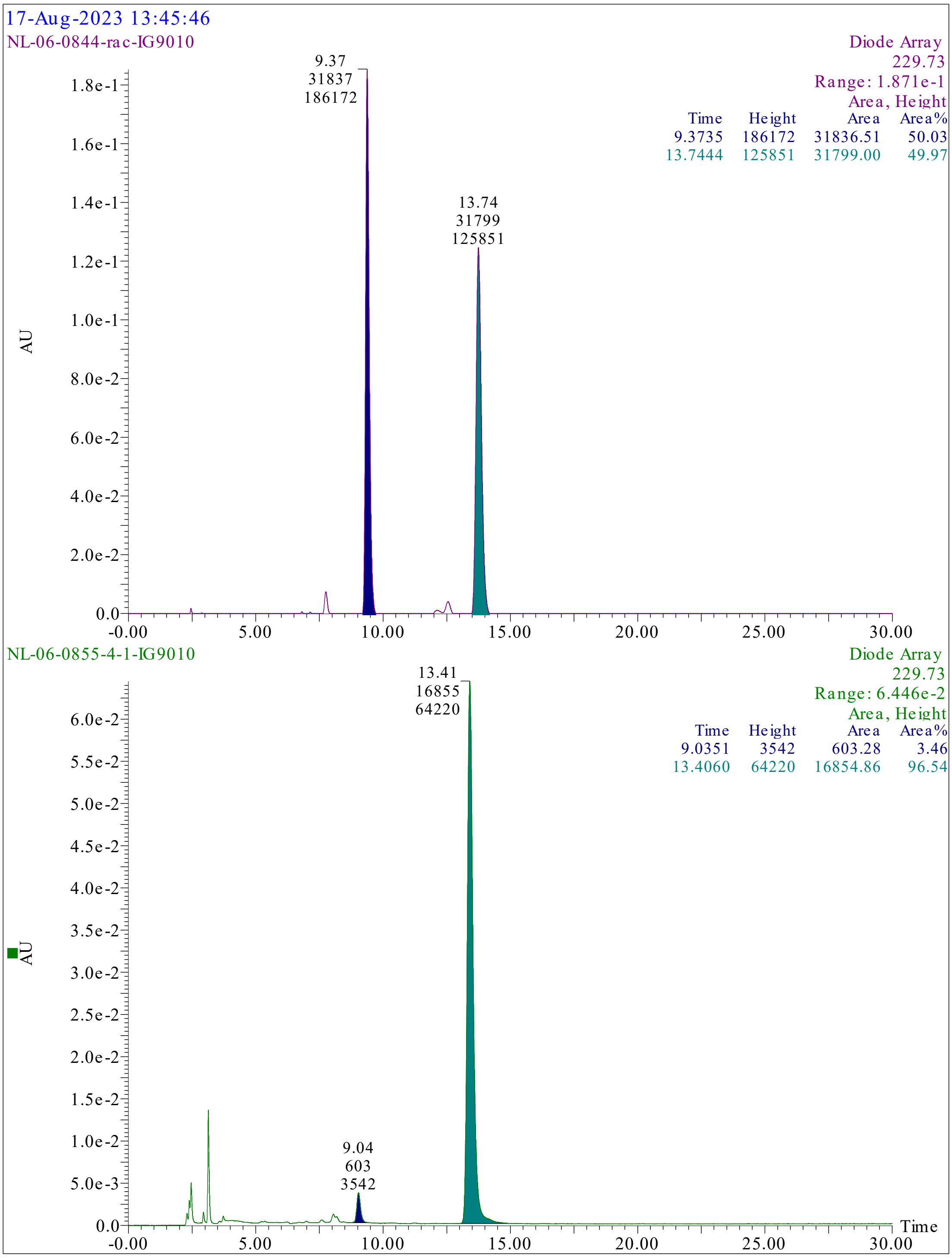

(3a*R*,7*S*,9a*S*)-7-hydroxy-2,2-dimethylhexahydrocycloocta[*d*][1,3]dioxol-6(3a*H*)-one (2ac)

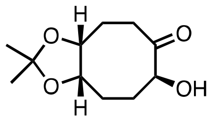

(3a*R*,6*R*,7*S*,9a*S*)-2,2-dimethyloctahydrocycloocta[*d*][1,3]dioxole-6,7-diol **1ac** (21.6 mg, 0.10 mmol) was subjected to General Procedure **B**, with 4CzIPN (3.9 mg, 0.005 mmol), *epi*-NHBoc-DHCN (4.0 mg, 0.010 mmol), TBA·H_2PO_4 (8.5 mg, 0.025 mmol), DIAD (20 mg, 0.10 mmol) in MeCN (4 mL) and reacted for 36 h. Purification by column chromatography (SiO_2, CH_2Cl_2 *then* 20% EtOAc/hexanes) afforded **2ac** as a clear oil (11.4 mg, 0.053 mmol, 53%).

^{1}H NMR (700 MHz, $CDCl_3$) δ 4.38 (ddd, *J* = 9.7, 7.7, 4.4 Hz, 1H), 4.13 (ddd, *J* = 10.8, 5.5, 3.3 Hz, 1H), 3.87 (ddd, *J* = 10.0, 5.6, 1.3 Hz, 1H), 3.07 (d, *J* = 7.7 Hz, 1H), 2.73 (ddd, *J* = 15.6, 6.9, 2.9 Hz, 1H), 2.53 (ddd, *J* = 15.8, 12.9, 3.3 Hz, 1H), 2.32 – 2.25 (m, 1H), 2.27 – 2.19 (m, 1H), 2.15 (ddt, *J* = 14.9, 6.7, 3.3 Hz, 1H), 1.99 (dddd, *J* = 15.1, 12.4, 10.1, 2.5 Hz, 1H), 1.65 (dddd, *J* = 15.5, 6.7, 2.8, 1.5 Hz, 1H), 1.57 – 1.54 (m, 1H), 1.45 (s, 3H), 1.33 (s, 3H).
^{13}C NMR (176 MHz, $CDCl_3$) δ 214.8, 107.2, 78.1, 77.2, 74.7, 37.9, 33.4, 28.0, 26.6, 25.5, 23.0.
$[\alpha]_D$: +10.4 (c = 0.67, T = 25 °C, $CHCl_3$)
HRMS (ESI+) $[M+H]^+$ m/z calc'd for $[C_{11}H_{18}O_4H]^+$ expect 215.1283; found 215.1286

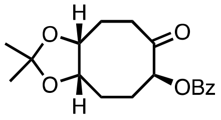

Derivatization for enantiomeric excess (ee) determination (**Bz-2ac**)
Bz-2ac was prepared via General Procedure **C1**. Purified by preparative thin layer chromatography (SiO_2, 10% EtOAc/CH_2Cl_2) afforded an analytical sample of **Bz-2ac** for ee determination. Chiral SFC analysis indicated that **2ac** was formed in 94% ee. A sample of racemic **Bz-2ac** was synthesized *via* General Procedure **D1**.

^{1}H NMR (700 MHz, $CDCl_3$) δ 8.16–8.07 (m, 2H), 7.68–7.59 (m, 1H), 7.54–7.46 (m, 2H), 5.33 (dd, *J* = 6.3, 3.2 Hz, 1H), 4.18 (ddd, *J* = 11.4, 5.7, 2.8 Hz, 1H), 4.03–3.93 (m, 1H), 2.77 (ddd, *J* = 13.5, 4.9, 3.7 Hz, 1H), 2.55 (tdd, *J* = 14.0, 11.4, 3.6 Hz, 1H), 2.46 (td, *J* = 13.5, 4.3 Hz, 1H), 2.21 (dtd, *J* = 12.1, 6.8, 2.0 Hz, 1H), 2.17–2.02 (m, 3H), 1.78–1.71 (m, 1H), 1.43 (s, 3H), 1.32 (s, 3H)
^{13}C NMR (176 MHz, $CDCl_3$) δ 211.0, 165.9, 133.8, 129.8, 129.1, 128.7, 106.8, 78.8, 78.4, 39.1, 29.9, 27.9, 25.9, 25.1, 24.5.
HRMS (ESI+) $[M+H]^+$ m/z calc'd for $[C_{18}H_{22}O_5H]^+$ expect 319.1545; found 319.1538

Chiral HPLC Analysis: CHIRALPAK IG (*n*-hexane: *i*PrOH, 90:10, 1.25 mL min^{-1}, 40 °C, 230 nm) t_R = 11.14 (minor), 12.13 (major) minutes. 94% ee

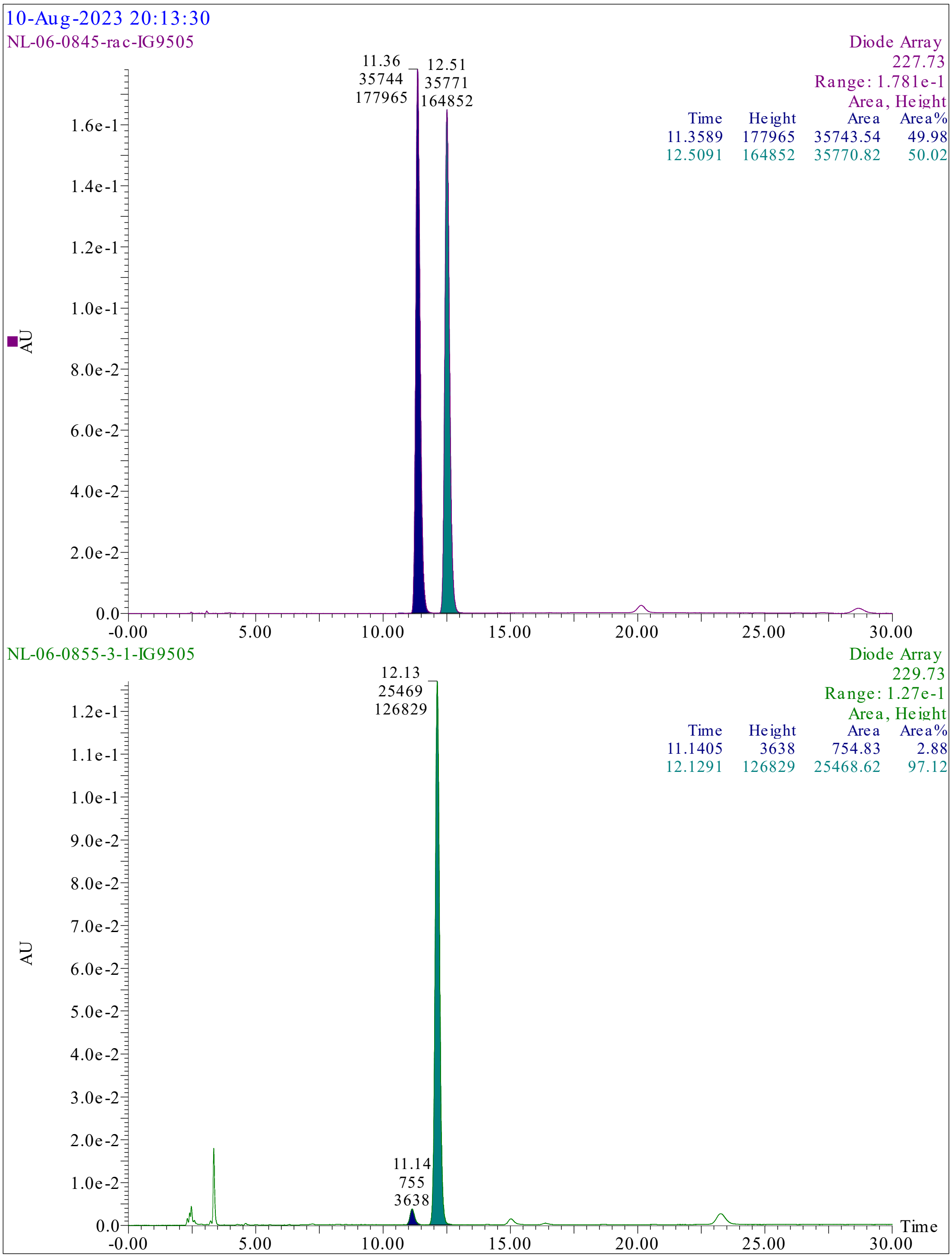

(*S*)-4-hydroxyhexan-3-one (4a)

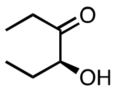

(3*R*,4*S*)-hexane-3,4-diol **3a** (11.8 mg, 0.10 mmol) was subjected to General Procedure **B**, with 4CzIPN (3.9 mg, 0.005 mmol), *epi*-NHBoc-DHCN (4.0 mg, 0.010 mmol), TBA·H_2PO_4 (8.5 mg, 0.025 mmol), DIAD (20 mg, 0.10 mmol) in MeCN (4 mL) and reacted for 24 h. Purification by column chromatography (SiO_2, 50-100% CH_2Cl_2/hexanes) afforded **4a** as a clear oil (9.4 mg, 0.081 mmol, 81%). **4a** is volatile and care should be taken on solvent evaporation to avoid product losses

^{1}**H NMR** (700 MHz, $CDCl_3$) δ 4.17 (dt, *J* = 6.6, 4.4 Hz, 1H), 3.49 (d, *J* = 4.9 Hz, 1H), 2.59 – 2.36 (m, 2H), 1.90 (dqd, *J* = 14.8, 7.5, 4.0 Hz, 1H), 1.65 – 1.57 (m , 1H), 1.12 (t, *J* = 7.3 Hz, 3H), 0.93 (t, *J* = 7.4 Hz, 3H).
^{13}**C NMR** (176 MHz, $CDCl_3$) δ 212.8, 77.0, 31.1, 26.8, 8.8, 7.6.
[α]$_D$: +88.8 (c = 0.42, T = 25 °C, $CHCl_3$)
Literature [α]$_D$ values for ***S*****-4a***:* +97.5 (c = 1.0; T = 22 °C $CHCl_3$).
Absolute configuration of **4a** *therefore assigned as* ***S***

NMR in agreement with the previously reported data[28]

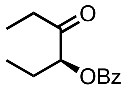

Derivatization for enantiomeric excess (ee) determination (**Bz-4a**)
Bz-4a was prepared via General Procedure **C1**. Purified by preparative thin layer chromatography (SiO_2, 10% EtOAc/hexanes) afforded an analytical sample of **Bz-4a** for ee determination. Chiral SFC analysis indicated that **4a** was formed in 96% ee. A sample of racemic **Bz-4a** was synthesized *via* General Procedure **D2**.

^{1}**H NMR** (700 MHz, $CDCl_3$) δ 8.12 (d, *J* = 8.2 Hz, 2H), 7.62 (t, *J* = 7.0 Hz, 1H), 7.50 (t, *J* = 7.6 Hz, 2H), 5.26 (dd, *J* = 7.9, 4.5 Hz, 1H), 2.72 – 2.58 (m, 1H), 2.58 – 2.40 (m, 1H), 2.04 – 1.89 (m, 2H), 1.11 (t, *J* = 7.3 Hz, 3H), 1.08 (t, *J* = 7.5 Hz, 3H).
^{13}**C NMR** (176 MHz, $CDCl_3$) δ 208.2, 166.2, 133.4, 129.8, 129.6, 128.5, 79.9, 32.1, 24.3, 9.7, 7.2.

NMR in agreement with the previously reported data[29]

Chiral HPLC Analysis: CHIRALPAK IG (*n*-hexane: *i*PrOH, 97:3, 1.25 mL min^{-1}, 40 °C, 226 nm) t_R = 7.83 (minor), 10.53 (major) minutes. 96% ee

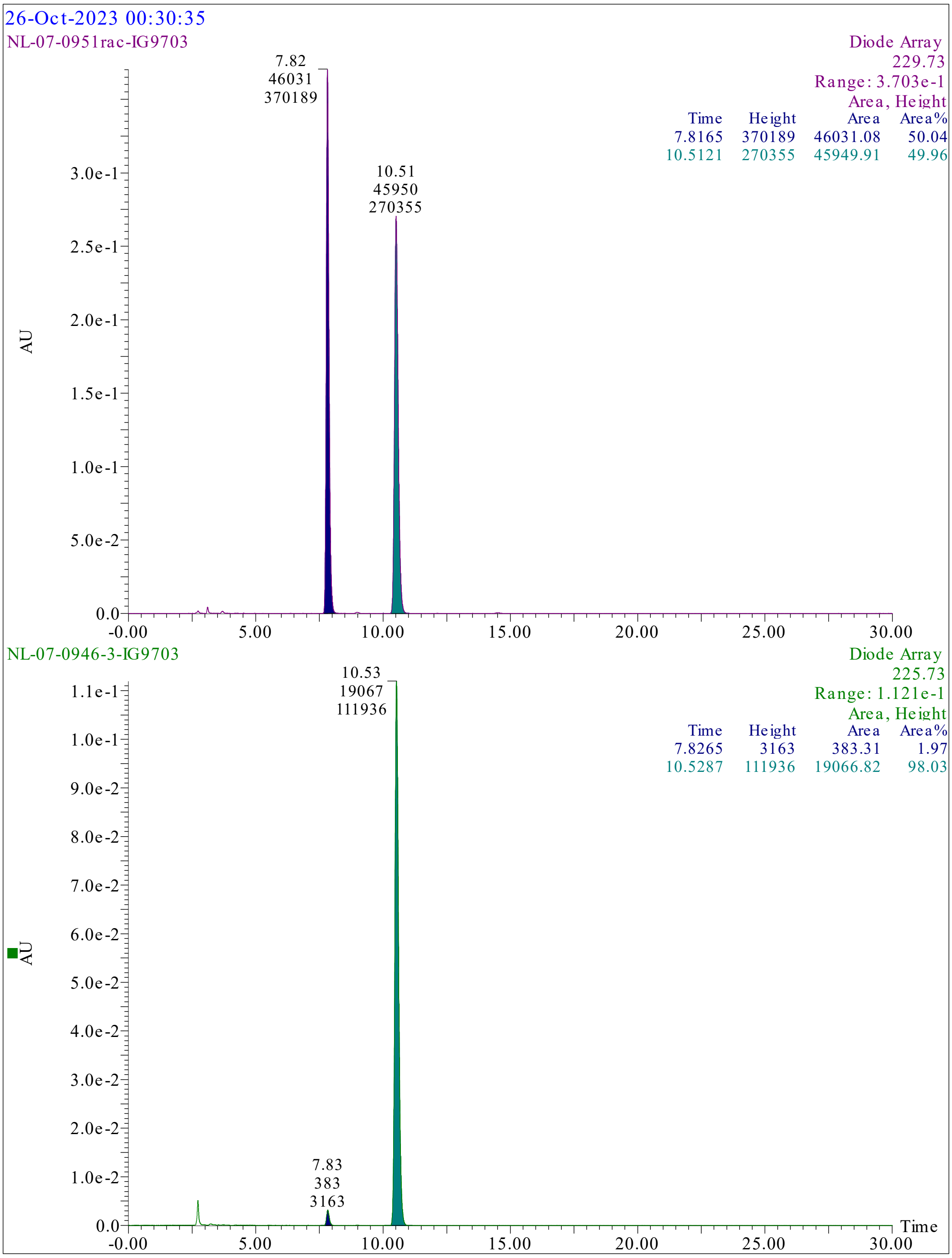

(*S*)-5-hydroxyoctan-4-one (4b)

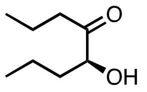

(4*R*,5*S*)-octane-4,5-diol **3b** (14.6 mg, 0.10 mmol) was subjected to General Procedure **B**, with 4CzIPN (3.9 mg, 0.005 mmol), *epi*-NHBoc-DHCN (4.0 mg, 0.010 mmol), TBA·H_2PO_4 (8.5 mg, 0.025 mmol), DIAD (20 mg, 0.10 mmol) in MeCN (4 mL) and reacted for 24 h. Purification by column chromatography (SiO_2, 50-100% CH_2Cl_2/hexanes) afforded **4b** as a clear oil (12.6 mg, 0.088 mmol, 88%).

Accessing product enantiomer (*ent*-**4b**)
3b (14.6 mg, 0.10 mmol) was subjected to General Procedure **B**, with 4CzIPN (3.9 mg, 0.005 mmol), *epi*-NHBoc-DHCD (4.0 mg, 0.01 mmol), TBA·H_2PO_4 (8.5 mg, 0.025 mmol), DIAD (20 mg, 0.10 mmol) in MeCN (4 mL) and reacted for 24 h. Purification by column chromatography (SiO_2, 50-100% CH_2Cl_2/hexanes) afforded ***ent*-4b** as a clear oil (10.9 mg, 0.076 mmol, 76%)

^{1}H NMR (700 MHz, $CDCl_3$) δ 4.19 (s, 1H), 3.51 (d, *J* = 4.3 Hz, 1H), 2.60 – 2.28 (m, 2H), 1.82 (ddd, *J* = 11.0, 9.0, 4.4 Hz, 1H), 1.69 (h, *J* = 7.4 Hz, 2H), 1.58 – 1.46 (m, 2H), 1.47 – 1.35 (m, 1H), 1.07 – 0.91 (m, 6H).
^{13}C NMR (176 MHz, $CDCl_3$) δ 212.4, 76.2, 39.7, 35.8, 18.2, 17.1, 13.9, 13.7.
$[\alpha]_D$: +53.9 (**4b**, c = 0.56, T = 25 °C, $CHCl_3$), –44.3 (*ent*-**4b**, c = 0.53, T = 25 °C, $CHCl_3$)
*Literature $[\alpha]_D$ values for **R**-***4b***:* –13.8 (c = 0.5; T = 20 °C $CHCl_3$).[30]
Absolute configuration of **4b** *therefore assigned as* ***S****, and ent*-**4b** *as* ***R***

NMR in agreement with the previously reported data[30]

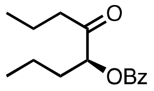

Derivatization for enantiomeric excess (ee) determination (**Bz-4b** and **Bz-*ent*-4b**)
Bz-4b and **Bz-*ent*-4b** was prepared via General Procedure **C1**. Purified by preparative thin layer chromatography (SiO_2, 10% EtOAc/hexanes) afforded an analytical sample of **Bz-4b** and **Bz-*ent*-4b** for ee determination. A sample of racemic **Bz-4b** was synthesized *via* General Procedure **D2**.

^{1}H NMR (700 MHz, $CDCl_3$) δ 8.12 (dd, *J* = 8.3, 1.2 Hz, 2H), 7.66 – 7.59 (m, 1H), 7.53 – 7.46 (m, 2H), 5.31 – 5.24 (m, 1H), 2.65 – 2.55 (m, 1H), 2.47 (dt, *J* = 17.5, 7.2 Hz, 1H), 1.94 – 1.86 (m, 2H), 1.67 (ddd, *J* = 14.6, 7.4, 2.1 Hz, 2H), 1.54 (ddt, *J* = 11.9, 7.4, 3.8 Hz, 2H), 1.01 (t, *J* = 7.4 Hz, 3H), 0.95 (t, *J* = 7.4 Hz, 3H).
^{13}C NMR (176 MHz, $CDCl_3$) δ 207.7, 166.2, 133.4, 129.8, 129.6, 128.5, 78.8, 40.5, 32.7, 18.7, 16.6, 13.8, 13.7.
HRMS (ESI+) $[M+H]^+$ m/z calc'd for $[C_{15}H_{20}O_3H]^+$ expect 249.1491; found 249.1493

Chiral SFC Analysis: CHIRALPAK IG (CO_2: MeOH, 98:02, 2.5 mL min^{-1}, 40 °C, 227 nm)

Bz-4b (middle): t_R = 4.87 (minor), 7.16 (major) minutes, 97% ee

Bz-*ent*-4b *via epi*-NHBoc-DHCD (bottom): t_R = 4.81 (major), 7.15 (major) minutes, –97% ee

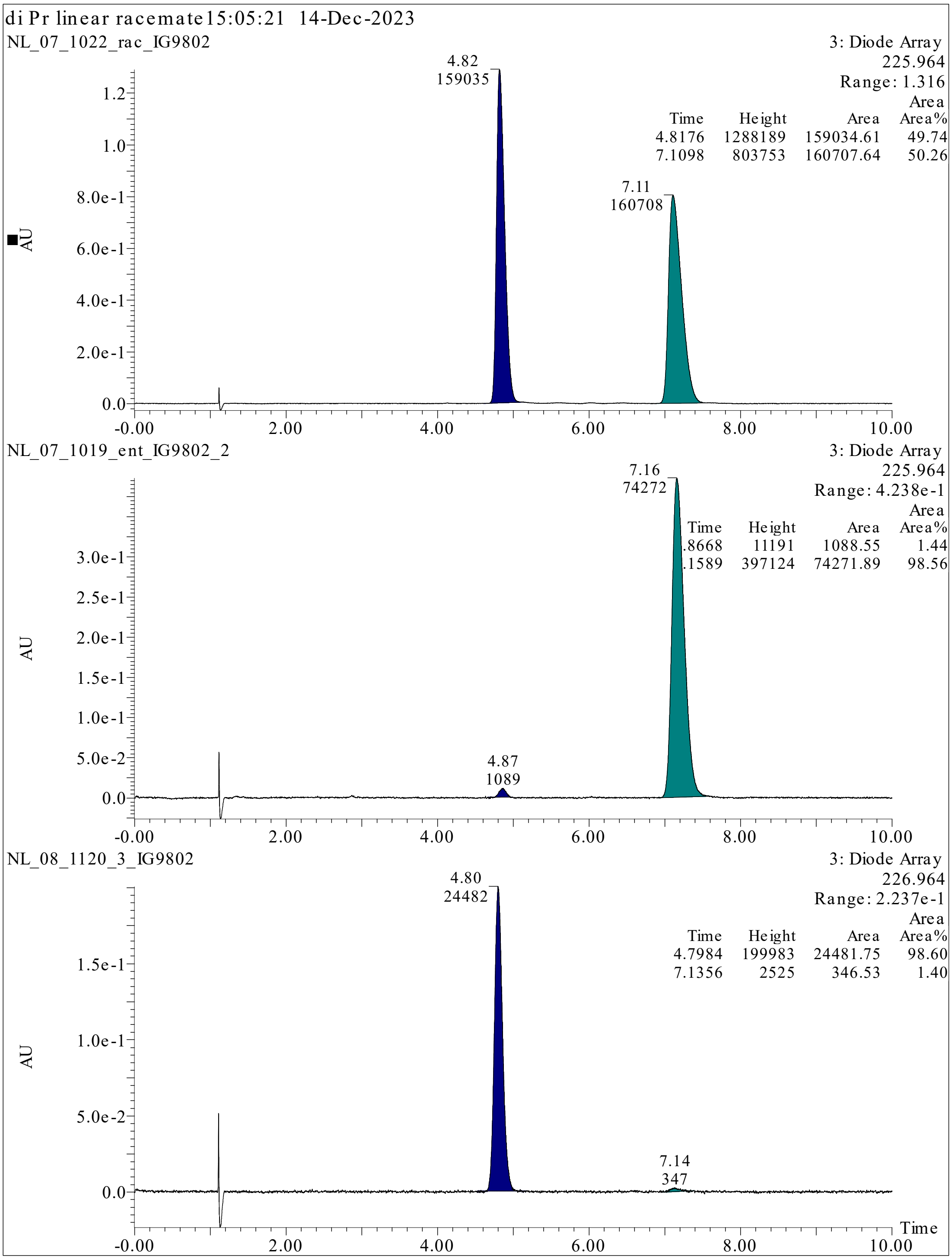

(*S*)-6-hydroxydecan-5-one (4c)

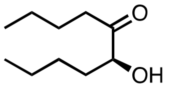

(5*R*,6*S*)-decane-5,6-diol **3c** (17.4 mg, 0.10 mmol) was subjected to General Procedure **B**, with 4CzIPN (3.9 mg, 0.005 mmol), *epi*-NHBoc-DHCN (4.0 mg, 0.010 mmol), TBA·H_2PO_4 (8.5 mg, 0.025 mmol), DIAD (20 mg, 0.10 mmol) in MeCN (4 mL) and reacted for 24 h. Purification by column chromatography (SiO_2, 50-100% CH_2Cl_2/hexanes) afforded **4c** as a clear oil (12.5 mg, 0.073 mmol, 73%).

^{1}H NMR (700 MHz, $CDCl_3$) δ 4.17 (dt, *J* = 8.3, 4.4 Hz, 1H), 3.48 (d, *J* = 4.9 Hz, 1H), 2.62 – 2.22 (m, 2H), 1.83 (ddt, *J* = 14.0, 9.2, 4.3 Hz, 1H), 1.61 (p, *J* = 7.5 Hz, 2H), 1.51 (dd, *J* = 13.8, 3.8 Hz, 1H), 1.48 – 1.41 (m, 1H), 1.34 (tt, *J* = 14.6, 7.2 Hz, 5H), 0.95 – 0.89 (m, 6H).
^{13}C NMR (176 MHz, $CDCl_3$) δ 212.5, 76.4, 37.6, 33.5, 27.0, 25.7, 22.6, 22.4, 13.9, 13.8.
$[\alpha]_D$: +62.1 (c = 0.73, T = 25 °C, $CHCl_3$)
Literature $[\alpha]_D$ value for ***S*-4b***:* +9.0 (c = 0.63; T = 20 °C, MeOH)[31]
Absolute configuration of **4c** *therefore assigned as* ***S***

NMR in agreement with the previously reported data[31]

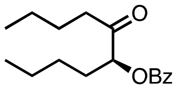

Derivatization for enantiomeric excess (ee) determination (**Bz-4c**)
Bz-4c was prepared via General Procedure **C1**. Purified by preparative thin layer chromatography (SiO_2, 10% EtOAc/hexanes) afforded an analytical sample of **Bz-4c** for ee determination. Chiral SFC analysis indicated that **4c** was formed in 88% ee. A sample of racemic **Bz-4c** was synthesized *via* General Procedure **D2**.

^{1}H NMR (700 MHz, $CDCl_3$) δ 8.09 (dd, *J* = 8.4, 1.3 Hz, 2H), 7.62 – 7.57 (m, 1H), 7.50 – 7.45 (m, 2H), 5.24 (dd, *J* = 8.0, 4.8 Hz, 1H), 2.58 (ddd, *J* = 17.4, 8.3, 6.5 Hz, 1H), 2.46 (ddd, *J* = 17.4, 8.2, 6.6 Hz, 1H), 1.94 – 1.83 (m, 2H), 1.66 – 1.57 (m, 2H), 1.49 – 1.28 (m, 6H), 0.91 (dt, *J* = 19.4, 7.4 Hz, 6H).
^{13}C NMR (176 MHz, $CDCl_3$) δ 207.8, 166.2, 133.3, 129.8, 129.6, 128.5, 79.0, 38.4, 30.4, 27.5, 25.3, 22.4, 22.3, 13.9 (2C).
HRMS (ESI+) $[M+H]^+$ m/z calc'd for $[C_{17}H_{25}O_3H]^+$ expect 277.1804; found 277.1804

Chiral SFC Analysis: CHIRALPAK IG (CO_2: MeOH, 98:2, 2.5 mL min^{-1}, 40 °C, 227 nm) t_R = 5.12 (minor), 8.33 (major) minutes. 88% ee

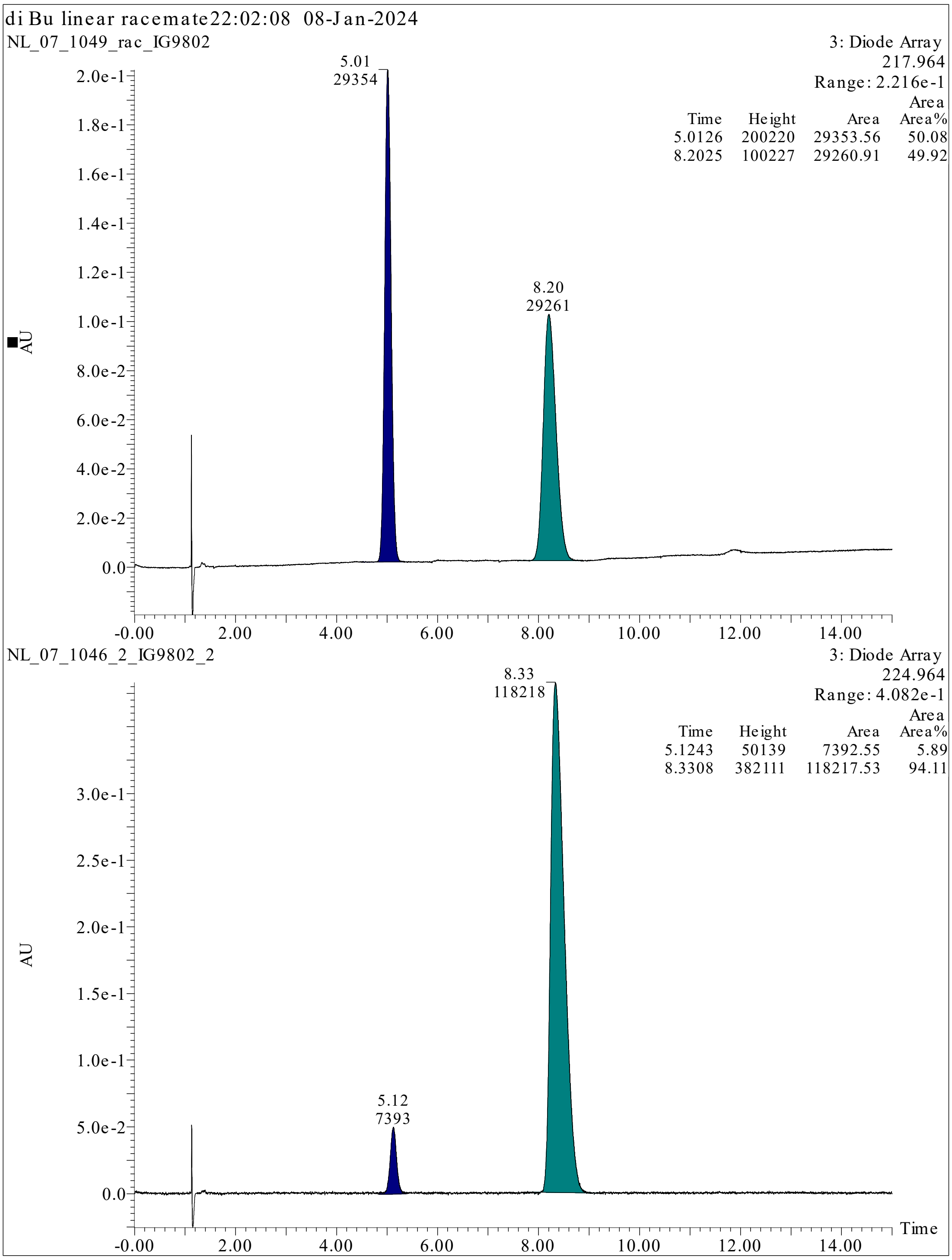

(*S*)-3-hydroxy-1,4-diphenylbutan-2-one (4d)

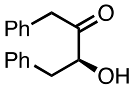

Following General Procedure **B** for the asymmetric oxidation using (2*R*,3*S*)-1,4-diphenylbutane-2,3-diol **3d** (12.1 mg, 0.050 mmol, 1 eq), *epi*-NHBoc-DHCN (2.0 mg, 0.005 mmol, 10 mol%), 4CzIPN (2.0 mg, 0.0025 mmol, 5 mol%), TBA·H_2PO_4 (4.3 mg, 0.0125 mmol, 25 mol%) and DIAD (10 mg, 0.050 mmol, 1 eq) in acetonitrile (4 ml, 0.025 M), for 24 h at –35 °C. The crude mixture was purified by column chromatography (eluting with 20% EtOAc in hexane) to yield the title compound **4d** (5.5 mg, 0.022 mmol, 46% yield, 90% ee) as a colourless solid. A sample of racemic **4d** was prepared via General Procedure **B** using quinuclidine (10 mol%) as the HAA catalyst.

^{1}H NMR (700 MHz, $CDCl_3$) δ 7.35 – 7.30 (m, 4H)), 7.30–7.24 (m, 2H), 7.22 (d, *J* = 7.4 Hz, 2H), 7.16 (d, *J* = 6.7 Hz, 2H), 4.51 (ddd, *J* = 7.5, 5.6, 4.8 Hz, 1H), 3.81 (d, *J* = 15.8 Hz, 1H), 3.76 (d, *J* = 15.9 Hz, 1H), 3.20 (d, *J* = 5.7 Hz, 1H), 3.16 (dd, *J* = 14.1, 4.8 Hz, 1H), 2.90 (dd, *J* = 14.1, 7.5 Hz, 1H).
^{13}C NMR (176 MHz, $CDCl_3$) δ 209.2, 136.6, 133.1, 129.7, 129.5, 129.0, 128.8, 127.5, 127.2, 76.9, 45.9, 40.4.
HRMS (ESI+) $[M+H]^+$ m/z calc'd for $[C_{16}H_{16}O_2H]^+$ = 241.1223, found = 241.1222.
$[\alpha]_D$: +14.6 (c = 0.27, T = 25 °C, $CHCl_3$)
*Literature $[\alpha]_D$ value for **S**-*4d*:* +75.9 (c = 0.62; T = 25 °C $CHCl_3$).[32]
Absolute configuration of 4d *therefore assigned as **S***

Chiral SFC Analysis: CHIRALPAK IG (CO_2: MeOH, 75:25, 2.5 mL min^{-1}, 40 °C, 201 nm) t_R = 4.56 (major), 8.28 (minor) minutes, 90% ee

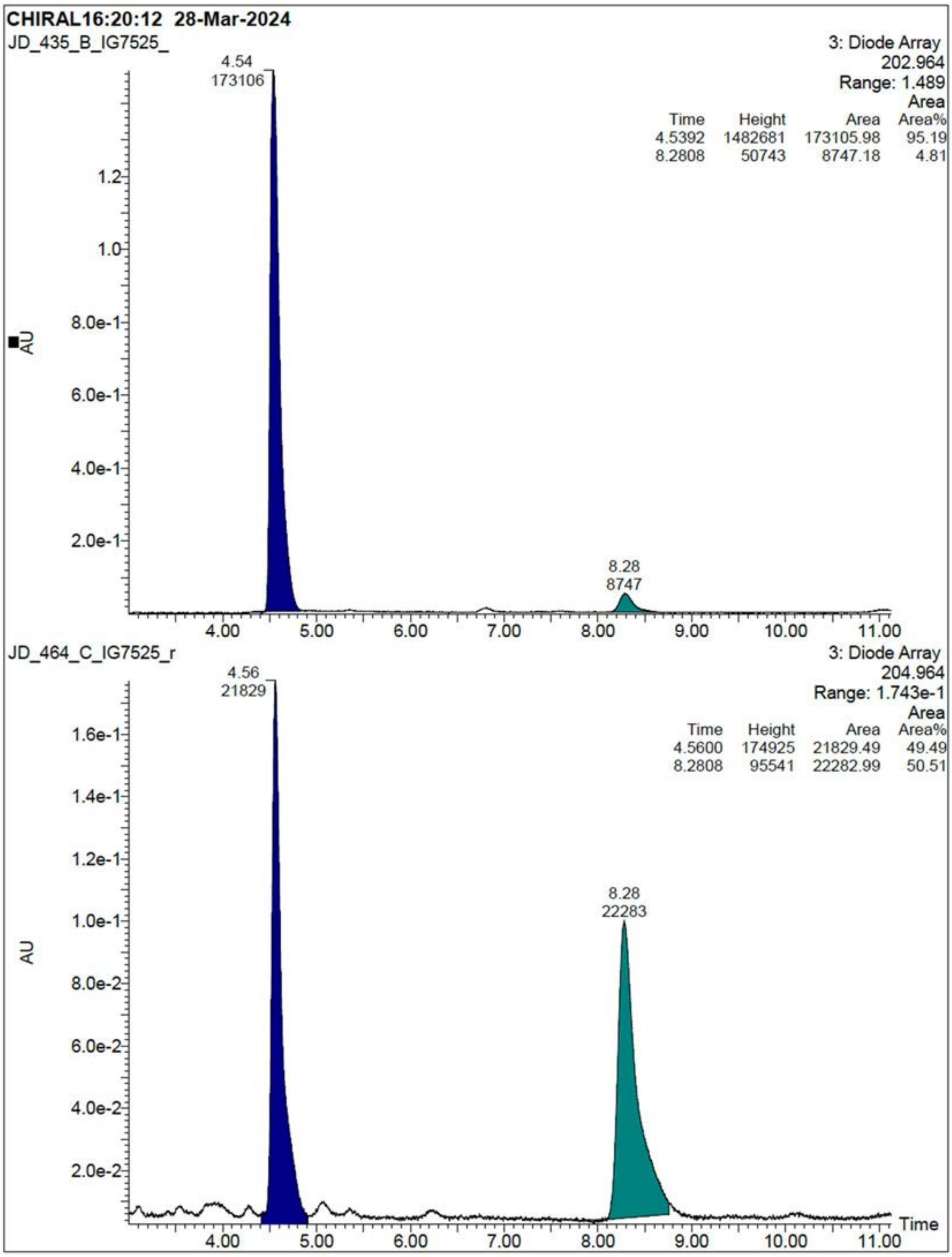

Time	Height	Area	Area%
4.5392	1482681	173105.98	95.19
8.2808	50743	8747.18	4.81

Time	Height	Area	Area%
4.5600	174925	21829.49	49.49
8.2808	95541	22282.99	50.51

(*S*)-5-hydroxy-1,8-diphenyloctan-4-one (4e)

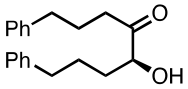

(4*R*,5*S*)-1,8-diphenyloctane-4,5-diol **3e** (29.8 mg, 0.10 mmol) was subjected to General Procedure **B**, with 4CzIPN (3.9 mg, 0.005 mmol), *epi*-NHBoc-DHCN (4.0 mg, 0.010 mmol), TBA·H_2PO_4 (8.5 mg, 0.025 mmol), DIAD (20 mg, 0.10 mmol) in MeCN (4 mL) and reacted for 24 h. Purification by column chromatography (SiO_2, 50-100% CH_2Cl_2/hexanes) afforded **4e** as a white solid (20.1 mg, 0.068 mmol, 68%). Chiral SFC analysis indicated that the product was obtained in 97% ee. A racemic sample of **4e** was synthesized according to General Procedure **E**.

^{1}H NMR (700 MHz, $CDCl_3$) δ 7.34 – 7.29 (m, 4H), 7.25 – 7.19 (m, 2H), 7.20 – 7.15 (m, 4H), 4.16 (dt, *J* = 8.1, 4.1 Hz, 1H), 3.47 (d, *J* = 4.9 Hz, 1H), 2.75 – 2.67 (m, 1H), 2.67 – 2.54 (m, 3H), 2.46–2.36 (m, 2H), 1.96 (p, *J* = 7.4 Hz, 2H), 1.87 – 1.77 (m, 2H), 1.70 – 1.61 (m, 1H), 1.57 – 1.46 (m, 1H).
^{13}C NMR (176 MHz, $CDCl_3$) δ 211.9, 141.7, 141.1, 128.5, 128.4, 128.4, 128.4, 126.1, 125.9, 76.2, 36.9, 35.4, 35.0, 33.0, 26.4, 24.9.
$[\alpha]_D$: +34.6 (c = 1.2, T = 25 °C, $CHCl_3$)

NMR in agreement with the previously reported data for racemic **4e**[33]

Chiral SFC Analysis: CHIRALPAK IG (CO_2: MeOH, 80:20, 2.5 mL min^{-1}, 40 °C, 209 nm) t_R = 6.94 (major), 11.13 (minor) minutes, 97% ee

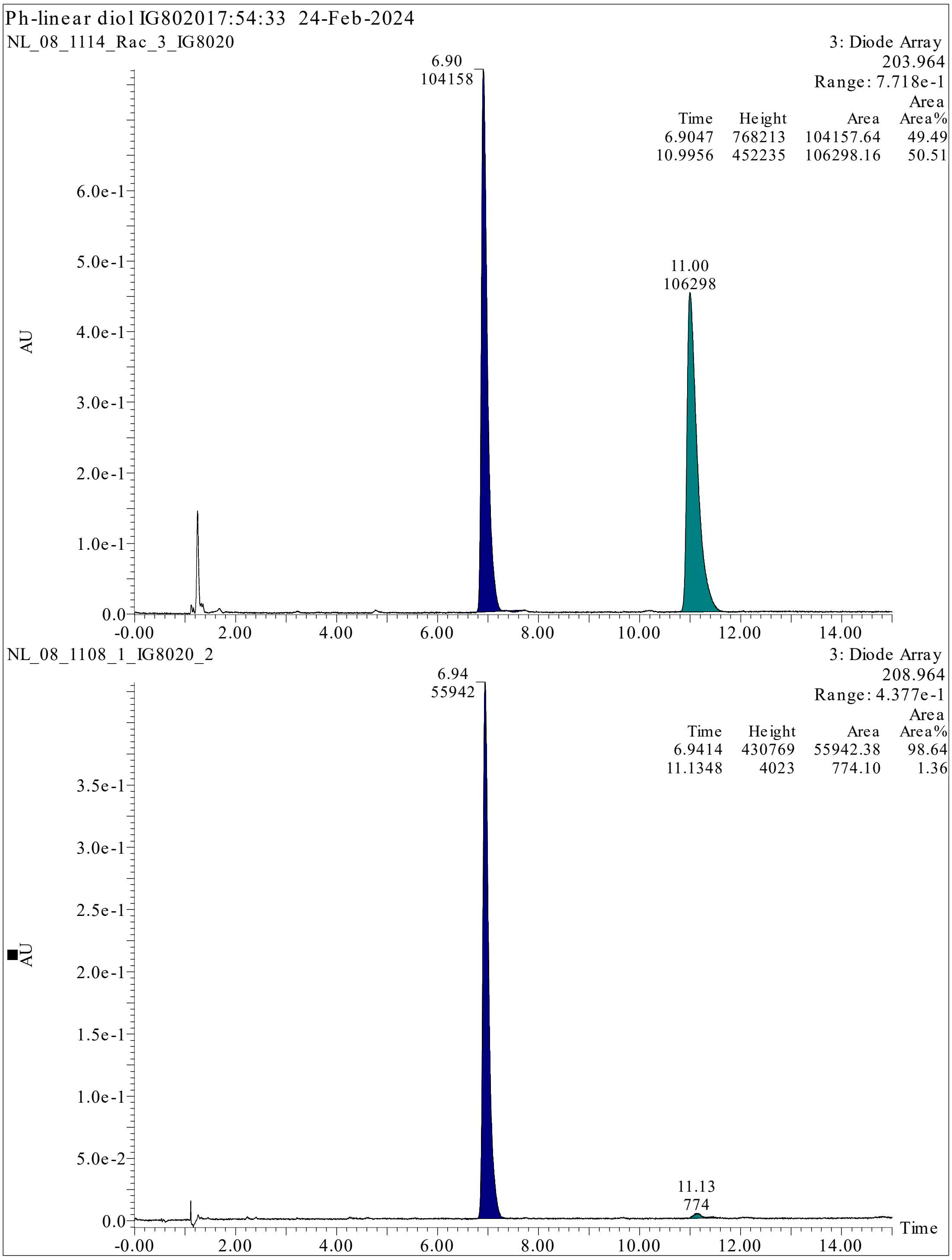

Di-*tert*-butyl (*S*)-4-hydroxy-5-oxooctanedioate (4f)

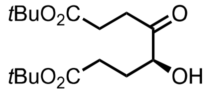

Di-*tert*-butyl (4*R*,5*S*)-4,5-dihydroxyoctanedioate **3f** (31.8 mg, 0.10 mmol) was subjected to General Procedure **B**, with 4CzIPN (3.9 mg, 0.005 mmol), *epi*-NHBoc-DHCN (4.0 mg, 0.010 mmol), TBA·H_2PO_4 (8.5 mg, 0.025 mmol), DIAD (20 mg, 0.10 mmol) in MeCN (4 mL) and reacted for 24 h. Purification by column chromatography (SiO_2, 10% EtOAc/hexanes) afforded **4f** as a white solid (11.0 mg, 0.035 mmol, 35%).

^{1}H NMR (700 MHz, $CDCl_3$) δ 4.25 (dt, *J* = 8.4, 4.2 Hz, 1H), 3.52 (d, *J* = 4.8, 1H), 2.82 (ddd, *J* = 18.0, 7.1, 5.7 Hz, 1H), 2.72 (ddd, *J* = 18.0, 7.3, 5.8 Hz, 1H), 2.65 – 2.52 (m, 2H), 2.45 (dt, *J* = 16.9, 7.5 Hz, 1H), 2.36 (ddd, *J* = 16.8, 7.8, 6.0 Hz, 1H), 2.20 (dtd, *J* = 14.8, 7.6, 3.7 Hz, 1H), 1.81 – 1.74 (m, 1H), 1.45 (s, 9H), 1.43 (s, 9H) .
^{13}C NMR (176 MHz, $CDCl_3$) δ 210.6, 172.7, 171.6, 80.9, 80.7, 75.7, 32.8, 30.7, 29.2, 28.8, 28.1, 28.1.
[α]$_D$: +12.8 (c = 0.73, T = 25 °C, $CHCl_3$)
HRMS (ESI+) $[M+Na]^+$ m/z calc'd for $[C_{16}H_{28}O_6Na]^+$ = 339.1784, found = 339.1790

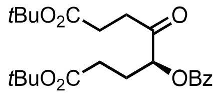

Derivatization for enantiomeric excess (ee) determination (**Bz-4f**)
Bz-4f was prepared via General Procedure **C1**. Purified by preparative thin layer chromatography (SiO_2, 10% EtOAc/CH_2Cl_2) afforded an analytical sample of **Bz-4f** for ee determination. Chiral SFC analysis indicated that **4f** was formed in 84% ee. A sample of racemic **Bz-4f** was synthesized *via* General Procedure **D1**.

^{1}H NMR (700 MHz, $CDCl_3$) δ 8.08 (d, *J* = 6.9 Hz, 2H), 7.59 (t, *J* = 7.5 Hz, 1H), 7.46 (t, *J* = 7.8 Hz, 2H), 5.33 (dd, *J* = 8.4, 4.3 Hz, 1H), 2.87 (dt, *J* = 18.6, 6.5 Hz, 1H), 2.81 (ddd, *J* = 18.7, 7.3, 6.1 Hz, 1H), 2.58 – 2.48 (m, 2H), 2.46 – 2.41 (m, 2H), 2.32 (ddd, *J* = 14.6, 7.7, 4.3 Hz, 1H), 2.17 (ddd, *J* = 15.0, 8.2, 6.8 Hz, 1H), 1.43 (s, 9H), 1.43 (s, 9H).
^{13}C NMR (176 MHz, $CDCl_3$) δ 205.6, 171.8, 171.8, 166.1, 133.6, 130.0, 129.4, 128.7, 81.0, 80.8, 77.8, 33.9, 31.0, 28.8, 28.23, 28.18, 26.0.
HRMS (ESI+) $[M+Na]^+$ m/z calc'd for $[C_{23}H_{32}O_7Na]^+$ = 443.2040, found = 443.2043

Chiral SFC Analysis: CHIRALPAK IG (CO_2: MeOH, 95:5, 2.5 mL min^{-1}, 40 °C, 231 nm) t_R = 5.02 (minor), 8.93 (major) minutes, 84% ee.

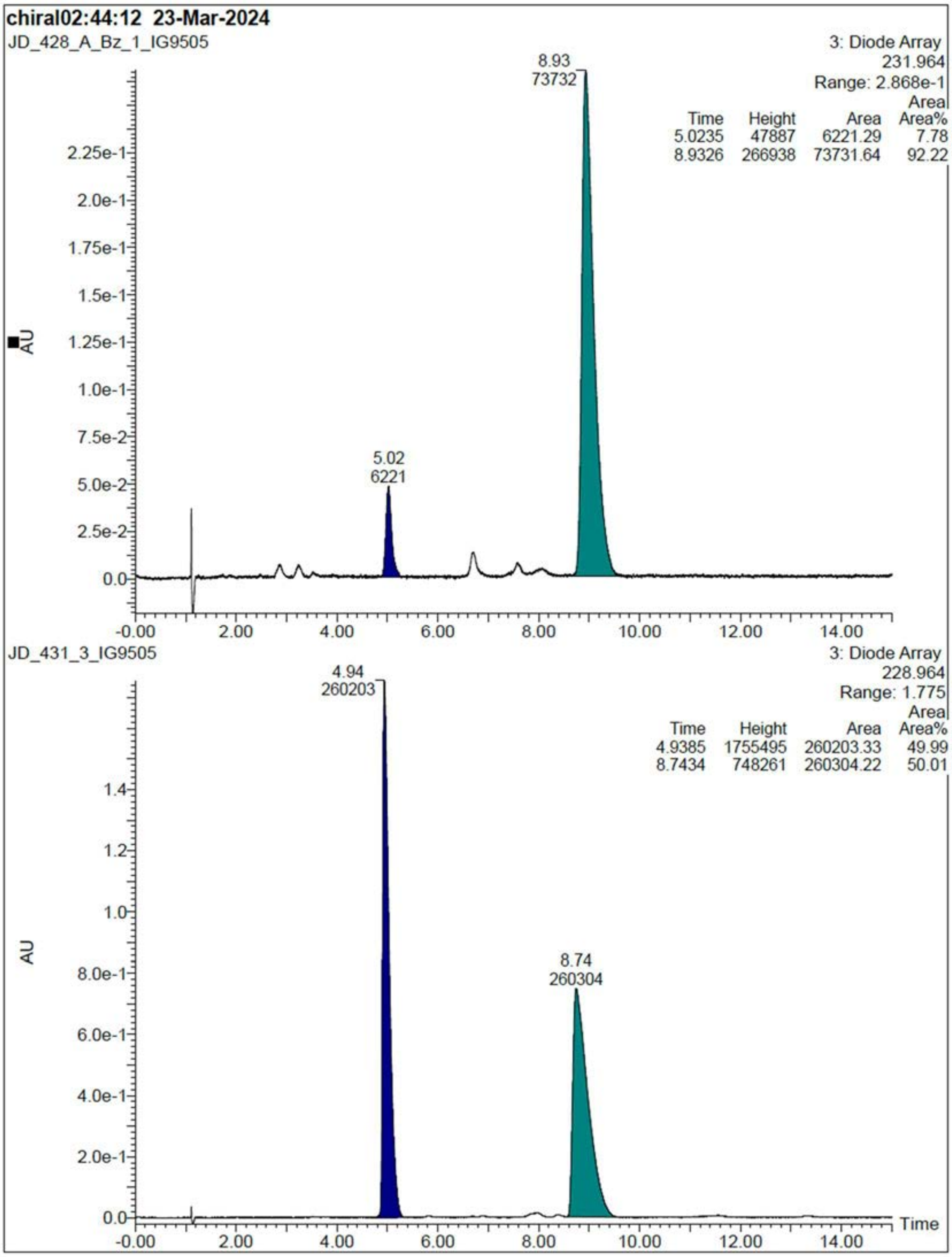

(*S*)-9-hydroxy-2,2,15,15-tetramethyl-3,3,14,14-tetraphenyl-4,13-dioxa-3,14-disilahexadecan-8-one (4g)

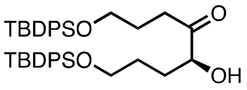

(8*R*,9*S*)-2,2,15,15-tetramethyl-3,3,14,14-tetraphenyl-4,13-dioxa-3,14-disilahexadecane-8,9-diol **3g** (65 mg, 0.1 mmol, 1 eq) was subjected General Procedure **B** with *epi*-NHBoc-DHCN (4.0 mg, 0.01 mmol, 10 mol%), 4CzIPN (4.0 mg, 0.005 mmol, 5 mol%), $TBAH_2PO_4$ (8.5 mg, 0.025 mmol, 25 mol%) and DIAD (20 mg, 0.1 mmol, 1 eq) in MeCN (4 ml) and reacted for 24 h. The crude mixture was purified by column chromatography (SiO_2, 5-10% acetone/hexanes) to obtain a crude product that contains the reduced H_2-DIAD impurity. This material was repurified by column chromatography (SiO_2, CH_2Cl_2) to yield the title compound **4g** (14.0 mg, 0.021 mmol, 21% yield, 94% ee) as a colourless oil. A sample of racemic **4g** was analogously synthesized *via* General Procedure **B** using quinuclidine (10 mol%) as the HAA catalyst

^{1}H NMR (700 MHz, $CDCl_3$) δ 7.69–7.61 (m, 8H), 7.43–7.39 (m, 4H), 7.38–7.33 (m, 8H), 4.17 (dt, *J* = 7.9, 4.1 Hz, 1H), 3.68 (dt, *J* = 16.6, 6.1 Hz, 4H), 3.54 (d, *J* = 4.8 Hz, 1H), 2.64–2.50 (m, 2H), 2.00–1.92 (m, 1H), 1.91–1.81 (m, 2H), 1.74–1.67 (m, 1H), 1.66–1.54 (m, 2H), 1.04 (s, 9H), 1.04 (s, 9H).
^{13}C NMR (176 MHz, $CDCl_3$) δ 212.4, 135.7, 135.7, 133.9, 133.8, 129.8, 129.8, 127.8, 127.8, 76.4, 63.6, 63.0, 34.4, 30.5, 28.0, 27.0, 26.6, 19.4, 19.3.
$[\alpha]_D$: +10.5 (c = 0.47, T = 25 °C, $CHCl_3$)
HRMS (ESI+) $[M+H]^+$ m/z calc'd for $[C_{40}H_{52}O_4Si_2H]^+$ = 653.3477, found = 653.3468

Chiral SFC Analysis: CHIRALPAK IK (CO_2: MeOH, 96:4, 1.25 mL min^{-1}, 40 °C, 202 nm) t_R = 28.70 (minor), 29.74 (major) minutes, 94% ee.

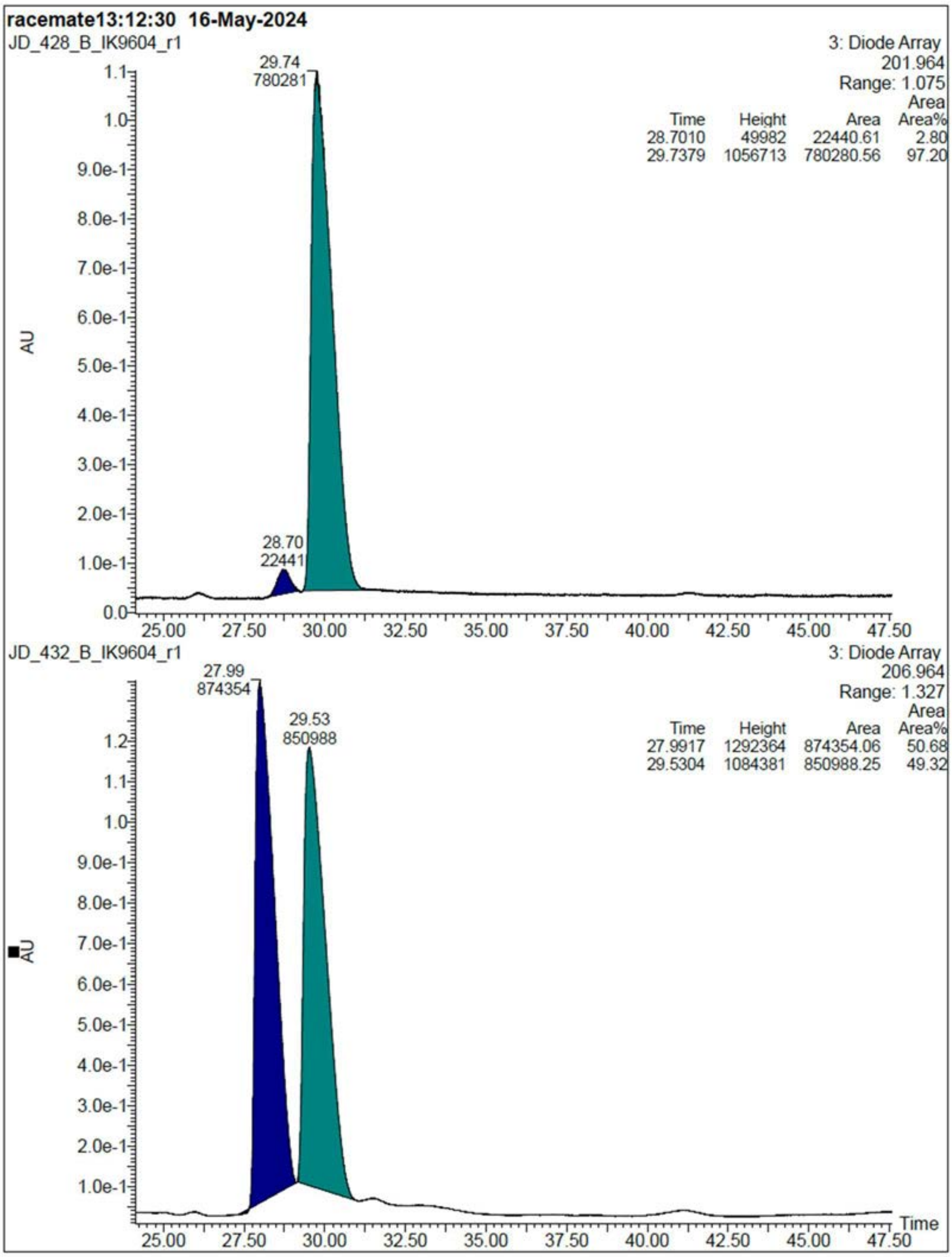

(*S*)-8-hydroxy-2,2,3,3,12,12,13,13-octamethyl-4,11-dioxa-3,12-disilatetradecan-7-one (4h)

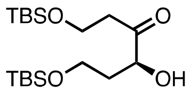

(7*R*,8*S*)-2,2,3,3,12,12,13,13-octamethyl-4,11-dioxa-3,12-disilatetradecane-7,8-diol **3h** (37.9 mg, 0.10 mmol) was subjected to General Procedure **B**, with 4CzIPN (3.9 mg, 0.005 mmol), *epi*-NHBoc-DHCN (4.0 mg, 0.010 mmol), TBA·H_2PO_4 (8.5 mg, 0.025 mmol), DIAD (20 mg, 0.10 mmol) in MeCN (4 mL) and reacted for 24 h. Purification by column chromatography (SiO_2, 20–100% CH_2Cl_2/hexanes) afforded **4h** as a clear oil (8.2 mg, 0.022 mmol, 22%).

^{1}H NMR (700 MHz, $CDCl_3$) δ 4.29 – 4.20 (m, 1H), 3.96 – 3.85 (m, 2H), 3.83 – 3.79 (m, 2H), 3.75 (dt, *J* = 10.1, 5.4 Hz, 1H), 2.75 (t, *J* = 6.3 Hz, 2H), 2.05 (dq, *J* = 14.5, 5.0, 4.4 Hz, 1H), 1.88 – 1.81 (m, 1H), 1.34 – 1.24 (m, 1H), 0.88 (s, 9H), 0.87 (s, 9H), 0.06–0.04 (m, 12H).
^{13}C NMR (176 MHz, $CDCl_3$) δ 211.5, 75.1, 59.1, 58.9, 41.1, 35.6, 25.9, 25.8, 18.3, 18.2, -5.5, –5.5, -5.6, -5.6.
$[\alpha]_D$: +12.8 (c = 0.73, T = 25 °C, $CHCl_3$)
HRMS (ESI+) $[M+H]^+$ m/z calc'd for $[C_{18}H_{40}O_4Si_2H]^+$ = 377.2543, found = 377.2545

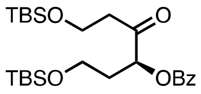

Derivatization for enantiomeric excess (ee) determination (**Bz-4h**)
Bz-4h was prepared via General Procedure **C1**. Purified by preparative thin layer chromatography (SiO_2, 10% EtOAc/hexanes) afforded an analytical sample of **Bz-4h** for ee determination. Chiral SFC analysis indicated that **4h** was formed in 85% ee. A sample of racemic **Bz-4h** was synthesized *via* General Procedure **D2**.

^{1}H NMR (700 MHz, $CDCl_3$) δ 8.14 – 8.04 (m, 2H), 7.66 – 7.57 (m, 1H), 7.53 – 7.41 (m, 2H), 5.40 (dd, *J* = 8.9, 3.8 Hz, 1H), 4.02 – 3.86 (m, 2H), 3.89 – 3.75 (m, 2H), 2.80 (td, *J* = 6.4, 1.8 Hz, 2H), 2.20 (dddd, *J* = 14.1, 8.0, 6.0, 3.8 Hz, 1H), 2.12 – 1.97 (m, 1H), 0.89 (s, 9H), 0.89 (s, 19H), 0.07 (s, 3H), 0.06 (s, 3H) 0.05 (s, 3H), 0.03 (s, 3H)
^{13}C NMR (176 MHz, $CDCl_3$) δ 205.8, 165.9, 133.3, 129.8, 129.5, 128.5, 76.2, 58.6, 58.1, 41.8, 33.1, 25.9, 25.9, 18.3, 18.3, -5.5 (2C), -5.5, -5.5.
HRMS (ESI+) $[M+H]^+$ m/z calc'd for $[C_{25}H_{44}O_5Si_2H]^+$ = 481.2806, found = 481.2805

Chiral SFC Analysis: CHIRALPAK IG (CO_2: MeOH, 97:3, 2.5 mL min^{-1}, 40 °C, 227 nm) t_R = 2.48 (minor), 2.92 (major) minutes. 85% ee

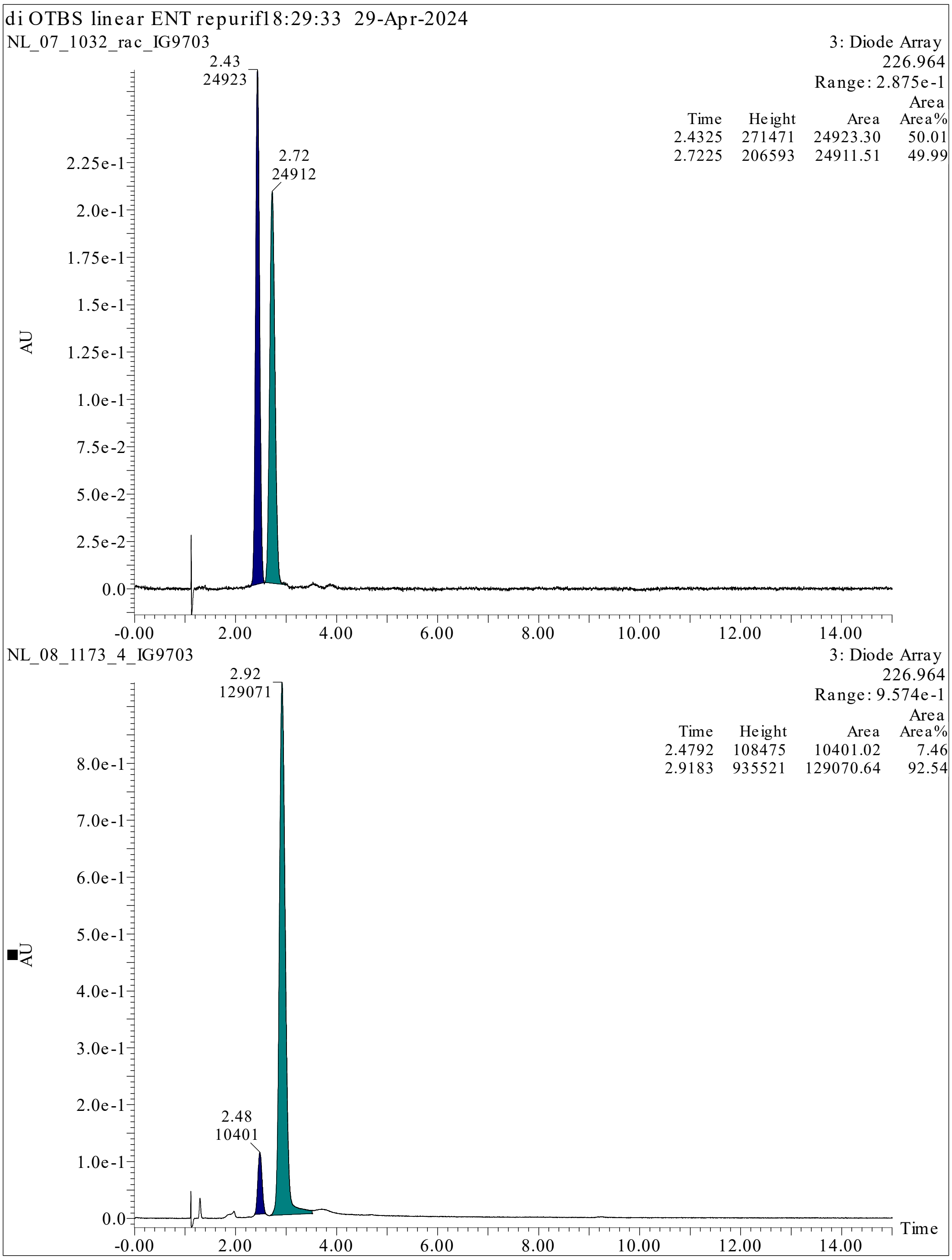

(*S*)-8-hydroxy-2,2,4,4,11,11,13,13-octamethyl-3,12-dioxa-2,13-disilatetradecan-7-one (4i)

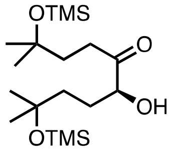

(7*R*,8*S*)-2,2,4,4,11,11,13,13-octamethyl-3,12-dioxa-2,13-disilatetradecane-7,8-diol **3i** (37.9 mg, 0.10 mmol) was subjected to General Procedure **B**, with 4CzIPN (3.9 mg, 0.005 mmol), *epi*-NHBoc-DHCN (4.0 mg, 0.010 mmol), TBA·H_2PO_4 (8.5 mg, 0.025 mmol), DIAD (20 mg, 0.10 mmol) in MeCN (4 mL) and reacted for 24 h. Purification by column chromatography (SiO_2, 5–10% EtOAc/hexanes) afforded **4i** as a clear oil (30.7 mg, 0.081 mmol, 81%).

^{1}H NMR (700 MHz, $CDCl_3$) δ 4.22 (dt, *J* = 8.2, 4.3 Hz, 1H), 3.61 (d, *J* = 4.7 Hz, 1H), 2.66 – 2.45 (m, 2H), 2.02 – 1.91 (m, 1H), 1.81 – 1.71 (m, 2H), 1.68 – 1.60 (m, 1H), 1.61 – 1.55 (m, 1H), 1.43 (td, *J* = 13.0, 12.4, 4.3 Hz, 1H), 1.24 (s, 3H), 1.23 (s, 6H), 1.22 (s, 3H), 0.11 (s, 9H), 0.10 (s, 9H).
^{13}C NMR (176 MHz, $CDCl_3$) δ 213.1, 76.7, 73.5, 73.0, 39.7, 38.2, 33.1, 29.9, 29.8, 29.8, 29.8, 28.5, 2.6, 2.5.
$[\alpha]_D$: +27.0 (c = 1.3, T = 25 °C, $CHCl_3$)
HRMS (ESI+) $[M+Na]^+$ m/z calc'd for $[C_{18}H_{40}O_4Si_2Na]^+$ = 399.2363, found = 399.2350

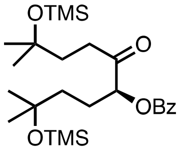

Derivatization for enantiomeric excess (ee) determination (**Bz-4i**)
Bz-4i was prepared via General Procedure **C1**. Purified by preparative thin layer chromatography (SiO_2, 10% EtOAc/hexanes) afforded an analytical sample of **Bz-4i** for ee determination. Chiral SFC analysis indicated that **4i** was formed in 95% ee. A sample of racemic **Bz-4i** was synthesized *via* General Procedure **D2**.

^{1}H NMR (700 MHz, $CDCl_3$) δ 8.11 (dd, *J* = 8.3, 1.2 Hz, 2H), 7.65 – 7.57 (m, 1H), 7.49 (t, *J* = 7.8 Hz, 2H), 5.34 – 5.26 (m, 1H), 2.70 (ddd, *J* = 17.6, 10.1, 5.8 Hz, 1H), 2.62 (ddd, *J* = 17.6, 9.9, 5.7 Hz, 1H), 2.07 (dddd, *J* = 16.7, 10.2, 6.4, 4.3 Hz, 1H), 2.02 – 1.94 (m, 1H), 1.82 – 1.71 (m, 2H), 1.65 – 1.57 (m, 2H), 1.27 (s, 3H), 1.27 (s, 3H), 1.24 (s, 3H), 1.23 (s, 3H), 0.13 (s, 9H), 0.09 (s, 9H).
^{13}C NMR (176 MHz, $CDCl_3$) δ 207.9, 166.1, 133.3, 129.8, 129.6, 128.5, 79.3, 73.3, 73.0, 40.1, 37.5, 34.0, 30.0, 29.9, 29.8, 29.7, 25.8, 2.6, 2.5.
HRMS (ESI+) $[M+Na]^+$ m/z calc'd for $[C_{25}H_{44}O_5Si_2Na]^+$ = 503.2625, found = 503.2615

Chiral SFC Analysis: CHIRALPAK IG (CO_2: MeOH, 97:3, 2.5 mL min^{-1}, 40 °C, 227 nm) t_R = 4.87 (minor), 5.40 (major) minutes. 95% ee

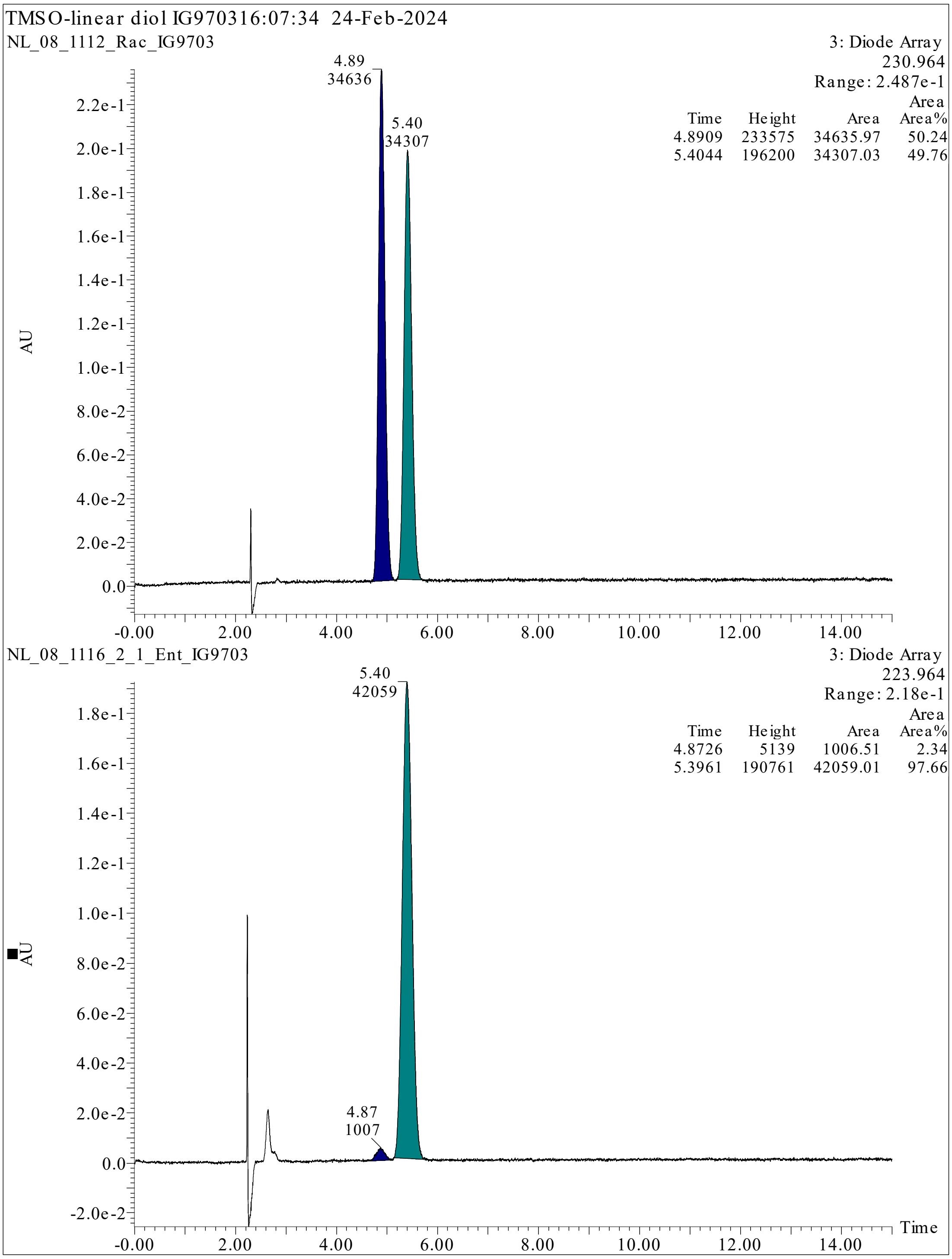

(*S*,2*E*,10*E*)-6-hydroxy-7-oxododeca-2,10-diene-1,12-diyl bis(2,2-dimethylpropanoate) (4k)

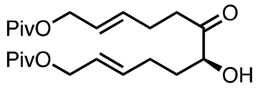

(2*E*,6*R*,7*S*,10*E*)-6,7-dihydroxydodeca-2,10-diene-1,12-diyl bis(2,2-dimethylpropanoate) **3k** (39.9 mg, 0.10 mmol) was subjected to General Procedure **B**, with 4CzIPN (3.9 mg, 0.005 mmol), *epi*-NHBoc-DHCN (4.0 mg, 0.010 mmol), TBA·H_2PO_4 (8.5 mg, 0.025 mmol), DIAD (20 mg, 0.10 mmol) in MeCN (4 mL) and reacted for 24 h. Purification by column chromatography (SiO_2, CH_2Cl_2 *then* 10% EtOAc/hexanes) afforded **4k** as a clear oil (13.6 mg, 0.034 mmol, 34%).

^{1}H NMR (700 MHz, $CDCl_3$) δ 5.77–5.70 (m, 2H), 5.63–5.58 (m, 2H), 4.54 – 4.44 (m, 4H), 4.15 (dt, *J* = 8.3, 4.0 Hz, 1H), 3.42 (d, *J* = 4.9 Hz, 1H), 2.61 (dt, *J* = 17.2, 7.3 Hz, 1H), 2.56–2.51 (m, 1H), 2.39 (q, *J* = 7.1 Hz, 2H), 2.24 (dq, *J* = 15.7, 7.3 Hz, 1H), 2.20 – 2.12 (m, 1H), 1.91 (dddd, *J* = 13.4, 9.9, 6.6, 3.5 Hz, 1H), 1.60 (ddt, *J* = 13.8, 8.7, 4.7 Hz, 3H), 1.20 (s, 9H), 1.20 (s, 9H).
^{13}C NMR (176 MHz, $CDCl_3$) δ 211.1, 178.3, 178.3, 133.6, 132.7, 125.8, 125.6, 75.8, 64.7, 64.5, 38.8, 37.1, 33.0, 27.7, 27.2 (2C), 26.1.
$[\alpha]_D$: +23.5 (c = 0.67, T = 25 °C, $CHCl_3$)
HRMS (ESI+) $[M+Na]^+$ m/z calc'd for $[C_{22}H_{36}O_6Na]^+$ = 419.2410, found = 419.2403

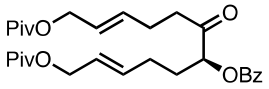

Derivatization for enantiomeric excess (ee) determination (**Bz-4k**)
Bz-4k was prepared via General Procedure **C1**. Purified by preparative thin layer chromatography (SiO_2, 2% EtOAc/CH_2Cl_2) afforded an analytical sample of **Bz-4k** for ee determination. Chiral SFC analysis indicated that **4k** was formed in 85% ee. A sample of racemic **Bz-4k** was synthesized *via* General Procedure **D2**.

^{1}H NMR (700 MHz, $CDCl_3$) δ 8.08 (d, *J* = 7.9 Hz, 2H), 7.61 (t, *J* = 7.1 Hz, 1H), 7.48 (t, *J* = 7.5 Hz, 2H), 5.73 (ddt, *J* = 21.8, 14.0, 6.6 Hz, 2H), 5.59 (tt, *J* = 13.8, 6.0 Hz, 2H), 5.25 (dd, *J* = 7.5, 5.2 Hz, 1H), 4.54 – 4.39 (m, 4H), 2.70 (dt, *J* = 17.6, 7.3 Hz, 1H), 2.56 (dt, *J* = 17.8, 7.2 Hz, 1H), 2.37 (s, 2H), 2.24 (d, *J* = 14.1 Hz, 2H), 2.06 – 1.90 (m, 2H), 1.19 (d, *J* = 2.5 Hz, 18H).
^{13}C NMR (176 MHz, $CDCl_3$) δ 206.5, 178.3, 166.0, 133.6, 133.0, 133.0, 129.8, 129.3, 128.6, 125.9, 125.5, 78.1, 64.6, 64.6, 38.7, 37.9, 29.9, 28.1, 27.2, 25.7.
HRMS (ESI+) $[M+Na]^+$ m/z calc'd for $[C_{29}H_{40}O_7Na]^+$ = 523.2672, found = 523.2671

Chiral SFC Analysis: CHIRALPAK IG (CO_2: MeOH, 88:12, 2.5 mL min^{-1}, 40 °C, 225 nm) t_R = 3.62 (minor), 5.28 (major) minutes. 85% ee

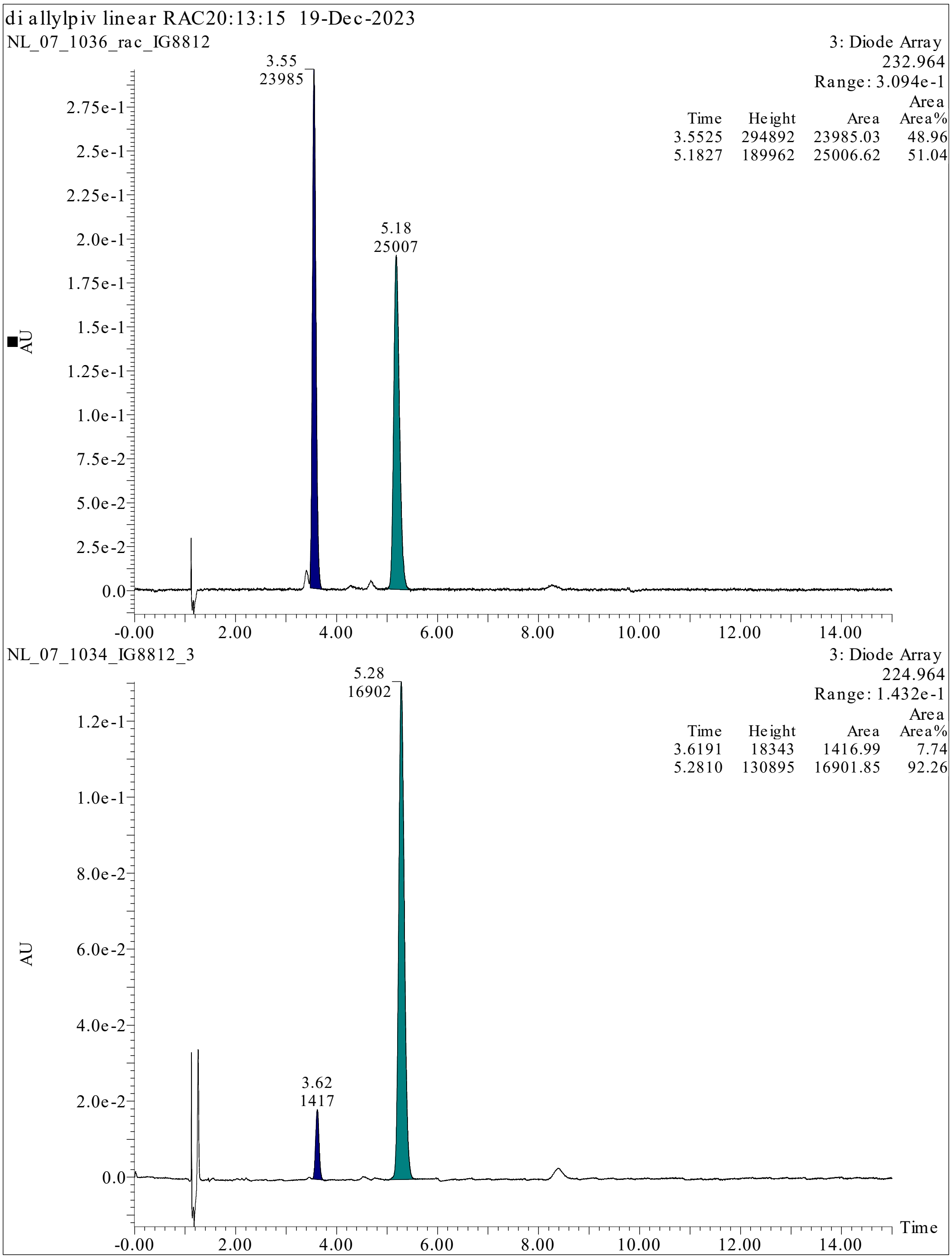

(*S*)-4-((*tert*-butyldiphenylsilyl)oxy)pentan-2-one (4l')

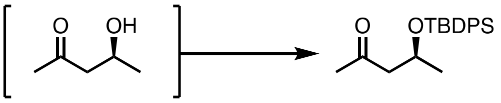

(2*R*,4*S*)-pentane-2,4-diol **3l** (10.4 mg, 0.10 mmol) was subjected to General Procedure **B**, with 4CzIPN (3.9 mg, 0.005 mmol), *epi*-NHBoc-DHCN (4.0 mg, 0.010 mmol), TBA·H_2PO_4 (8.5 mg, 0.025 mmol), DIAD (20 mg, 0.10 mmol) in MeCN (4 mL) and reacted for 24 h. Compound **4l** is highly volatile; at this stage, careful evaporation of the reaction mixture and dissolving the crude material in $CDCl_3$ with CH_2Br_2 as the internal standard facilitated crude NMR yield determination (86%, see below for NMR yield determination). This solution was carefully concentrated under reduced pressure, and filtered over a thin plug of SiO_2 eluting first with 1:1 CH_2Cl_2/hexane to remove the photocatalyst, and next with CH_2Cl_2 (*ca.* 2 mL) to give **4l**.

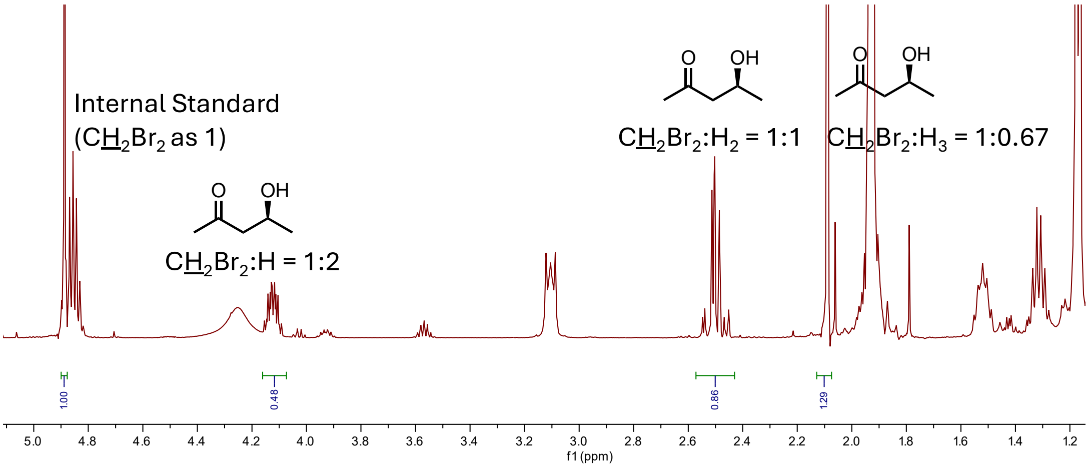

For preparative isolation, TBDPSCl (30 µL, 0.11 mmol) and imidazole (10.2 mg, 0.15 mmol) was added to the above CH_2Cl_2 solution. The reaction mixture was stirred for 4 h before concentrated under a stream of air. Purification by column chromatography (SiO_2, 0–5% EtOAc/hexanes) afforded the TBDPS ether **4l'** as a clear oil (20.6 mg, 0.066 mmol, 66%). Chiral SFC analysis indicated that **4l** was formed in 94% ee.

^{1}H NMR (700 MHz, $CDCl_3$) δ 7.71–7.68 (m, 4H), 7.48 – 7.43 (m, 2H), 7.43 – 7.37 (m, 4H), 4.33 (h, *J* = 6.1 Hz, 1H), 2.66 (dd, *J* = 15.2, 6.0 Hz, 1H), 2.50 (dd, *J* = 15.2, 6.4 Hz, 1H), 2.08 (s, 3H), 1.12 (d, *J* = 6.1 Hz, 3H), 1.06 (s, 9H).
^{13}C NMR (176 MHz, $CDCl_3$) δ 207.5, 135.8, 135.8, 134.3, 133.8, 129.7, 129.6, 127.6, 127.5, 66.5, 53.3, 31.1, 26.9, 23.7, 19.2.
[α]$_D$: +2.6 (c = 1.2, T = 25 °C, $CHCl_3$)
*Literature [α]$_D$ value for **R**-4l'*: –5.0 (c = 0.4; T = 20 °C $CHCl_3$).[34]
Absolute configuration of 4l *therefore assigned as **S***

NMR in agreement with the previously reported data for *R*- **4l'**[34]

Chiral SFC Analysis: CHIRALPAK IA (CO_2: MeOH, 99:1, 1.25 mL min^{-1}, 40 °C, 213 nm) t_R = 10.56 (minor), 11.01 (major) minutes. 94% ee

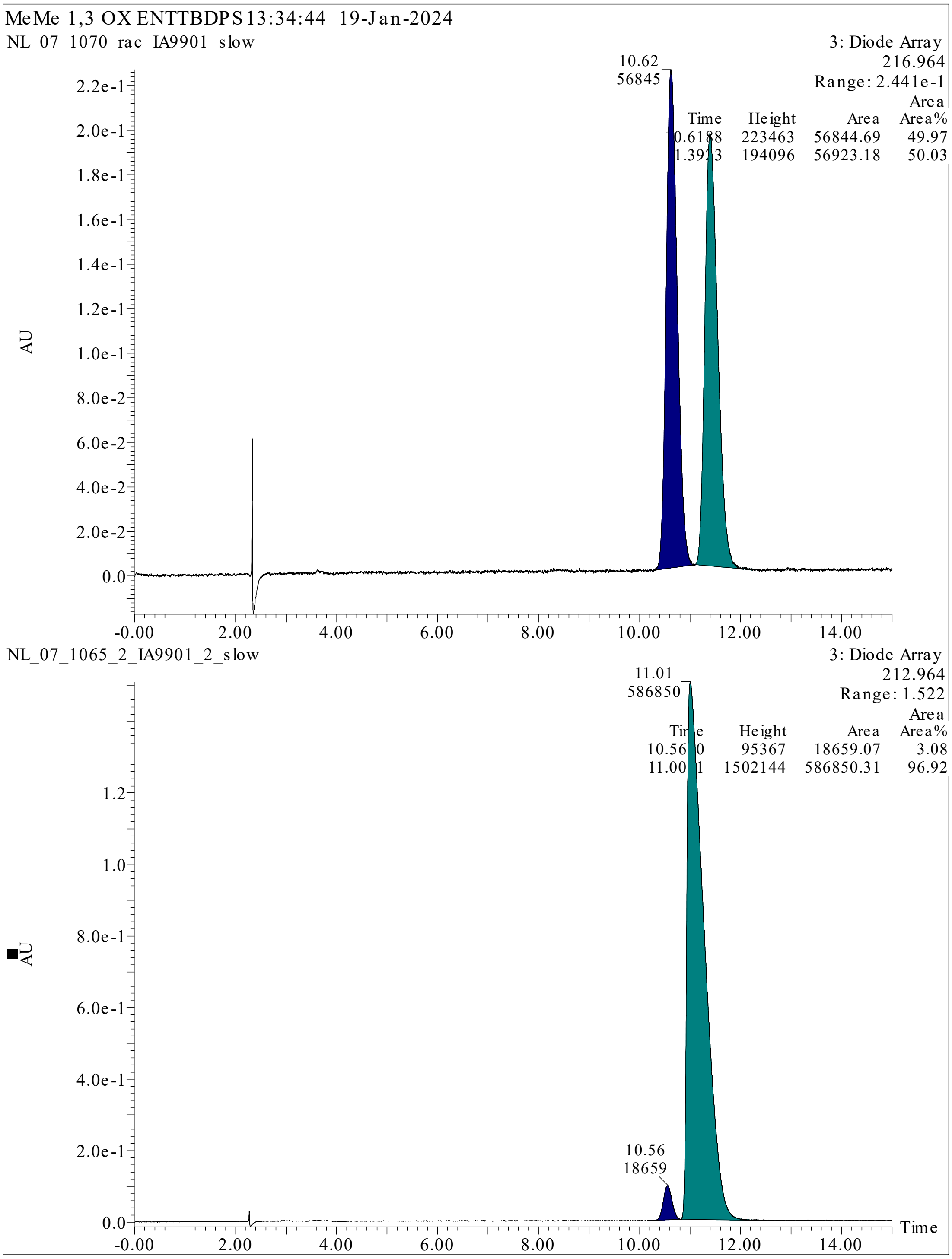

4.2. Absolute stereochemistry assignment

Eight of the hydroxyketones synthesized using our standard conditions (*epi*-NHBoc-DHCN) are known compounds, with recorded $[\alpha]_D$ values. Comparison of the optical rotation sign for all eight compounds consistently indicate that the major enantiomer formed by our enantioselective oxidation with *epi*-NHBoc-DHCN is *S* configured consistent with what was observed for the previously reported enantioselective isomerisation process. This gives confidence that our assigned *S*-configuration for for all novel hydroxyketone products is likely correct.

Structure	$[\alpha]_D$ (from *epi*-NHBoc-DHCN)	Literature $[\alpha]_D$
**2a** 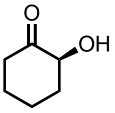	*S*-enantiomer deduced<br>**–13.2** (c = 0.9, T = 25 °C, $CHCl_3$)	*S*-enantiomer reported<br>**–18.8** (c = 0.65; T = 22 °C $CHCl_3$).<br>*J. Am. Chem. Soc.,* **2016***, 138,* 16839–16848
**2b** 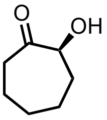	*S*-enantiomer deduced<br>**+93.9** (c = 0.54, T = 25 °C, $CHCl_3$)	*R*-enantiomer reported<br>**–86.4** (c = 0.65; T = 28 °C $CHCl_3$)<br>*Adv. Synth. Catal.*, **2013**, *355*, 3147–3153
**2c** 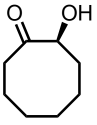	*S*-enantiomer deduced<br>**+50.4** (c = 0.59, T = 25 °C, $CHCl_3$).	*R*-enantiomer reported<br>**–36.5** (c = 1; T = 28 °C $CHCl_3$).<br>*Adv. Synth. Catal.*, **2013**, *355*, 3147–3153
**2d** 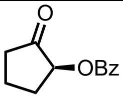	*S*-enantiomer deduced<br>**+50.2** (c = 0.9, T = 25 °C, $CHCl_3$).	*S*-enantiomer reported<br>**+38.4** (c = 1.0; T = 22 °C, $CHCl_3$).<br>*J. Am. Chem. Soc.*, **2016**, *138*, 16839 –16848
**4a** 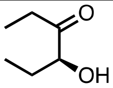	*S*-enantiomer deduced<br>**+88.8** (c = 0.42, T = 25 °C, $CHCl_3$)	*S*-enantiomer reported<br>**+97.5** (c = 1.0; T = 22 °C $CHCl_3$).<br>*Helv. Chim. Act.,* **1989**. *72,* 980 -984
**4b** 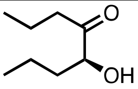	*S*-enantiomer deduced<br>**+53.9** (c = 0.56, T = 25 °C, $CHCl_3$)	*R*-enantiomer reported<br>**–13.8** (c = 0.5; T = 20 °C $CHCl_3$).<br>*Tetrahedron Asym.*, **2007**, *18*, 12, 1465–1474
**4c** 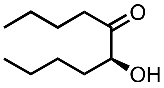	*S*-enantiomer deduced<br>**+62.1** (c = 0.73, T = 25 °C, $CHCl_3$)	*S*-enantiomer reported<br>**+9.0** (c = 0.63; T = 20 °C, MeOH).<br>*J. Org. Chem.*, **2009**. *74* 8377–8380
**4d** 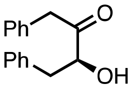	*S*-enantiomer deduced<br>**+14.6** (c = 0.27, T = 25 °C, $CHCl_3$)	*S*-enantiomer reported<br>**+75.9** (c = 0.62; T = 25 °C $CHCl_3$).<br>*J Am Chem Soc* **2003**, *125* (11), 3220–3221.
**4l’** 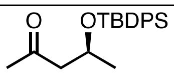	*S*-enantiomer deduced<br>**+2.6** (c = 1.2, T = 25 °C, $CHCl_3$)	*R*-enantiomer reported<br>**–5.0** (c = 0.4; T = 20 °C $CHCl_3$).<br>*Tetrahedron Asym.*, **2003**,14, *22*, 3619 - 3625

Additionally, the use of the pseudoenantiomeric catalysts (*epi*-NHBoc-DHCD) gives the antipodal product—data of which corroborated by both chiral SFC analysis as well as sign comparison for optical rotation data.

4.3. Enantioselective oxidation with O_2

Screen of reaction parameters: O_2 as oxidant

For all examples, a static amount of O_2 was used, which was introduced to the reaction system by purging the reaction mixture with an O_2 balloon for 15 minutes.

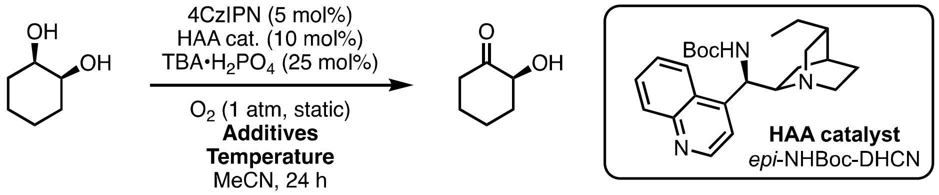

Entry	Oxidant	Changes to condition	Product % (SM %)	ee (%)
1.	O_2	T = +10 °C	20% (77%)	81%
2.	O_2	T = +10 °C; Omit TBA H_2PO_4	0% (58%)	-
3.	O_2	**T = –35 °C;** C = 0.025 M (standard concentration)	40% (20%)	87%
**4.**	**O_2**	**T = –35 °C; C = 0.0125 M** (0.5x relative to entry 3)	**52% (6%)** **50% isolated**	**92%**
5.	O_2	T = –35 °C + 4 Å MS (20 mg)	43% (20%)	-
6.	O_2	T = –35 °C + $MgSO_4$ (20 mg)	32% (25%)	-
7.	O_2	T = –35 °C+ ZnO (20 mg) *to sequester H_2O_2*	28% (54%)	-
8.	O_2	T = –35 °C + TiO_2 (20 mg) *to adsorb O_2*	26% (55%)	-
9.	O_2	T = –35 °C + MnO_2 (20 mg) *to break down H_2O_2*	21% (43%)	-
10.	O_2	T = –35 °C + SiO_2 (20 mg)	36% (32%)	-
11.	O_2	T = –35 °C + Al_2O_3 (20 mg)	25% (60%)	-
12.	O_2	T = –35 °C + dodecanethiol (25 mol%)	32% (43%)	-

Part of the consideration for further diluting the reaction mixture is the recognition that a greater solvent volume would solubilize a greater amount of the O_2 (Henry's Law). Addition of additives hindered reaction efficacy. Overall, O_2 is a less capable/general/efficacious oxidant than DIAD for our present transformation in a batch context; examples shown below in Figure S9.

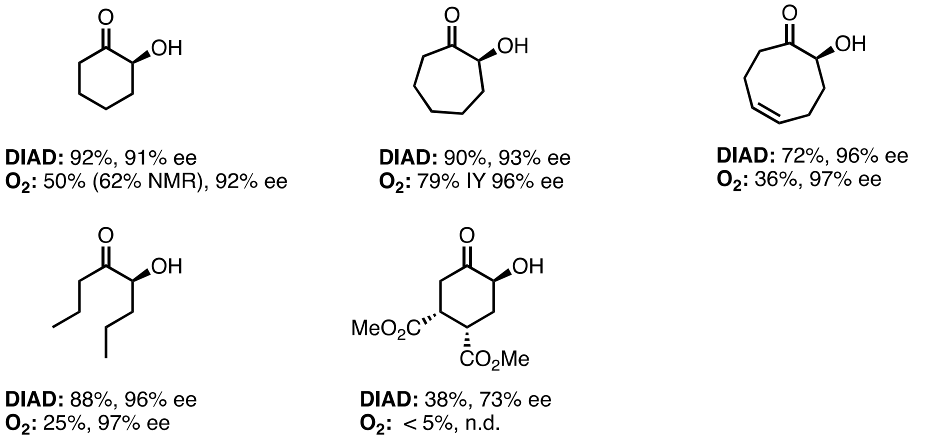

Figure S9. Reaction efficacy comparison between DIAD and O_2-mediated oxidation

General Procedure F: Catalytic enantioselective oxidation: O_2 as oxidant

Enantioselective oxidation reactions with O_2 were carried out in 20 mL (6 dram) snap cap vials

Meso diol substrate (0.1 mmol), HAA catalyst (4.0 mg, 0.01 mmol, 10 mol%), 4CzIPN (3.9 mg, 0.005 mmol, 5 mol%) and TBA H_2PO_4 (8.5 mg, 0.025 mmol, 25 mol%) was added in a 6 dram vial equipped with a stirrer bar. Dry MeCN (8 mL) was next added, and the vial was sealed with a B19 septa and further sealed with Parafilm. The sealed reaction vial was directly placed in the cooling bath. O_2 (introduced *via* a balloon through a needle) was next bubbled through the reaction mixture at –35 °C for 30 min. The O_2 balloon was removed, the lamp was next switched on, and the reaction was stirred with photoirradiation at –35 °C. Upon completion, the reaction was analogously worked up and purified according to **General Procedure B** to afford the enantioenriched hydroxyketone, and derivatized as described to obtain ee values. All characterization data are in accordance with that obtained from DIAD oxidation (*vide supra*).

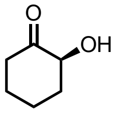

2a

1a (11.6 mg, 0.10 mmol) was subjected to General Procedure **F** and reacted for 24 h. Purification by column chromatography (SiO_2, 50–100% CH_2Cl_2/hexane) afforded **2a** (5.6 mg, 0.050 mmol, 50%) as a clear oil. Derivation to **Bz-2a** indicated **2a** was formed in 93% ee

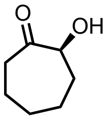

2b

1b (11.6 mg, 0.10 mmol) was subjected to General Procedure **F** and reacted for 24 h. Purification by column chromatography (SiO_2, 50–100% CH_2Cl_2/hexane) afforded **2b** (10.1 mg, 0.079 mmol, 79%) as a clear oil. Derivation to **Bz-2b** indicated **2b** was formed in 95% ee

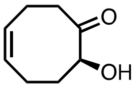

2l

1l (14.2 mg, 0.10 mmol) was subjected to General Procedure **F** and reacted for 36 h. Purification by column chromatography (SiO_2, 50–100% CH_2Cl_2/hexane) afforded **2l** (5.0 mg, 0.036 mmol, 36%, 97% ee) as a clear oil. Derivation to **Bz-2l** indicated **2l** was formed in 97% ee

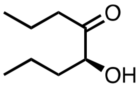

4b

3b (11.6 mg, 0.10 mmol) was subjected to General Procedure **F** and reacted for 24 h. Purification by column chromatography (SiO_2, 50–100% CH_2Cl_2/hexane) afforded **4b** (3.6 mg, 0.025 mmol, 25%) as a clear oil. Derivation to **Bz-4b** indicated **4b** was formed in 97% ee

Chiral SFC Analysis (Bz-2a with O_2) CHIRALPAK IC (CO_2: MeOH, 90:10, 2.5 mL min^{-1}, 40 °C, 226 nm) t_R = 3.82 (minor), 5.06 (major) minutes, 93% ee

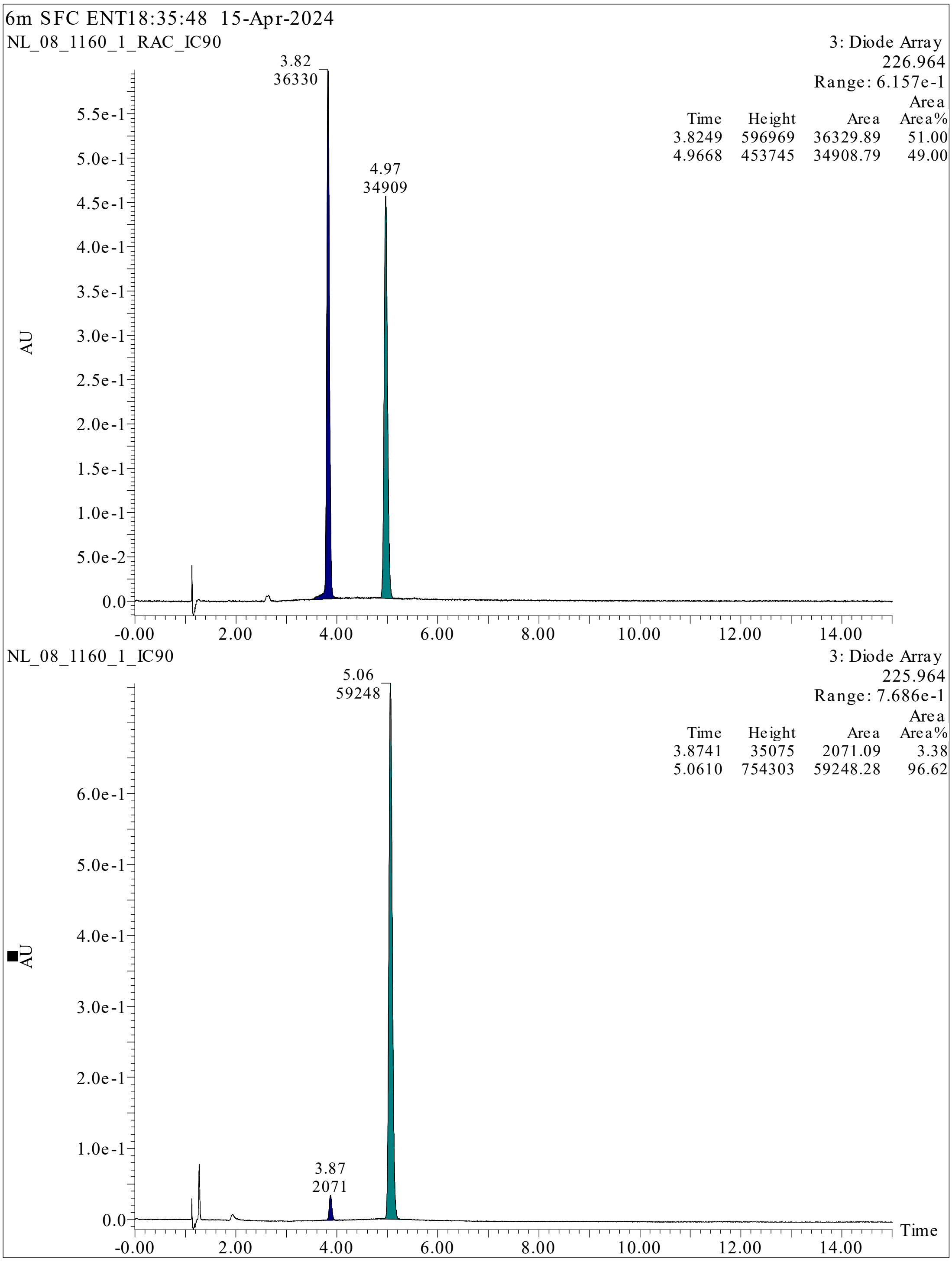

Chiral SFC Analysis (Bz-2b with O_2): CHIRALPAK IC (CO_2: MeOH, 91:9 2.5 mL min^{-1}, 40 °C, 227 nm) t_R = 4.76 (minor), 5.53 (major) minutes, 95% ee

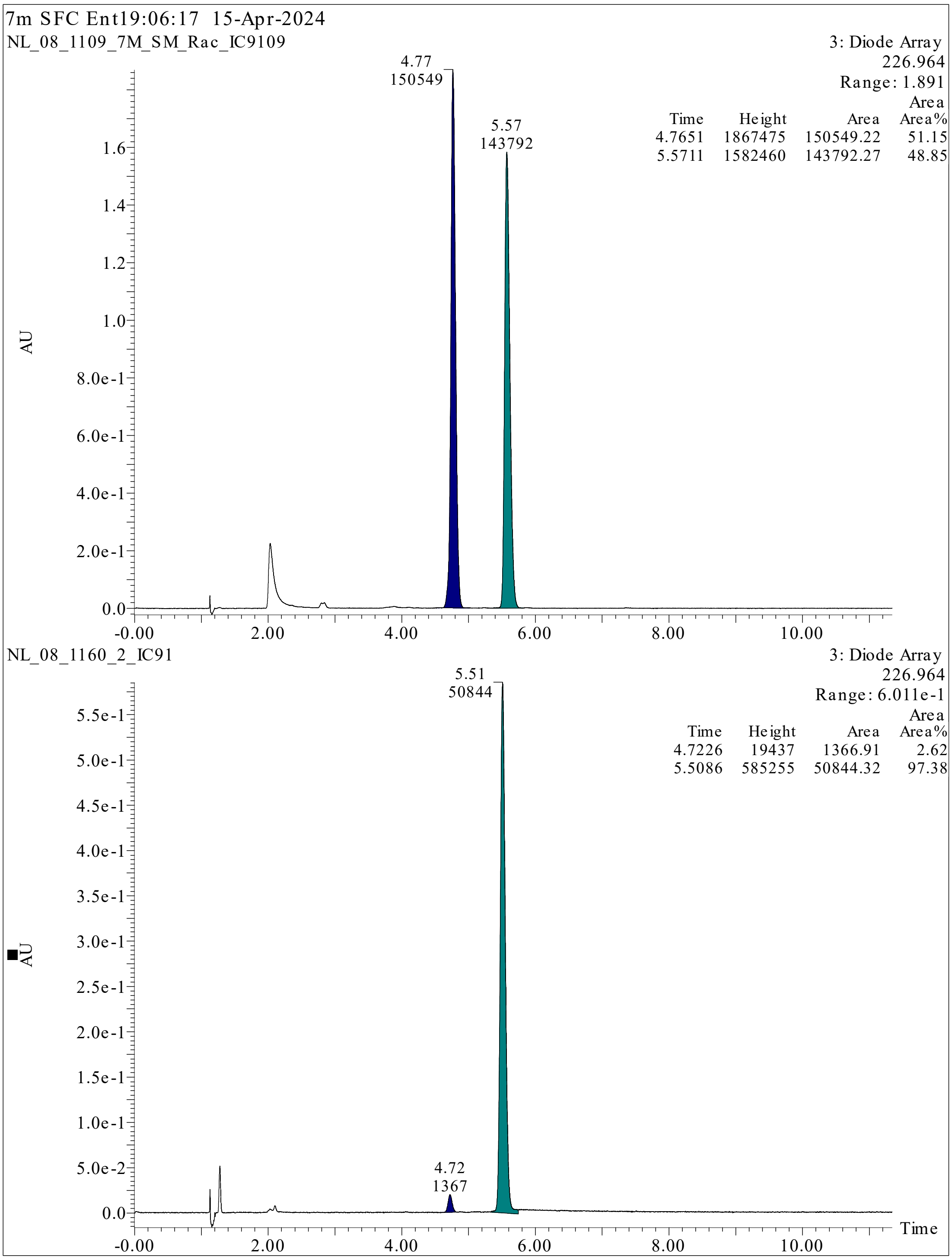

Chiral SFC Analysis (Bz-2l with O_2): CHIRALPAK IG (CO_2: MeOH, 90:10, 2.5 mL min^{-1}, 40 °C, 228 nm) t_R = 7.96 (major), 9.36 (minor) minutes, 98% ee

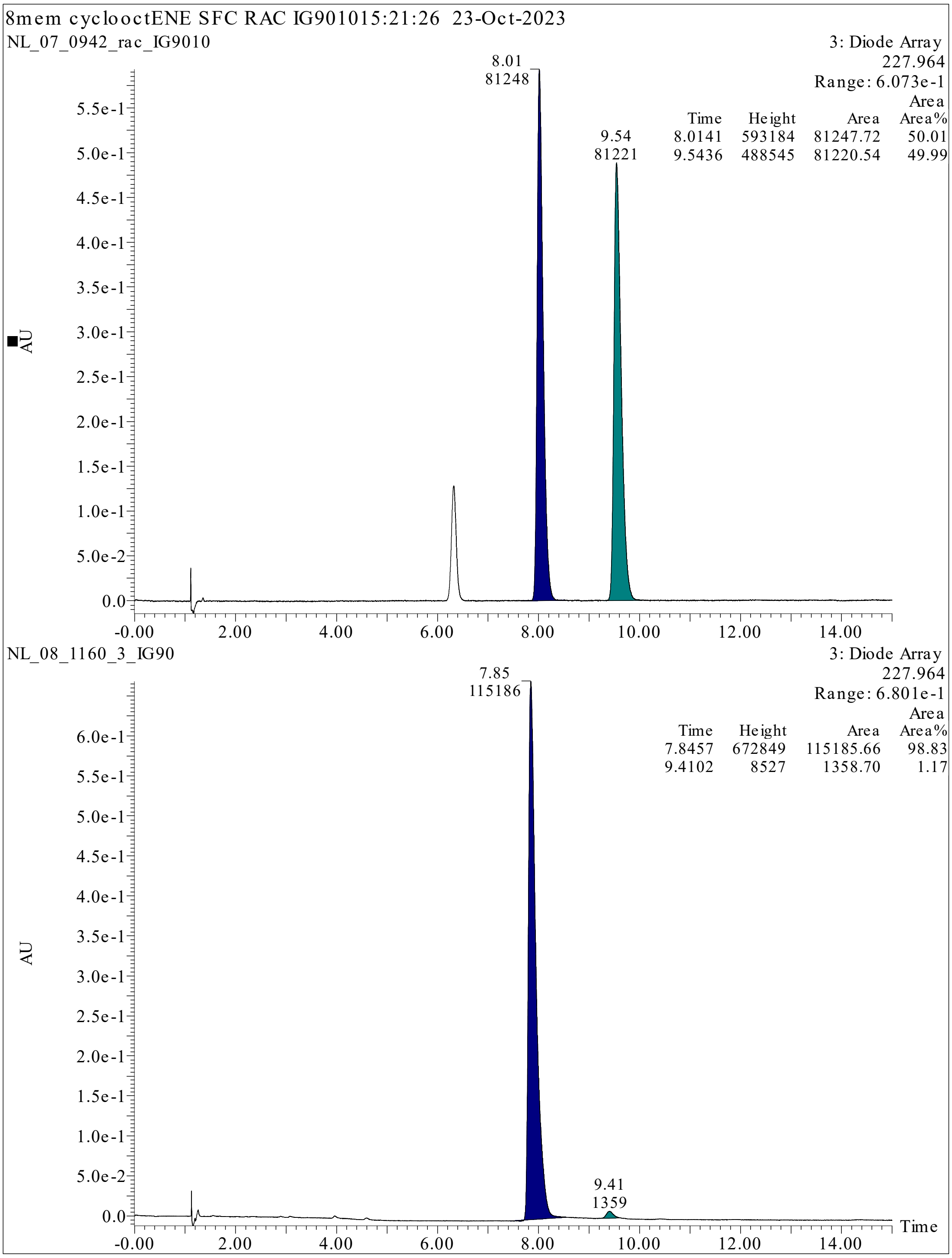

Chiral SFC Analysis (Bz-4b with O_2): CHIRALPAK IG (CO_2: MeOH, 98:2, 2.5 mL min^{-1}, 40 °C, 225 nm) t_R = 4.83 (minor), 7.16 (major) minutes, 96% ee

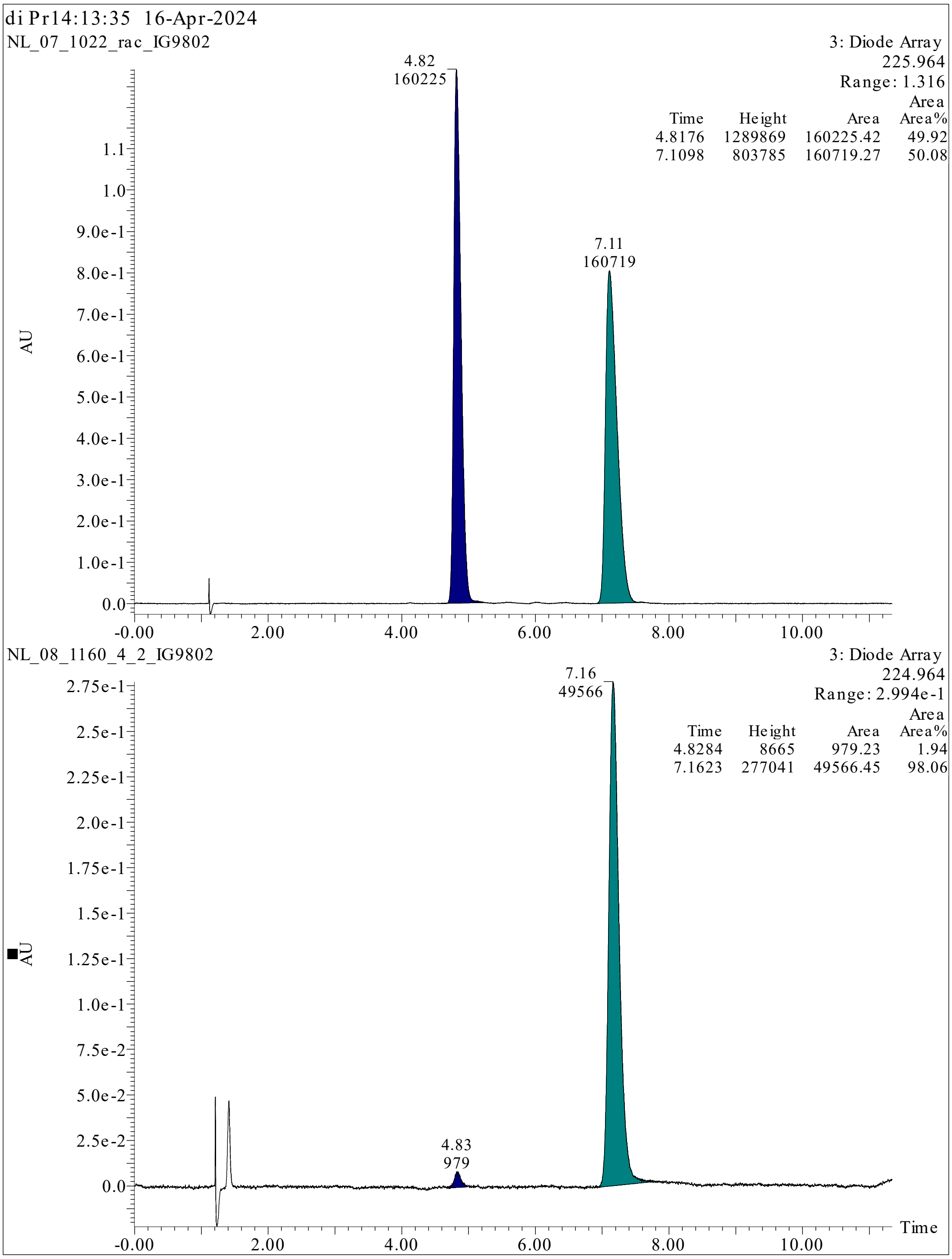

4.4. Reaction scale up

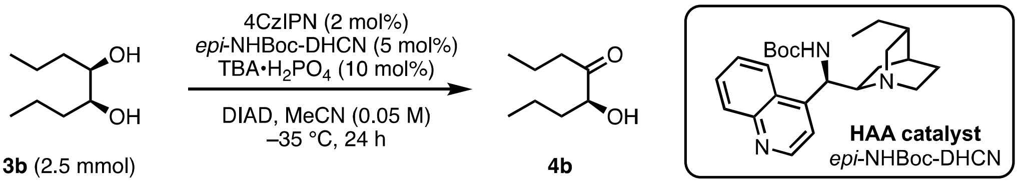

(4*R*,5*S*)-octane-4,5-diol **3b** (366 mg, 2.5 mmol), 4CzIPN (39.0 mg, 0.05 mmol), *epi*-NHBoc-DHCN (49.0 mg, 0.125 mmol), TBA·H_2PO_4 (84.9 mg, 0.25 mmol) and DIAD (505 mg, 2.5 mmol) were weighed into a 50 mL conical flask (with B19 neck) equipped with a stirrer. Dry MeCN (50 mL) was next added and the conical flask was sealed with a B19 septa and wrapped with Parafilm®. The flask was placed into the reactor and allowed to cool to –35 ˚C with stirring for 0.5 h, after which the lights were switched on (see **Figure S10** for illustration). The reaction was irradiated and stirred at –35 ˚C for 24 h, after which the solvent was removed under reduced pressure. Analysis of the crude reaction mixture indicated *ca.* 3:1 mixture of product **4b** relative to remaining starting material **3b**.

Purification by column chromatography (SiO_2, 20–100% CH_2Cl_2/hexanes) afforded **4b** as a clear oil (255 mg, 1.78 mmol, 71%), with spectroscopic data identical to that reported for **4b** arising from reaction at 0.1 mmol scale. An analogous derivation for chiral SFC analysis (*vide supra*) afforded an analytical sample of **Bz-4b** for analysis. This indicated that **4b** was formed in 97% ee (chiral SFC traces shown below).

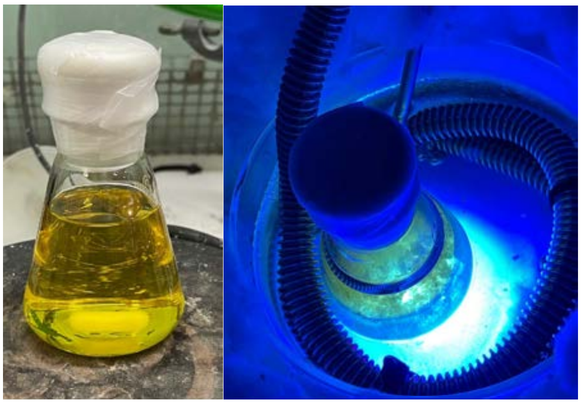

Figure S10*. (Left) reaction set up after sealing with B19 septa and parafilmed; (right) top view of reaction vessel in reactor with photoirradiation*

Chiral SFC Analysis (Bz-4b for product 4b at 2.5 mmol scale): CHIRALPAK IG (CO_2: MeOH, 98:2, 2.5 mL min^{-1}, 40 °C, 227 nm) t_R = 4.83 (minor), 7.16 (major) minutes, 97% ee

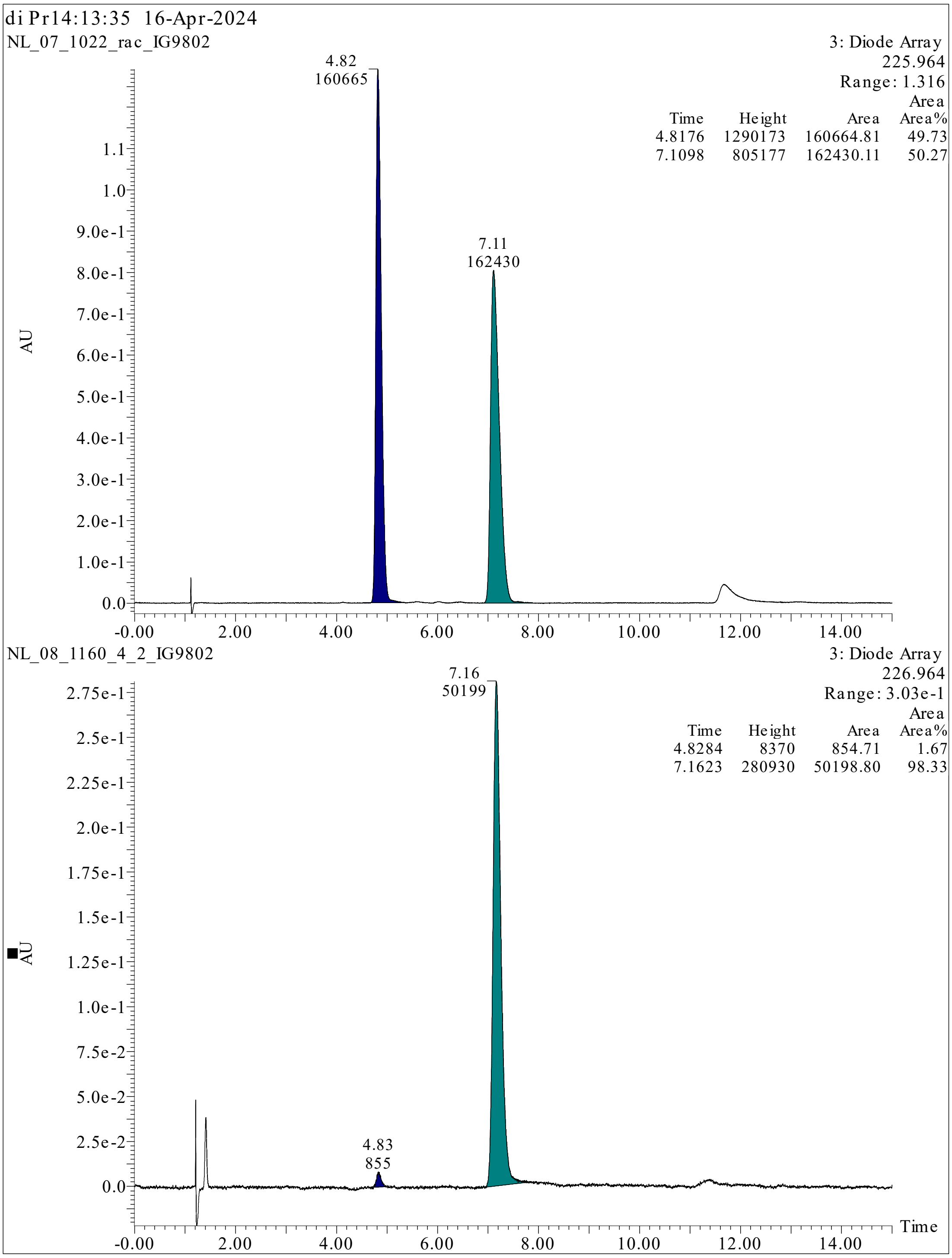

5. Ineffective substrates

For full transparency of the limitations of this method, a list of ineffective substrates are included below. These are categorized as substrates that exhibit *either* 1) poor reactivity, *or* 2) suboptimal reactivity and/or enantioselectivity. In general, five-membered meso diols, and substrates bearing inductively withdrawing group close to the reaction site are poorly effective.

5.1. Substrates that give poor reactivity

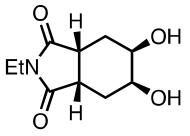

8%, messy profile

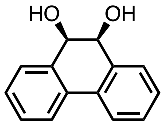

0%, no SM recovered
Putative oxidation to dione

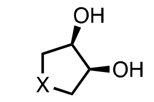

X = O: 0%, poor SM recovery
X = NHBoc: 6%, messy profile

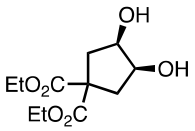

10% (80% SM)

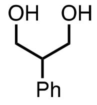

13% (80% SM)

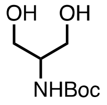

<2% (80% SM)

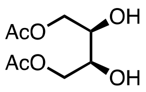

0% (95% SM)

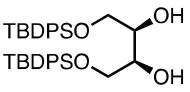

0% (95% SM)

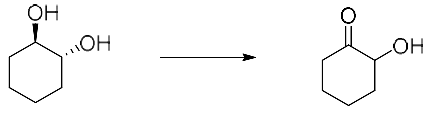

rac

7% NMR yield

The following substrates gave no reactivity, but were also insoluble in the reaction solvent

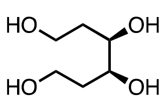

0% (90% SM)

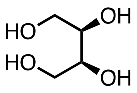

0% (77% SM)

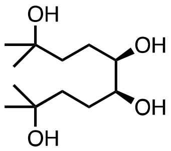

0% (63% SM)

5.2. Substrates with sub-optimal reactivity and/or enantioselectivity

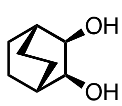

54%, 30% ee
Good reactivity
but poor enantioselectivity

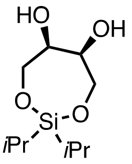

13%, 80% ee
Poor reactivity but
good enantioselectivity

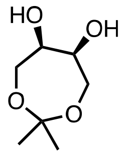

20%, 60% ee
Product unstable +
Moderate enantioselectivity

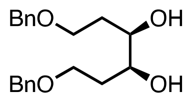

30%, 32% ee
Moderate reactivity but
poor enantioselectivity

5.3 Cyclic 1,3 diol substrate

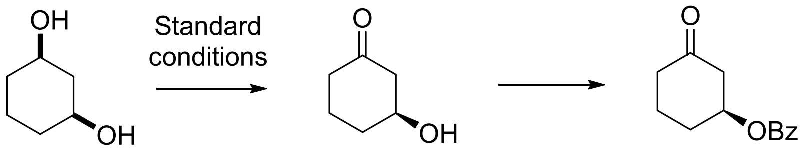

(1*R*,3*S*)-cyclohexane-1,3-diol

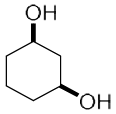

A commercially available mixture of syn and anti-cyclohexane-1,3-diol (1g, 8.61 mmol) was purified by column chromatography (extensive amount of SiO_2, 20 to 30% acetone/hexanes) to afford the syn isomer (136 mg, 1.17 mmol) as a white solid.
^{1}H NMR (700 MHz, $CDCl_3$) δ 3.85 – 3.79 (m, 2H), 2.03 (d, *J* = 12.4 Hz, 1H), 1.96 – 1.84 (m, 4H), 1.80 – 1.73 (m, 2H), 1.60 – 1.52 (m, 1H), 1.47 – 1.38 (m, 2H), 1.35 – 1.28 (m, 1H).
^{13}C NMR (176 MHz, $CDCl_3$) δ 68.5, 41.9, 33.9, 18.1.

Data in agreement with literature (*Chem. Lett.*, **2021**, *50*, 471 – 474)

(S)-3-hydroxycyclohexanone

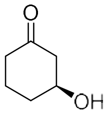

(1*R*,3*S*)-cyclohexane-1,3-diol (11.6 mg, 0.10 mmol) was subjected to General Procedure **B**, with 4CzIPN (3.9 mg, 0.005 mmol), *epi*-NHBoc-DHCN (4.0 mg, 0.01 mmol), TBA·H_2PO_4 (8.5 mg, 0.025 mmol), DIAD (20 mg, 0.1 mmol) in MeCN (4 mL) and reacted for 24 h. Purification by column chromatography (SiO_2, 1% EtOH in $CHCl_3$ to 15 % Et_2O and 1% EtOH in $CHCl_3$) afforded the title compound as a clear oil (4.5 mg, 0.040 mmol, 40%). Note: product is volatile, and care must be taken on solvent evaporation to avoid product loss.

^{1}H NMR (700 MHz, $CDCl_3$) δ 4.21 – 4.17 (m, 1H), 2.66 (ddt, *J* = 14.0, 4.1, 1.1 Hz, 1H), 2.41 (dd, *J* = 14.1, 7.6 Hz, 1H), 2.35 – 2.28 (m, 2H), 2.12 – 2.06 (m, 1H), 2.05 – 1.99 (m, 1H), 1.80 – 1.75 (m, 1H), 1.73 – 1.67 (m, 1H).
^{13}C NMR (176 MHz, $CDCl_3$) δ 209.7, 69.9, 50.6, 41.0, 33.0, 20.7.
$[\alpha]_D$: +3.7 (c = 0.43, T = 25 °C, $CHCl_3$).

Literature $[\alpha]_D$ value: +40.1 (c = 0.8; $CHCl_3$). (*J. Am. Chem. Soc.* **2016**, *138*, 16839−16848)

Derivatization for enantiomeric excess (ee) determination through benzoylation according to literature procedures (*J. Am. Chem. Soc.* **2016**, *138*, 16839−16848). **Chiral SFC Analysis:** CHIRALPAK IC (CO_2: MeOH, 95:5, 2.5 mL min^{-1}, 40 °C, 238 nm) t_R = 7.41 (minor), 8.21 (major) minutes. 10% ee.

6. Mechanistic analysis

6.1. Initial Mechanistic Hypothesis

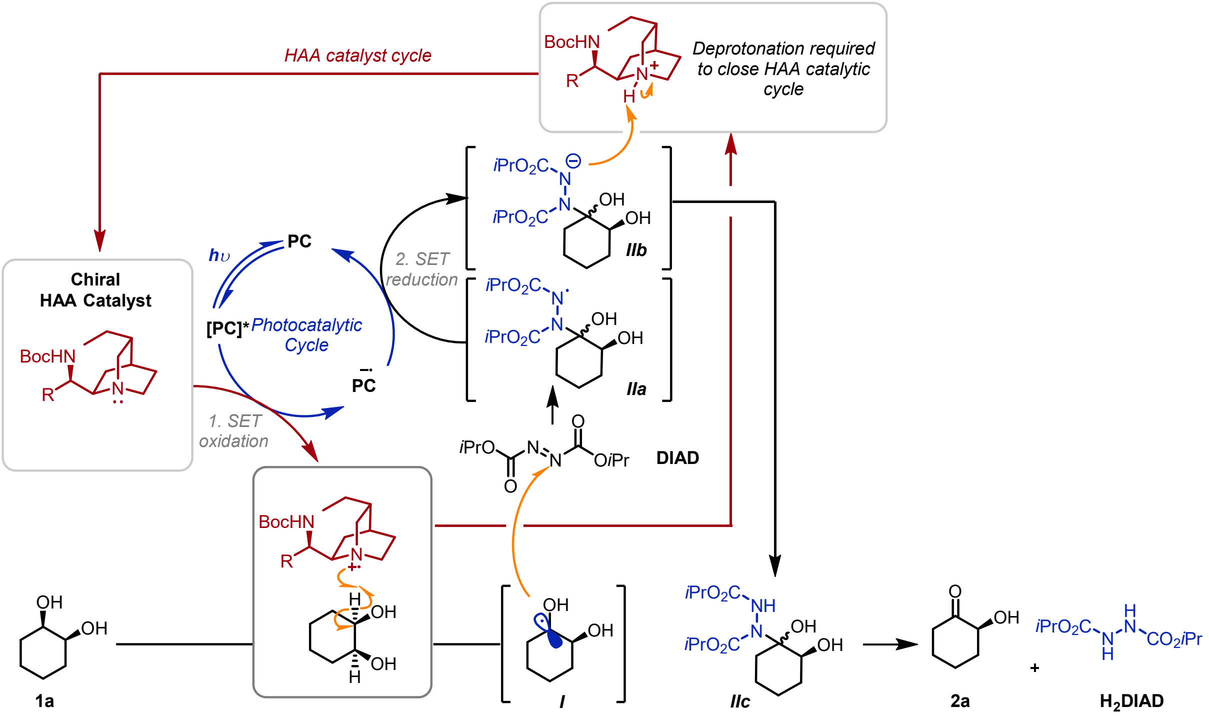

Our initial mechanistic hypothesis stems from our prior work and key literature precedents (SI Refs 2, 37). This hypothesis invokes:

1. Initial reductive quenching of the excited state 4CzIPN photocatalyst to form 4CzIPN$^{\bullet-}$, with a concomitant single electron transfer (SET)-mediated oxidation of the chiral HAA catalyst to the corresponding radical cation, analogous to Refs 2, 37.
2. Enantiodetermining HAA by the chiral HAA catalyst to generate desymmetrized ketyl radical **I**
3. Capture of the ketyl radical with DIAD to generate the radical adduct *IIa*, which undergoes SET reduction to close the photocatalytic cycle generate anion *IIb*
4. Anion *IIb* is expected to deprotonate the cationic chiral quinuclidinium species to close the chiral HAA catalytic cycle. This forms adduct *IIc*, which spontaneously eliminates to generate product **2a** and H_2DIAD as the isolable stoichiometric reduced byproduct.

6.2. Stern Volmer Fluorescence Quenching Study

To investigate the validity of the mechanism proposed in Section **6.1**, Stern Volmer Fluorescence quenching studies were conducted to identify which species preferentially quenches the excited state photocatalyst.

Stern-Volmer quenching was performed using an Agilent Cary Eclipse Fluorimeter. Experiments were recorded using a 3.5 mL quartz cuvette (Thor Lab: catalog number: CV10Q35FEP) equipped with septa-lined screw cap under nitrogen. A general procedure is described below:

To a quartz cuvette cell was added i) 0.2 mL of a photocatalyst stock solution (0.01 mM in MeCN), ii) varying volume (0.0, 0.1, 0.2, 0.4, 0.6, 0.8 mL) of a quencher stock solution (20 mM in MeCN), and iii) acetone such that the total cell volume was 2.0 mL. The cell was sealed and gently sparged with N_2 (balloon) for 5 minutes prior to irradiation. Samples were irradiated at the l_{max} of 4CzIPN (365 nm) and fluorescence was measured at 550 nm. Individual data points (I_0/I) denotes the ratio of emission intensity between a blank sample and a defined concentration of quencher. Each I_0/I value represent an average of two readings.

Fluorescence quenching studies were conducted with individual reaction components (i) DIAD, (ii) cyclohexane-1,2-diol **1a**, iii) *epi*-NHBoc-DHCN and iv) TBA·H_2PO_4 to determine the relative quenching rate between each species with the excited state photocatalyst.

Fluorescence quenching studies for individual reaction components

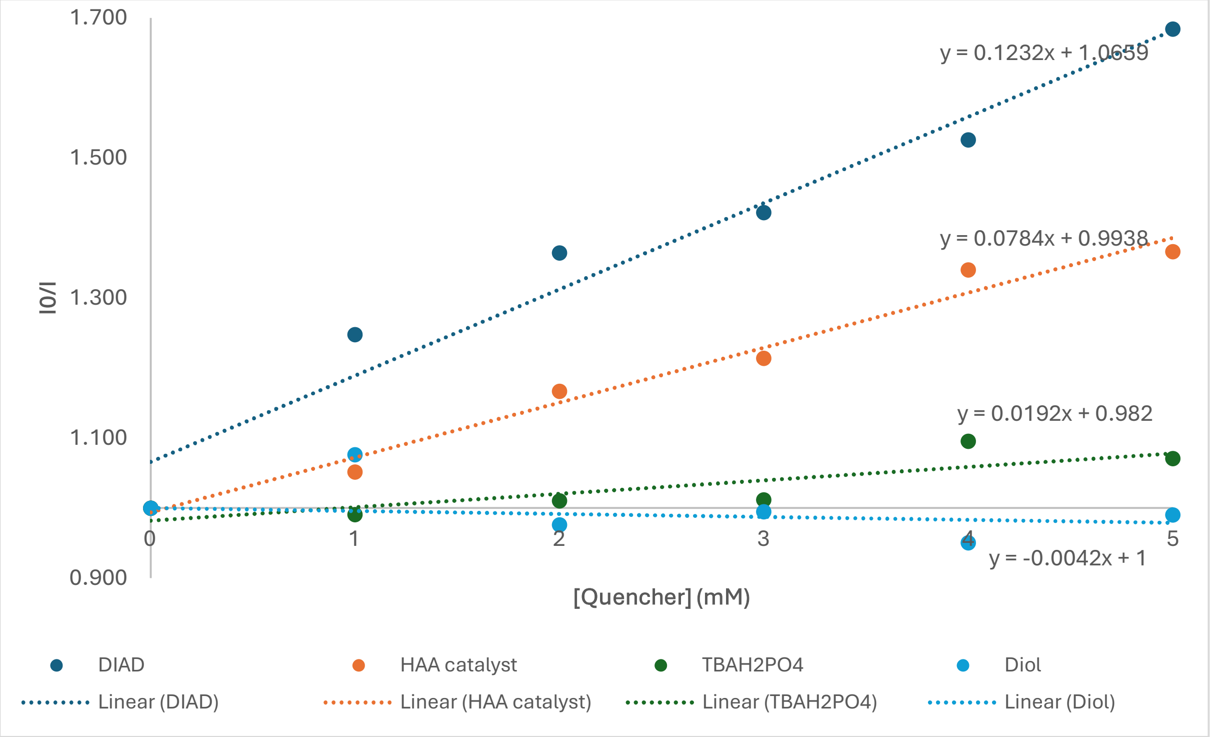

Cyclohexane-1,2-diol

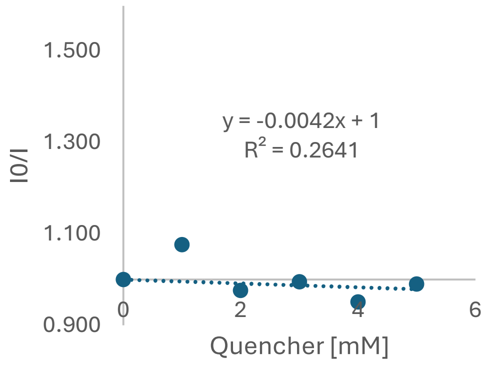

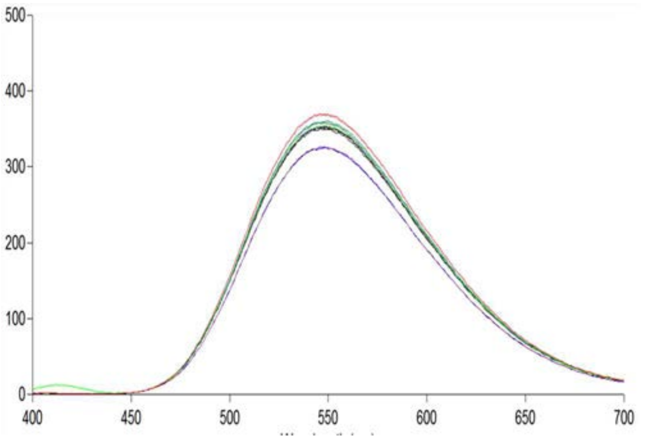

TBA·H$_2$PO$_4$

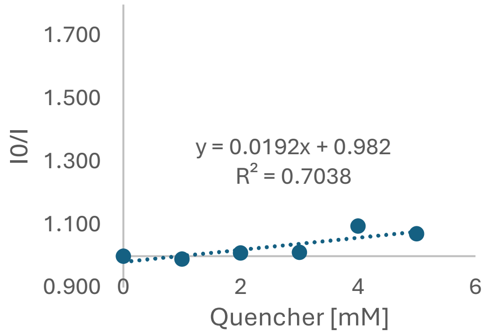

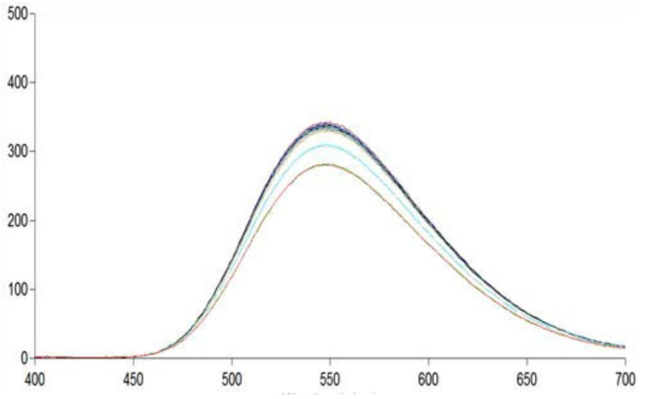

HAA catalyst (epi-NHBoc-DHCN)

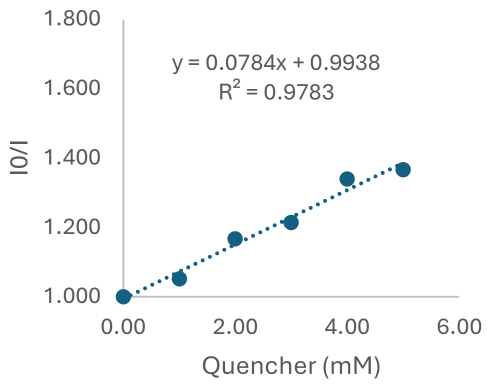

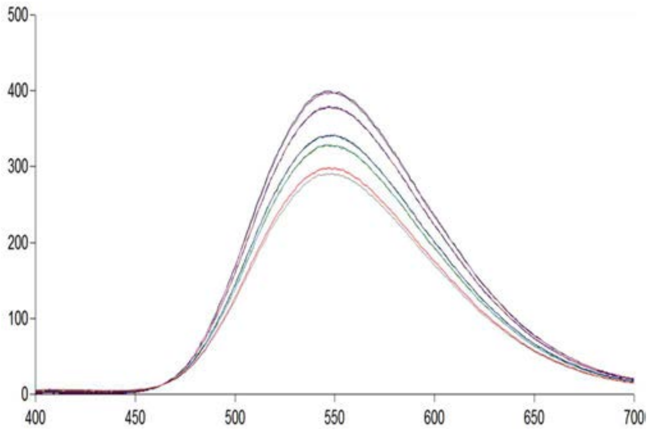

TBA·H$_2$PO$_4$

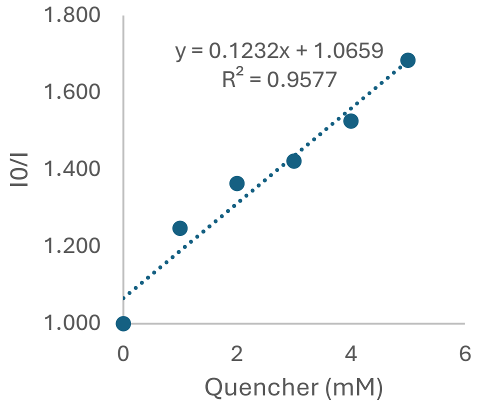

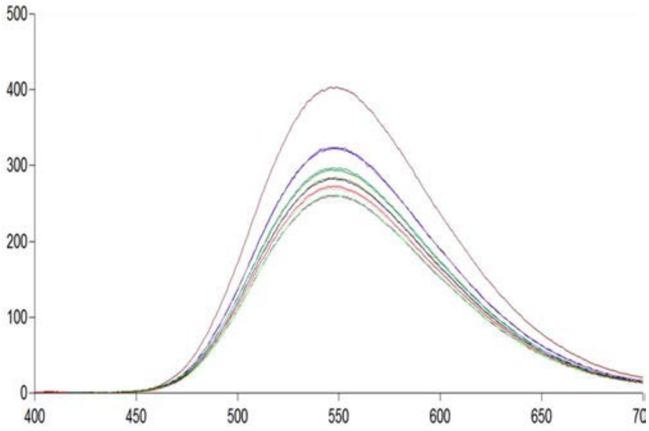

No quenching was observed for cyclohexane-1,2-diol. Low quenching observed for TBA·H_2PO_4. Appreciable linear quenching was observed for *epi*-NHBoc-DHCN and DIAD; rate of quenching was higher for DIAD than *epi*-NHBoc-DHCN. Given that the initial reaction concentration of DIAD is 10x higher than *epi*-NHBoc-DHCN, this indicates that DIAD is the preferential quencher of the excited state photocatalyst and that preferential quenching of the photocatalyst by *epi*-NHBoc-DHCN to initially form the corresponding radical cation does not take place (*i.e.* mechanistic scenario outlined in Section **6.1** is unlikely the major pathway).

Additive studies to verify nature of fluorescence quenching

Two possible modes of quenching could occur between the excited state 4CzIPN and DIAD:

- *Possibility 1*: DIAD could quench the excited state photocatalyst through single electron transfer, reducing DIAD to generate $DIAD^{-\cdot}$ (i.e. oxidative quenching of 4CzIPN to generate $4CzIPN^{+\cdot}$).
- *Possibility 2*: DIAD could quench the excited state photocatalyst through Dexter energy transfer to generate the DIAD triplet diradical

To deduce which of these is the most likely quenching pathway, a series of additive studies were conducted with DIAD to investigate its reactivity in the presence of blue light irradiation, as well as in the presence of the photocatalyst

Monoadditive studies with DIAD under photoirradiation

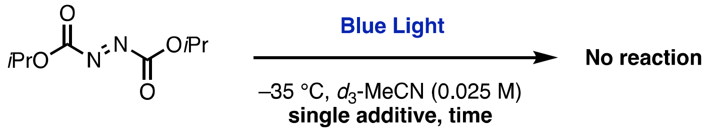

Entry	Additive	2h	8 h
1	None	n/r	n/r
2.	+ 4CzIPN (5 mol%)	n/r	n/r
3.	+ *epi*-NHBoc-DHCN (10 mol%)	n/r	n/r
4.	+ TBA·H_2PO_4 (25 mol%)	n/r	n/r

Blue light irradiation of DIAD with each individual reaction component leads to no changes in the ^{1}H NMR for DIAD (clean observation of DIAD only, see **Figure S11**). This indicates that DIAD does not react by itself under blue light irradiation or in the presence of a photocatalyst (4CzIPN)

Multiple additive studies with DIAD under photoirradiation

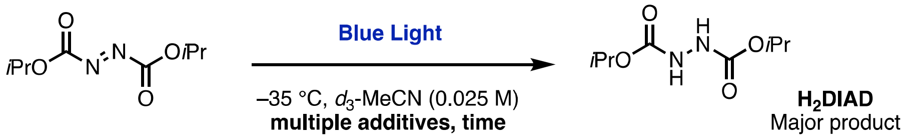

Entry	Additive 1	Additive 2	2h<br>DIAD:H_2DIAD	8 h<br>DIAD:H_2DIAD
1	**4CzIPN (5 mol%)**	***epi*-NHBoc-DHCN (10 mol%)**	**1:0.43**	**1:2.55**
2.	4CzIPN (5 mol%)	TBA·H_2PO_4 (25 mol%)	1:0.05	1:0.25
3.	*epi*-NHBoc-DHCN (10 mol%)	TBA·H_2PO_4 (25 mol%)	1:0.05	1:0.26
4.	**Additives in Entry 3**	**+ 4CzIPN (5 mol%)**	**1:0.23**	**1:3.55**

Main reactivity seen is reduction of DIAD to form H_2DIAD. Without exogenous reductants, this implies that the combination of photocatalyst and HAA catalyst results in the catalytic reduction

of DIAD to generate H_2DIAD (**Figure S12**). Reactivity only seen with both photocatalyst and HAA catalyst *epi*-NHBoc-DHCN; omission of either gives minimal reactivity over the same time period.

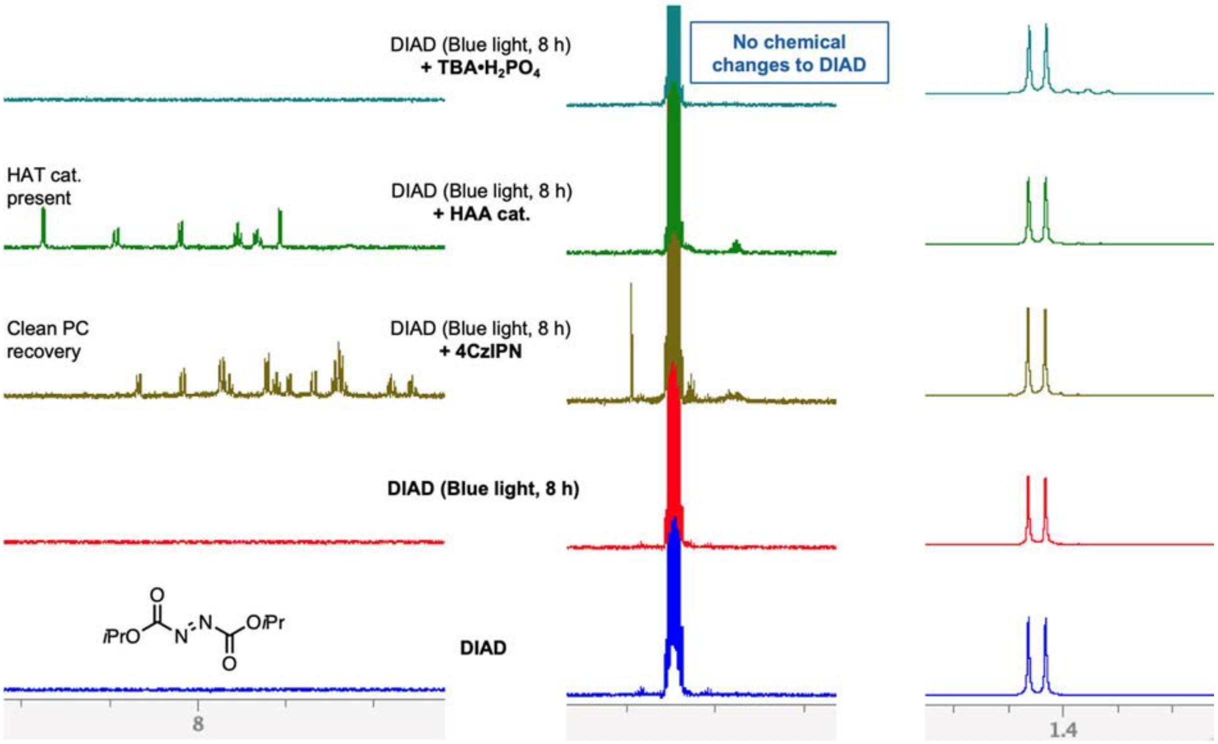

Figure S11. ^{1}H NMR from mono-additive studies indicating no reaction to DIAD under blue light irradiation, with 4CzIPN, HAA catalyst or with TBA·H_2PO_4

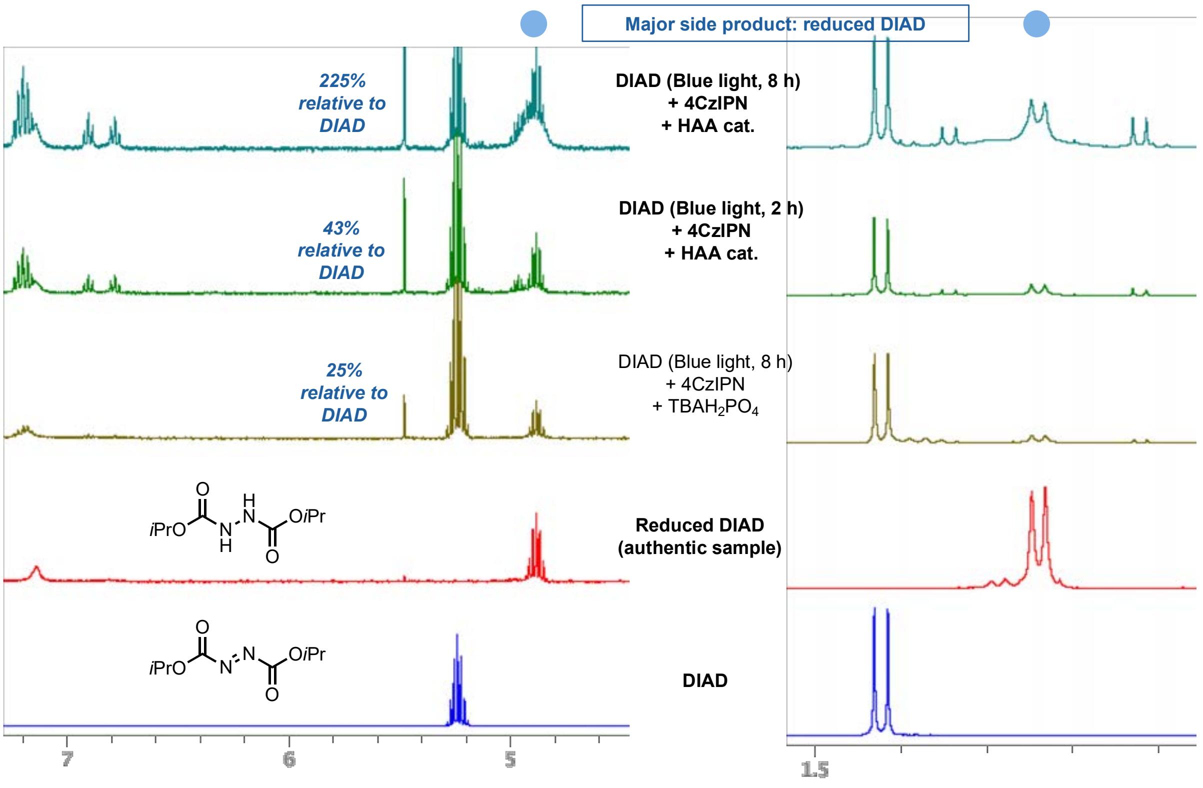

Figure S12. ^{1}H NMR identifying the main byproduct being reduced DIAD (H_2DIAD), and its relative formation with and without HAA catalyst. Substantially increased amounts of H_2DIAD was only formed in the presence of HAA catalyst

This experiment implies that, for catalytic reduction of DIAD to occur (a redox dependent pathway), both a photocatalyst and the HAA catalyst are required. *Together with the empirical observation that alkenes are tolerated in this reaction, this provides evidence against a triplet diradical quenching pathway* (1. triplet diradical species would react with alkenes, which are tolerated in this reaction, 2. reactivity leading to chemical modification of DIAD would be observed in the absence of HAA catalyst), *while providing support for an oxidative quenching process* (4CzIPN oxidizes to form $4CzIPN^{+\cdot}$, DIAD reduces to form $DIAD^{-\cdot}$, which can only operate catalytically if $4CzIPN^{+\cdot}$ then oxidizes the HAA catalyst to close the catalytic cycle) operating in this reaction manifold.

Further evidence to support an oxidative quenching pathway (4CzIPN to $4CzIPN^{+\cdot}$) with concomitant single electron reduction of DIAD to form $DIAD^{-\cdot}$ is found by comparing experimental $E_{1/2}$ values: $E_{1/2}(DIAD/DIAD^{-\cdot}) = -0.5$ V;[35] $E_{1/2}(4CzIPN^{+\cdot}/4CzIPN^{*}) = -1.18$ V,[36] which gives an overall reaction E_{cell} ($E_{reduction} - E_{oxidation}$) value of = +0.68 V, indicating thermodynamic feasibility of the proposed oxidative quenching process. The resultant strongly oxidizing $4CzIPN^{+\cdot}$ radical cation then oxidizes the HAA catalyst ($E_{1/2}(4CzIPN^{+\cdot}/4CzIPN) = +1.49$ V)[36] to close the photocatalytic cycle, while generating the active aminium radical cation required for enantioselective HAA to take place

For this set of Additive experiments, H_2DIAD formation constitutes a formal reduction of DIAD. A putative source of H could arise from the solvent acetonitrile. To probe this, the same reaction was conducted in protiated acetonitrile, and a primary KIE was observed, providing evidence suggesting that the terminal reductant in this process arises from the solvent.

Relative amounts of product formed at partial conversion (2 h)

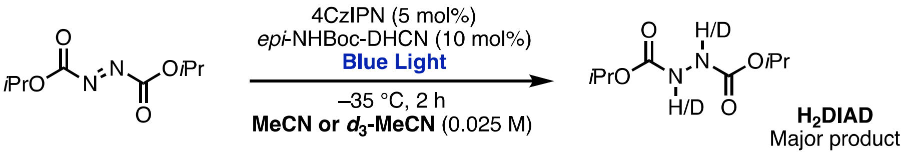

Entry	Conditions	d_3-MeCN DIAD:H_2DIAD at 2 h	MeCN DIAD:H_2DIAD at 2 h	P_H/P_D
1	4CzIPN (5 mol%) *epi*-NHBoc-DHCN (10 mol%)	**1:0.43**	**1:1.22**	**2.83**

Conclusions

- *Taking into relative account concentrations of reaction components, it seems likely that DIAD could be the preferential quencher of the excited state photocatalyst*
- *On the excited state photocatalyst and is reduced to form $DIAD^{-\cdot}$*

Impact of TBA·H_2PO_4 additive on fluorescence quenching

TBA·H_2PO_4 additive was found to be important for high reaction efficacy. To probe whether the additive was important on enabling more effective quenching of the excited state photocatalyst, a separate study was conducted with mixture of reaction components in an equimolar ratio (i) DIAD + TBA·H_2PO_4 (1:1), ii) HAA catalyst *epi*-NHBoc-DHCN + TBA·H_2PO_4 (1:1), denoted by diamond data labels. For comparison, datapoints from DIAD and *epi*-NHBoc-DHCN (circle data labels) are plotted alongside.

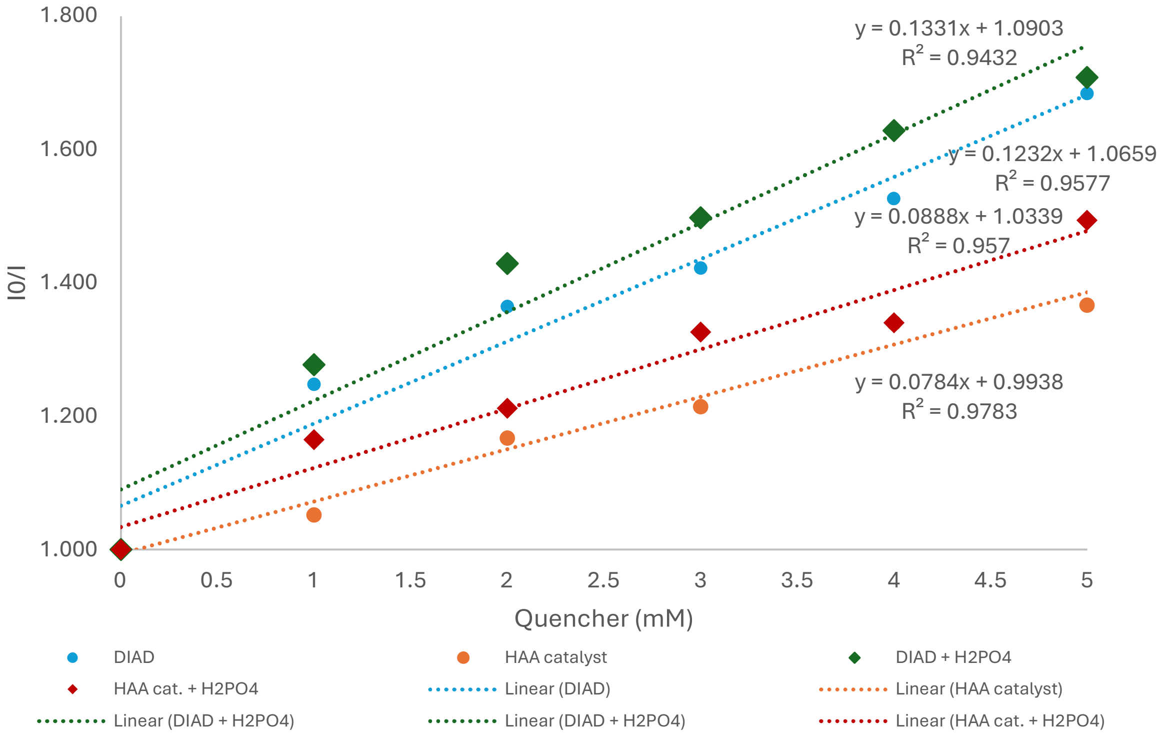

DIAD (blue circle) *vs.* DIAD + TBA·H_2PO_4 (1:1; green diamonds; fluorescence trace on right)

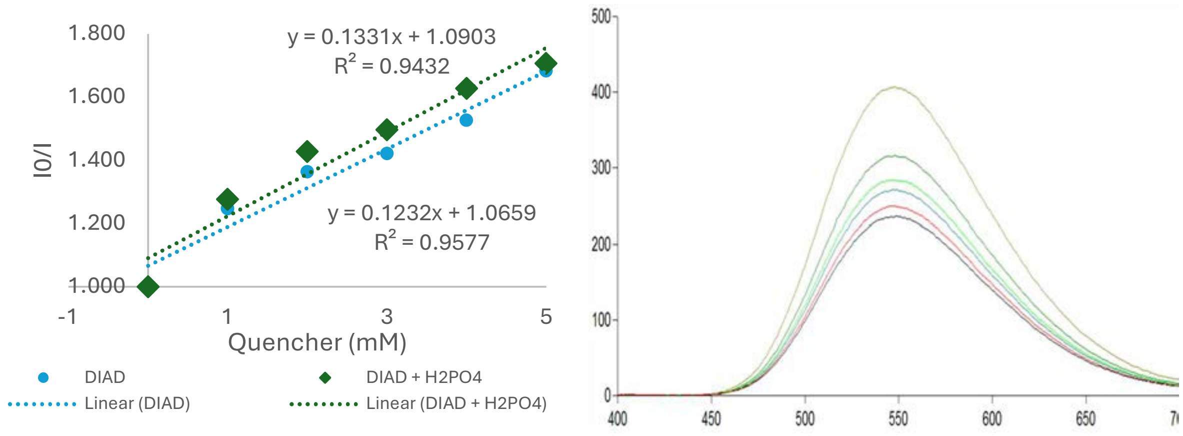

epi-NHBoc-DHCN (orange circle) *vs.* *epi*-NHBoc-DHCN + TBA·H_2PO_4
(1:1; red diamonds; fluorescence trace on right)

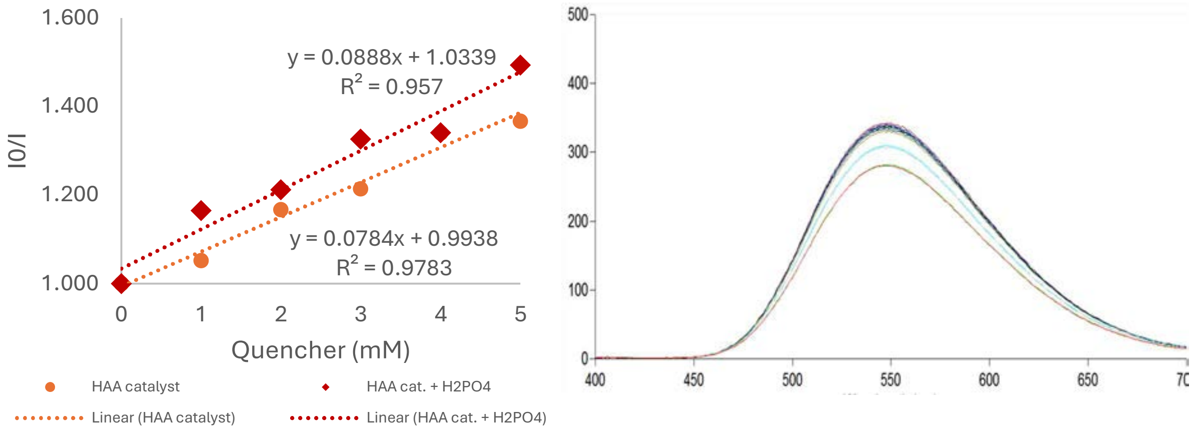

This analysis indicates that the addition of TBA·H_2PO_4 did not meaningfully alter the relative quenching ability of both DIAD and HAA catalyst *epi*-NHBoc-DHCN. This provides evidence against TBA·H_2PO_4 playing an essential role in promoting energy transfer from the excited state photocatalyst to either quenchers (DIAD or *epi*-NHBoc-DHCN), and implicates its vital role downstream of this process.

Key conclusions

- *TBA·H_2PO_4 additive does not meaningfully affect relative quenching abilities of either DIAD or HAA catalyst to the extent where it explains the dramatic drop-off in reactivity*
- *TBA·H_2PO_4 additive plays a role downstream of photocatalyst quenching step*

6.3. Role of TBA·H_2PO_4 in reaction

Reaction order analysis with TBA·H_2PO_4

In addition to the control experiments outlined in **Reaction Optimization** (*vide supra*), we verified the importance of TBA·H_2PO_4 by conducting reaction order analysis. **General Procedure B** was followed and the reaction was stopped at time points specified below.

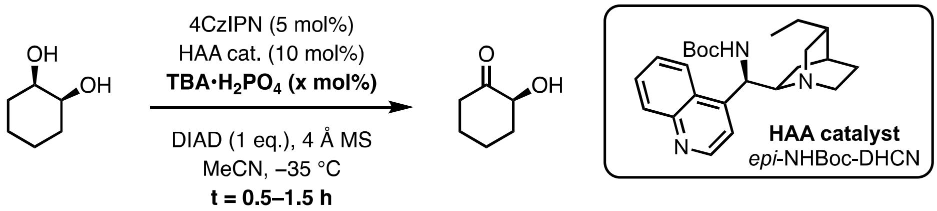

Time (h)	No TBA·H_2PO_4	TBA·H_2PO_4 (5 mol%)	TBA·H_2PO_4 (12.5 mol%)	TBA·H_2PO_4 (25 mol%)
0.5	0%	20%	30%	20%
0.75	12%	36%	40%	56%
1.0	14%	72%	80%	74%
1.5	15%	90%	88%	82%

Yield of product reported and determined by crude NMR using CH_2Br_2 as an internal standard

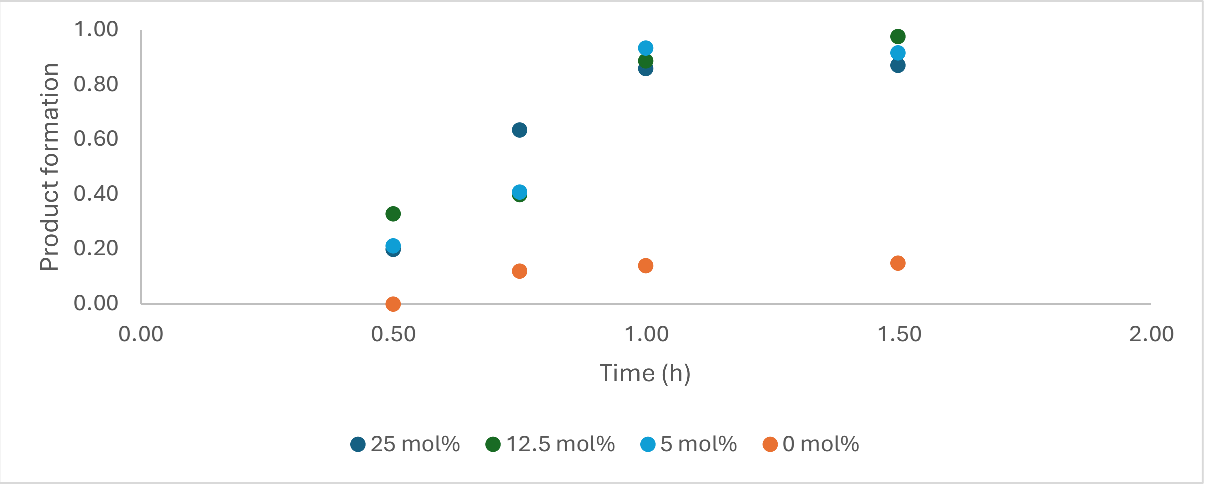

Comparable initial rates were observed for reactions containing 5, 12.5 and 25 mol% of the TBA·H_2PO_4, implying that the reaction is not ordered in TBA·H_2PO_4 at reaction relevant concentrations. In contrast, the reaction both slows down and stalls on omission of the TBA·H_2PO_4 additive, implying both its vital role in the reaction pathway, as well as indicating that the reaction begins to be ordered in TBA·H_2PO_4 below 5 mol%.

This analysis confirms that TBA·H_2PO_4 is an important additive to achieve high conversions. It is co-catalytic in nature, and is not ordered at reaction relevant concentration in our present catalytic system.

Time course analysis for enantioselective diol epimerization with and without TBA·H_2PO_4

Stern Volmer analysis indicates that the TBA·H_2PO_4 additive does not play a major role in facilitating the photoredox process (i.e. it is non-essential for either the generation of DIAD$^{\cdot-}$ or

HAA$^{+\cdot}$). Knowing that this additive plays a vital role in facilitating reactivity and continued catalytic turnover, its importance is deduced to be relevant for a downstream process after the initial photoredox event. This could include

1. **HAA step** (ketyl radical generation from the meso diol substrate)
2. **Proton transfer step** (either deprotonation of protonated HAA catalyst, or protonation of an intermediary anionic species, e.g. DIAD$^{-\cdot}$)

We note that the TBA·H_2PO_4 additive was not strictly essential for our previously reported enantioselective diol epimerization process, which is thought to proceed via a common HAA step to generate the same ketyl radical intermediate. Therefore, we conducted a time course experiment investigating the rate of product formation for the enantioselective diol epimerization *with and without* TBA·H_2PO_4, and directly comparing this against the rate of product formation for enantioselective oxidation *with and without* TBA·H_2PO_4 (see next page for full reaction conditions). To ensure consistency across both reaction classes, 1 eq. of dodecanethiol was used for the diol epimerization (c.f. 1 eq. of DIAD was used for diol oxidation) and 4Å MS—a non essential additive—was omitted.

	Diol Oxidation		Diol Epimerization	
Time (h)	No TBA·H_2PO_4	TBA·H_2PO_4 (12.5 mol%)	No TBA·H_2PO_4	TBA·H_2PO_4 (12.5 mol%)
0.5	0%	30%	33%	15%
0.75	12%	40%	-	-
1.0	14%	80%	48%	-
1.25	-	-	-	52%
1.5	15%	88%	61%	57%
2.0	-	-	73%	65%

Yield of product reported and determined by crude NMR using CH_2Br_2 as an internal standard

The time course graph below shows a superimposed plot of product formation over time for enantioselective oxidation (blue) and enantioselective diol epimerization (green). Light blue and light green denote the corresponding reactions that were conducted in the absence of TBA·H_2PO_4.

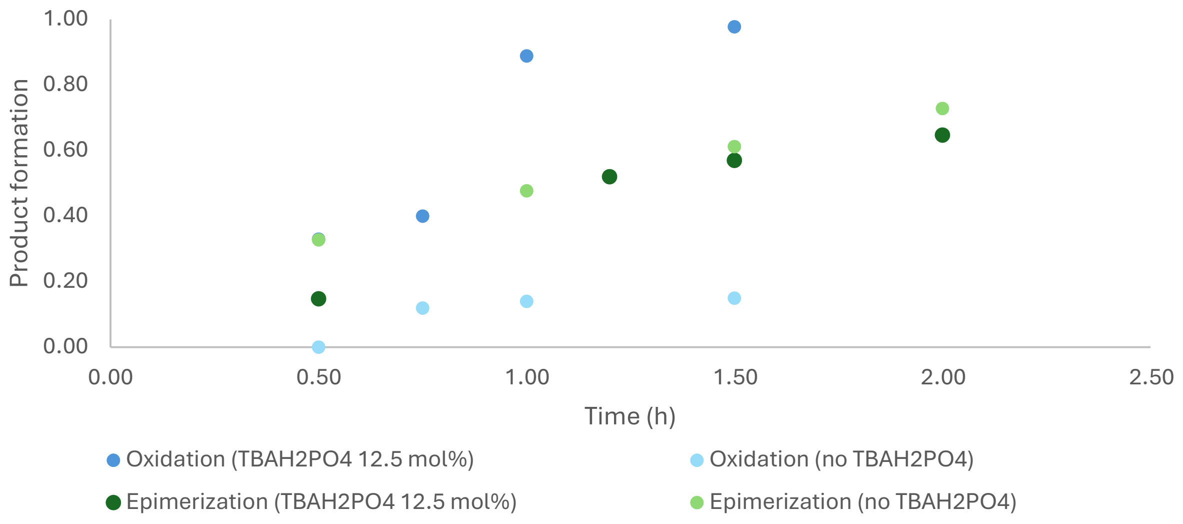

Oxidation

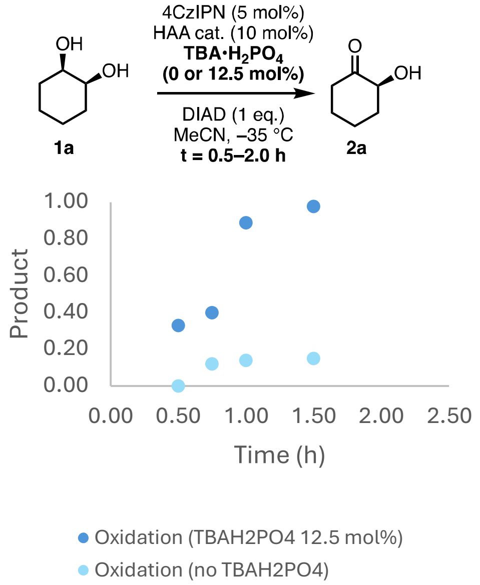

Epimerization

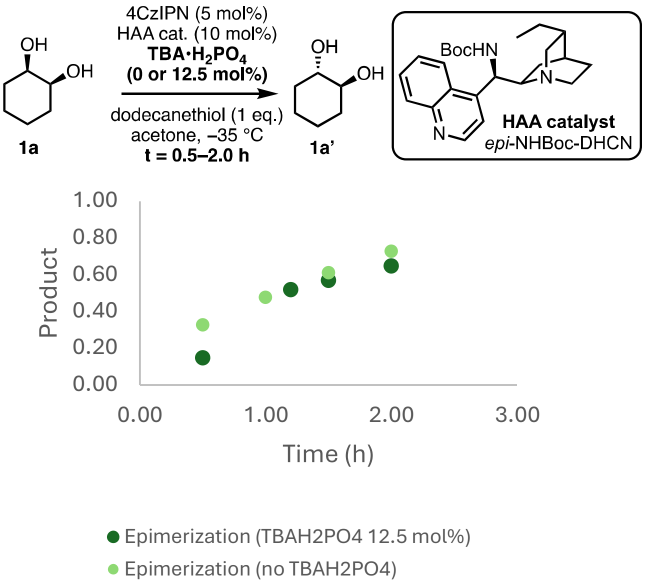

From the graphs presented above, two conclusions can be made from the time course analysis. First, there is no meaningful difference in the rate of product formation with and without TBA·H_2PO_4 in the enantioselective epimerization process (right – green data points). This is in direct contract where the omission of TBA·H_2PO_4 dramatically reduces reactivity in the enantioselective oxidation process (left – blue data points).

Because both the enantioselective oxidation and epimerization processes proceed through a common ketyl radical intermediate and utilizes largely similar reaction conditions (same photocatalyst, HAA catalyst and substrate), this implies that the TBA·H_2PO_4 additive is non-essential for the HAA step/generation of the ketyl radical, or else we would expect to see a comparable reduction in reaction rate for the enantioselective epimerization process.

On first glance, it appears that the rate of reaction is slower for the enantioselective diol epimerization compared to the oxidation. However, we must consider that in the epimerization process, the presumed unselective thiol (RSH) quenching of the ketyl radical requires >1 equivalent of ketyl radical ***I*** to form one equivalent of product **1a'** (**Figure S13**). This is contrasted with the enantioselective oxidation process, where each ketyl radical is irreversibly oxidized to the hydroxyketone (i.e. a 1:1 correspondence between product to the ketyl radical that is formed).

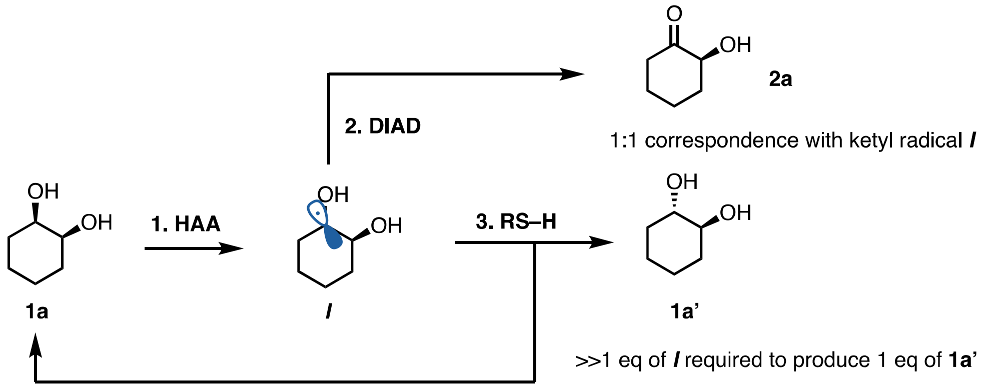

Figure S13. Molar comparison between oxidation vs. epimerization

Applying a crude correction factor, which estimates a 1.5:1 *dr* (*i.e.* 0.6) in the quenching step favouring product formation (approximate *dr* observed in the Giese addition of the ketyl radical to electron deficient olefins),[2] this gives a "corrected" plot accounting for the total mol of ketyl radical formed in the enantioselective epimerization process. Note that this correction factor does not take into account a non-linear decrease in reaction rate resultant of low substrate concentration and must be considered a high-level estimate.

This analysis suggests that the rate of HAA/ketyl radical formation is, in fact, comparable for diol epimerization *with and without* TBA·H_2PO_4, *and* with diol oxidation *with* TBA·H_2PO_4. This provides further evidence in support that the TBA·H_2PO_4 additive is having little impact on the HAA step/ketyl radical generation.[37] This result also implies that the TBA·H_2PO_4 additive plays an important role to support catalytic turnover downstream of this step.

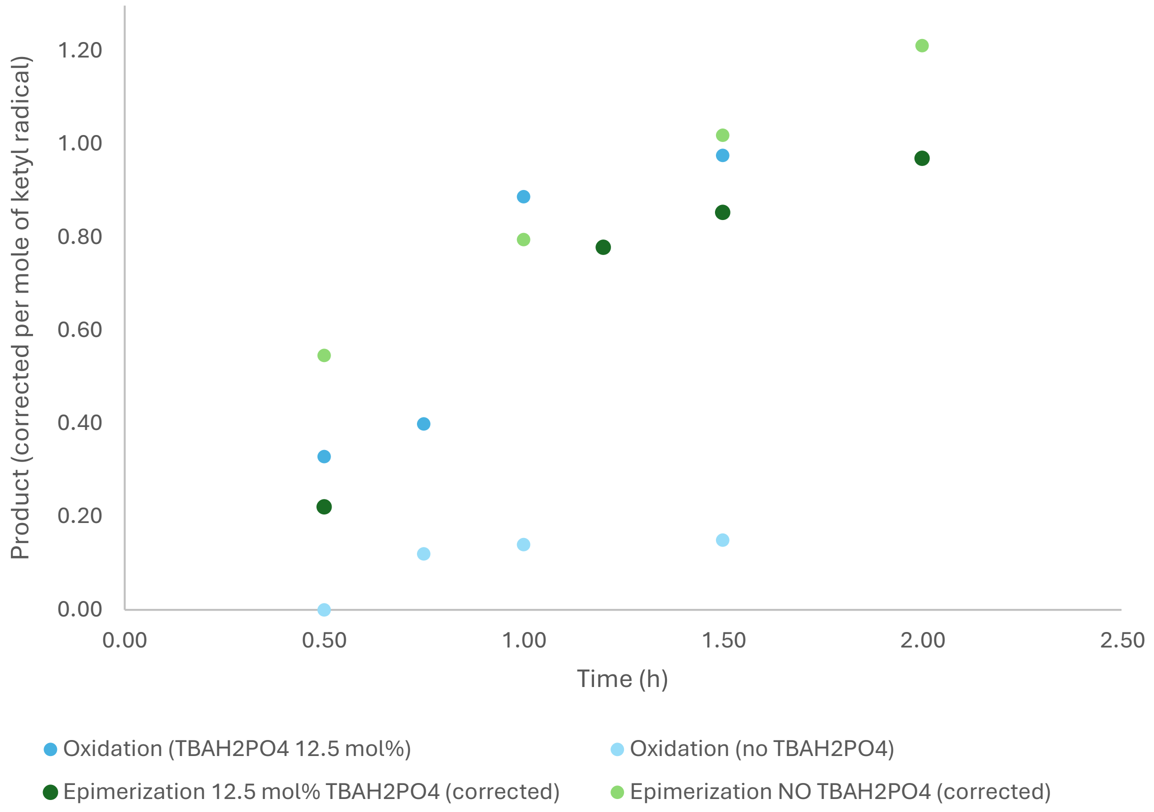

Key conclusions

- *TBA·H_2PO_4 additive is non-essential for HAA step/ketyl radical generation*
- *TBA·H_2PO_4 additive is important downstream of HAA step*

Isologous additives to probe the role of TBA·H_2PO_4 as an acid/base additive

With the insights gained from the above studies, further experiments were next designed to probe which structural features/properties in the TBA·H_2PO_4 additive was essential for reactivity. Understanding this may enable us to deduce in which part of the reaction pathway the additive was playing the important role.

To verify the importance of the dihydrogen phosphate motif, controlling for charge, geometry and protonation state *inter alia*, a series of isologous additive experiments were next conducted and its effect on the rate of product formation was investigated.

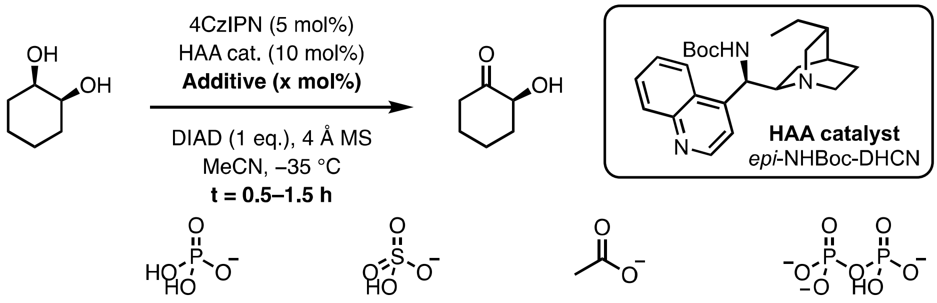

Time (h)	No TBA·H_2PO_4	TBA·H_2PO_4 (12.5 mol%)	TBA·HSO_4 (25 mol%)	TBA·OAc (25 mol%)	$(TBA)_3$·HP_2O_7 (12.5 mol%)
0.5	0%	30%	5%	7%	41%
0.75	12%	40%	6%	10%	71%
1.0	14%	80%	12%	15%	87%
1.5	15%	88%	19%	23%	95%

Yield of product reported and determined by crude NMR using CH_2Br_2 as an internal standard

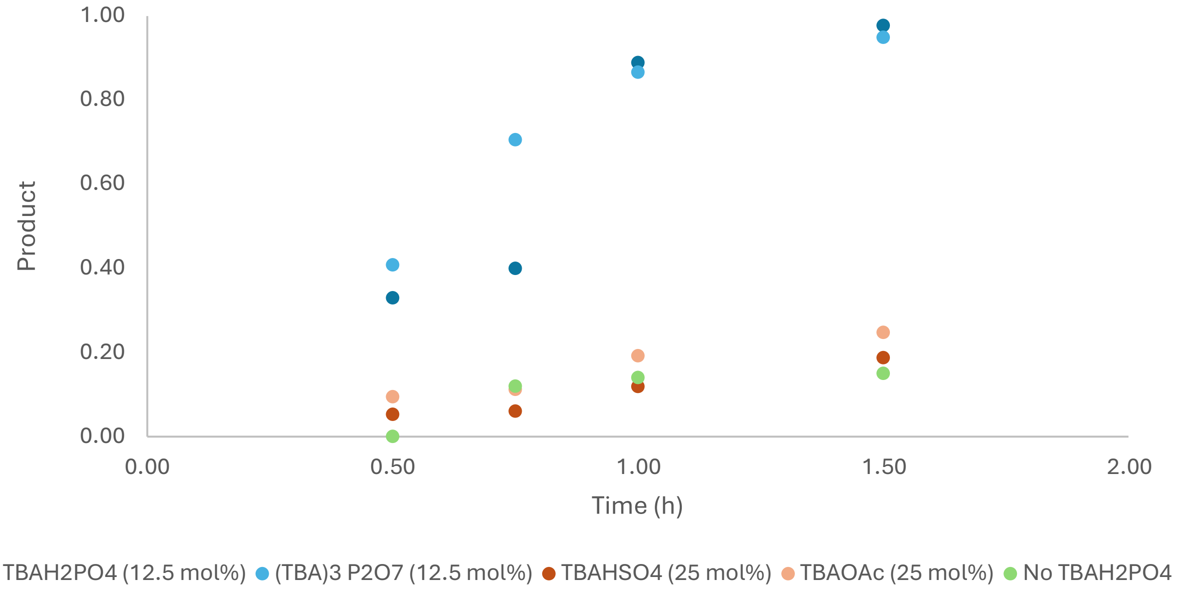

This time course experiment indicates that:

1. Structurally analogous TBA·HSO_4 (proton source), was ineffective
2. A source of exogenous mild base (acetate; proton acceptor) was similarly ineffective

Both TBA·HSO_4 and TBA·OAc additives gave comparable rates to the reaction without any additive, and implies that a simple proton donor or proton acceptor by itself were ineffective in the reaction. This analysis also indicated that the use of an analogous pyrophosphate (one that contains the phosphate motif at a protonation state one below dihydrogen phosphate) was similarly effective, and as effective as TBA·H_2PO_4 in promoting reactivity. Taking the results above into consideration in conjunction with the pKa values of various conjugate bases (**Figure S14**), this raises the

possibility that the TBA·H_2PO_4 additive may be serving as both a catalytic proton donor and acceptor, where the most relevant protonation state is potentially between **$H_2PO_4^-$** and **HPO_4^{2-}**. This is envisaged for the following reasons:

1. Results from HSO_4^- additive rule out the H_3PO_4/$H_2PO_4^-$ protonation states as relevant in the reaction as no rate acceleration was seen with an isologous additive with similar pKa
2. Results from AcO^- additive rule out that the role of TBA·H_2PO_4 as a simple proton acceptor
3. Results from $(TBA)_3$·HP_2O_7 indicate that similar reactivity could be achieved with a phosphate additive one protonation state below TBA·H_2PO_4

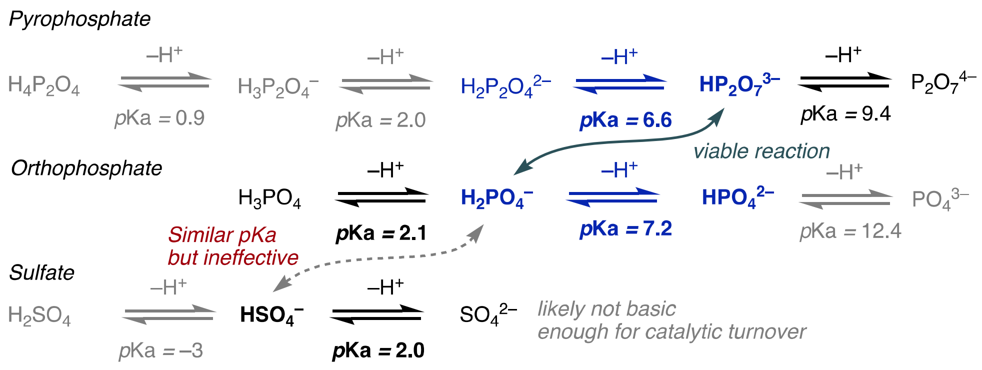

***Figure S14**. Relative pKa values (quoted in H_2O but used as a heuristic for the present reaction system) for various ortho and pyrophosphates, in comparison with sulfates, with the relevant protonation states important in the oxidation reaction highlighted in blue*

Key conclusions

This leads to the proposal that the exogenous TBA·H_2PO_4 additive is likely serving as a proton shuttle/acid-base catalyst, where it serves initially as a proton donor ($H_2PO_4^-$ deprotonation to afford HPO_4^{2-}) and then a proton acceptor (HPO_4^{2-} protonation to reform $H_2PO_4^-$). Simple acids and bases by themselves are ineffective, implicating the role of an amphoteric acid/base to facilitate the oxidation to take place.

Taking into consideration all mechanistic studies detailed above, we believe that the most likely process in which the TBA·H_2PO_4 additive plays an important role is first the protonation of the $DIAD^{-\cdot}$ radical anion to generate the neutral $DIAD^{\cdot}$ radical, and later on the downstream deprotonation of the protonated HAA catalyst.

6.4. Proposed reaction mechanism deduced from mechanistic experiments

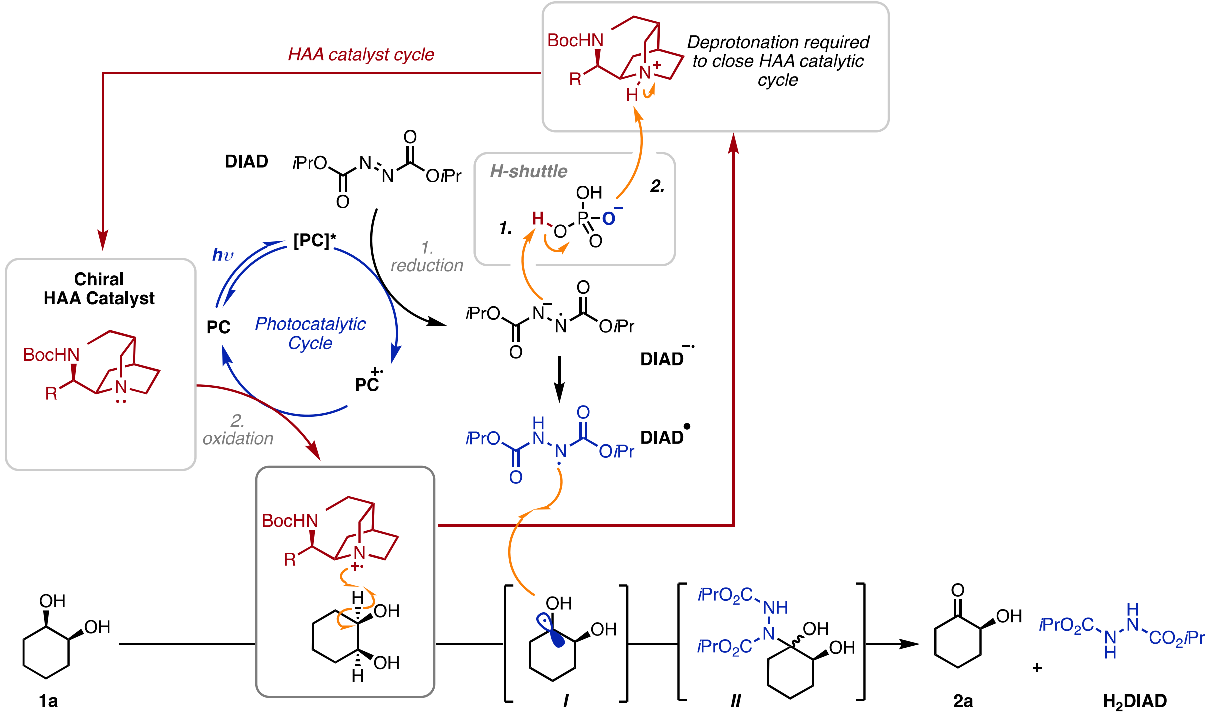

Key mechanistic features

1. Oxidative quenching of 4CzIPN to form 4CzIPN$^{+\cdot}$ to result in SET reduction of DIAD to afford DIAD$^{-\cdot}$ - *deduced by Stern Volmer studies*
2. Oxidation of HAA catalyst to the corresponding aminium radical cation by 4CzIPN$^{+\cdot}$ — this closes the photocatalytic cycle by reforming the ground state 4CzIPN, whilst generating the active HAA catalyst primed for enantioselective HAA.
3. TBA·H_2PO_4 additive does not play a role in assisting SET process, or facilitates HAA from meso diol substrate *- deduced by time course experiments and Stern Volmer studies*
4. TBA·H_2PO_4 serves as an amphoteric proton shuttle/acid-base catalyst initiated first by its deprotonation (i.e. it serves to first protonate another species in the reaction). As the DIAD$^{-\cdot}$ is the next most basic species, TBA·H_2PO_4 likely protonates the radical anion to afford the neutral DIAD$^{\cdot}$. Functionally, this likely mitigates unproductive back electron transfer, where the newly formed 4CzIPN$^{+\cdot}$ accepts an electron from DIAD$^{-\cdot}$ to reform the respective ground state species, preventing the photocatalytic cycle from turning over *– evidence for protonation deduced by isologous additive experiments. Further evidence for its vital role in assisting catalytic turnover can be found in time course experiment against diol epimerization, where the photocatalytic cycle turnover is not contingent upon protonation of a radical anion.*
5. After HAA, we tentatively hypothesize that the formed enantioenriched ketyl radical ***I*** rapidly recombines with the DIAD· to form ***II***, which eliminates to afford the enantioenriched hydroxyketone product **2a** + **H$_2$DIAD** (isolable stoichiometric byproduct). *We cannot rule out alternative mechanisms, for example: direct SET between I and DIAD· to directly form **2a** + **H$_2$DIAD** following protonation, or alternative addition pathways, for example: between I and DIAD, followed by SET reduction to generate II after protonation. The true mechanism may be more complex than the possibilities illustrated here.*
6. The deprotonated HPO_4^{2-} species then assists in turning over the HAA catalyst by deprotonating the protonated amine, as well as regenerating the $H_2PO_4^-$ protonation state

7. References

1. Luo, J.; Zhang, J., *ACS Catal.* **2016,** *6*, 873-877.
2. Lahdenpera, A. S. K.; Dhankhar, J.; Davies, D. J.; Lam, N. Y. S.; Bacos, P. D.; de la Vega-Hernandez, K.; Phipps, R. J., *Science* **2024,** *386*, 42-49.
3. Kuga, T.; Sasano, Y.; Iwabuchi, Y., *Chem. Commun.* **2018,** *54*, 798-801.
4. Beligny, S.; Eibauer, S.; Maechling, S.; Blechert, S., *Angew. Chem. Int. Ed.* **2006,** *45*, 1900-3.
5. Scholte, A. A.; An, M. H.; Snapper, M. L., *Org. Lett.* **2006,** *8*, 4759-4762.
6. Nagasawa, S.; Sasano, Y.; Iwabuchi, Y., *Angew. Chem. Int. Ed.* **2016,** *55*, 13189-13194.
7. Hopf, H.; Hussain, Z.; Menon, R. S.; Raev, V.; Jones, P. G.; Pohl, L. M., *Synlett* **2011,** *2011*, 1273-1276.
8. Sander, M.; Dehmlow, Eckehard V., *Eur. J. Org. Chem.* **2001,** *2001*, 399-404.
9. Johnson, T. A.; Jang, D. O.; Slafer, B. W.; Curtis, M. D.; Beak, P., *J. Am. Chem. Soc.* **2002,** *124*, 11689-11698.
10. Zheng, S.; Chengying, X.; Zheng, M.; Xiaojie, L.; Gang, Q. Polycyclic amide derivative as CDK9 inhibitor, preparation method therefor and use thereof. CN113149996A, 2021.
11. Blakemore, D. C.; Bryans, J. S.; Carnell, P.; Carr, C. L.; Chessum, N. E. A.; Field, M. J.; Kinsella, N.; Osborne, S. A.; Warren, A. N.; Williams, S. C., *Bioorg Med Chem Lett* **2010,** *20*, 461-464.
12. Belokon, Y. N.; Chusov, D.; Peregudov, A. S.; Yashkina, L. V.; Timofeeva, G. I.; Maleev, V. I.; North, M.; Kagan, H. B., *Adv. Synth. Cat.* **2009,** *351*, 3157-3167.
13. Dalmizrak, D.; Göksu, H.; Gültekin, M. S., *RSC Adv.* **2015,** *5*, 20751-20755.
14. Bekfelavi, E. Y.; Yilmaz, Ö.; Sahin, E.; Kus, N. S., *Monatsh Chem* **2021,** *152*, 295-304.
15. Baalmann, M.; Neises, L.; Bitsch, S.; Schneider, H.; Deweid, L.; Werther, P.; Ilkenhans, N.; Wolfring, M.; Ziegler, M. J.; Wilhelm, J.; Kolmar, H.; Wombacher, R., *Angewandte Chemie* **2020,** *132*, 12985-12993.
16. Hodgson, D. M.; Galano, J. M.; Christlieb, M., *Tetrahedron* **2003,** *59*, 9719-9728.
17. Van Delft, F. L.; Rutjes, F. P. J. T.; Dommerholt, F. J. Fused cyclooctyne compounds and their use in metal-free click reactions. US20130137763A1, 2014.
18. Wysocki, L. M.; Dodge, M. W.; Voight, E. A.; Burke, S. D., *Org. Lett.* **2006,** *8*, 5637-5640.
19. Bartlett, S.; Hodgson, R.; Holland, J. M.; Jones, M.; Kilner, C.; Nelson, A.; Warriner, S., *Org. Biomol. Chem.* **2003,** *1*, 2393-2402.
20. Ruggles, E. L.; Hondal, R. J., *Tetrahedron Lett.* **2006,** *47*, 4281-4284.
21. Ariawan, A. D.; Mansour, F.; Richardson, N.; Bhadbhade, M.; Ho, J.; Hunter, L., *Molecules* **2021,** *26*,
22. Hennecke, U.; Müller, C. H.; Fröhlich, R., *Org. Lett.* **2011,** *13*, 860-863.
23. Alvarez, E.; Diaz, M. T.; Perez, R.; Ravelo, J. L.; Regueiro, A.; Vera, J. A.; Zurita, D.; Martin, J. D., *J. Org. Chem.* **1994,** *59*, 2848-2876.
24. Hao, B.; Gunaratna, M. J.; Zhang, M.; Weerasekara, S.; Seiwald, S. N.; Nguyen, V. T.; Meier, A.; Hua, D. H., *J. Am. Chem. Soc.* **2016,** *138*, 16839-16848.
25. Zhang, J.; Xu, T.; Li, Z., *Adv. Synth. Cat.* **2013,** *355*, 3147-3153.
26. Feng, X. M.; Shu, L. H.; Shi, Y., *J. Am. Chem. Soc.* **1999,** *121*, 11002-11003.
27. Anderson, R. J.; Henrick, C. A., *J. Am. Chem. Soc.* **1975,** *97*, 4327-4334.
28. Lohray, B. B.; Enders, D., *Helv. Chim. Acta* **2004,** *72*, 980-984.
29. Wang, L. S.; Wu, F. S.; Jia, X. L.; Xu, Z. S.; Guo, Y. A.; Ye, T., *Org. Lett.* **2018,** *20*, 2213-2215.
30. Nestl, B. M.; Bodlenner, A.; Stuermer, R.; Hauer, B.; Kroutil, W.; Faber, K., *Tetrahedron Asymm.* **2007,** *18*, 1465-1474.
31. Wong, O. A.; Shi, Y. A., *J. Org. Chem.* **2009,** *74*, 8377-8380.
32. Ooi, T.; Saito, A.; Maruoka, K., *J. Am. Chem. Soc.* **2003,** *125*, 3220-3221.
33. Igawa, K.; Kawasaki, Y.; Tomooka, K., *Chem Lett* **2011,** *40*, 233-235.
34. Miller, K. M.; Molinaro, C.; Jamison, T. F., *Tetrahedron Asymm.* **2003,** *14*, 3619-3625.

35. Zweig, A.; Hoffmann, A. K., *J. Am. Chem. Soc.* **2002**, *85*, 2736-2739.
36. Engle, S. M.; Kirkner, T. R.; Kelly, C. B., *Org. Synth.* **2003**, *96*, 1-19.
37. Jeffrey, J. L.; Terrett, J. A.; MacMillant, D. W. C., *Science* **2015**, *349*, 1532-1536.

8. Spectroscopic data for novel compounds

Full ^{1}H and ^{13}C NMR characterization of all novel meso diols, all hydroxyketone products and benzoylated derivatives for ee determination are provided below.

Spectroscopic data for novel *meso* diol substrates

^{1}H NMR (400 MHz, $CDCl_3$) **1f**

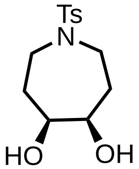

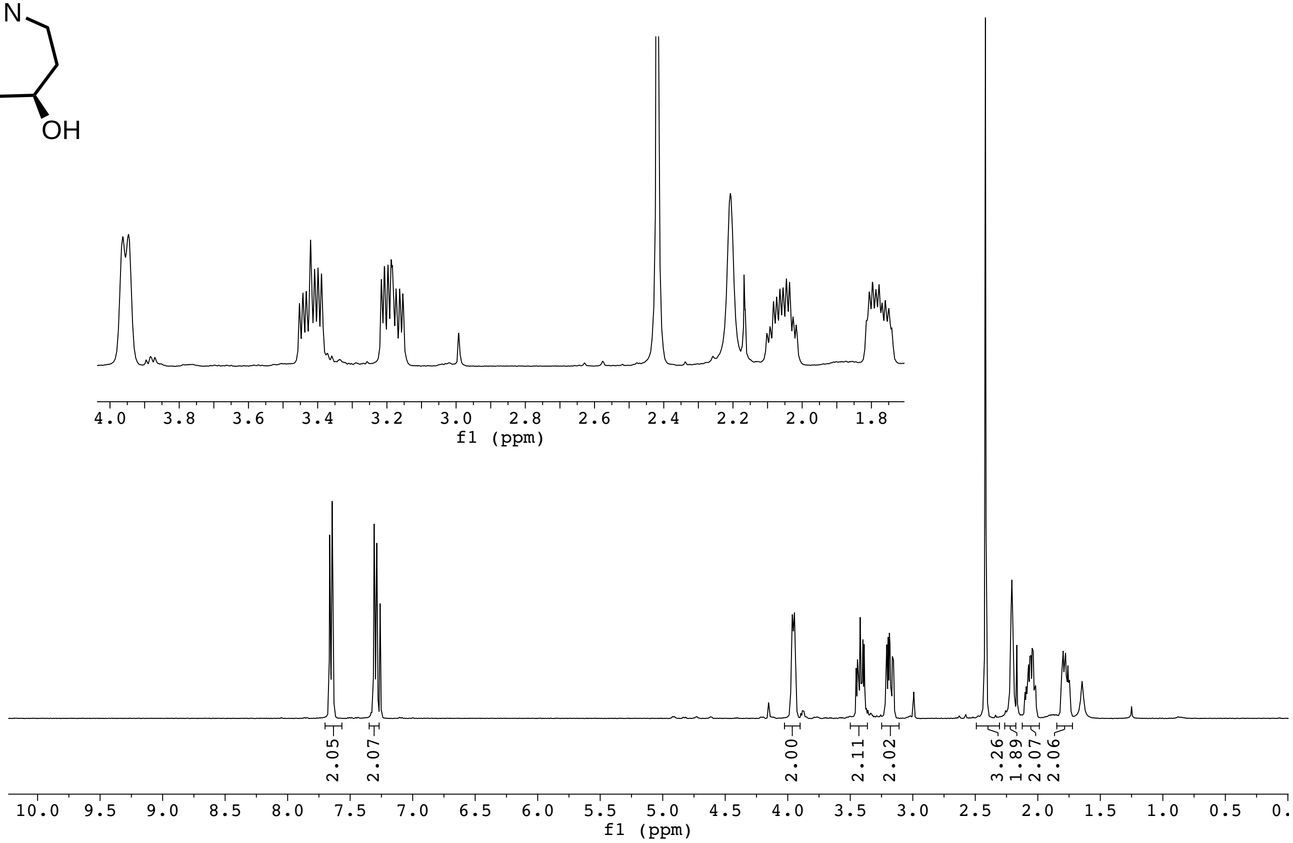

^{13}C NMR (126 MHz, $CDCl_3$) **1f**

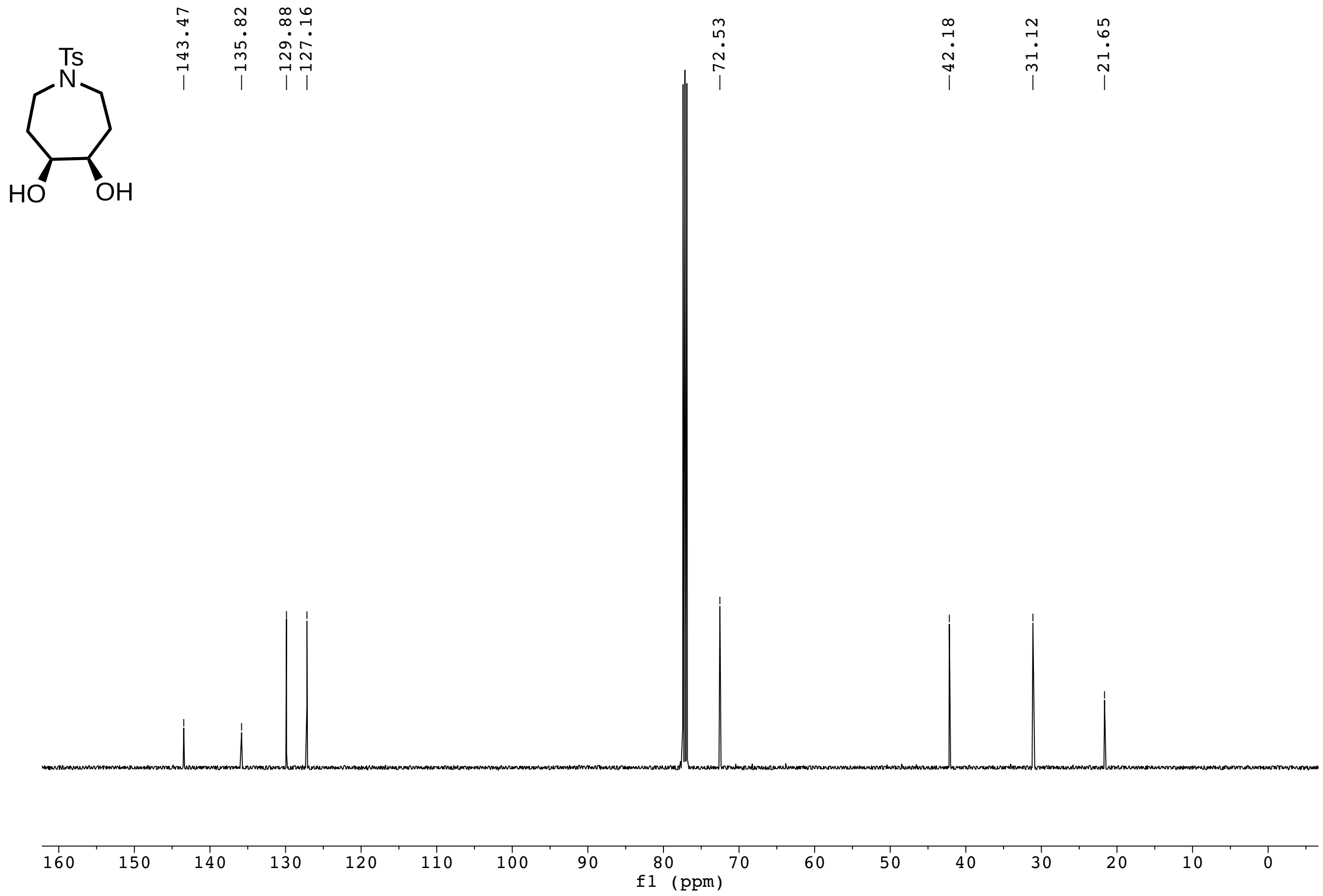

¹H NMR (700 MHz, $CDCl_3$) **1j**

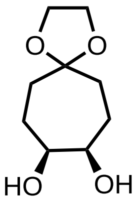

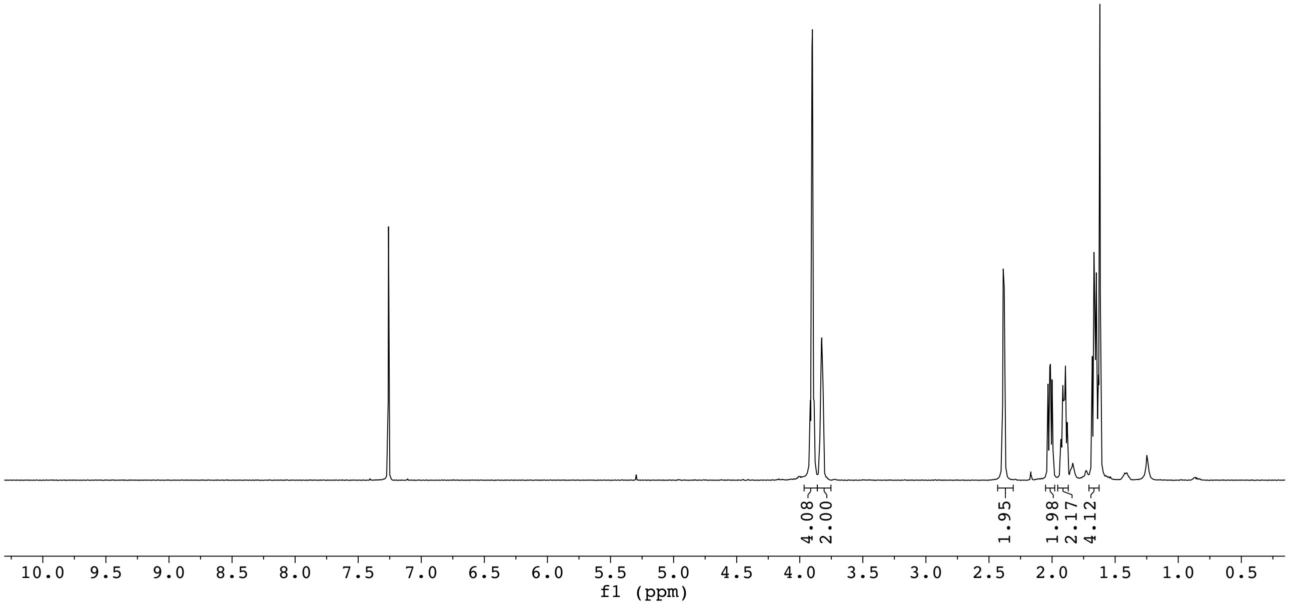

^{13}C NMR (176 MHz, $CDCl_3$) **1j**

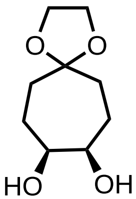

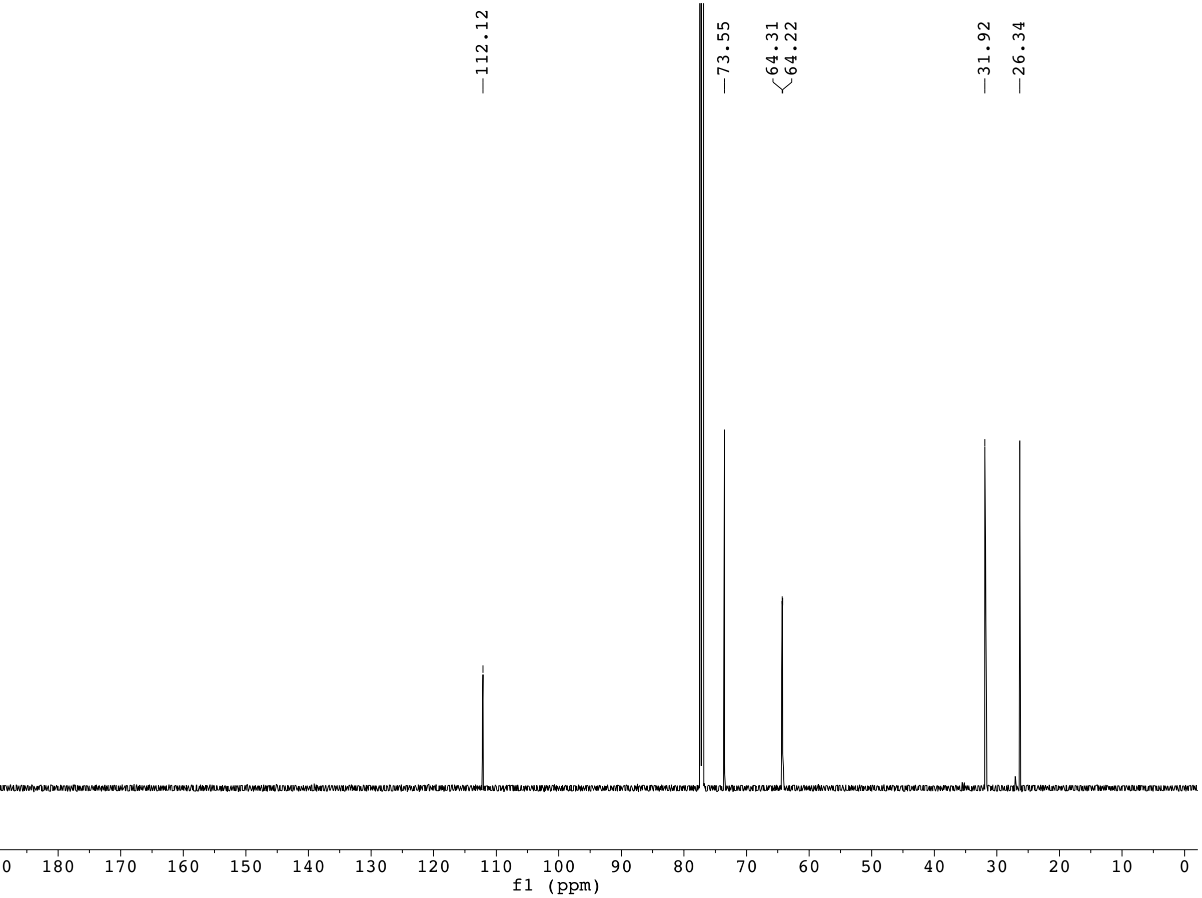

^{1}H NMR (700 MHz, d_3-MeOD) **1n**

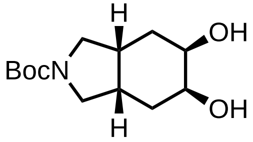

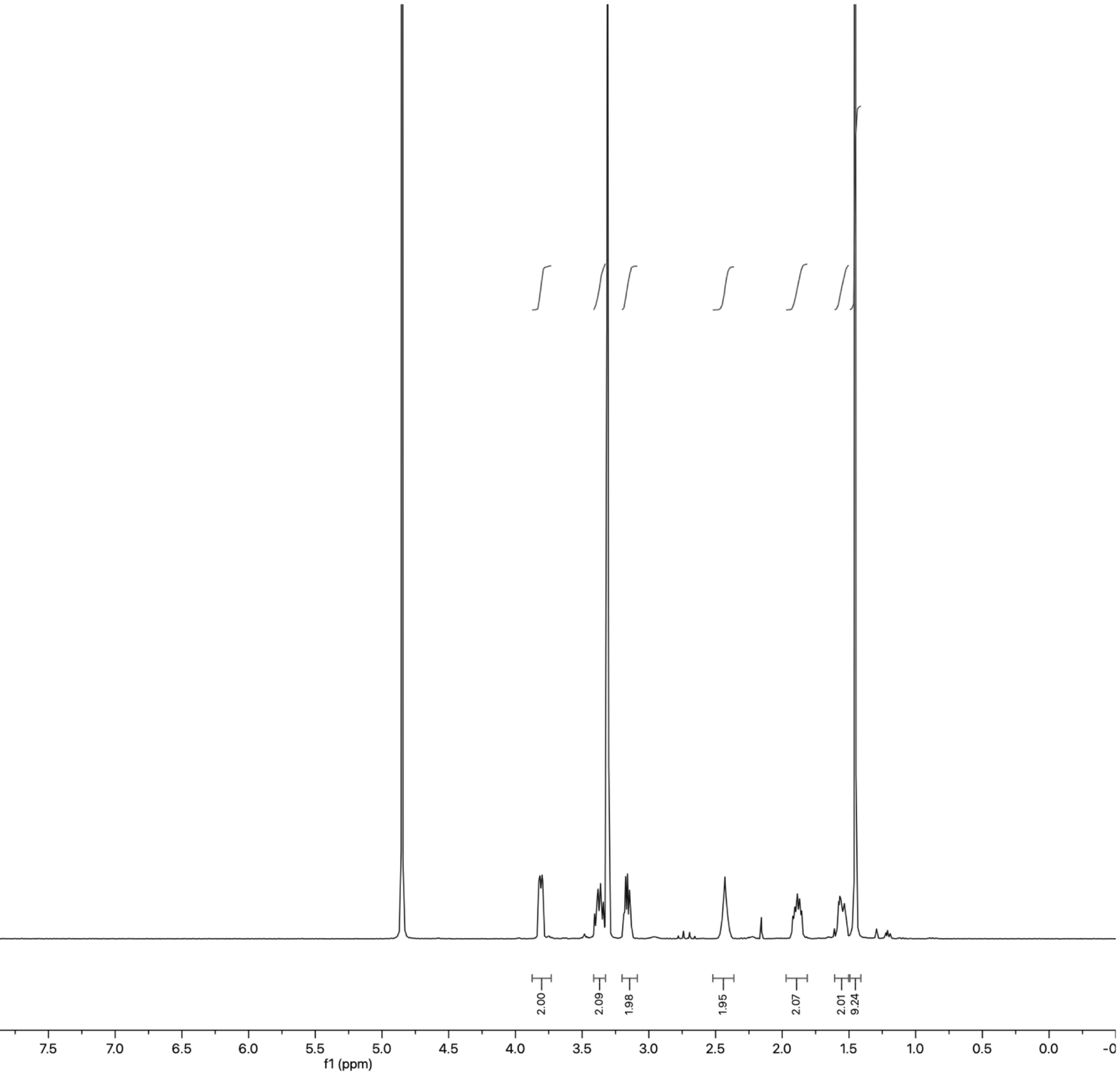

^{13}C NMR (176 MHz, d_3-MeOD) **1n**

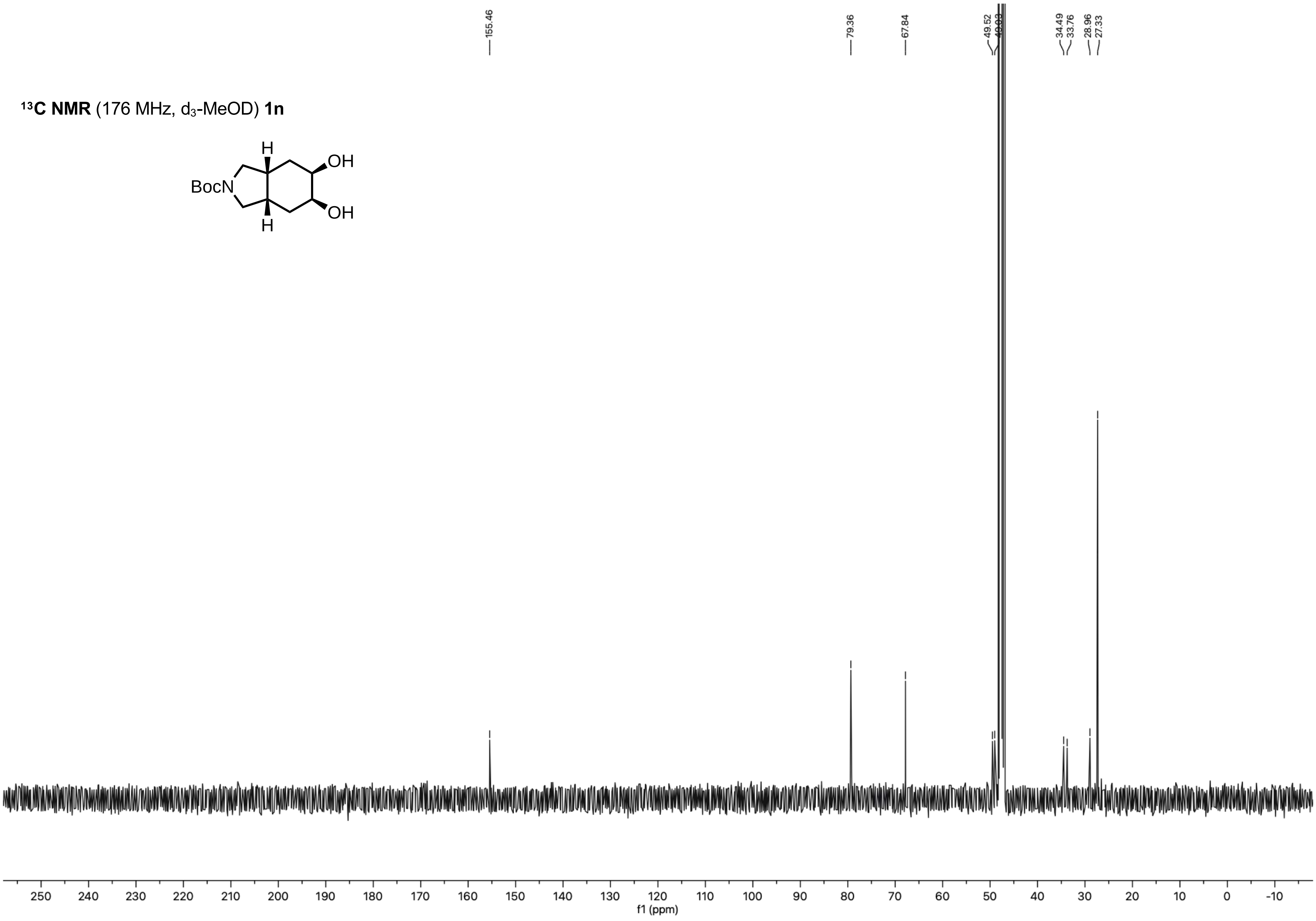

¹H NMR (700 MHz, $CDCl_3$) **1p**

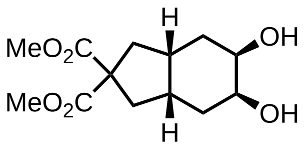

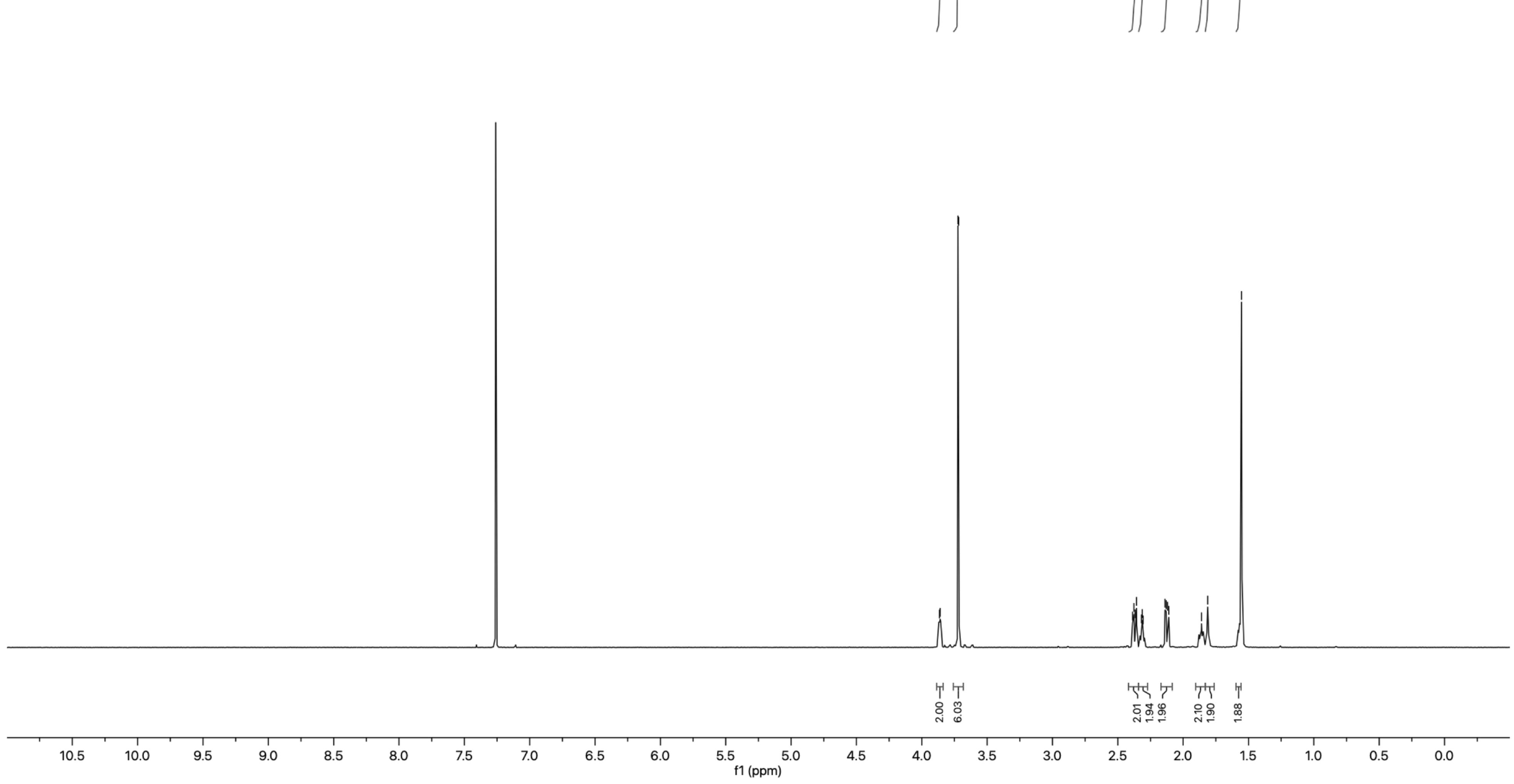

^{13}**C NMR** (176 MHz, $CDCl_3$) **1p**

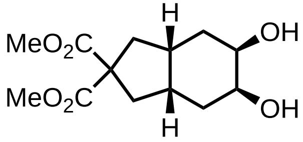

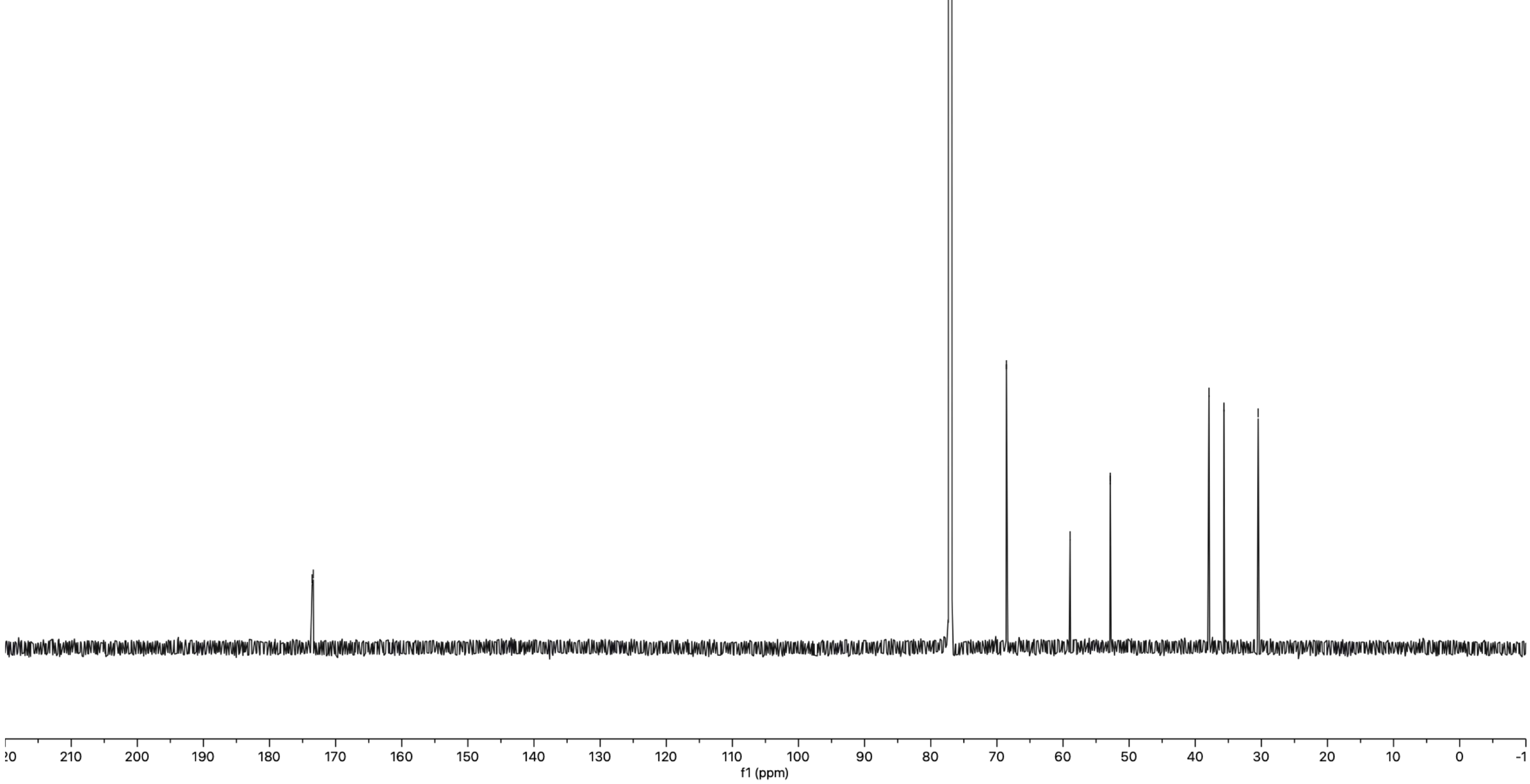

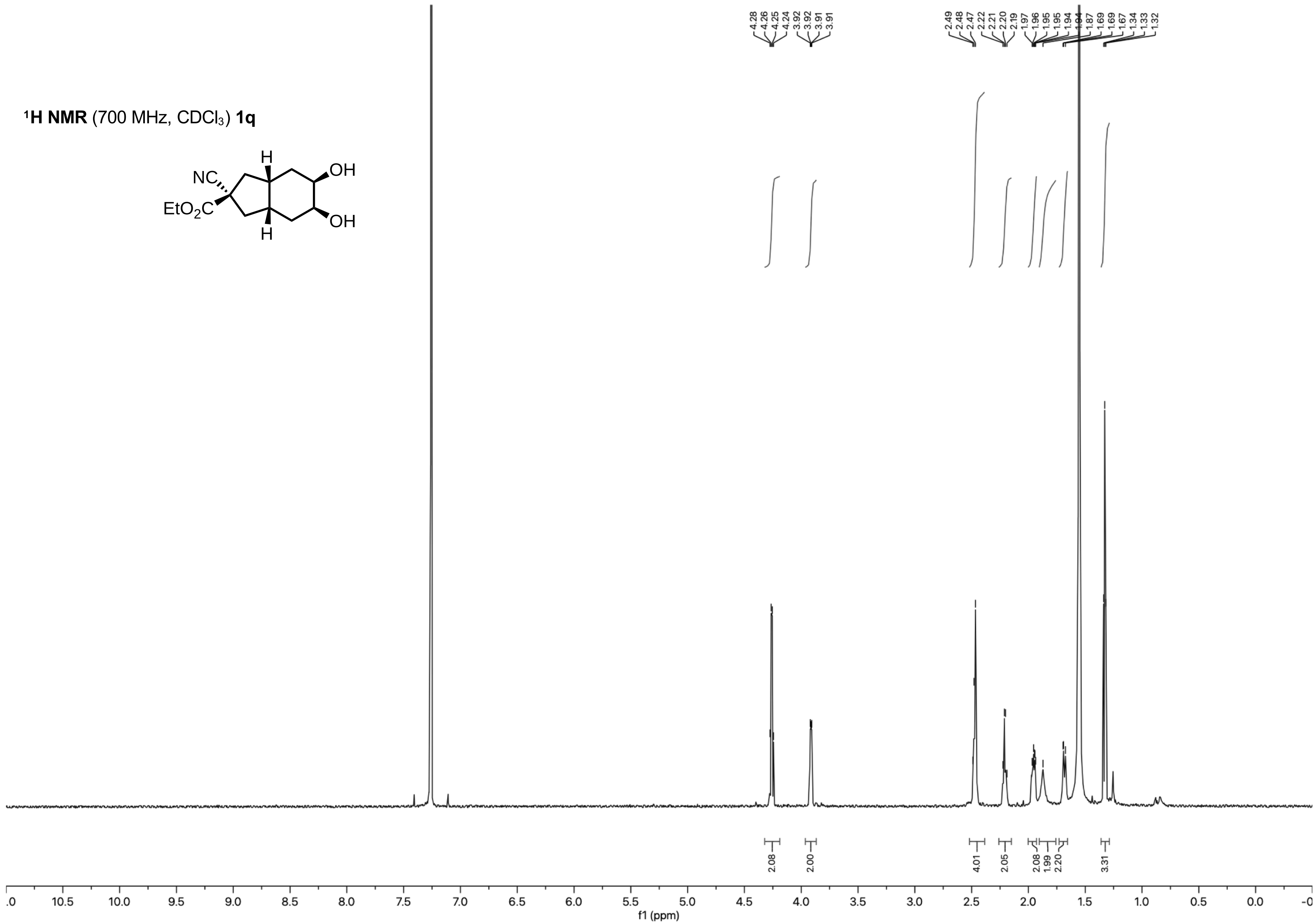

¹H NMR (700 MHz, CDCl₃) 1q
NC
EtO₂C
H
H
OH
OH
4.28 4.26 4.25 4.24 3.92 3.92 3.91 3.91
2.49 2.48 2.47 2.22 2.21 2.20 2.19 1.97 1.96 1.95 1.95 1.94 1.94 1.87 1.69 1.69 1.67 1.34 1.33 1.32
2.08 2.00 4.01 2.05 2.08 1.99 2.20 3.31
.0 10.5 10.0 9.5 9.0 8.5 8.0 7.5 7.0 6.5 6.0 5.5 5.0 4.5 4.0 3.5 3.0 2.5 2.0 1.5 1.0 0.5 0.0 -0
f1 (ppm)

^{13}C NMR (176 MHz, $CDCl_3$) **1q**

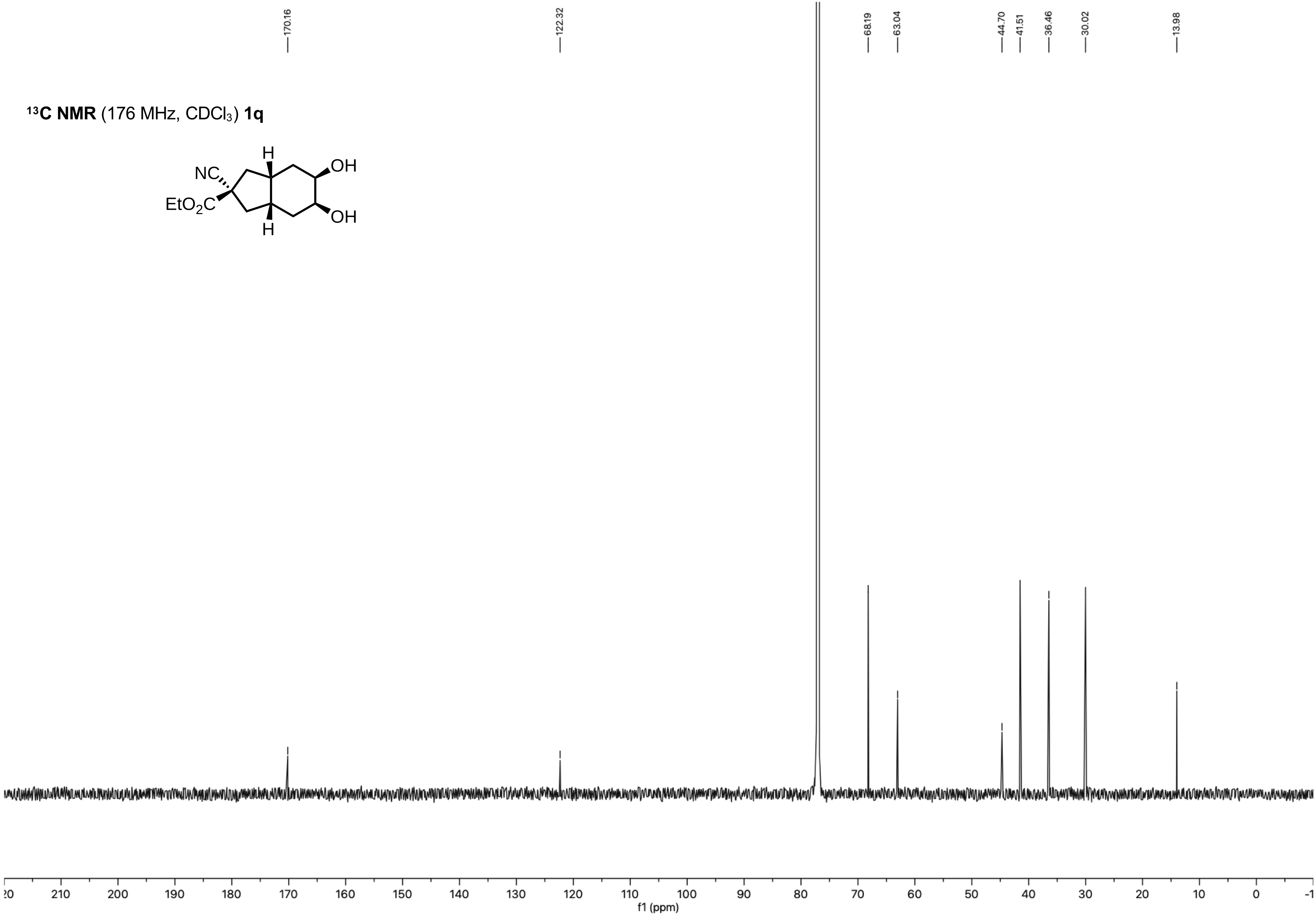

¹H NMR (700 MHz, $CDCl_3$) **1r**

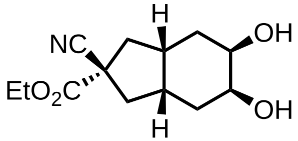

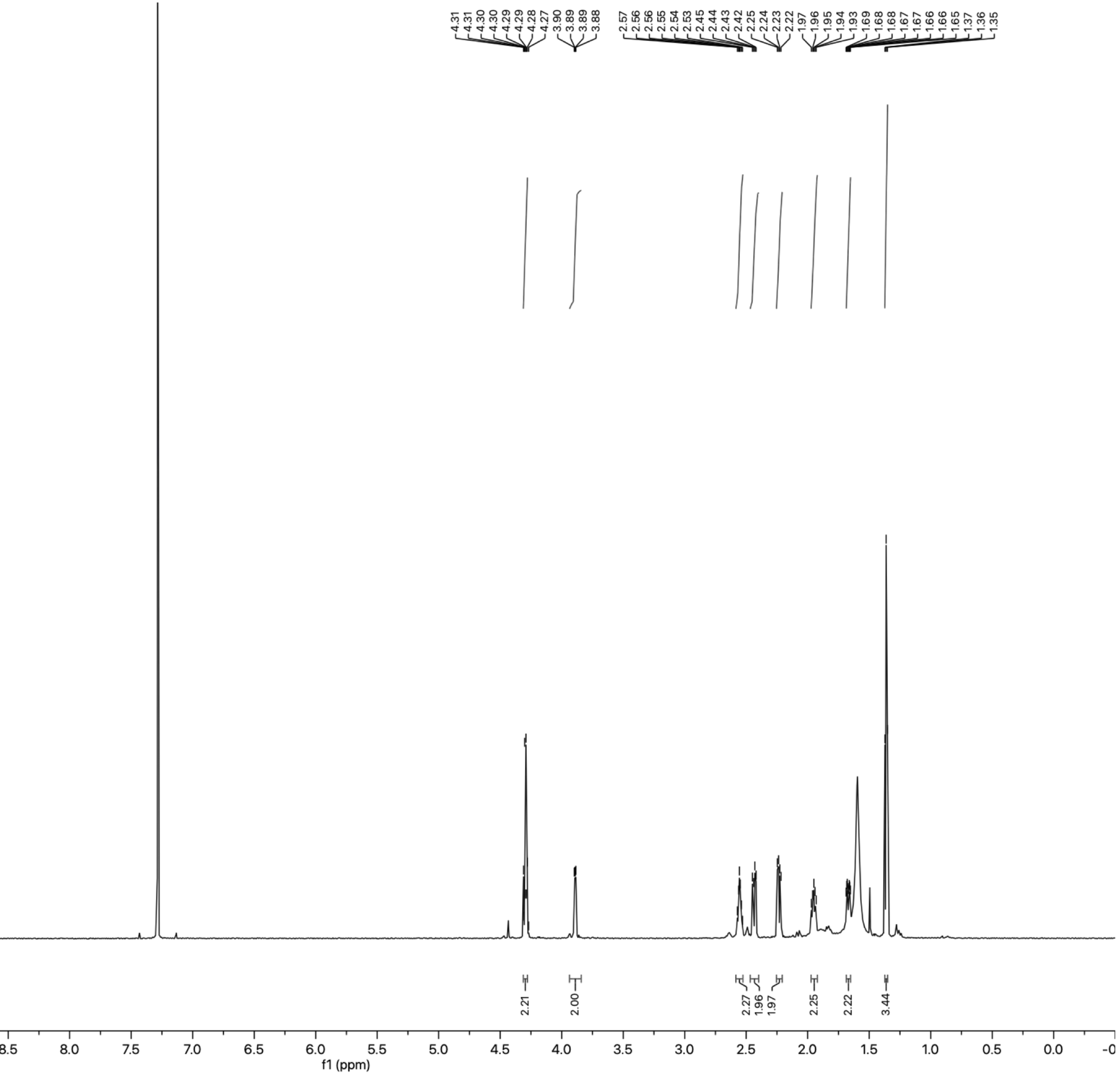

^{13}C NMR (176 MHz, $CDCl_3$) **1r**

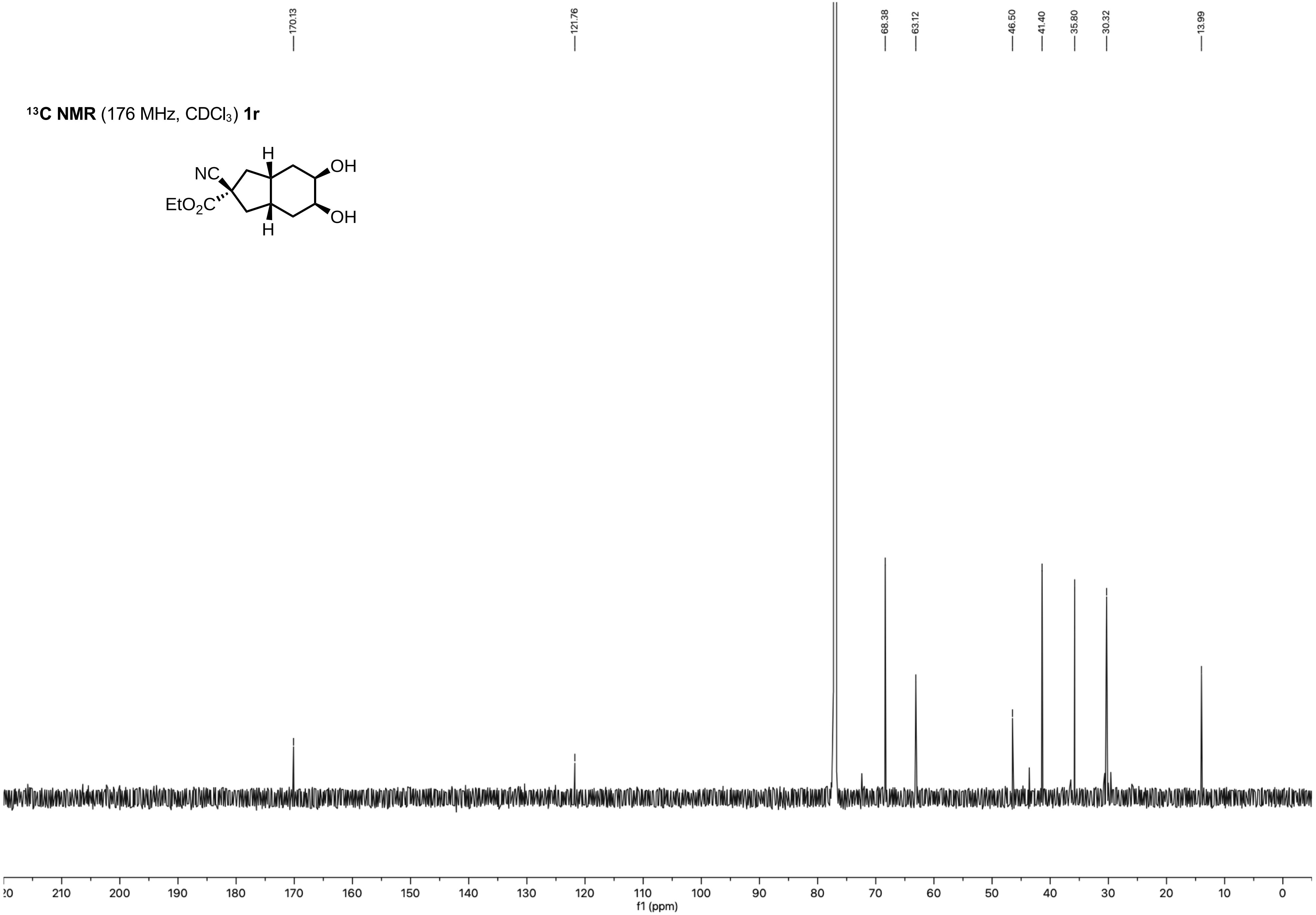

^{1}H NMR (700 MHz, $CDCl_3$) **1s**

ca. 1:1.3 mixture of epimers *

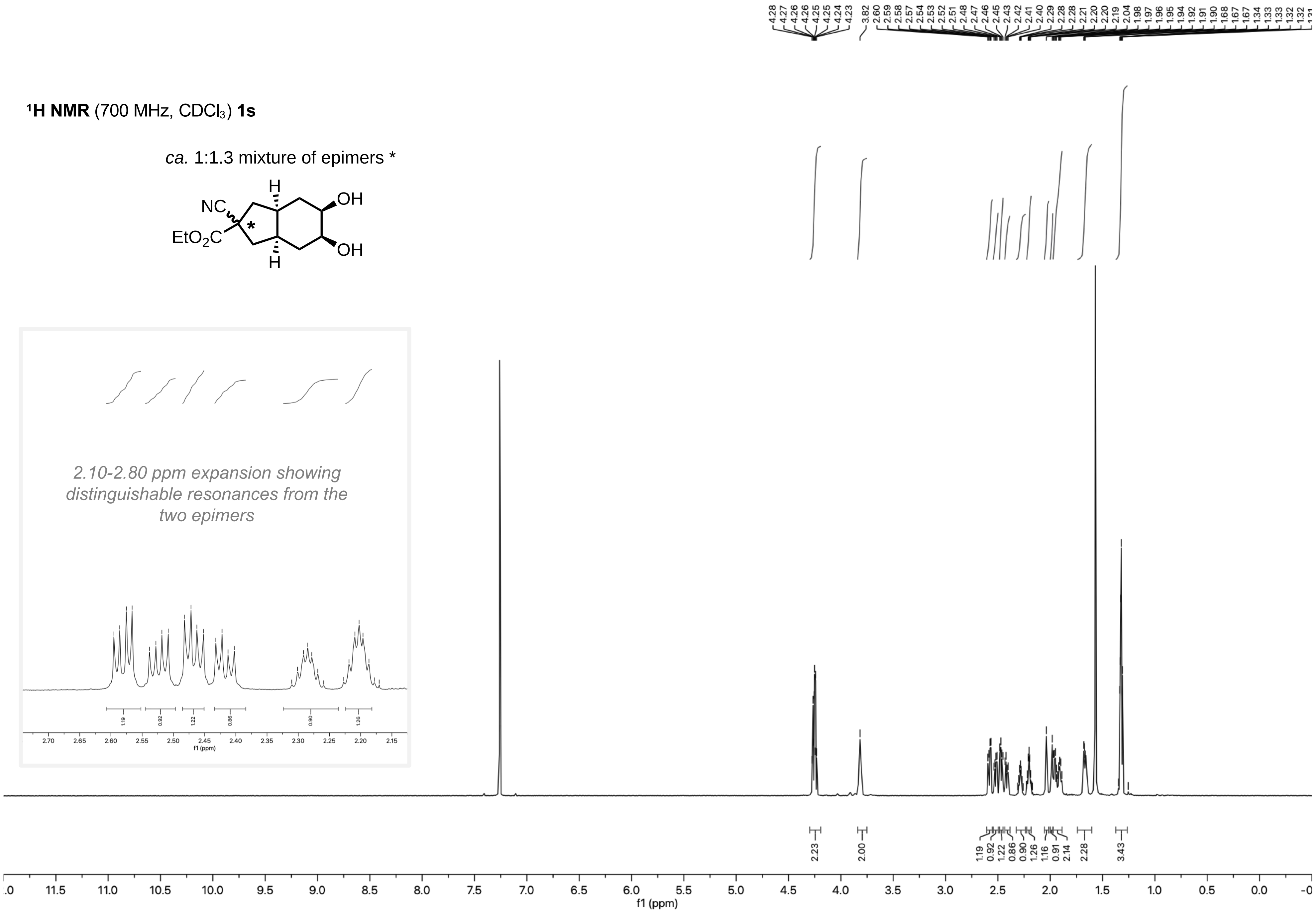

^{13}C NMR (176 MHz, $CDCl_3$) **1s**

ca. 1:1.3 mixture of epimers *

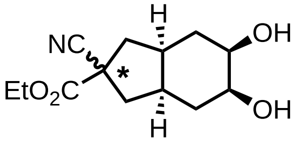

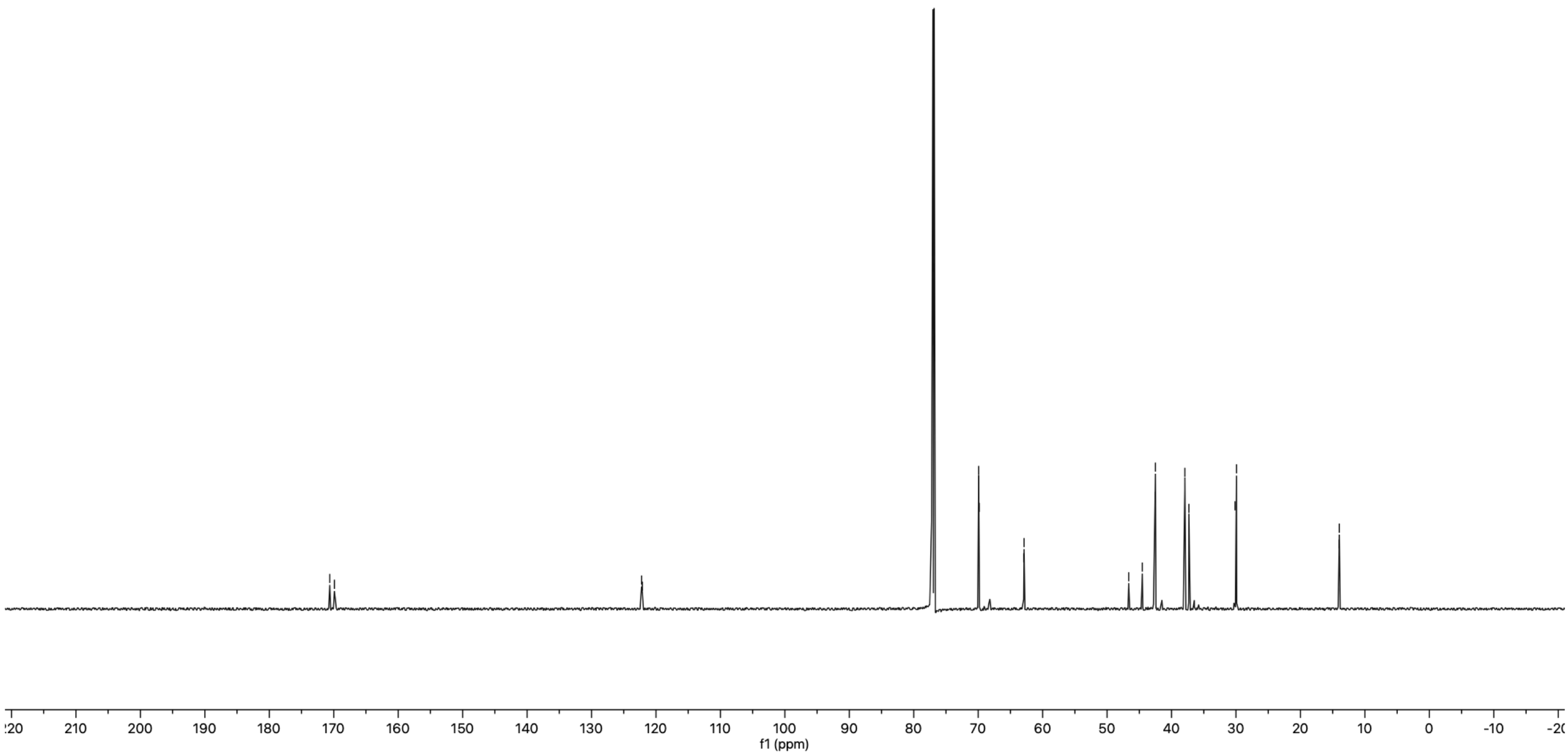

^{1}H NMR (400 MHz, d_3-MeOD) **1t**

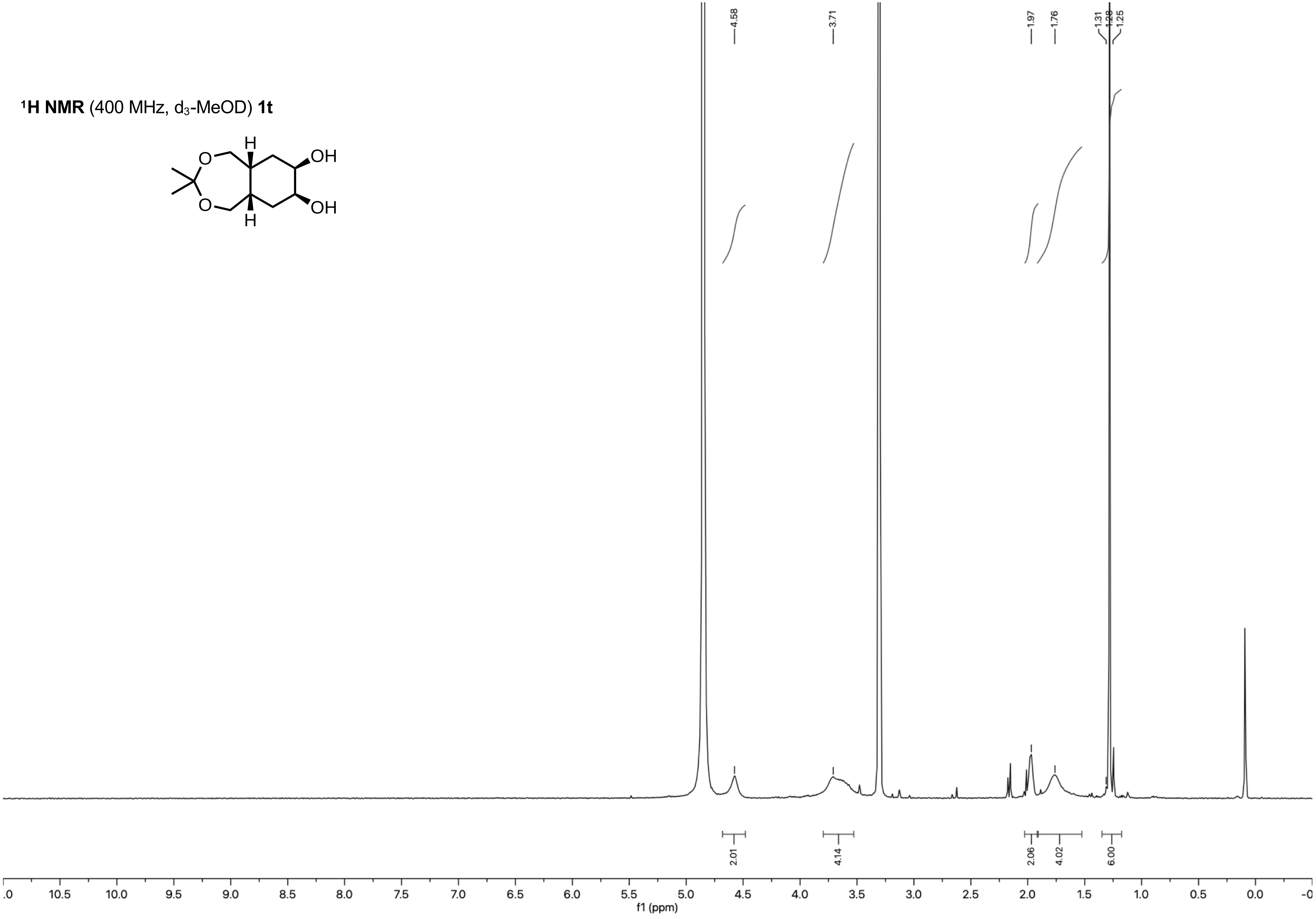

¹H NMR (400 MHz, d_3-MeOD) **1u**

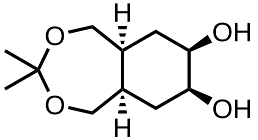

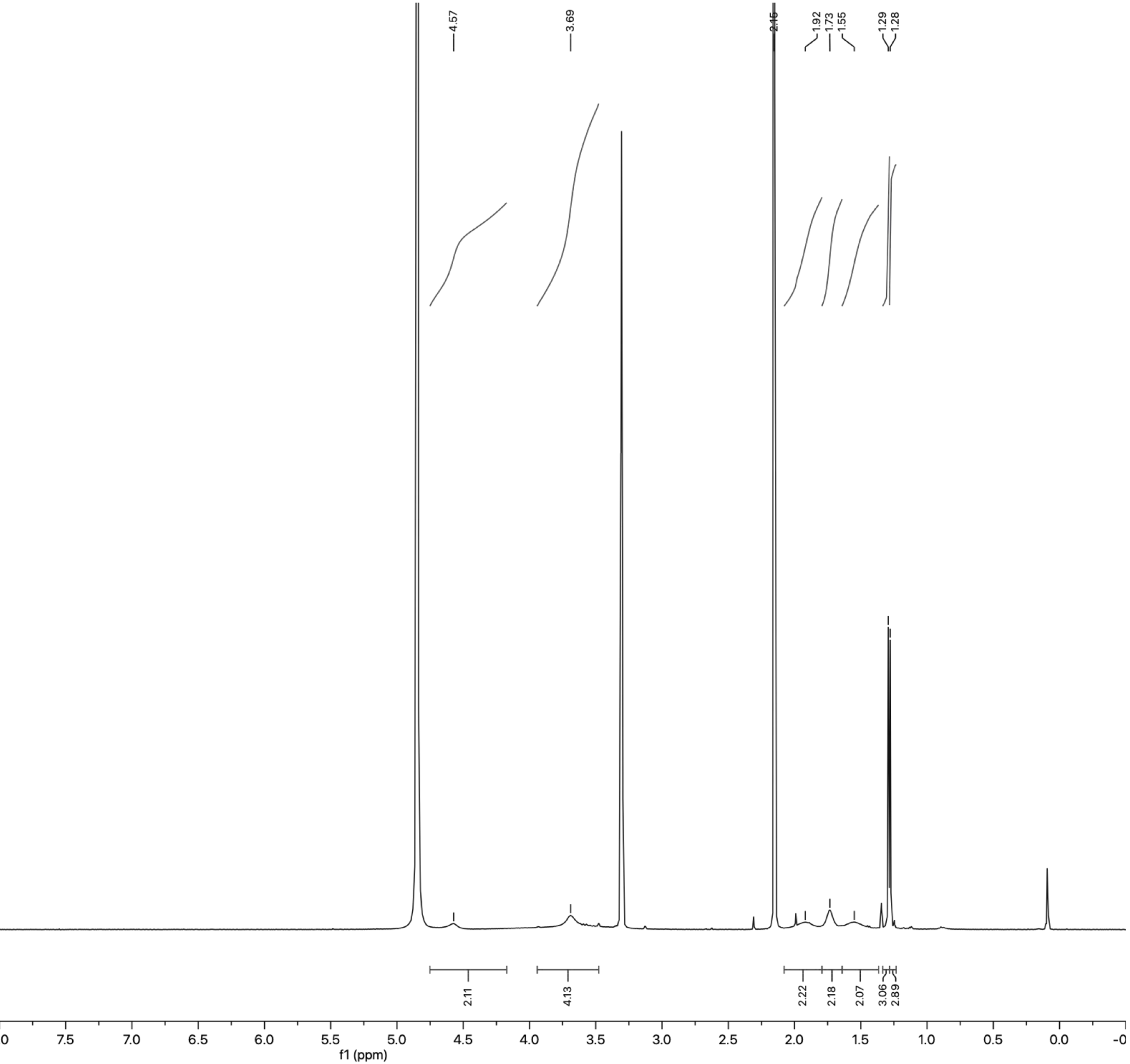

¹H NMR (700 MHz, $CDCl_3$) **1v**

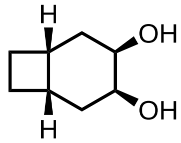

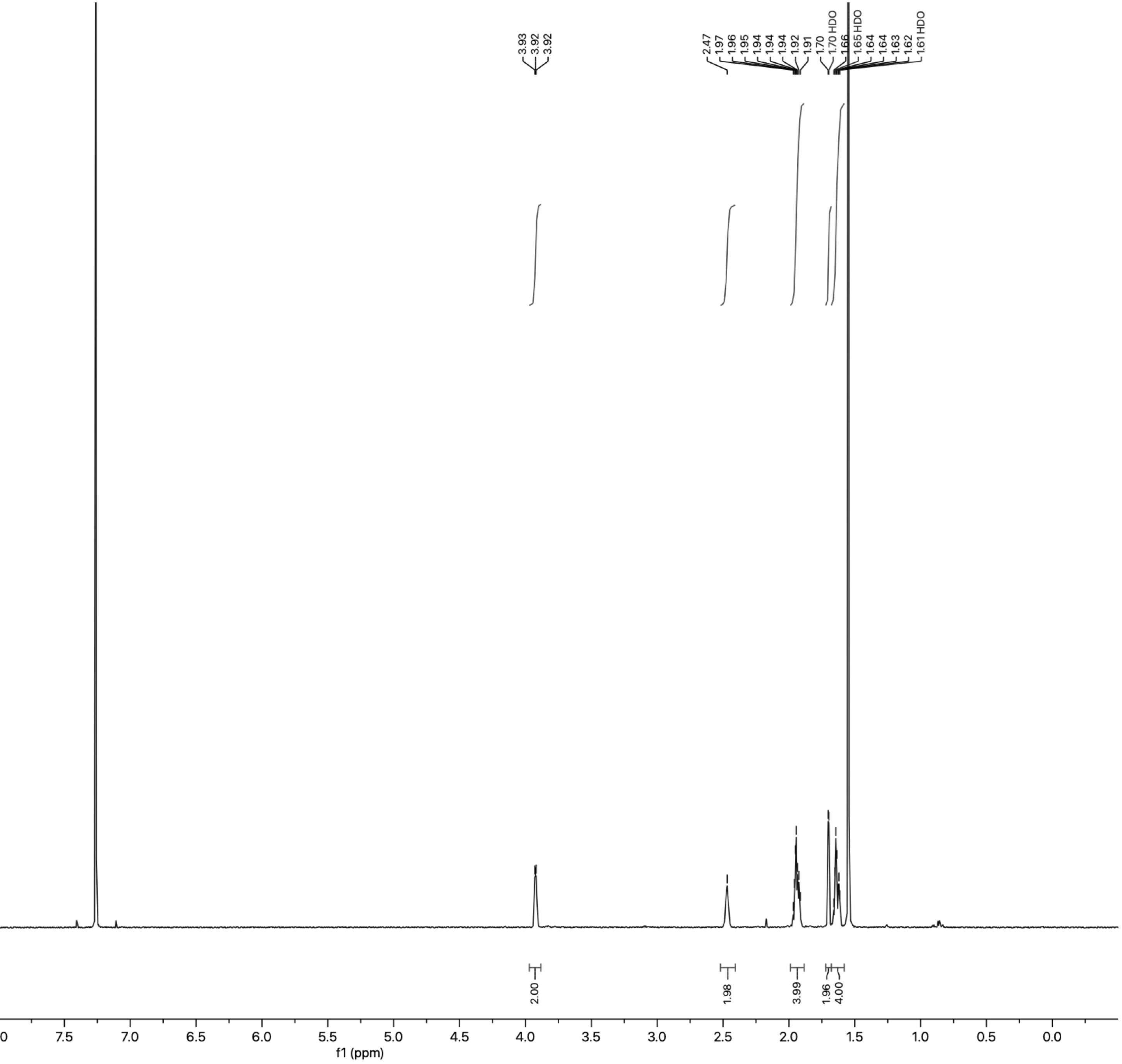

^{13}C NMR (176 MHz, $CDCl_3$) **1v**

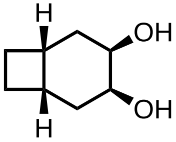

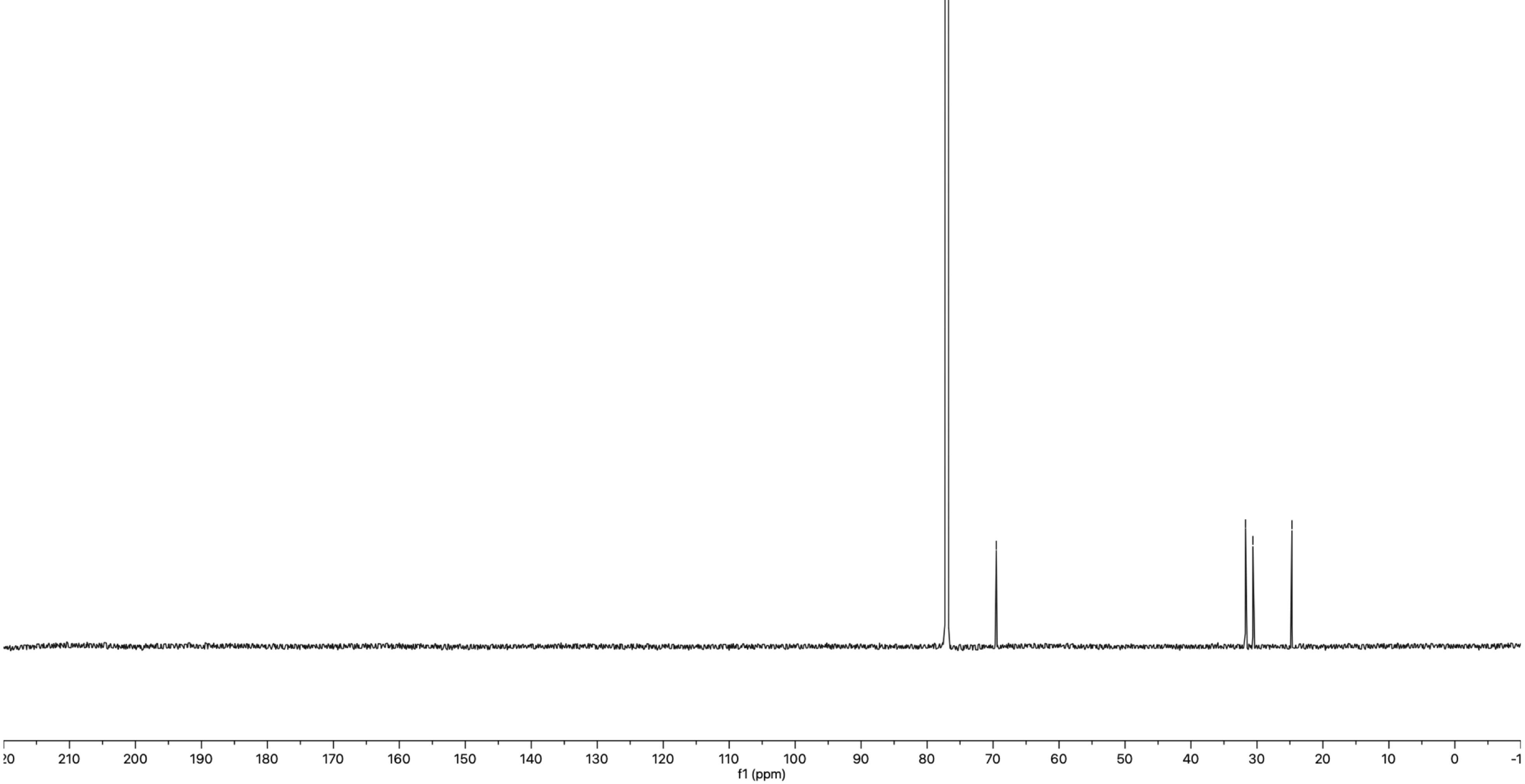

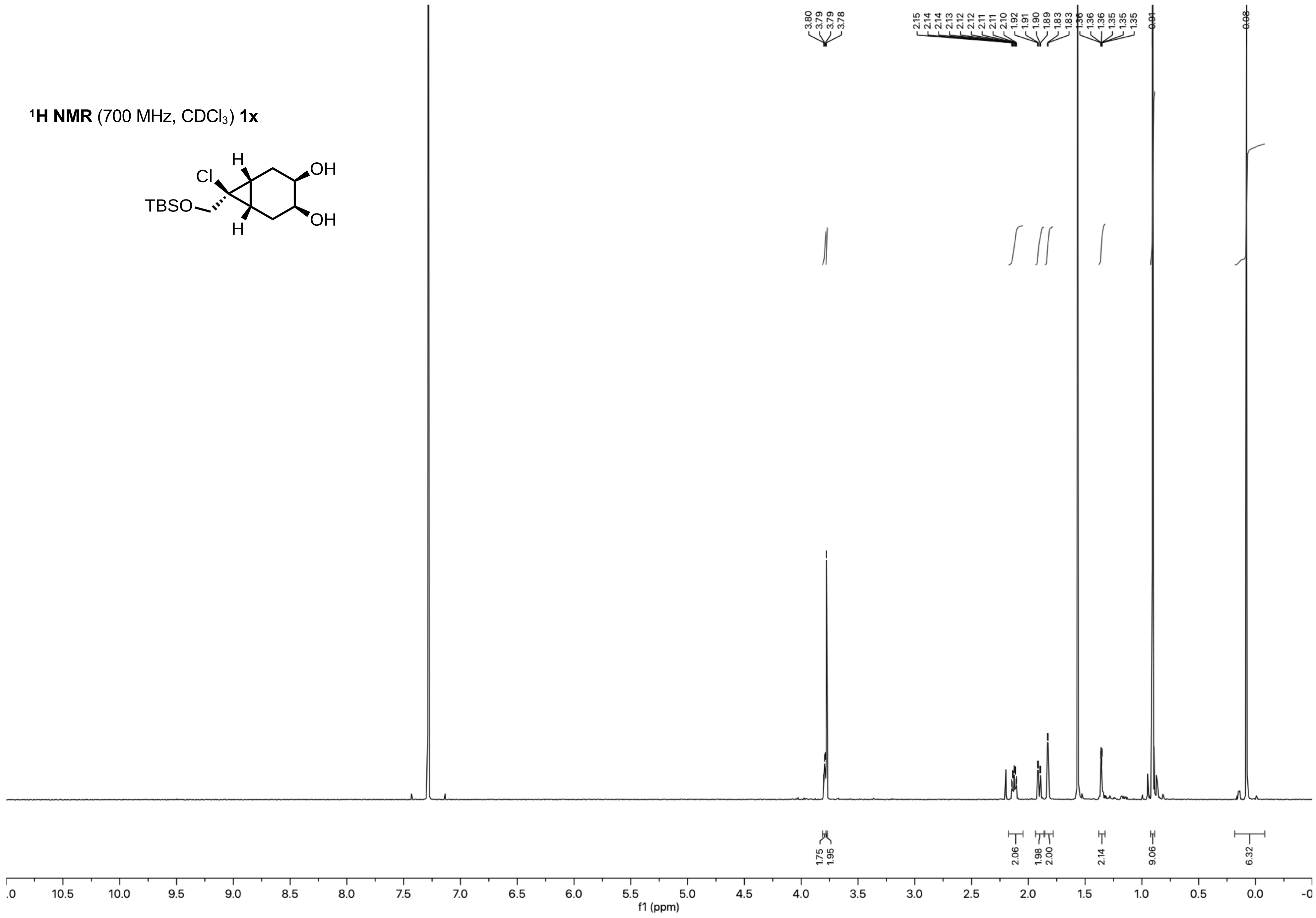

¹H NMR (700 MHz, CDCl₃) 1x
Cl
H
OH
TBSO
H
OH
3.80 3.79 3.79 3.78
2.15 2.14 2.14 2.13 2.12 2.12 2.11 2.11 2.10 1.92 1.91 1.90 1.89 1.83 1.83 1.36 1.36 1.36 1.35 1.35 1.35
0.91
0.08
1.75 1.95
2.06 1.98 2.00
2.14
9.06
6.32
.0 10.5 10.0 9.5 9.0 8.5 8.0 7.5 7.0 6.5 6.0 5.5 5.0 4.5 4.0 3.5 3.0 2.5 2.0 1.5 1.0 0.5 0.0 -0
f1 (ppm)

^{13}C NMR (176 MHz, $CDCl_3$) **1x**

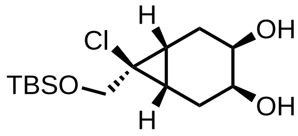

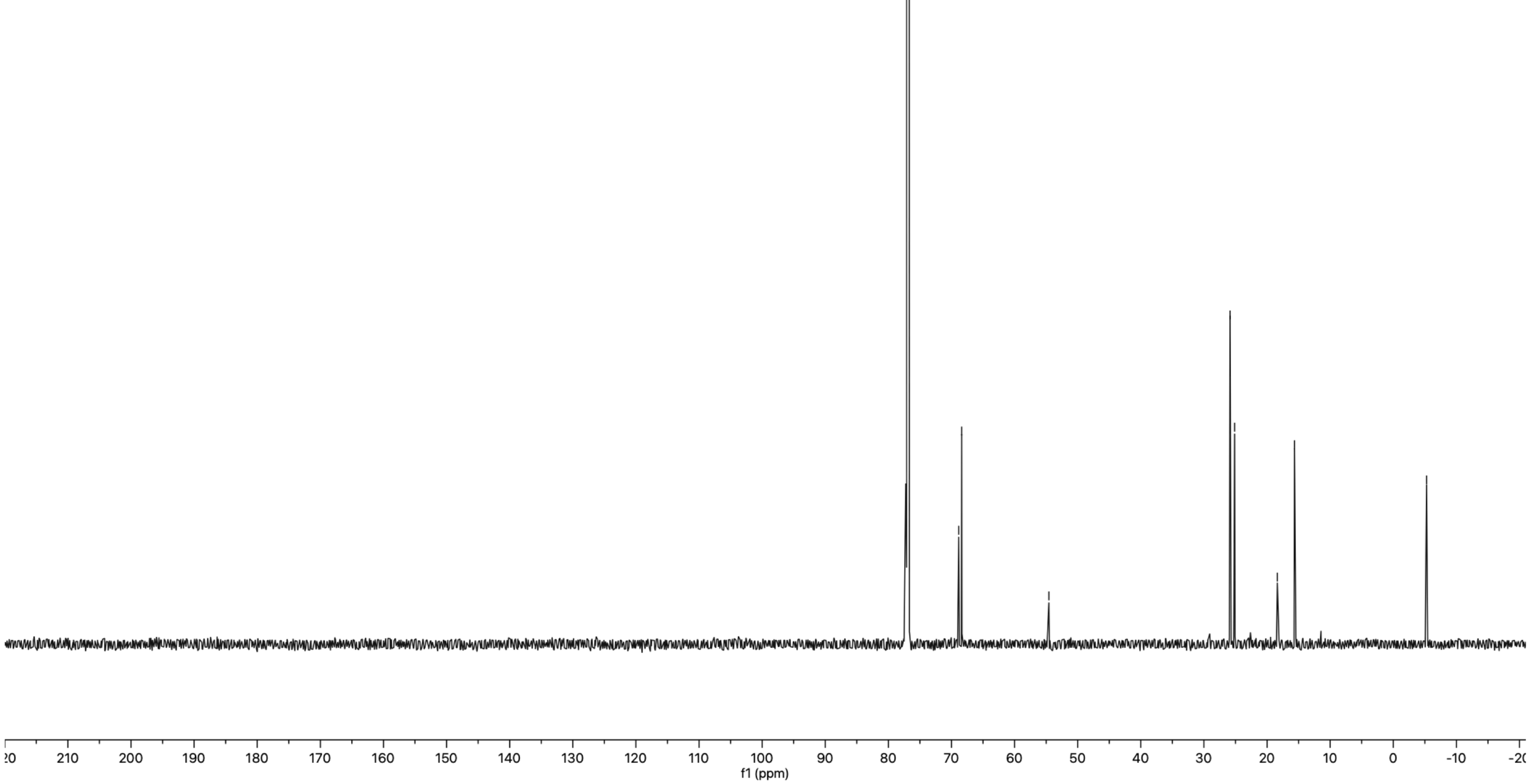

^{1}**H NMR** (700 MHz, $CDCl_3$) **1z**

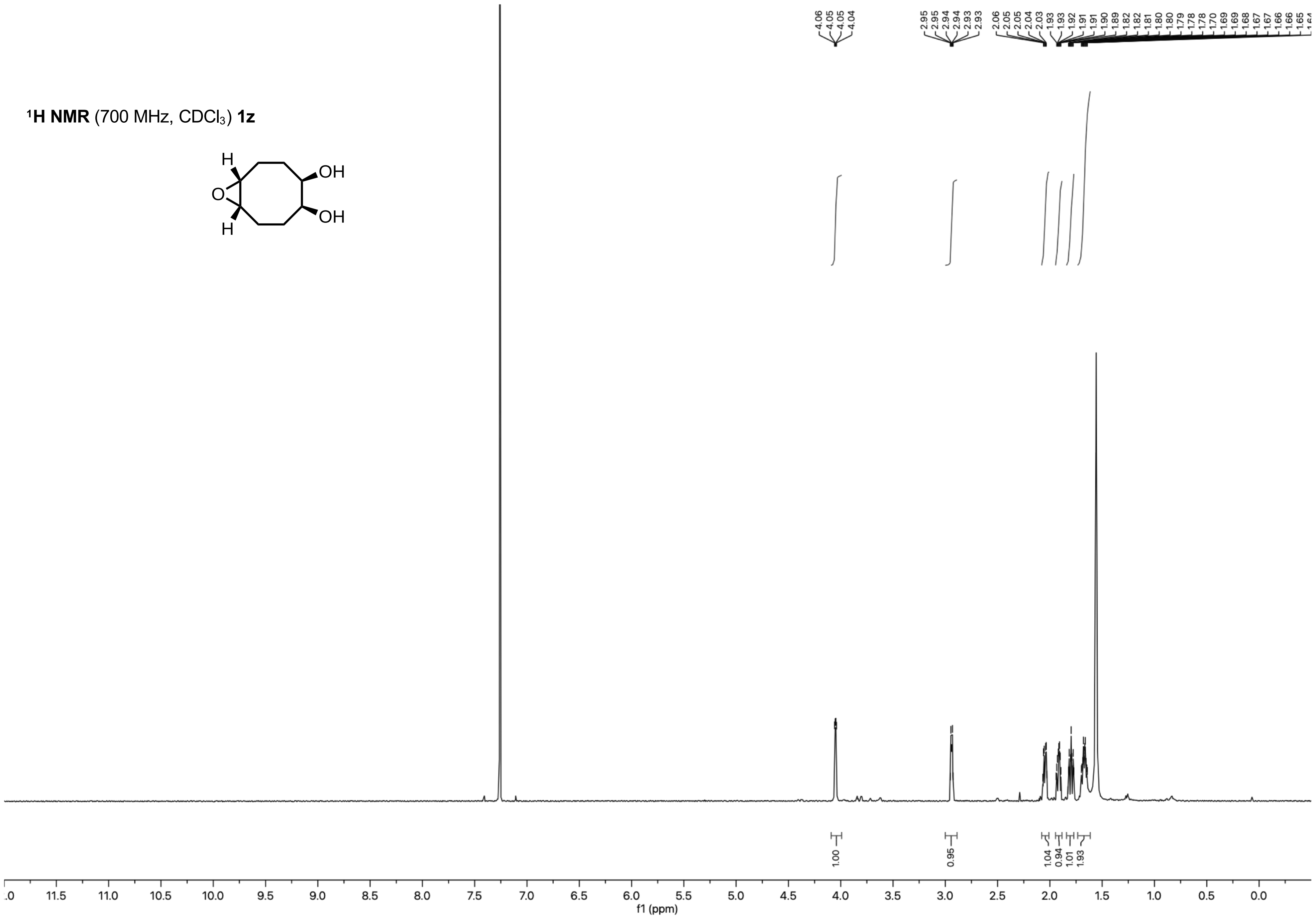

^{13}C NMR (176 MHz, $CDCl_3$) **1z**

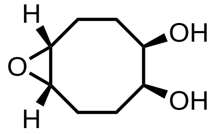

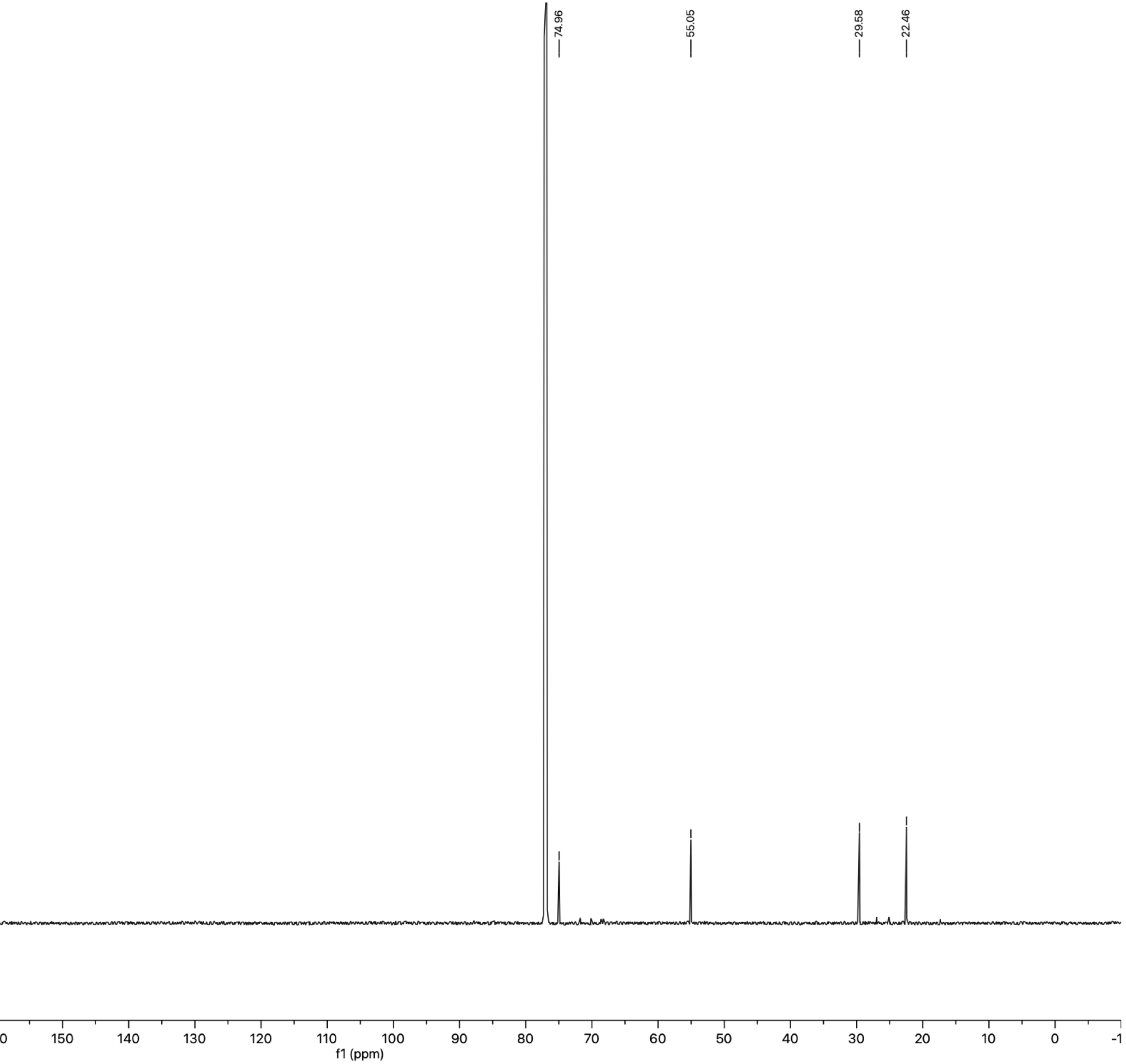

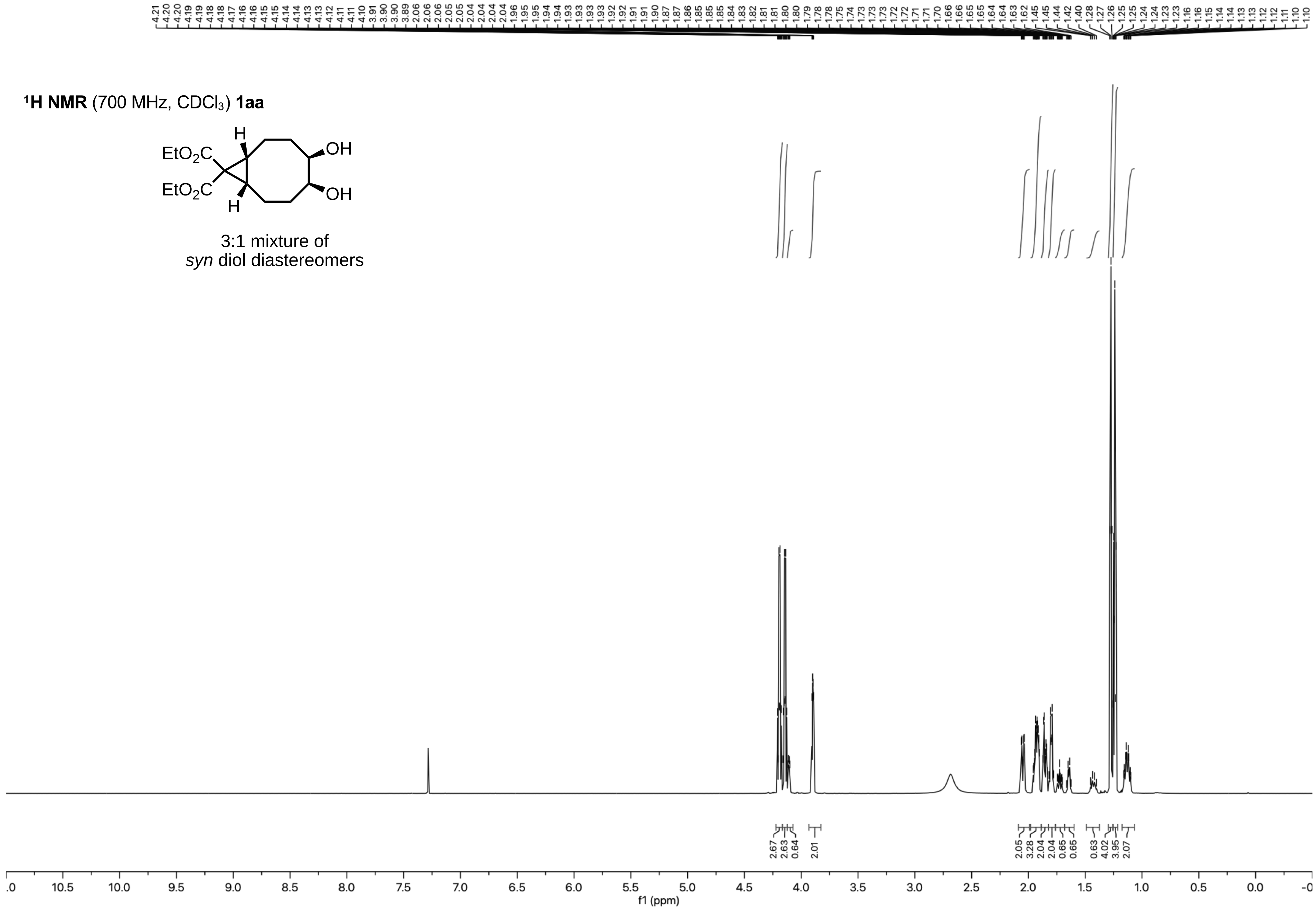

¹H NMR (700 MHz, CDCl₃) 1aa
EtO₂C
EtO₂C
H
H
OH
OH
3:1 mixture of
syn diol diastereomers
2.67
2.63
0.64
2.01
2.05
3.28
2.04
2.04
0.65
0.65
0.63
4.02
3.95
2.07
f1 (ppm)

^{13}C NMR (176 MHz, $CDCl_3$) **1aa**

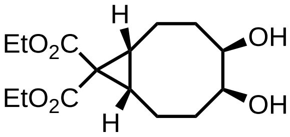

3:1 mixture of
syn diol diastereomers

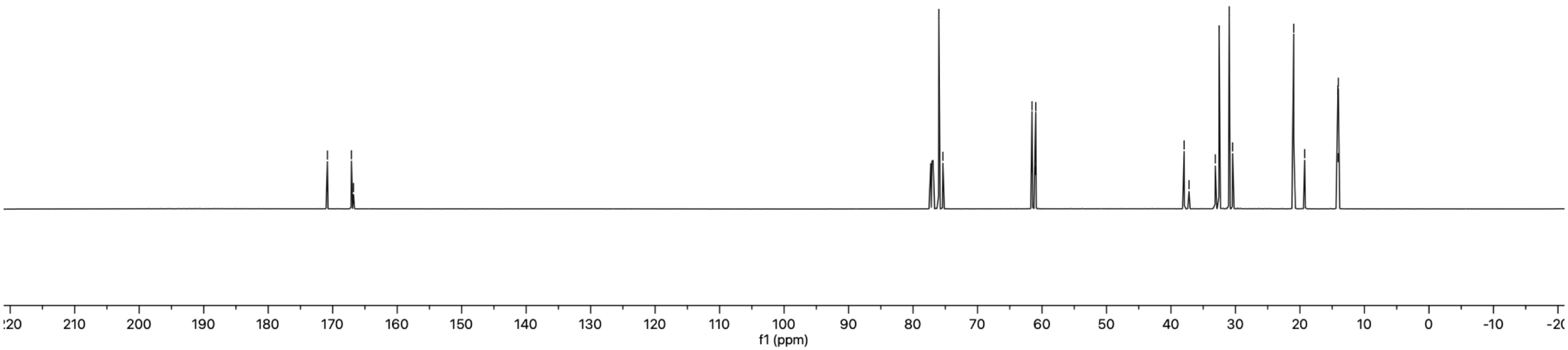

^{1}H NMR (700 MHz, $CDCl_3$) **1ab**

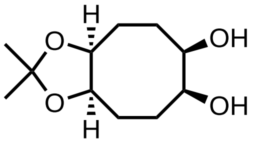

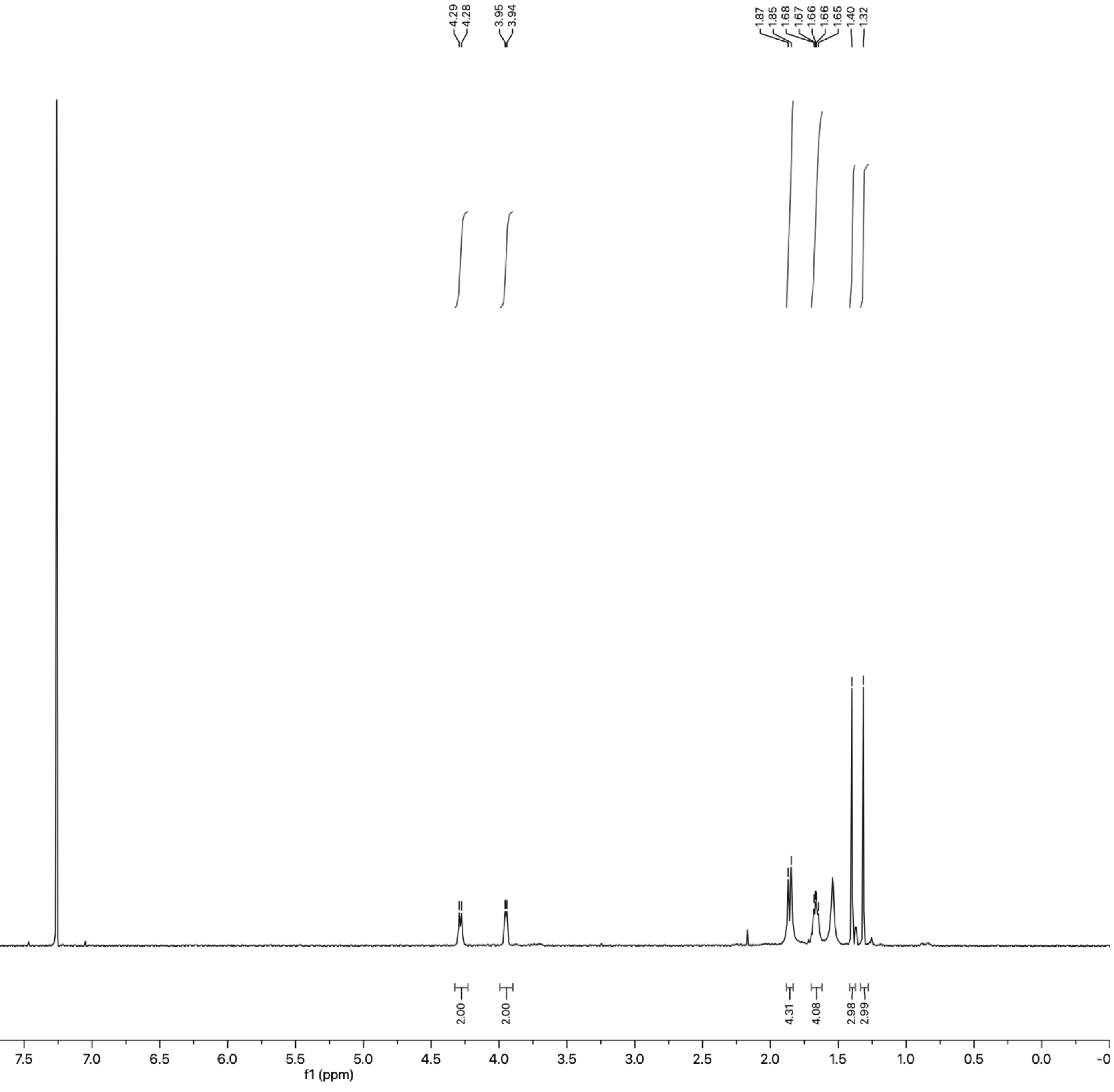

^{13}C NMR (176 MHz, $CDCl_3$) **1ab**

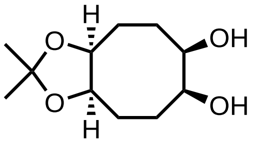

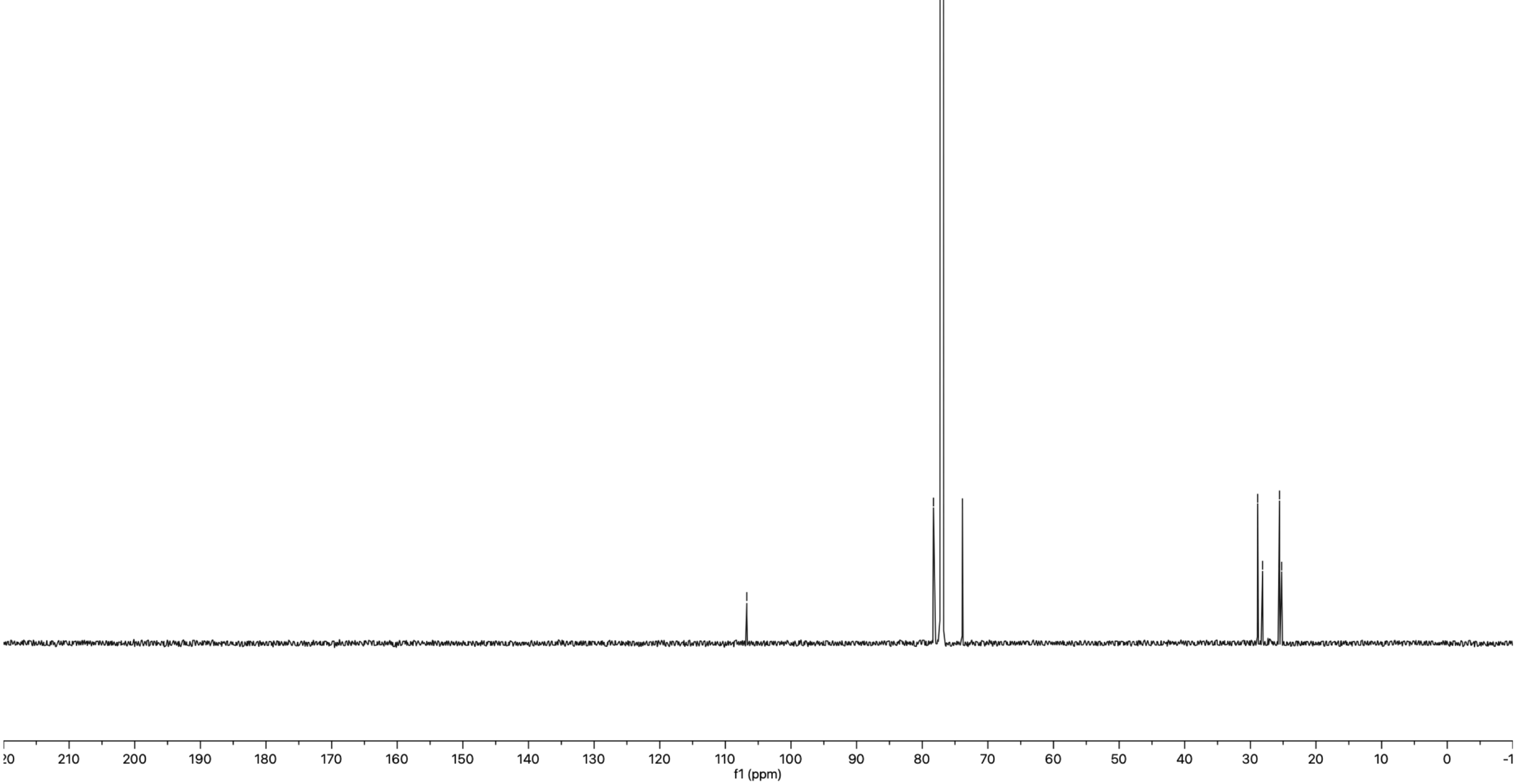

¹H NMR (700 MHz, CDCl$_3$) **1ac**

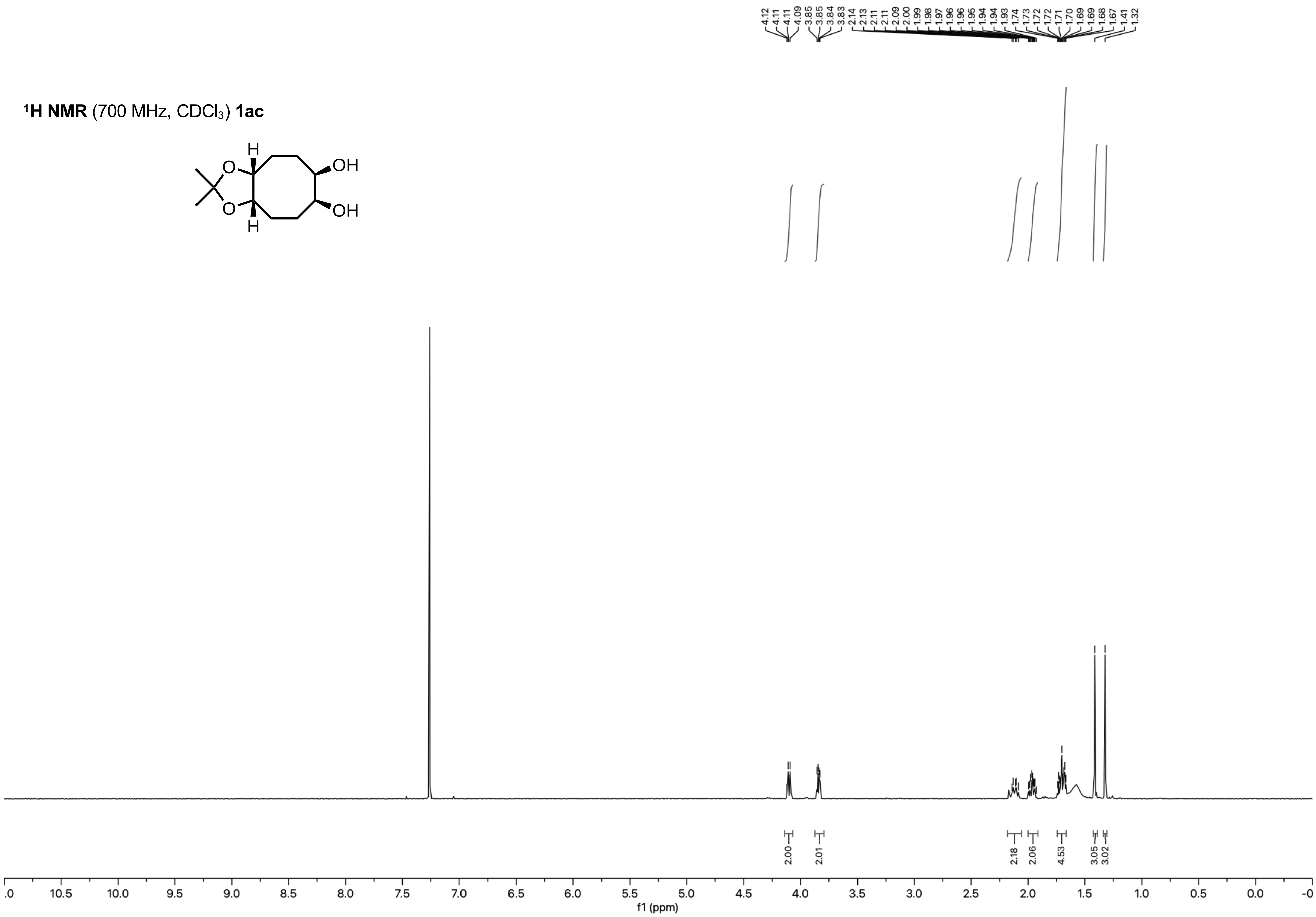

^{13}C NMR (176 MHz, $CDCl_3$) **1ac**

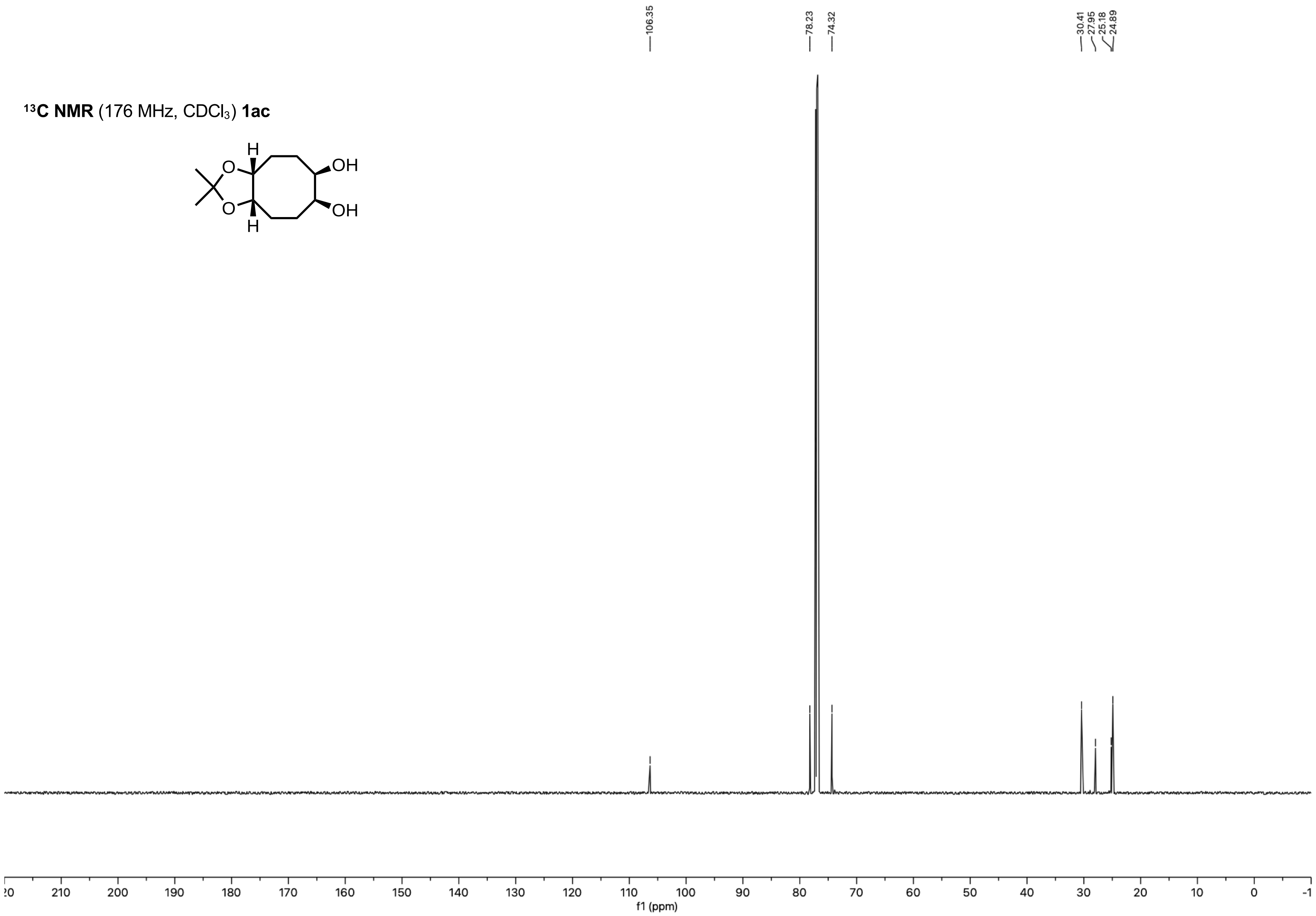

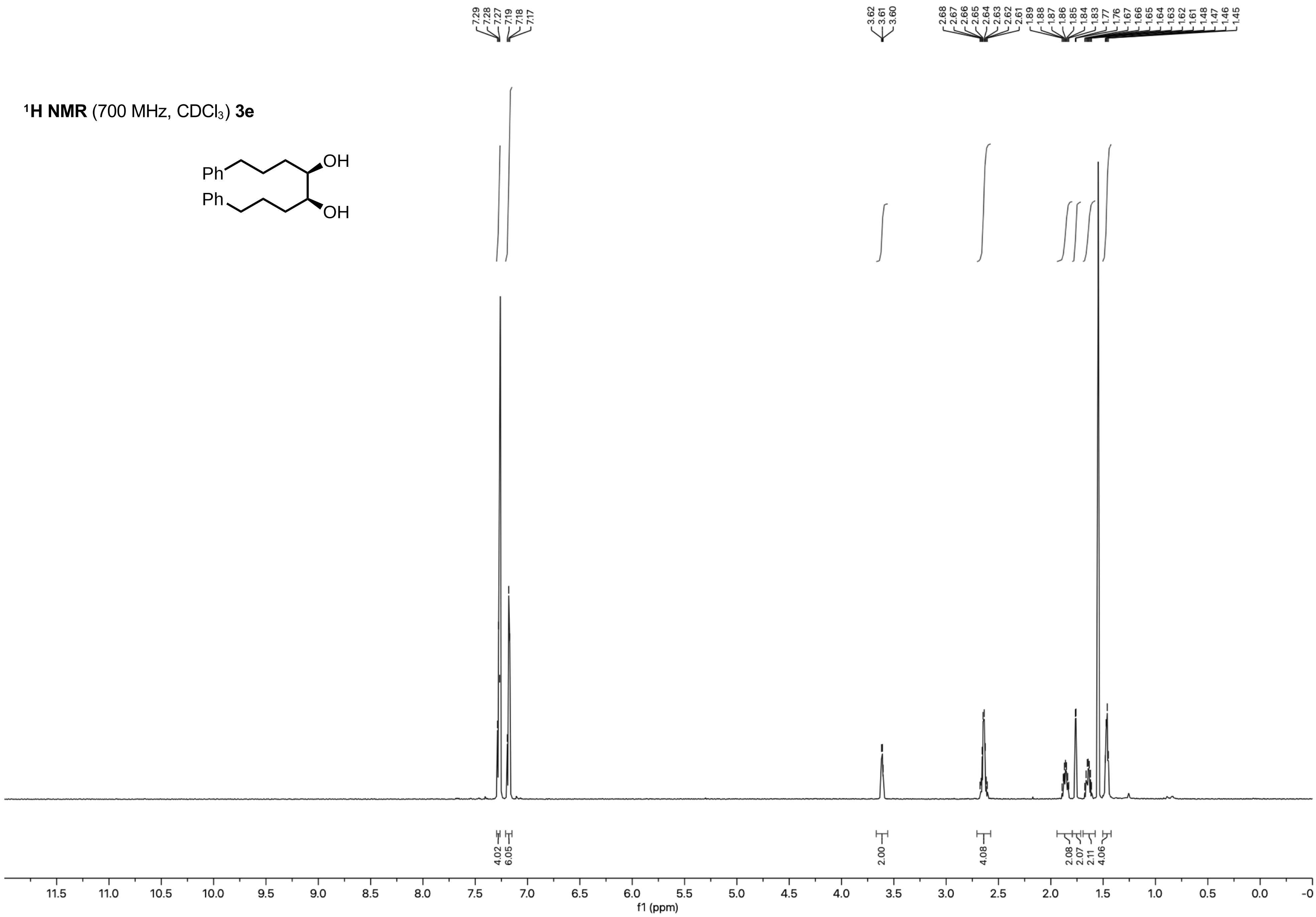
¹H NMR (700 MHz, CDCl₃) 3e
Ph
Ph
OH
OH
7.29
7.28
7.27
7.19
7.18
7.17
3.62
3.61
3.60
2.68
2.67
2.66
2.65
2.64
2.63
2.62
2.61
1.89
1.88
1.87
1.86
1.85
1.84
1.83
1.77
1.76
1.67
1.66
1.65
1.64
1.63
1.62
1.61
1.48
1.47
1.46
1.45
4.02
6.05
2.00
4.08
2.08
2.07
2.11
4.06
11.5
11.0
10.5
10.0
9.5
9.0
8.5
8.0
7.5
7.0
6.5
6.0
5.5
5.0
4.5
4.0
3.5
3.0
2.5
2.0
1.5
1.0
0.5
0.0
-0
f1 (ppm)

^{13}C NMR (176 MHz, $CDCl_3$) **3e**

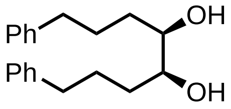

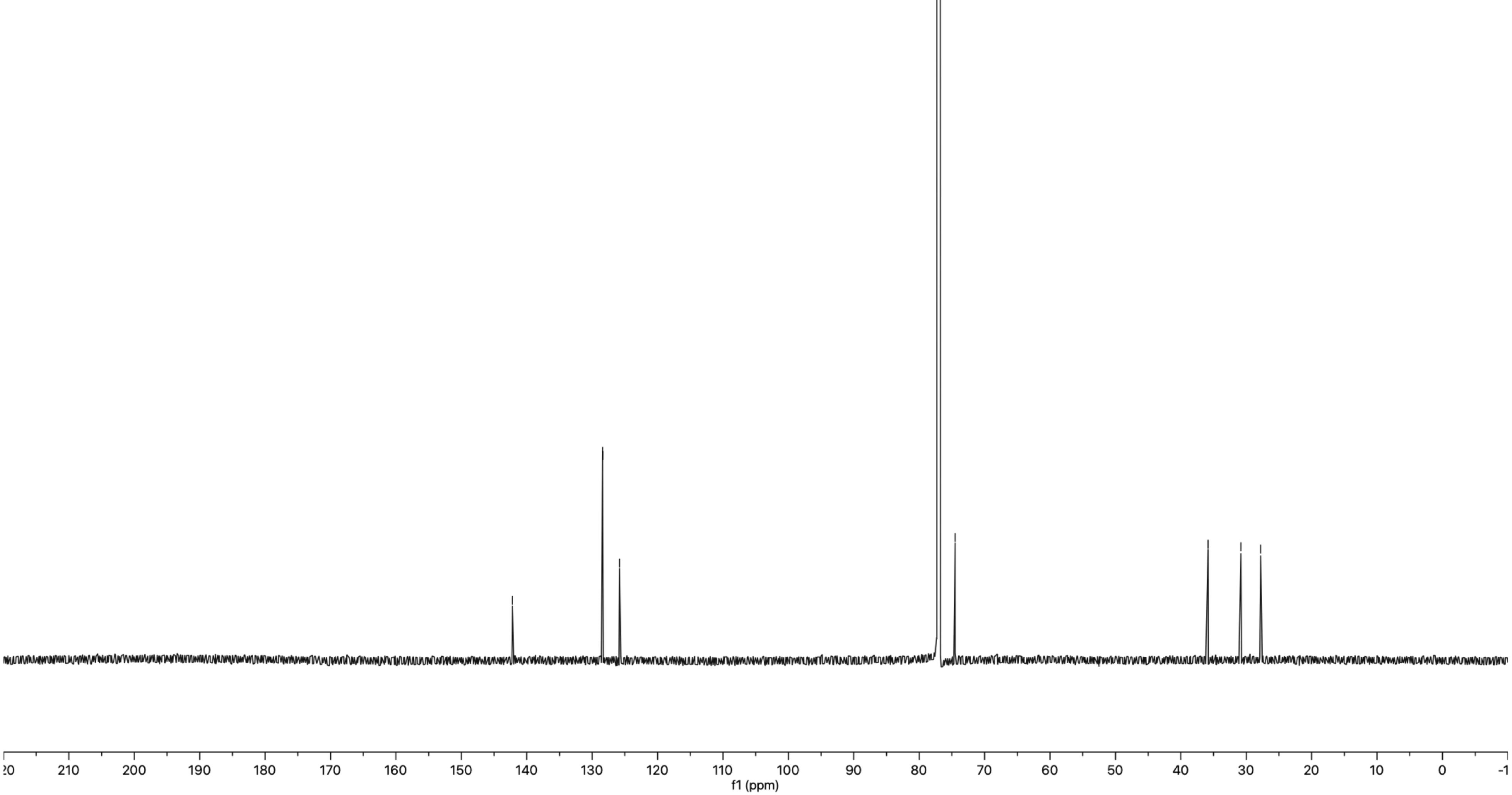

¹H NMR (700 MHz, $CDCl_3$) **3h**

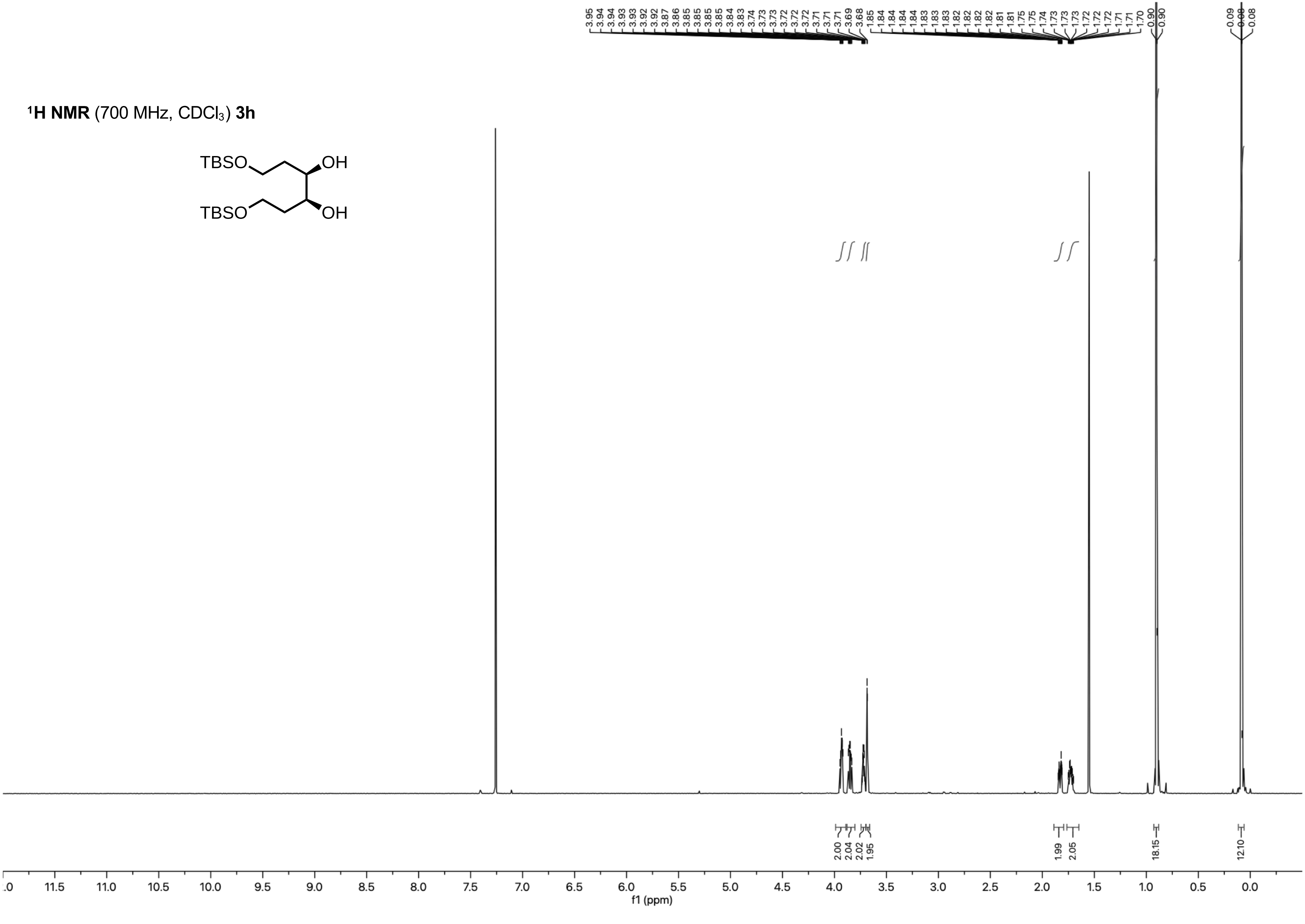

^{13}**C NMR** (176 MHz, $CDCl_3$) **3h**

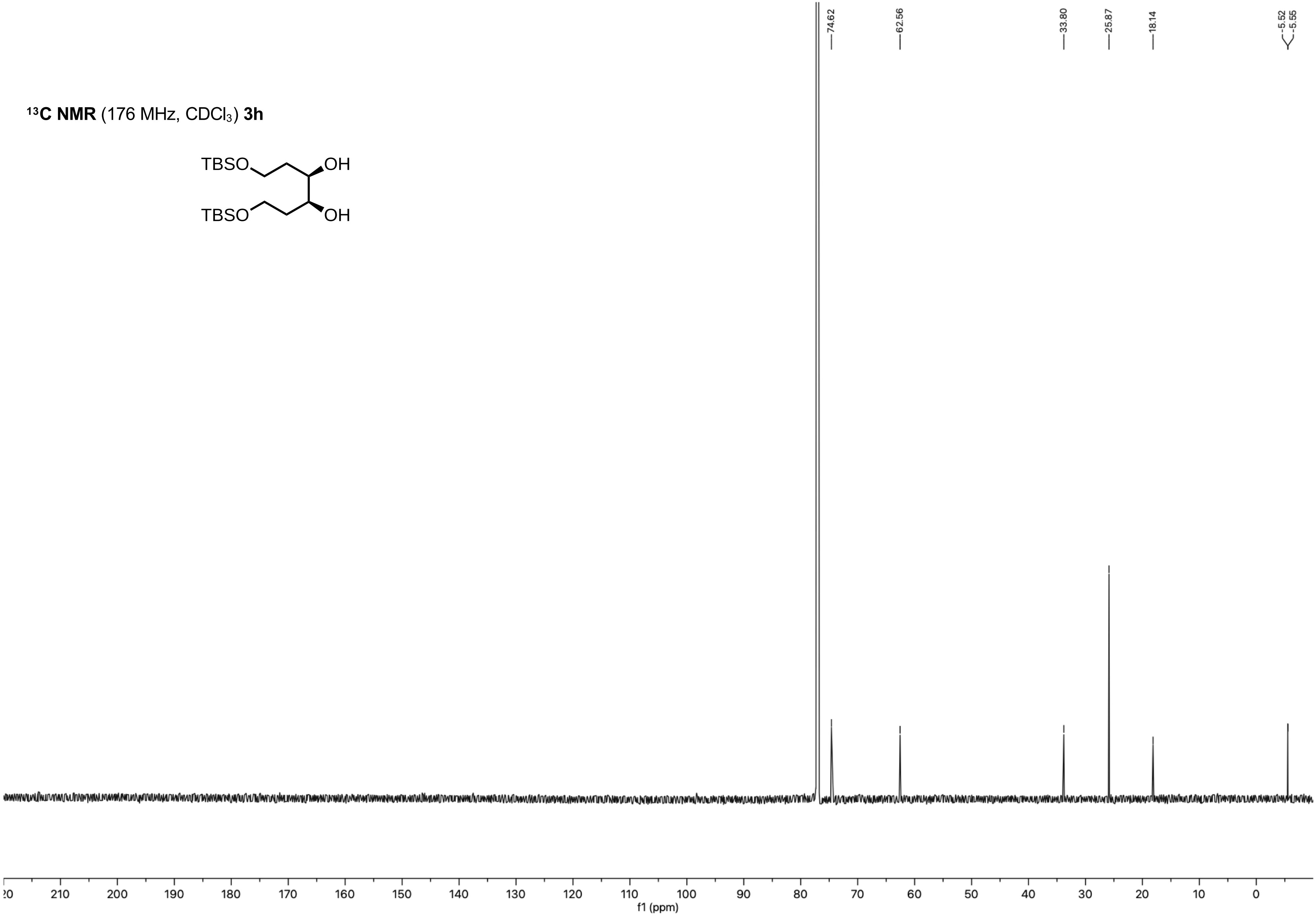

¹H NMR (700 MHz, $CDCl_3$) **3g**

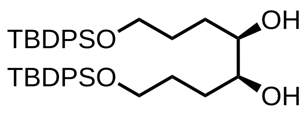

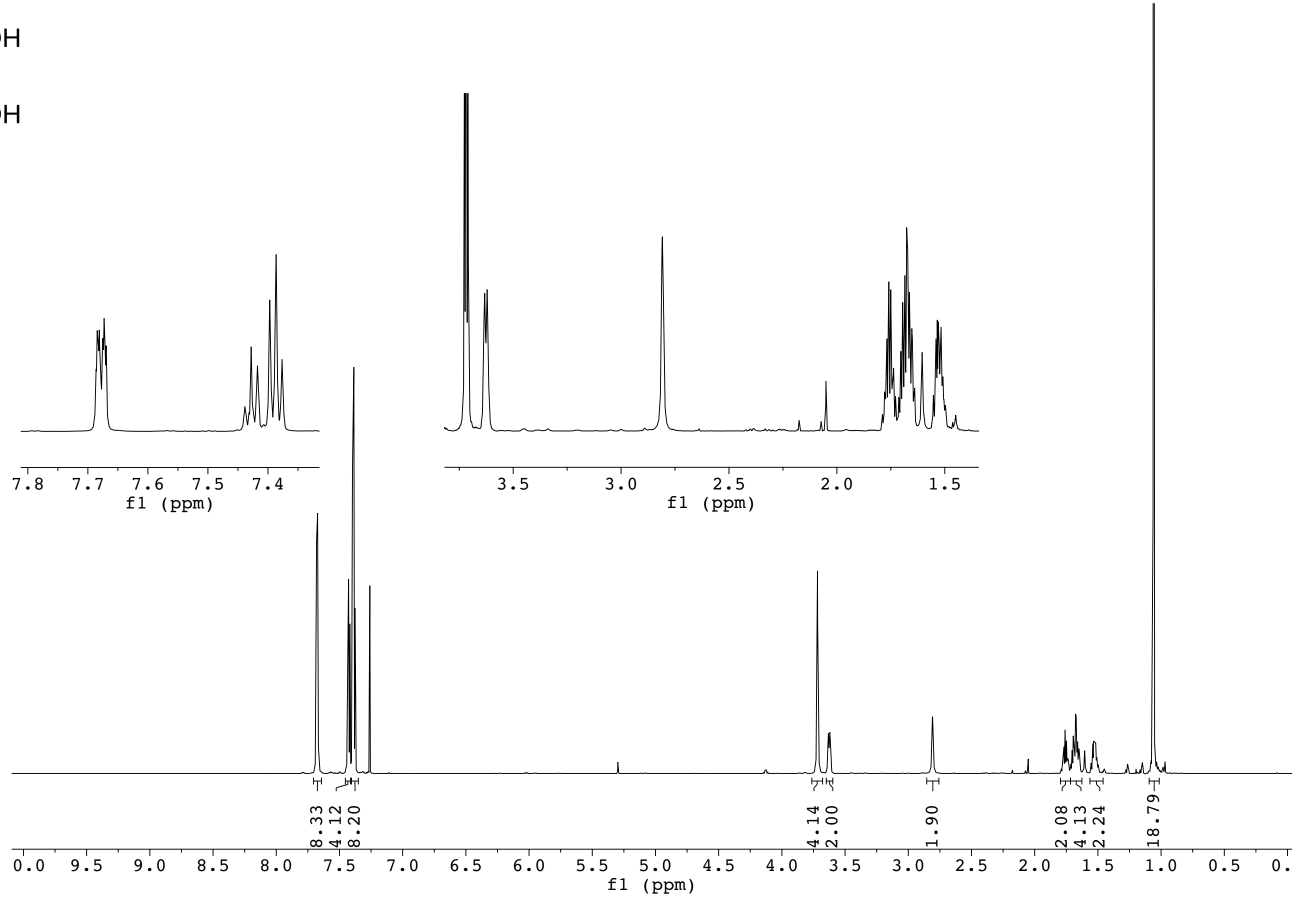

^{13}C NMR (176 MHz, $CDCl_3$) **3g**

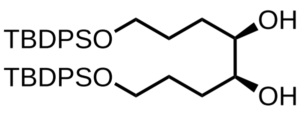

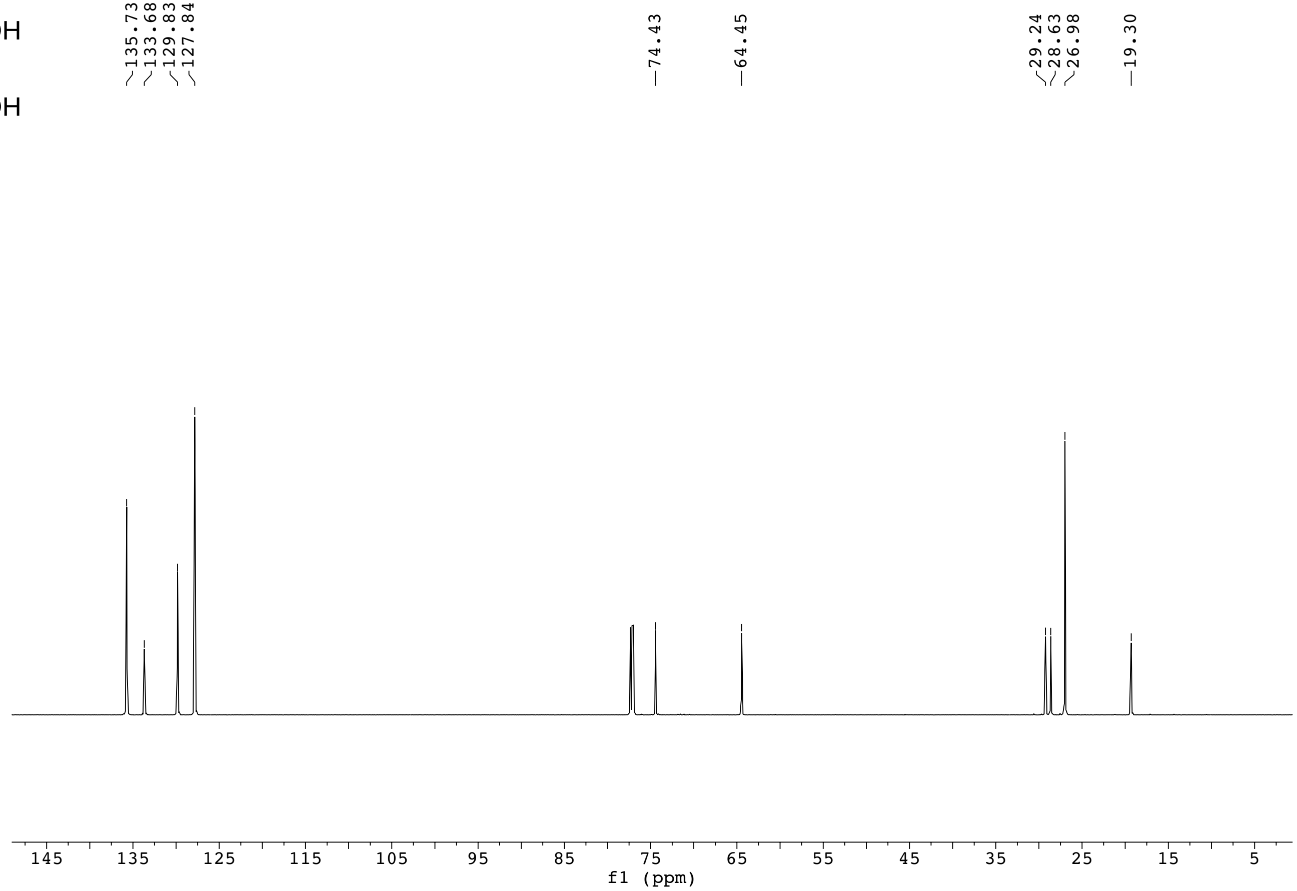

^{1}H NMR (700 MHz, $CDCl_3$) **3i**

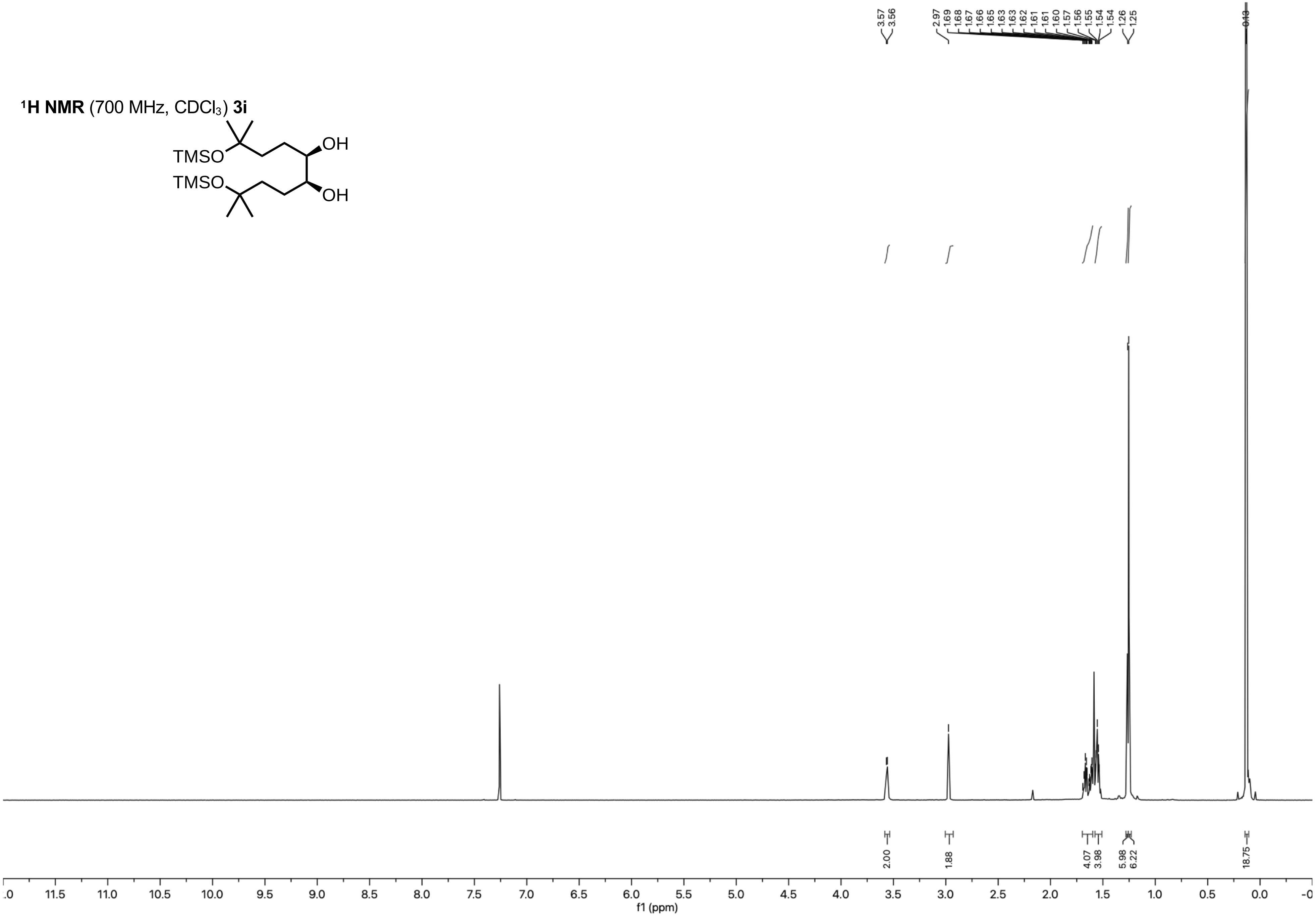

^{13}C NMR (176 MHz, $CDCl_3$) **3i**

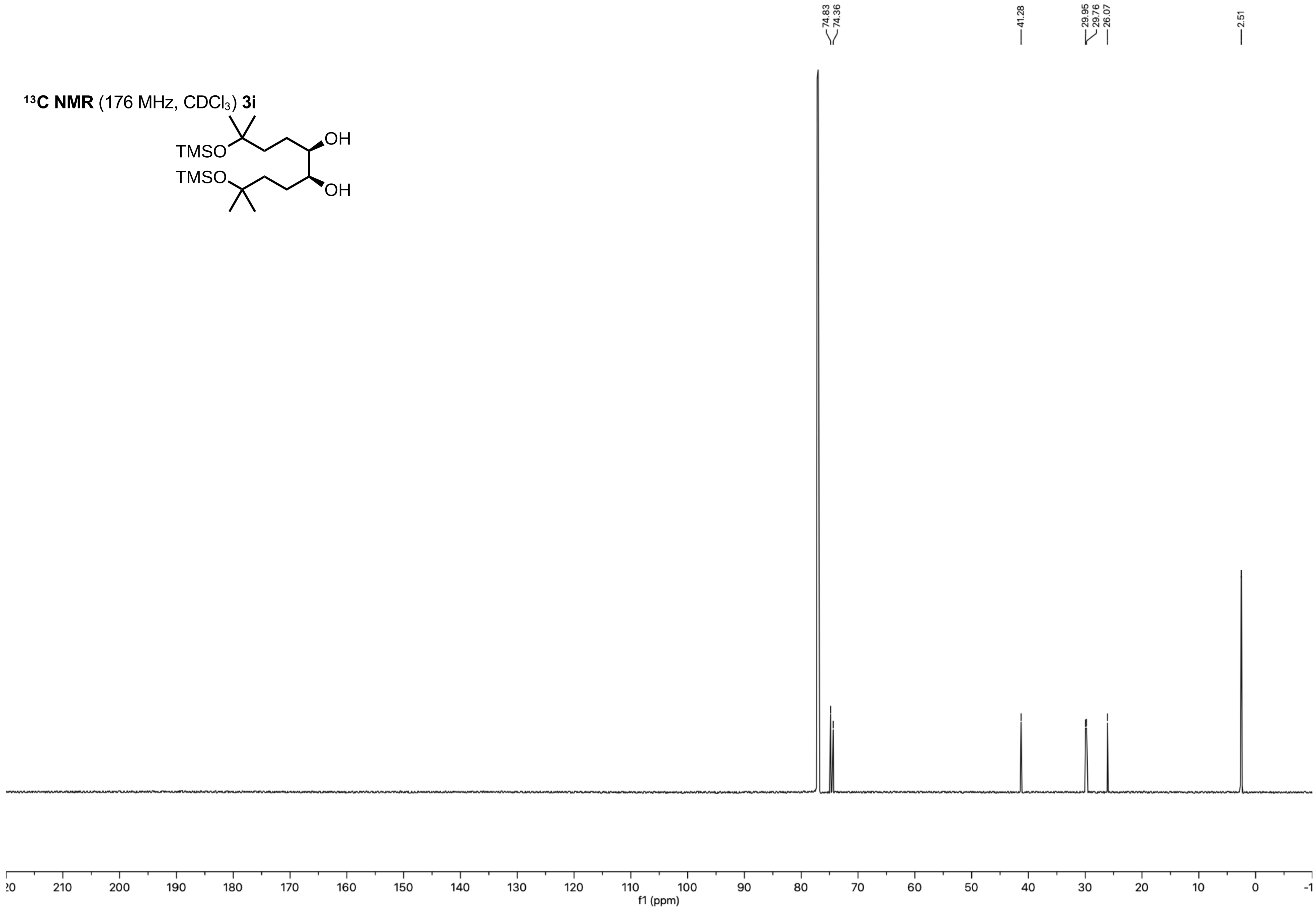

^{1}**H NMR** (700 MHz, $CDCl_3$) **3k**

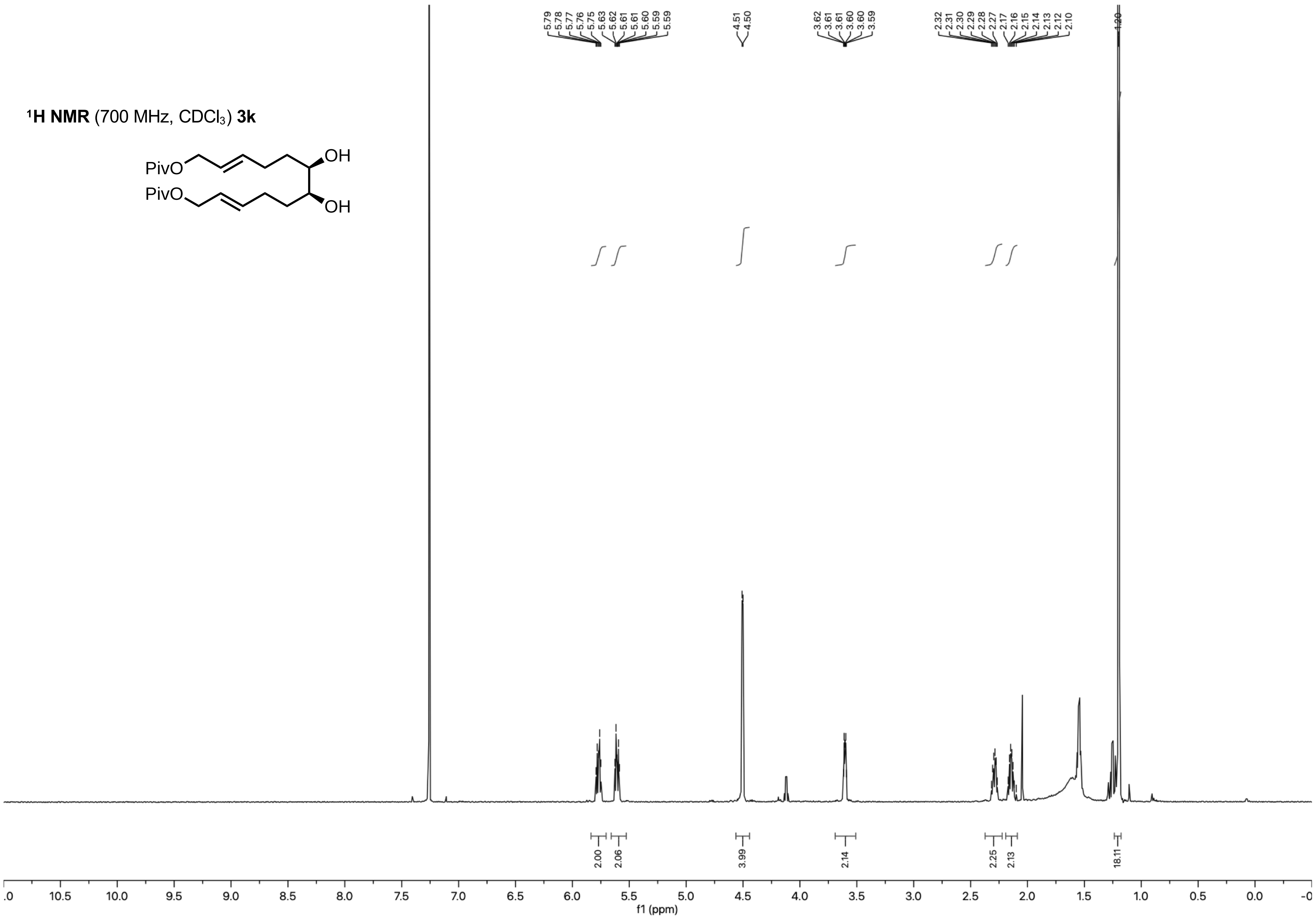

¹³C NMR (176 MHz, $CDCl_3$) **3k**

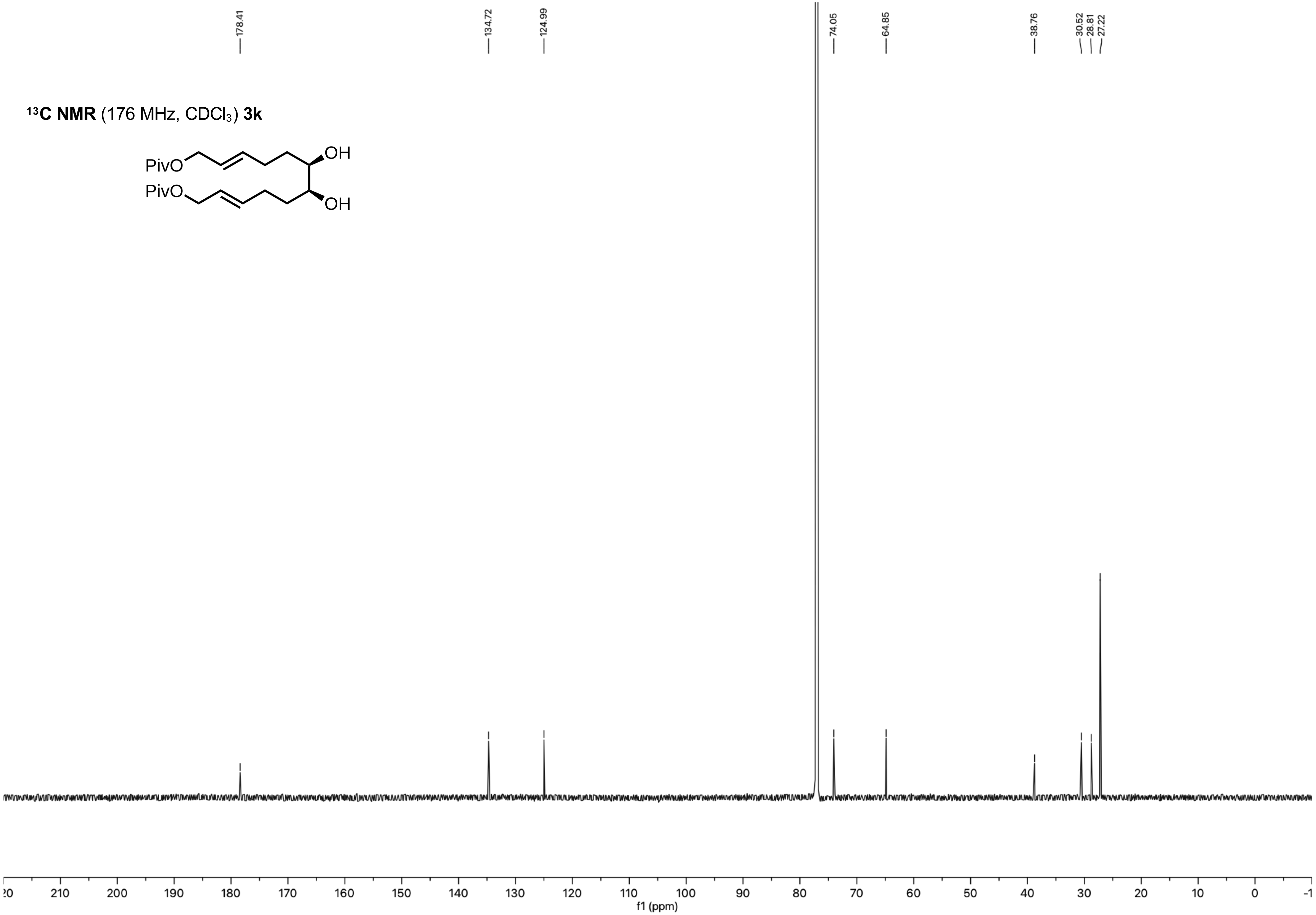

Spectroscopic data for enantioenriched hydroxyketones

¹H NMR (500 MHz, $CDCl_3$) **2a**

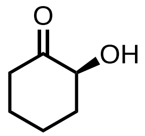

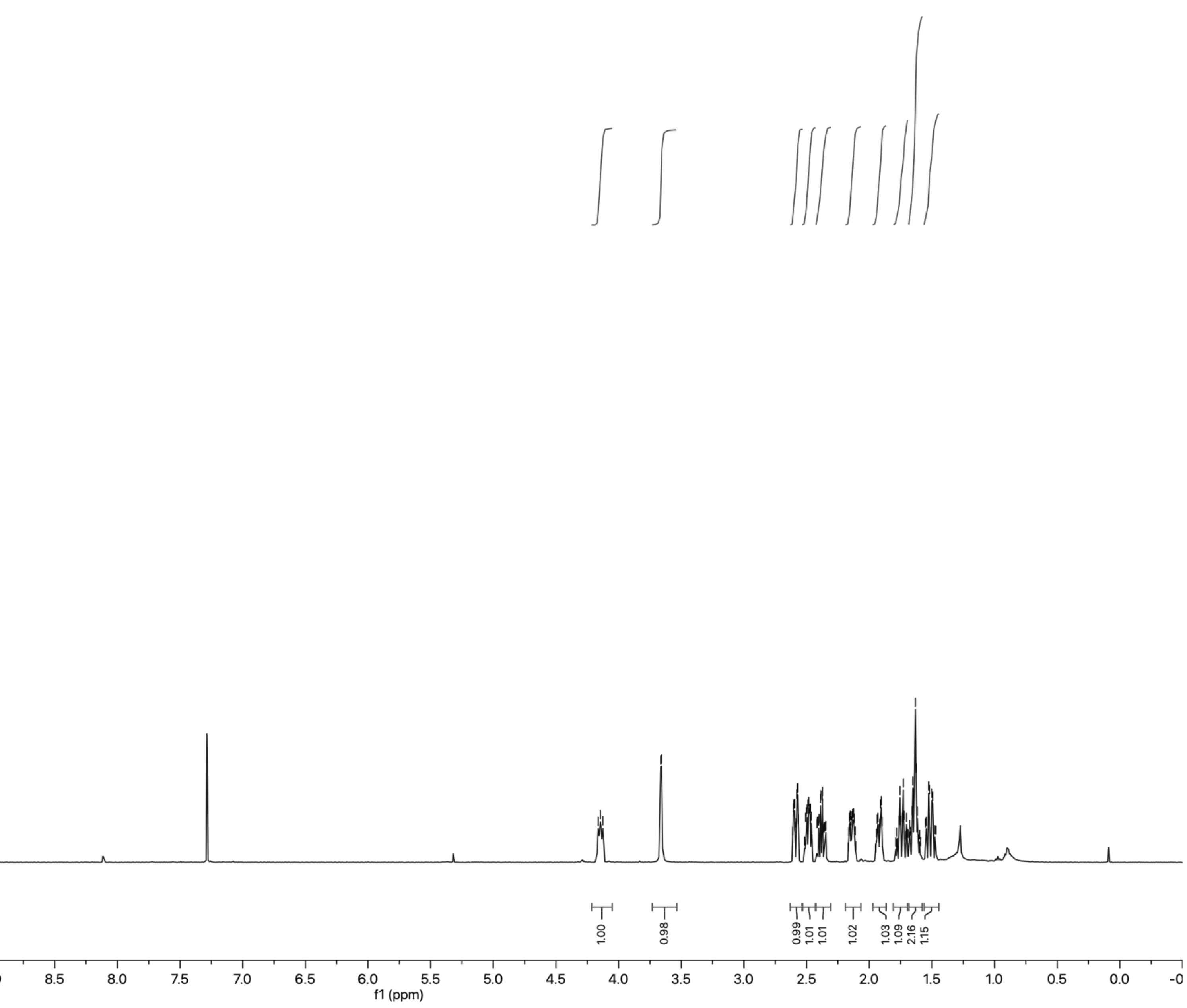

^{13}C NMR (126 MHz, $CDCl_3$) **2a**

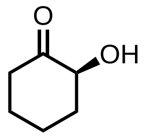

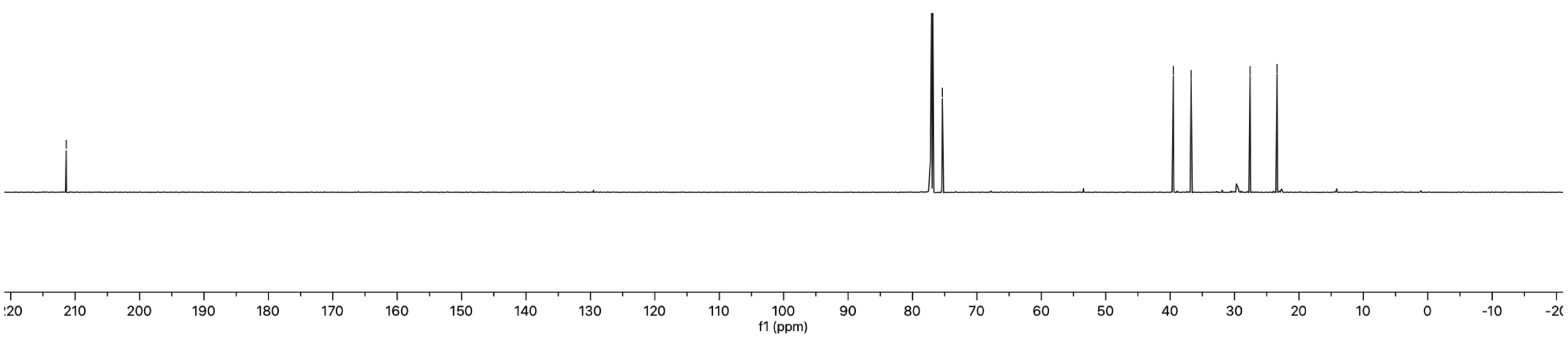

^{1}H NMR (700 MHz, $CDCl_3$) **2b**

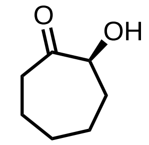

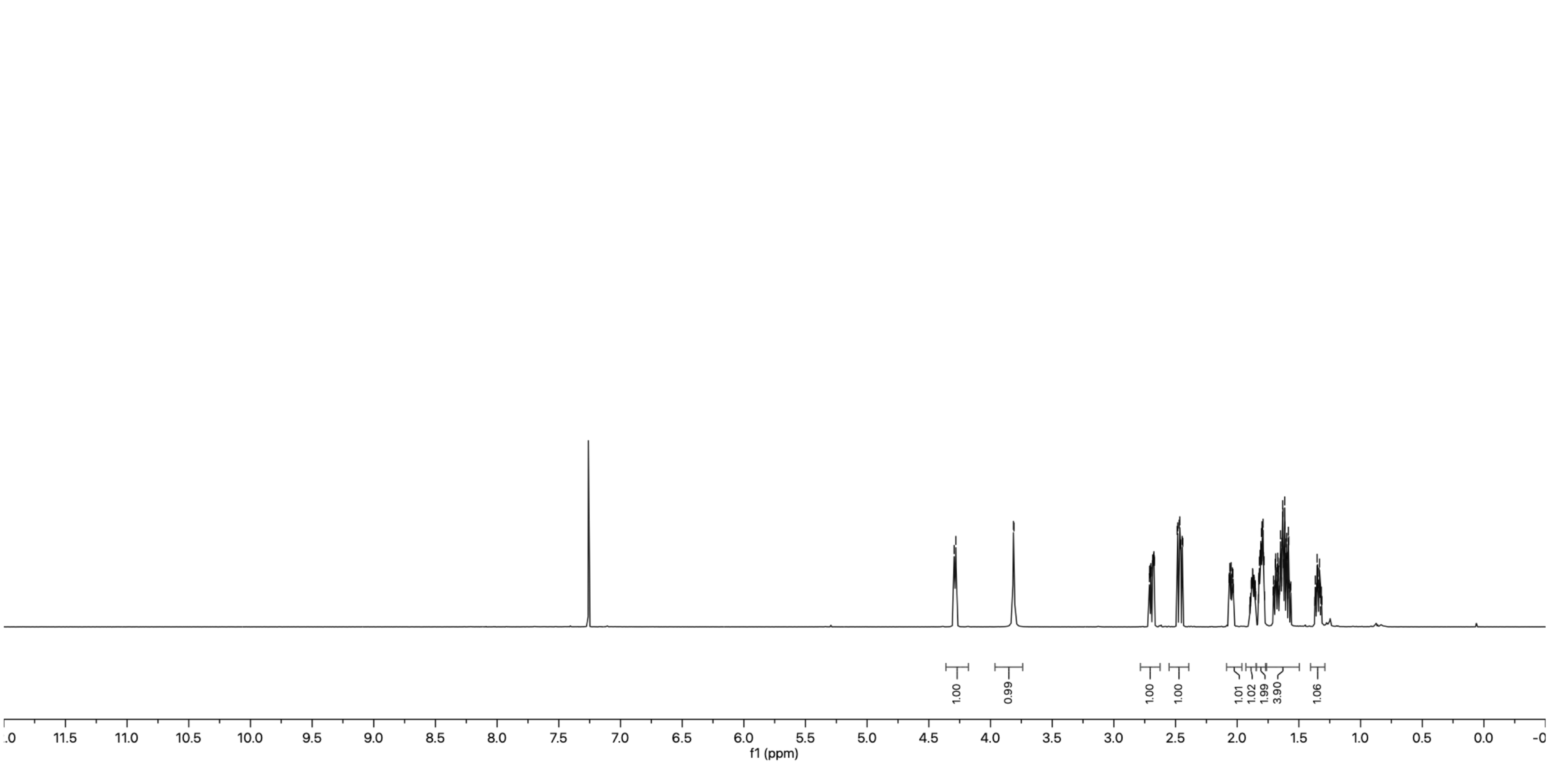

^{13}C NMR (176 MHz, $CDCl_3$) **2b**

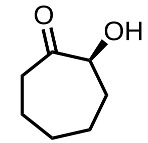

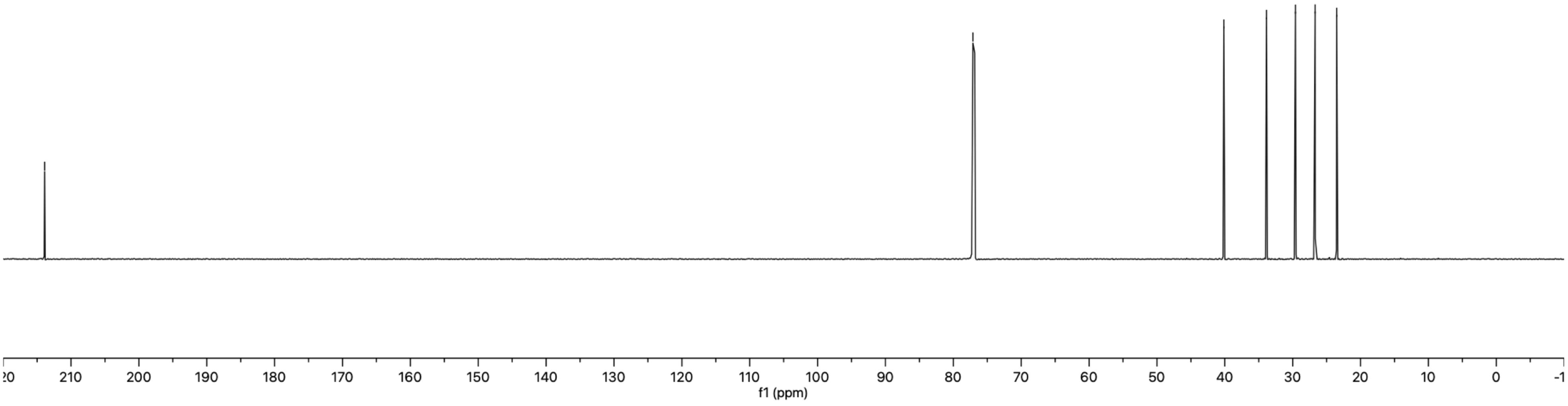

^{1}H NMR (400 MHz, $CDCl_3$) **2c**

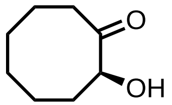

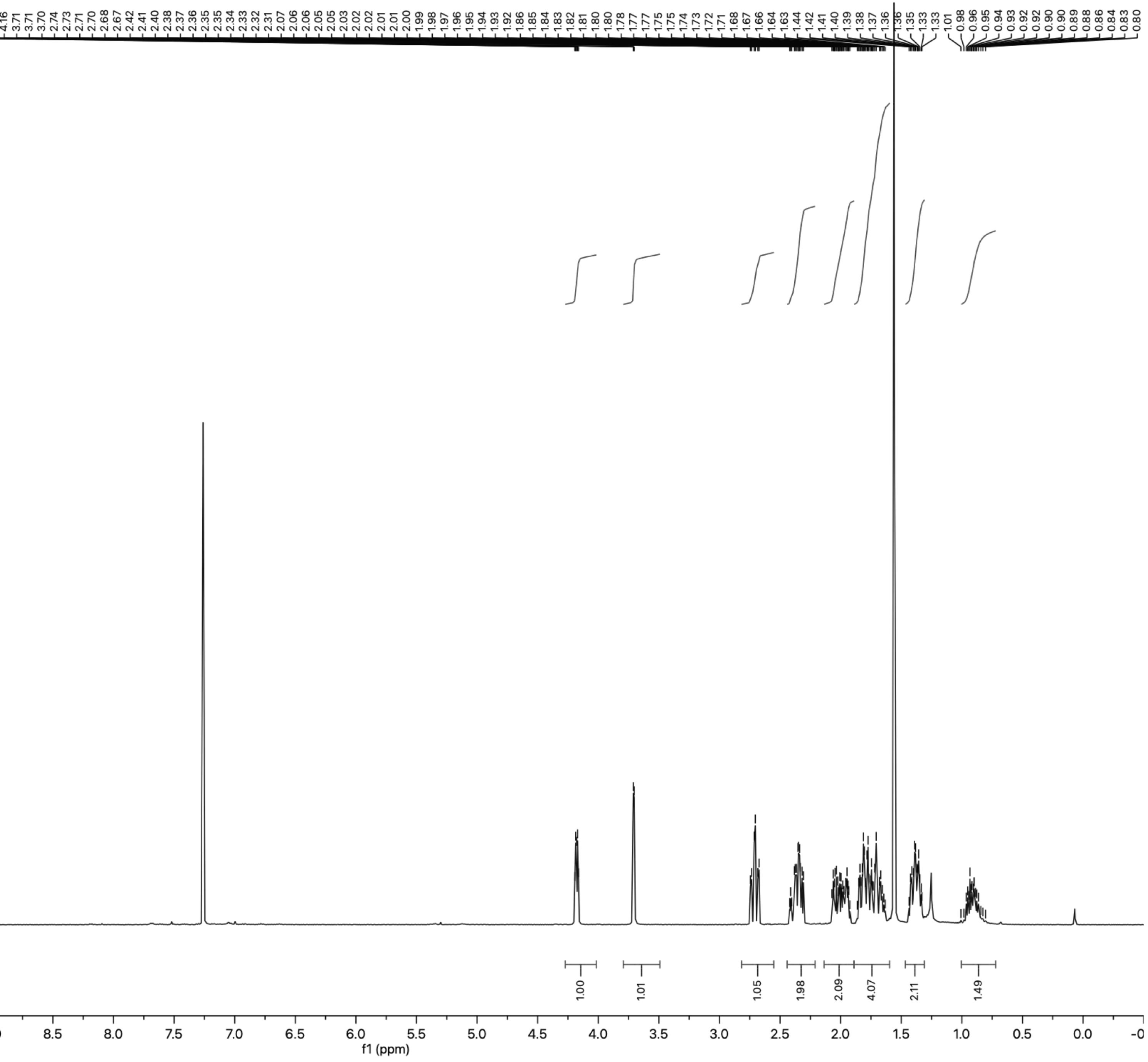

^{13}C NMR (101 MHz, $CDCl_3$) **2c**

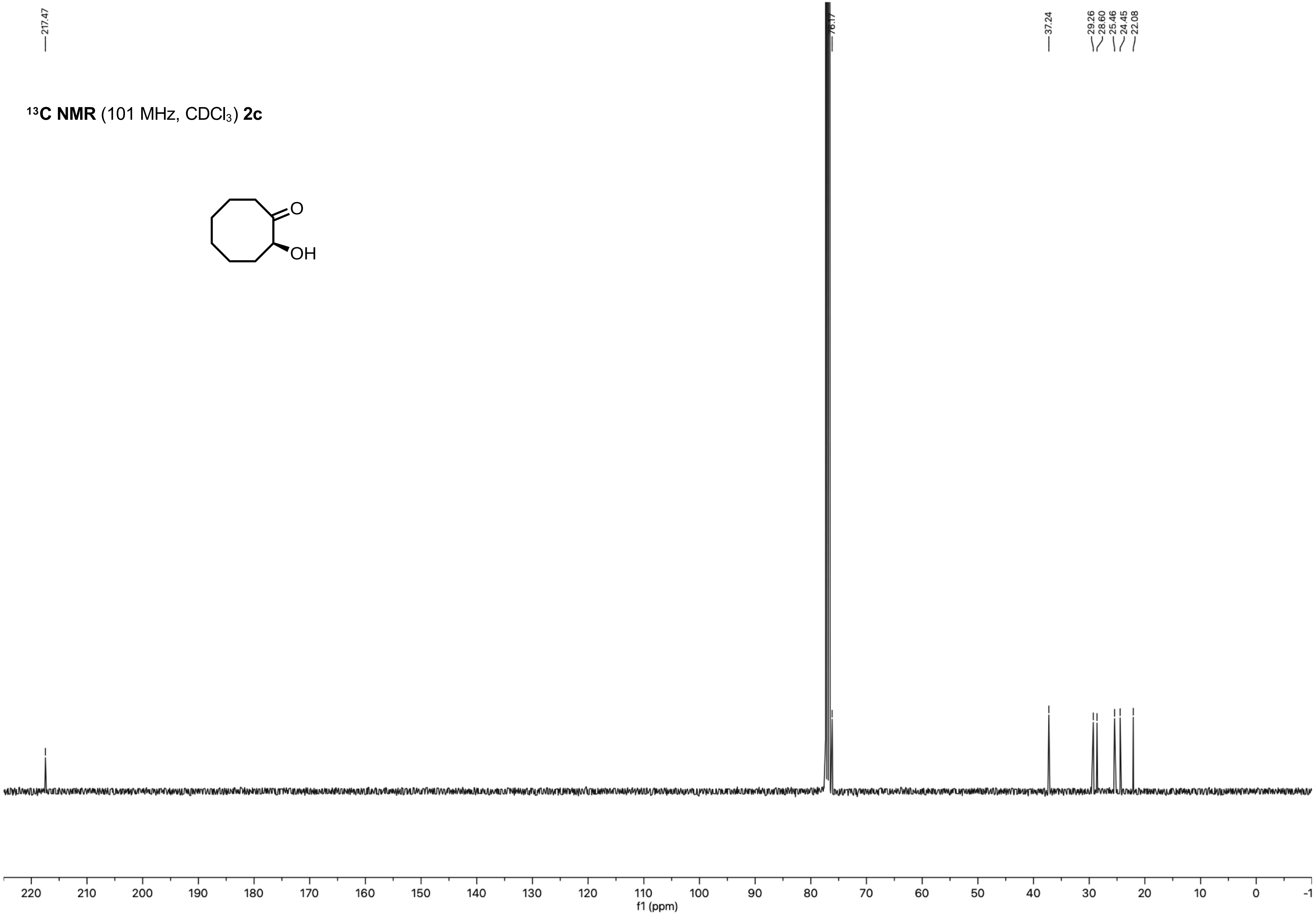

¹H NMR (700 MHz, $CDCl_3$) **Bz-2d**

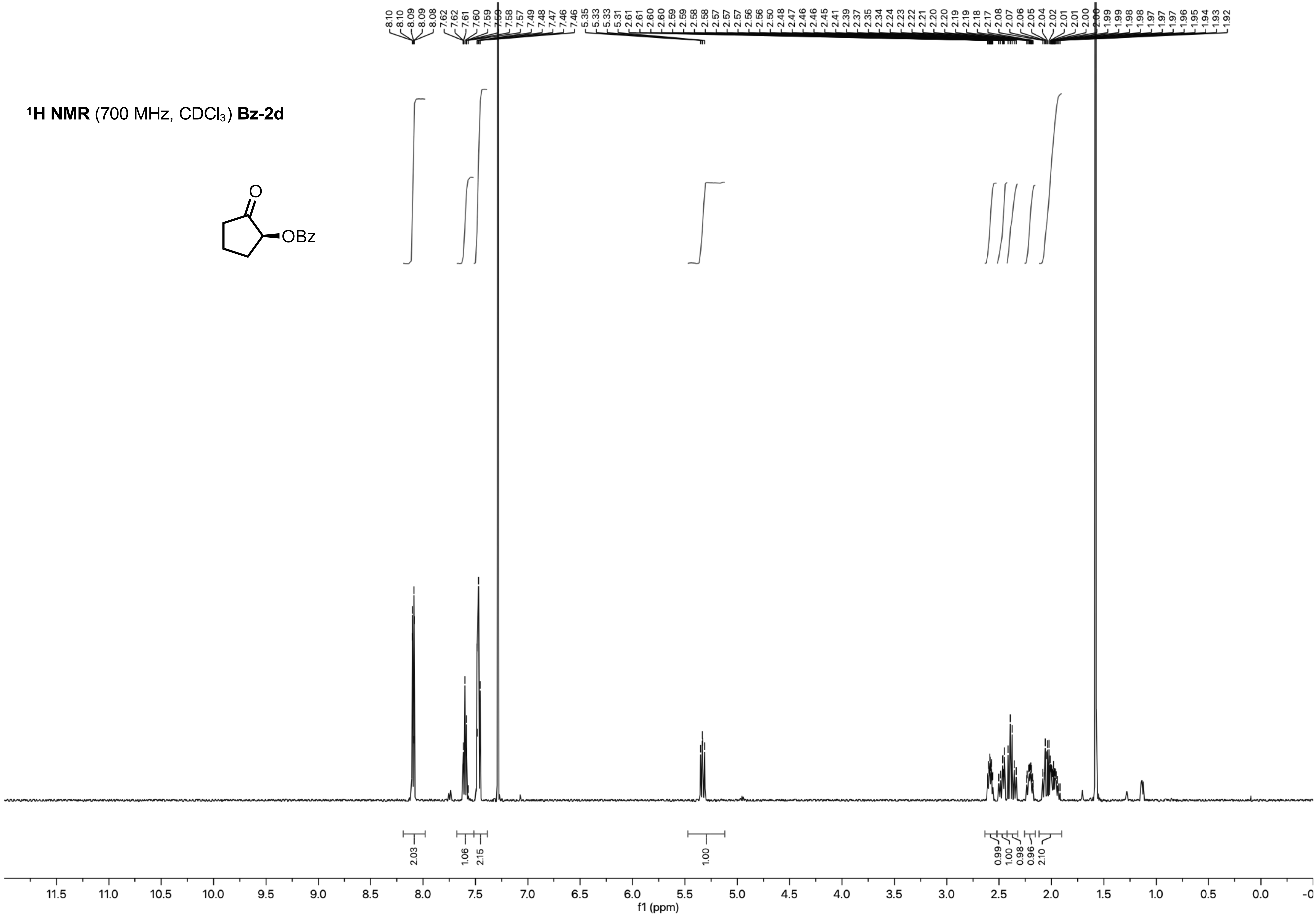

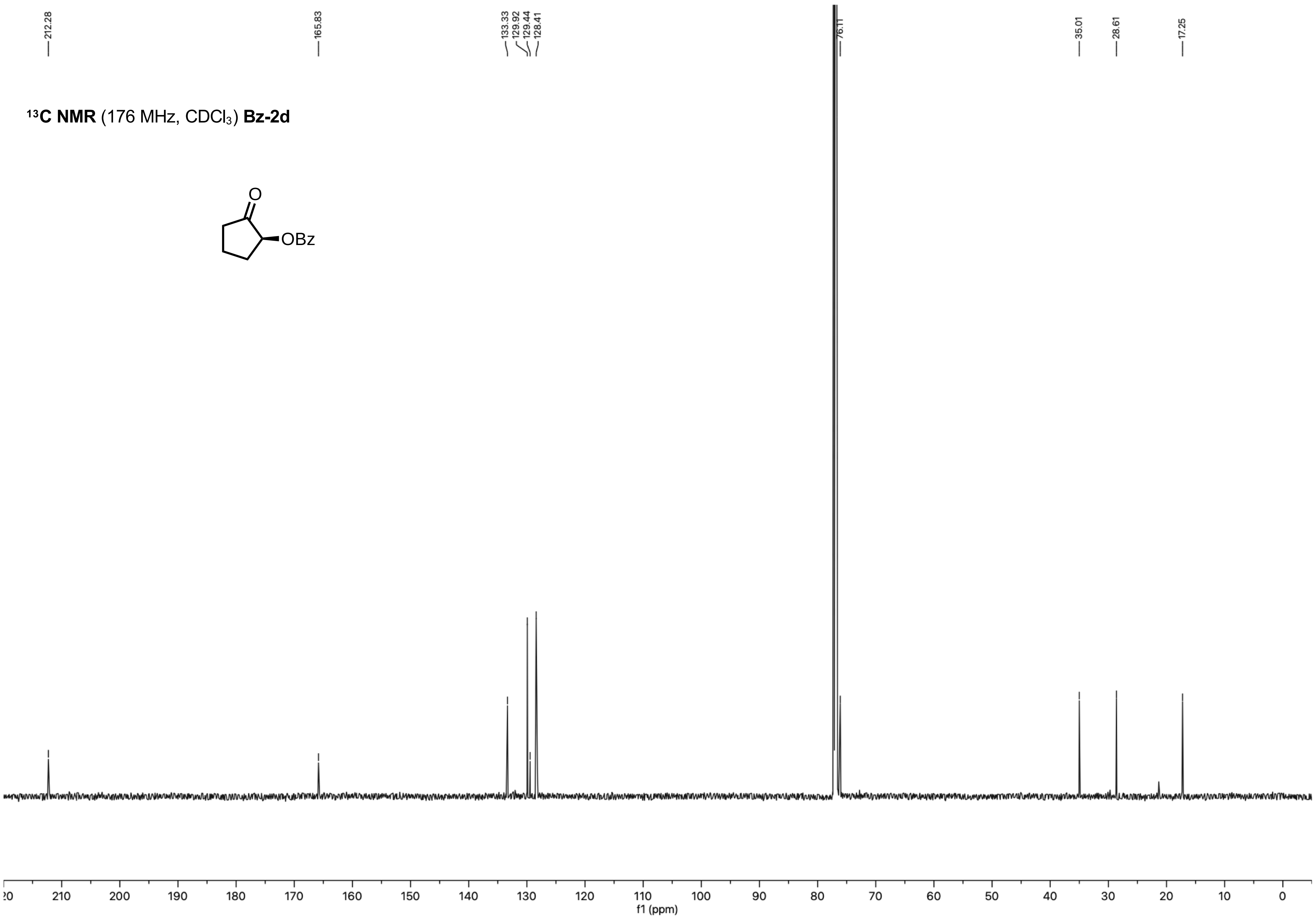

^{13}C NMR (176 MHz, CDCl$_3$) Bz-2d
OBz
O
212.28
165.83
133.33
129.92
129.44
128.41
76.11
35.01
28.61
17.25
f1 (ppm)

^{1}H NMR (400 MHz, $CDCl_3$) **2e**

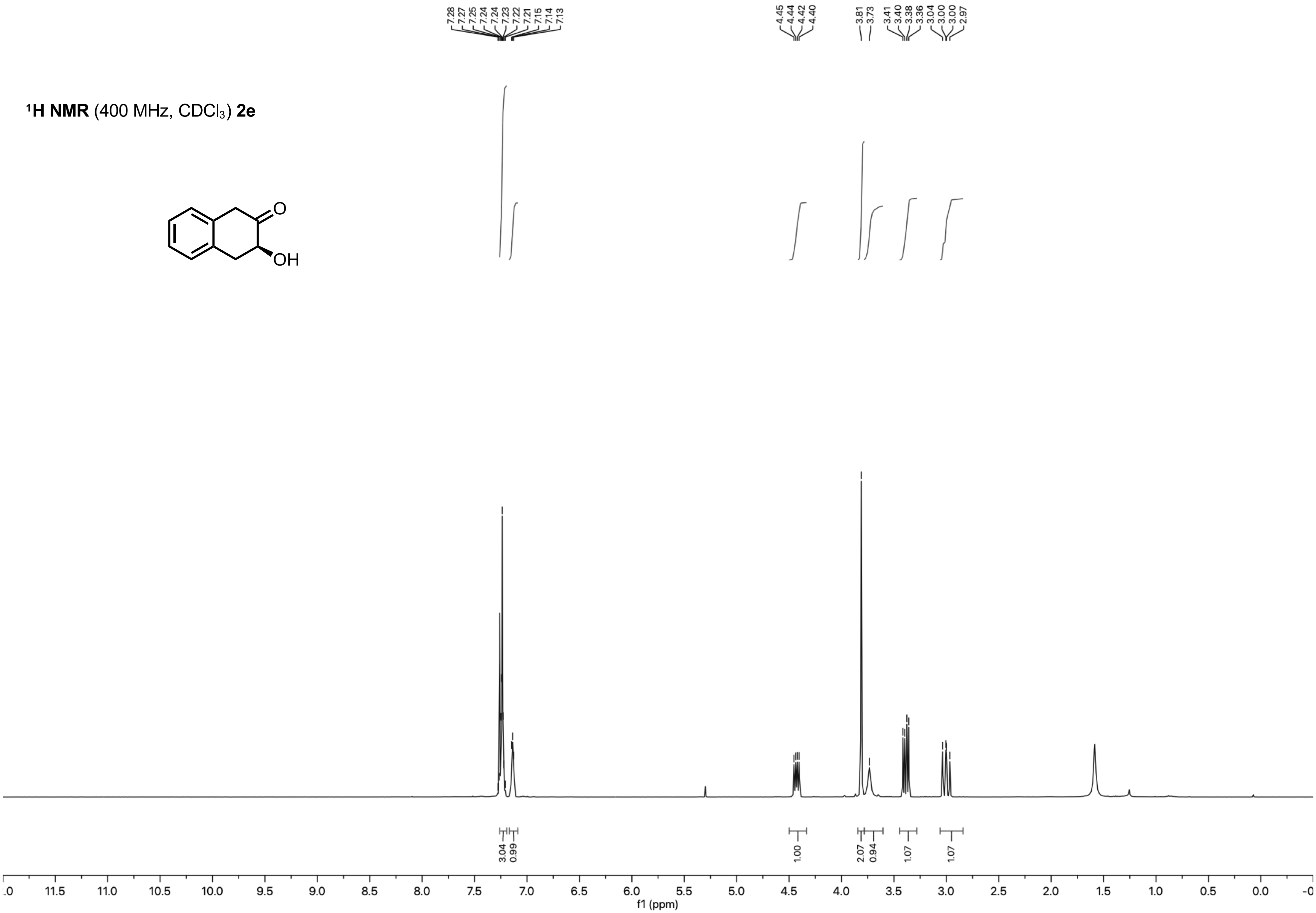

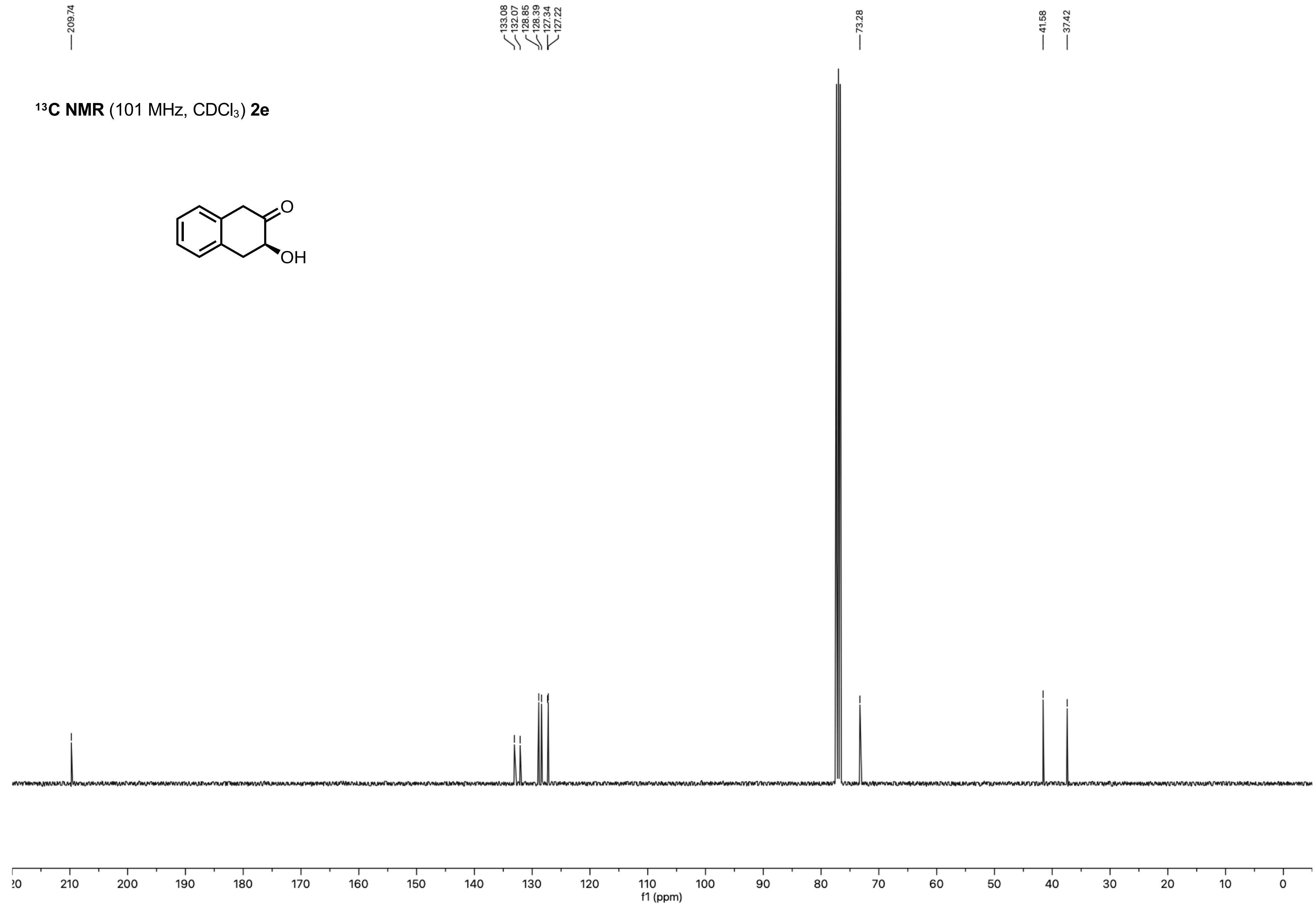

209.74
133.08
132.07
128.85
128.39
127.34
127.22
73.28
41.58
37.42
^{13}C NMR (101 MHz, $CDCl_3$) 2e
O
OH
20
210
200
190
180
170
160
150
140
130
120
110
100
90
80
70
60
50
40
30
20
10
0
f1 (ppm)

^{1}H NMR (700 MHz, $CDCl_3$) **2f**

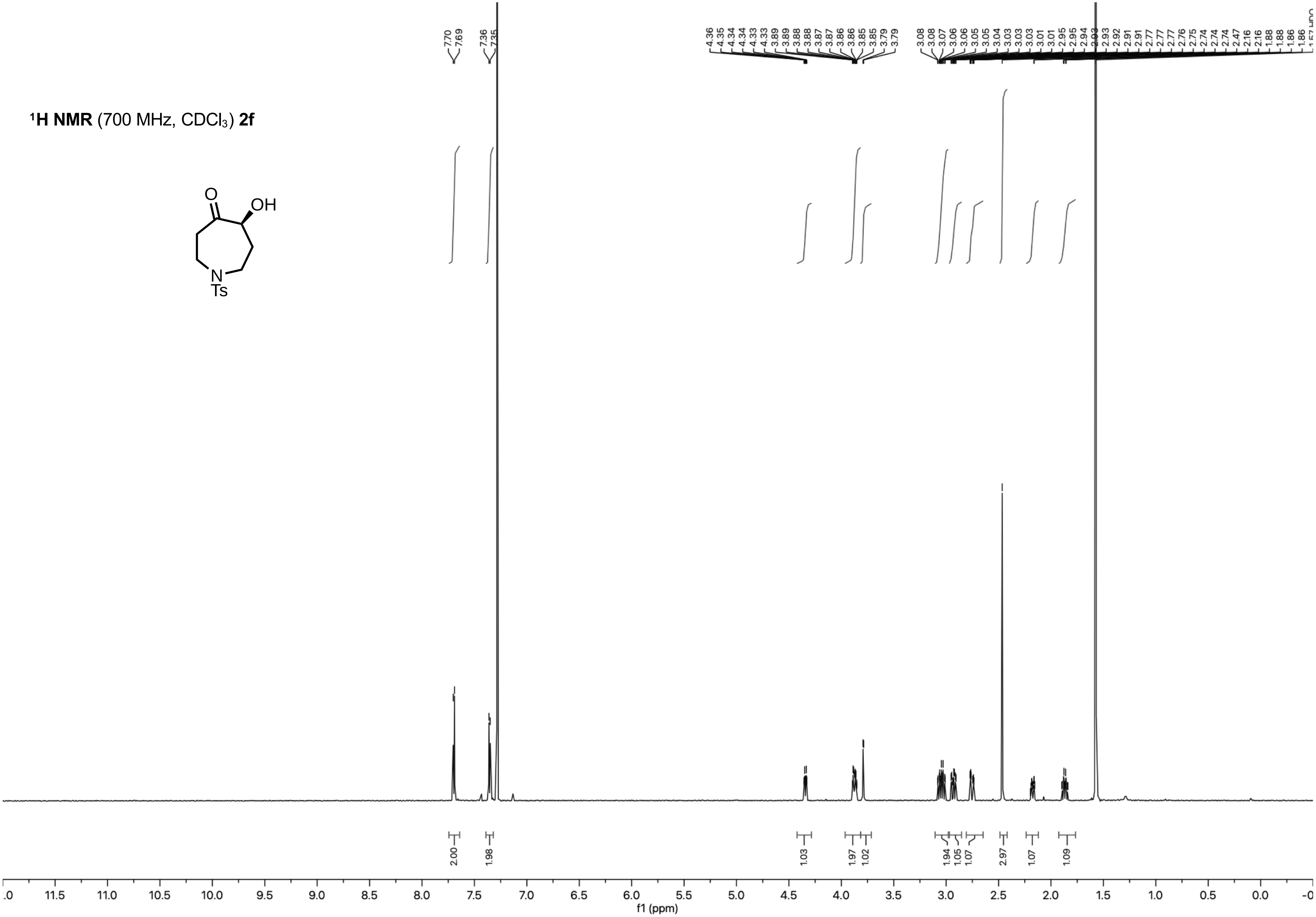

^{13}C NMR (176 MHz, $CDCl_3$) **2f**

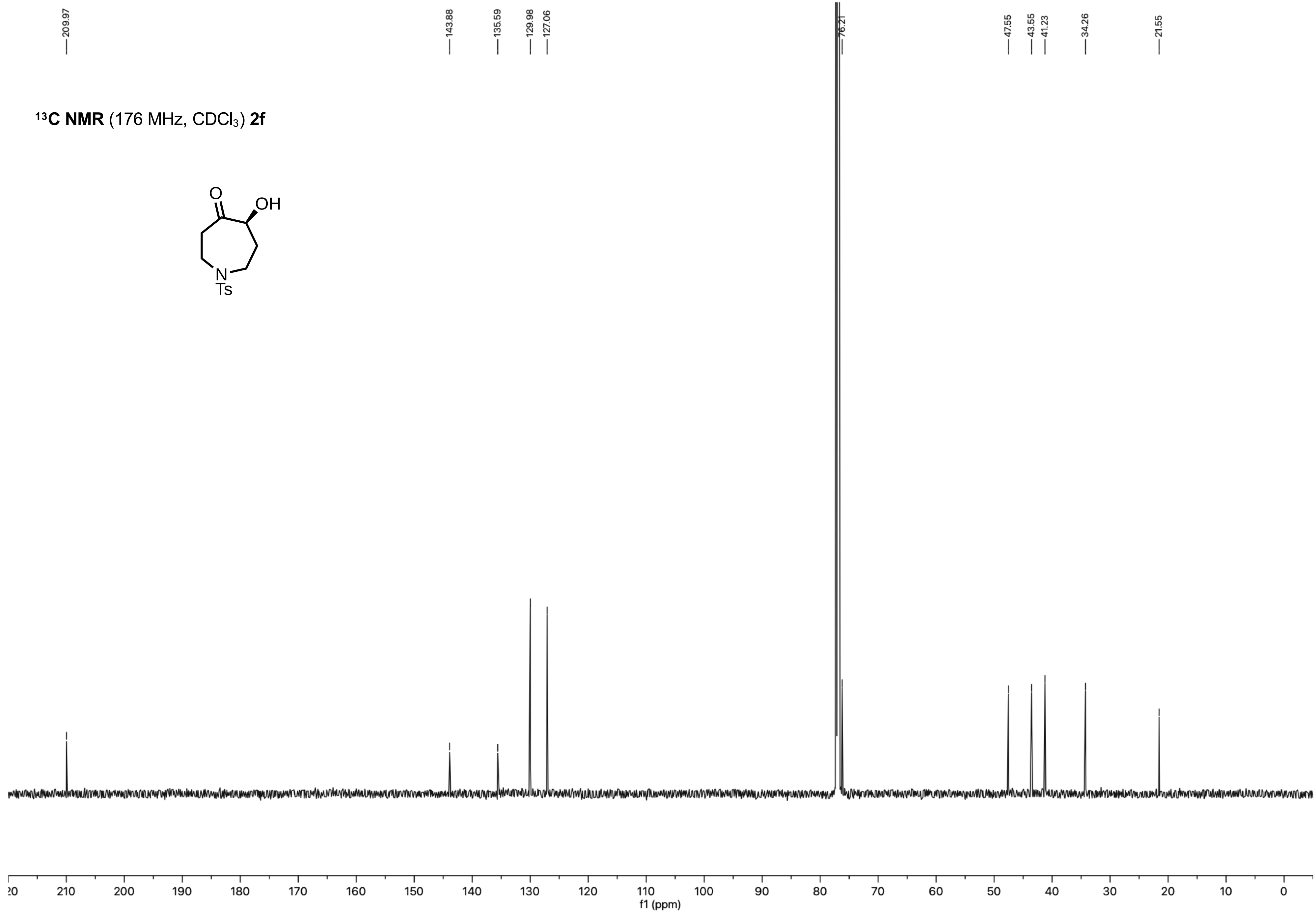

¹H NMR (700 MHz, $CDCl_3$) **2g**

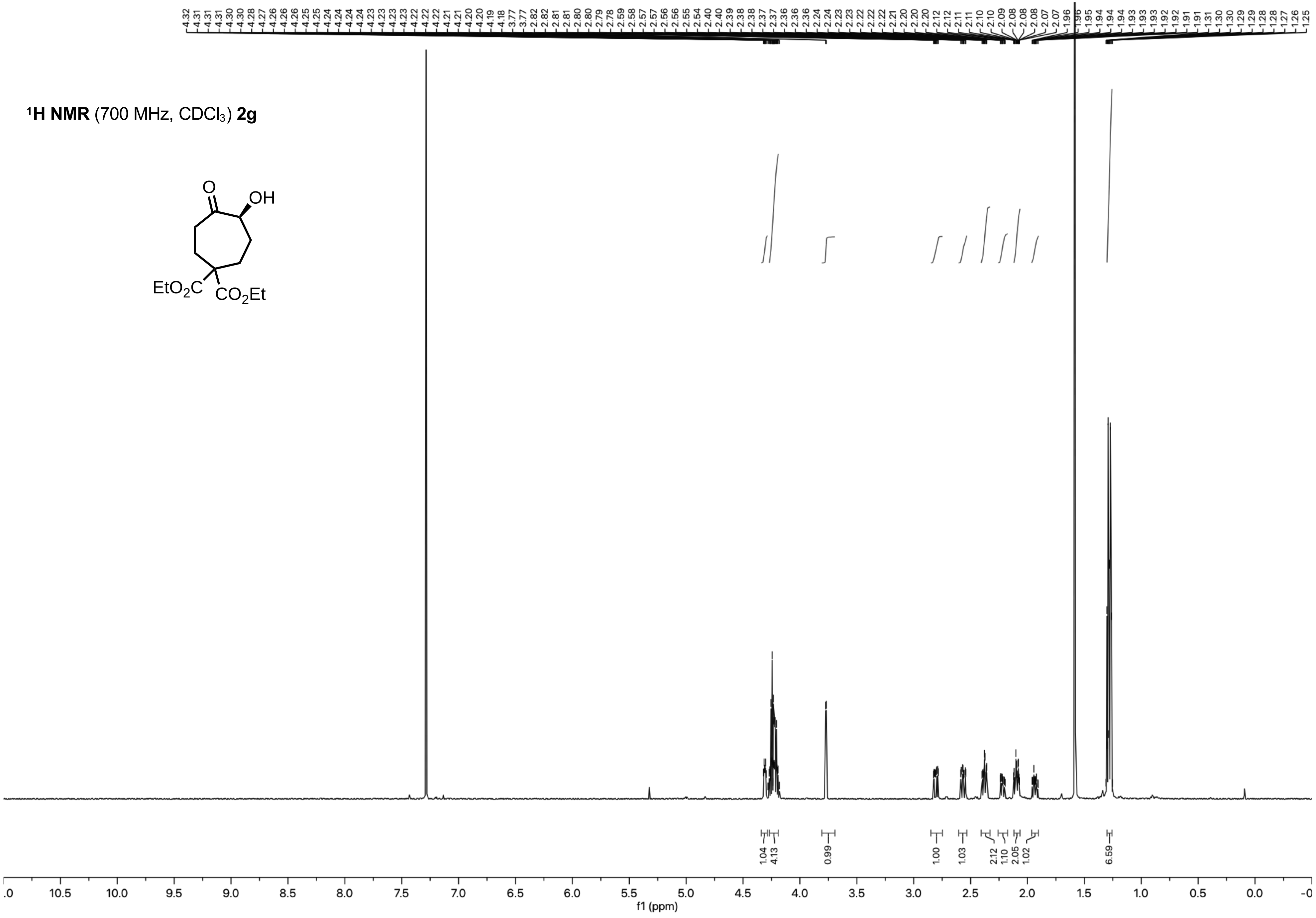

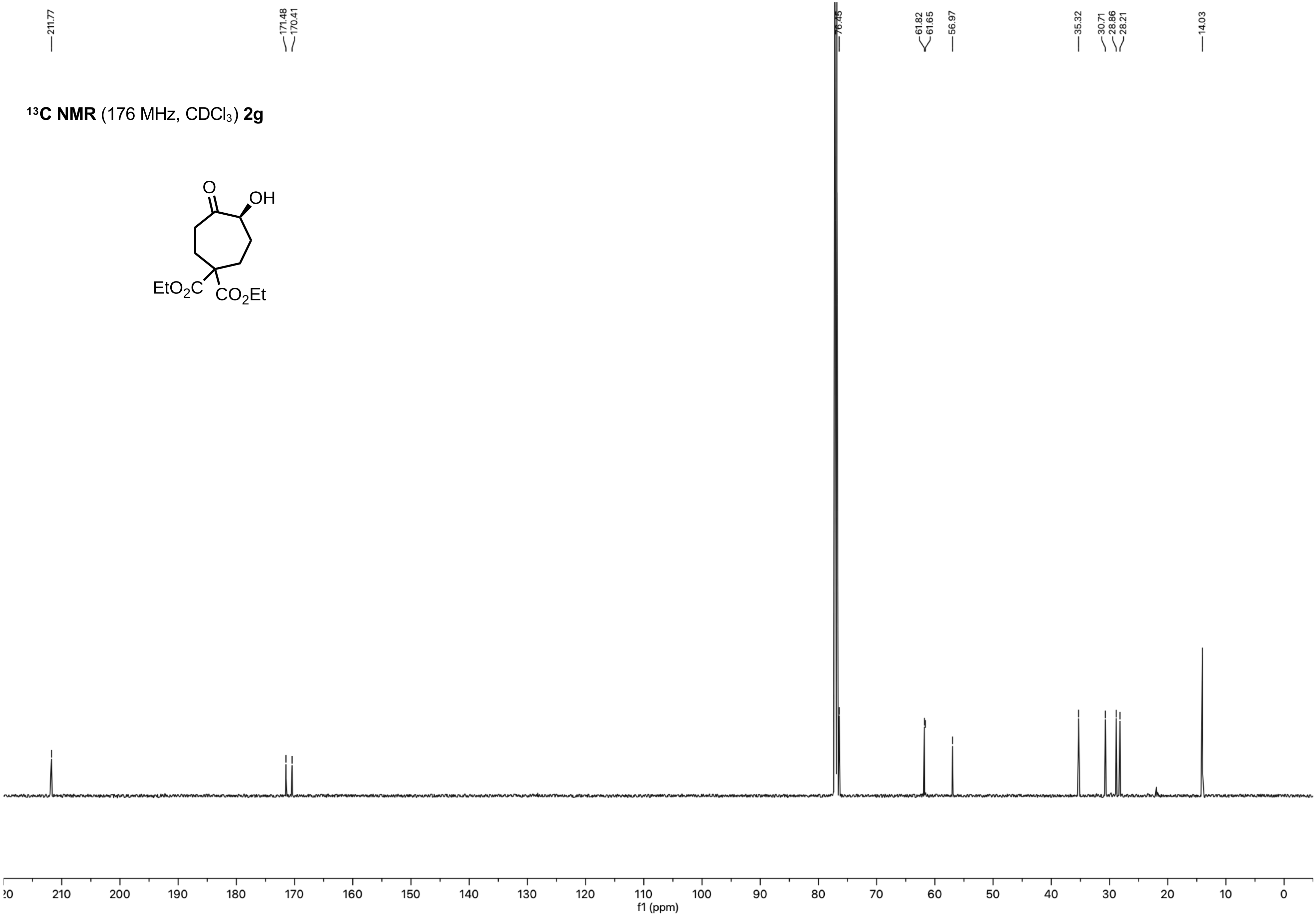

211.77
171.48
170.41
76.45
61.82
61.65
56.97
35.32
30.71
28.86
28.21
14.03
¹³C NMR (176 MHz, CDCl₃) 2g
O
OH
EtO₂C
CO₂Et
20
210
200
190
180
170
160
150
140
130
120
110
100
90
80
70
60
50
40
30
20
10
0
f1 (ppm)

¹H NMR (700 MHz, $CDCl_3$) **2h**

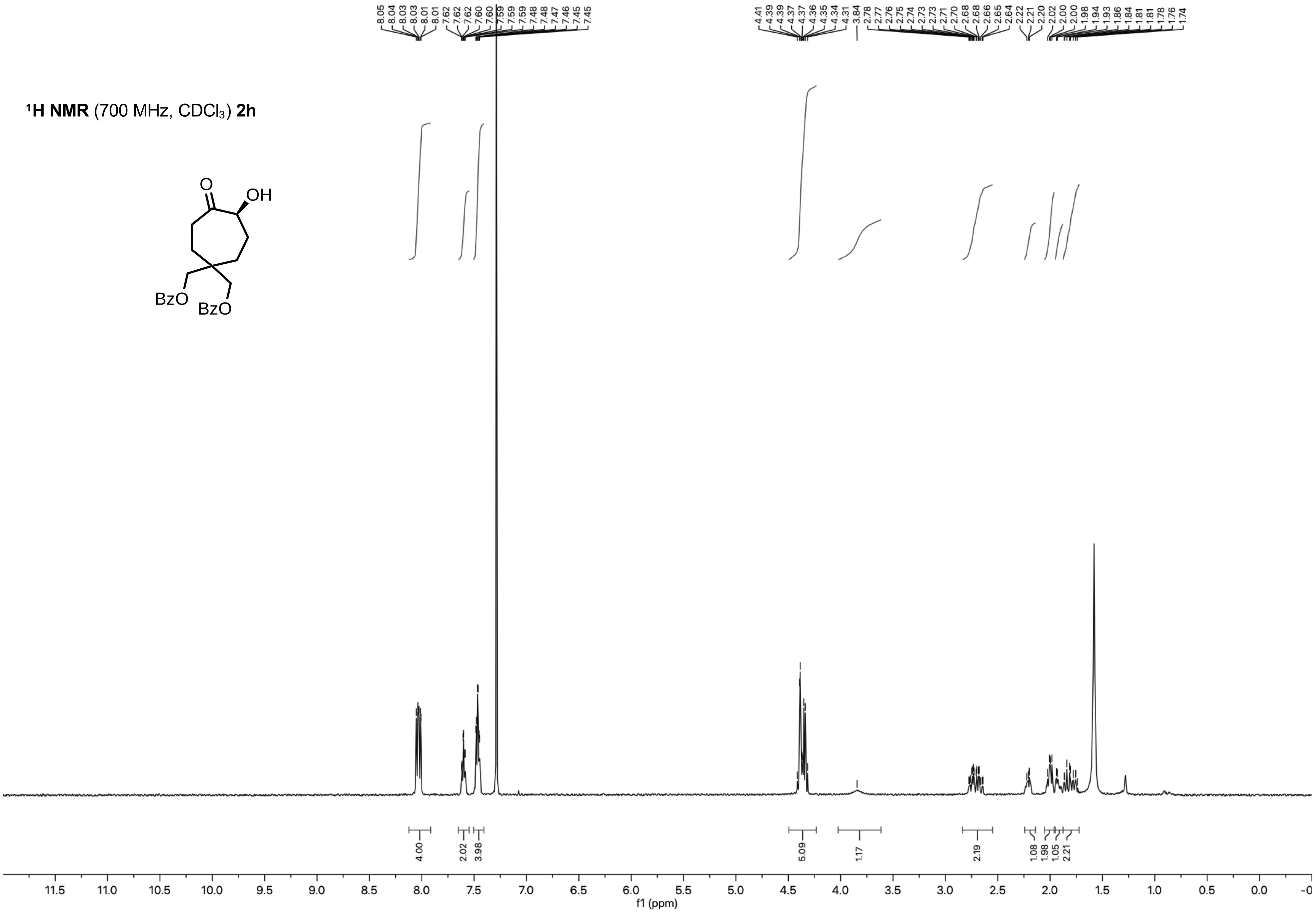

^{13}C NMR (176 MHz, $CDCl_3$) **2h**

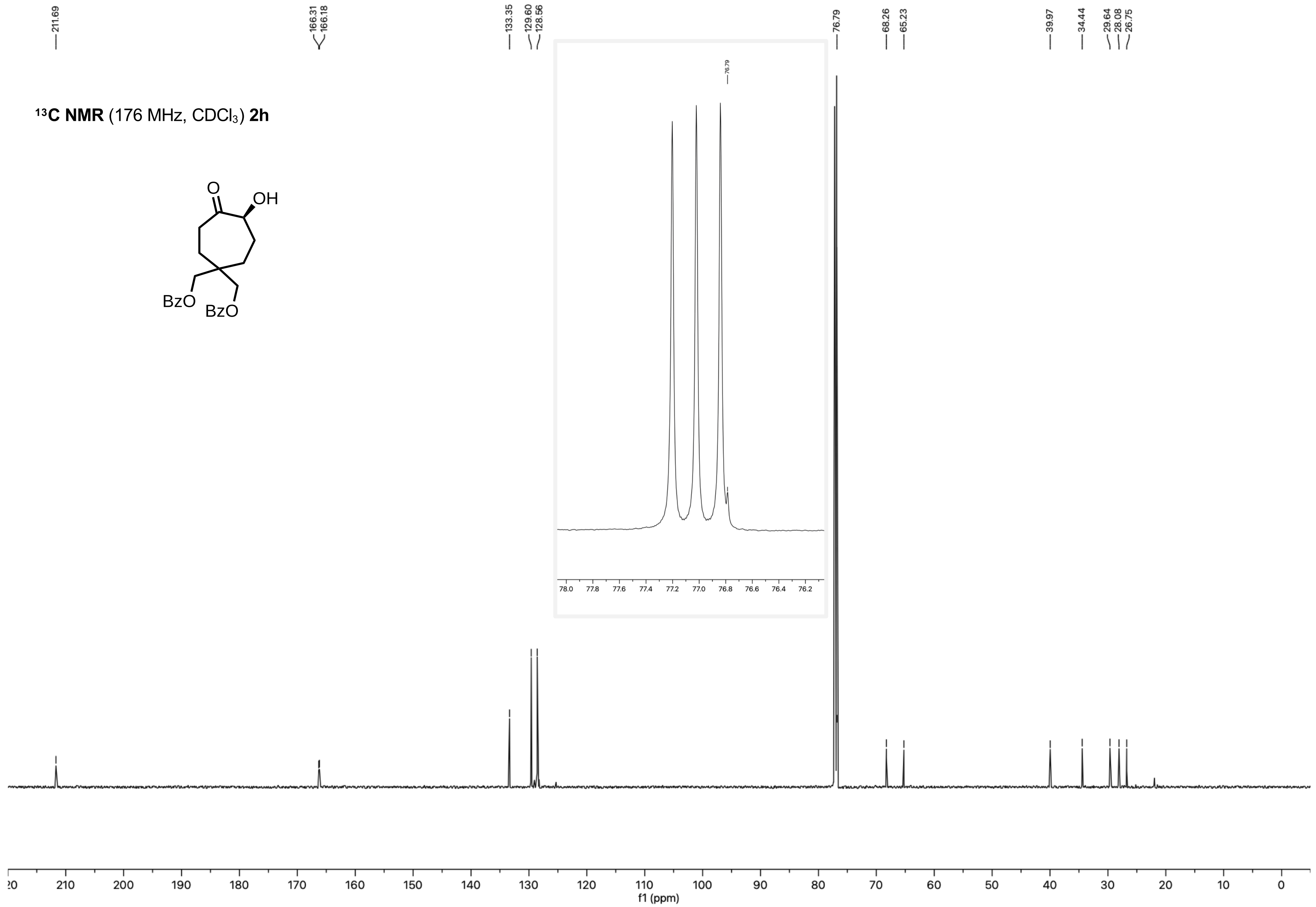

¹H NMR (700 MHz, $CDCl_3$) **2i**

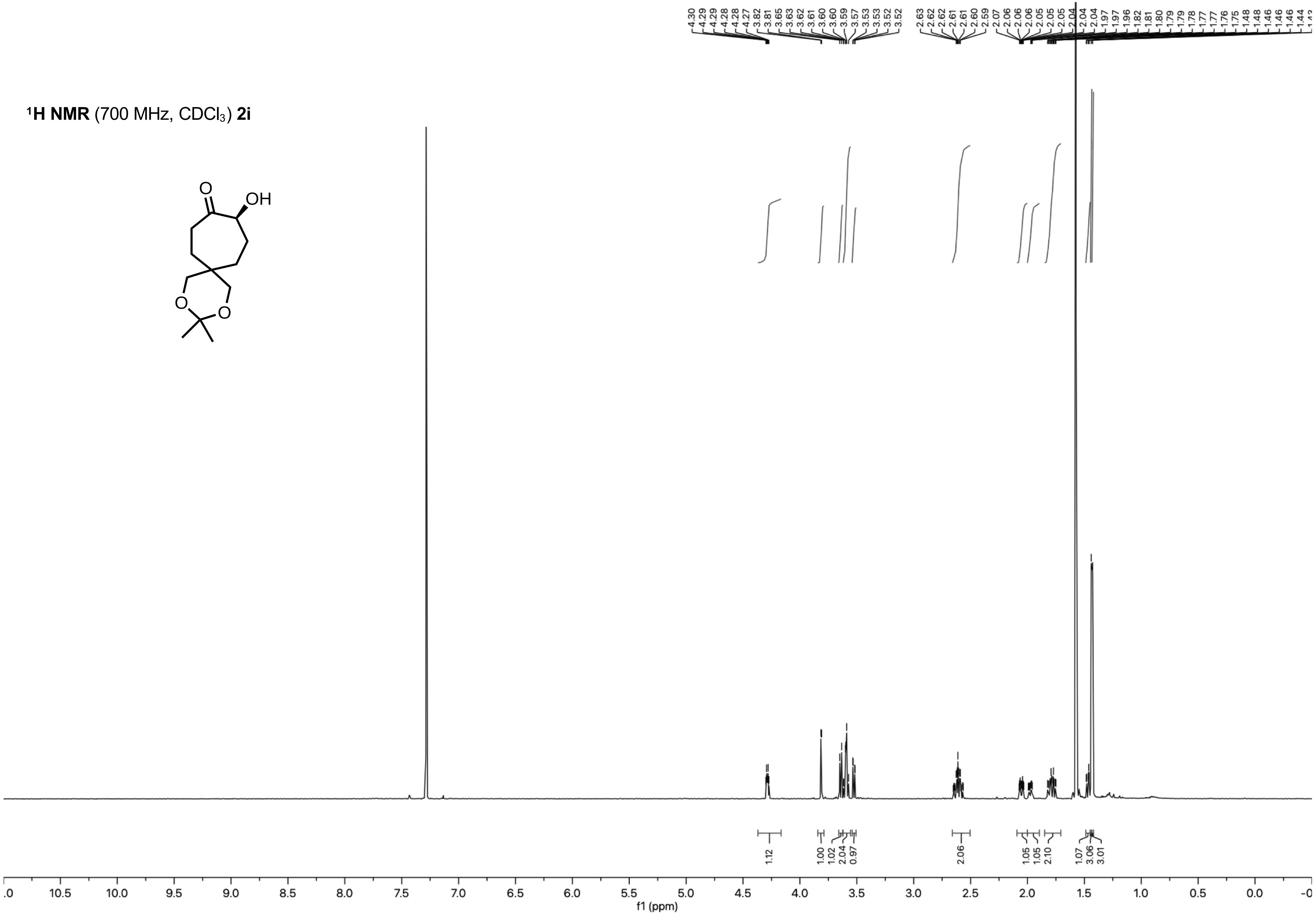

^{13}C NMR (176 MHz, $CDCl_3$) 2i

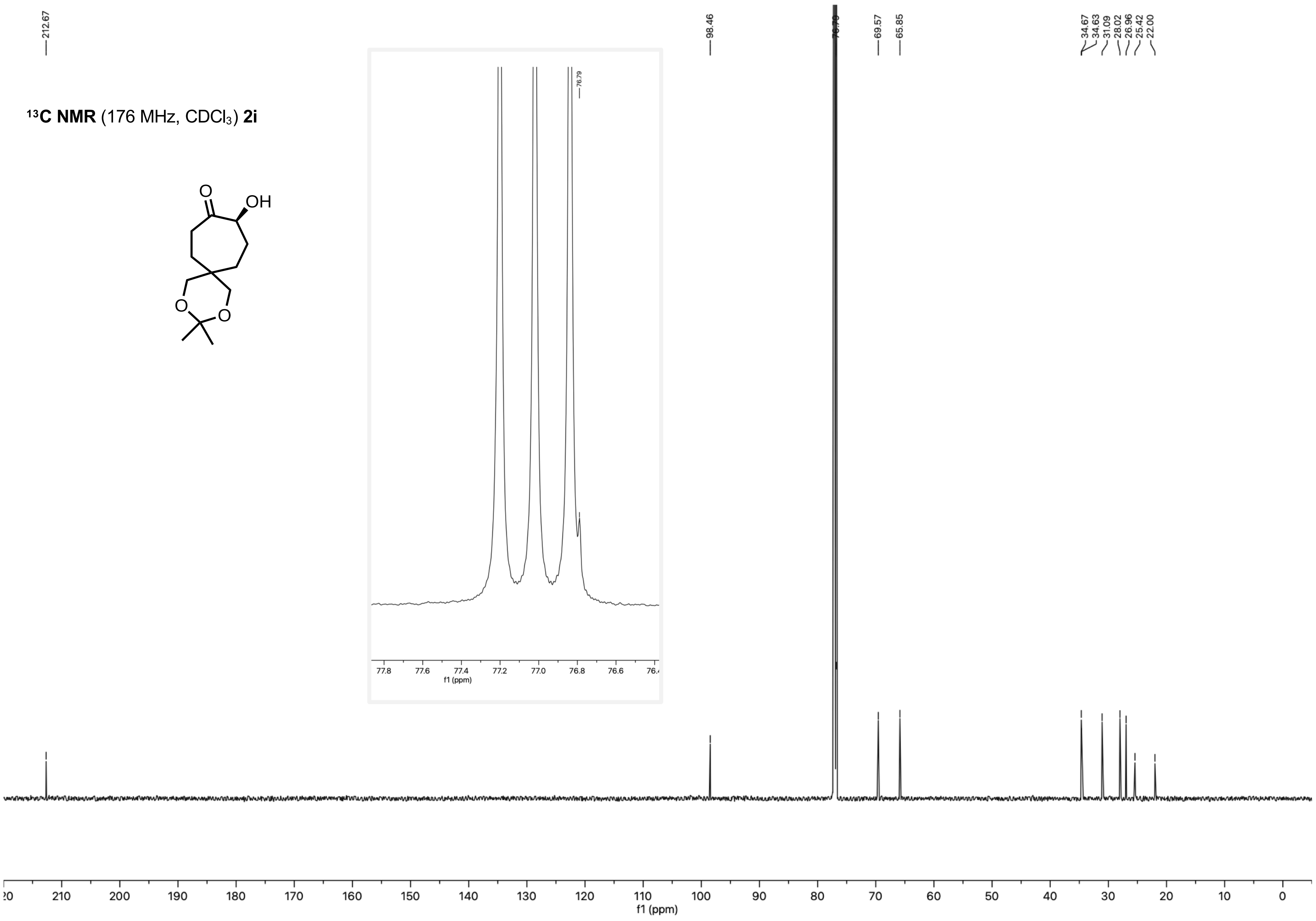

¹H NMR (700 MHz, CDCl₃) **2j**

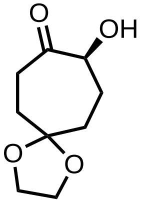

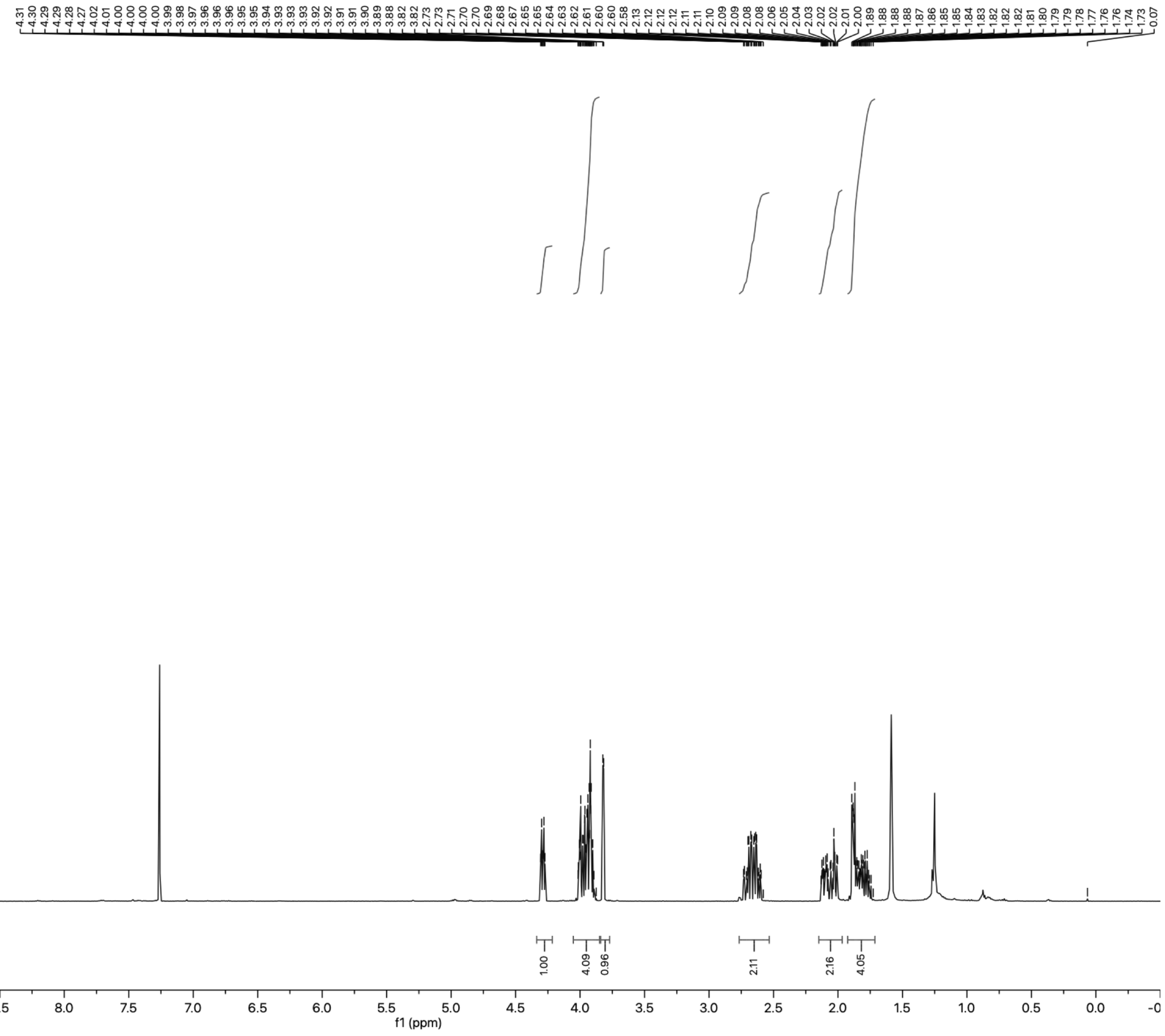

^{13}C NMR (176 MHz, $CDCl_3$) **2j**

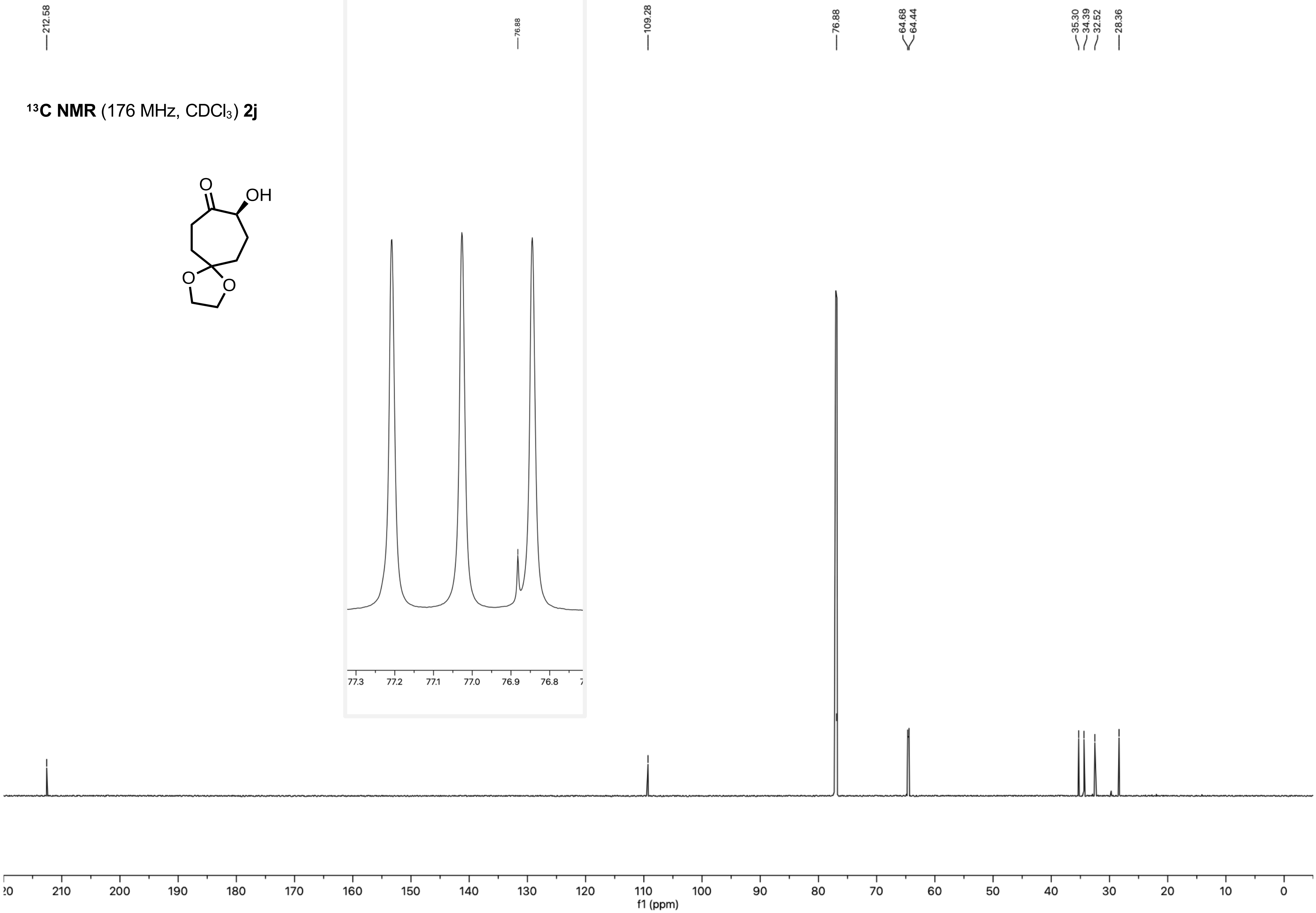

^{1}H NMR (700 MHz, $CDCl_3$) **2k**

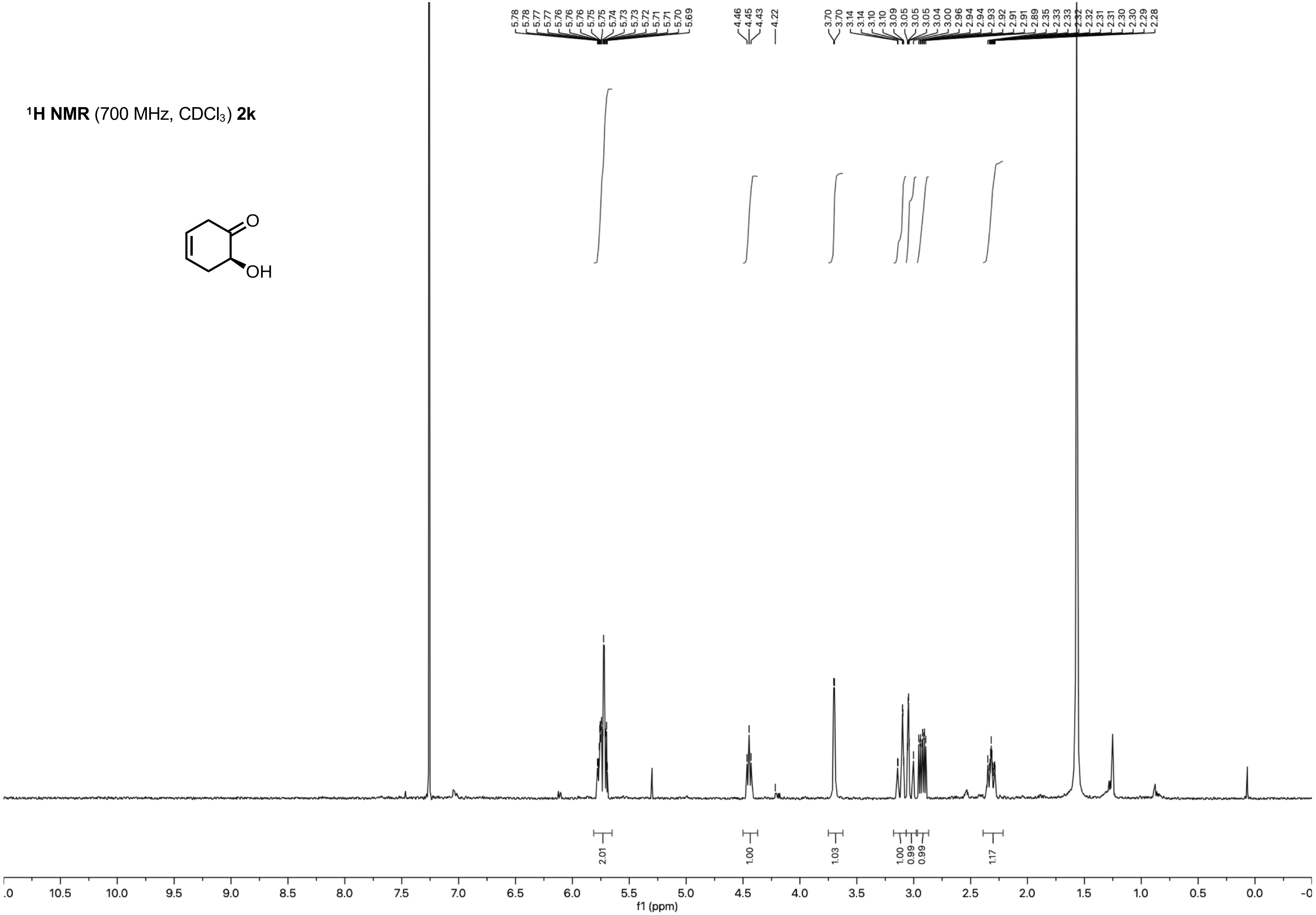

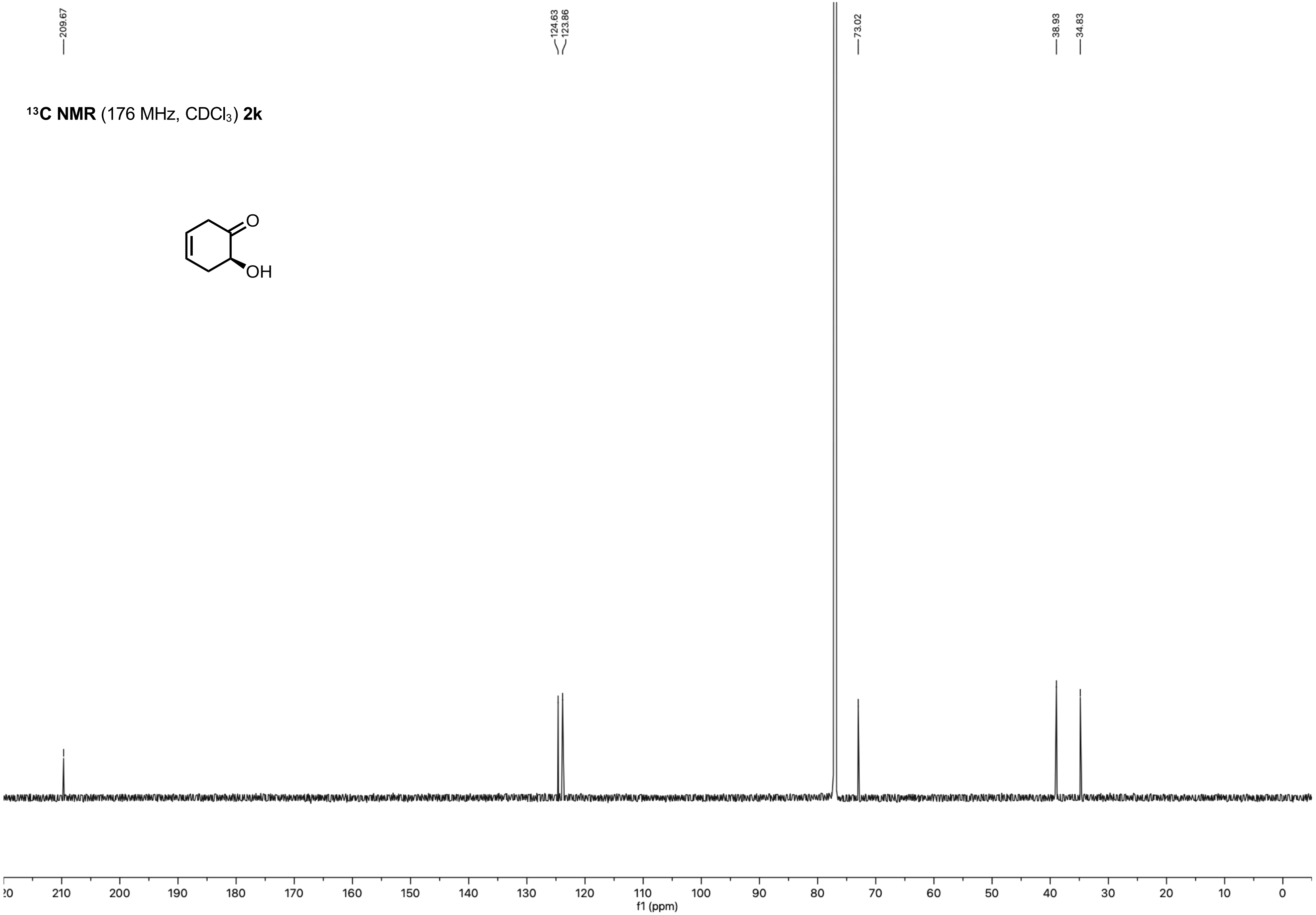
¹³C NMR (176 MHz, CDCl$_3$) 2k
O
OH
209.67
124.63
123.86
73.02
38.93
34.83
210
200
190
180
170
160
150
140
130
120
110
100
90
80
70
60
50
40
30
20
10
0
f1 (ppm)

^{1}H NMR (400 MHz, $CDCl_3$) **2l**

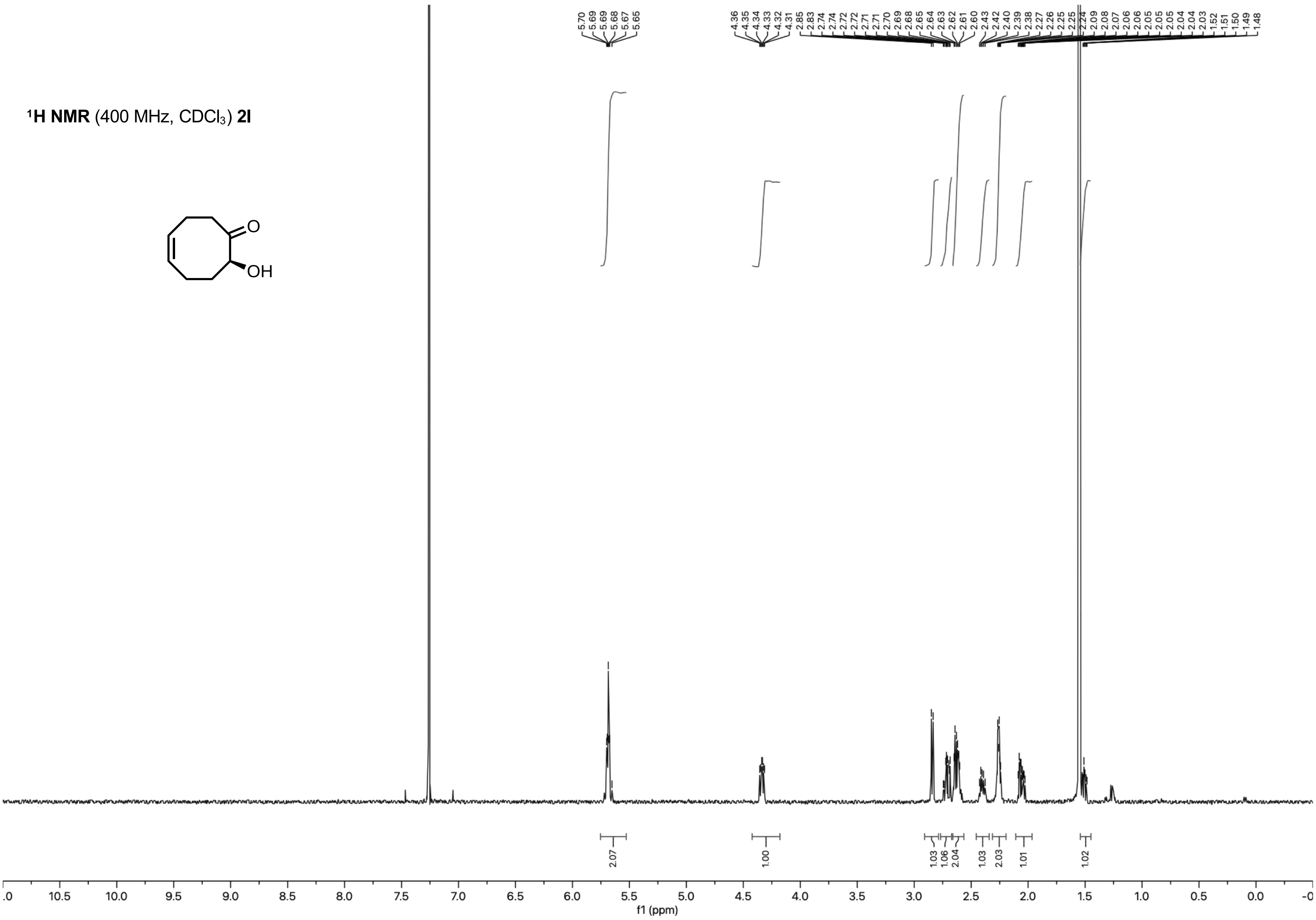

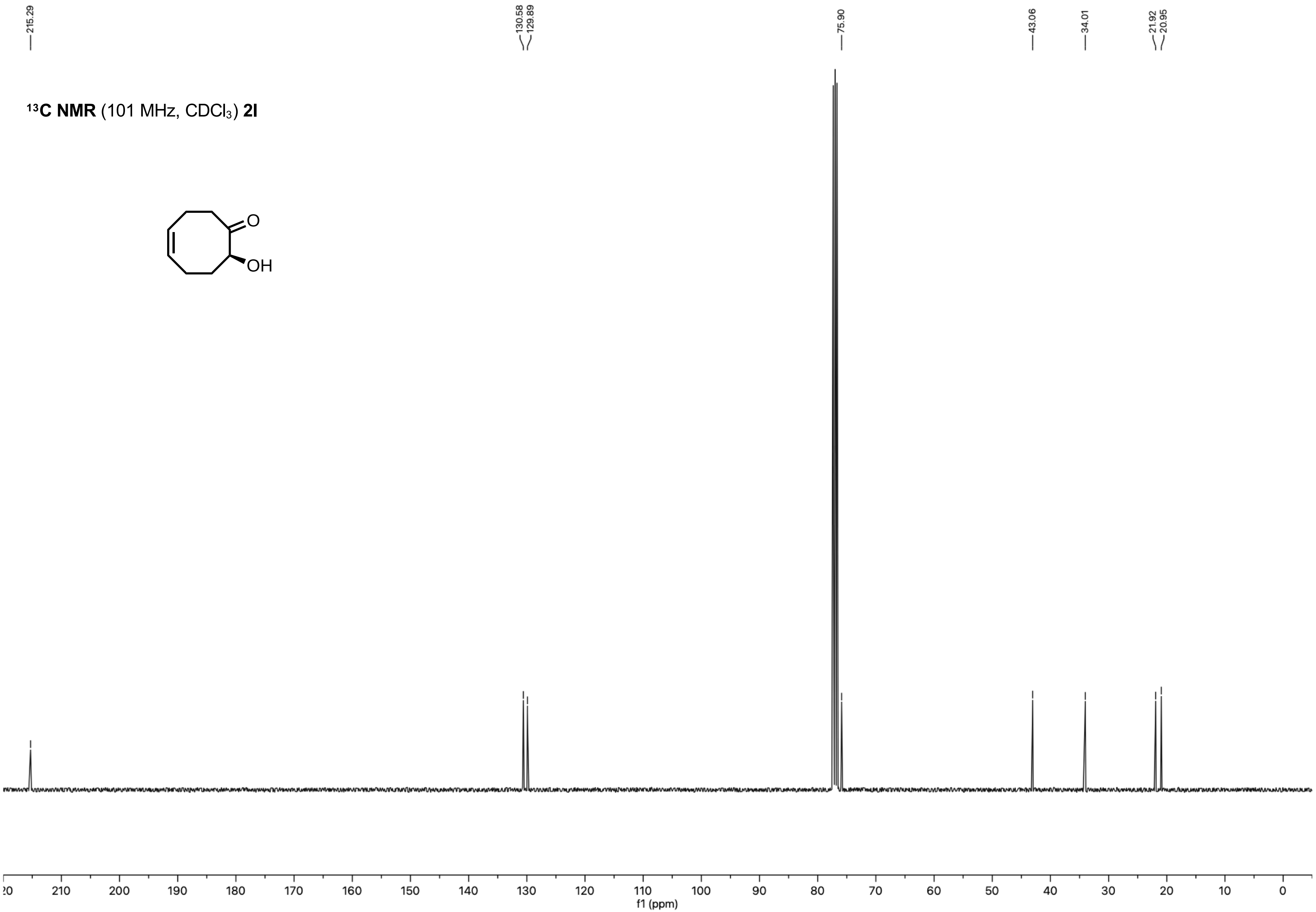
215.29
130.58
129.89
75.90
43.06
34.01
21.92
20.95
^{13}C NMR (101 MHz, $CDCl_3$) 2l
O
OH
210
200
190
180
170
160
150
140
130
120
110
100
90
80
70
60
50
40
30
20
10
0
f1 (ppm)

¹H NMR (700 MHz, $CDCl_3$) **2m**

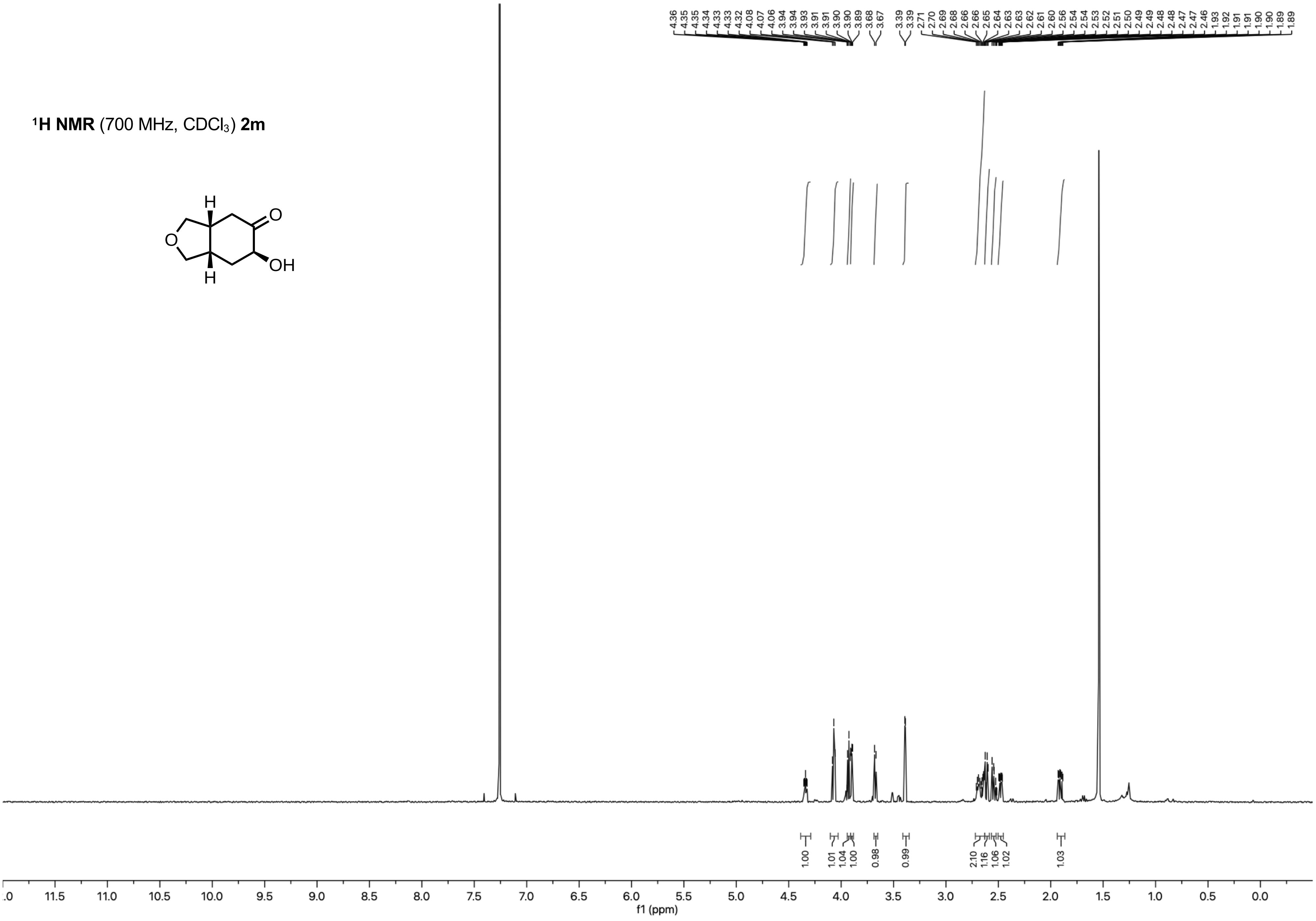

^{13}C NMR (176 MHz, $CDCl_3$) **2m**

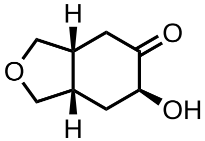

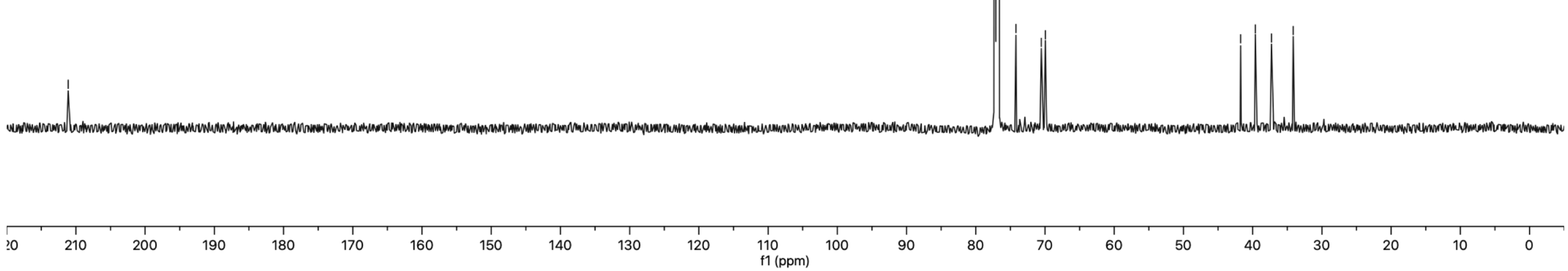

¹H NMR (700 MHz, $CDCl_3$) **2n**

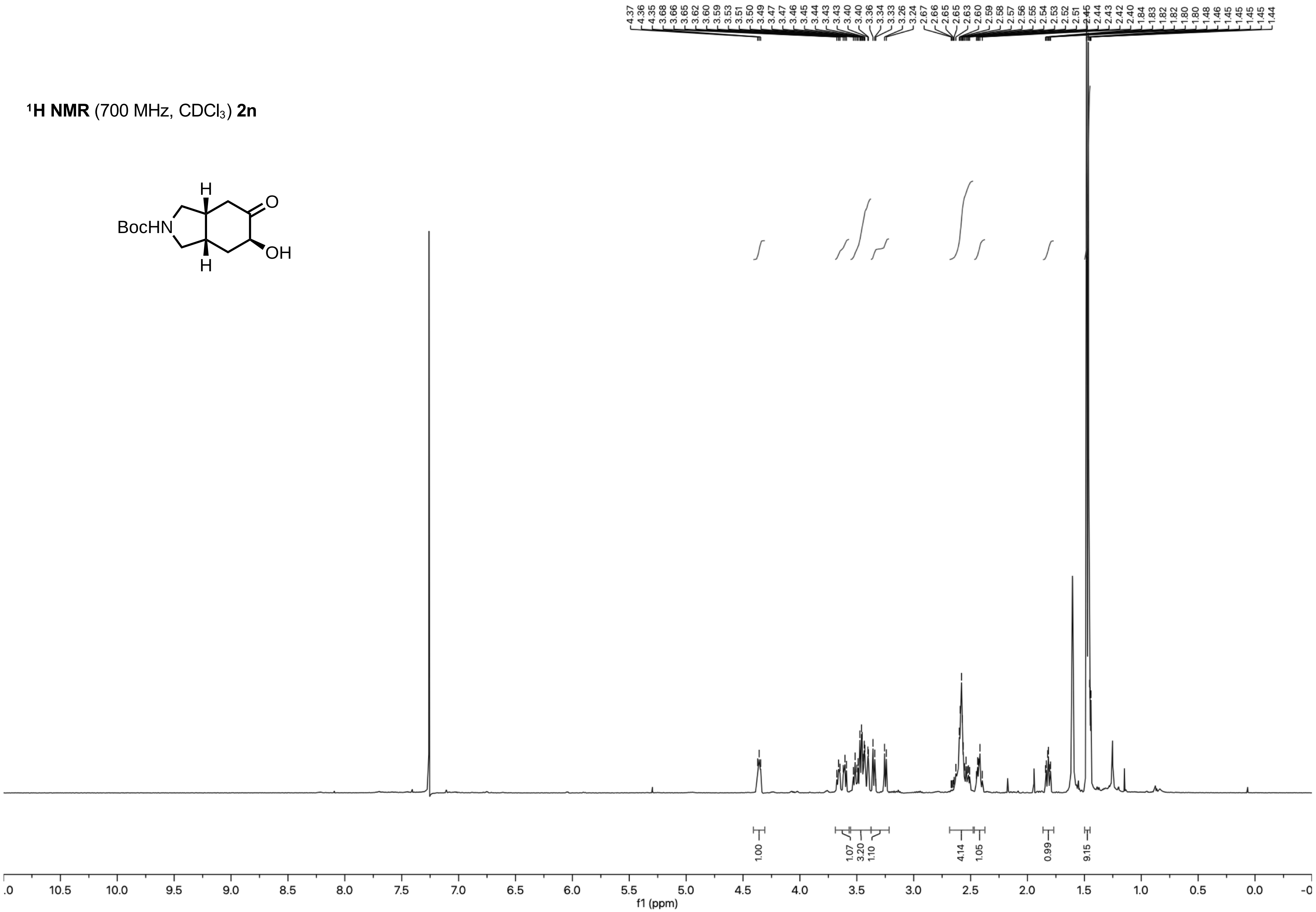

^{13}C NMR (176 MHz, $CDCl_3$) **2n**

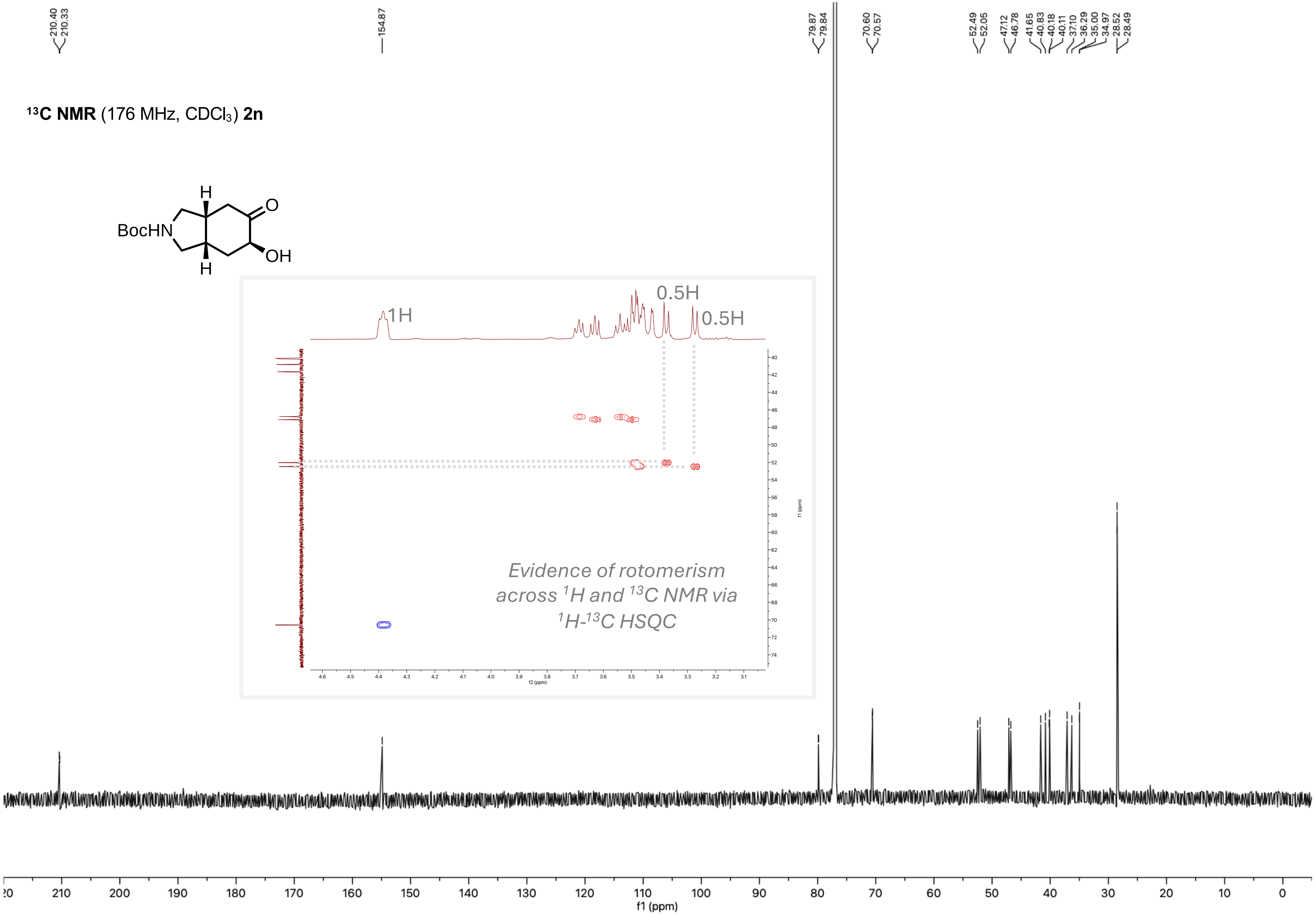

¹H NMR (700 MHz, $CDCl_3$) **2o**

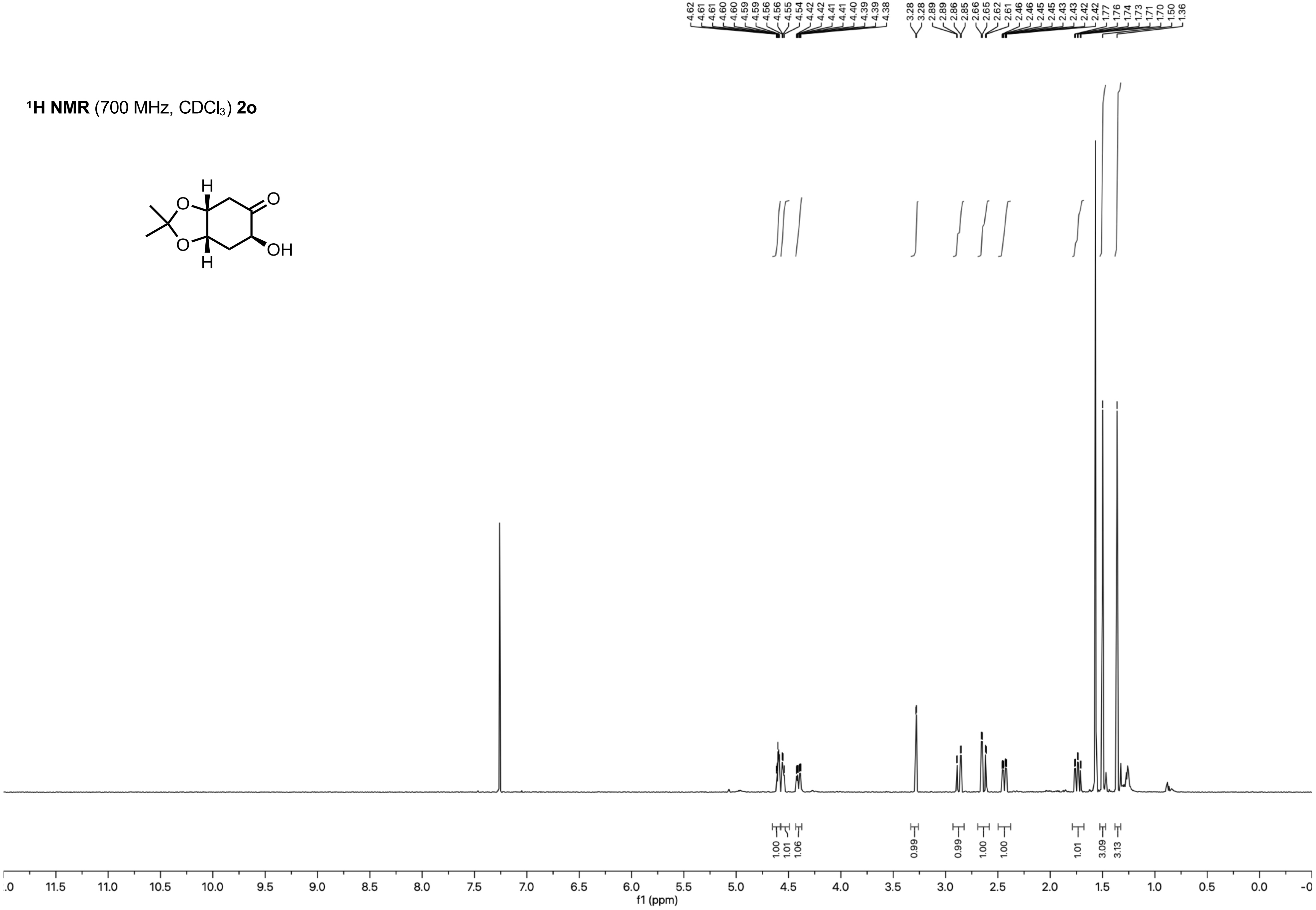

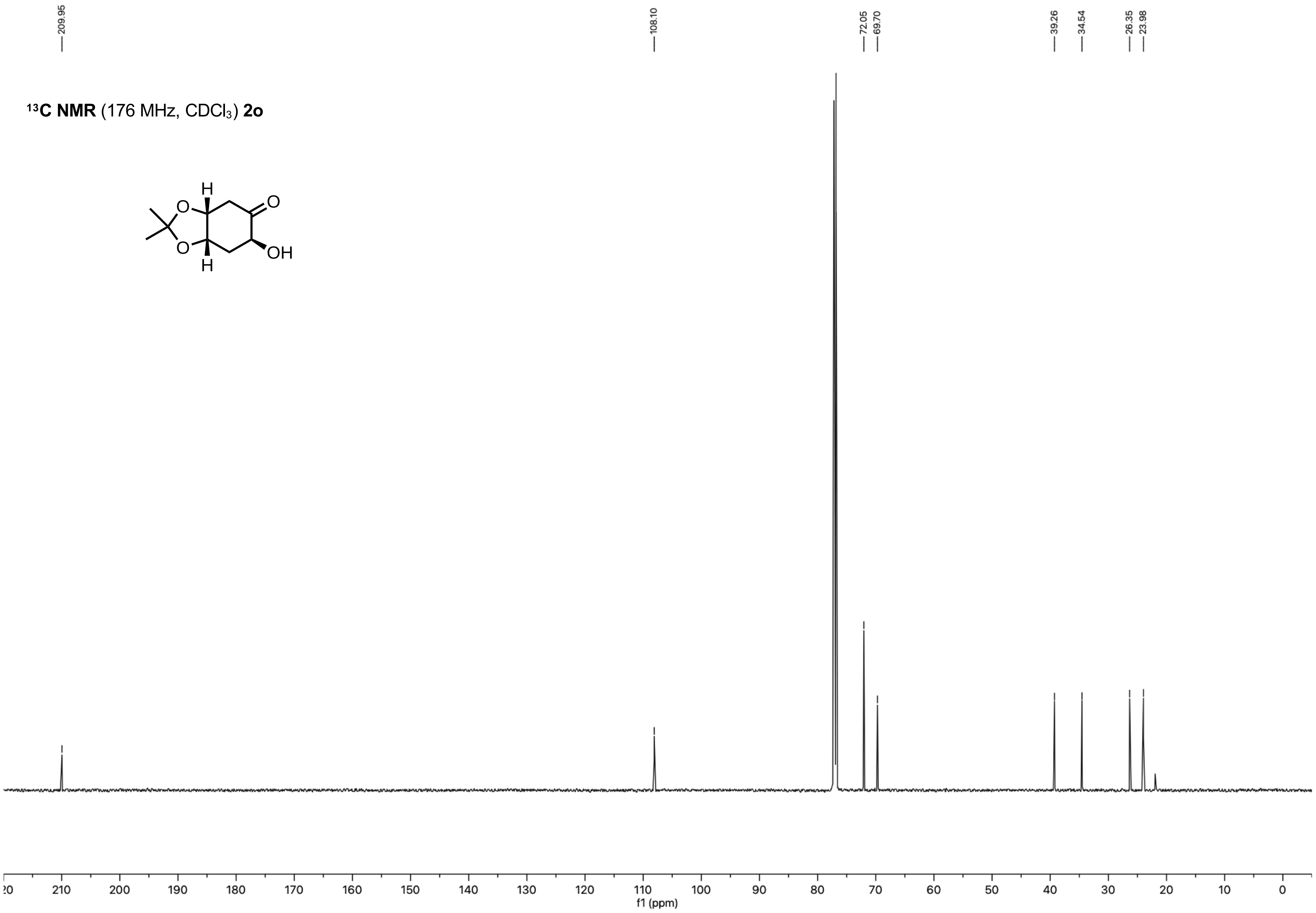

13C NMR (176 MHz, CDCl3) 2o
209.95
108.10
72.05
69.70
39.26
34.54
26.35
23.98
H
O
O
O
H
OH
20 210 200 190 180 170 160 150 140 130 120 110 100 90 80 70 60 50 40 30 20 10 0
f1 (ppm)

^{1}H NMR (700 MHz, $CDCl_3$) **2p**

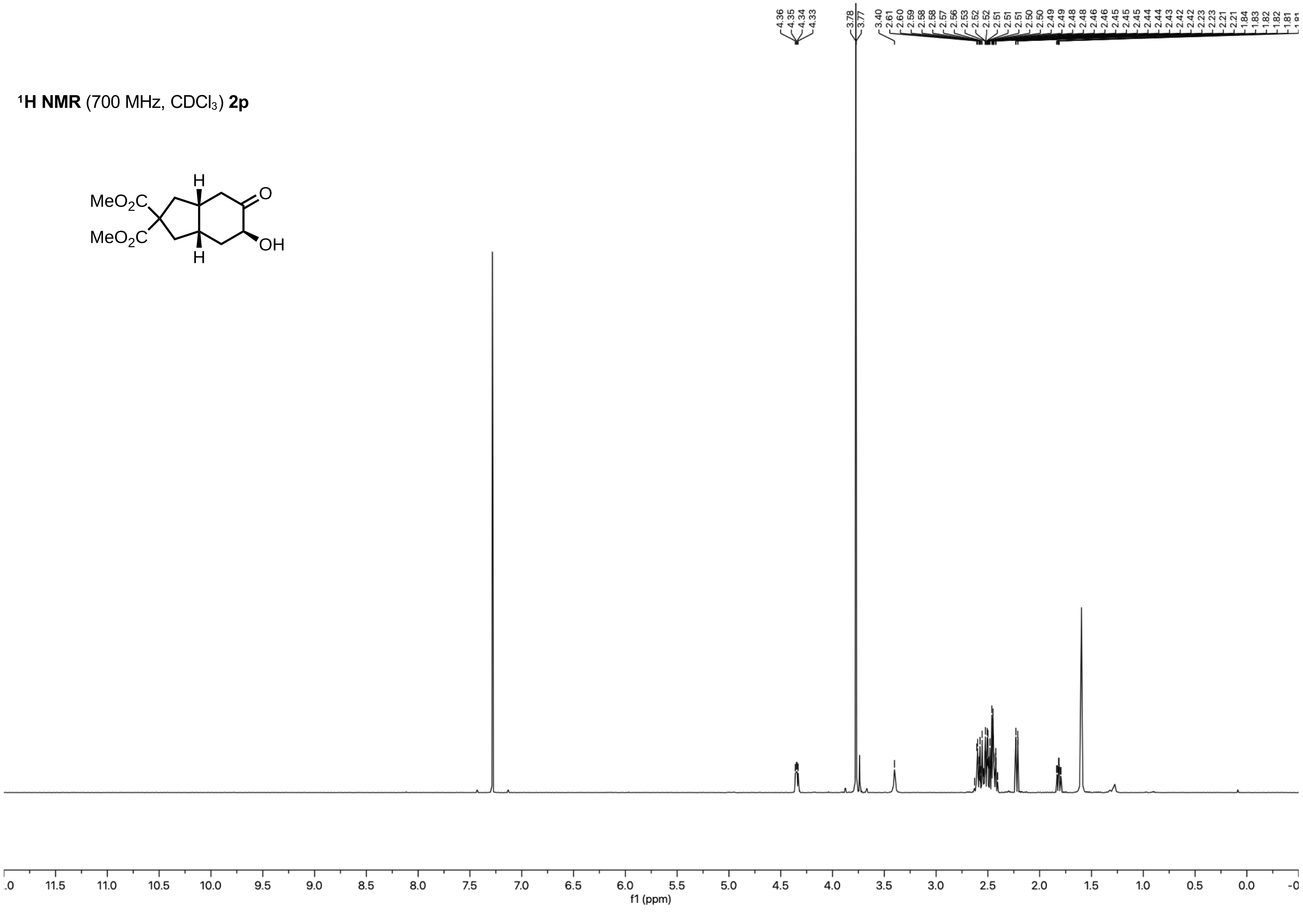

^{13}C NMR (176 MHz, $CDCl_3$) **2p**

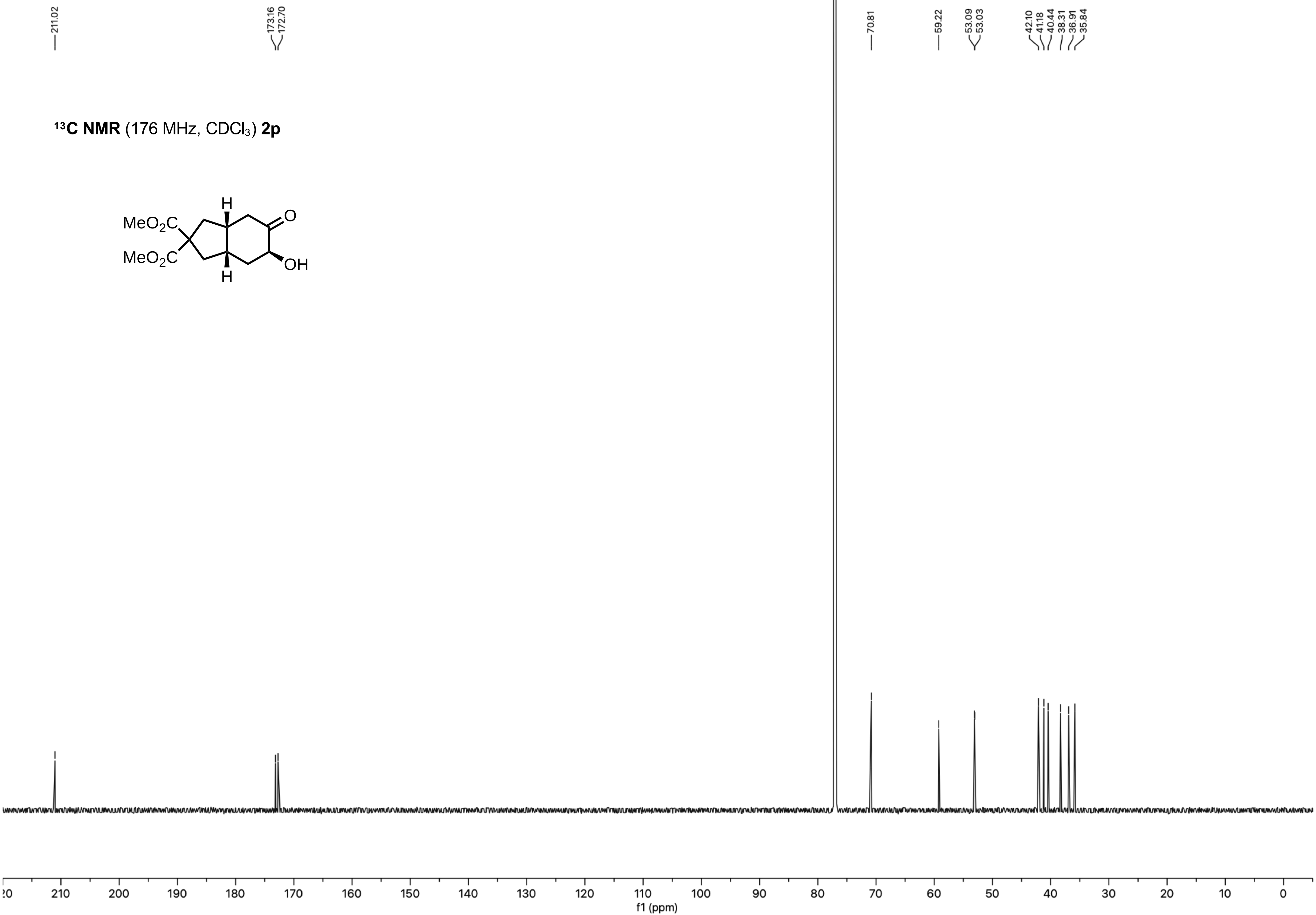

¹H NMR (700 MHz, CDCl₃) **2q**

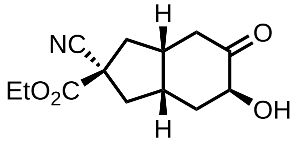

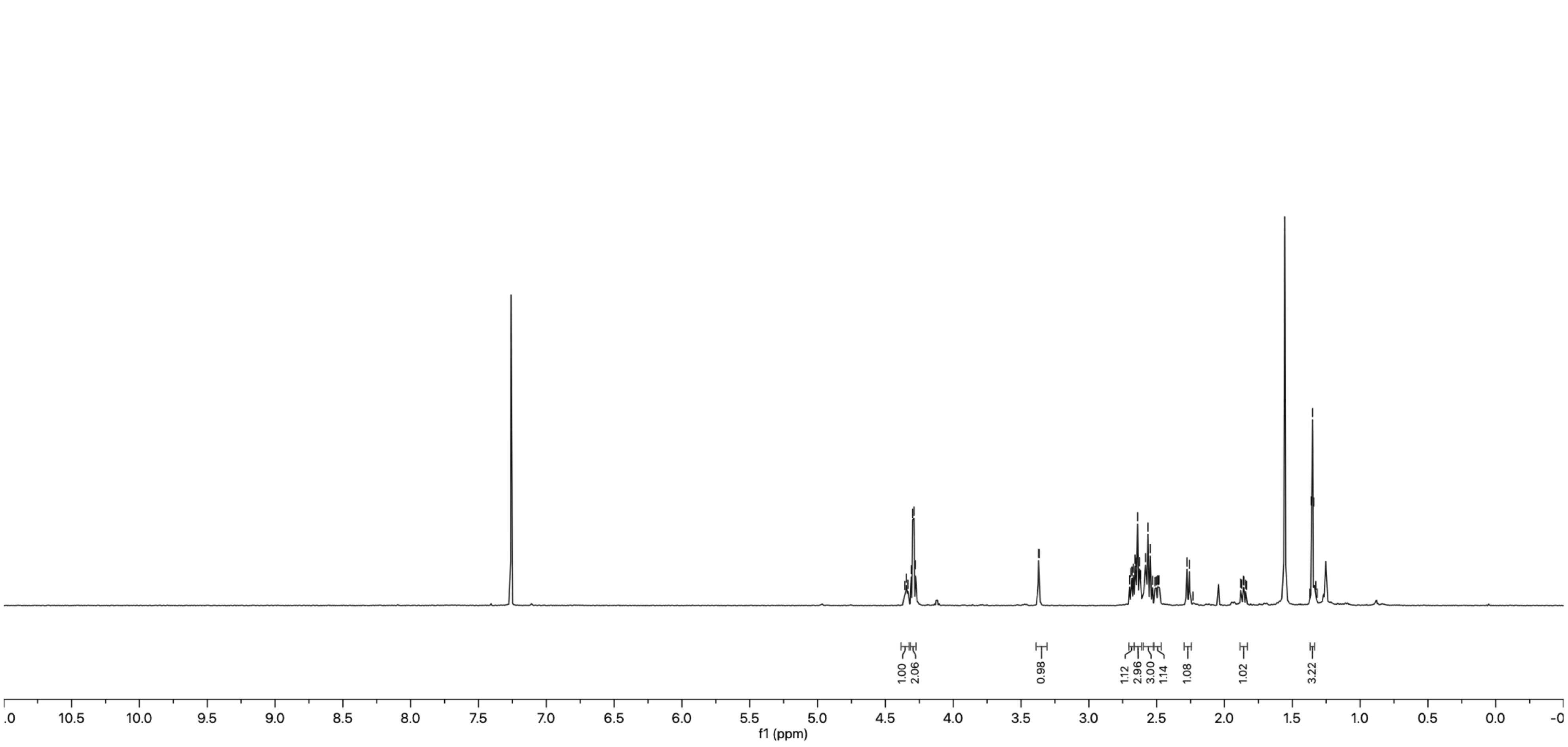

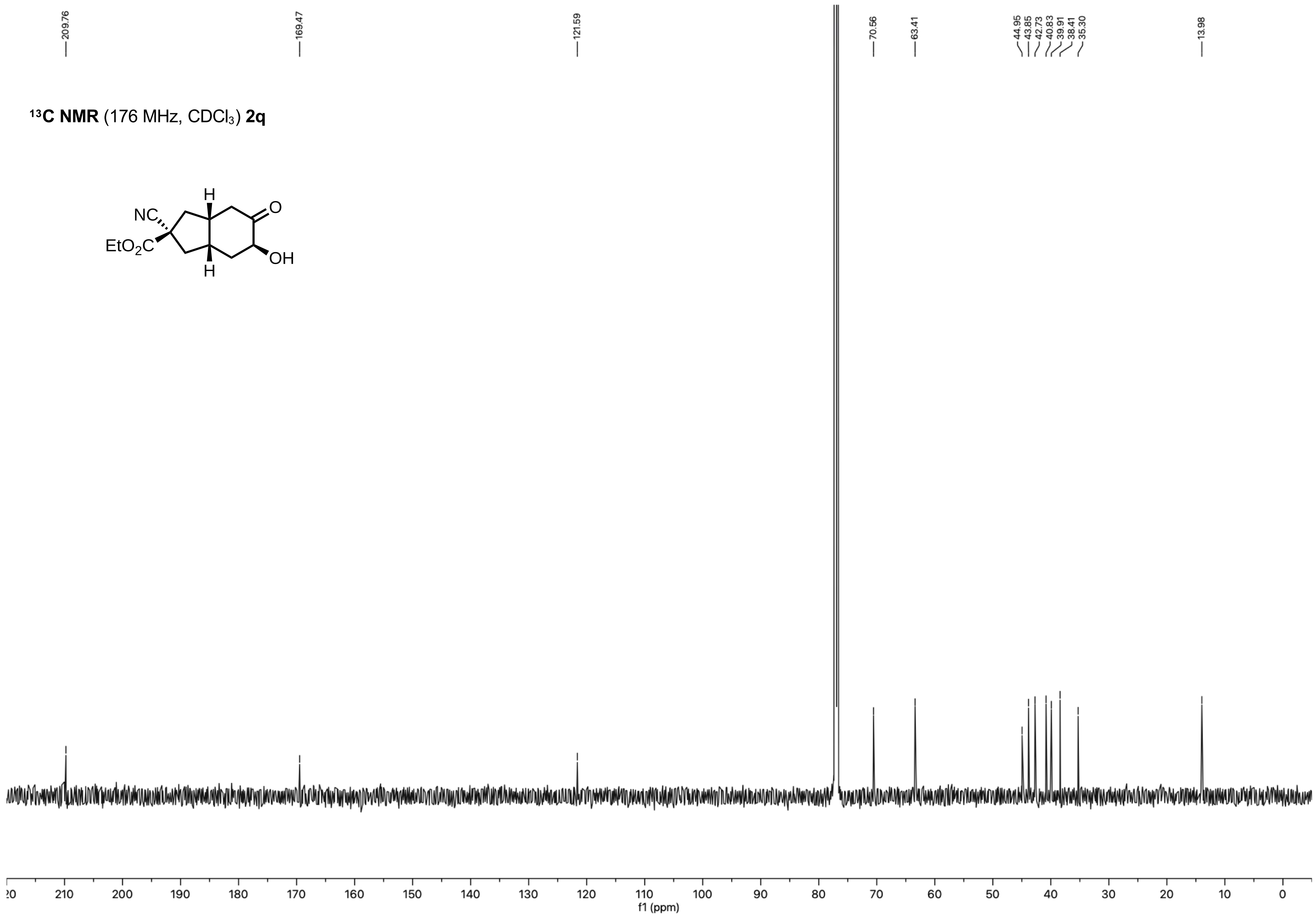

209.76
169.47
121.59
70.56
63.41
44.95
43.85
42.73
40.83
39.91
38.41
35.30
13.98
¹³C NMR (176 MHz, CDCl₃) 2q
NC
EtO₂C
H
H
O
OH
f1 (ppm)

^{1}**H NMR** (400 MHz, $CDCl_3$) **2r**

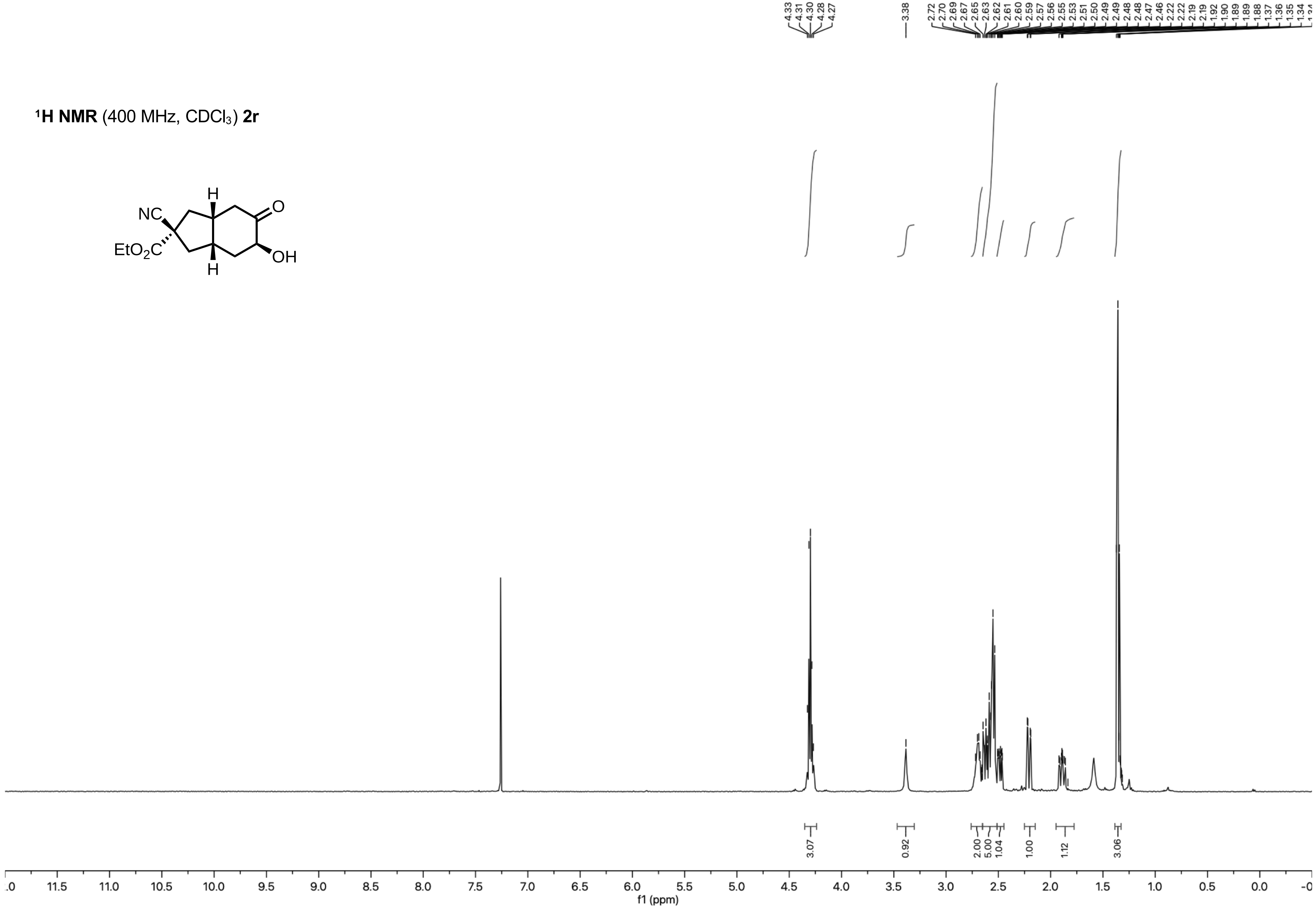

^{13}C NMR (101 MHz, $CDCl_3$) **2r**

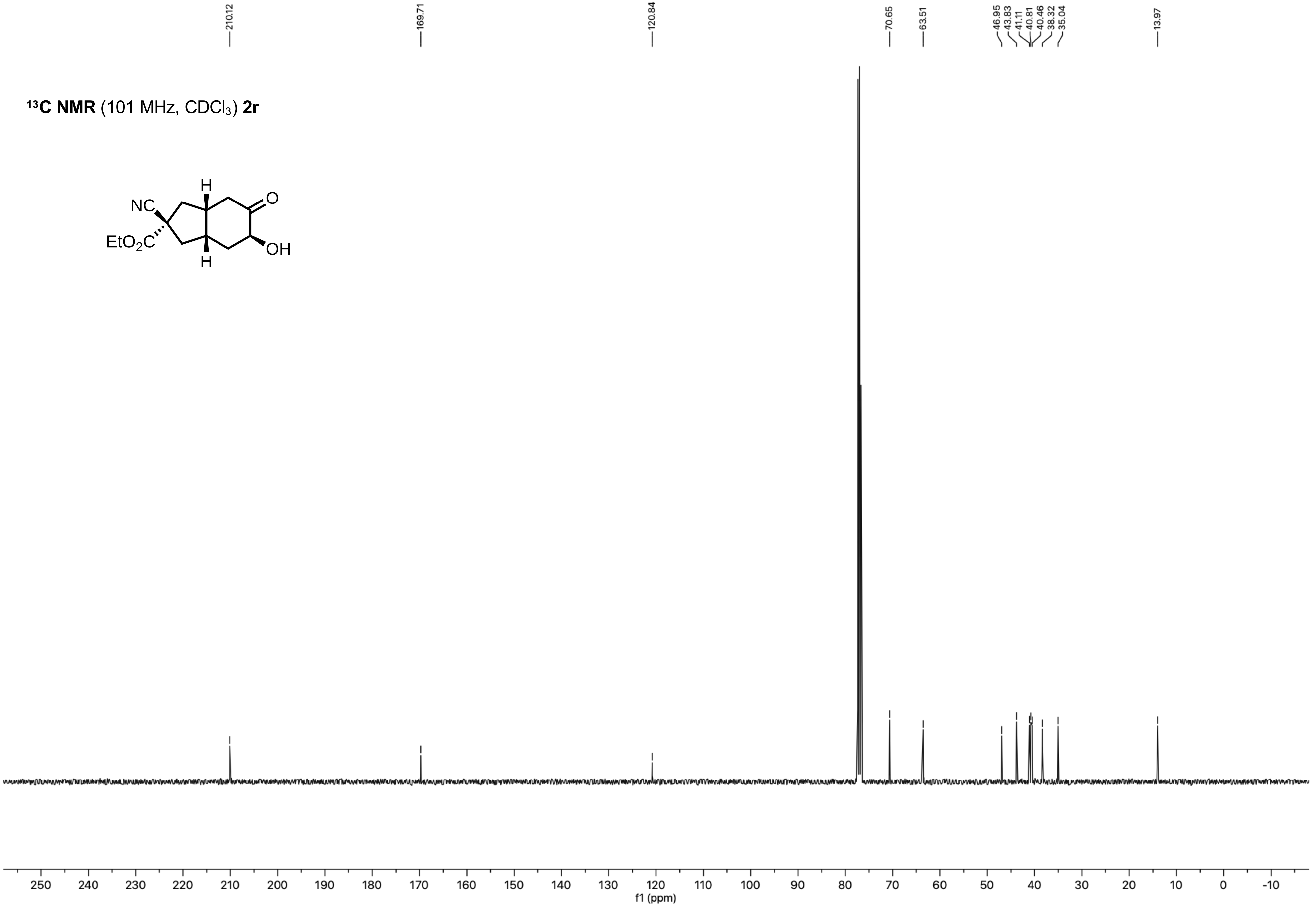

¹H NMR (700 MHz, CDCl₃) **2s**

**ca. 1:1.3 mixture of epimers*

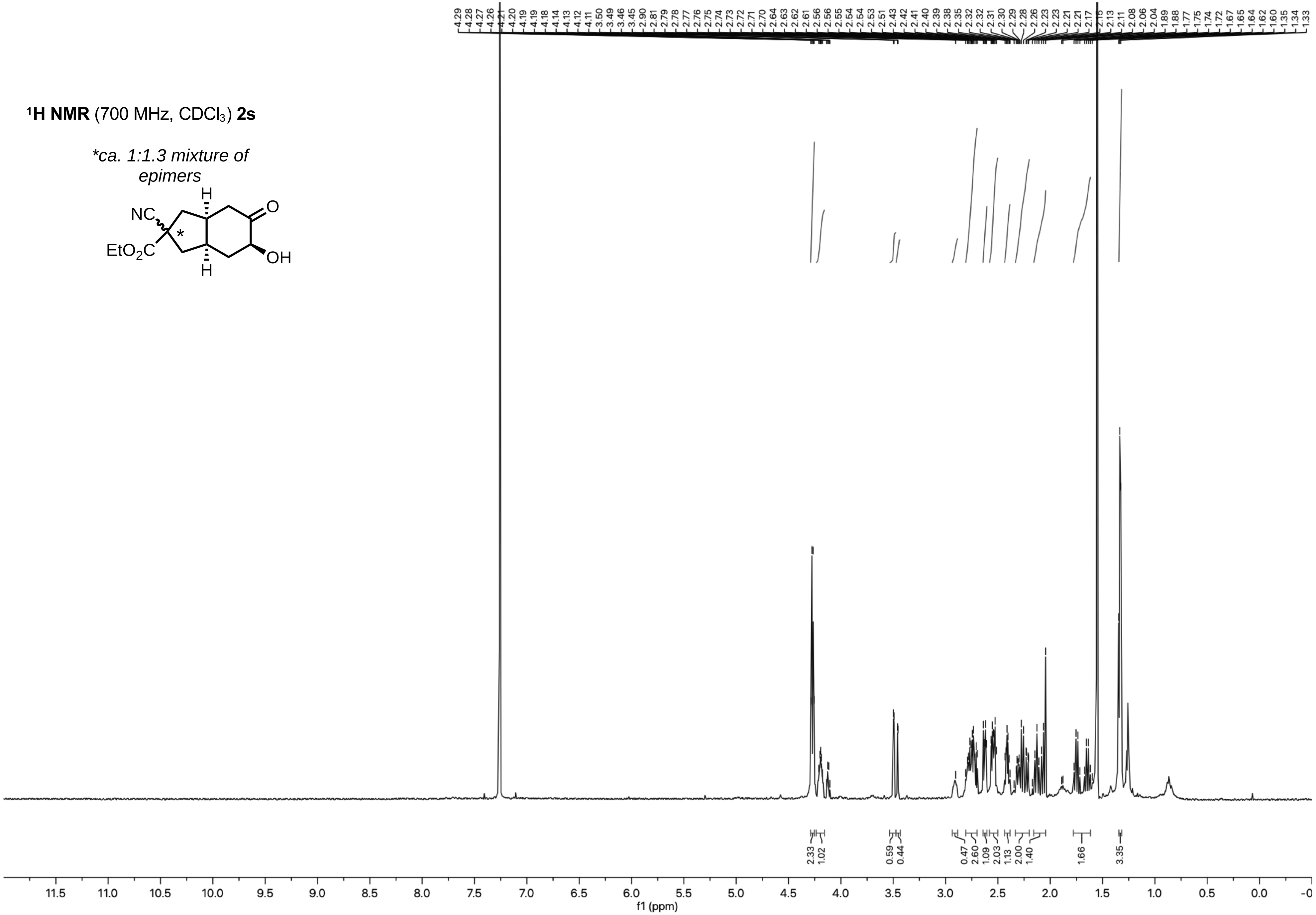

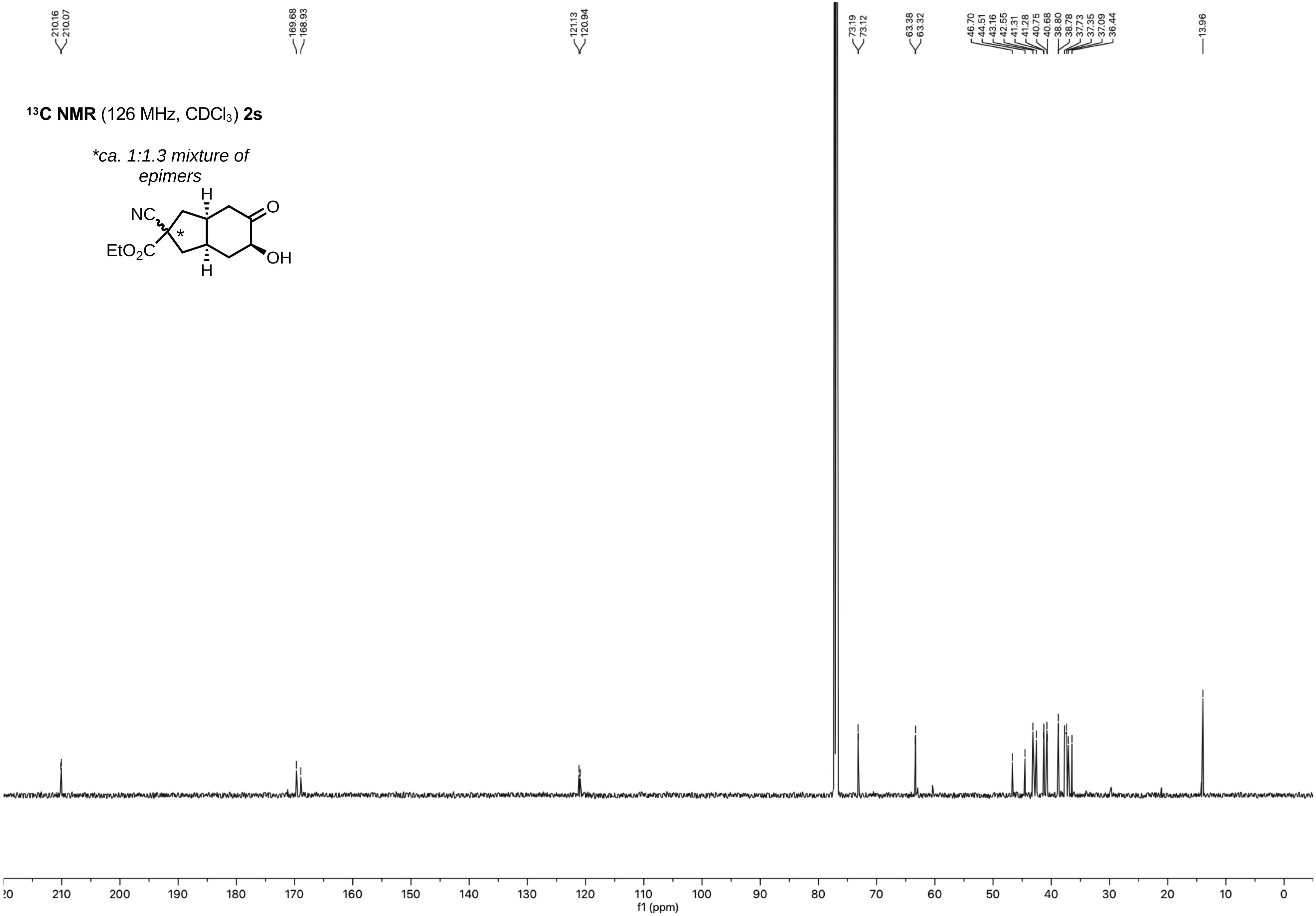

13C NMR (126 MHz, CDCl3) 2s
*ca. 1:1.3 mixture of epimers
NC
EtO2C
H
H
O
OH
*
210.16
210.07
169.68
168.93
121.13
120.94
73.19
73.12
63.38
63.32
46.70
44.51
43.16
42.55
41.31
41.28
40.75
40.68
38.80
38.78
37.73
37.35
37.09
36.44
13.96
20
210
200
190
180
170
160
150
140
130
120
110
100
90
80
70
60
50
40
30
20
10
0
f1 (ppm)

^{1}H NMR (700 MHz, $CDCl_3$) **2t**

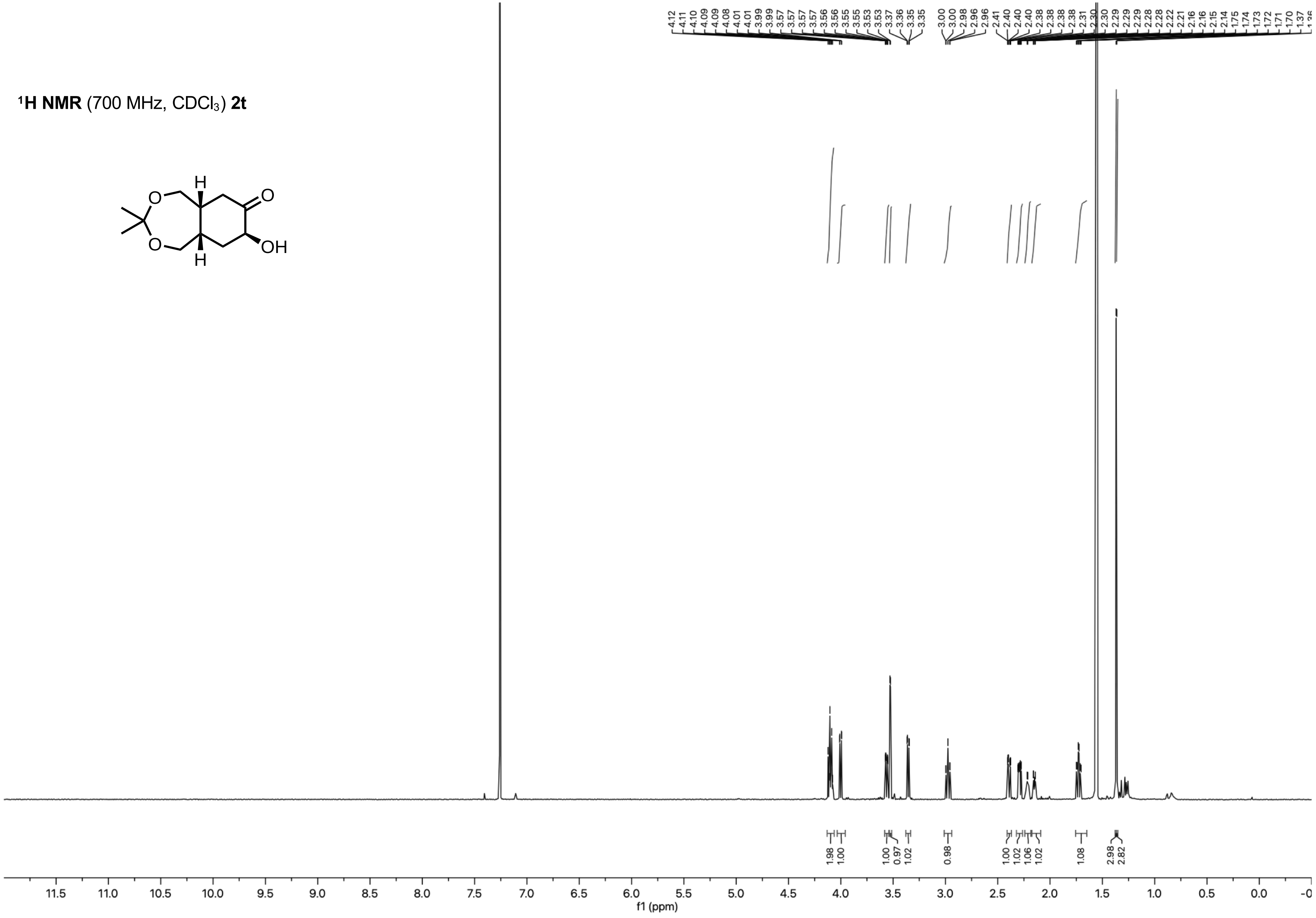

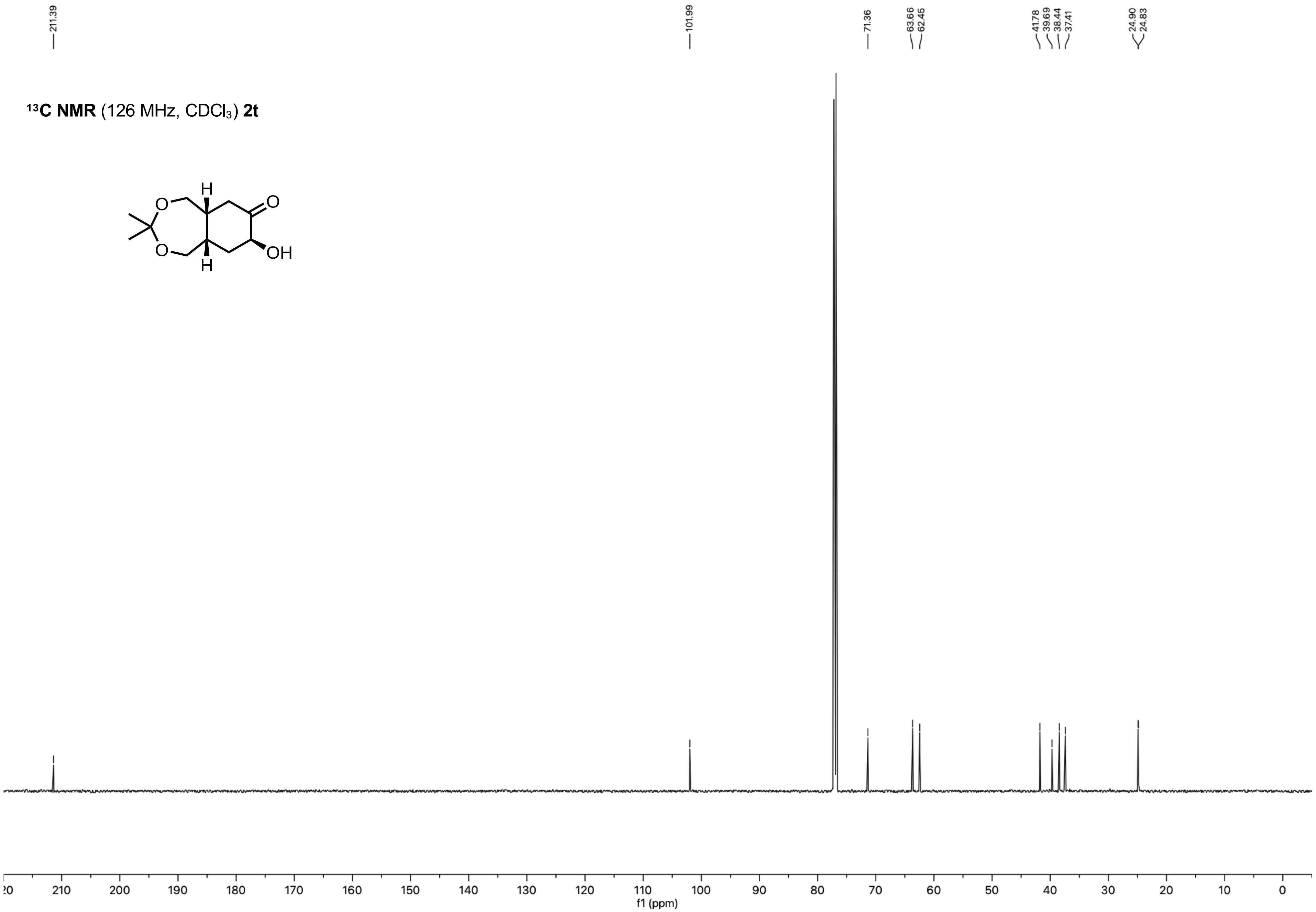

211.39
101.99
71.36
63.66
62.45
41.78
39.69
38.44
37.41
24.90
24.83
¹³C NMR (126 MHz, CDCl₃) 2t
H
O
O
OH
H
O
20
210
200
190
180
170
160
150
140
130
120
110
100
90
80
70
60
50
40
30
20
10
0
f1 (ppm)

^{1}**H NMR** (700 MHz, $CDCl_3$) **2u**

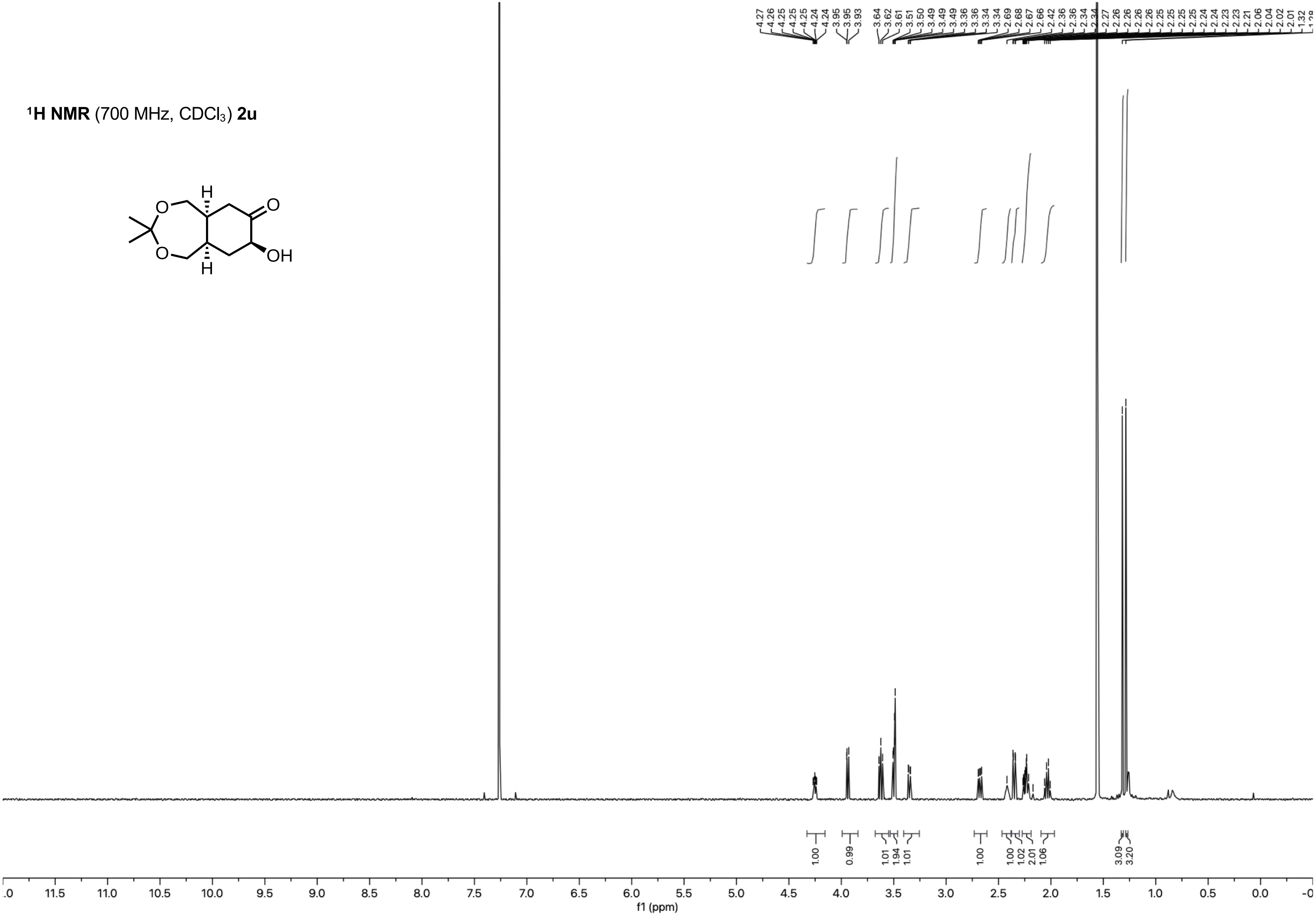

^{13}C NMR (126 MHz, $CDCl_3$) **2u**

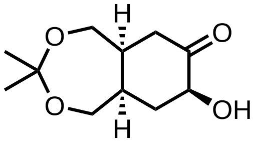

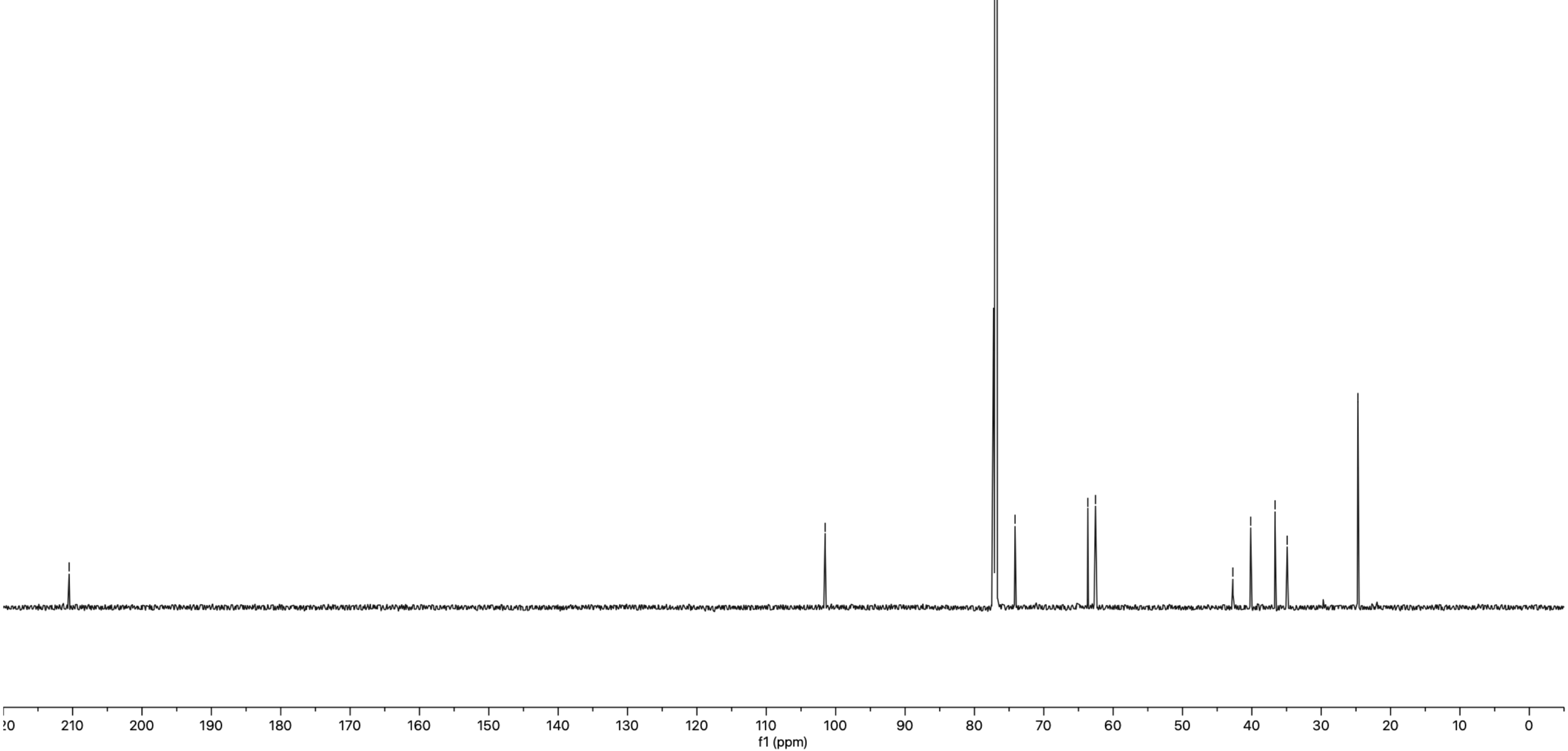

¹H NMR (700 MHz, $CDCl_3$) **2v**

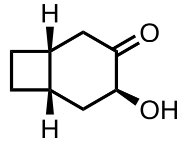

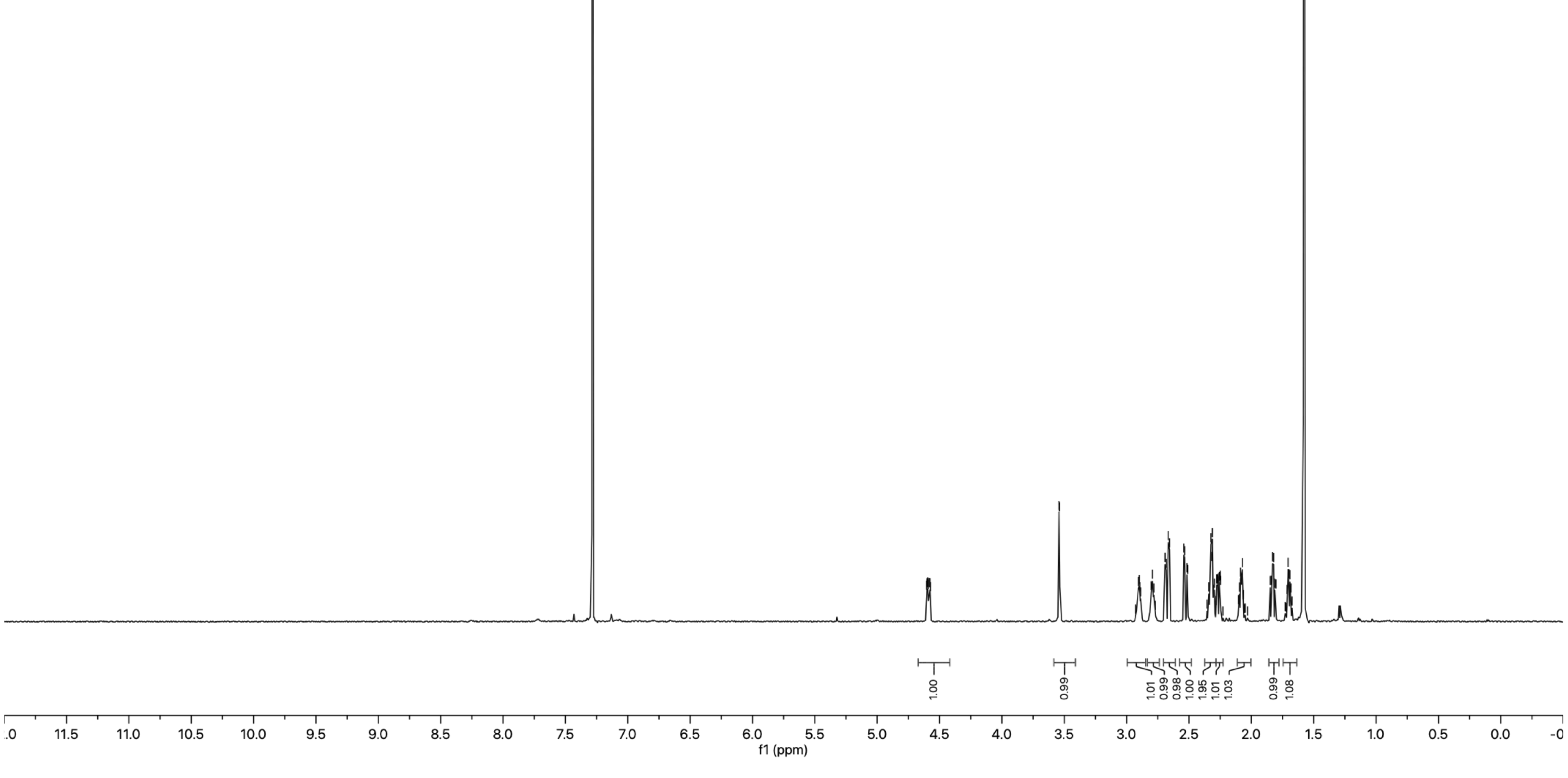

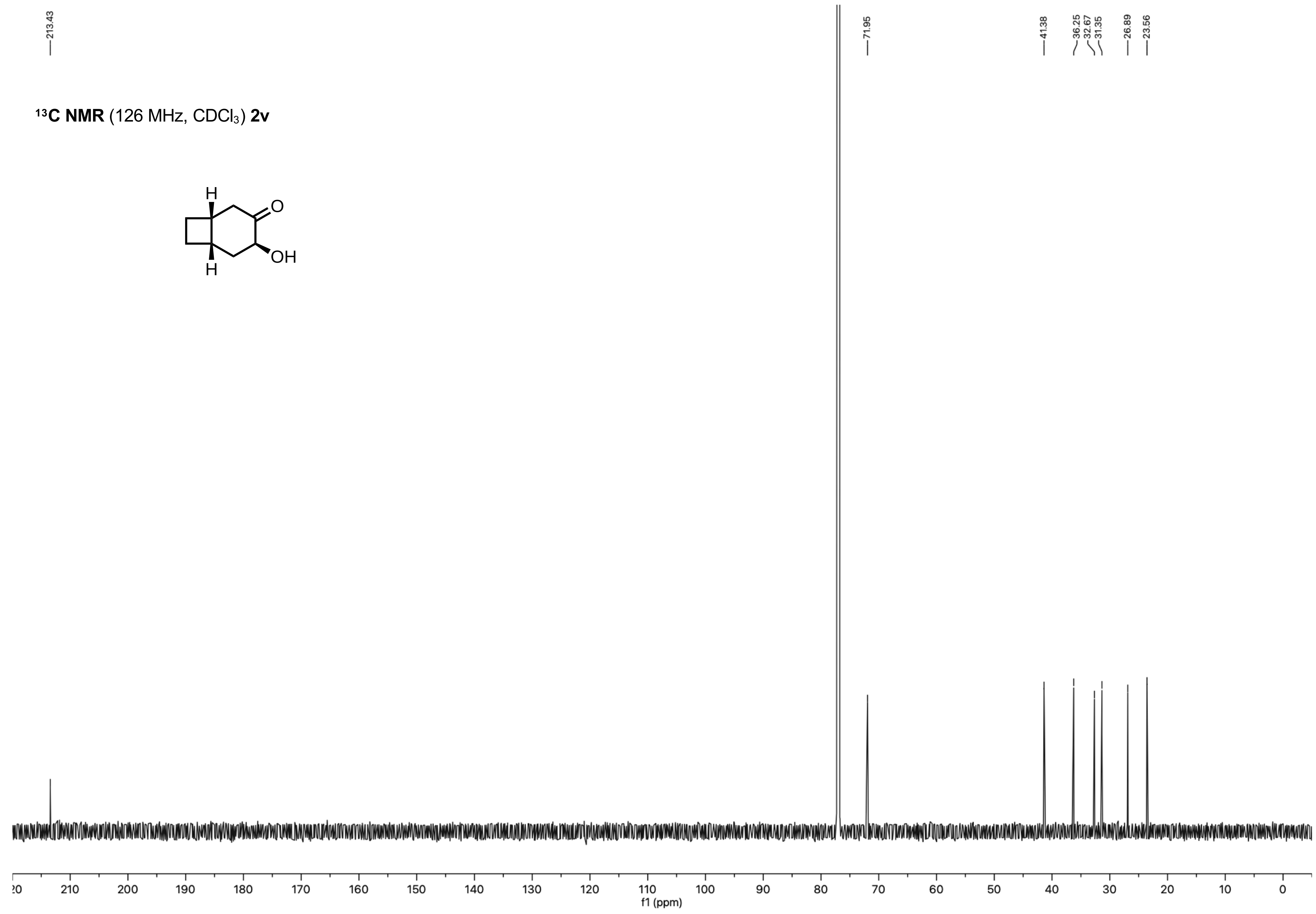
213.43
71.95
41.38
36.25
32.67
31.35
26.89
23.56
¹³C NMR (126 MHz, CDCl₃) 2v
H
O
OH
H
210
200
190
180
170
160
150
140
130
120
110
100
90
80
70
60
50
40
30
20
10
0
f1 (ppm)

¹H NMR (700 MHz, $CDCl_3$) **2w'**

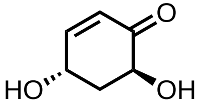

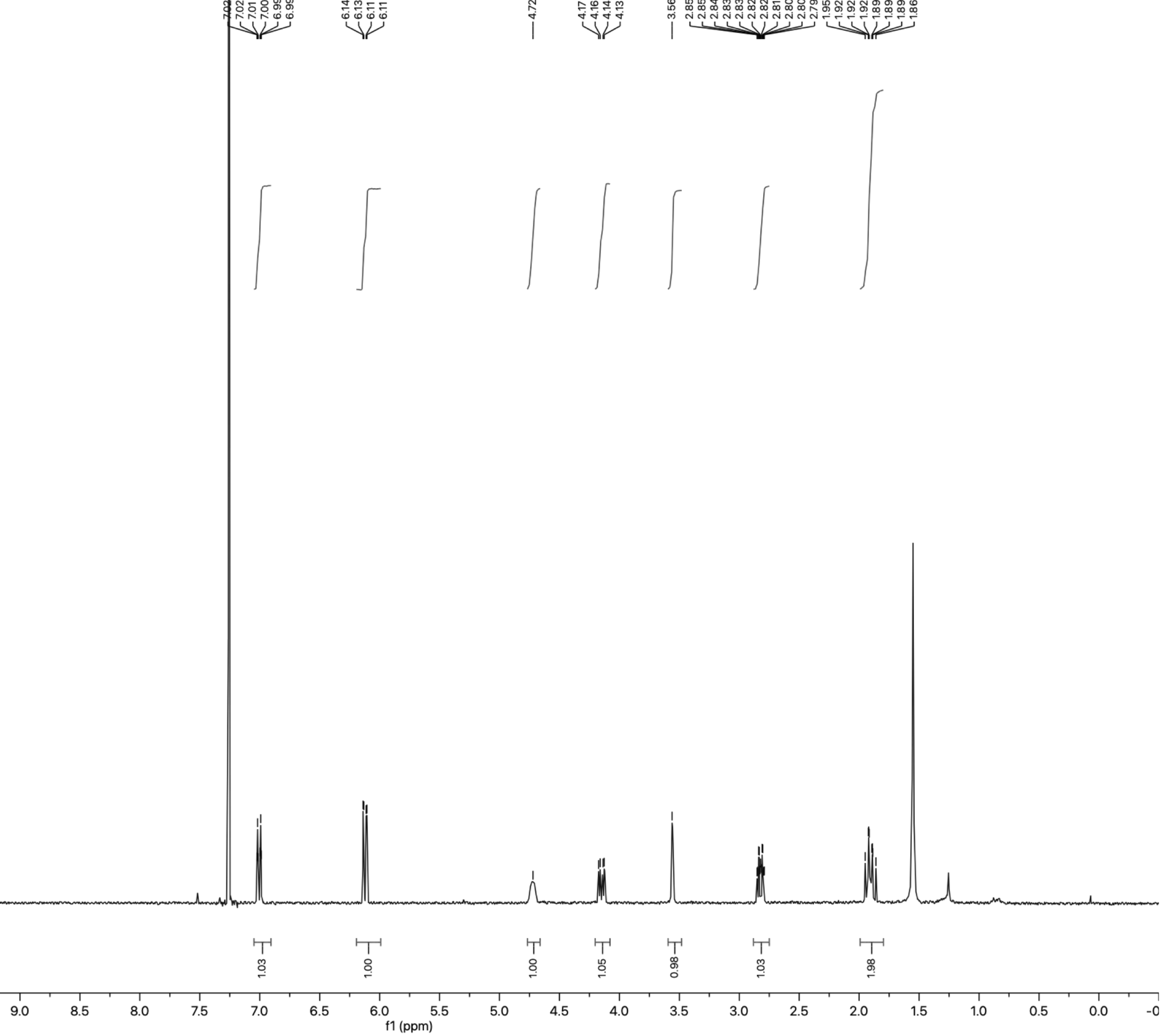

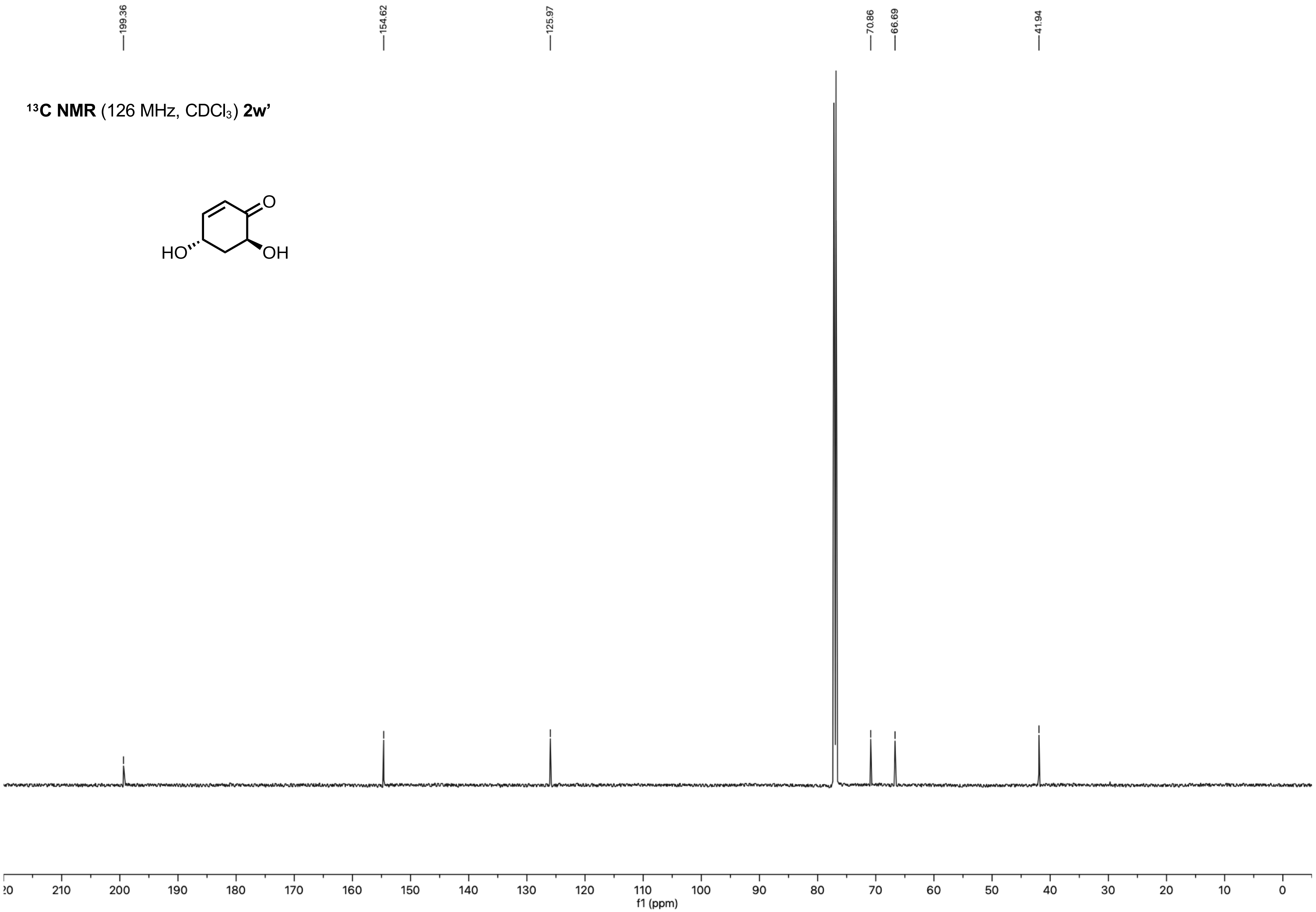

^{13}C NMR (126 MHz, $CDCl_3$) 2w'
199.36
154.62
125.97
70.86
66.69
41.94
HO
OH
O
20
210
200
190
180
170
160
150
140
130
120
110
100
90
80
70
60
50
40
30
20
10
0
f1 (ppm)

¹H NMR (700 MHz, $CDCl_3$) **2x**

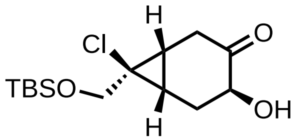

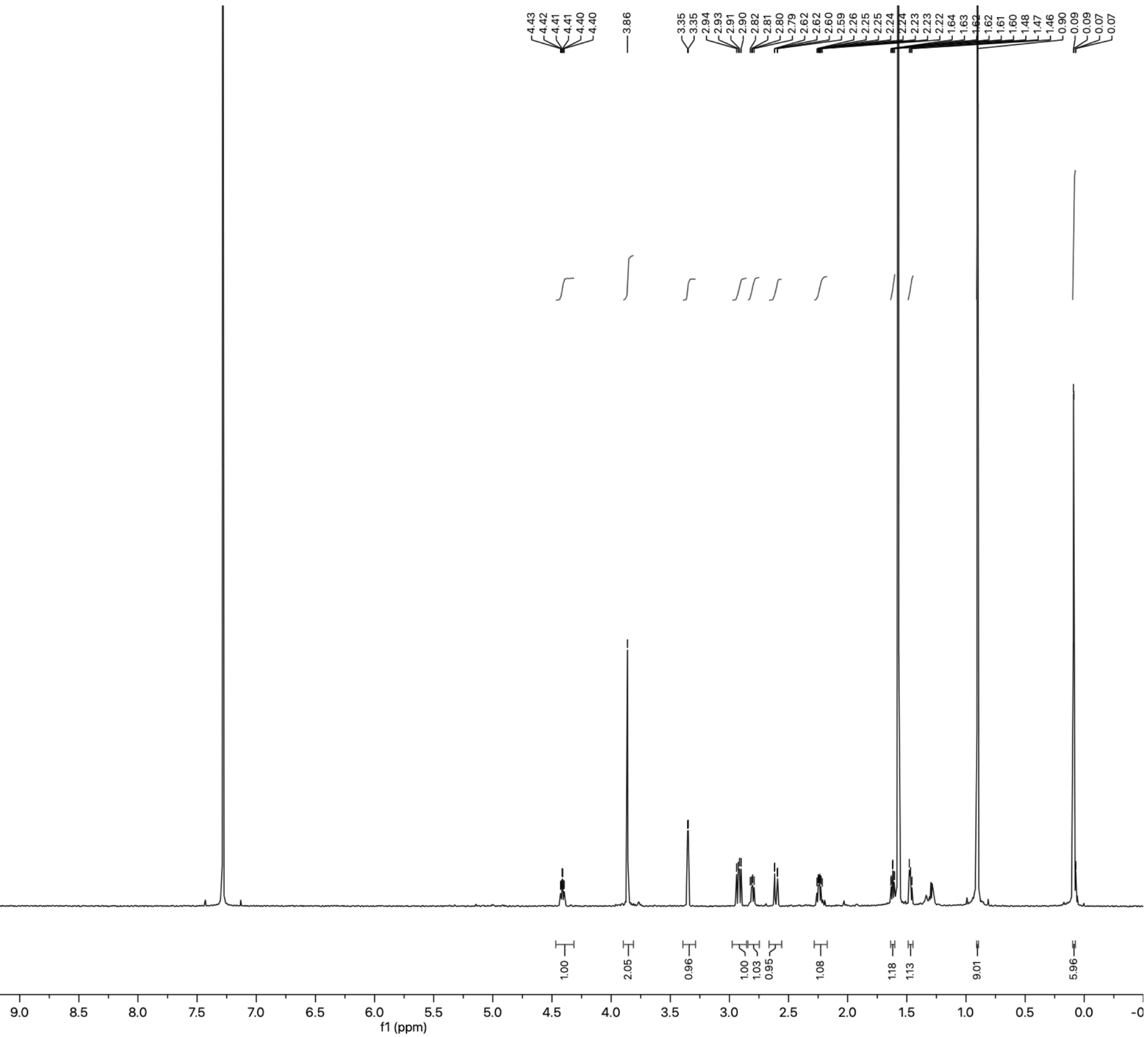

^{13}C NMR (126 MHz, $CDCl_3$) **2x**

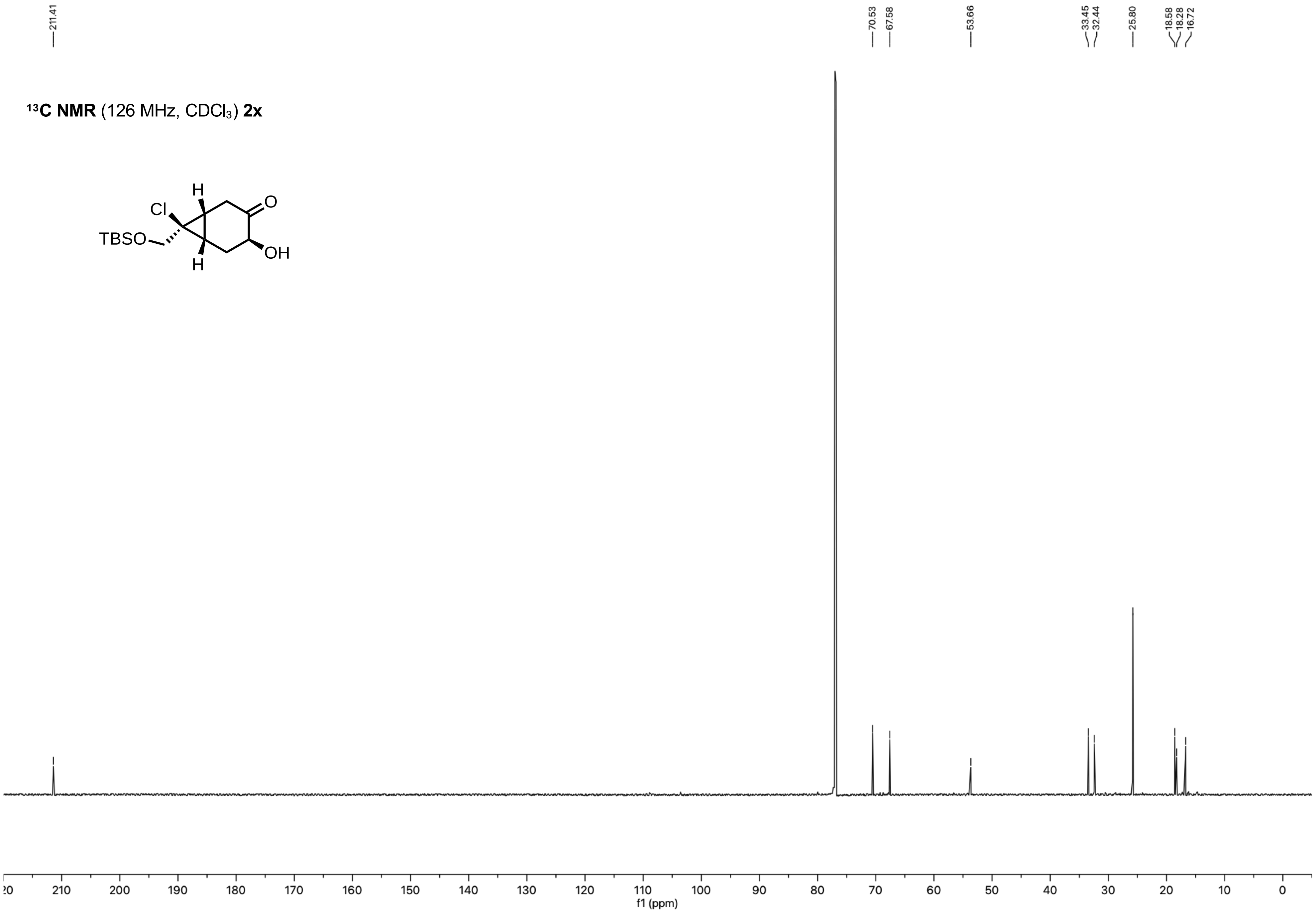

¹H NMR (700 MHz, CDCl₃) **2y**

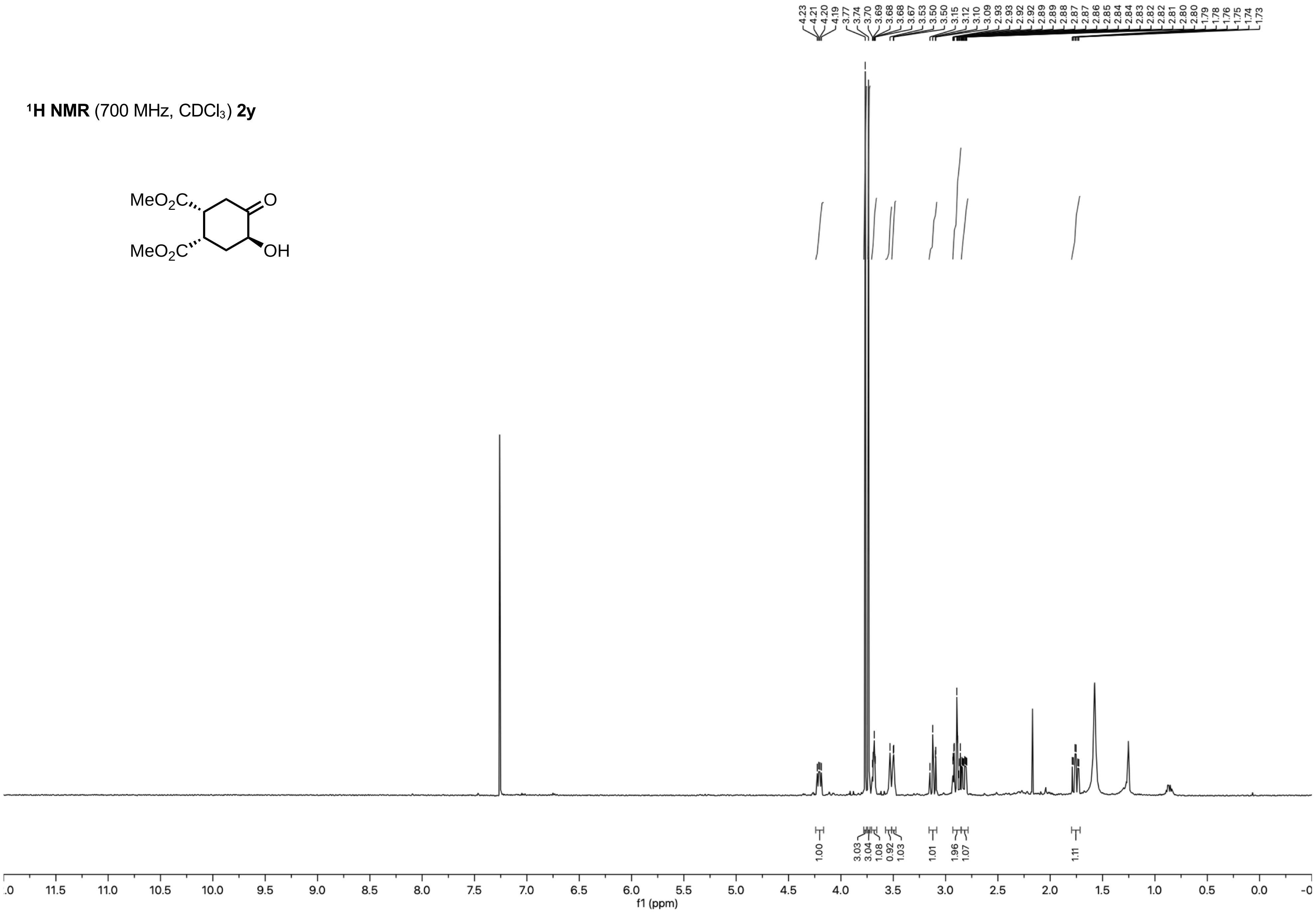

^{13}C NMR (126 MHz, $CDCl_3$) **2y**

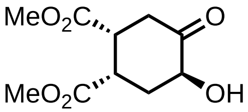

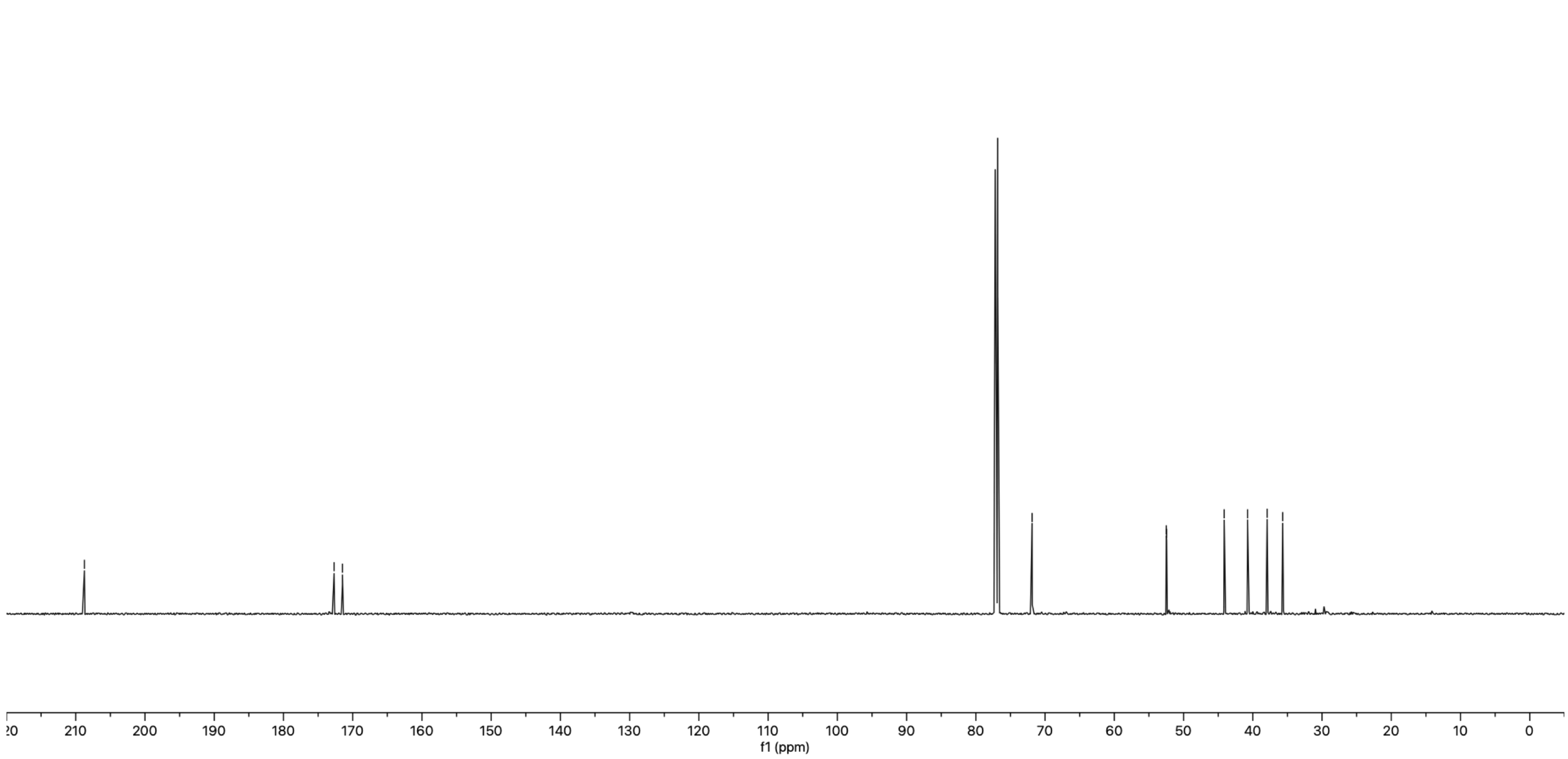

¹H NMR (700 MHz, $CDCl_3$) **2z**

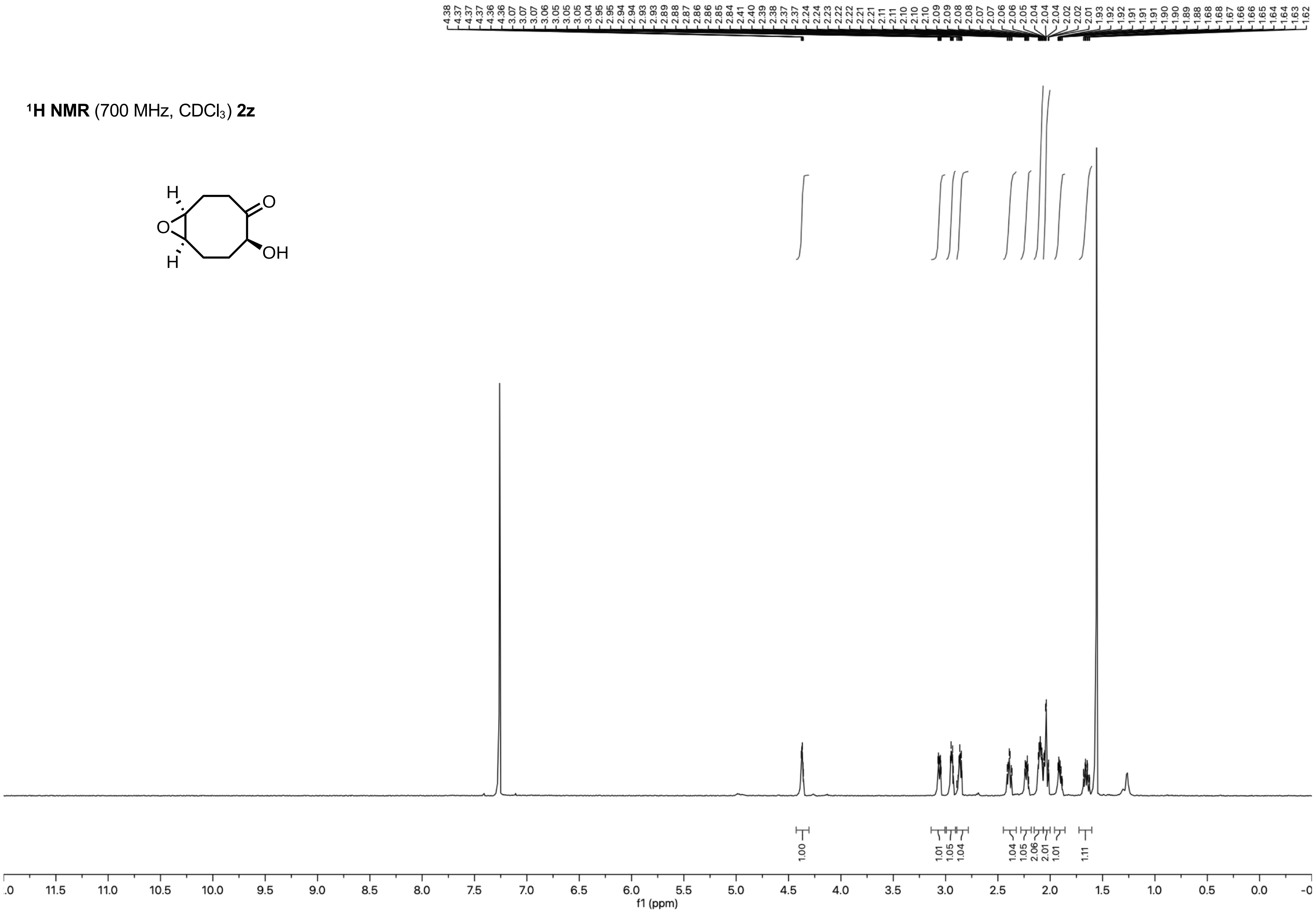

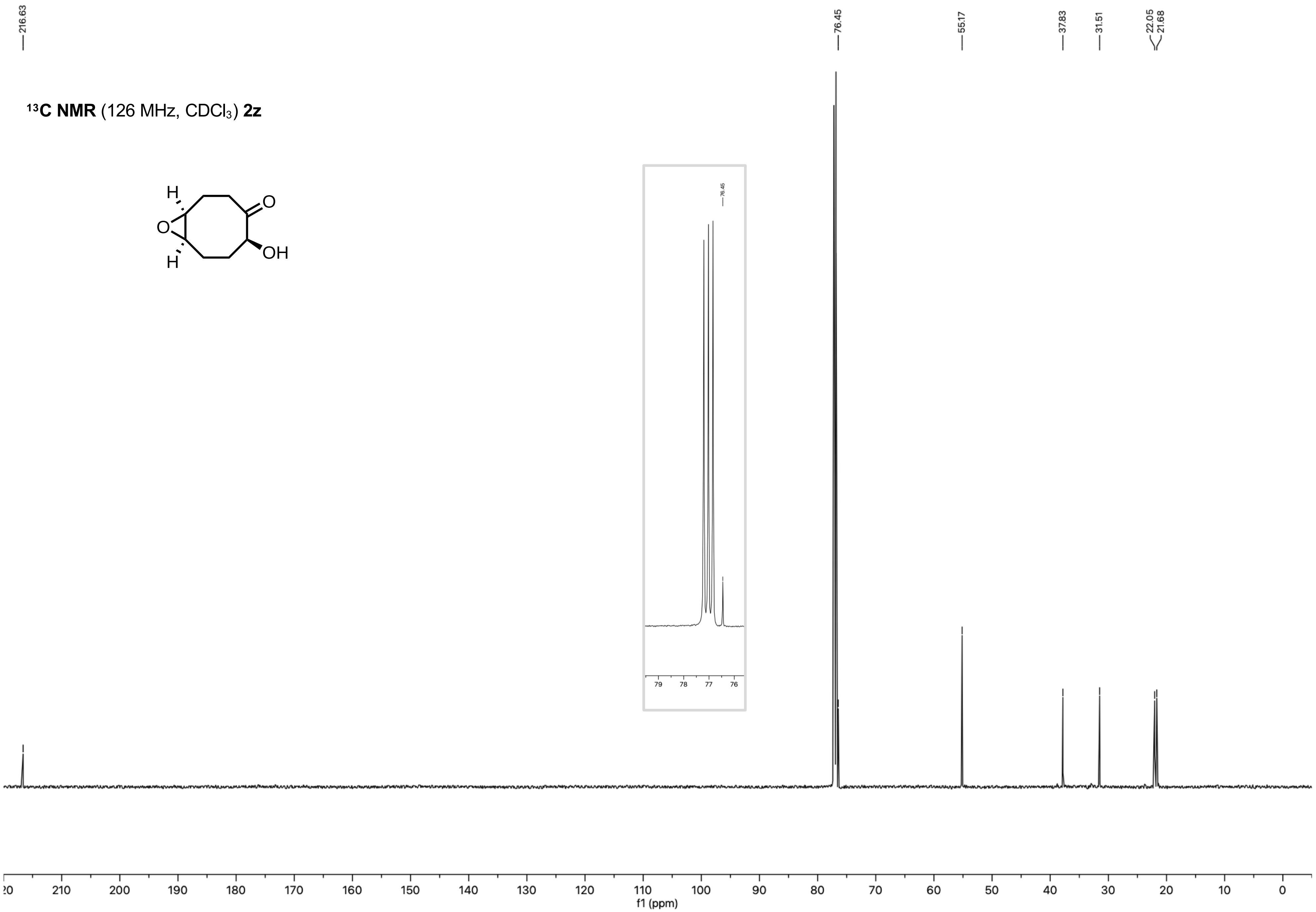

216.63
76.45
55.17
37.83
31.51
22.05
21.68
^{13}C NMR (126 MHz, CDCl$_3$) 2z
H
O
H
O
OH
76.45
79
78
77
76
210
200
190
180
170
160
150
140
130
120
110
100
90
80
70
60
50
40
30
20
10
0
f1 (ppm)

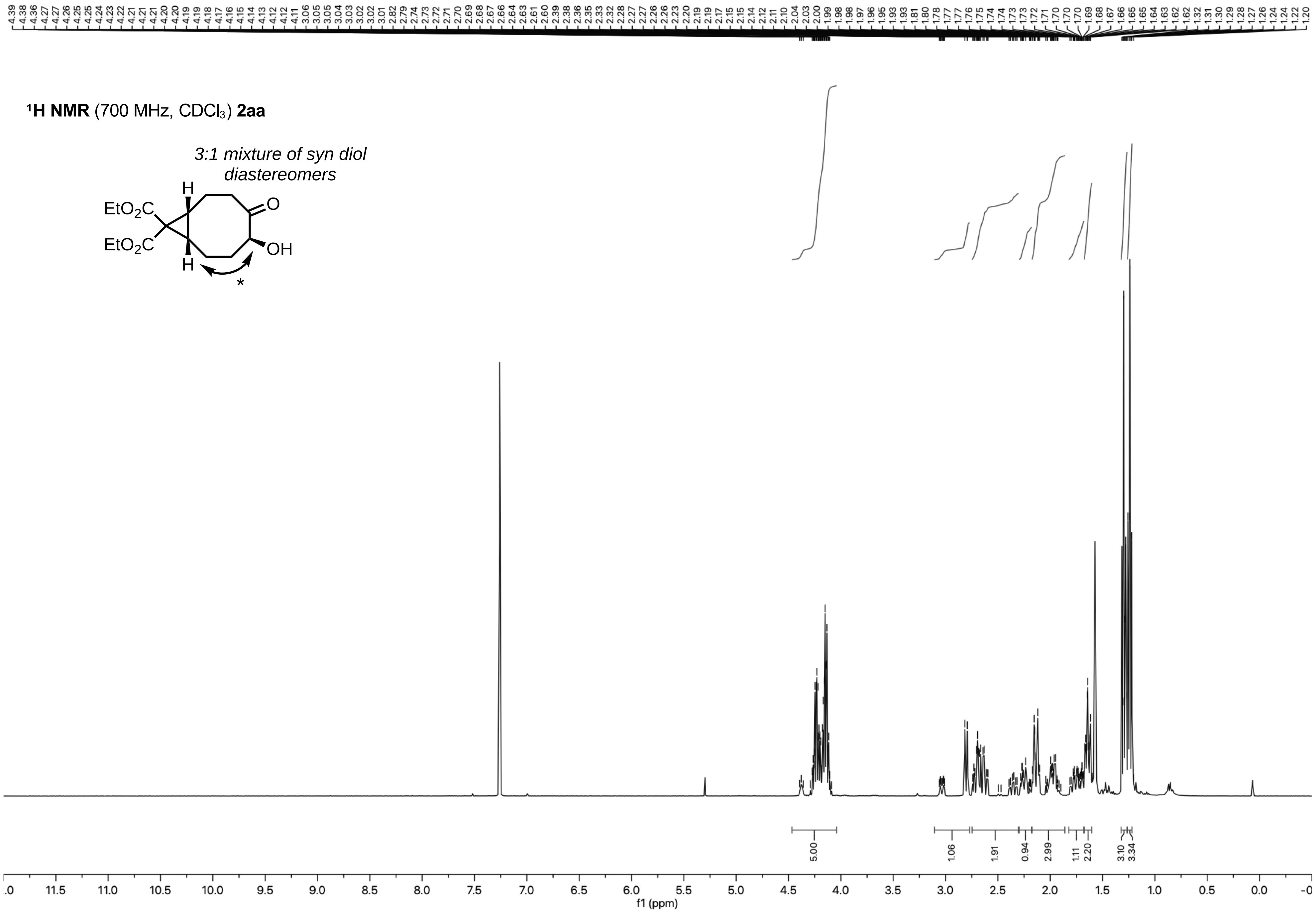

¹H NMR (700 MHz, CDCl₃) 2aa
3:1 mixture of syn diol diastereomers
EtO2C
EtO2C
H
H
O
OH
*
5.00
1.06
1.91
0.94
2.99
1.11
2.20
3.10
3.34
f1 (ppm)

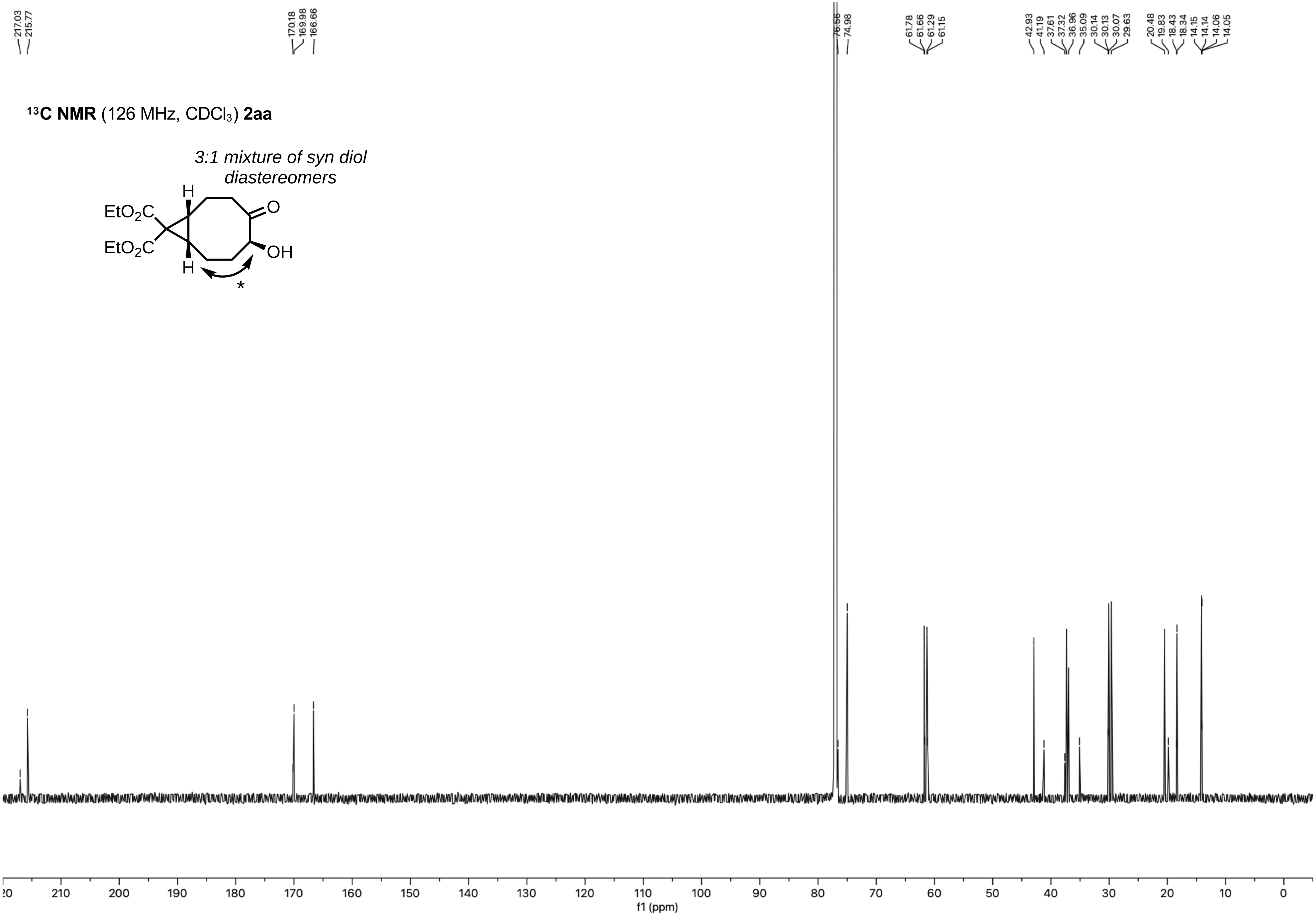

217.03
215.77
170.18
169.98
166.66
76.56
74.98
61.78
61.66
61.29
61.15
42.93
41.19
37.61
37.32
36.96
35.09
30.14
30.13
30.07
29.63
20.48
19.83
18.43
18.34
14.15
14.14
14.06
14.05
13C NMR (126 MHz, CDCl3) 2aa
3:1 mixture of syn diol diastereomers
H
EtO2C
EtO2C
H
O
OH
*
20
210
200
190
180
170
160
150
140
130
120
110
100
90
80
70
60
50
40
30
20
10
0
f1 (ppm)

¹H NMR (700 MHz, $CDCl_3$) **2ab**

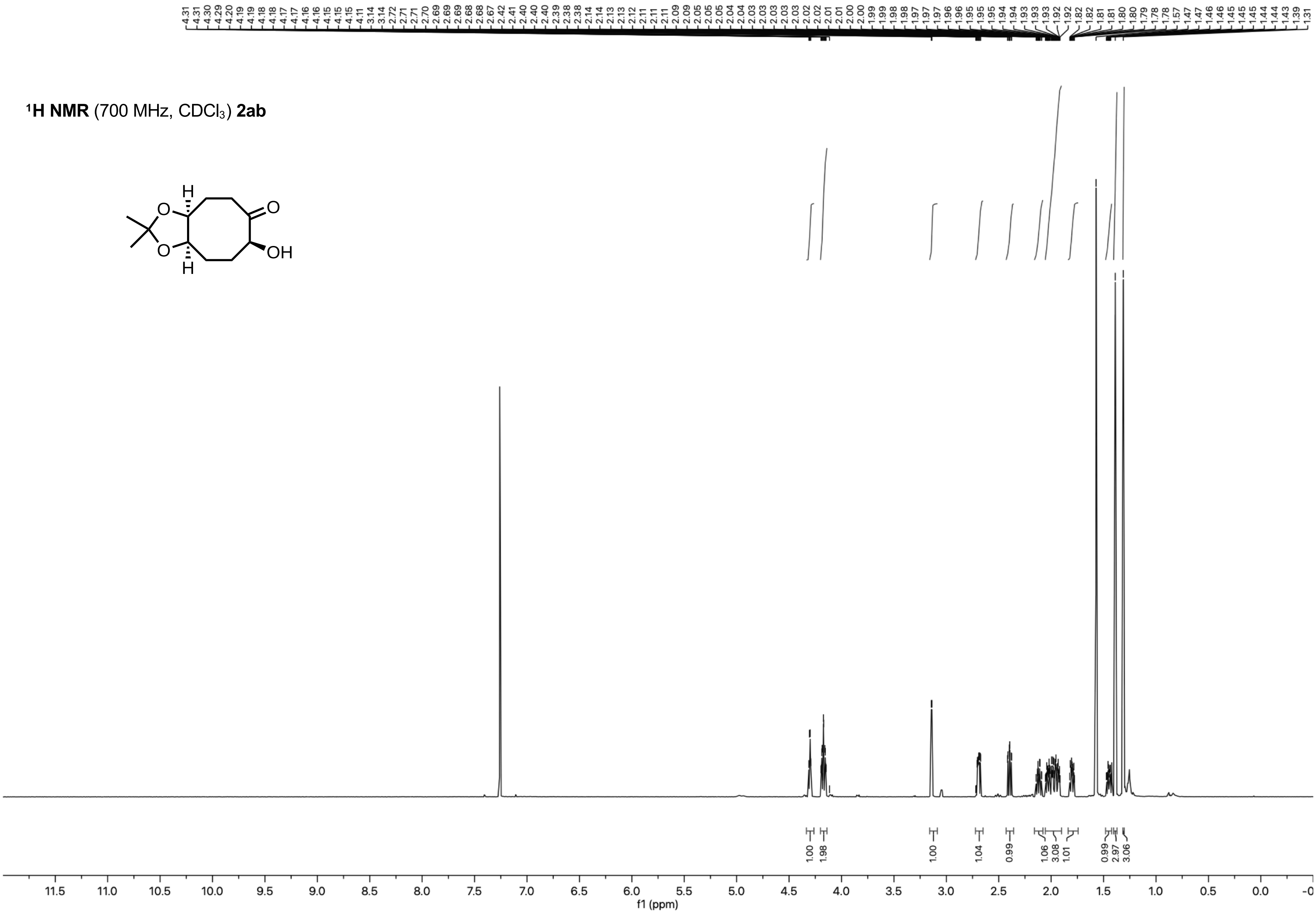

^{13}C NMR (126 MHz, $CDCl_3$) **2ab**

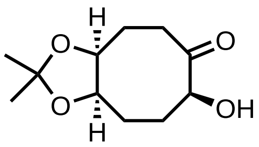

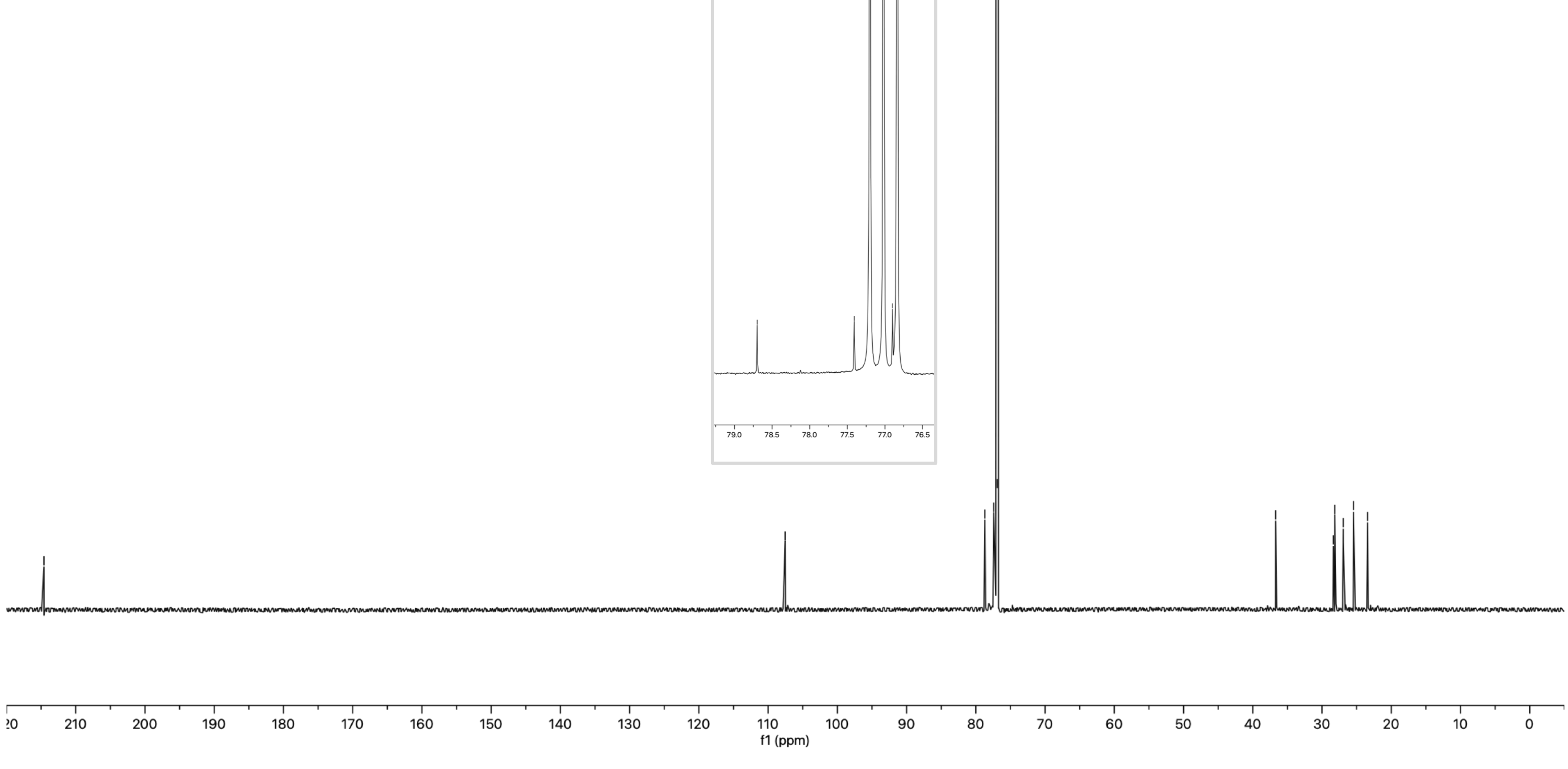

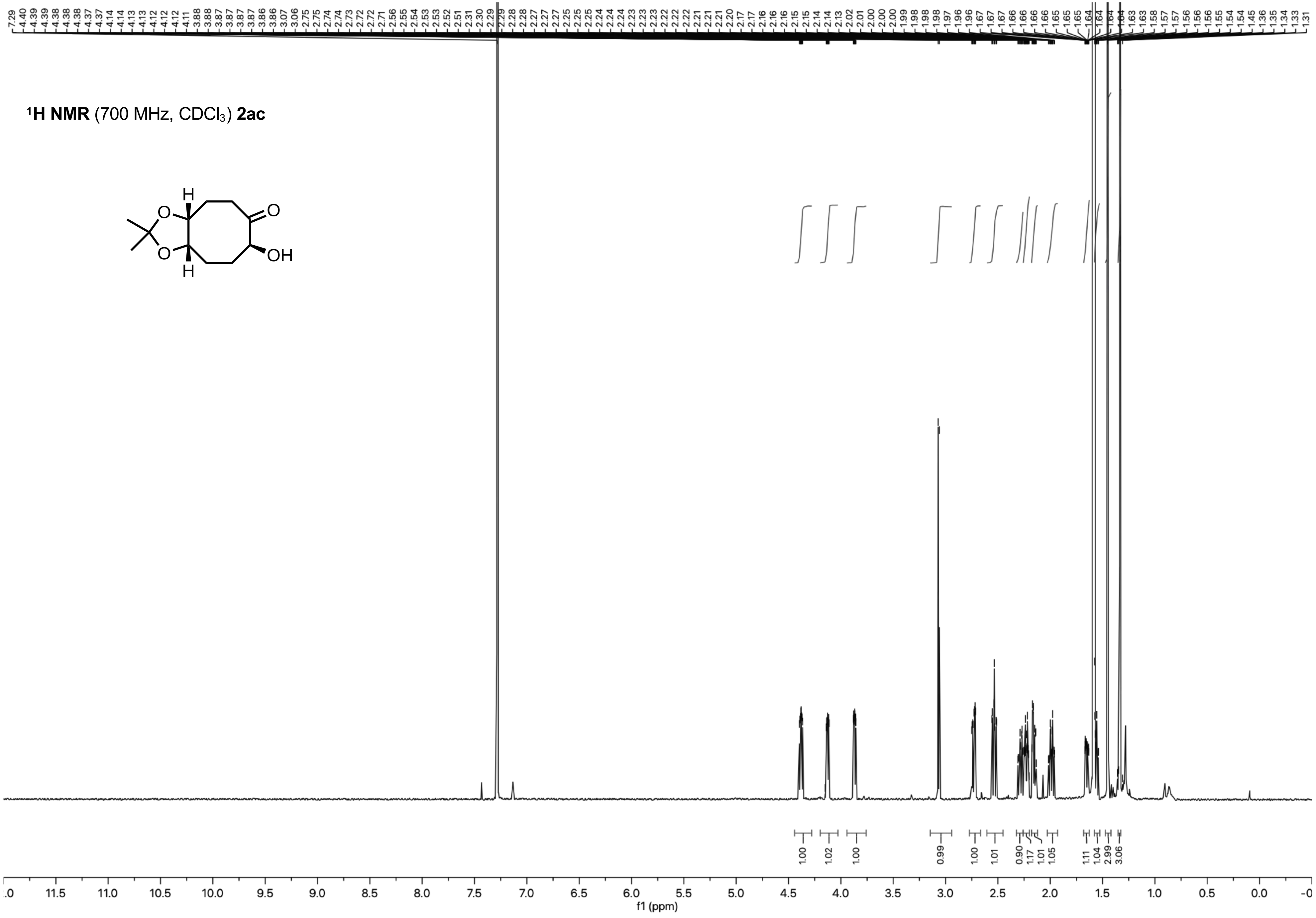

¹H NMR (700 MHz, CDCl₃) 2ac
f1 (ppm)

^{13}C NMR (126 MHz, $CDCl_3$) **2ac**

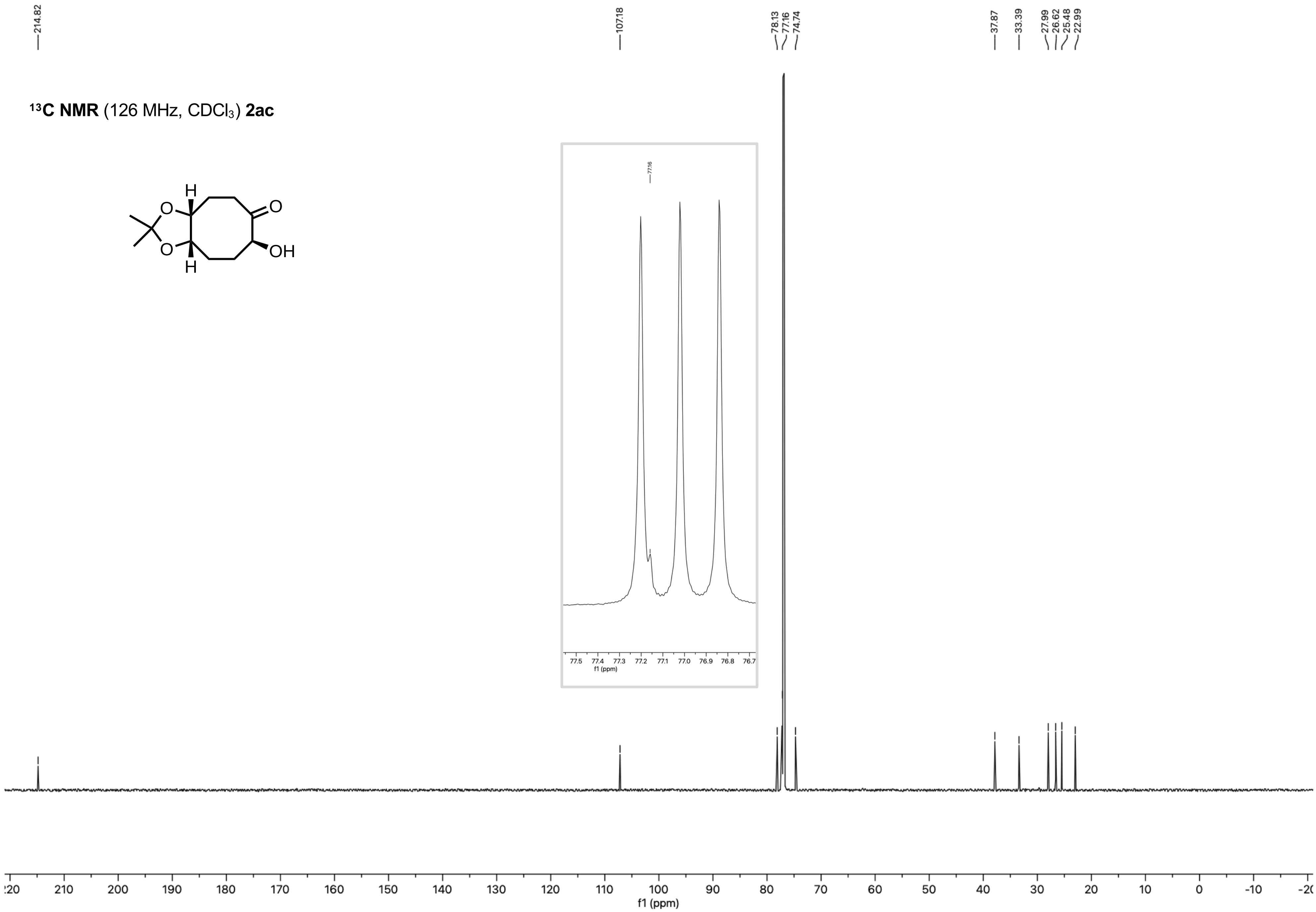

¹H NMR (700 MHz, $CDCl_3$) **4a**

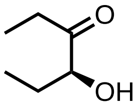

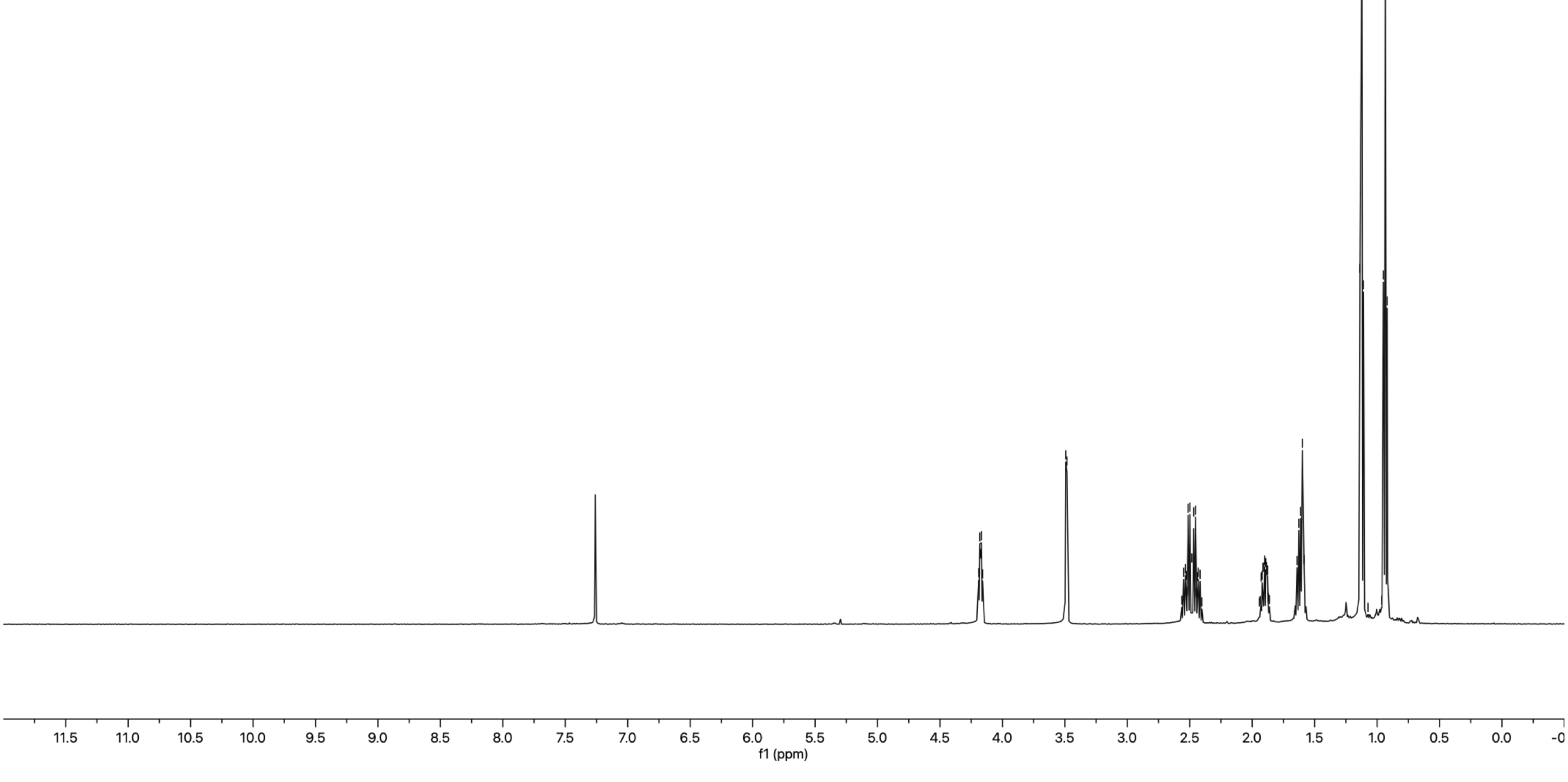

^{13}C NMR (176 MHz, $CDCl_3$) **4a**

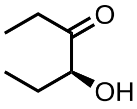

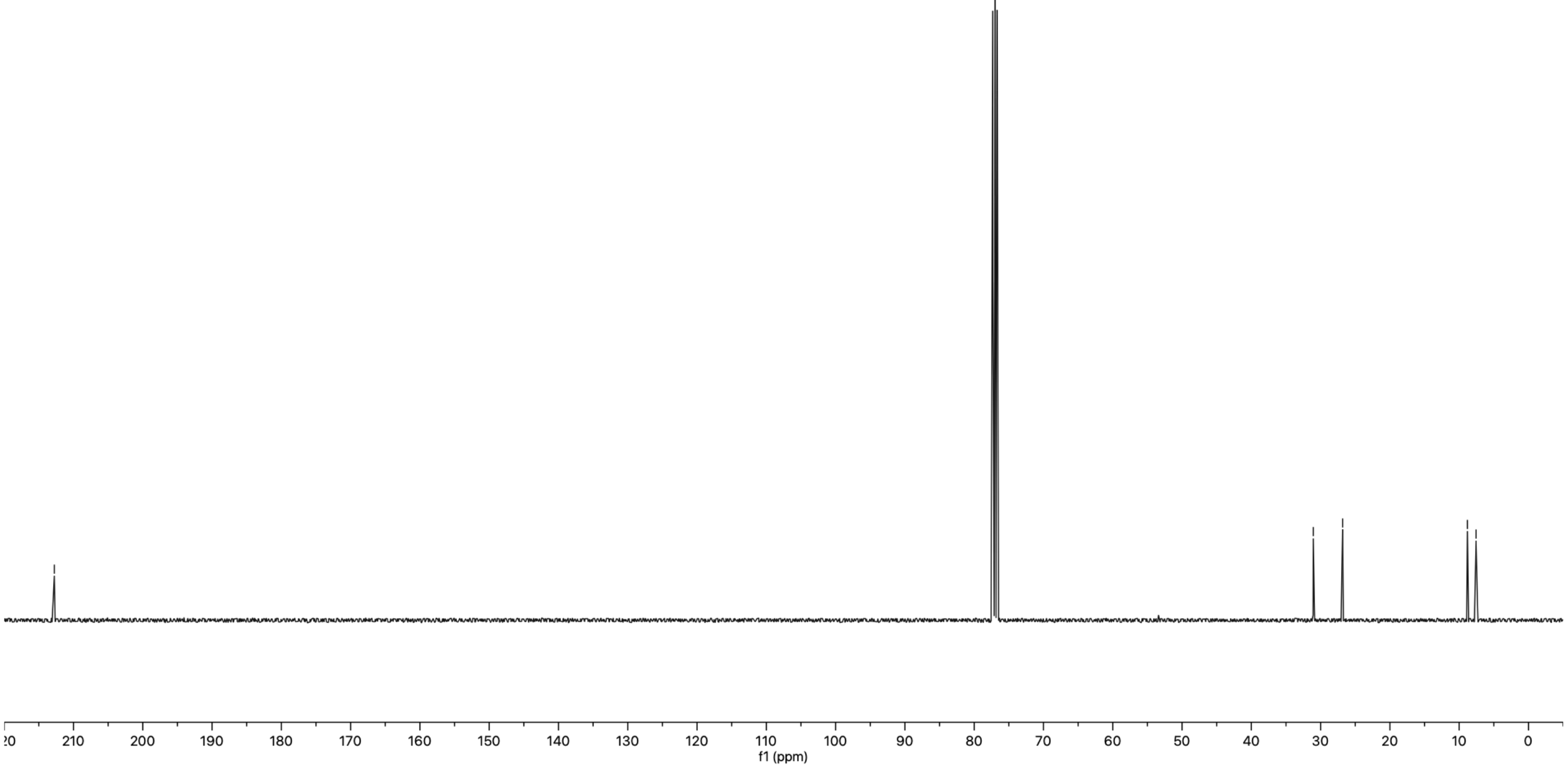

^{1}H NMR (700 MHz, $CDCl_3$) **4b**

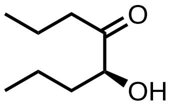

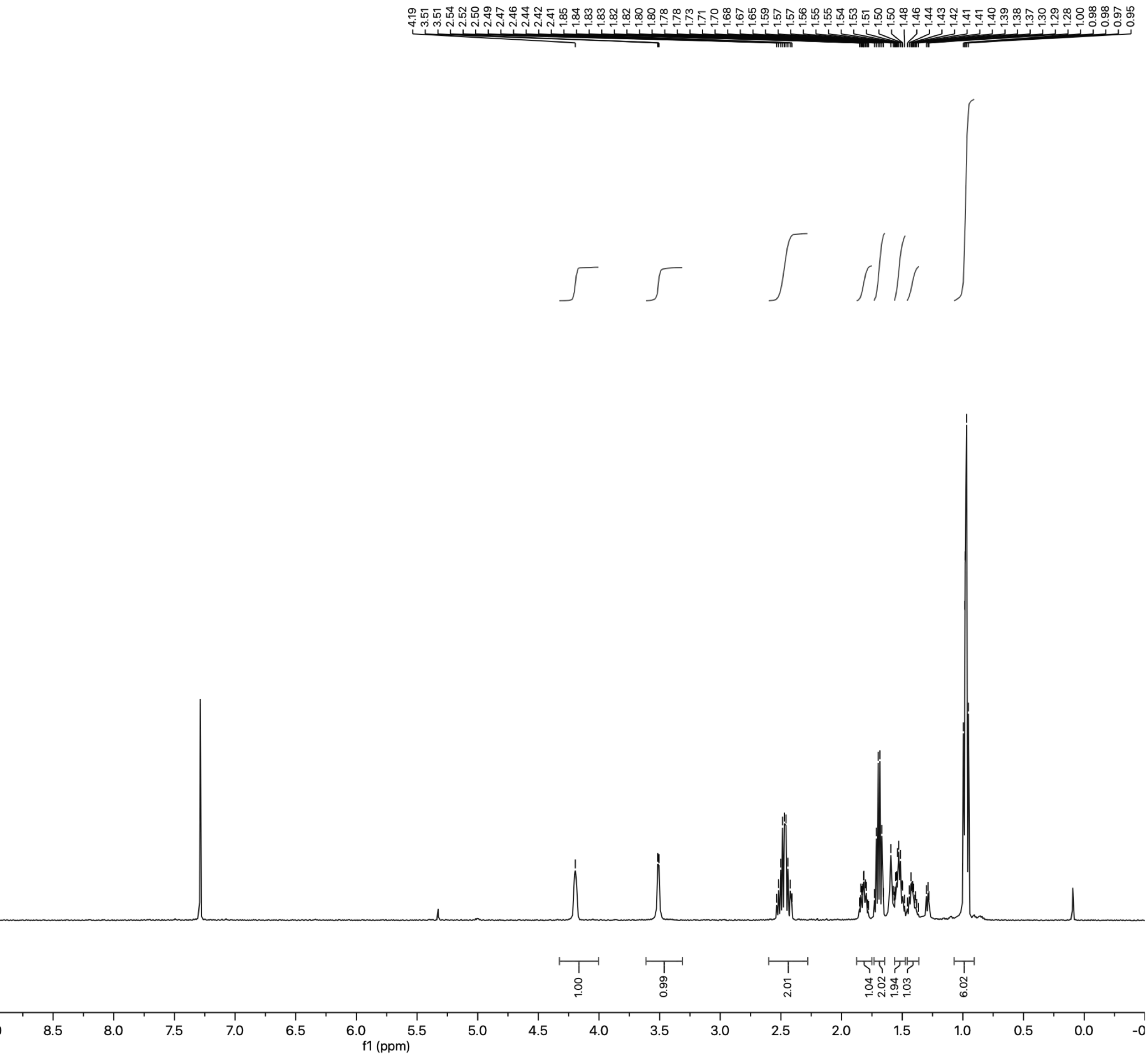

^{13}C NMR (176 MHz, $CDCl_3$) **4b**

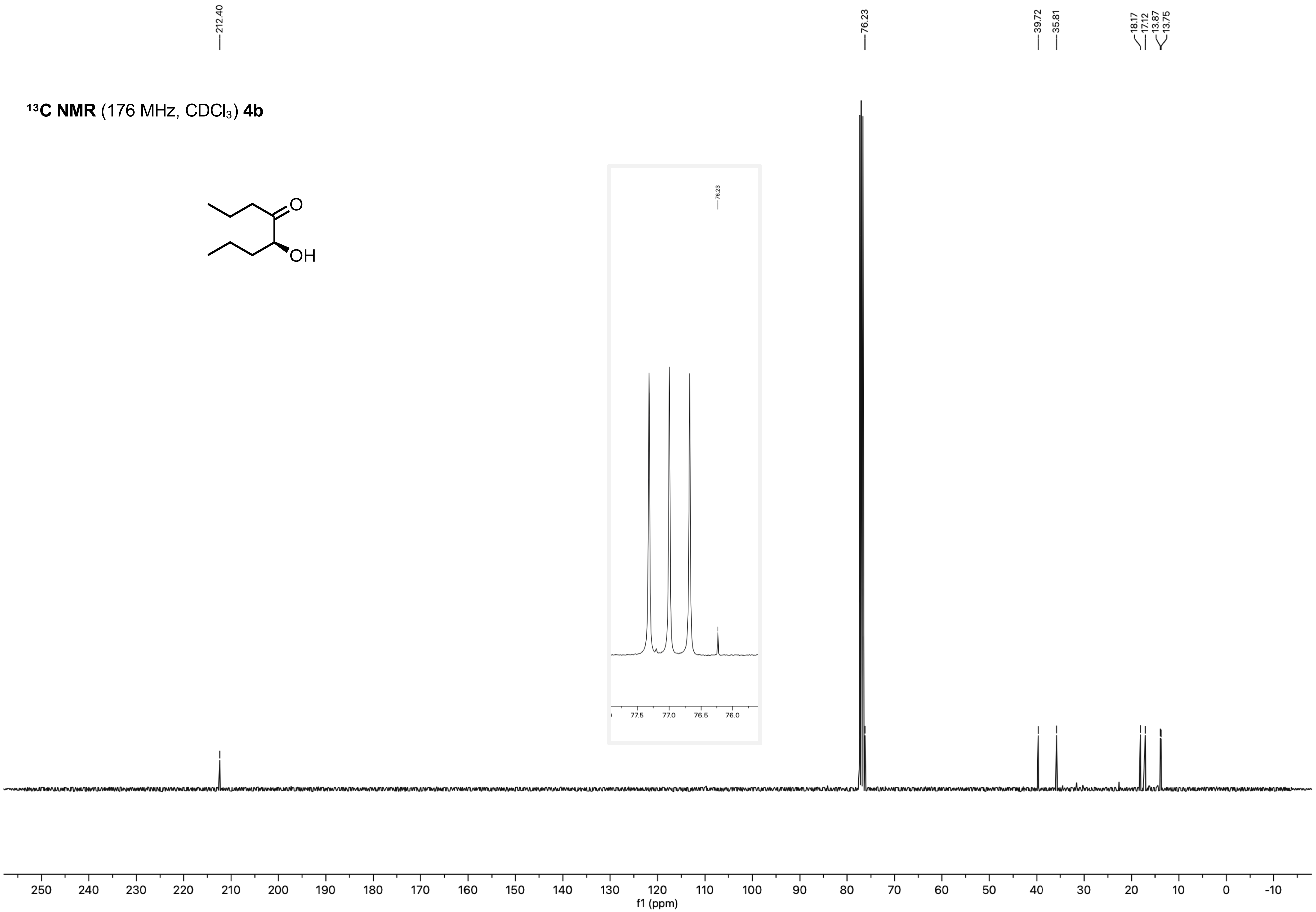

¹H NMR (700 MHz, $CDCl_3$) **4c**

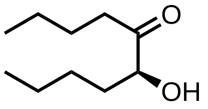

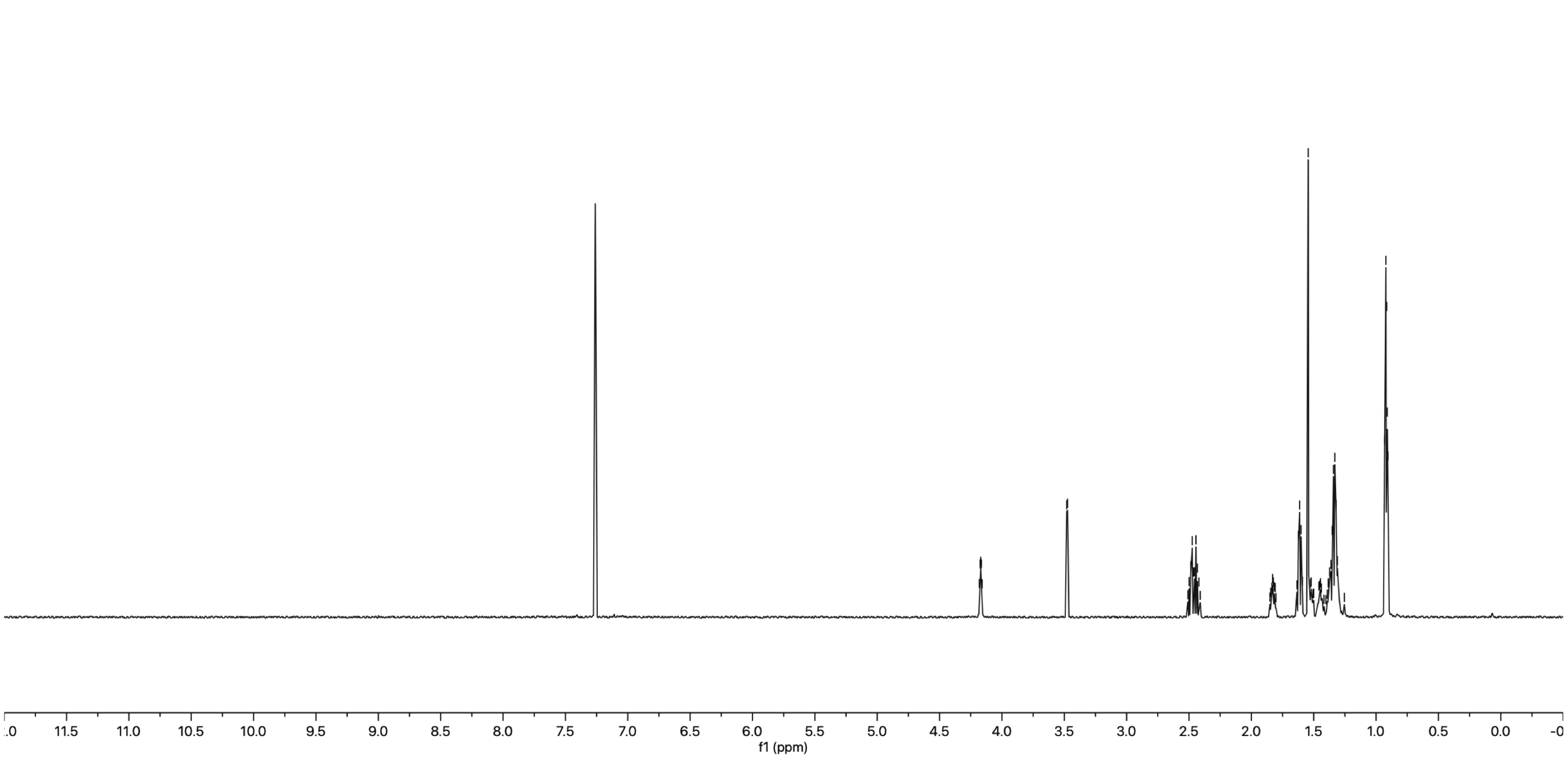

^{13}C NMR (176 MHz, $CDCl_3$) **4c**

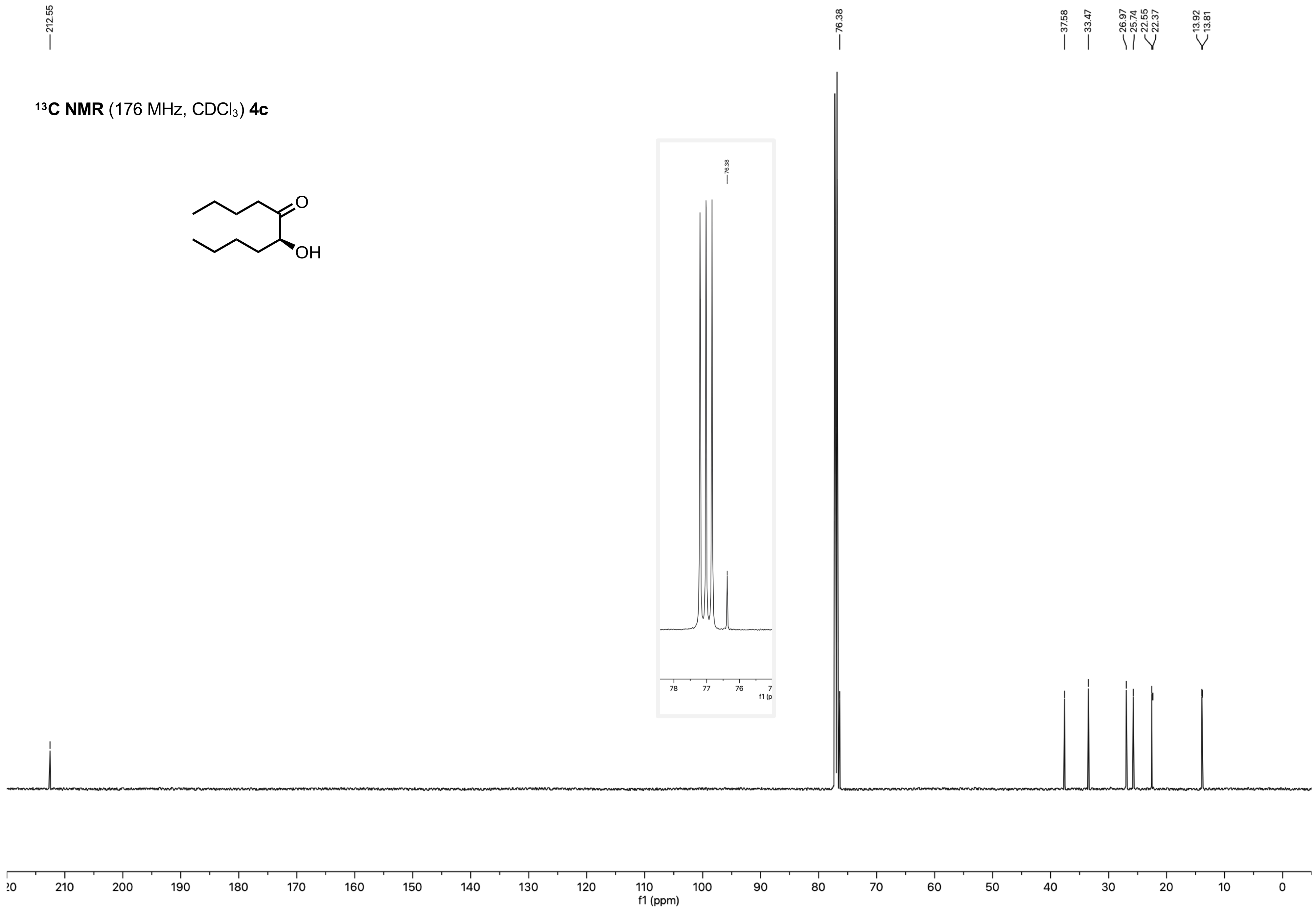

¹H NMR (700 MHz, $CDCl_3$) **4d**

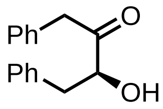

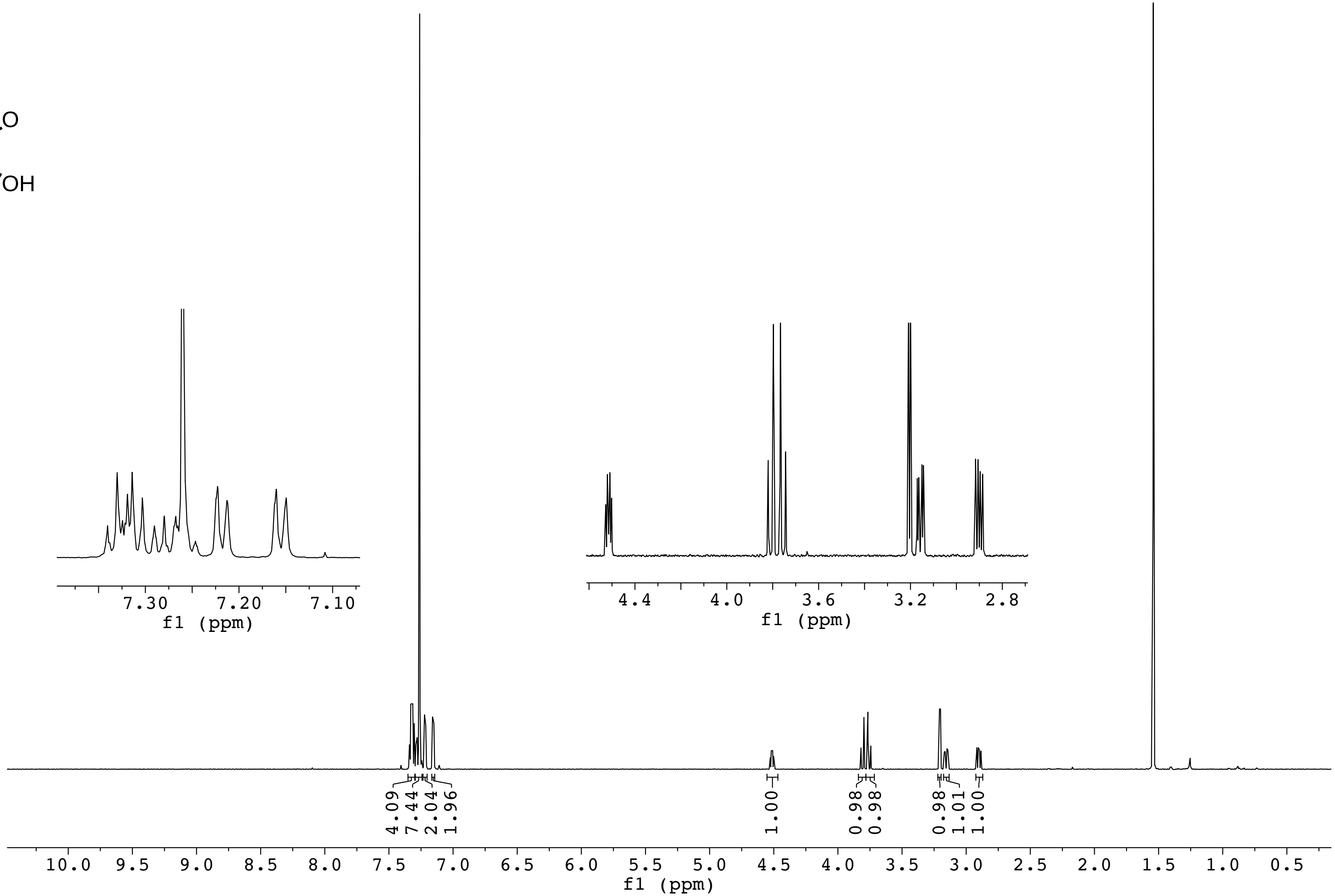

^{13}C NMR (176 MHz, $CDCl_3$) **4d**

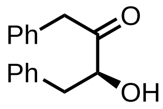

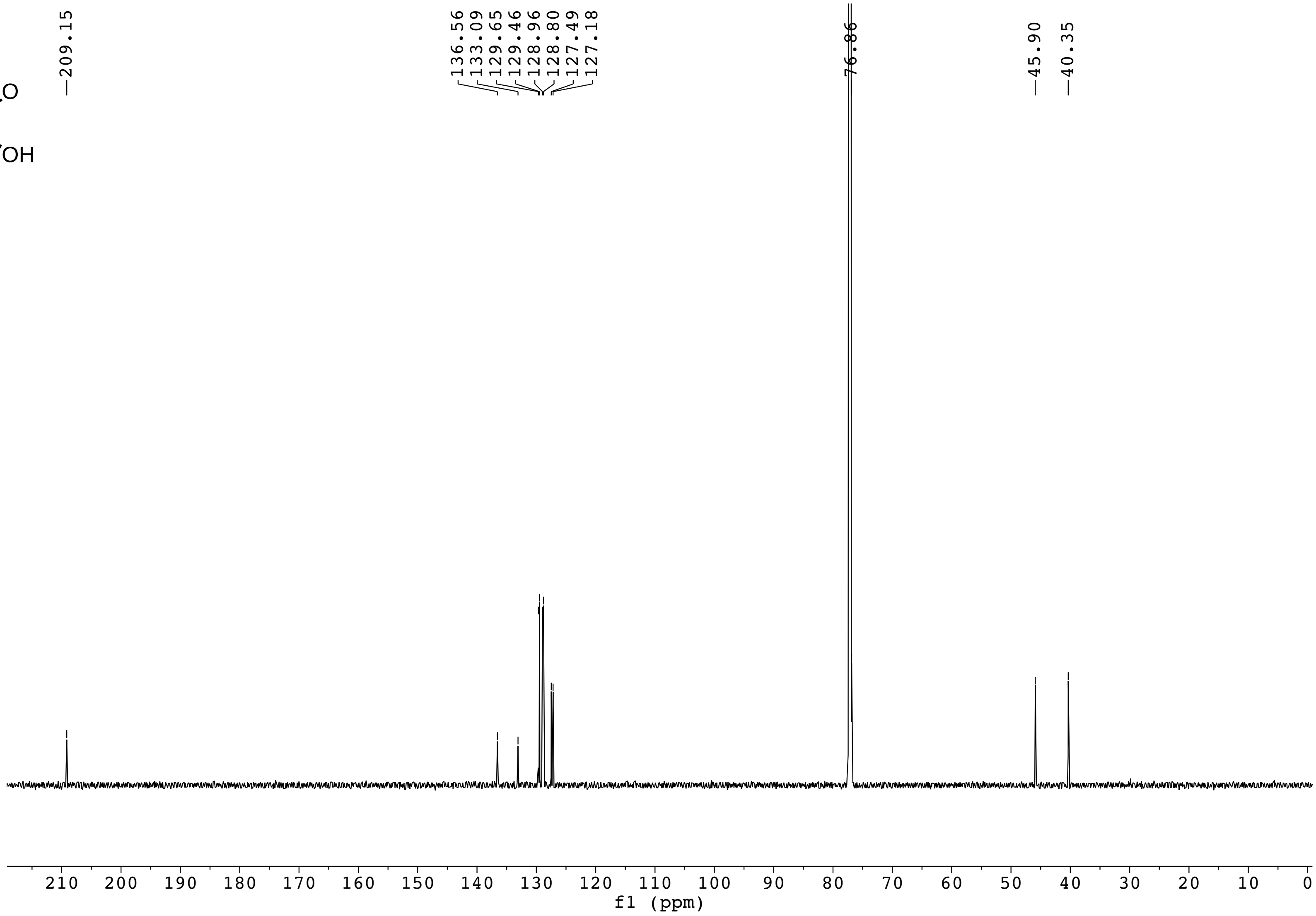

¹H NMR (700 MHz, $CDCl_3$) **4e**

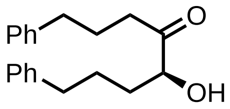

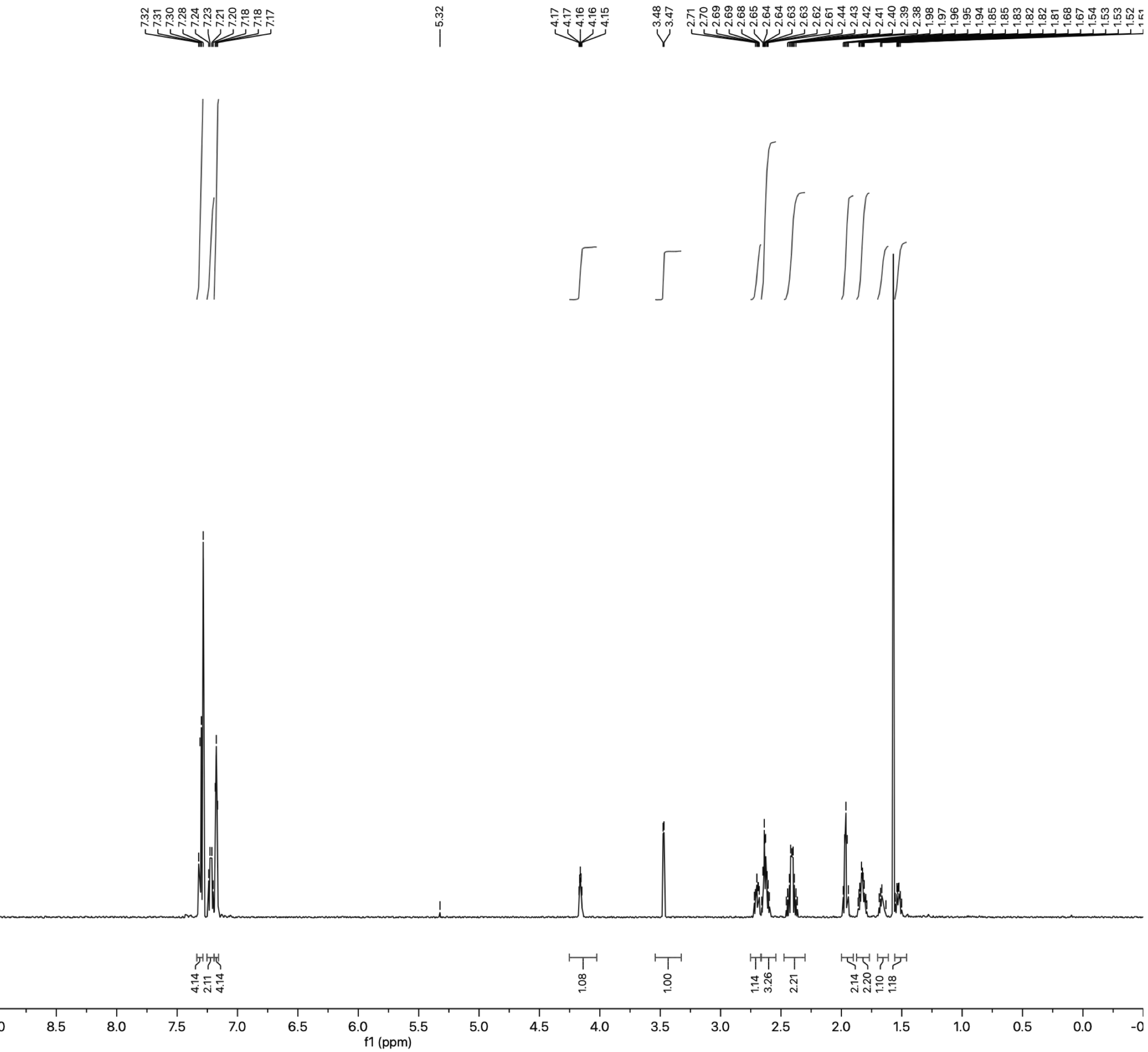

^{13}C NMR (176 MHz, $CDCl_3$) **4e**

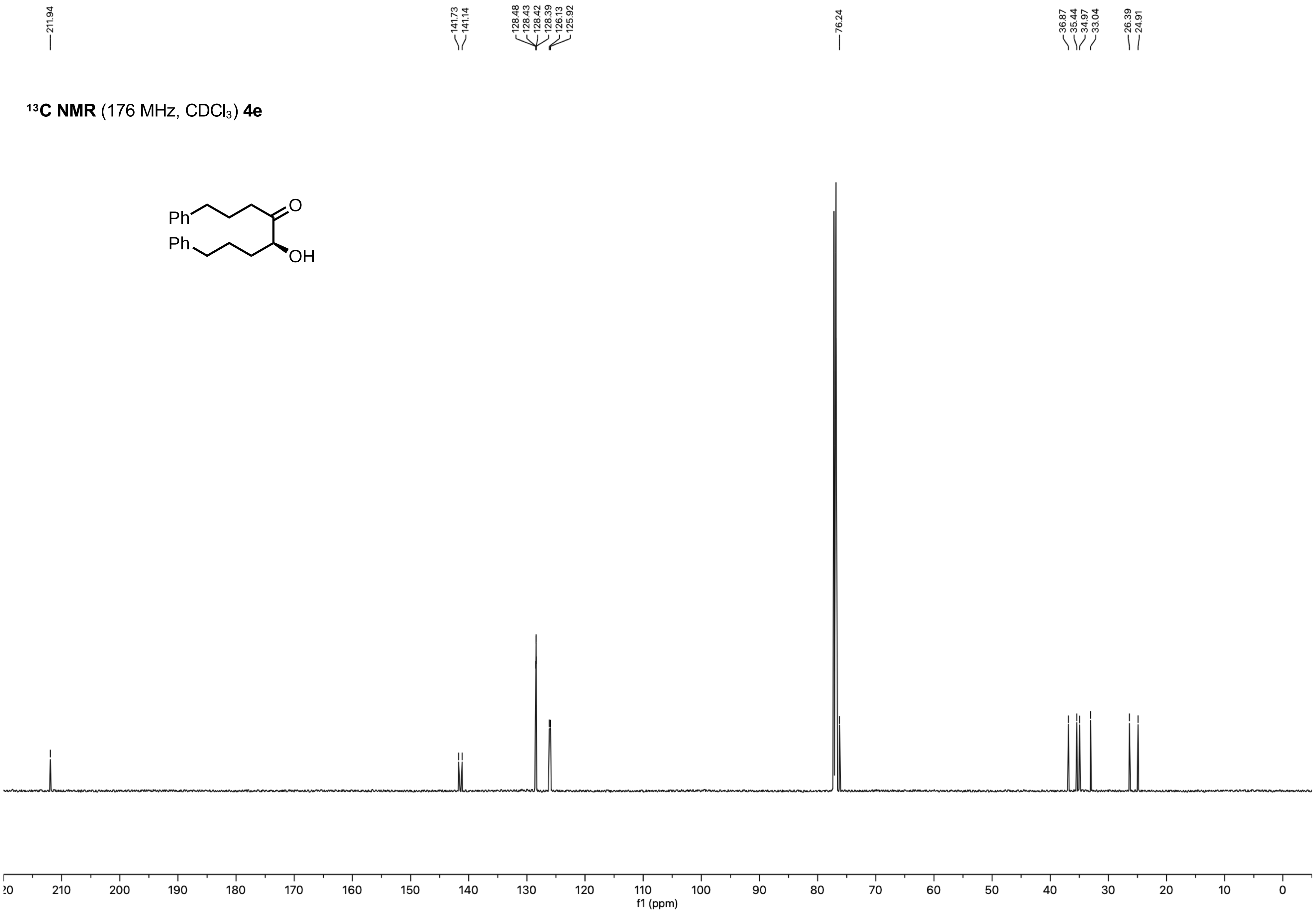

[1]**H NMR** (700 MHz, $CDCl_3$) **4f**

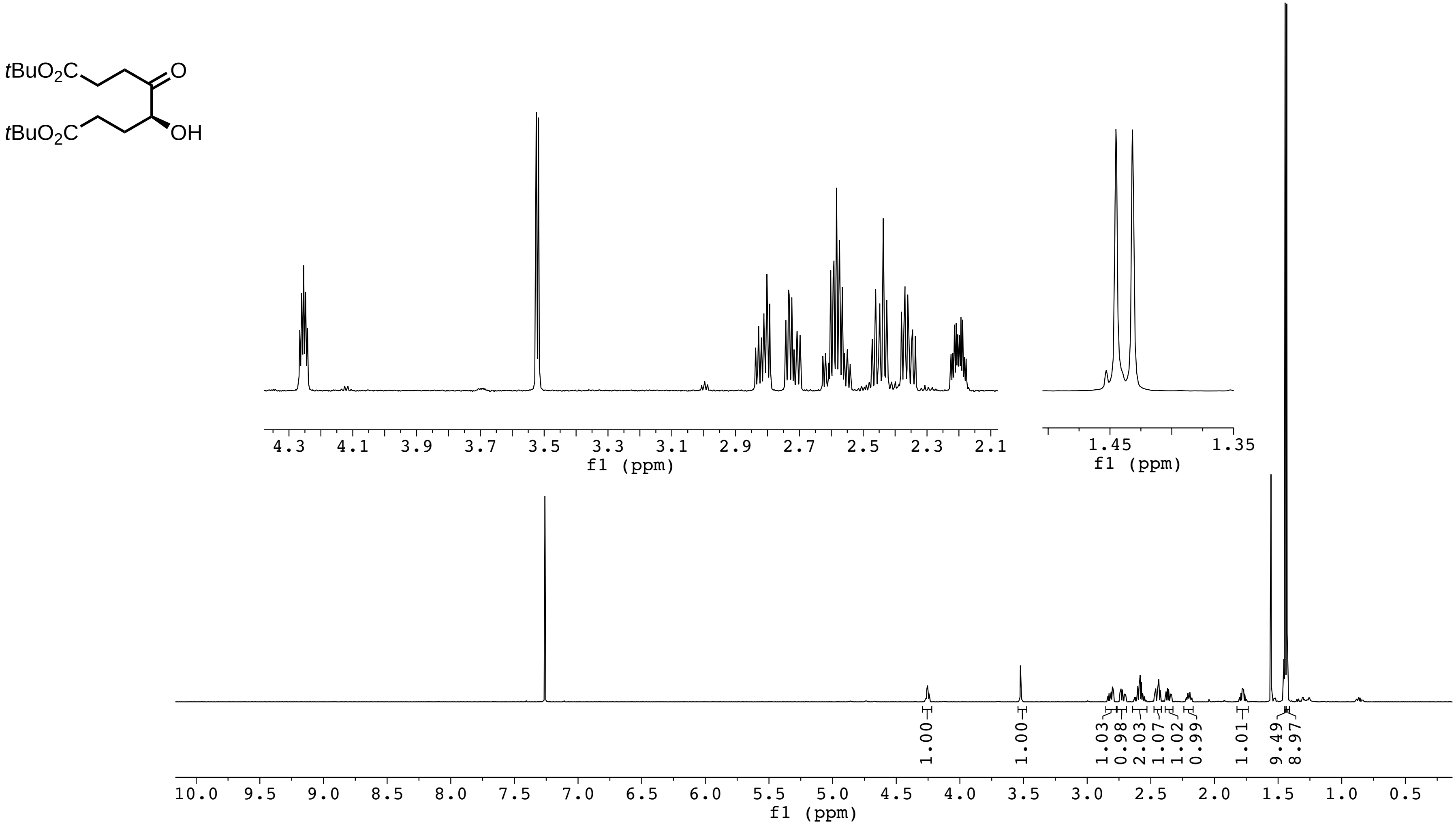

^{13}C NMR (176 MHz, $CDCl_3$) **4f**

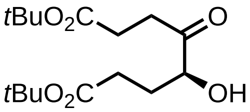

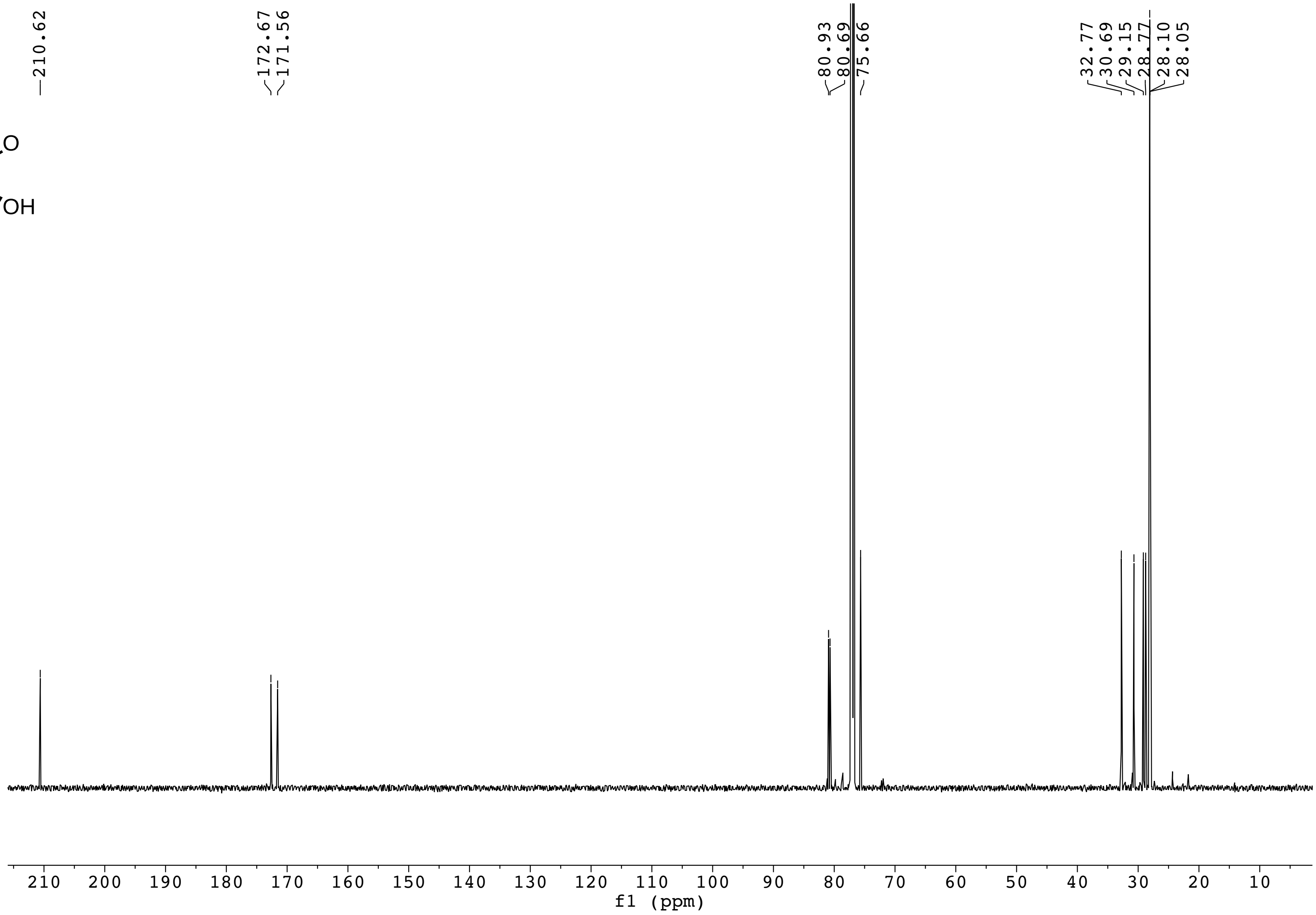

¹H NMR (700 MHz, $CDCl_3$) **4g**

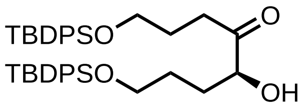

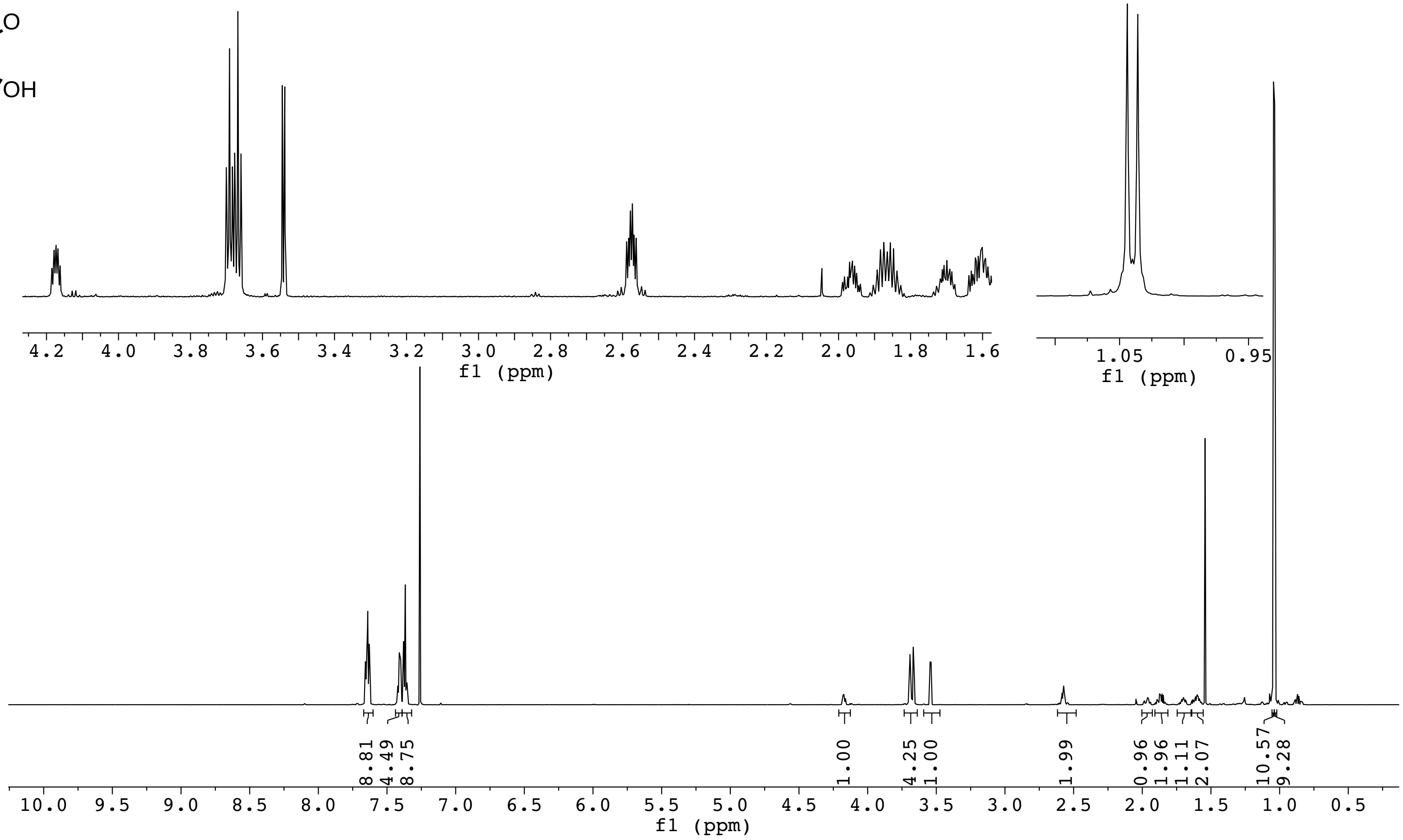

^{13}C NMR (176 MHz, $CDCl_3$) **4g**

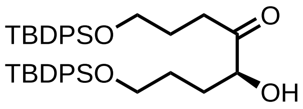

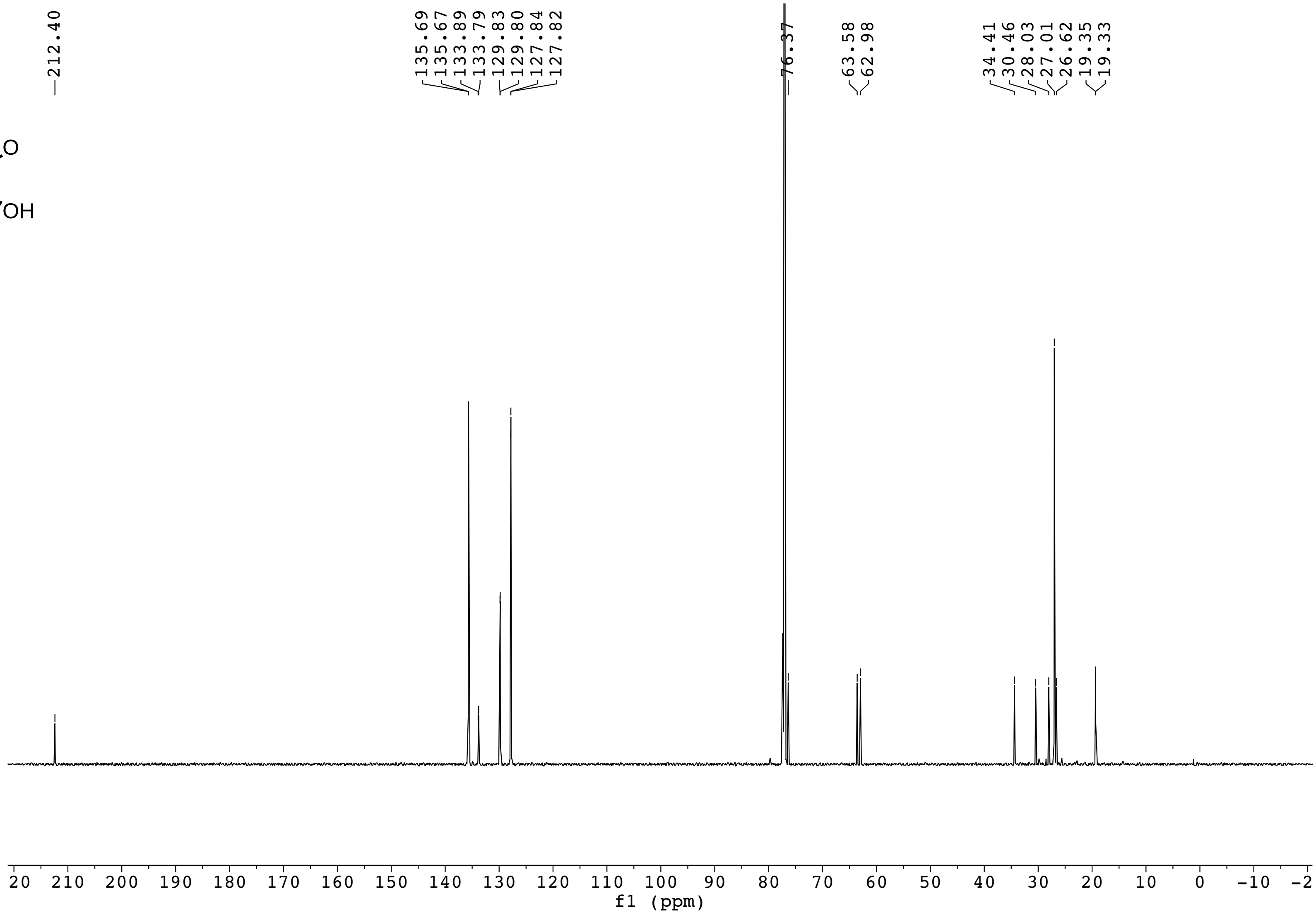

^{1}H NMR (700 MHz, $CDCl_3$) **4h**

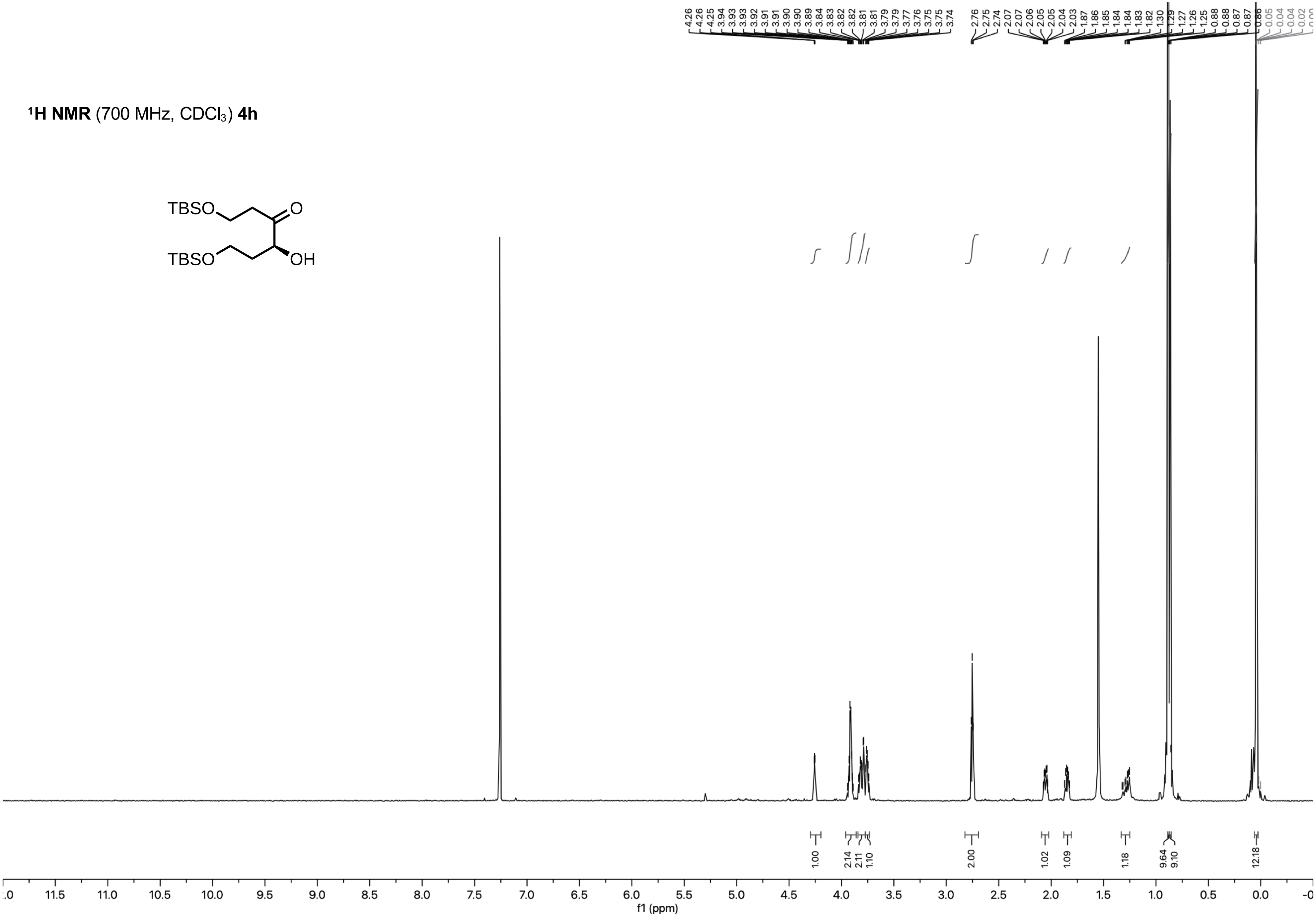

^{13}C NMR (176 MHz, $CDCl_3$) **4h**

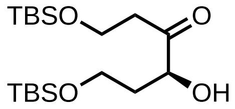

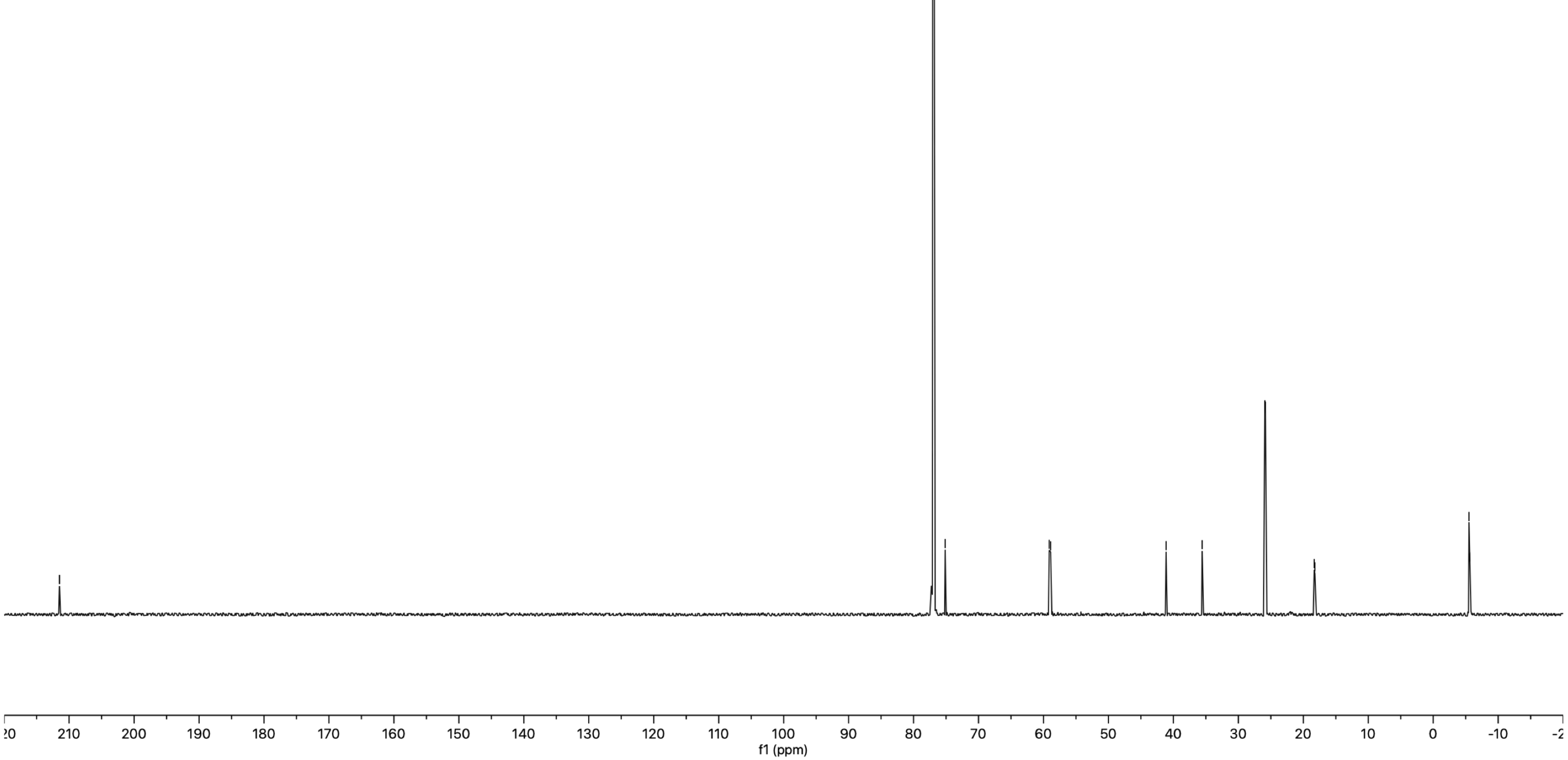

¹H NMR (700 MHz, $CDCl_3$) **4i**

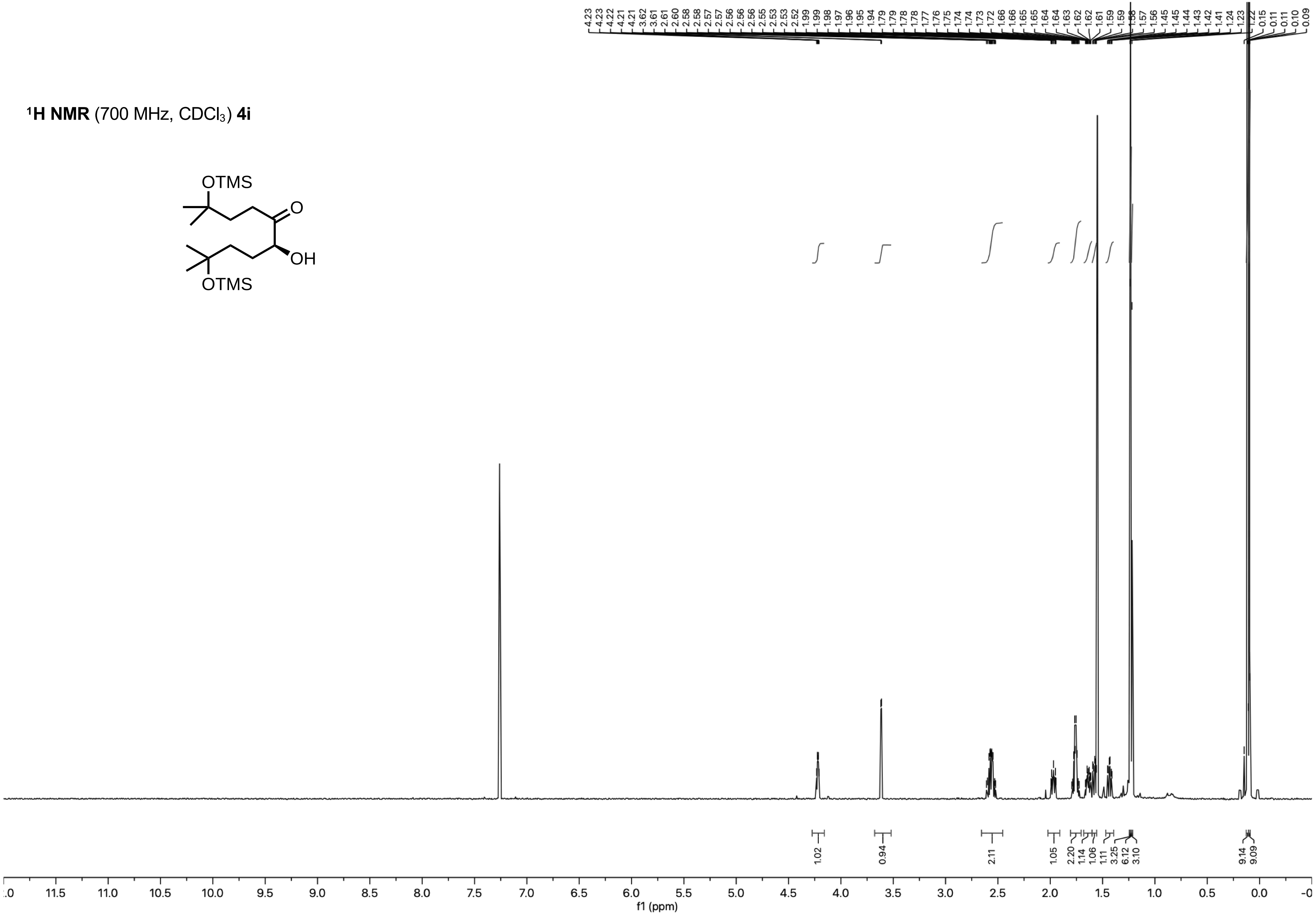

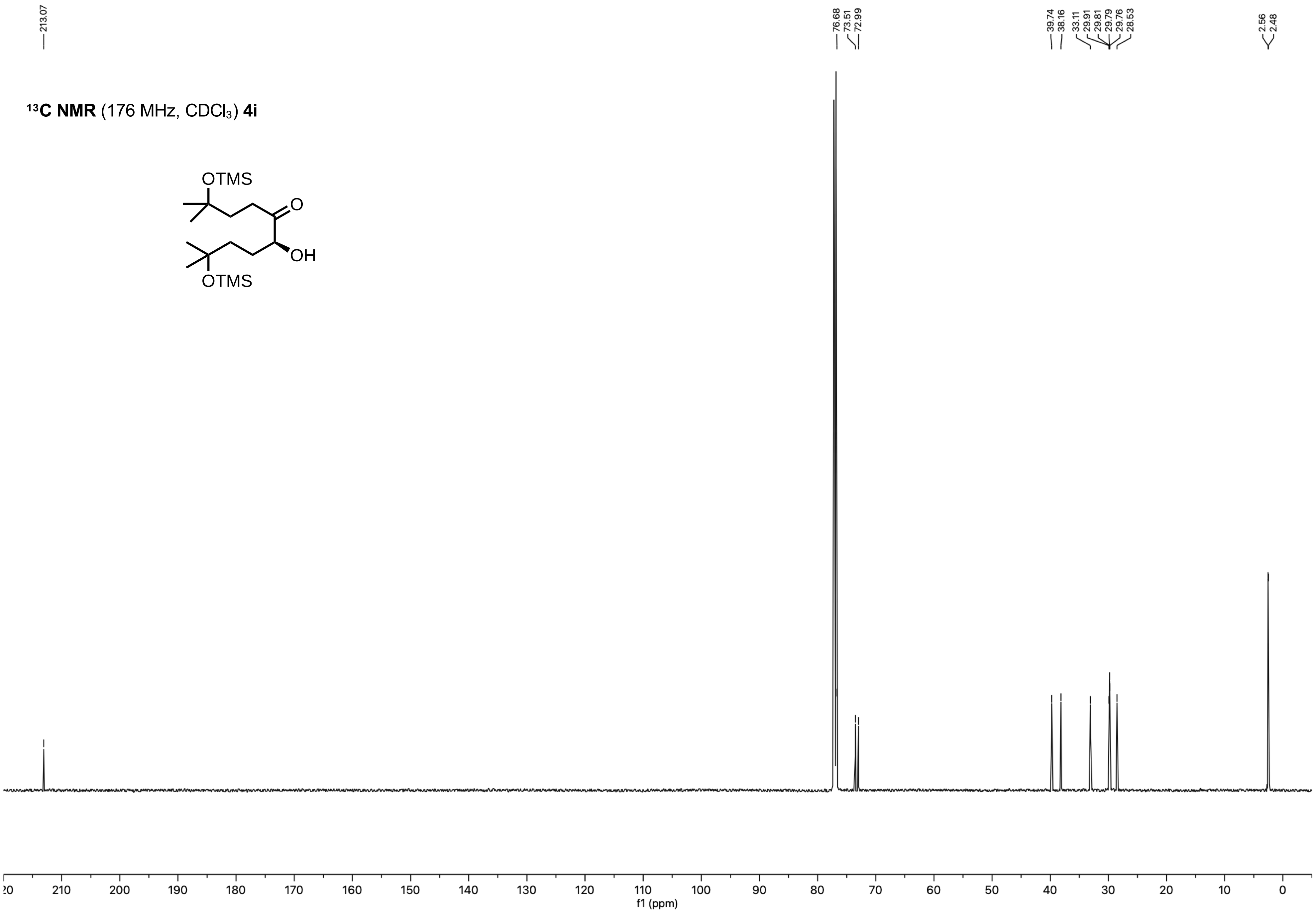

13C NMR (176 MHz, CDCl3) 4i
OTMS
O
OH
OTMS
213.07
76.68
73.51
72.99
39.74
38.16
33.11
29.91
29.81
29.79
29.76
28.53
2.56
2.48
210
200
190
180
170
160
150
140
130
120
110
100
90
80
70
60
50
40
30
20
10
0
f1 (ppm)

¹H NMR (700 MHz, $CDCl_3$) **4k**

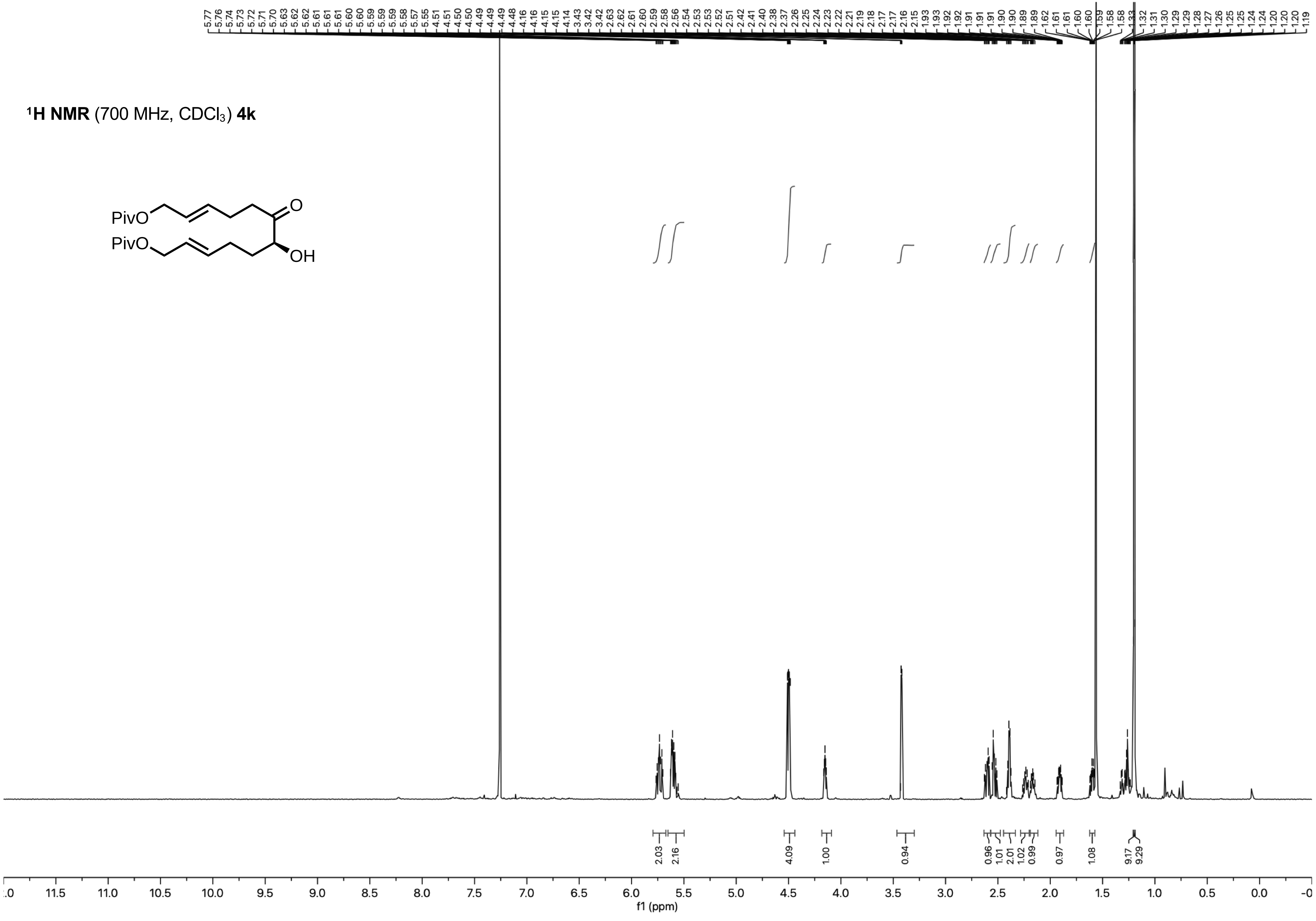

^{13}**C NMR** (176 MHz, $CDCl_3$) **4k**

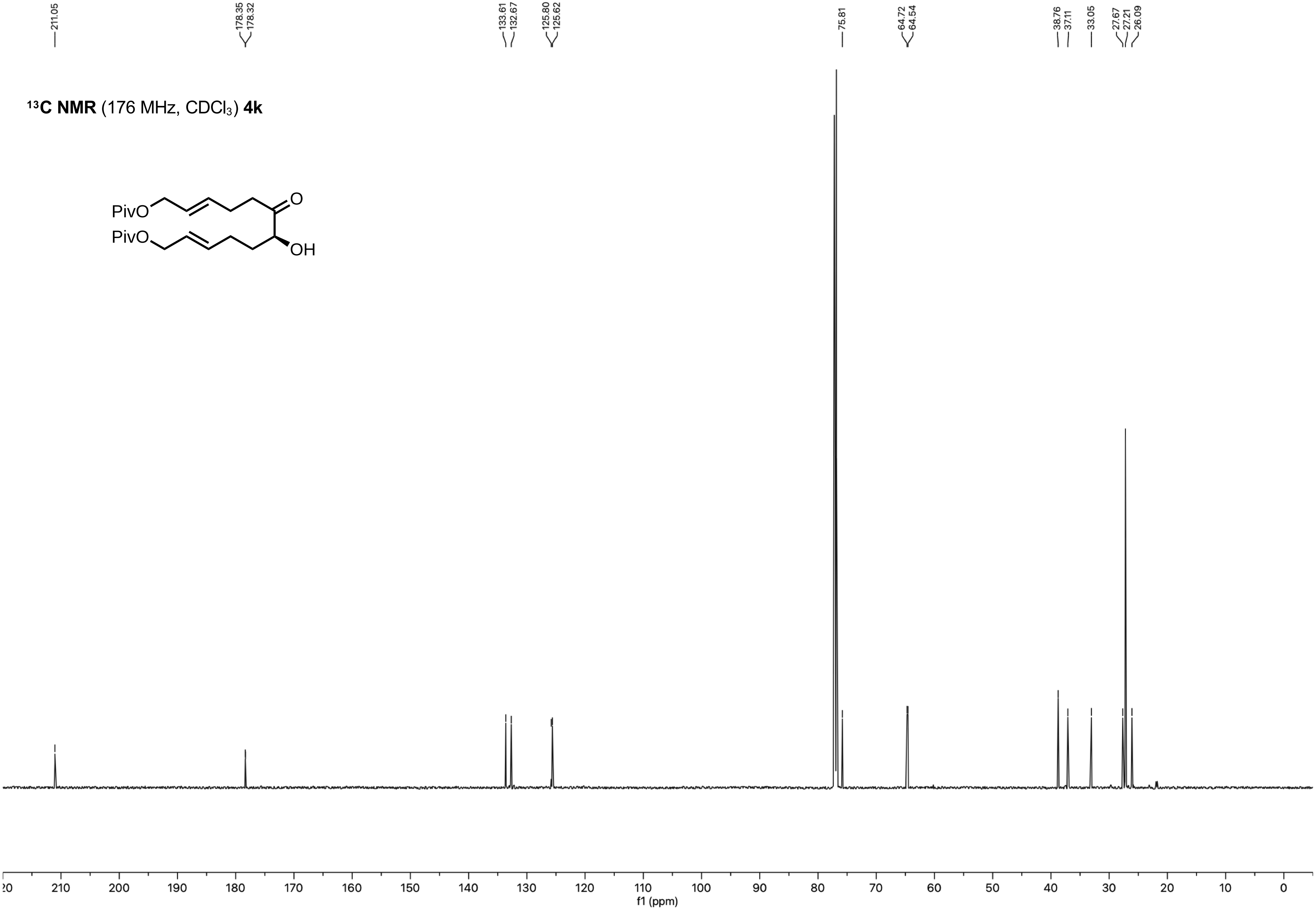

^{1}H NMR (700 MHz, $CDCl_3$) **4l**

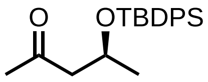

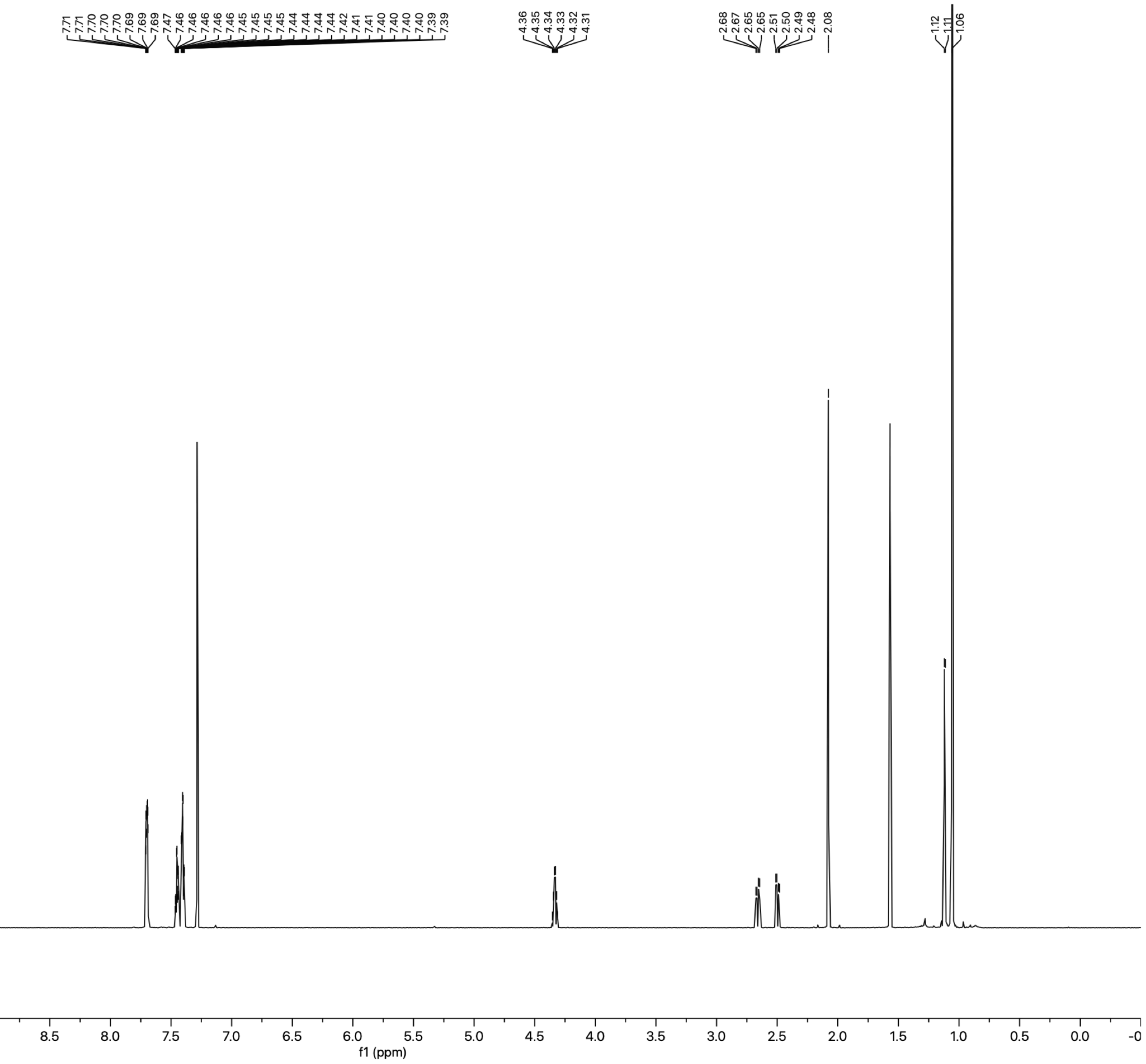

^{13}C NMR (176 MHz, $CDCl_3$) **4l**

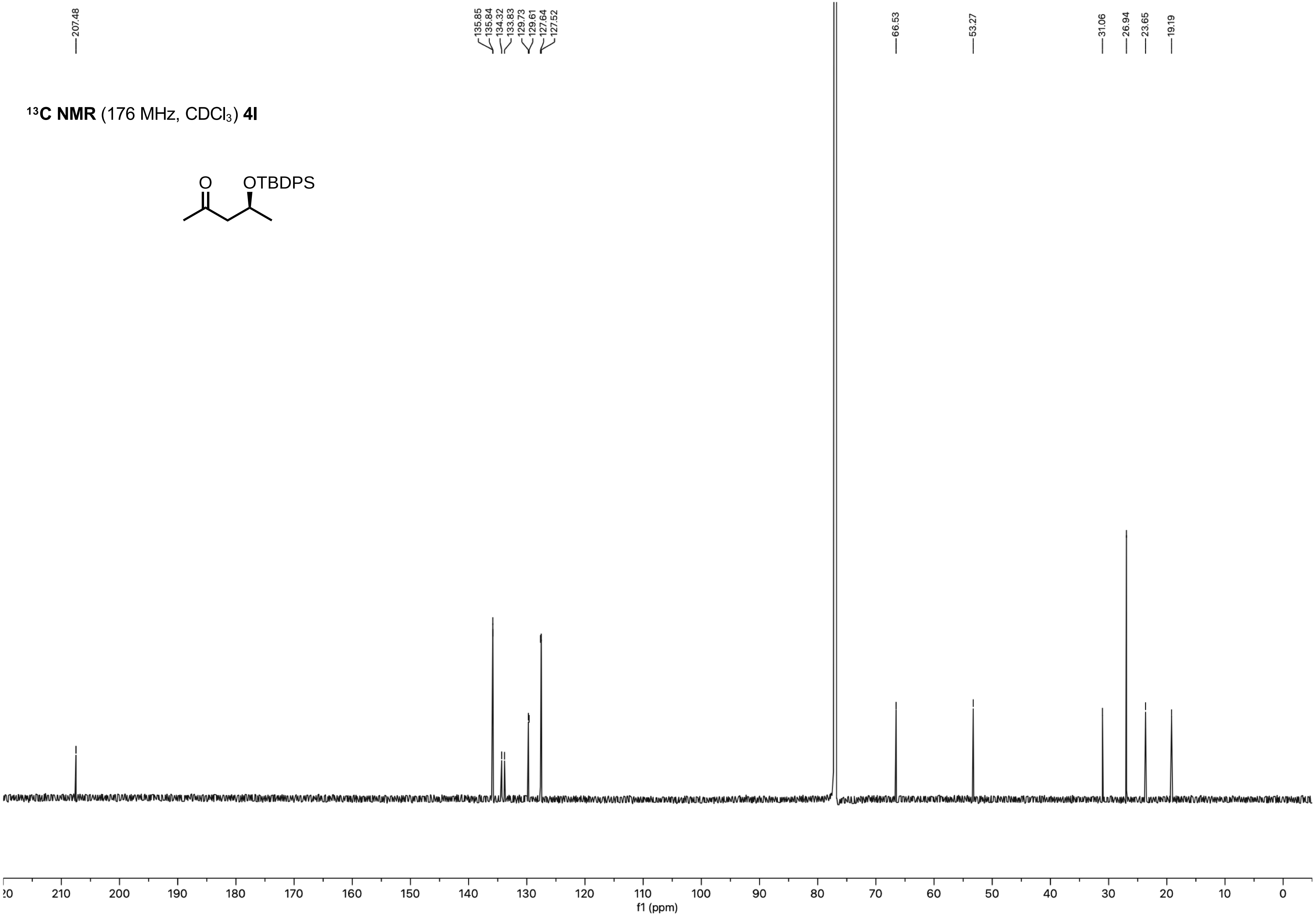

Spectroscopic data for benzoylated hydroxyketones

^{1}H NMR (700 MHz, $CDCl_3$) **Bz-2a**

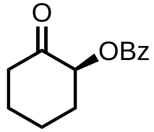

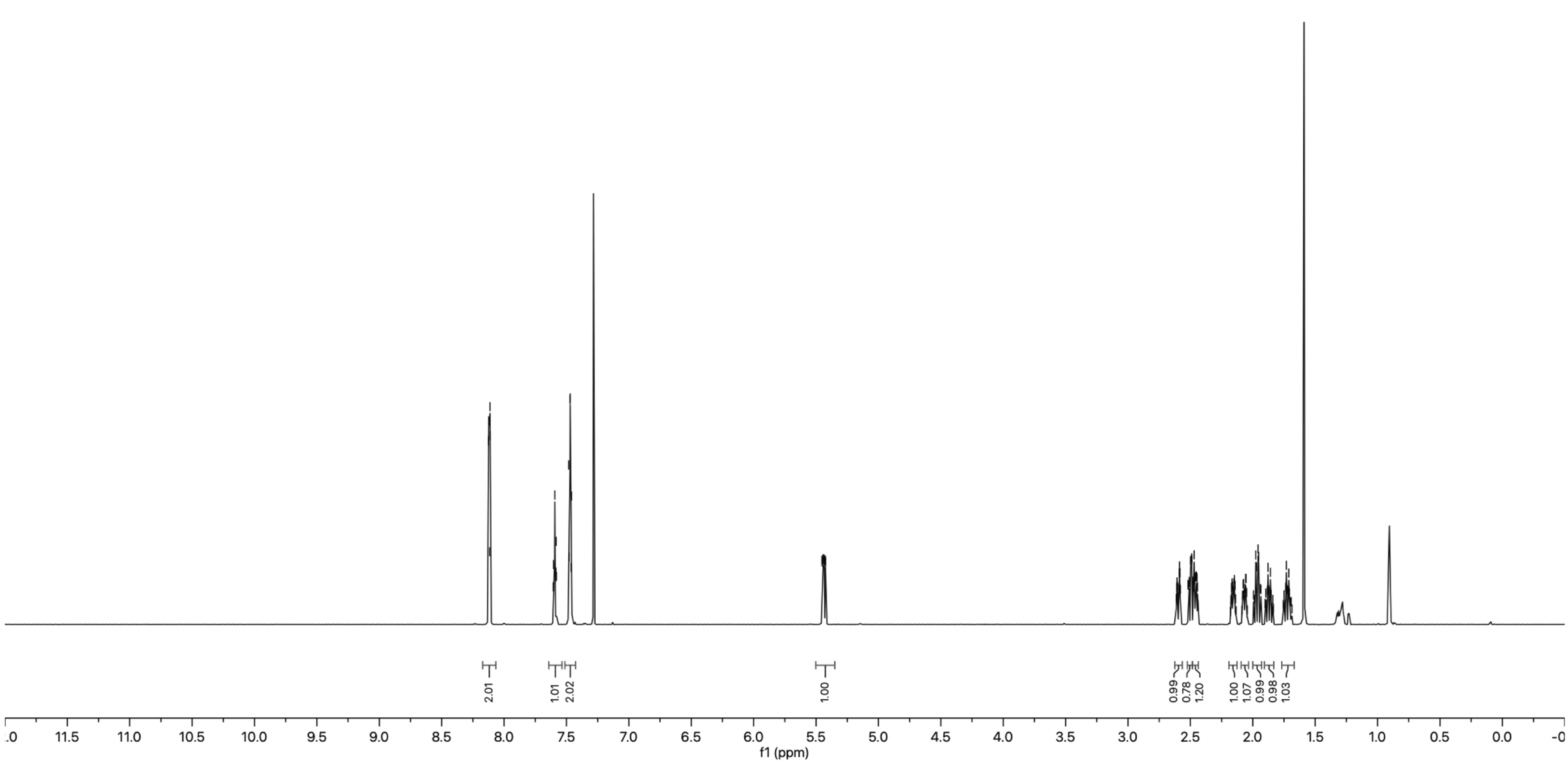

^{13}C NMR (126 MHz, $CDCl_3$) **Bz-2a**

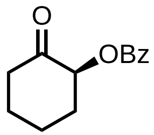

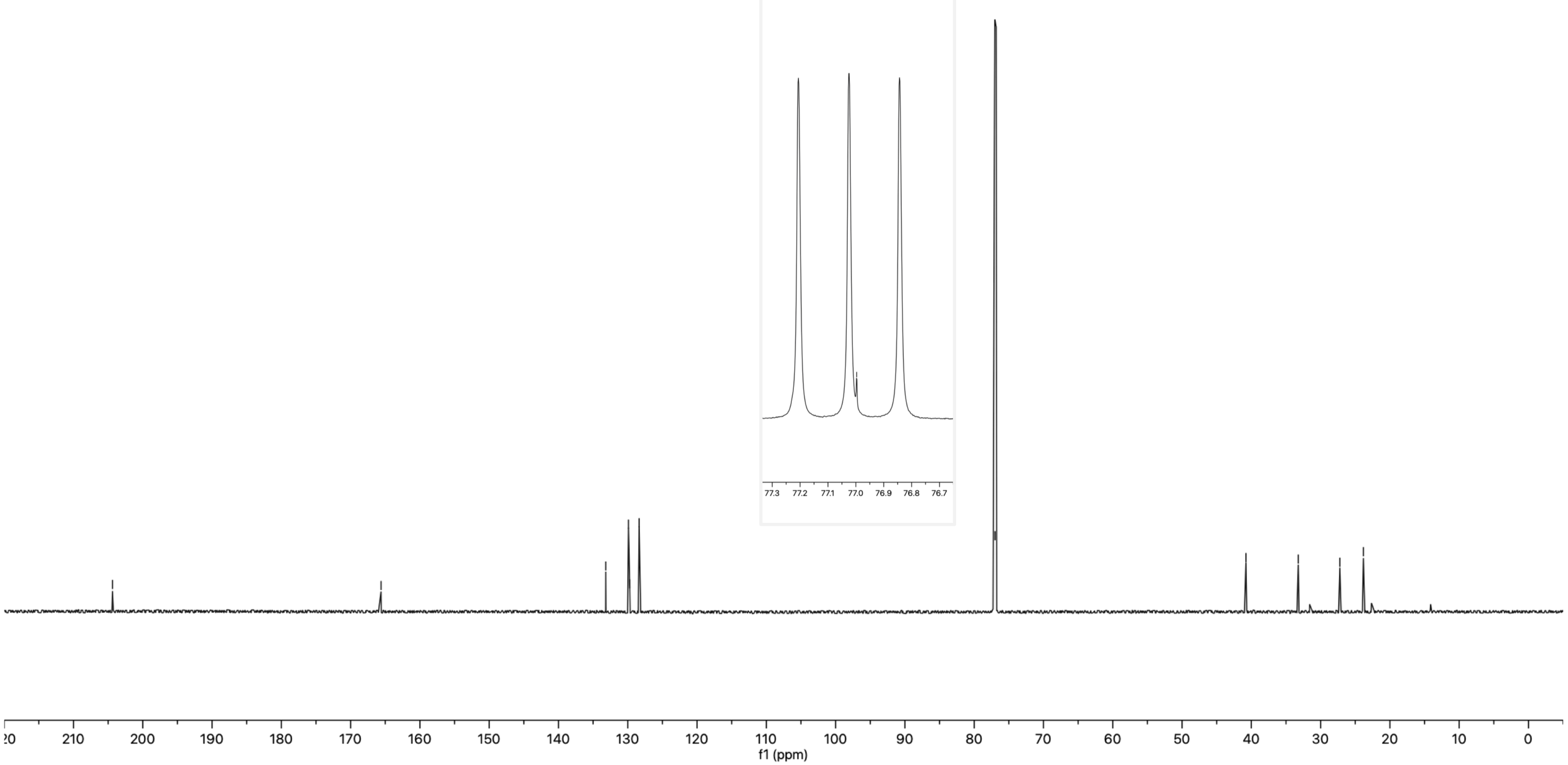

¹H NMR (700 MHz, $CDCl_3$) **Bz-2b**

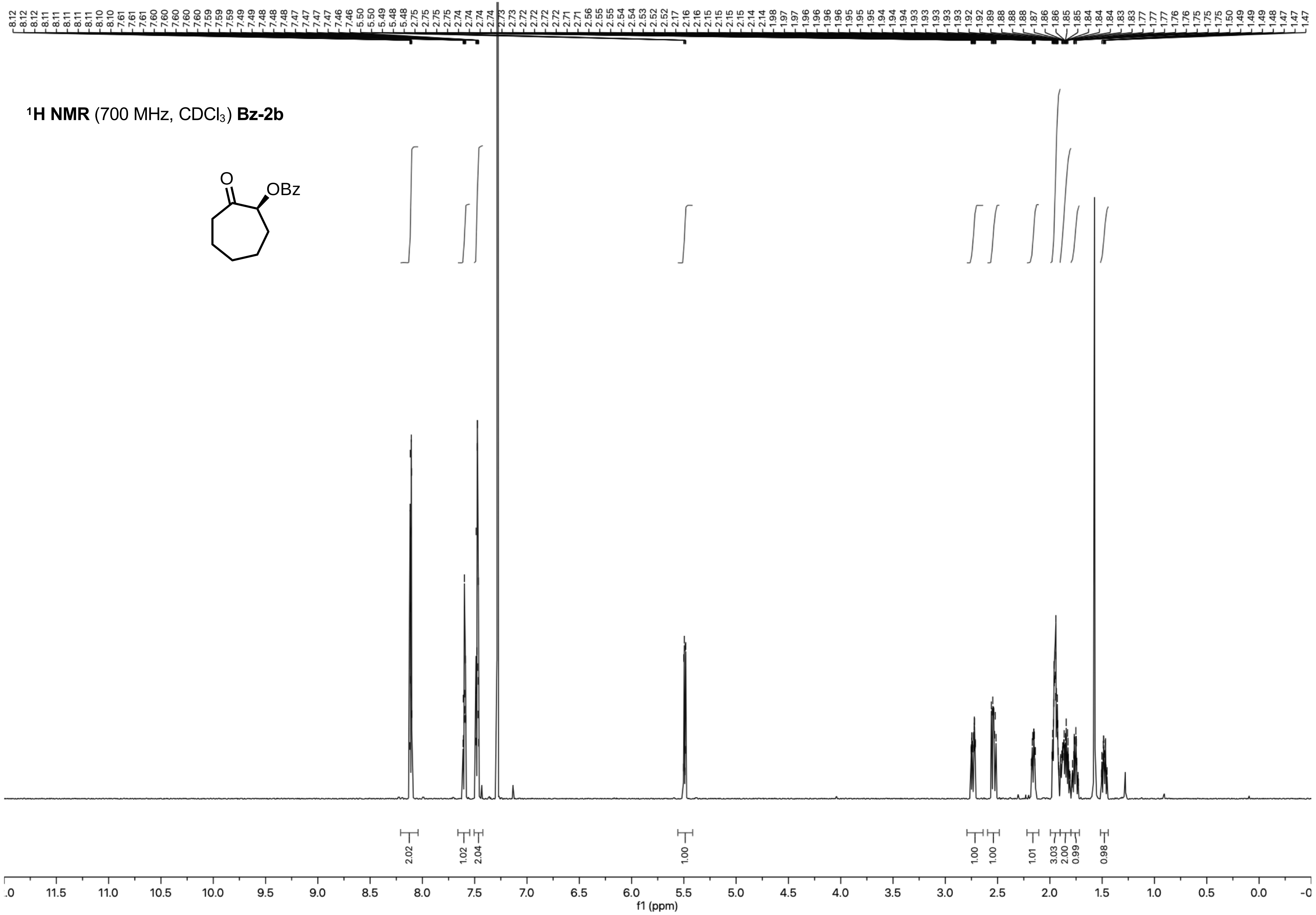

^{13}C NMR (176 MHz, $CDCl_3$) **Bz-2b**

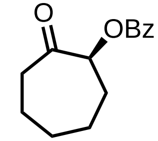

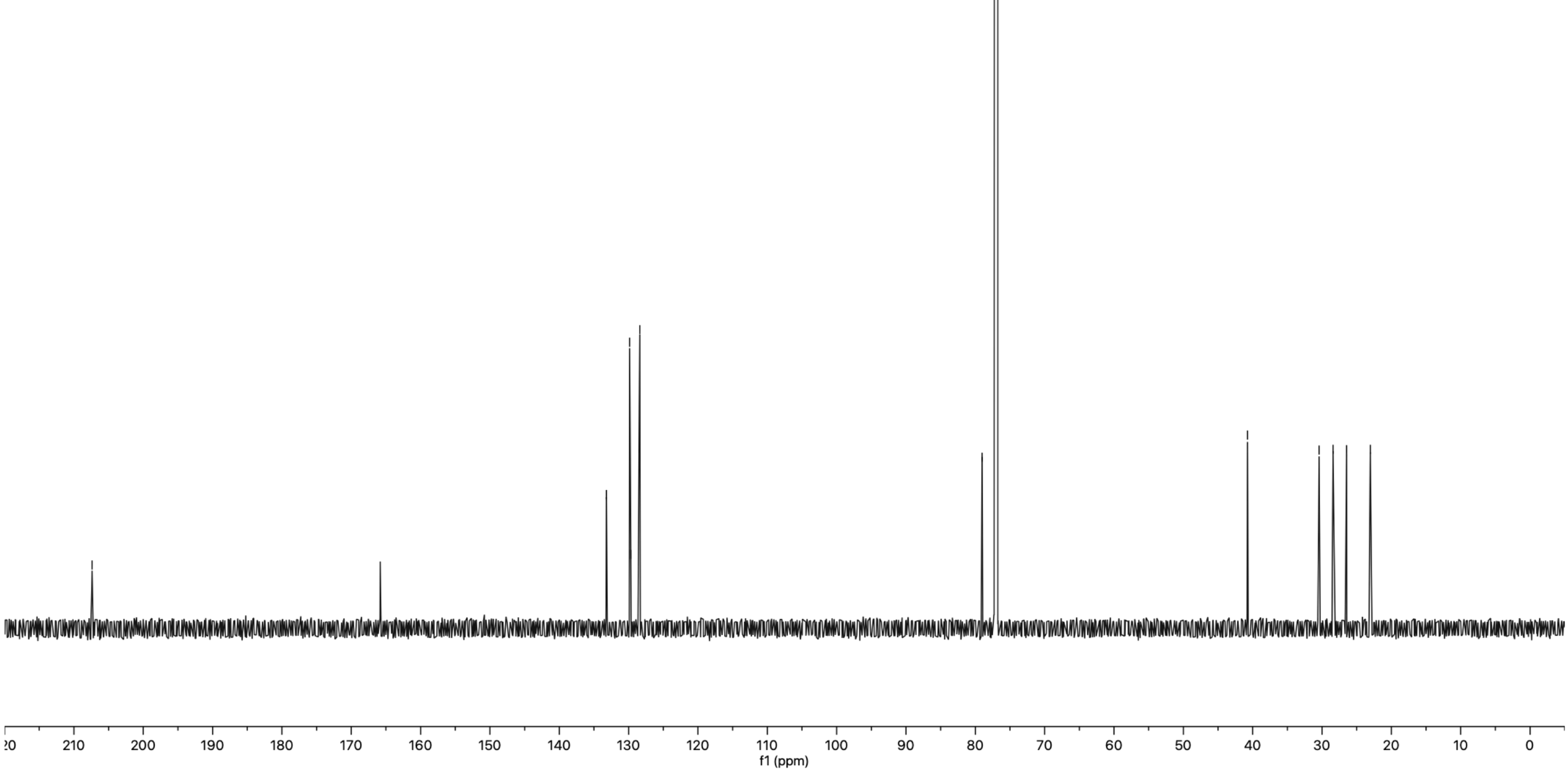

¹H NMR (700 MHz, $CDCl_3$) **Bz-2c**

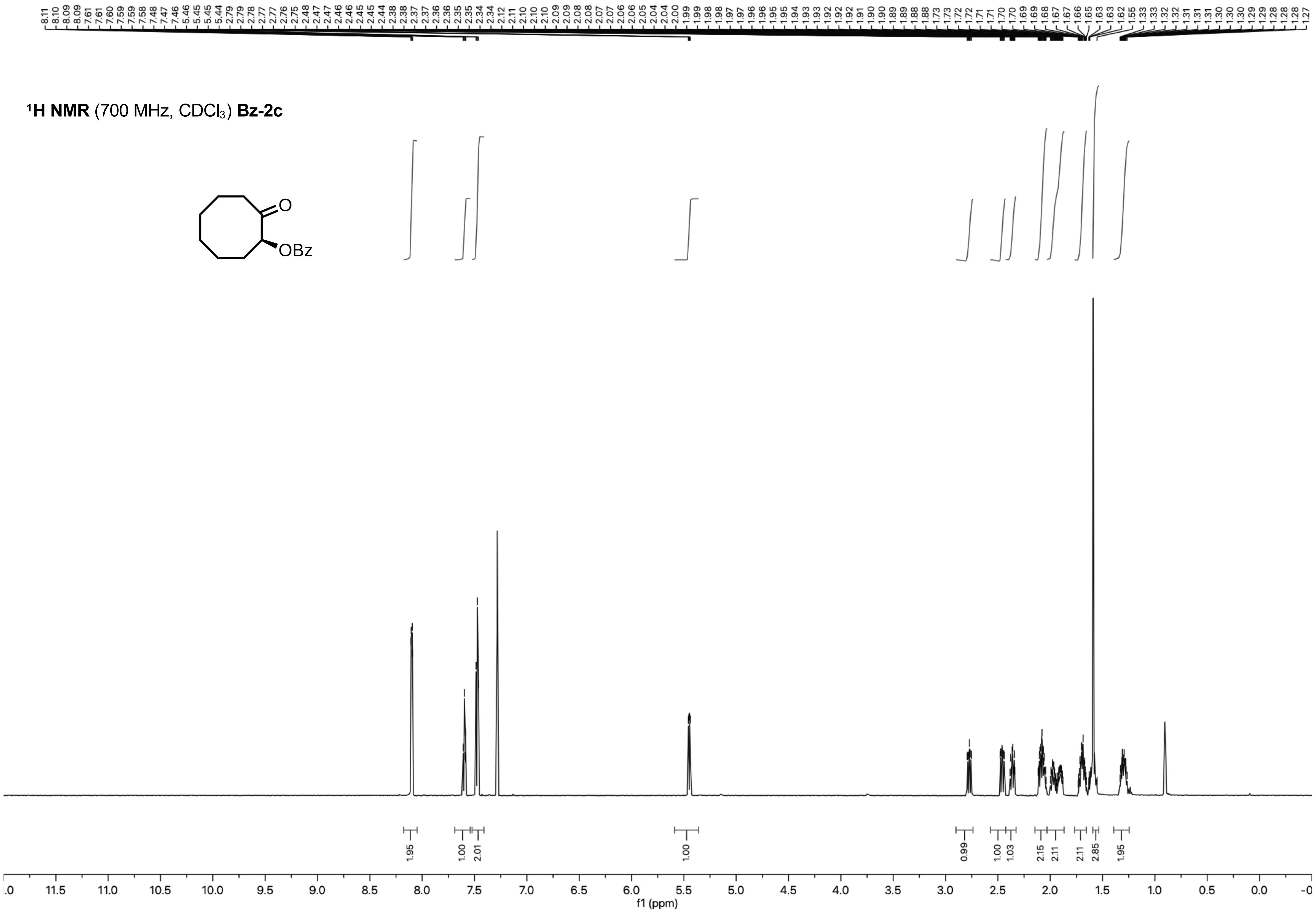

^{13}C NMR (176 MHz, $CDCl_3$) **Bz-2c**

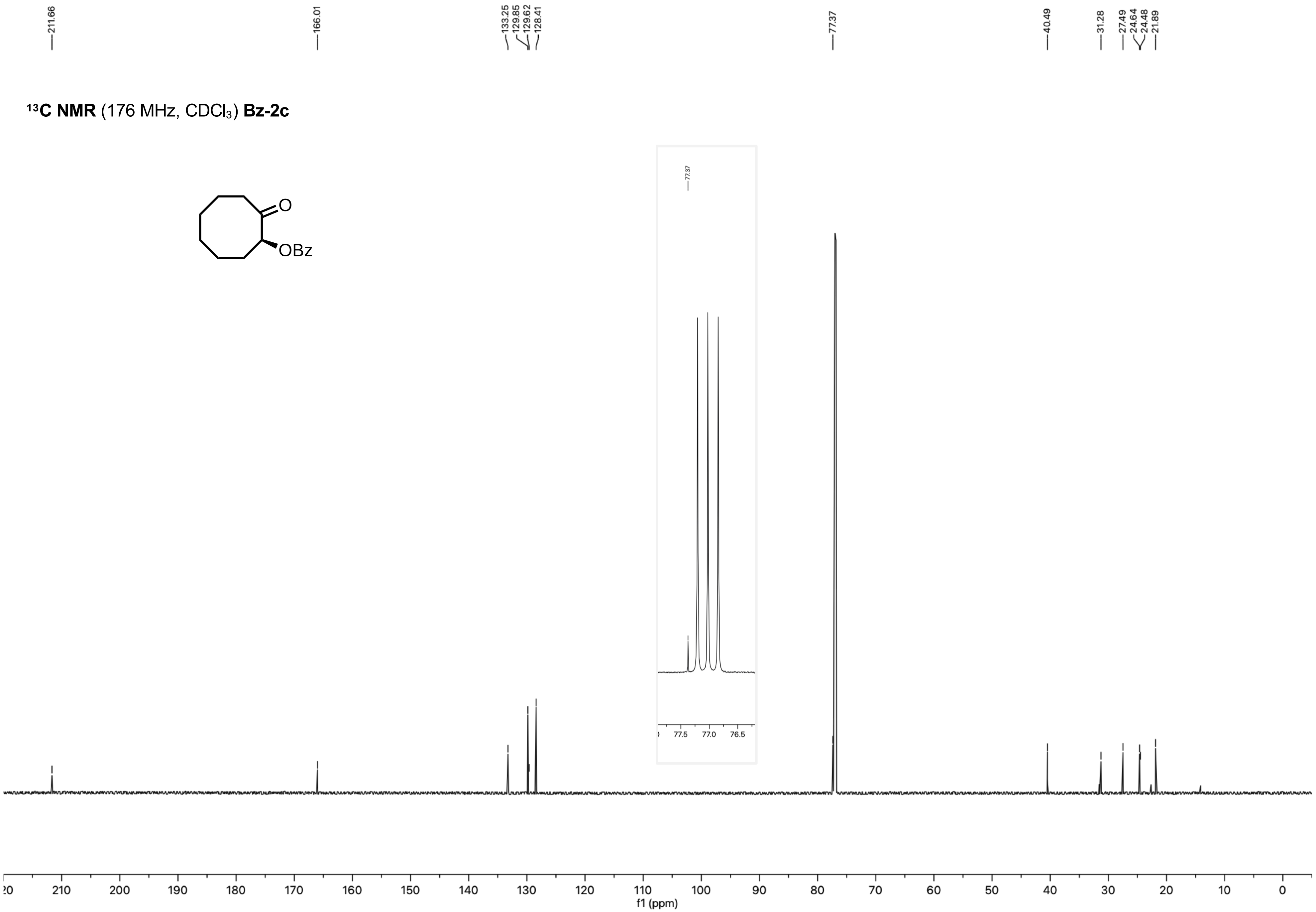

^{1}H NMR (700 MHz, $CDCl_3$) **Bz-2g**

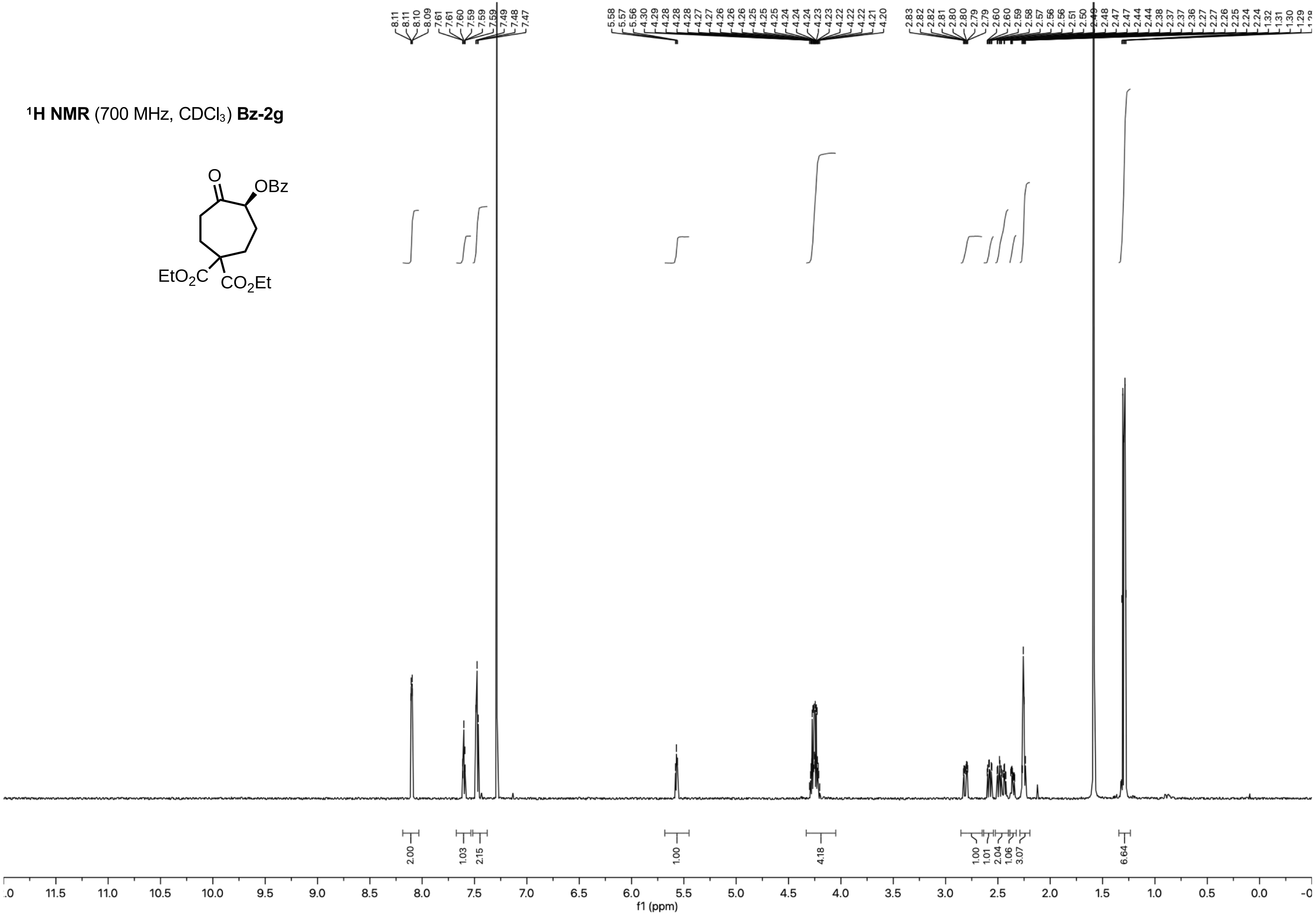

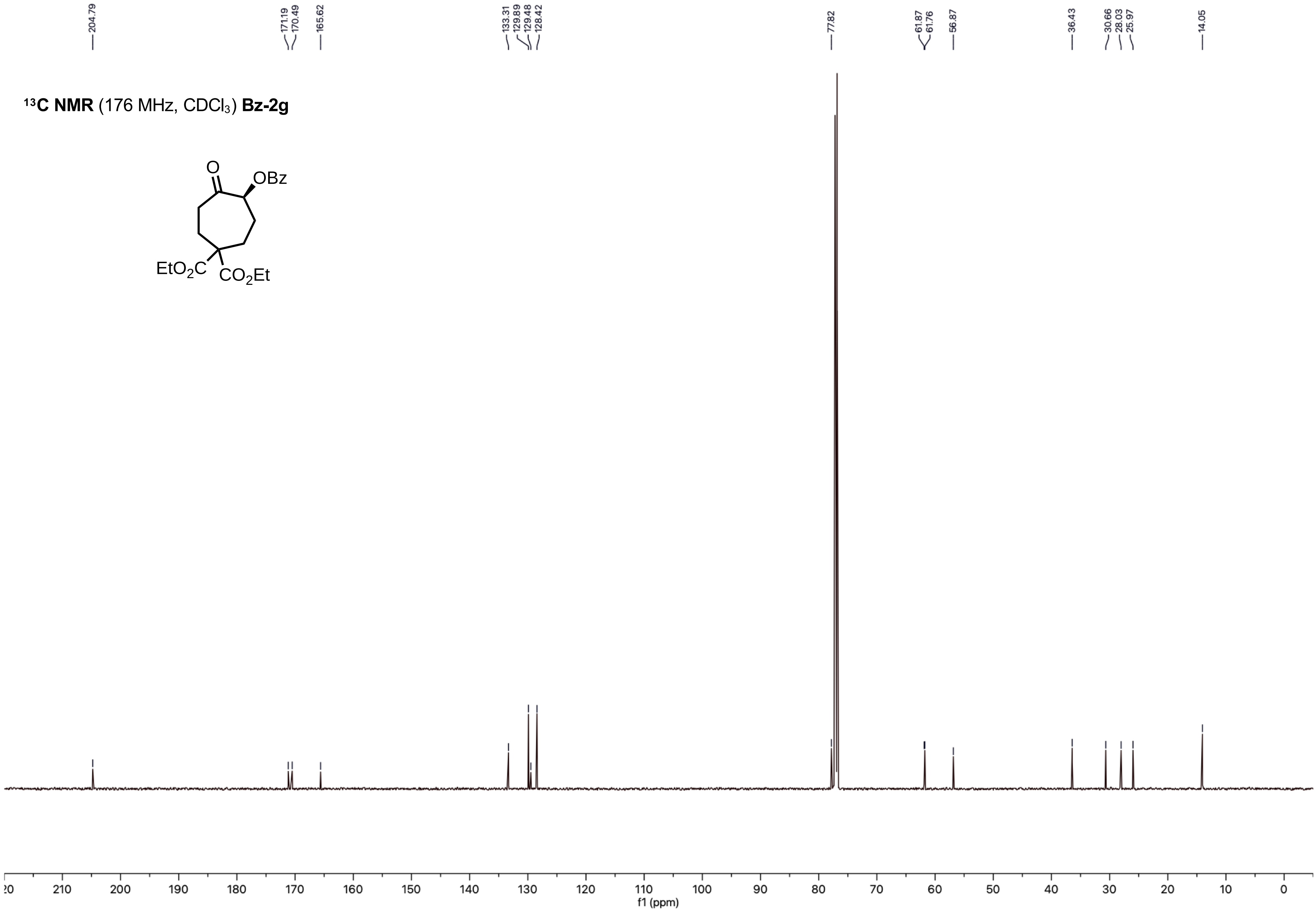

204.79
171.19
170.49
165.62
133.31
129.89
129.48
128.42
77.82
61.87
61.76
56.87
36.43
30.66
28.03
25.97
14.05
^{13}C NMR (176 MHz, CDCl$_3$) Bz-2g
O
OBz
EtO$_2$C
CO$_2$Et
f1 (ppm)

¹H NMR (700 MHz, $CDCl_3$) **Bz-2i**

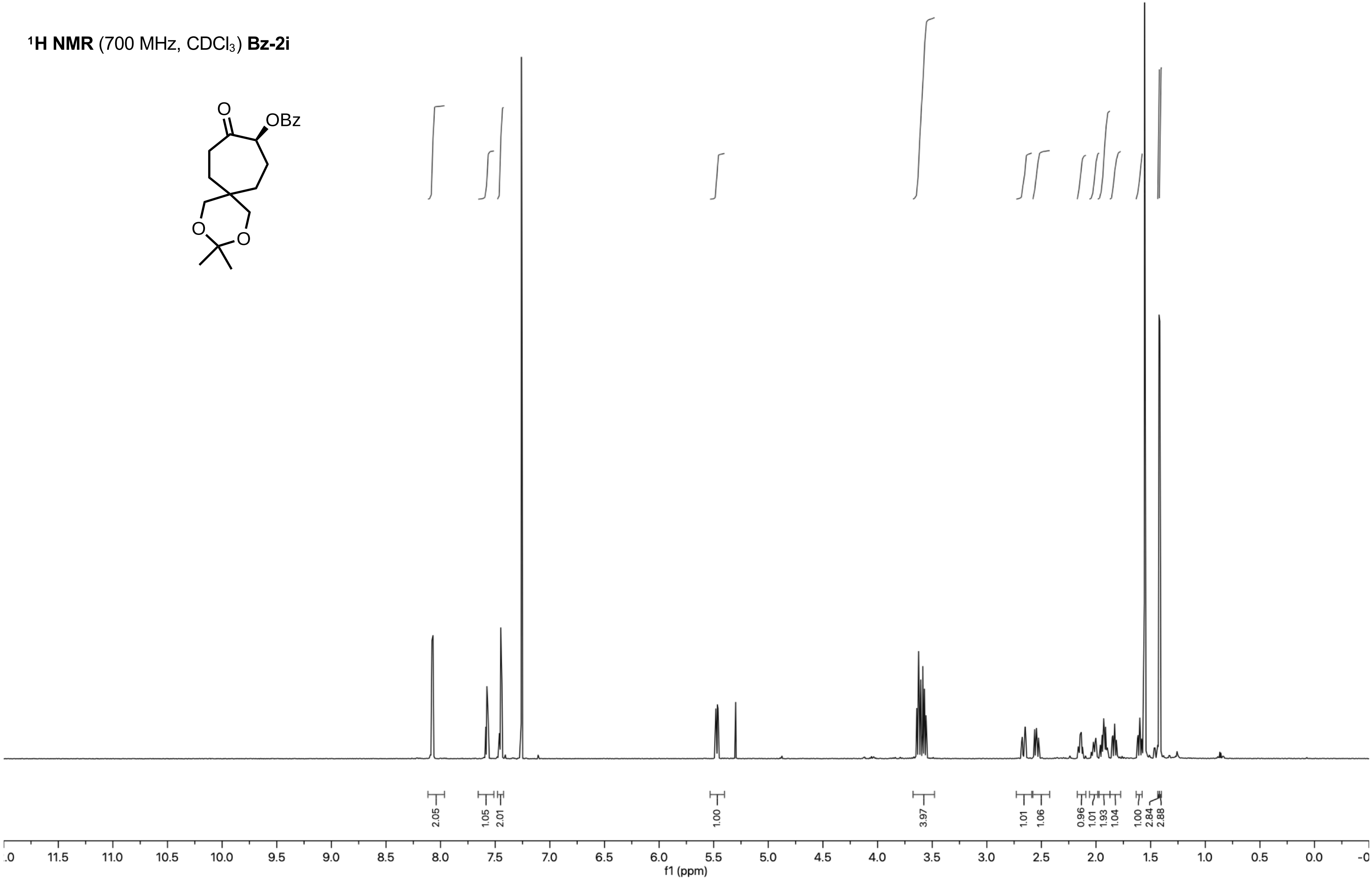

^{13}C NMR (176 MHz, $CDCl_3$) **Bz-2i**

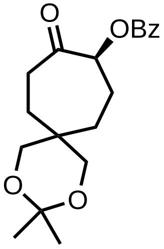

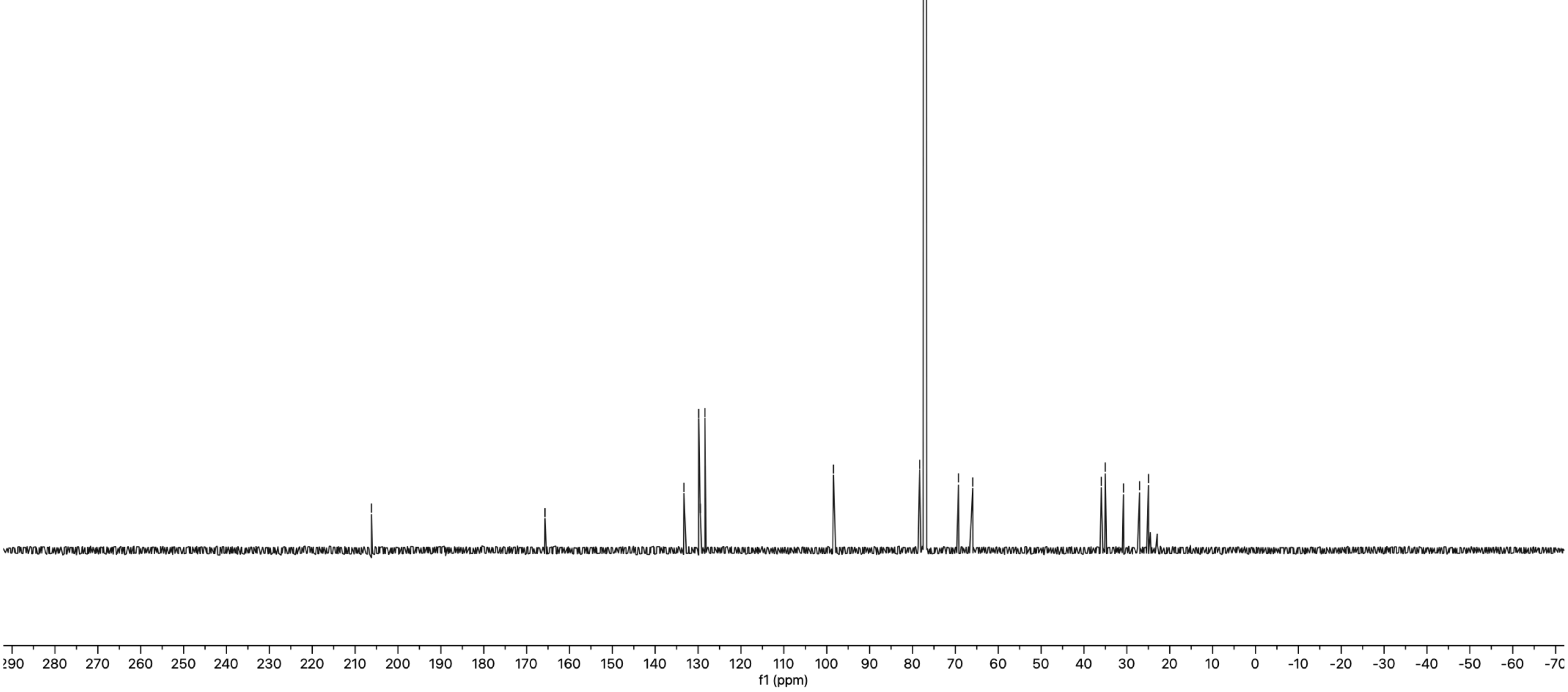

^{1}H NMR (700 MHz, $CDCl_3$) **Bz-2j**

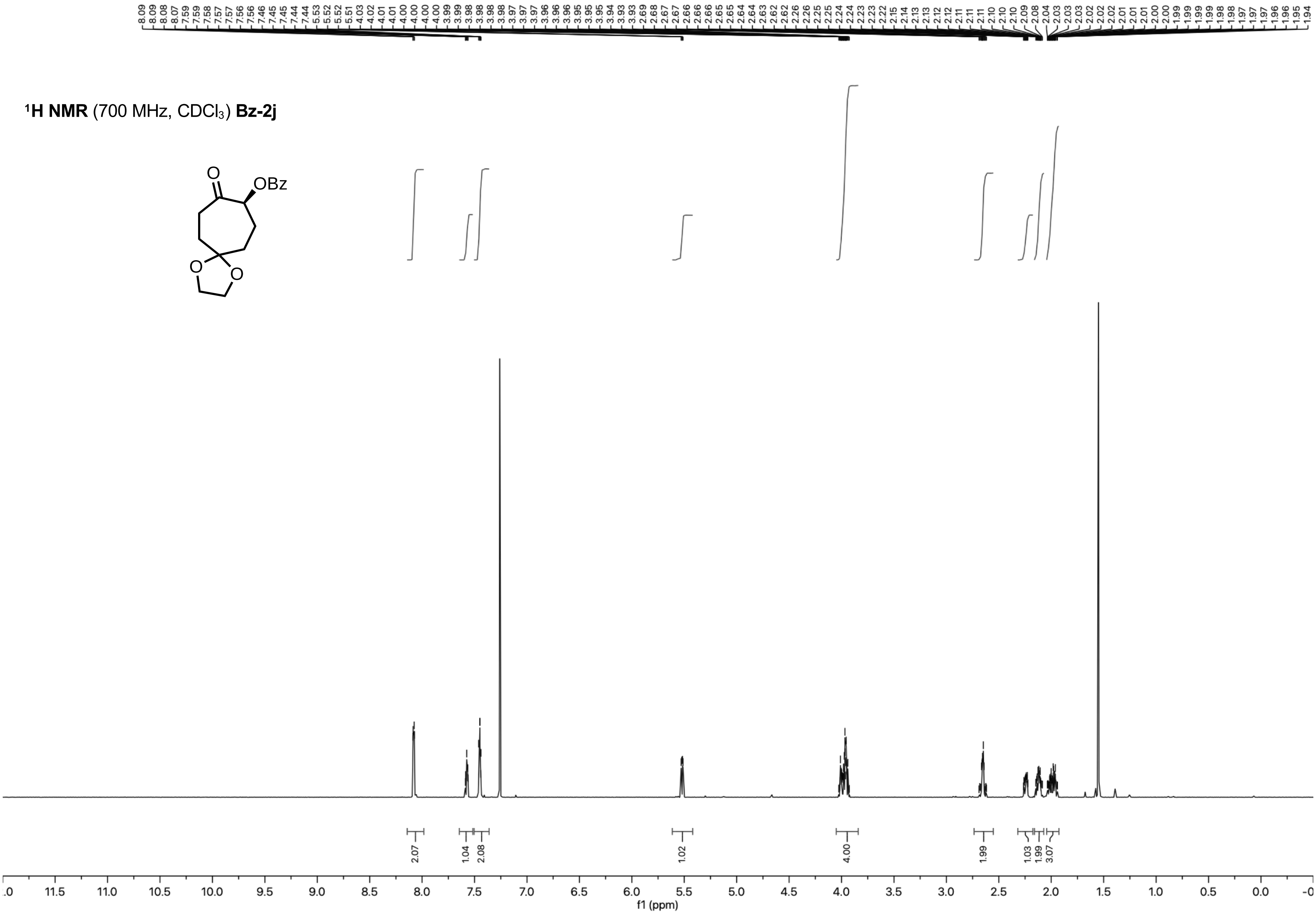

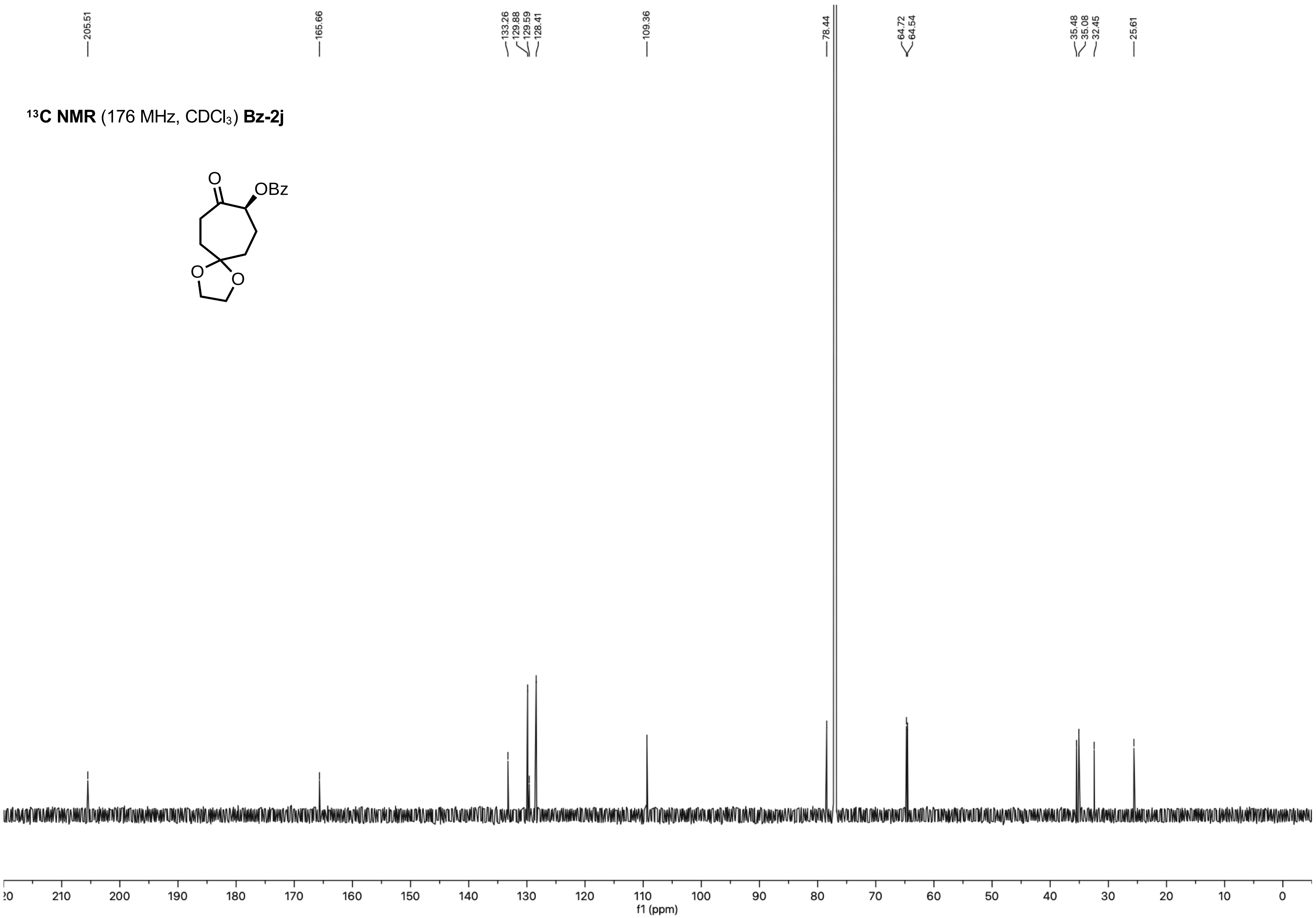

205.51
165.66
133.26
129.88
129.59
128.41
109.36
78.44
64.72
64.54
35.48
35.08
32.45
25.61
13C NMR (176 MHz, CDCl3) Bz-2j
O
OBz
O
O
20
210
200
190
180
170
160
150
140
130
120
110
100
90
80
70
60
50
40
30
20
10
0
f1 (ppm)

¹H NMR (700 MHz, $CDCl_3$) **Bz-2k**

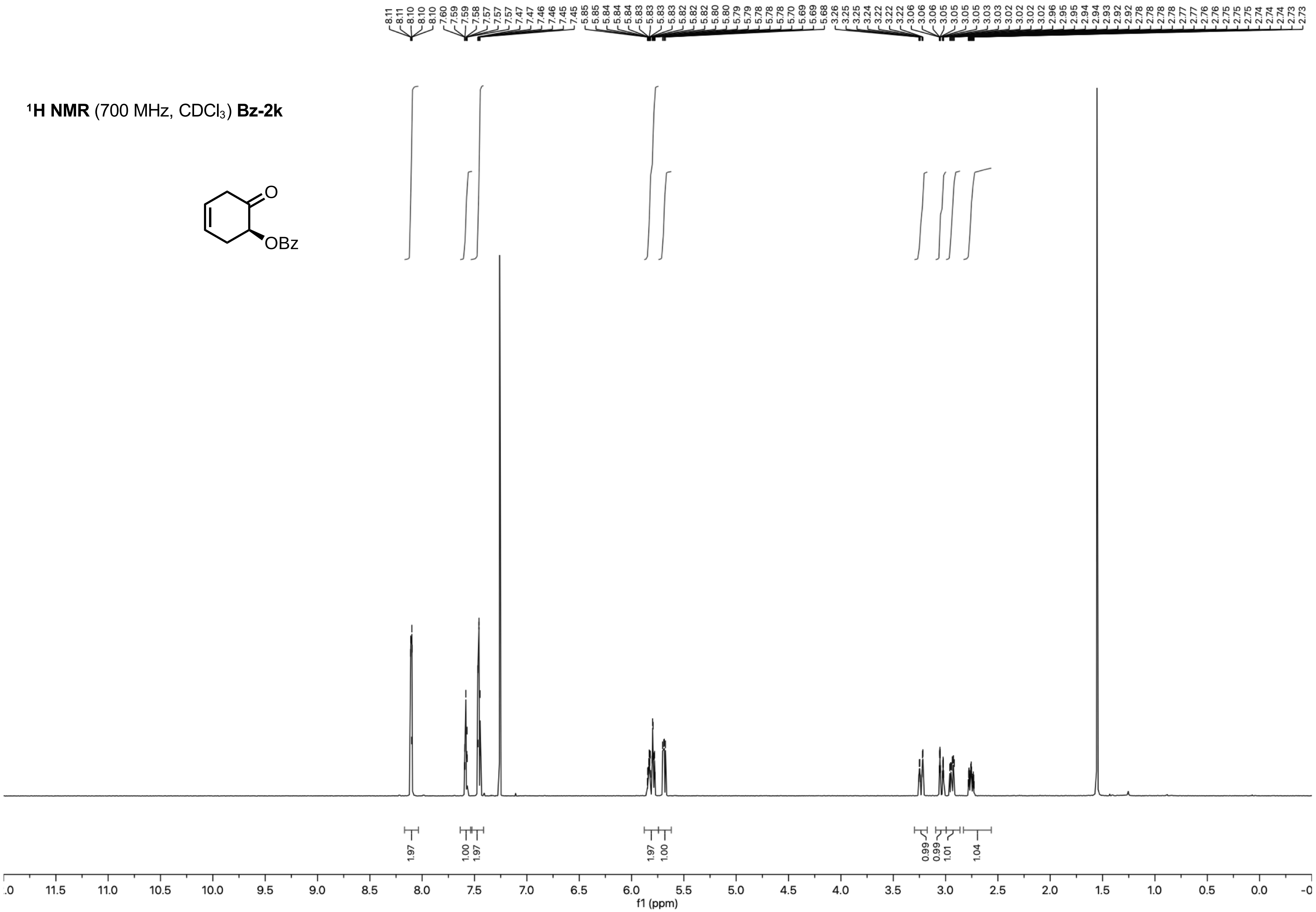

^{13}C NMR (176 MHz, $CDCl_3$) **Bz-2k**

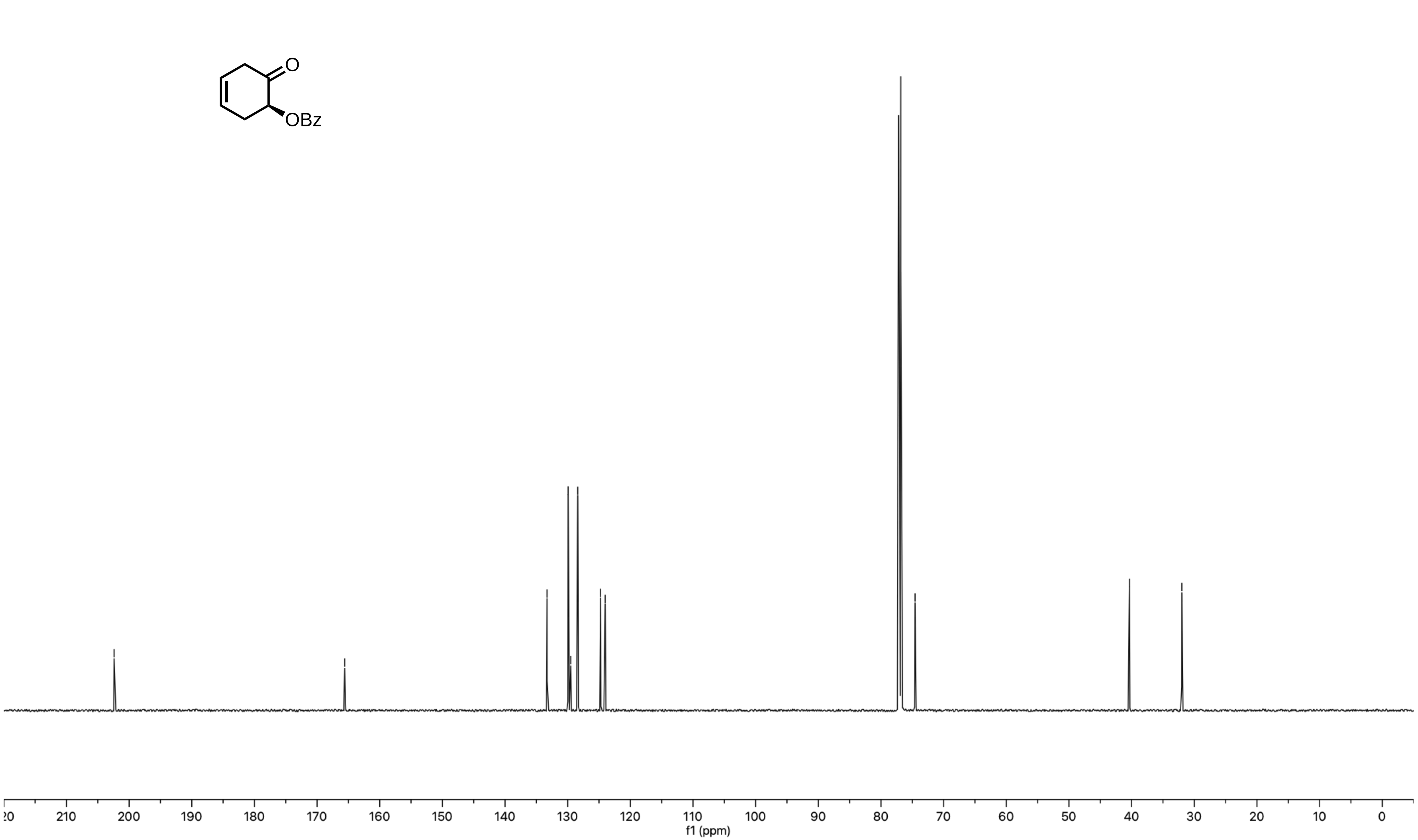

¹H NMR (400 MHz, $CDCl_3$) **Bz-2l**

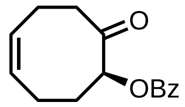

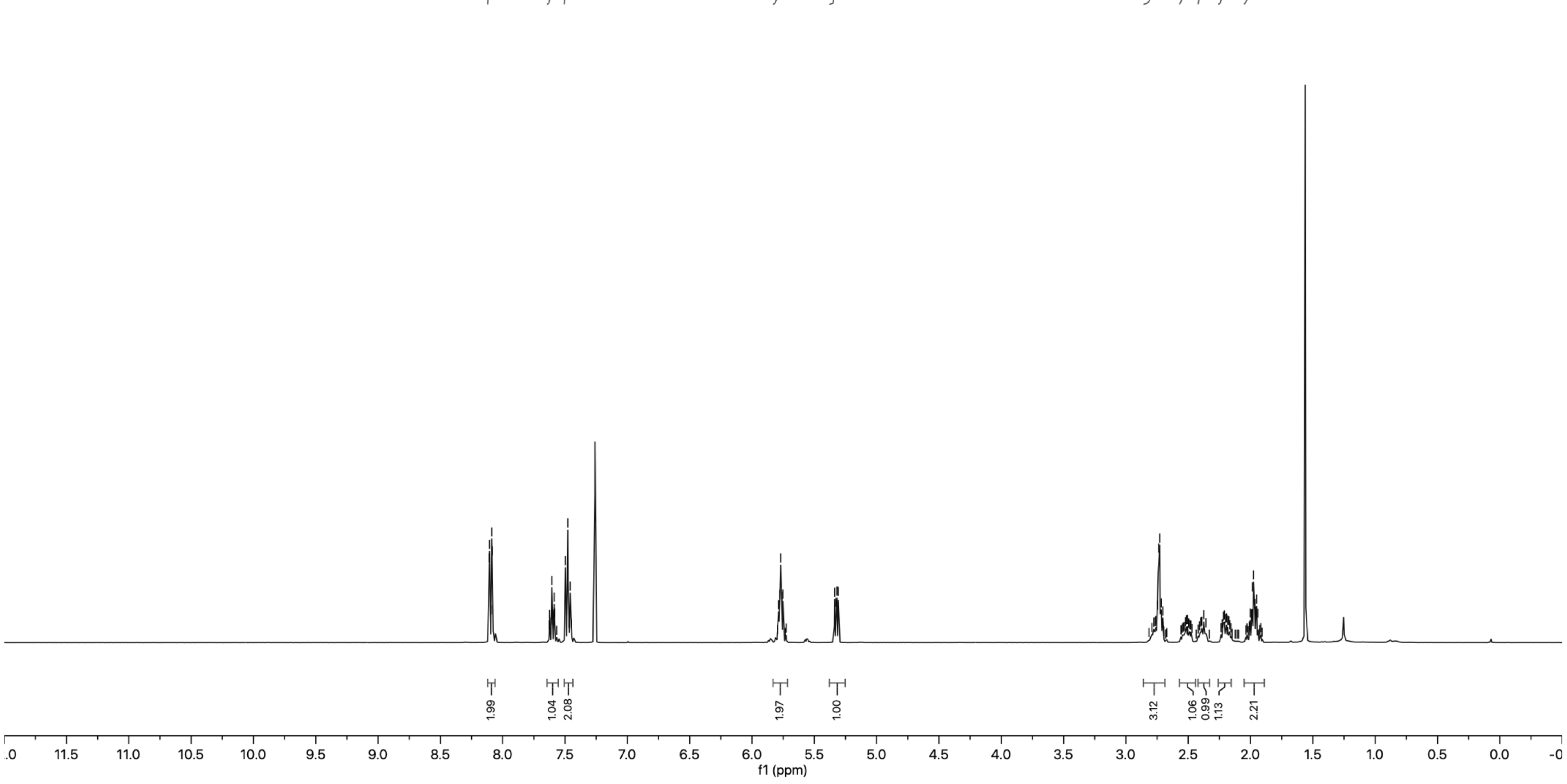

^{13}C NMR (101 MHz, $CDCl_3$) **Bz-2l**

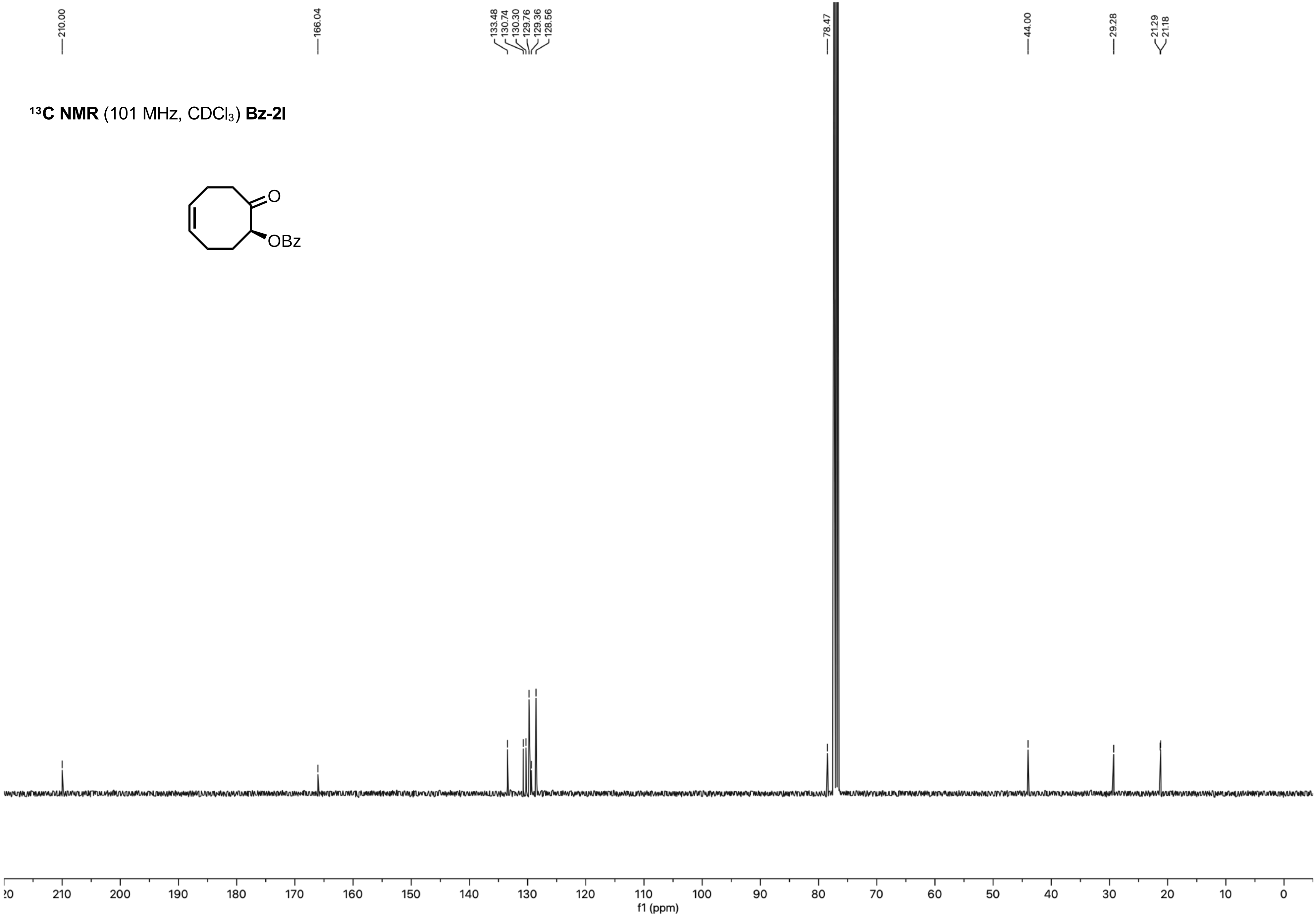

¹H NMR (700 MHz, $CDCl_3$) **Bz-2m**

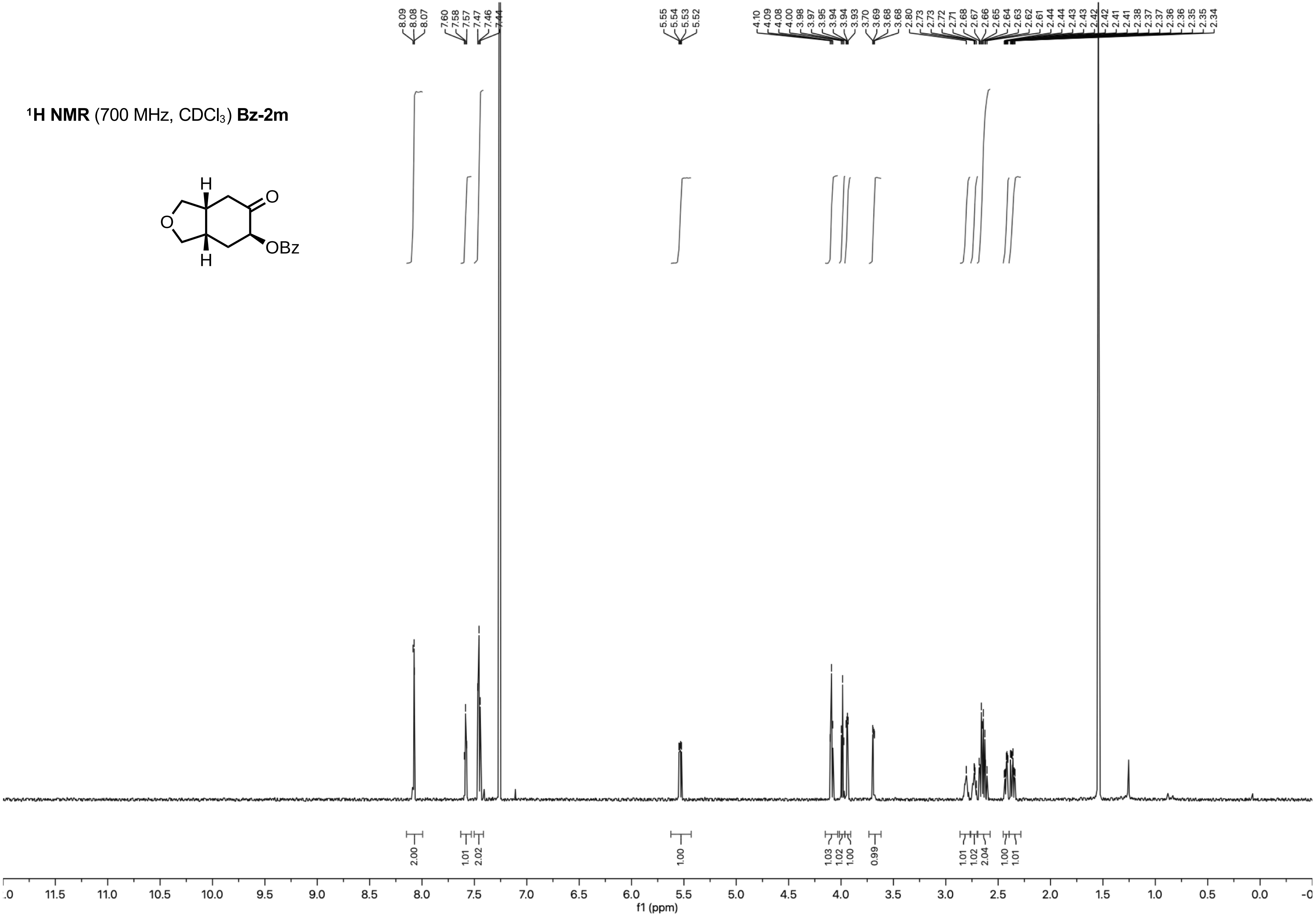

^{13}C NMR (176 MHz, $CDCl_3$) **Bz-2m**

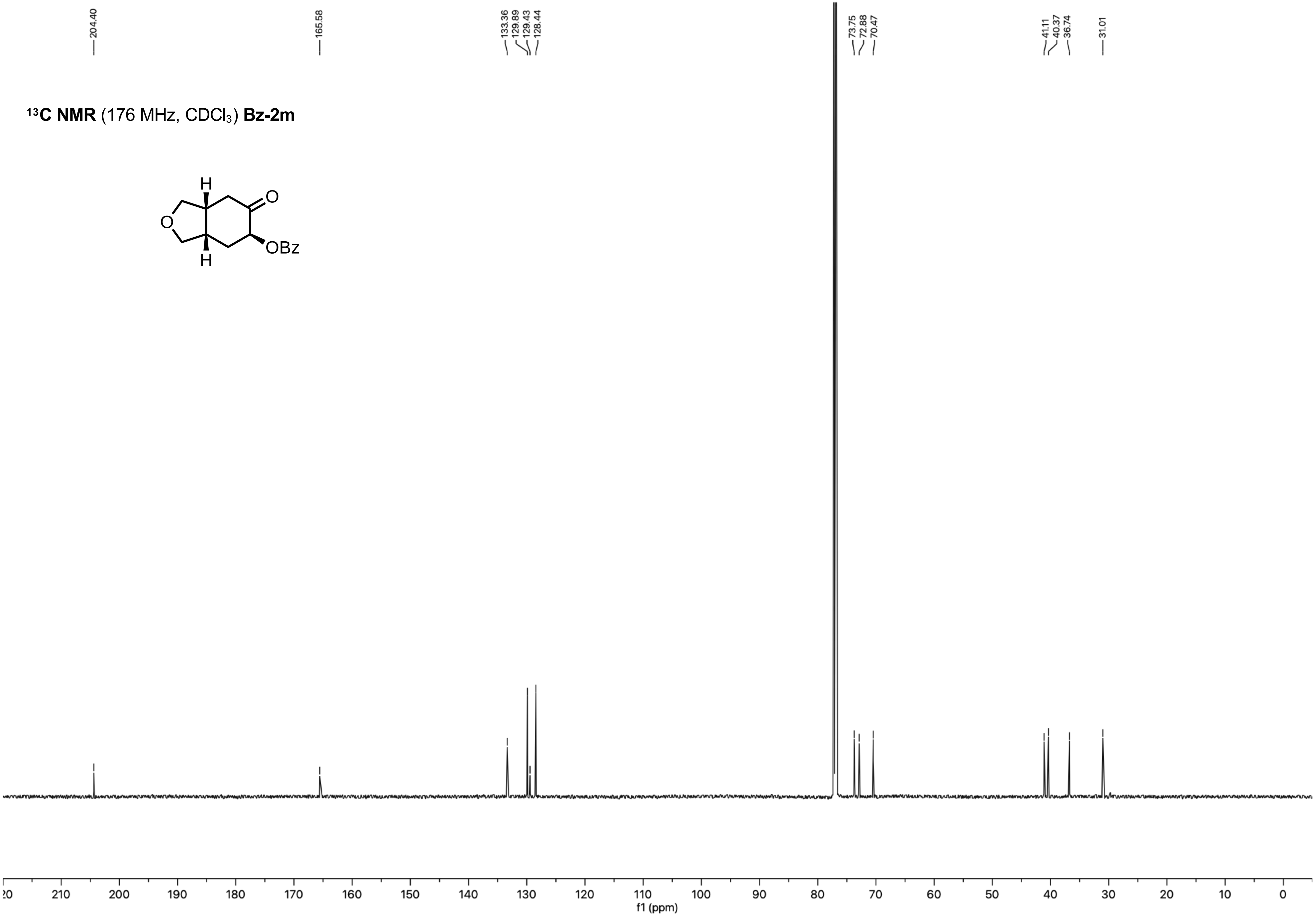

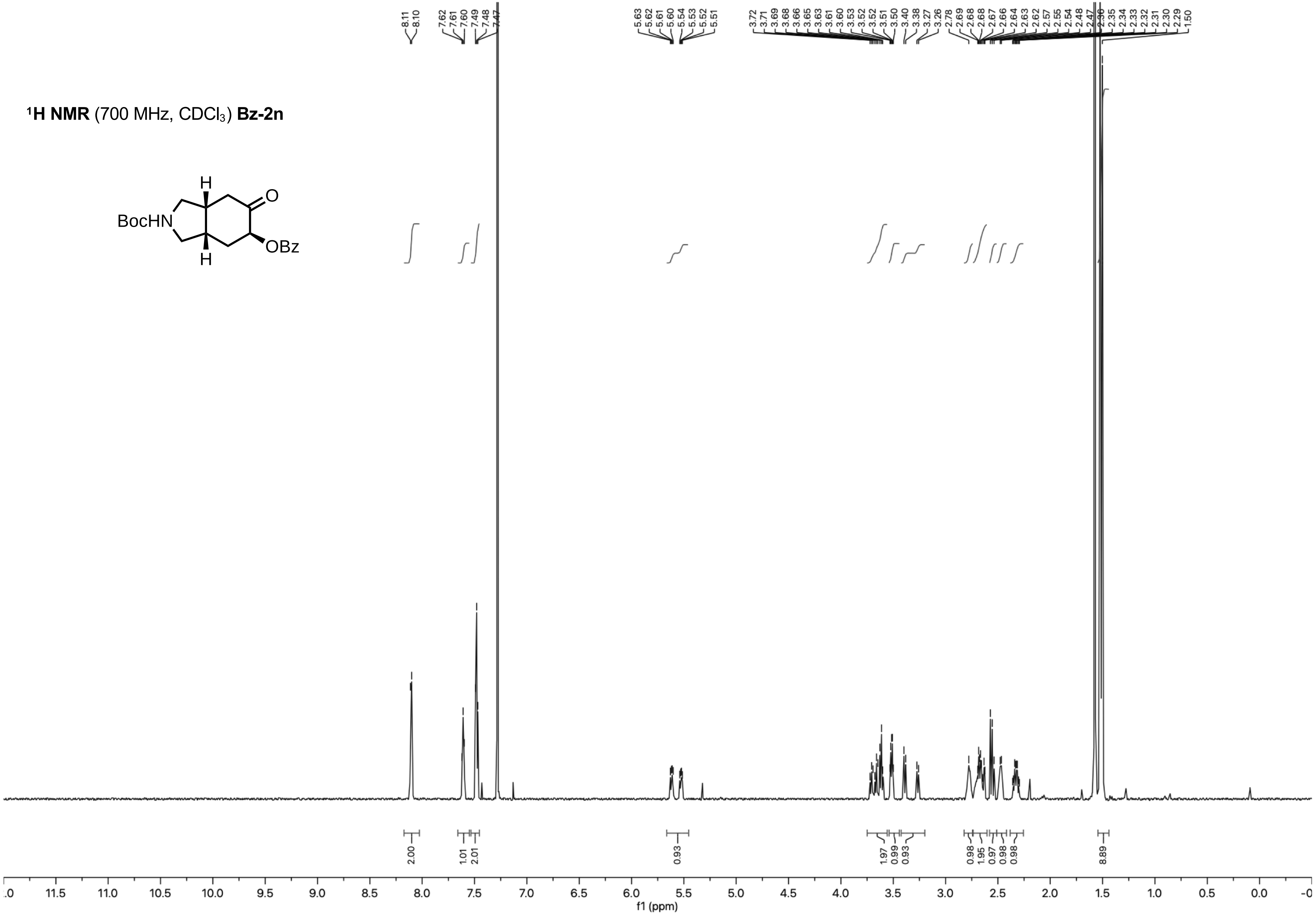

¹H NMR (700 MHz, CDCl₃) Bz-2n
BocHN
OBz
f1 (ppm)

^{13}C NMR (176 MHz, $CDCl_3$) **Bz-2n**

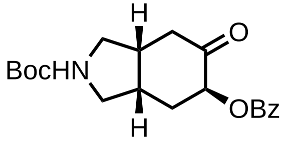

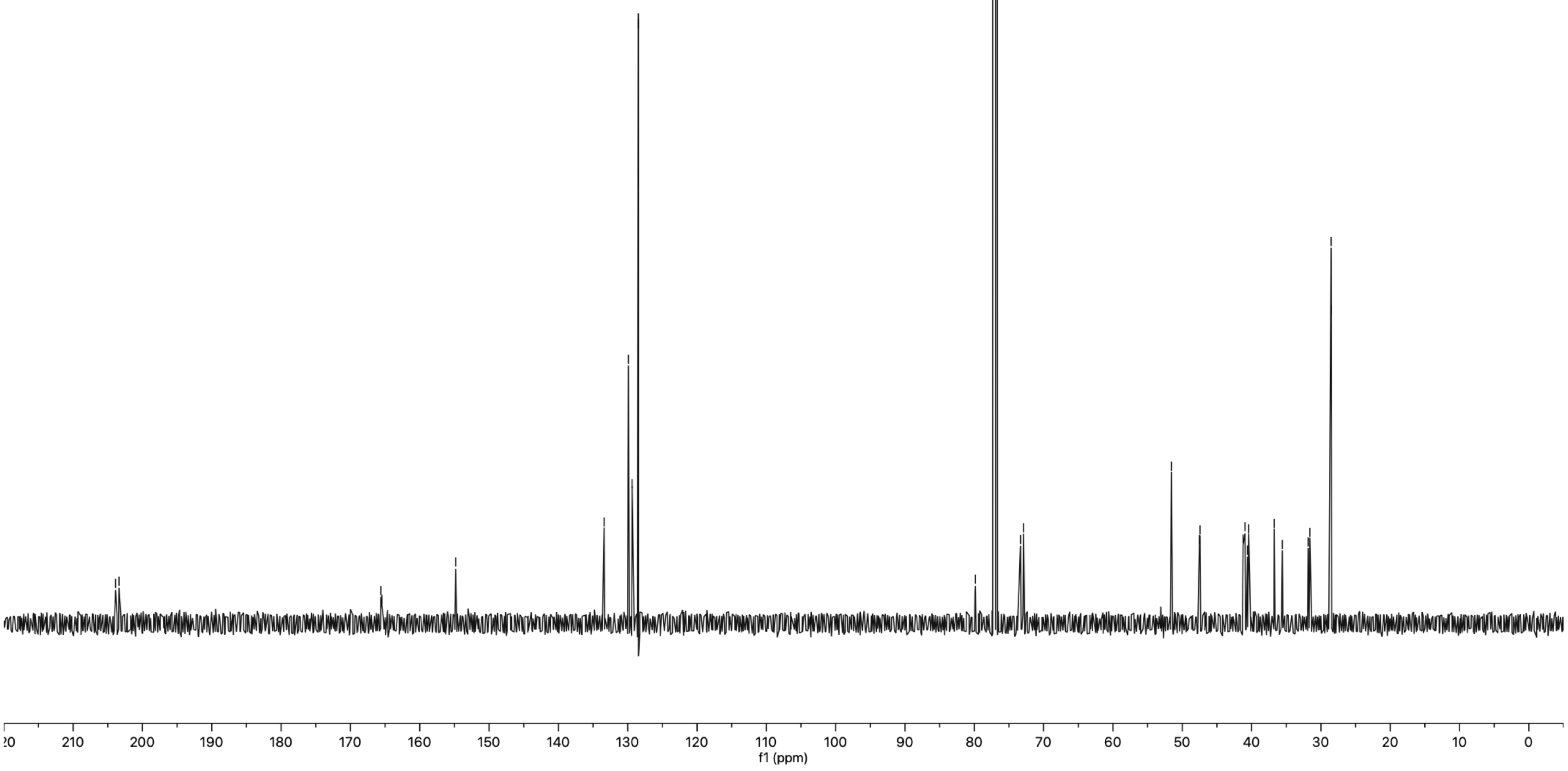

^{1}H NMR (400 MHz, $CDCl_3$) **Bz-2o**

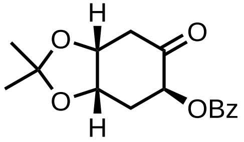

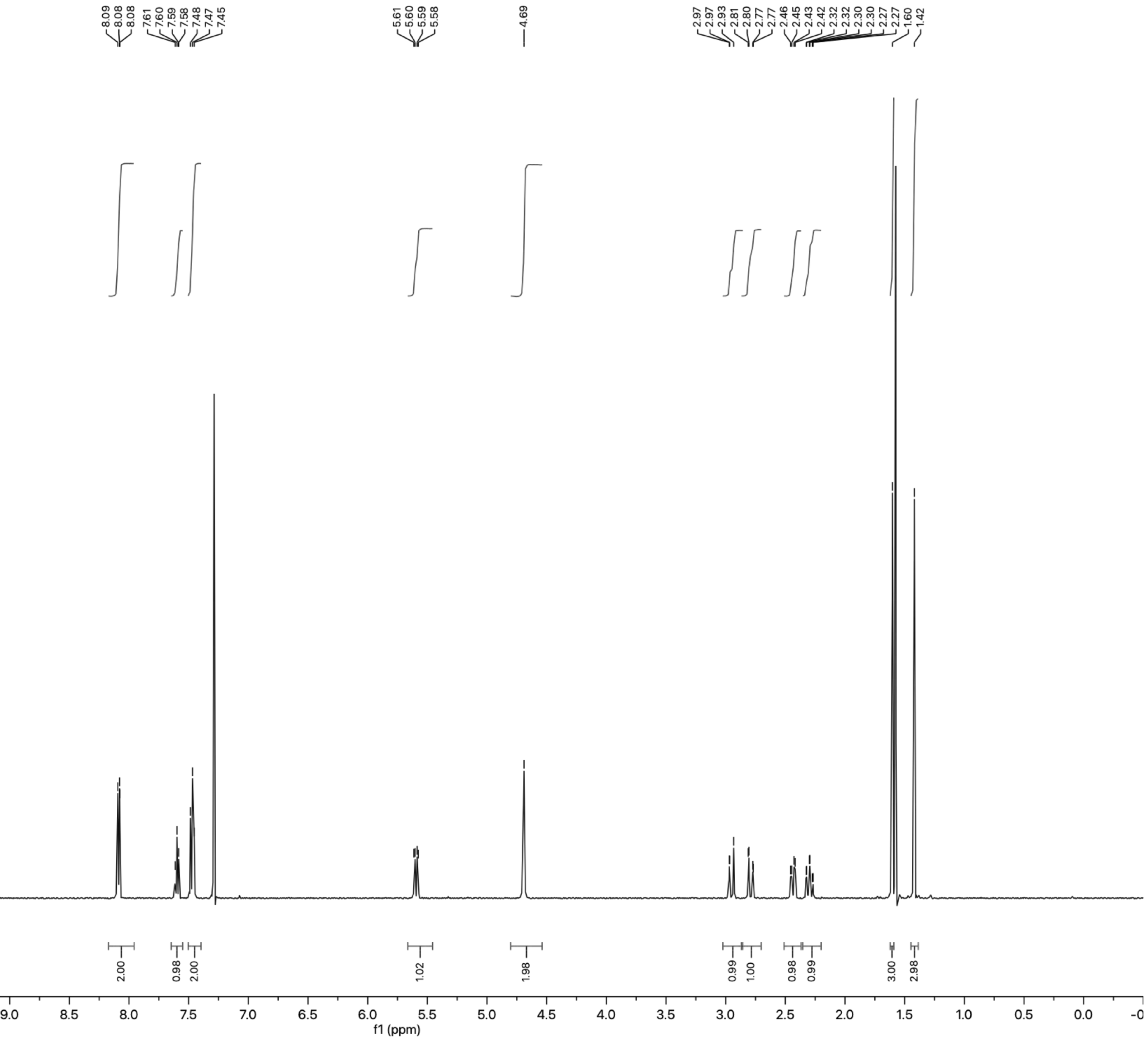

^{13}C NMR (101 MHz, $CDCl_3$) **Bz-2o**

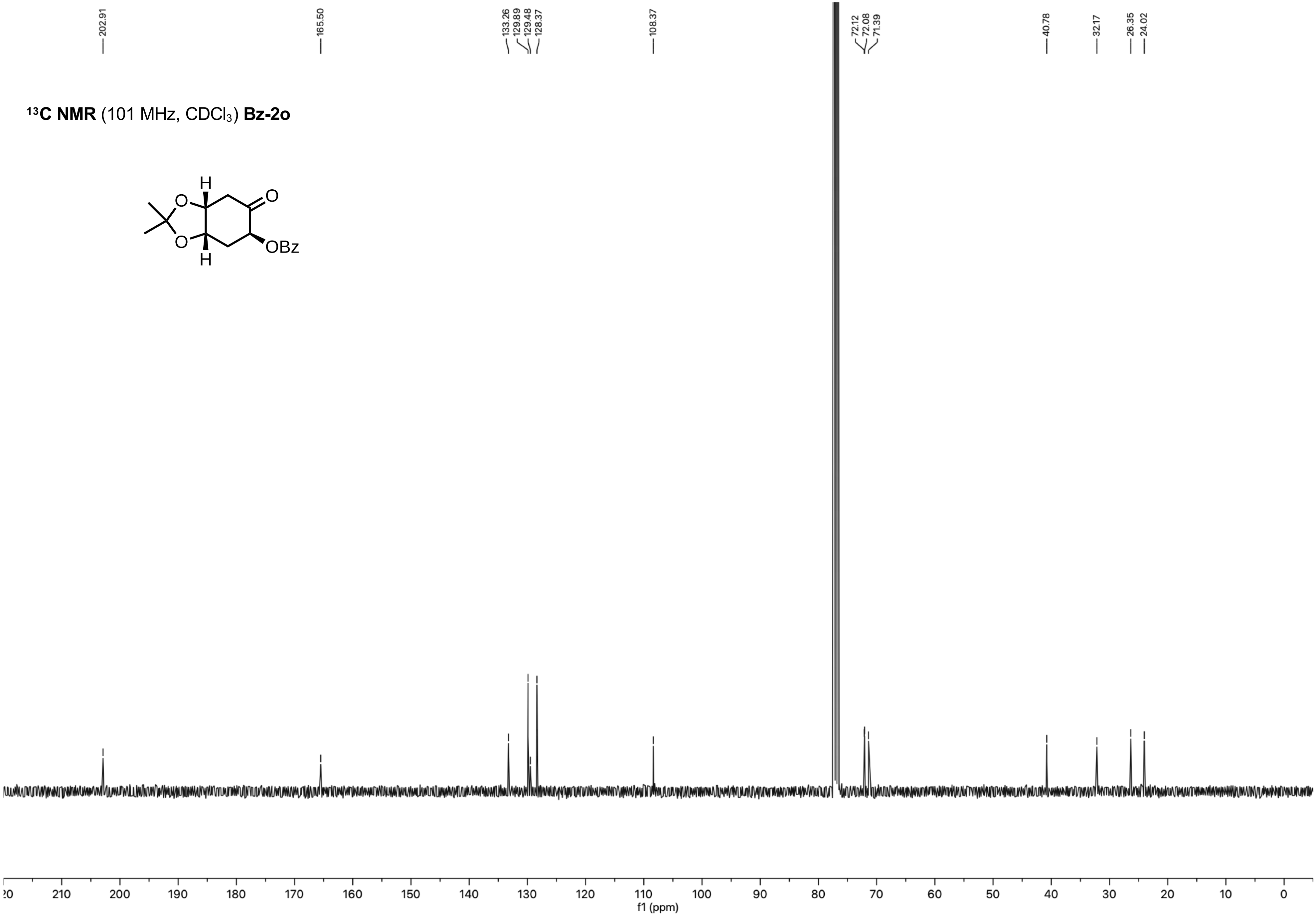

^{1}H NMR (700 MHz, $CDCl_3$) **Bz-2p**

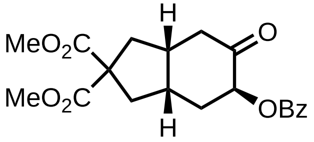

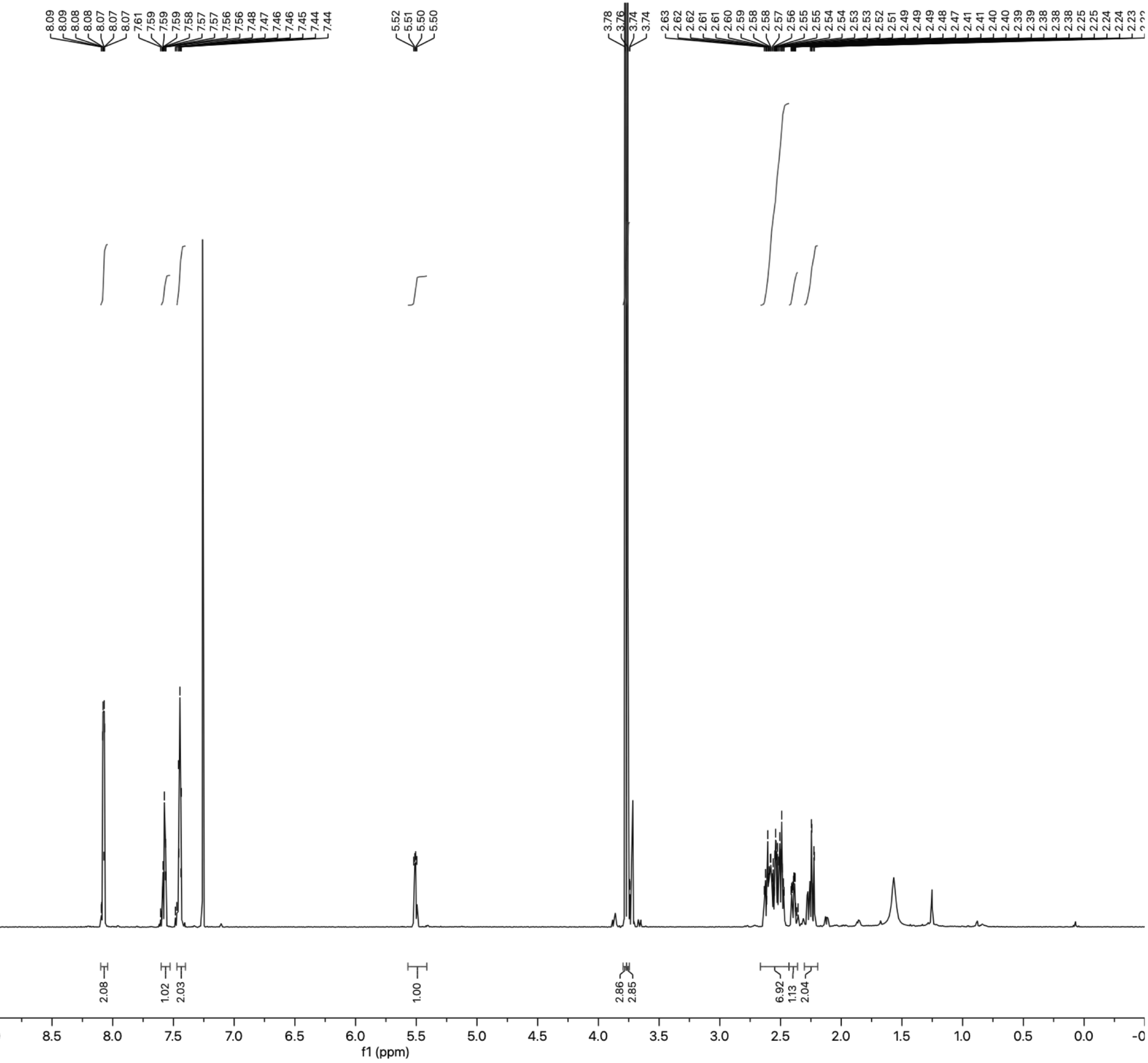

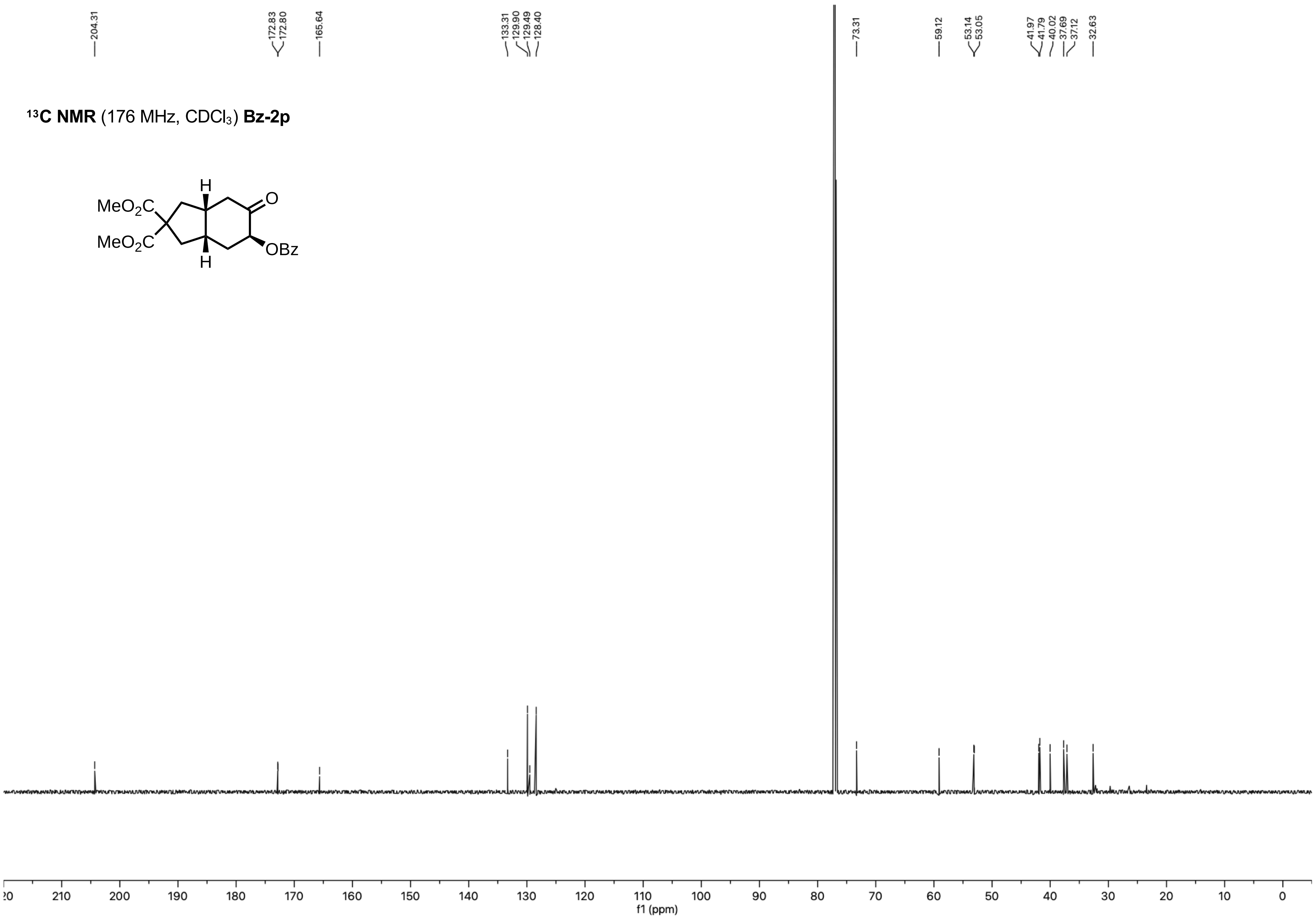
204.31
172.83
172.80
165.64
133.31
129.90
129.49
128.40
73.31
59.12
53.14
53.05
41.97
41.79
40.02
37.69
37.12
32.63
¹³C NMR (176 MHz, CDCl₃) Bz-2p
MeO₂C
MeO₂C
H
H
O
OBz
210
200
190
180
170
160
150
140
130
120
110
100
90
80
70
60
50
40
30
20
10
0
f1 (ppm)

¹H NMR (700 MHz, $CDCl_3$) **Bz-2q**

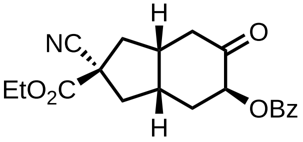

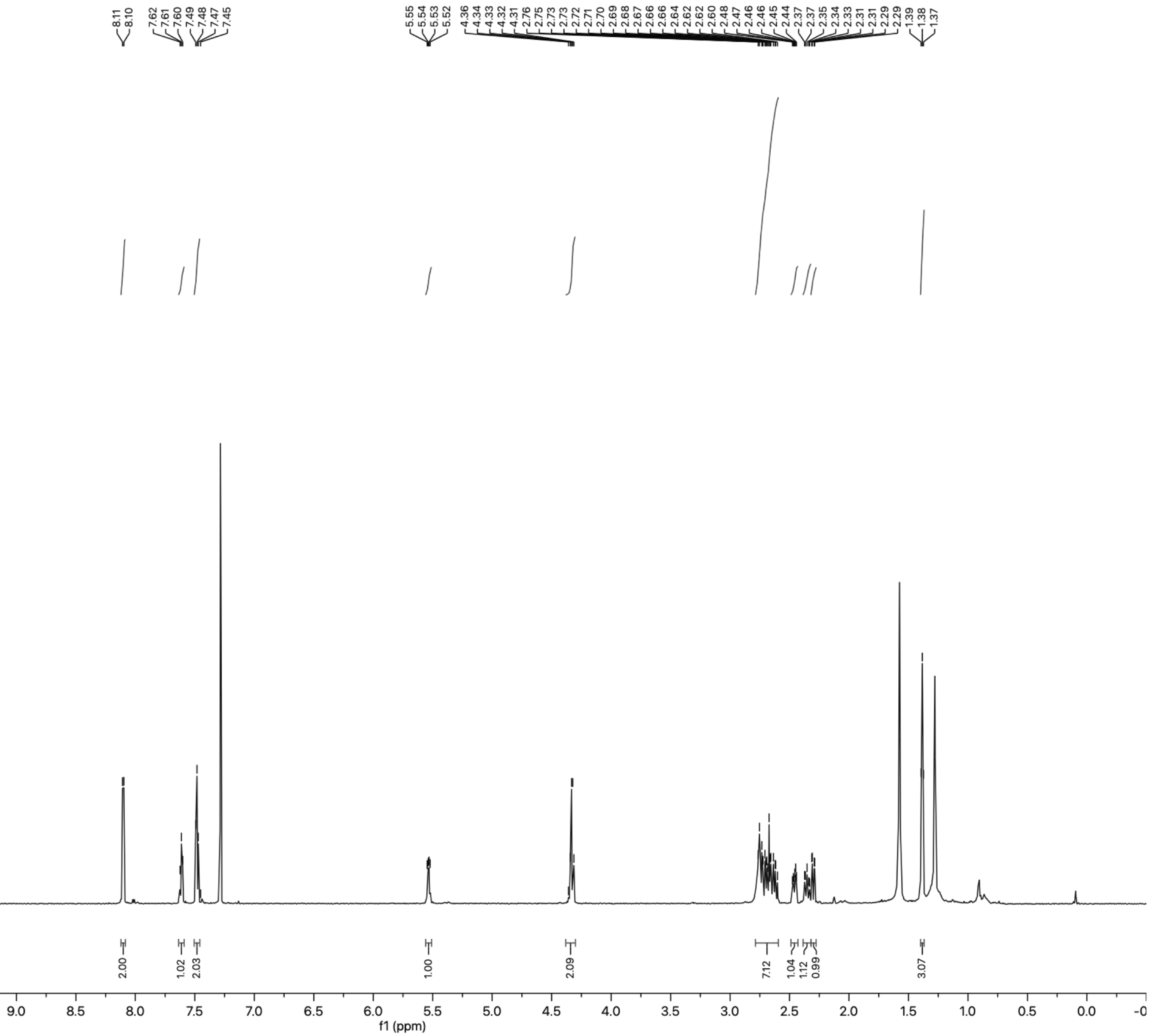

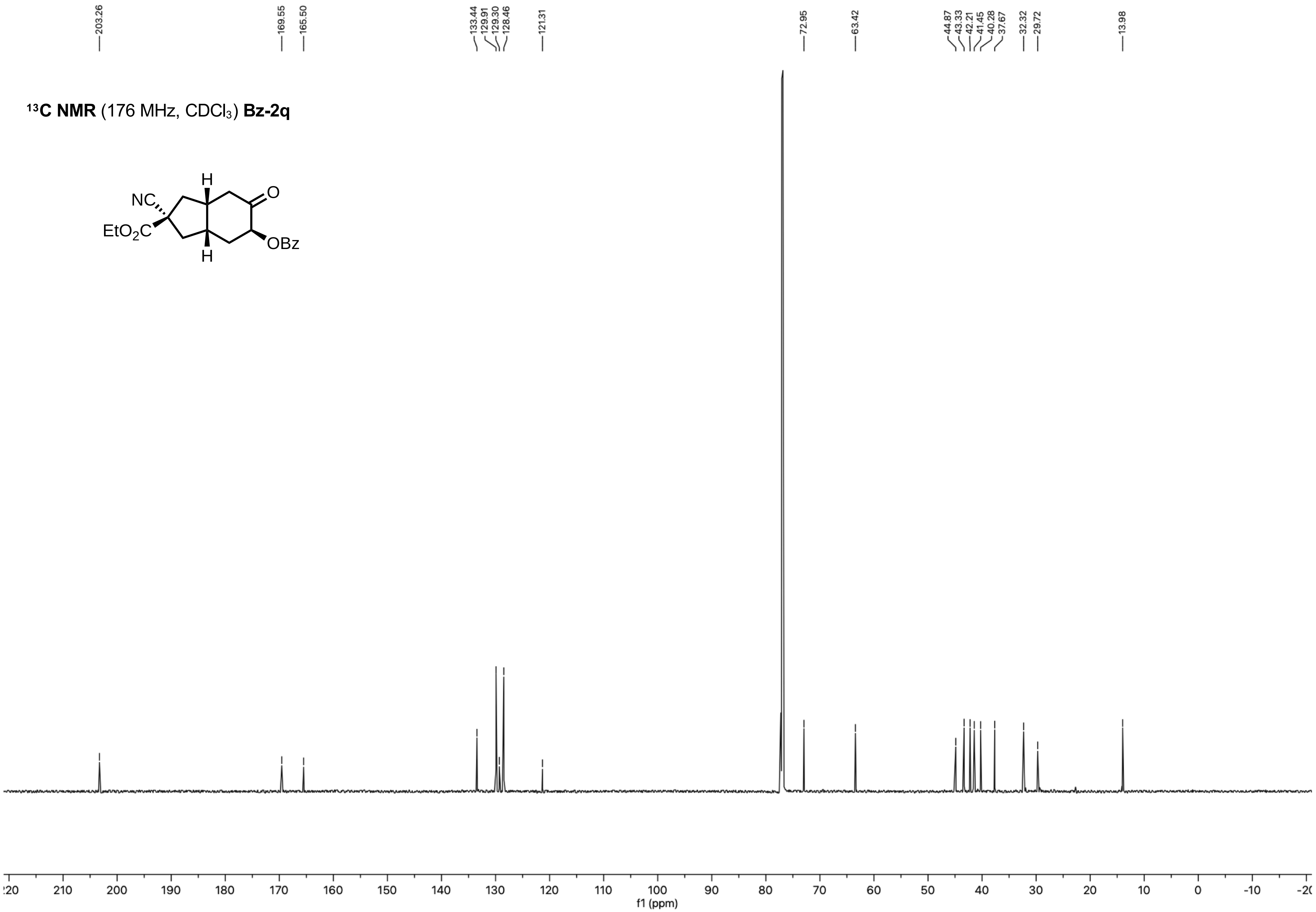

203.26
169.55
165.50
133.44
129.91
129.30
128.46
121.31
72.95
63.42
44.87
43.33
42.21
41.45
40.28
37.67
32.32
29.72
13.98
13C NMR (176 MHz, CDCl3) Bz-2q
NC
EtO2C
H
H
O
OBz
210
200
190
180
170
160
150
140
130
120
110
100
90
80
70
60
50
40
30
20
10
0
-10
f1 (ppm)

^{1}H NMR (500 MHz, $CDCl_3$) **Bz-2r**

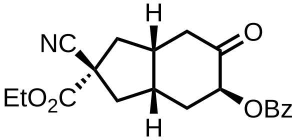

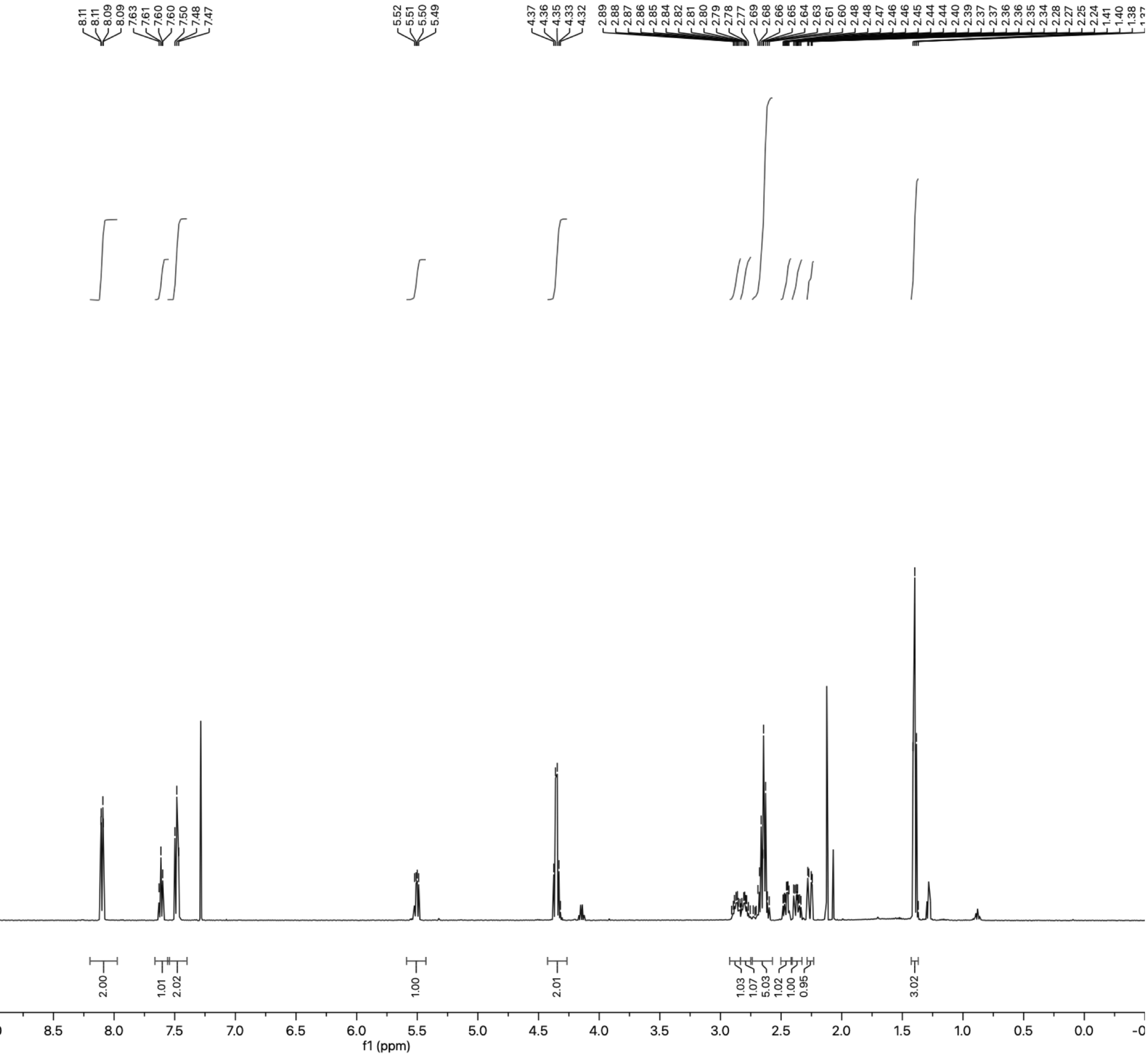

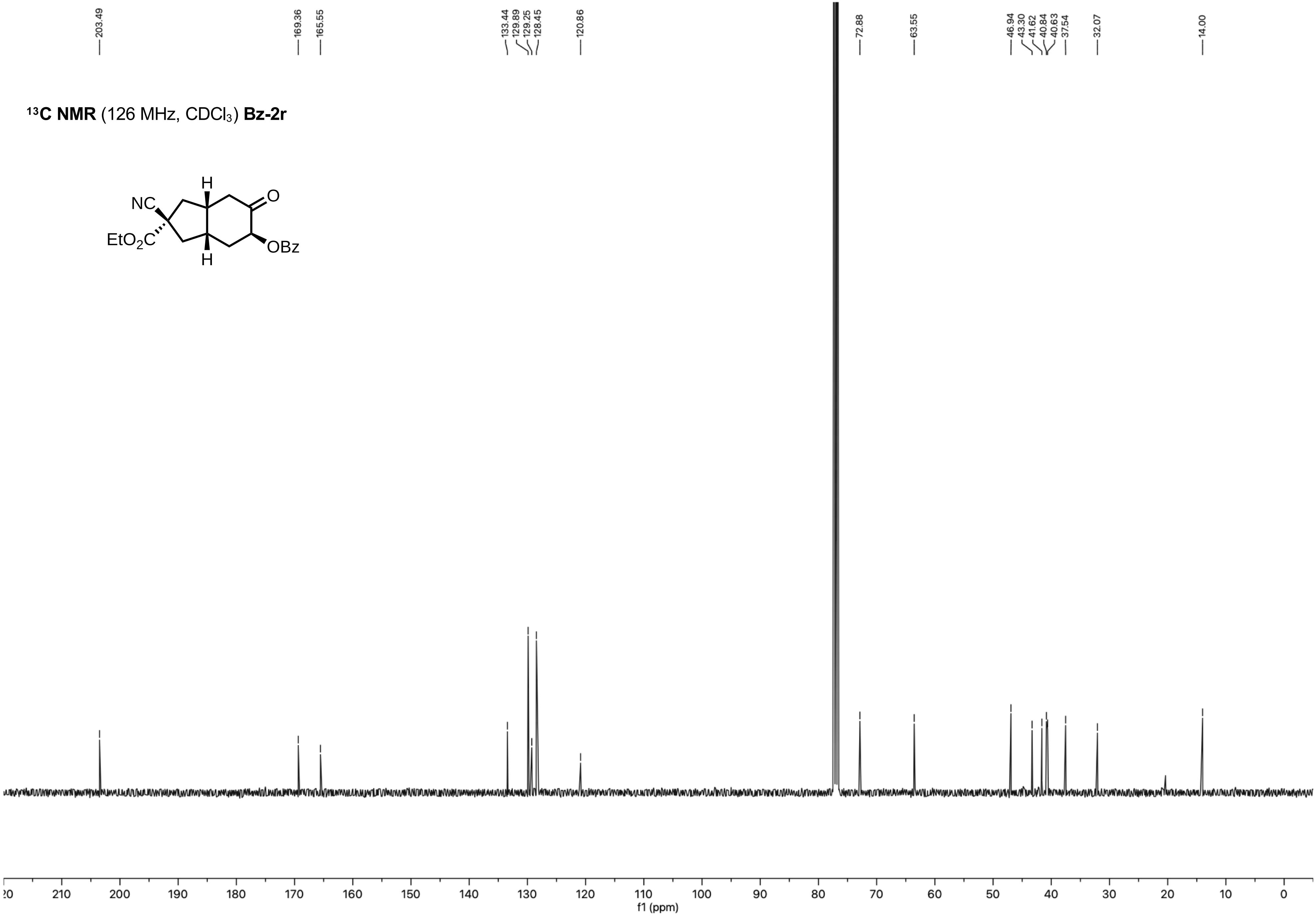

^{13}C NMR (126 MHz, $CDCl_3$) **Bz-2r**

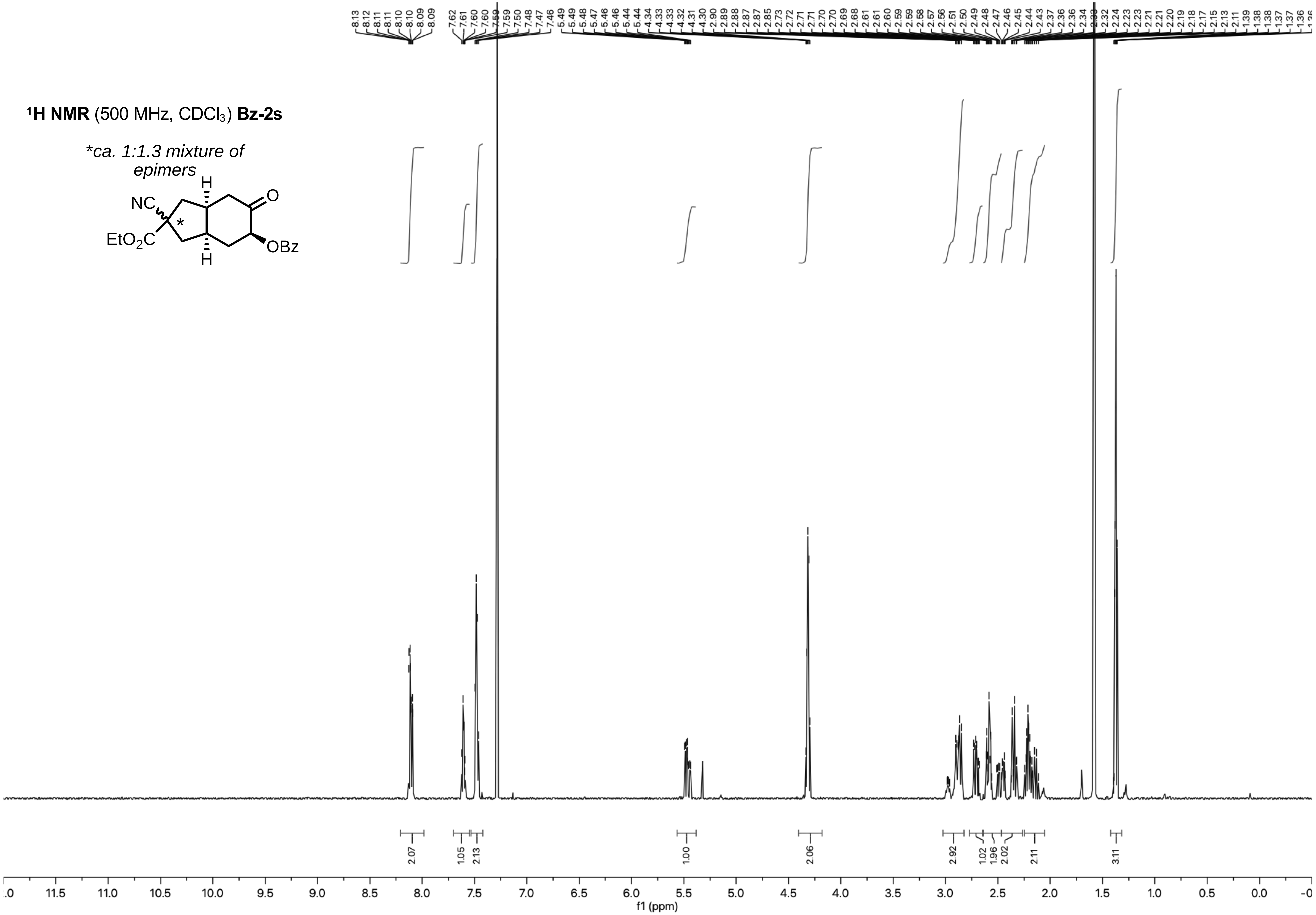

¹H NMR (500 MHz, CDCl₃) Bz-2s
*ca. 1:1.3 mixture of epimers
NC
EtO₂C
H
H
O
OBz
*
8.13 8.12 8.11 8.11 8.10 8.10 8.09 8.09
7.62 7.61 7.60 7.60 7.59 7.59 7.50 7.48 7.47 7.46
5.49 5.49 5.48 5.47 5.46 5.46 5.44 5.44 4.34 4.33 4.32 4.31 4.30
2.90 2.89 2.88 2.87 2.87 2.85 2.73 2.72 2.71 2.71 2.70 2.70 2.69 2.68 2.61 2.60 2.59 2.59 2.58 2.57 2.56 2.51 2.50 2.49 2.48 2.47 2.46 2.45 2.44 2.43 2.37 2.36 2.36 2.34 2.33 2.32 2.24 2.23 2.23 2.21 2.21 2.20 2.19 2.18 2.17 2.15 2.13 2.11 1.39 1.38 1.37 1.36
2.07
1.05
2.13
1.00
2.06
2.92
1.02
1.96
2.02
2.11
3.11
11.5 11.0 10.5 10.0 9.5 9.0 8.5 8.0 7.5 7.0 6.5 6.0 5.5 5.0 4.5 4.0 3.5 3.0 2.5 2.0 1.5 1.0 0.5 0.0
f1 (ppm)

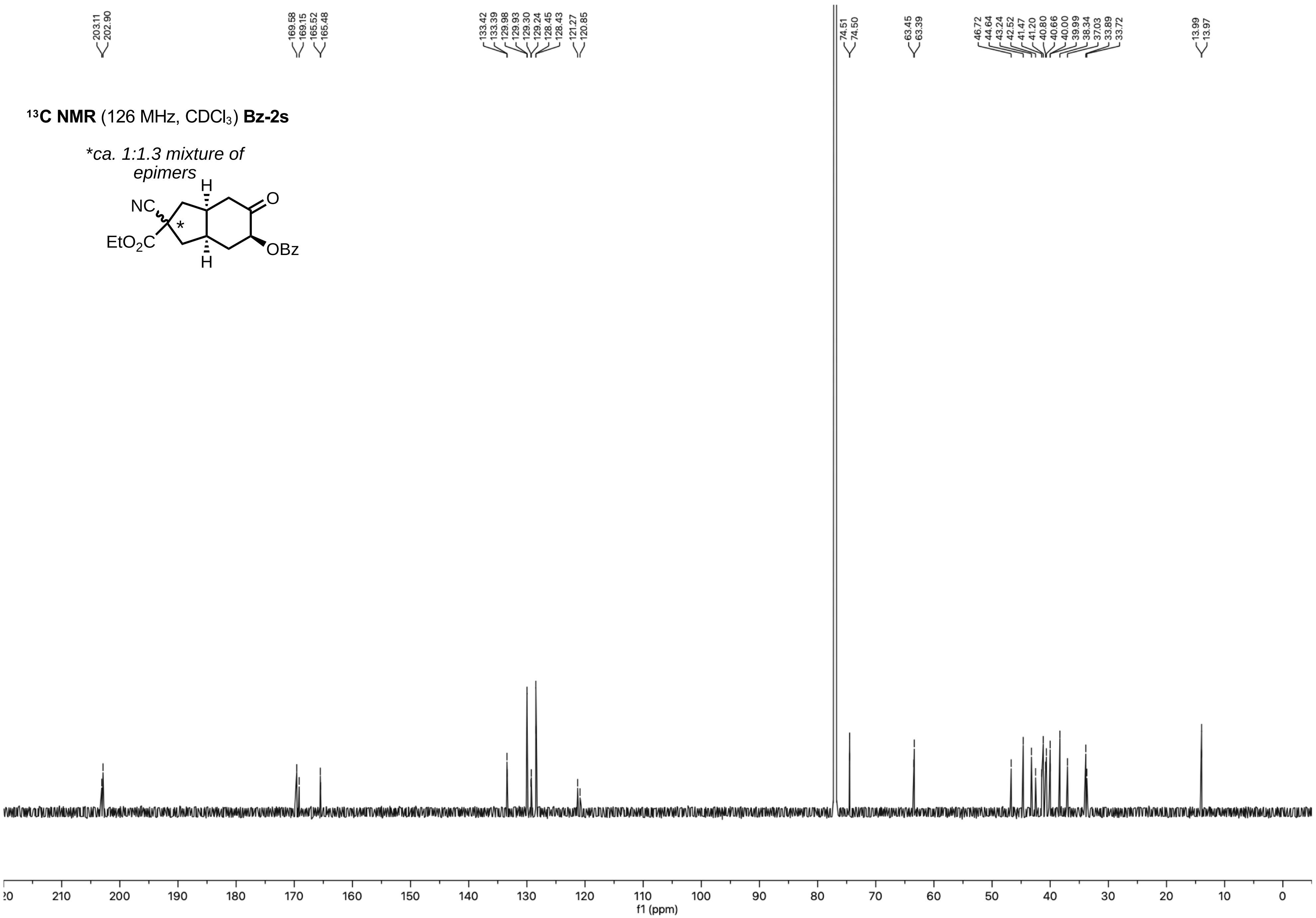

203.11
202.90
169.58
169.15
165.52
165.48
133.42
133.39
129.98
129.93
129.30
129.24
128.45
128.43
121.27
120.85
74.51
74.50
63.45
63.39
46.72
44.64
43.24
42.52
41.47
41.20
40.80
40.66
40.00
39.99
38.34
37.03
33.89
33.72
13.99
13.97
¹³C NMR (126 MHz, CDCl₃) Bz-2s
*ca. 1:1.3 mixture of epimers
NC
EtO₂C
H
H
O
OBz
*
210
200
190
180
170
160
150
140
130
120
110
100
90
80
70
60
50
40
30
20
10
0
f1 (ppm)

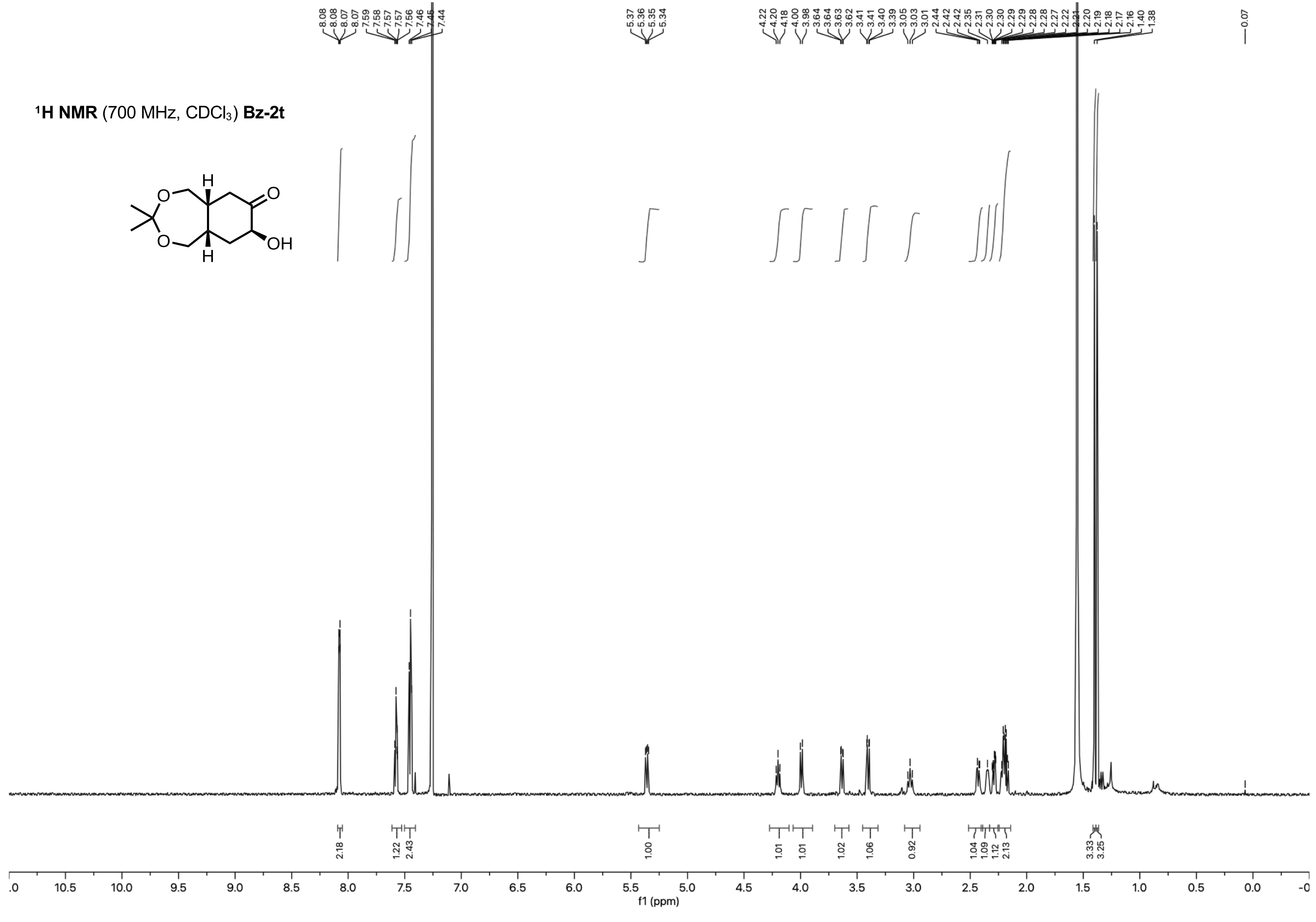
¹H NMR (700 MHz, CDCl3) Bz-2t
f1 (ppm)

^{13}C NMR (176 MHz, $CDCl_3$) **Bz-2t**

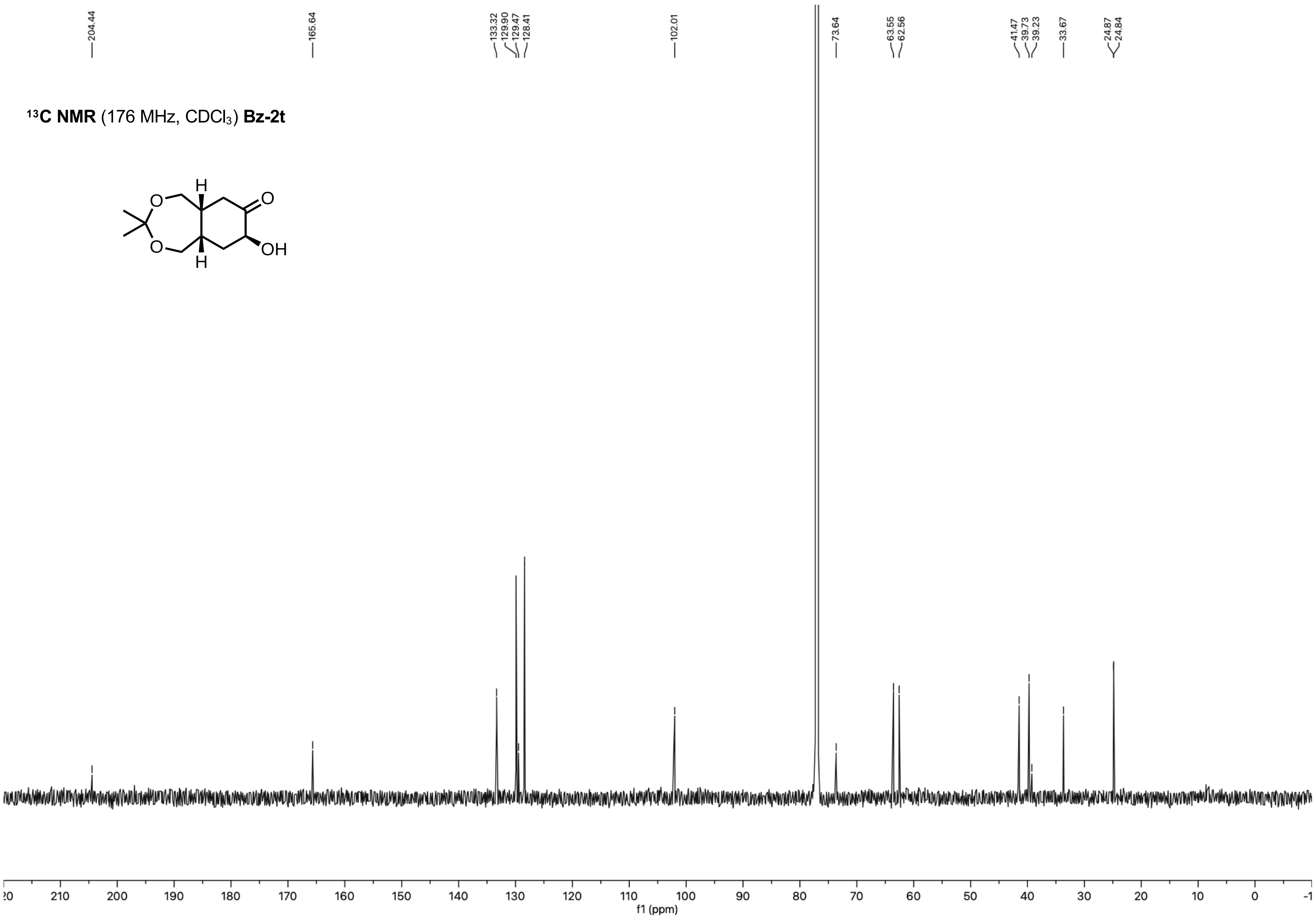

¹H NMR (700 MHz, $CDCl_3$) **Bz-2u**

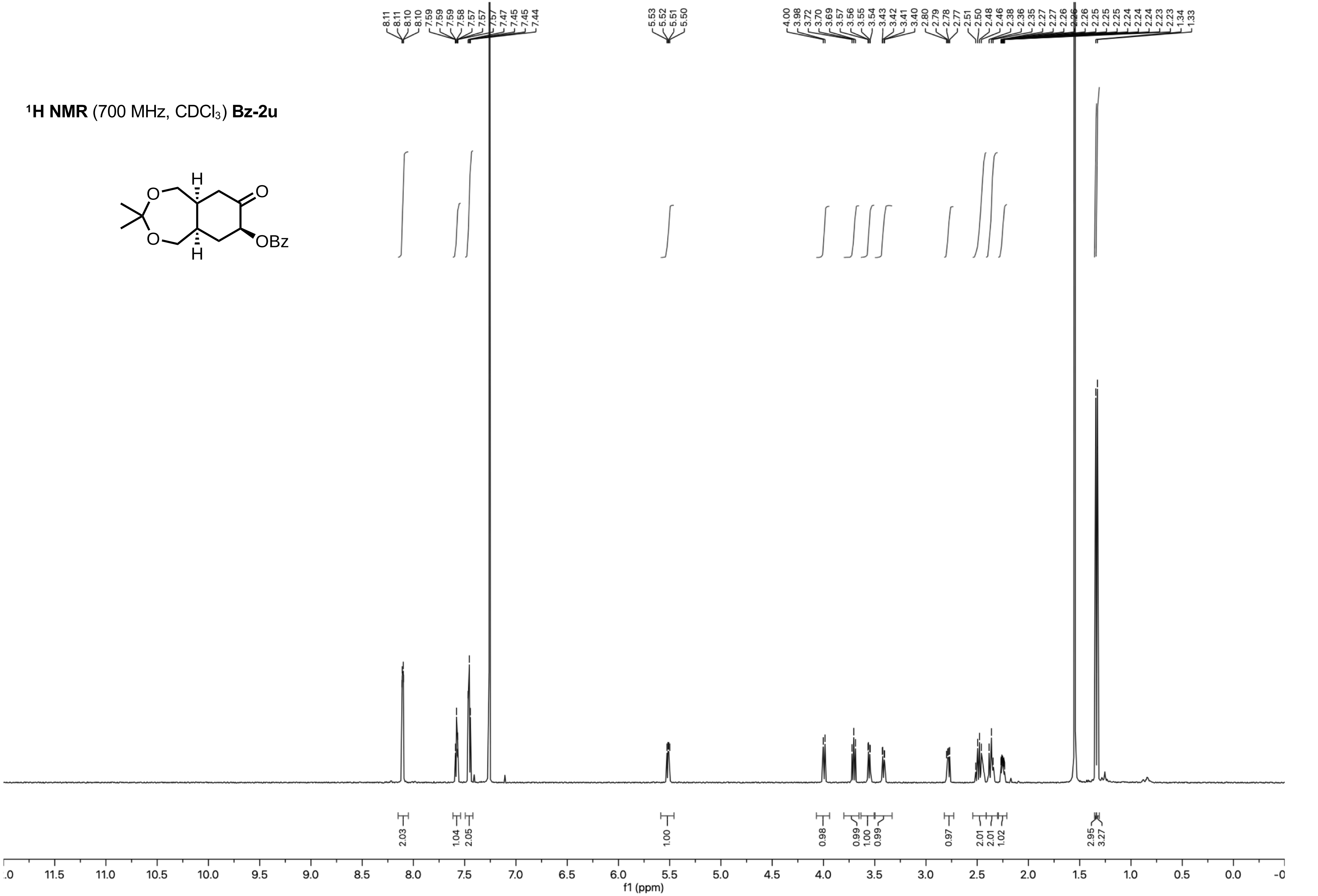

^{13}**C NMR** (176 MHz, $CDCl_3$) **Bz-2u**

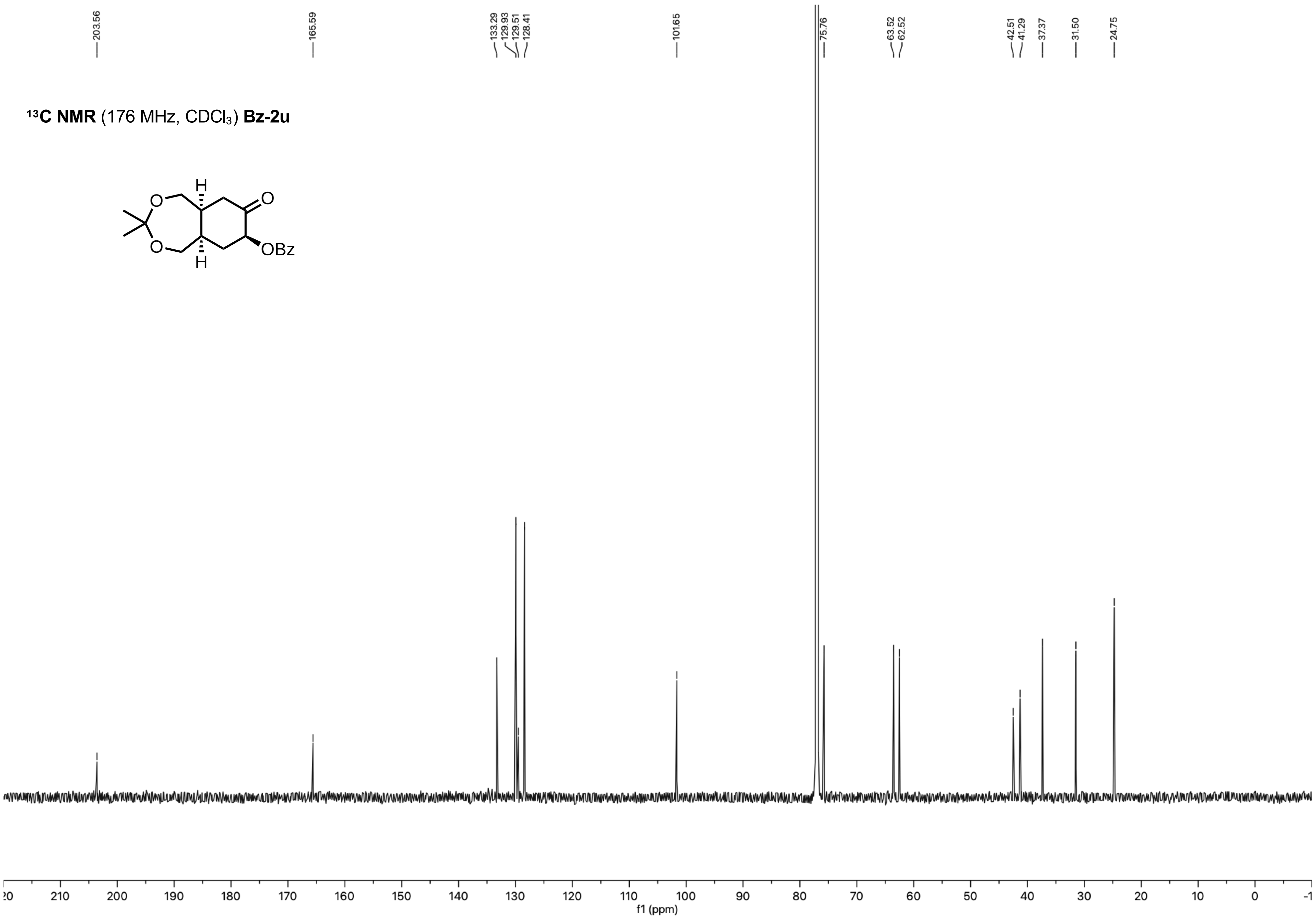

^{1}H NMR (700 MHz, $CDCl_3$) **Bz-2v**

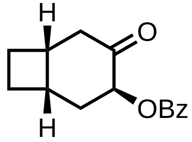

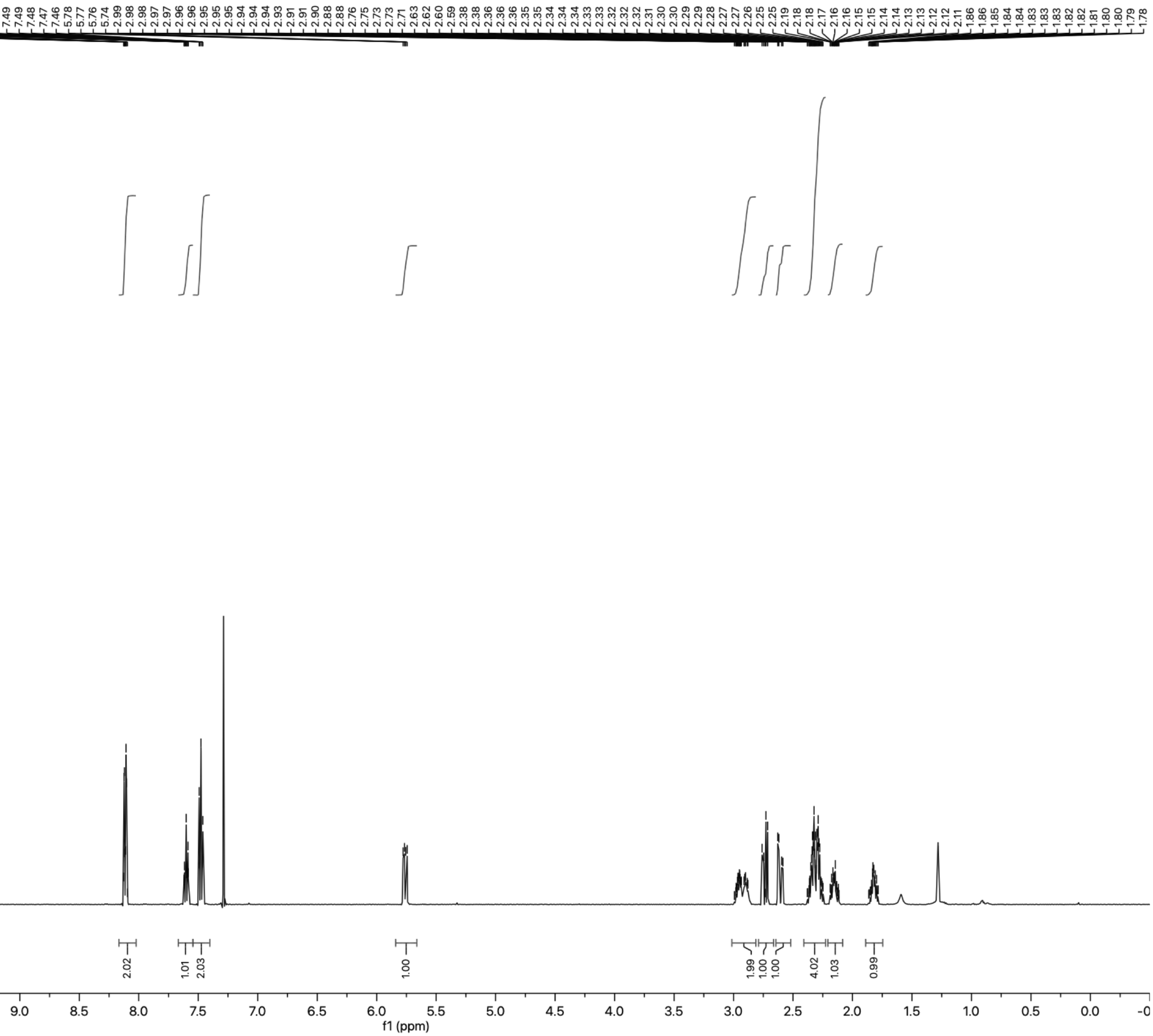

^{13}**C NMR** (176 MHz, $CDCl_3$) **Bz-2v**

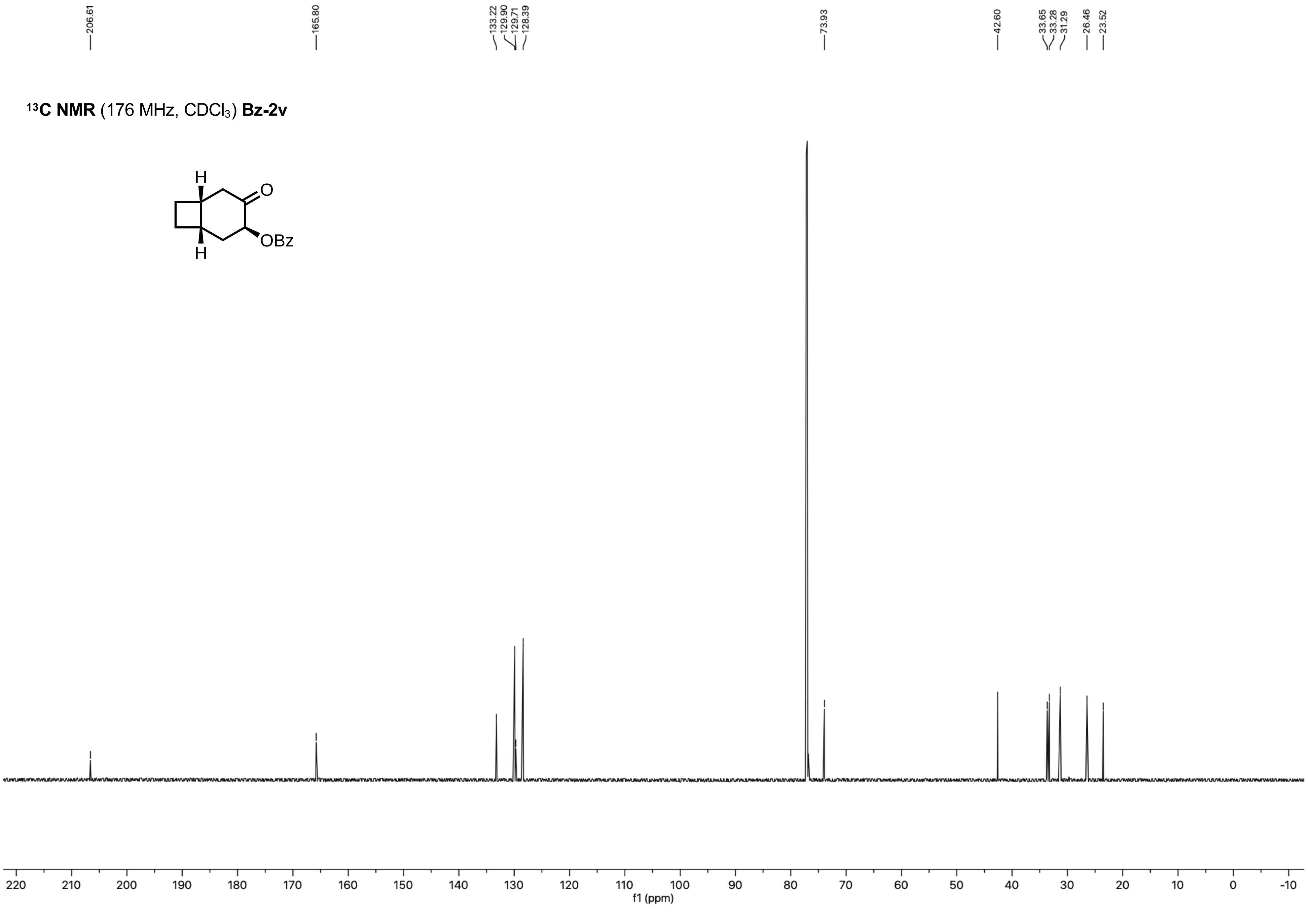

^{1}H NMR (700 MHz, $CDCl_3$) **Bz-2x**

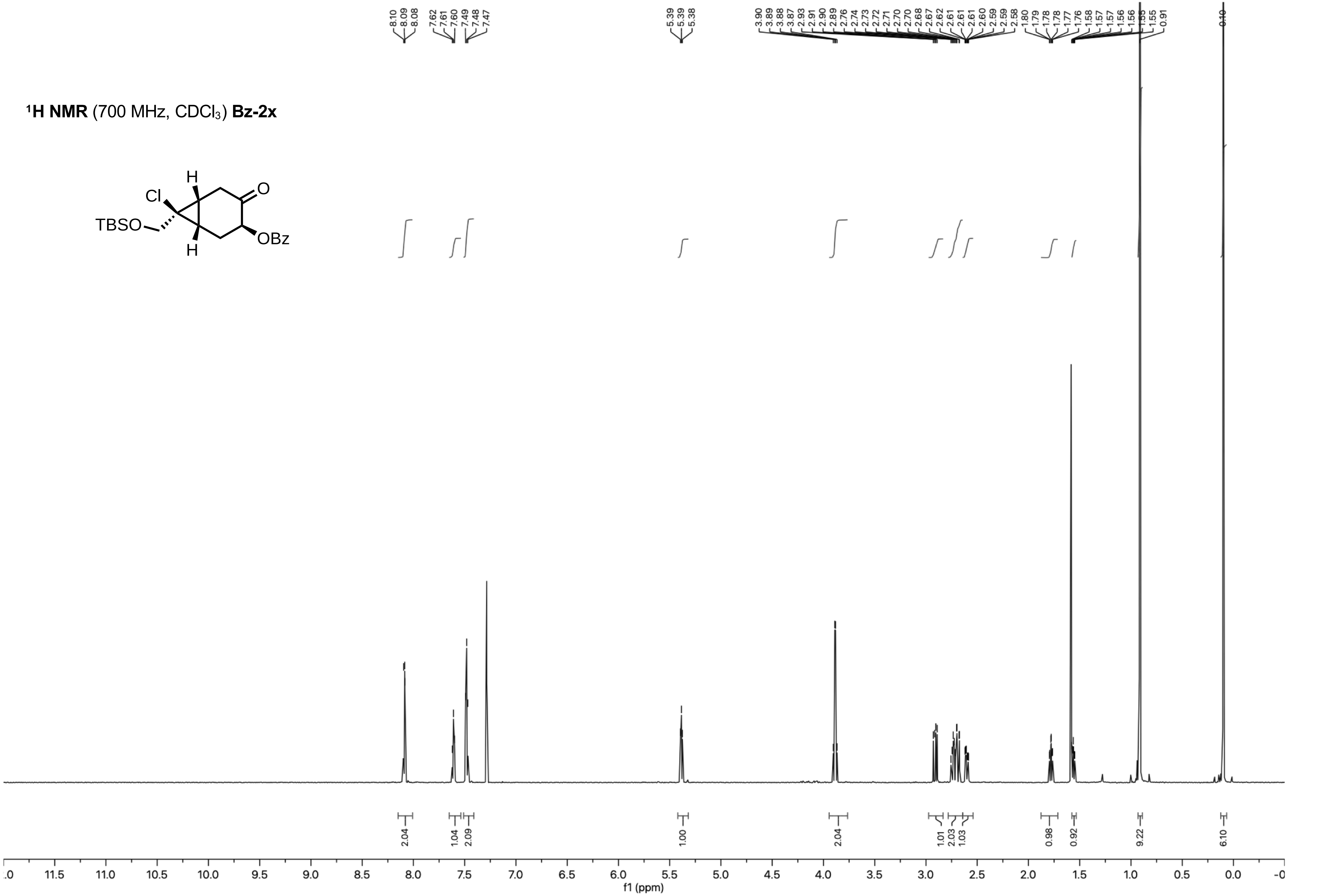

^{13}C NMR (176 MHz, $CDCl_3$) **Bz-2x**

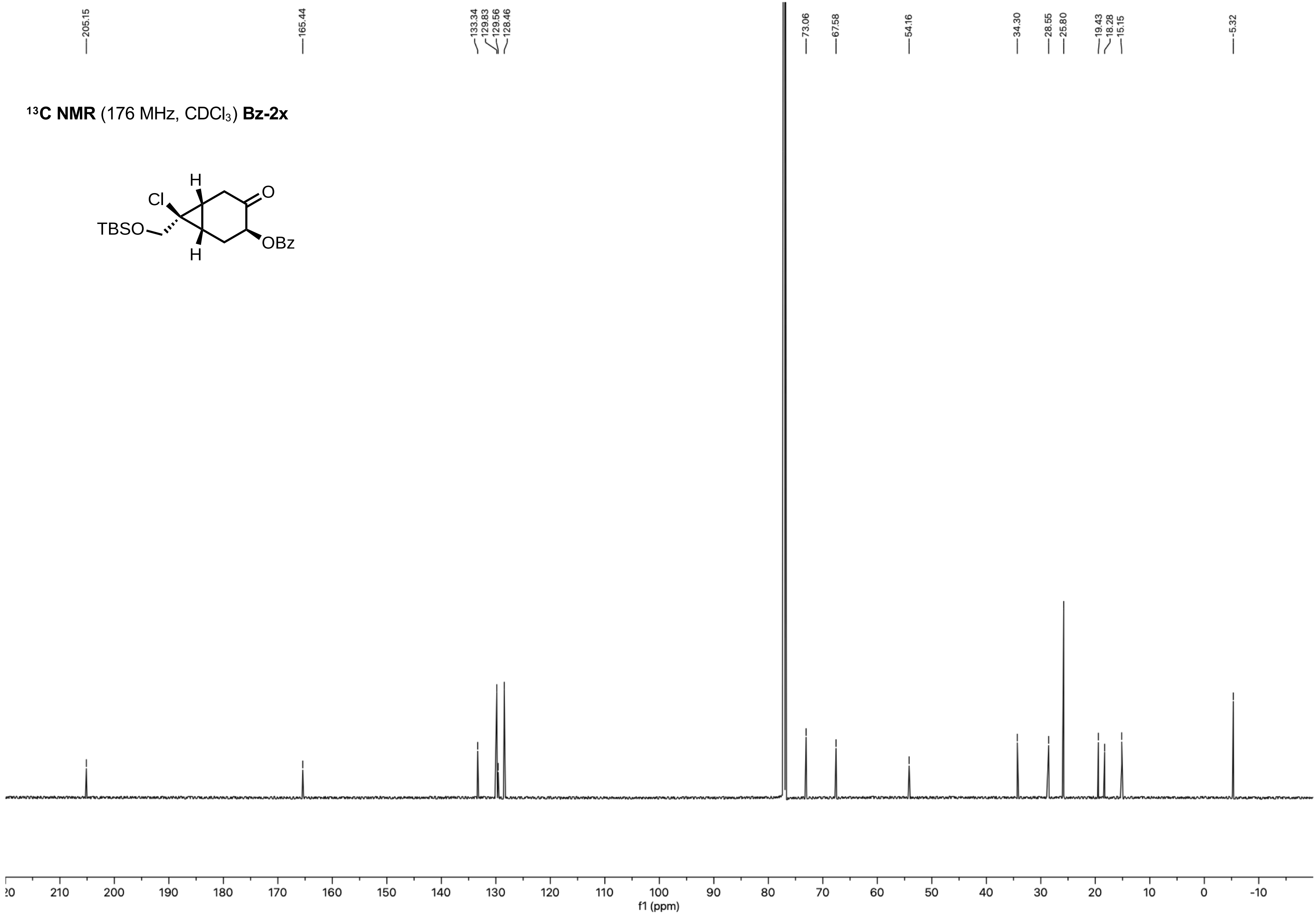

^{1}H NMR (700 MHz, $CDCl_3$) **Bz-2y**

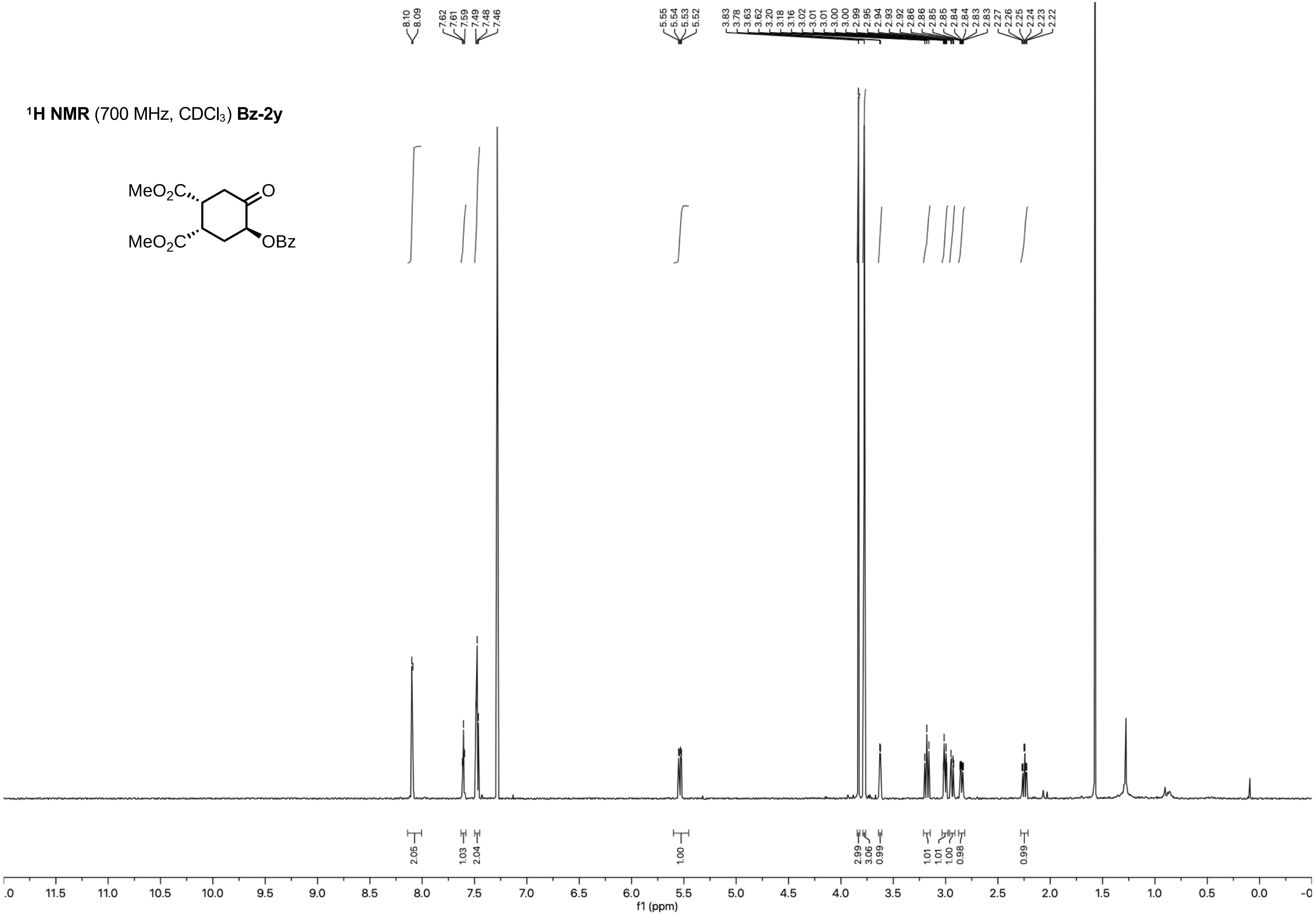

^{13}C NMR (176 MHz, $CDCl_3$) **Bz-2y**

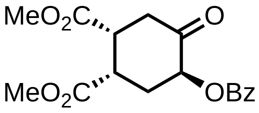

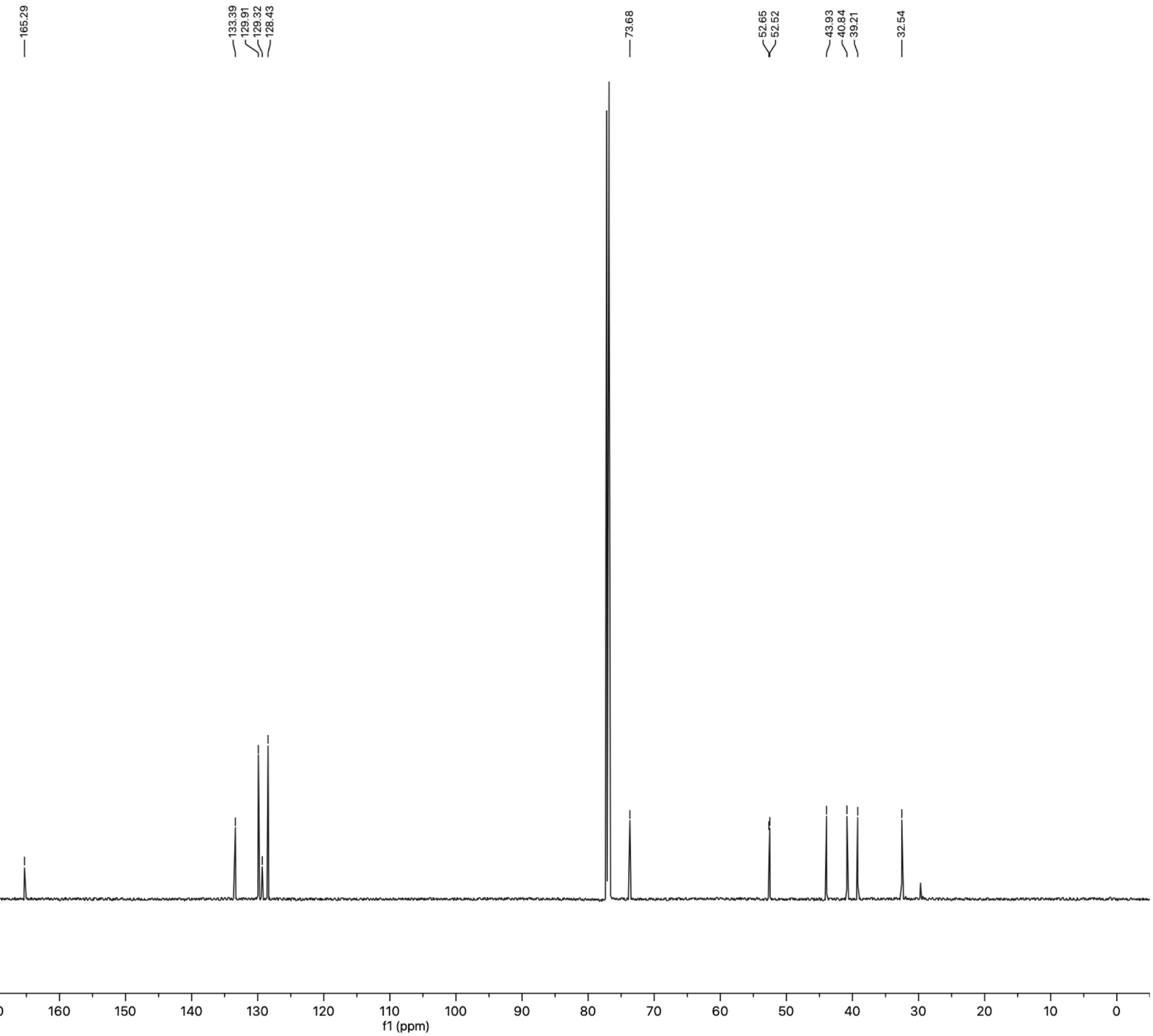

¹H NMR (700 MHz, $CDCl_3$) **Bz-2z**

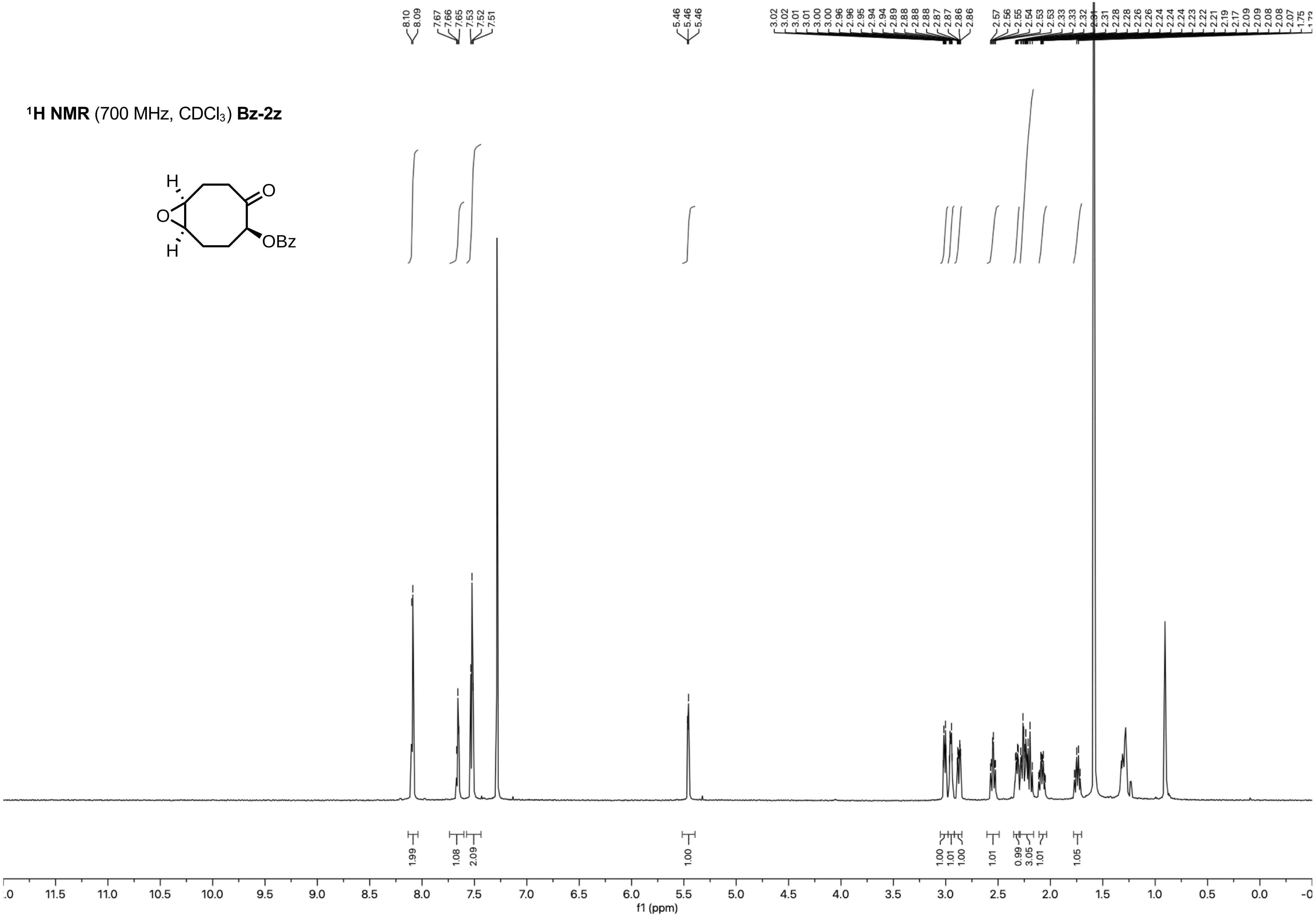

^{13}C NMR (176 MHz, $CDCl_3$) **Bz-2z**

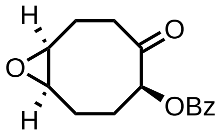

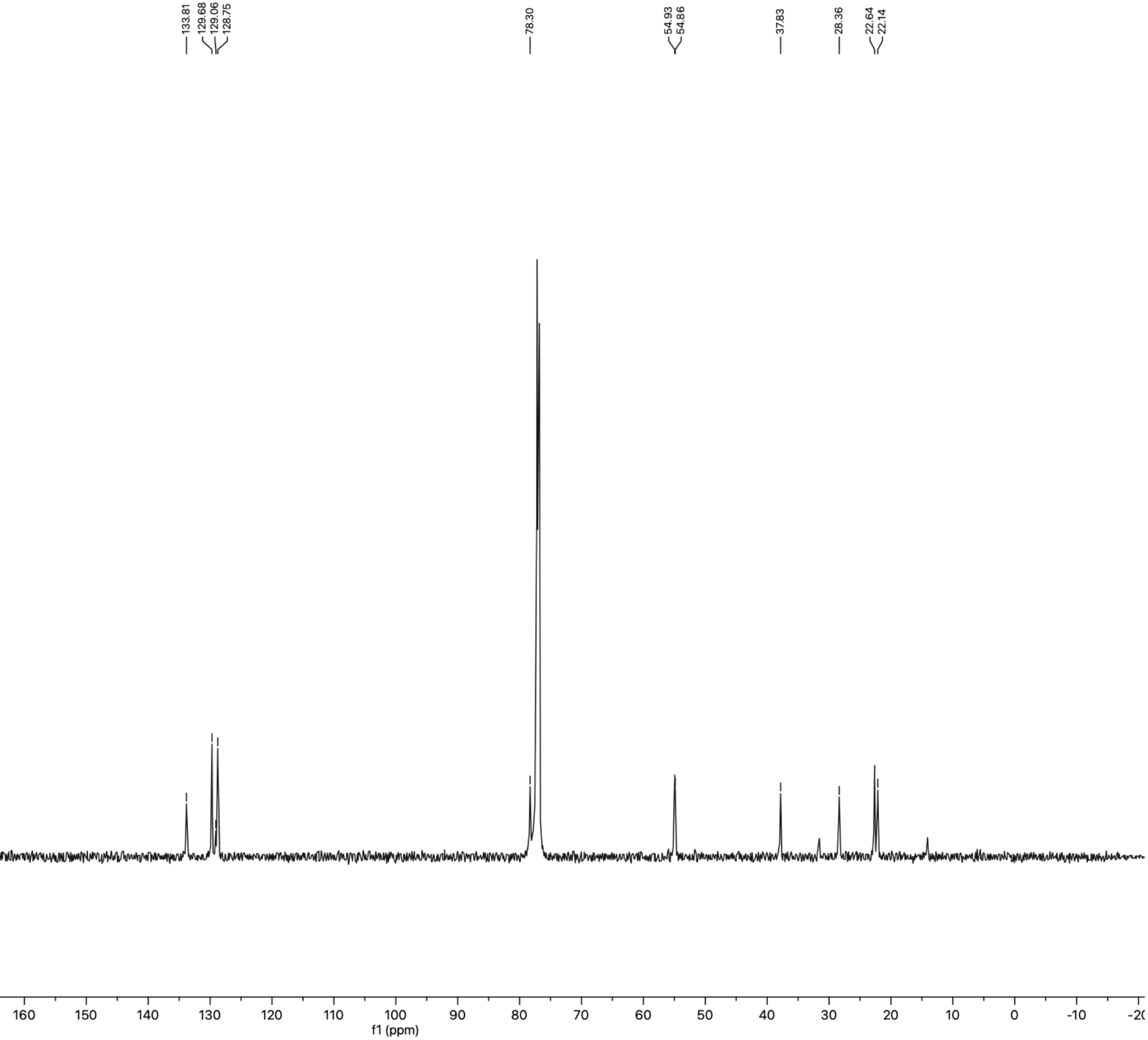

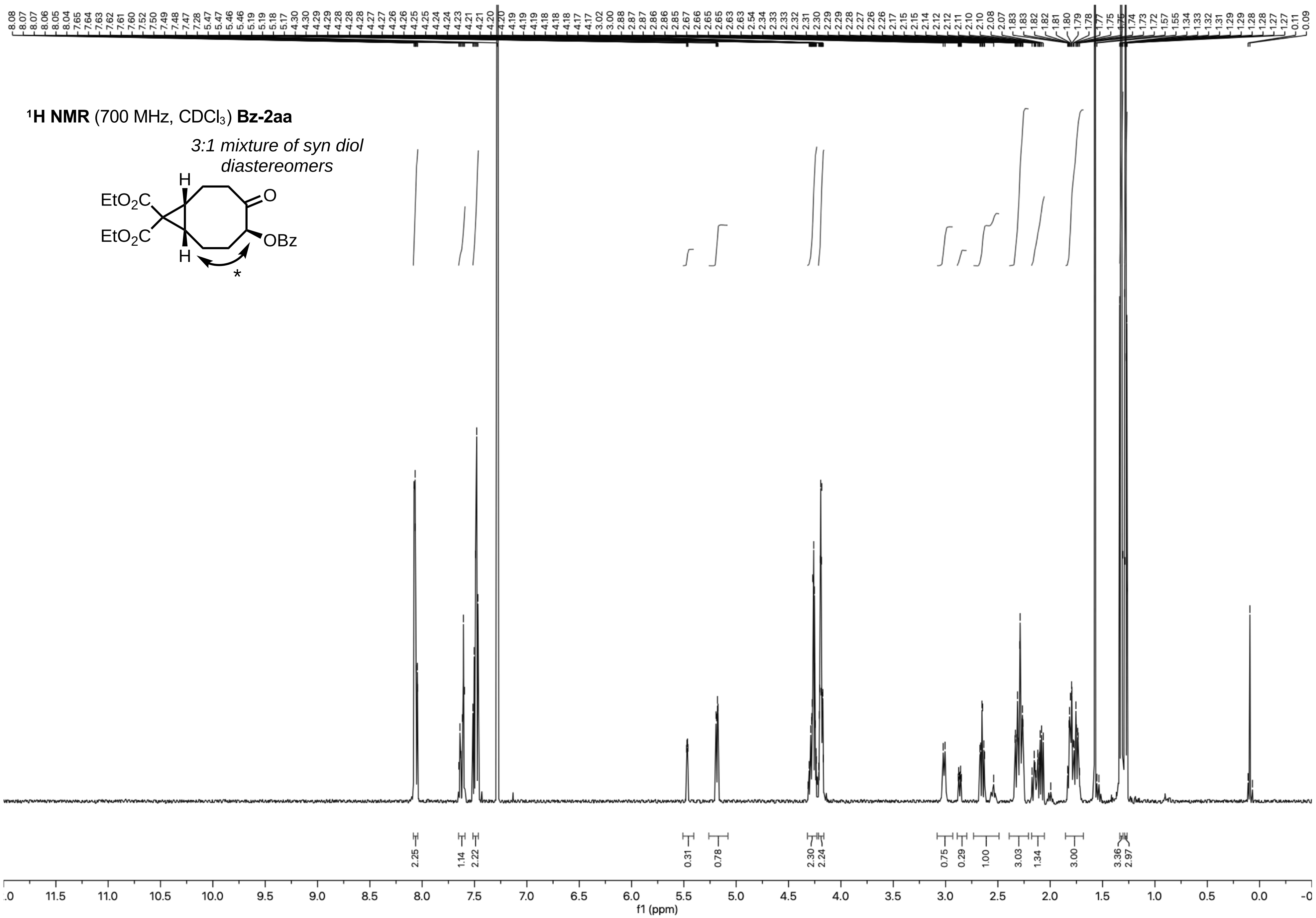

¹H NMR (700 MHz, CDCl3) Bz-2aa
3:1 mixture of syn diol diastereomers
H
EtO2C
O
EtO2C
OBz
H
*
8.08 8.07 8.07 8.06 8.05 8.04 7.65 7.64 7.63 7.62 7.61 7.60 7.52 7.50 7.49 7.48 7.47 7.28 5.47 5.47 5.46 5.19 5.19 5.18 5.17 4.30 4.30 4.29 4.29 4.28 4.28 4.28 4.27 4.26 4.26 4.25 4.25 4.24 4.24 4.23 4.21 4.21 4.20 4.20 4.19 4.19 4.18 4.18 4.18 4.17 4.17 3.02 3.00 2.88 2.87 2.87 2.86 2.86 2.85 2.67 2.66 2.65 2.65 2.63 2.63 2.54 2.34 2.33 2.33 2.32 2.31 2.30 2.29 2.29 2.28 2.27 2.26 2.26 2.17 2.15 2.15 2.14 2.12 2.12 2.11 2.10 2.10 2.08 2.07 1.83 1.83 1.82 1.82 1.81 1.80 1.79 1.78 1.77 1.76 1.75 1.74 1.73 1.72 1.57 1.55 1.34 1.33 1.32 1.31 1.29 1.29 1.28 1.28 1.27 0.11 0.09
2.25 1.14 2.22 0.31 0.78 2.30 2.24 0.75 0.29 1.00 3.03 1.34 3.00 3.36 2.97
11.5 11.0 10.5 10.0 9.5 9.0 8.5 8.0 7.5 7.0 6.5 6.0 5.5 5.0 4.5 4.0 3.5 3.0 2.5 2.0 1.5 1.0 0.5 0.0
f1 (ppm)

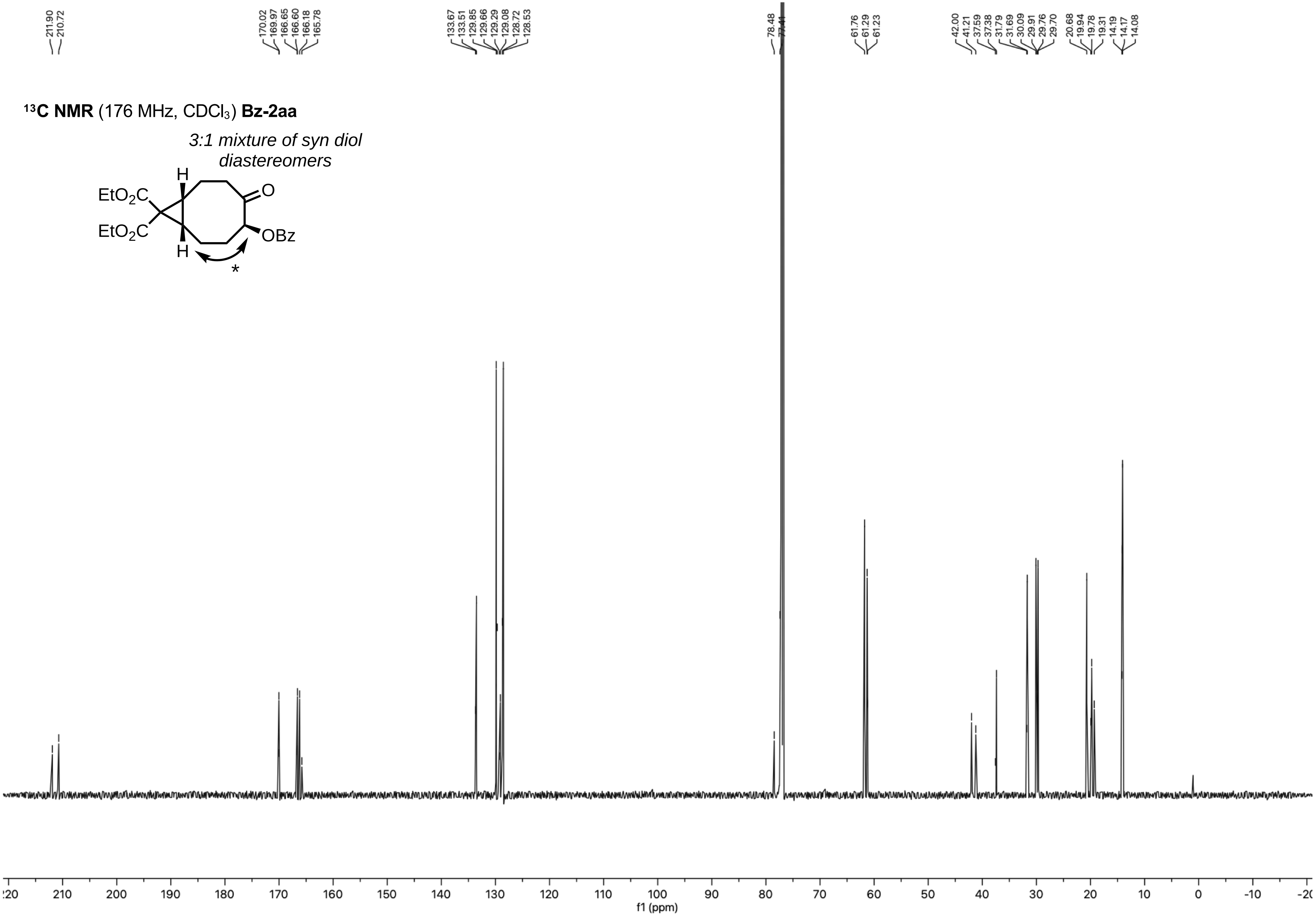
211.90
210.72
170.02
169.97
166.65
166.60
166.18
165.78
133.67
133.51
129.85
129.66
129.29
129.08
128.72
128.53
78.48
77.41
61.76
61.29
61.23
42.00
41.21
37.59
37.38
31.79
31.69
30.09
29.91
29.76
29.70
20.68
19.94
19.78
19.31
14.19
14.17
14.08
13C NMR (176 MHz, CDCl3) Bz-2aa
3:1 mixture of syn diol diastereomers
EtO2C
EtO2C
H
H
O
OBz
*
f1 (ppm)

¹H NMR (700 MHz, $CDCl_3$) **Bz-2ab**

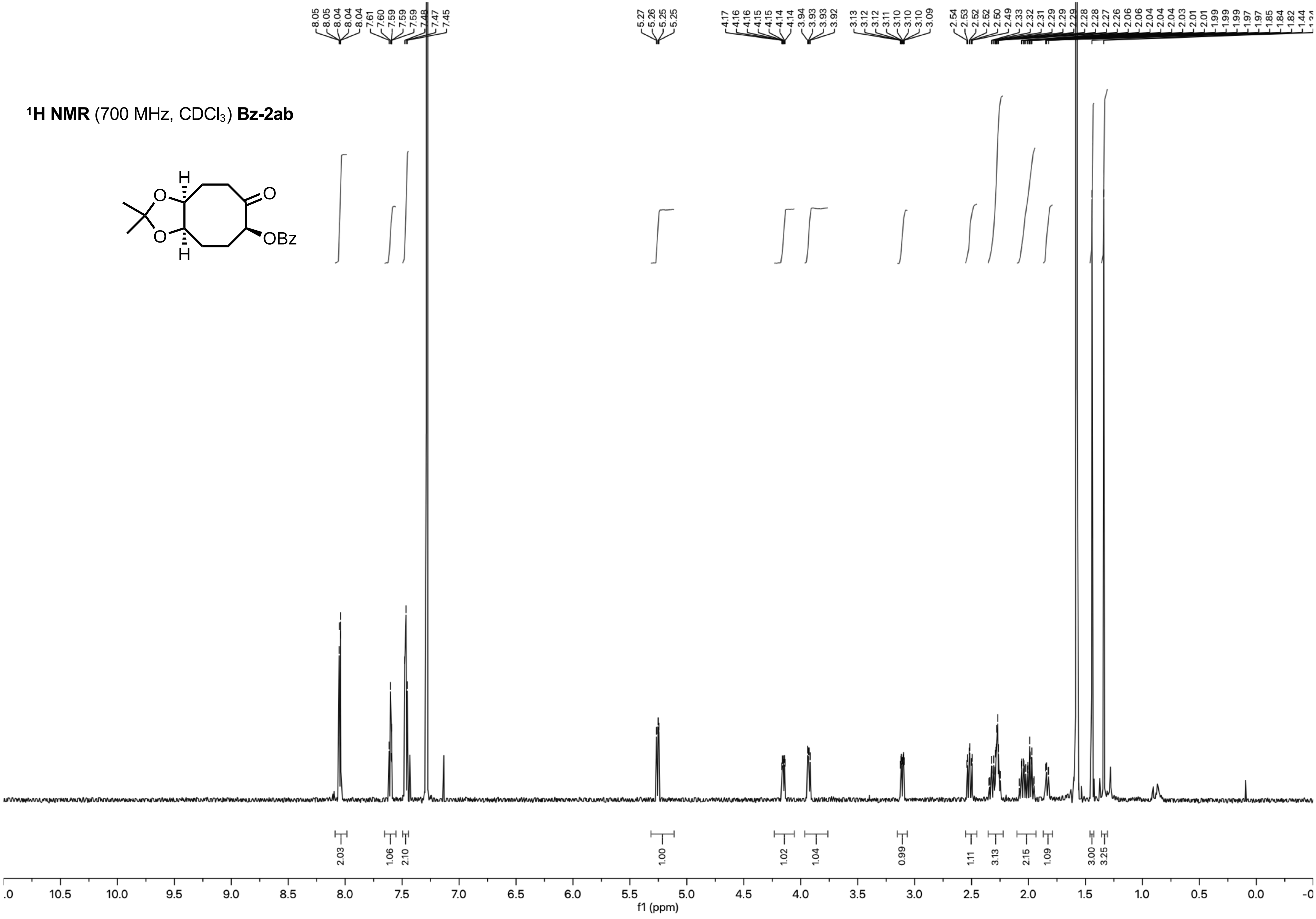

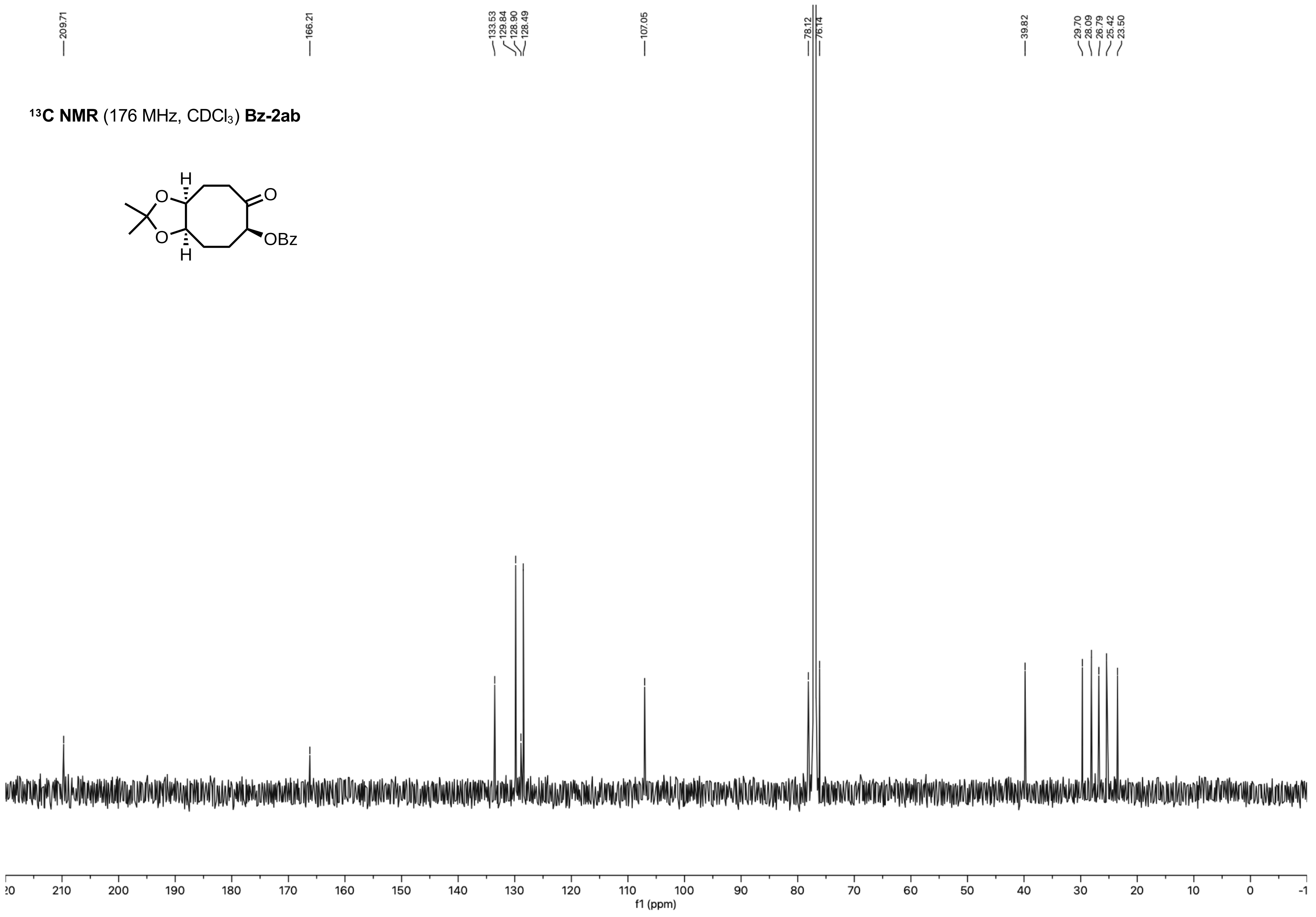
13C NMR (176 MHz, CDCl3) Bz-2ab
H
O
O
H
O
OBz
209.71
166.21
133.53
129.84
128.90
128.49
107.05
78.12
76.14
39.82
29.70
28.09
26.79
25.42
23.50
f1 (ppm)

¹H NMR (700 MHz, $CDCl_3$) **Bz-2ac**

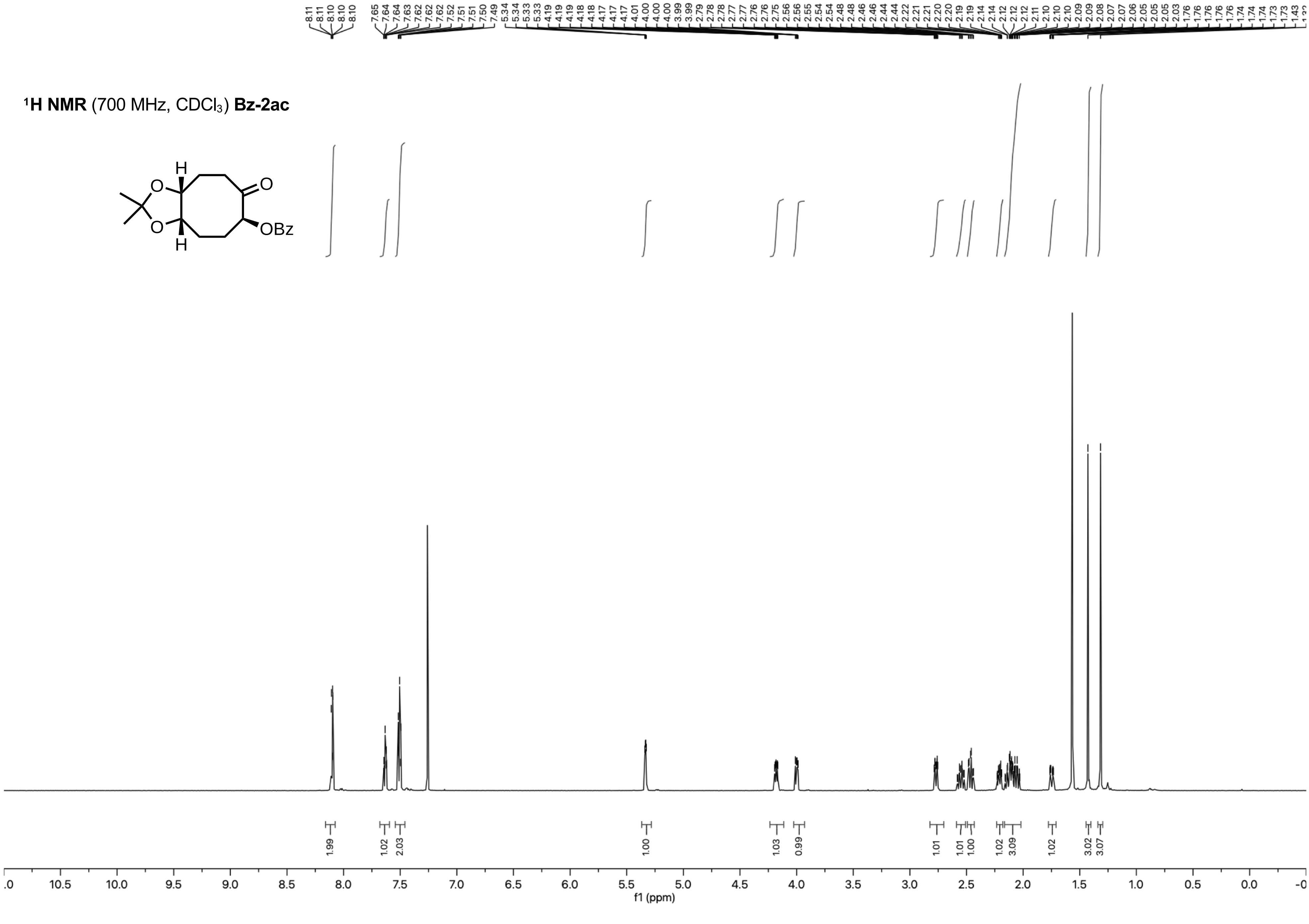

^{13}C NMR (176 MHz, $CDCl_3$) **Bz-2ac**

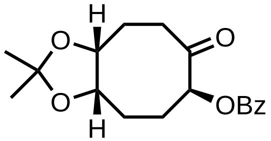

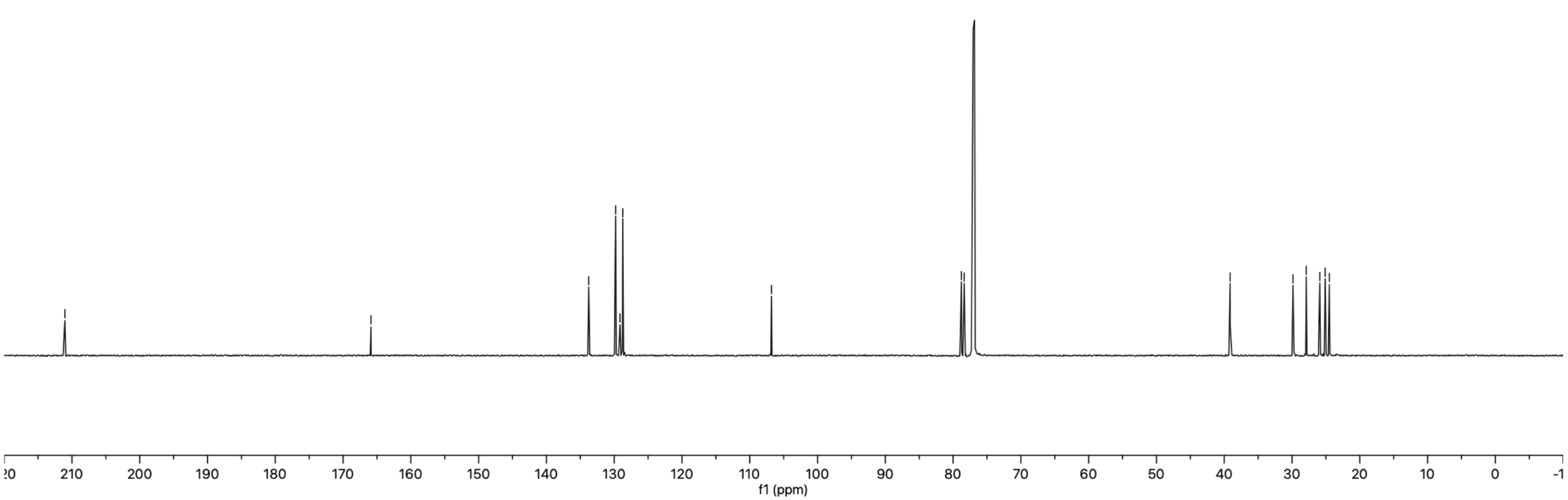

^{1}**H NMR** (700 MHz, $CDCl_3$) **Bz-4a**

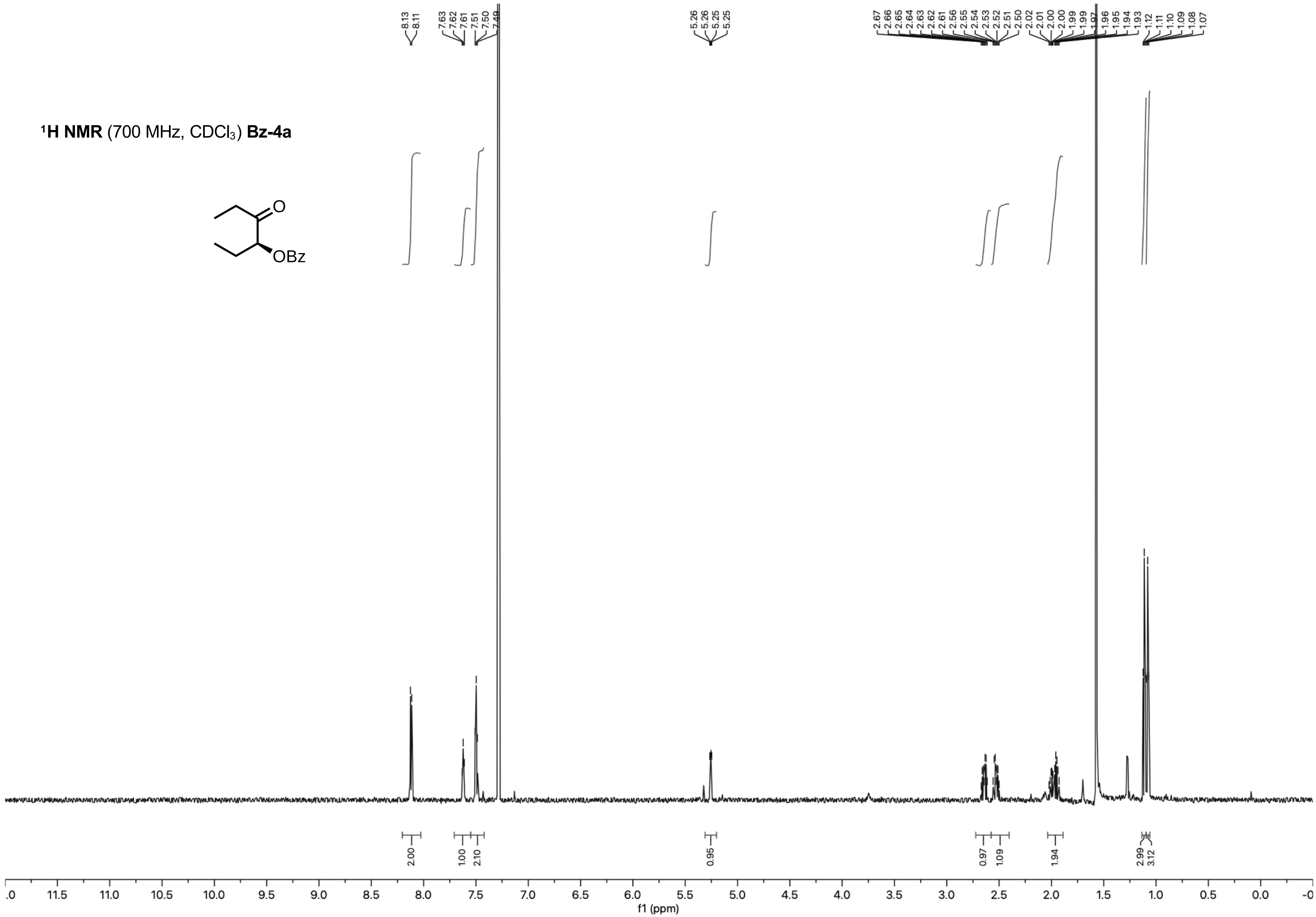

^{13}C NMR (176 MHz, $CDCl_3$) **Bz-4a**

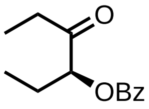

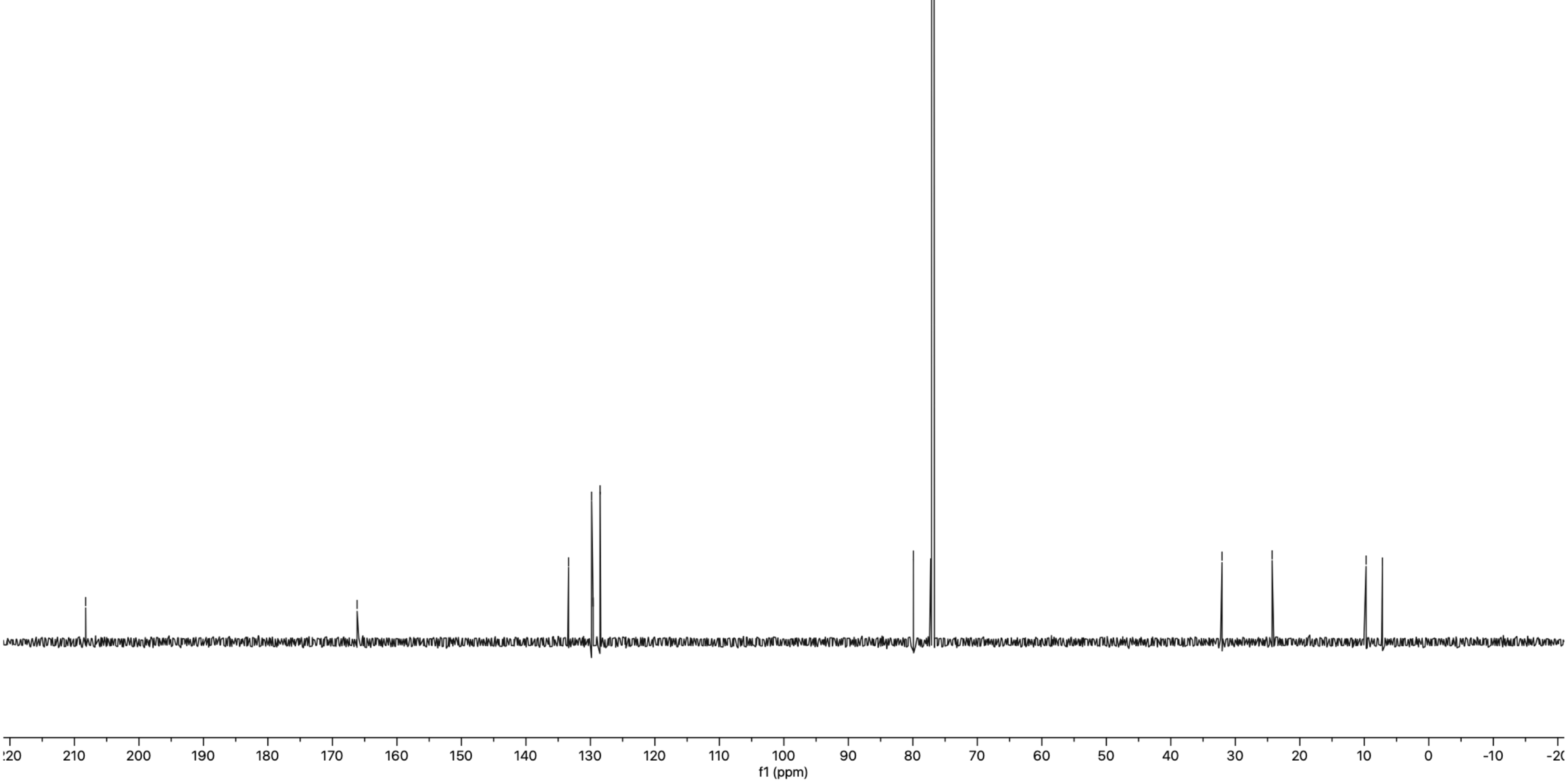

¹H NMR (700 MHz, $CDCl_3$) **Bz-4b**

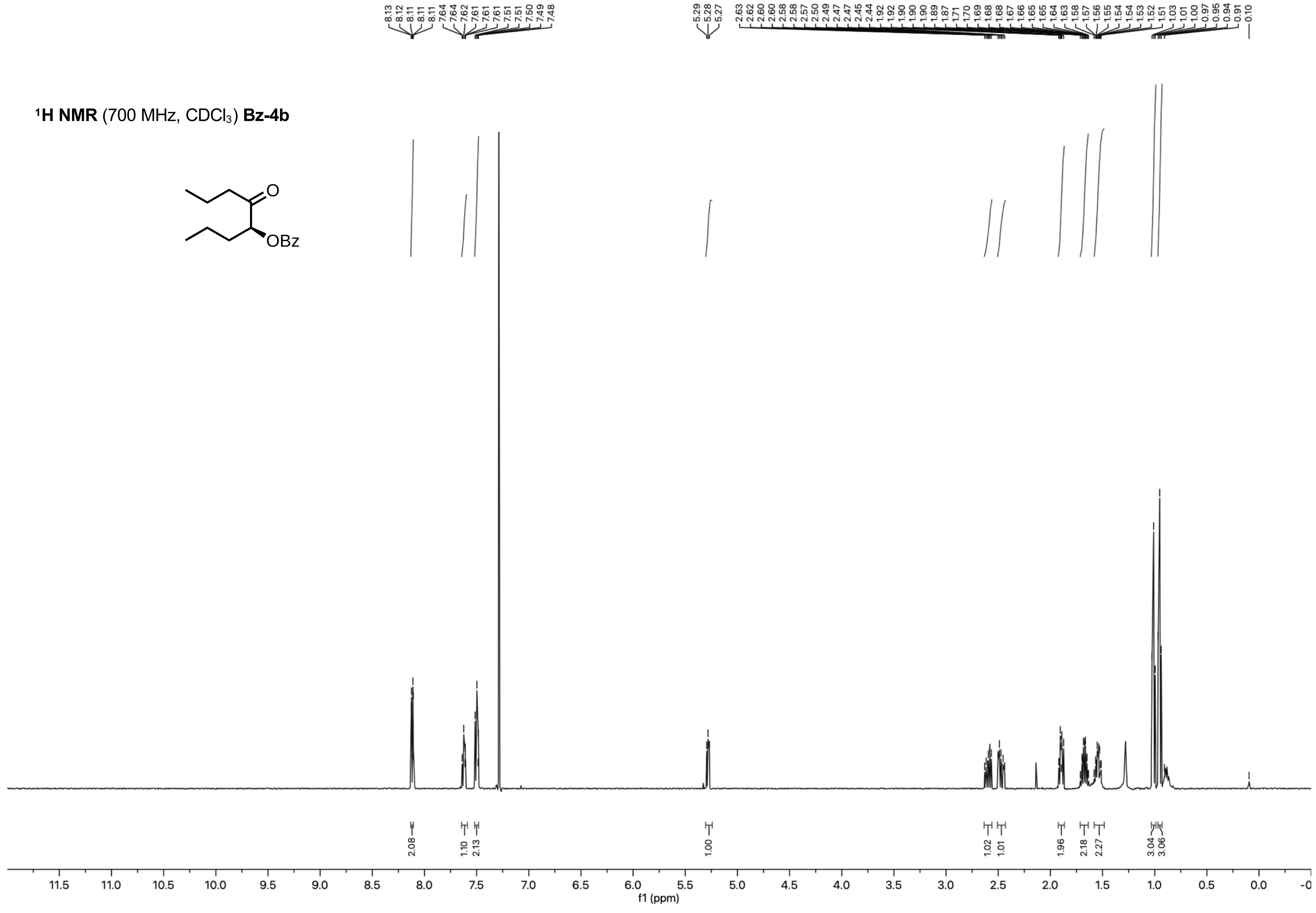

^{13}C NMR (176 MHz, $CDCl_3$) **Bz-4b**

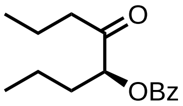

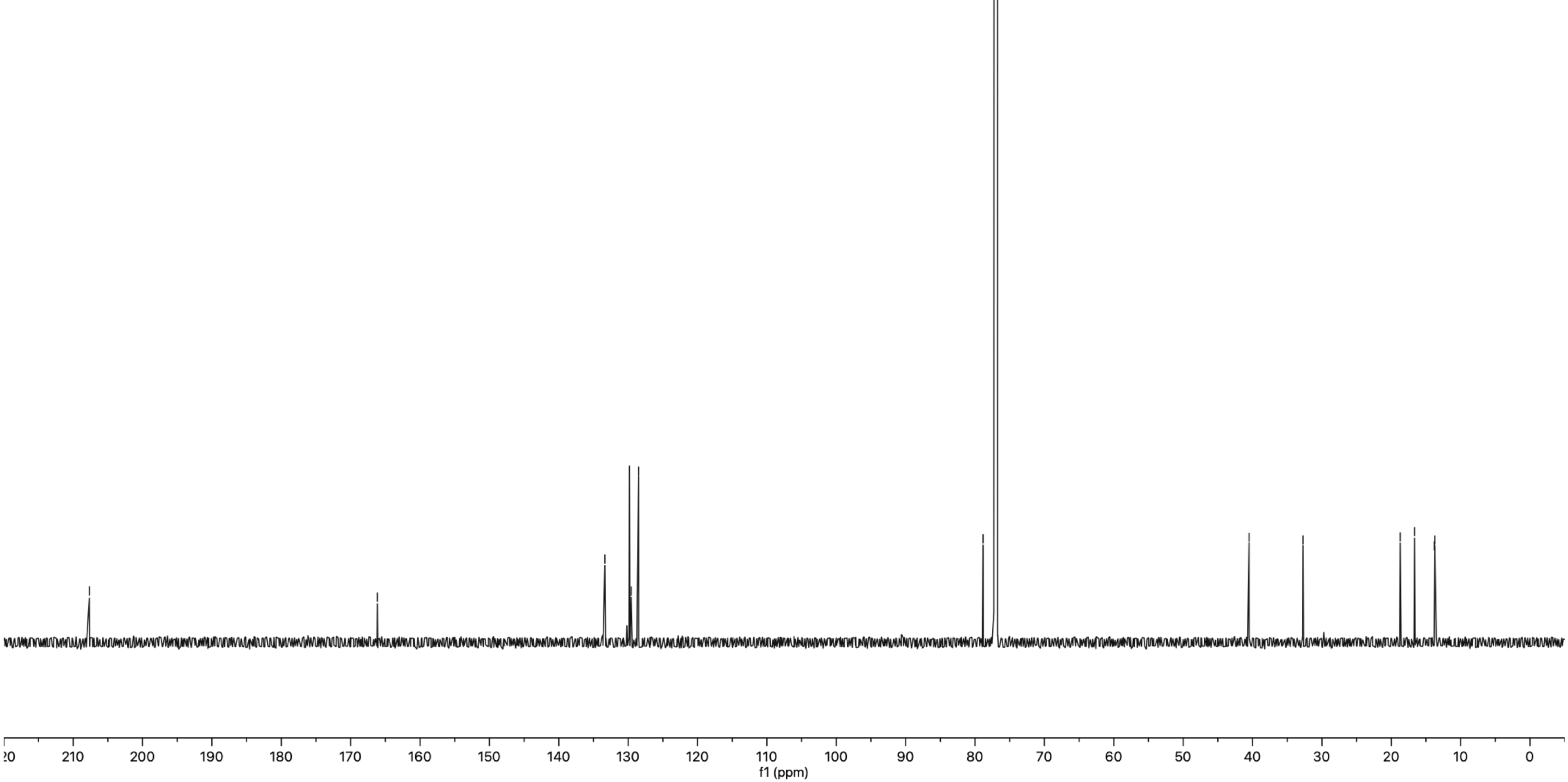

^{1}H NMR (700 MHz, $CDCl_3$) **Bz-4c**

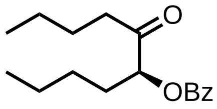

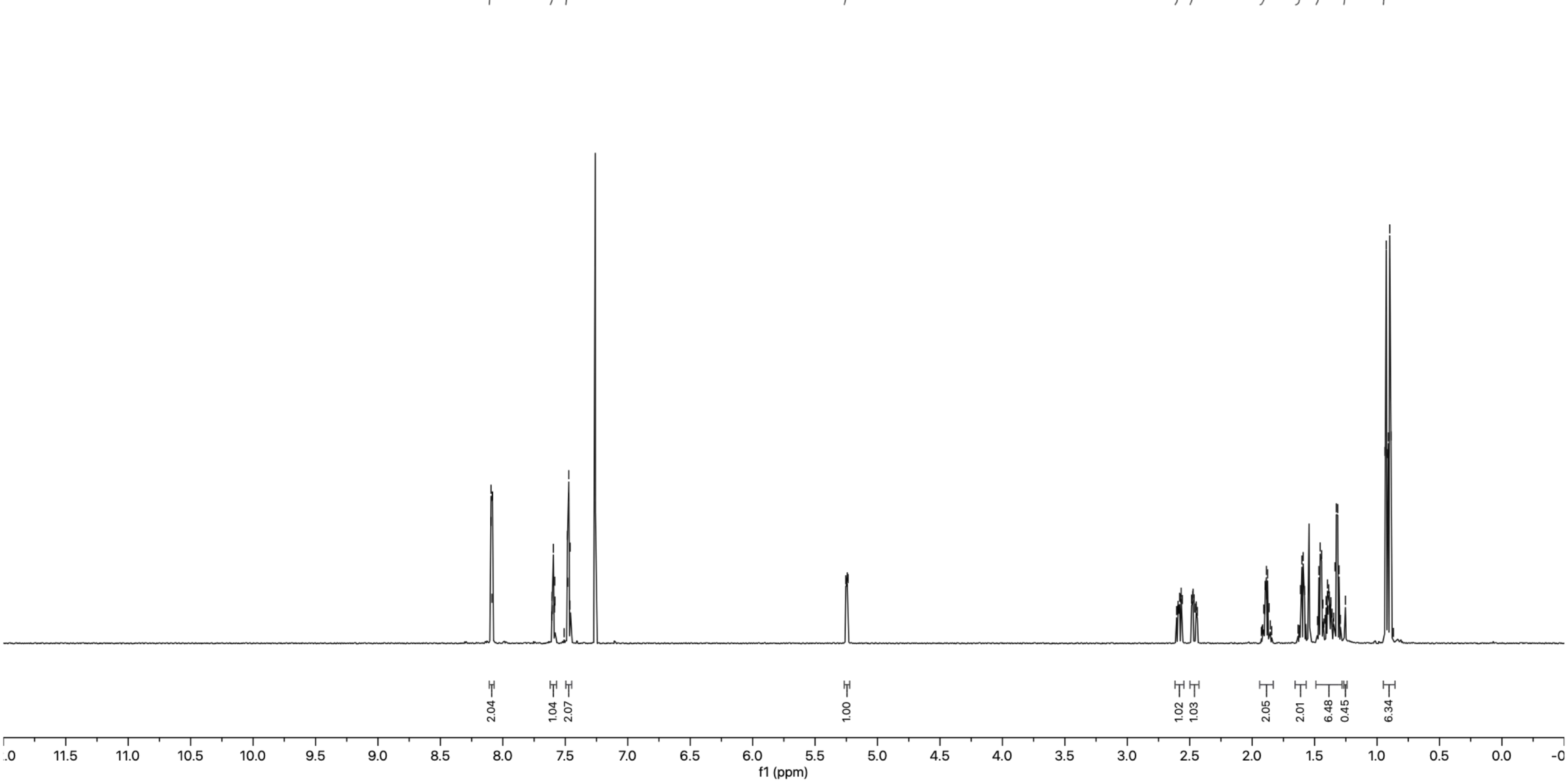

^{13}C NMR (176 MHz, $CDCl_3$) **Bz-4c**

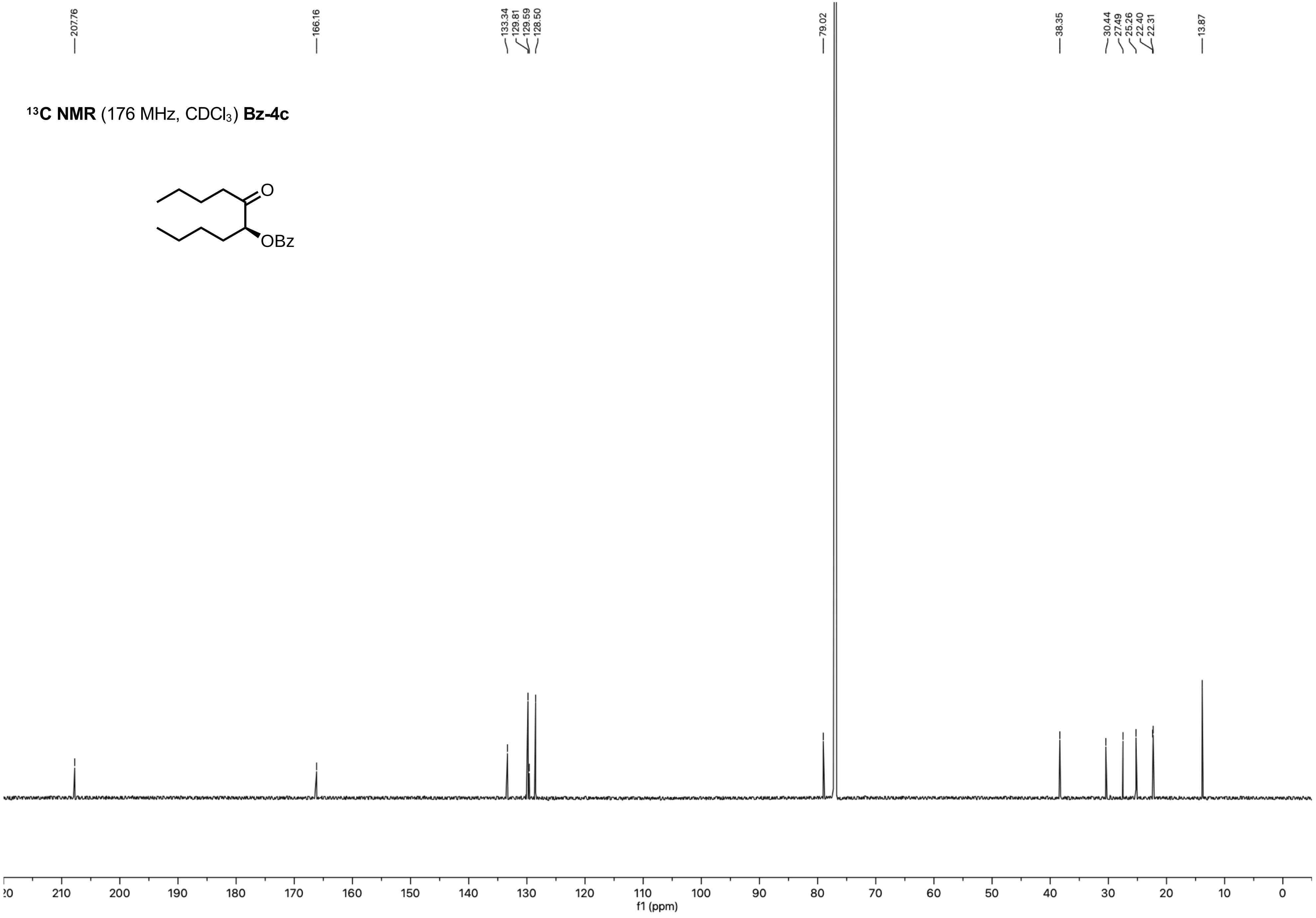

^{1}H NMR (700 MHz, $CDCl_3$) **Bz-4f**

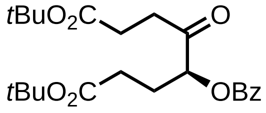

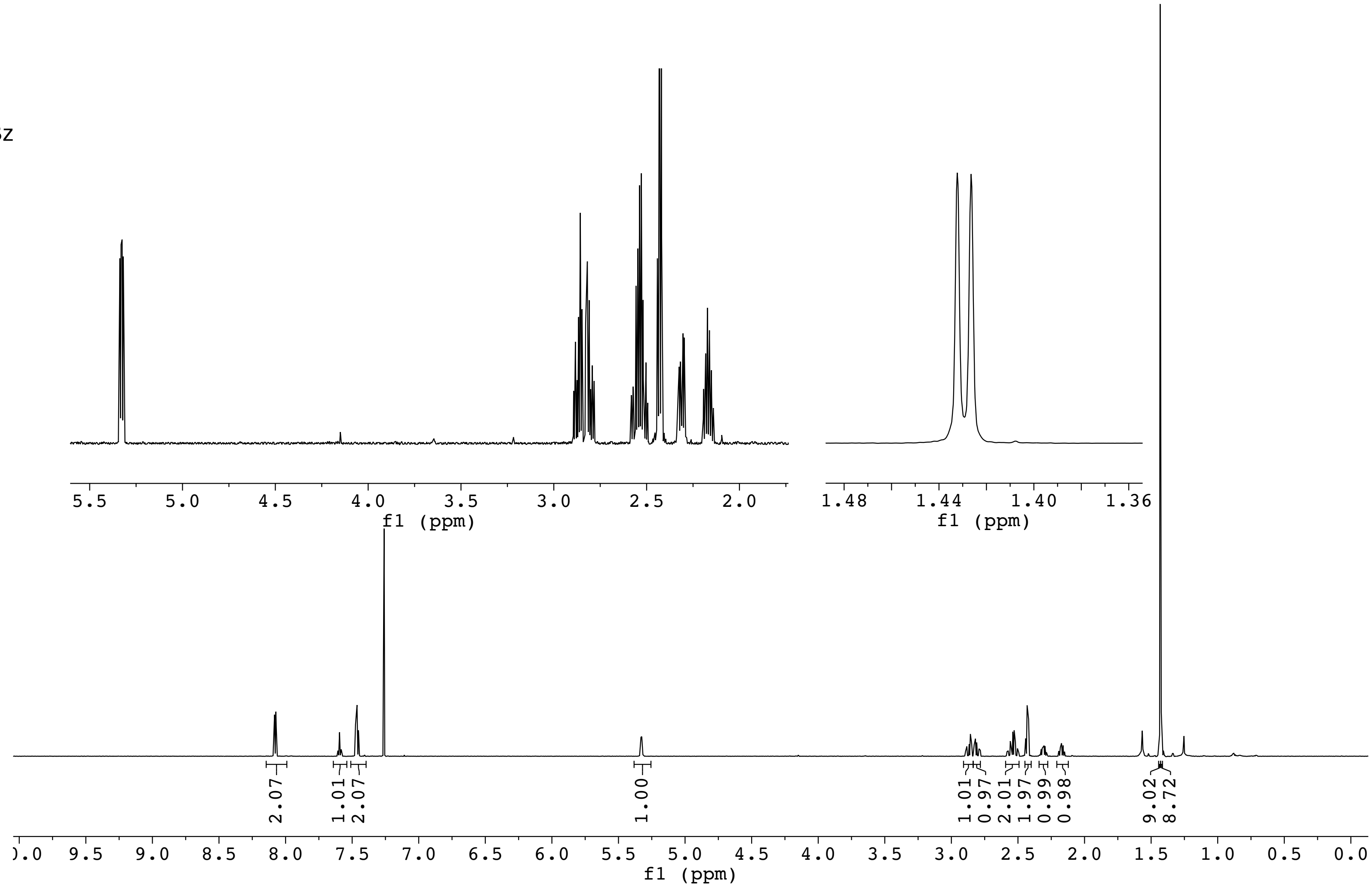

^{13}C NMR (176 MHz, $CDCl_3$) **Bz-4f**

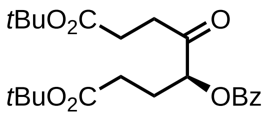

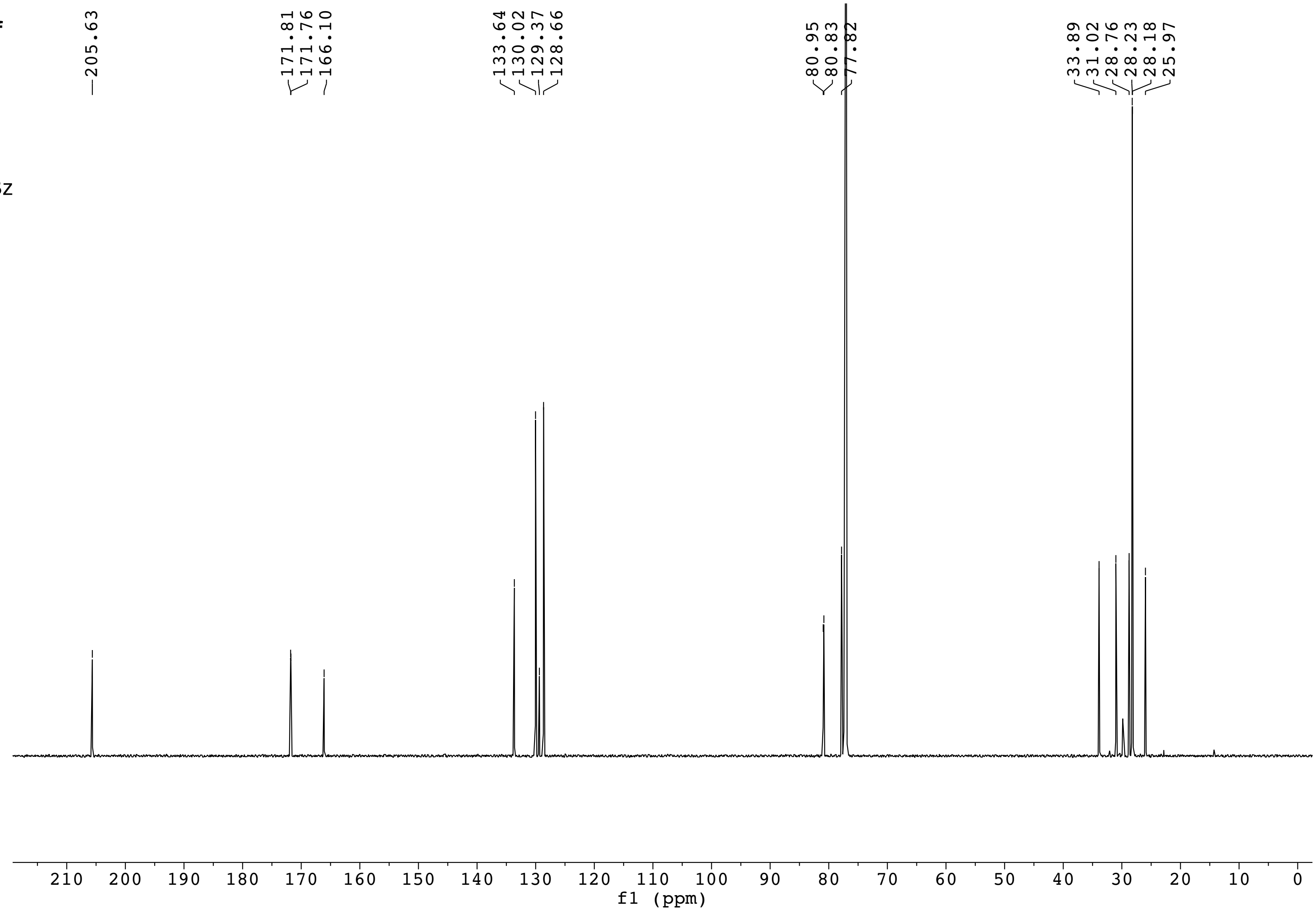

^{1}H NMR (700 MHz, $CDCl_3$) **Bz-4h**

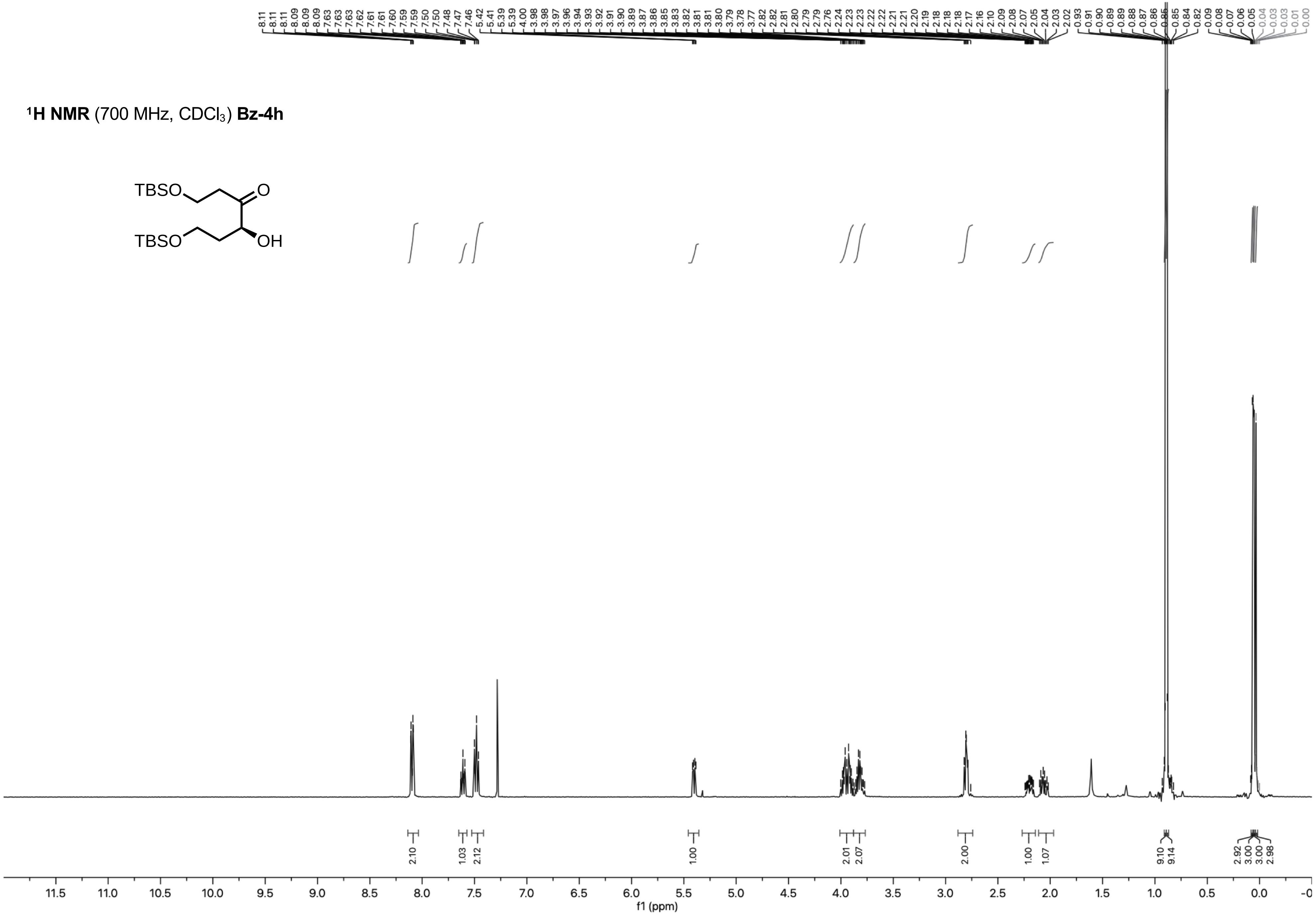

^{13}C NMR (176 MHz, $CDCl_3$) **Bz-4h**

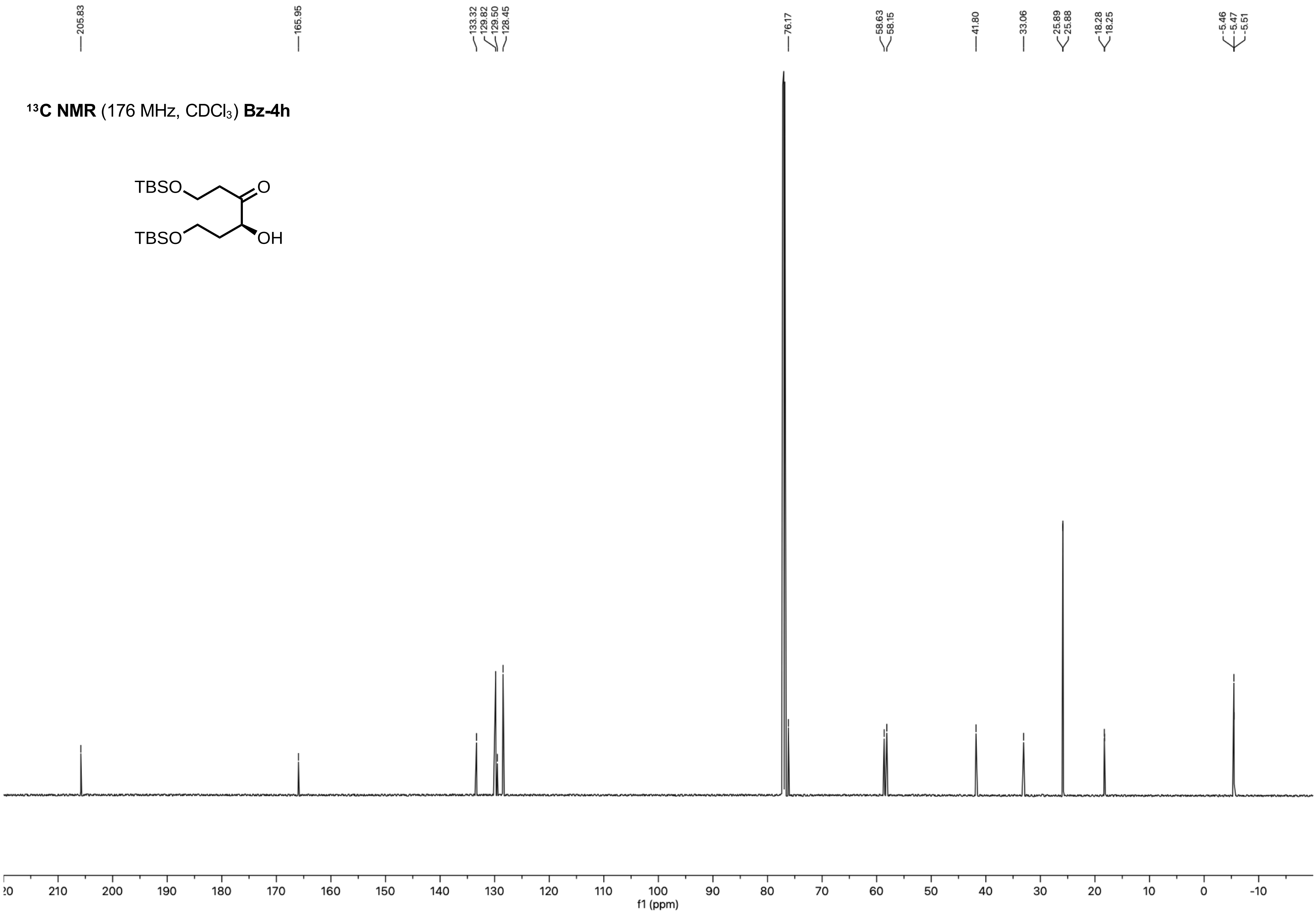

^{1}H NMR (700 MHz, $CDCl_3$) **Bz-4i**

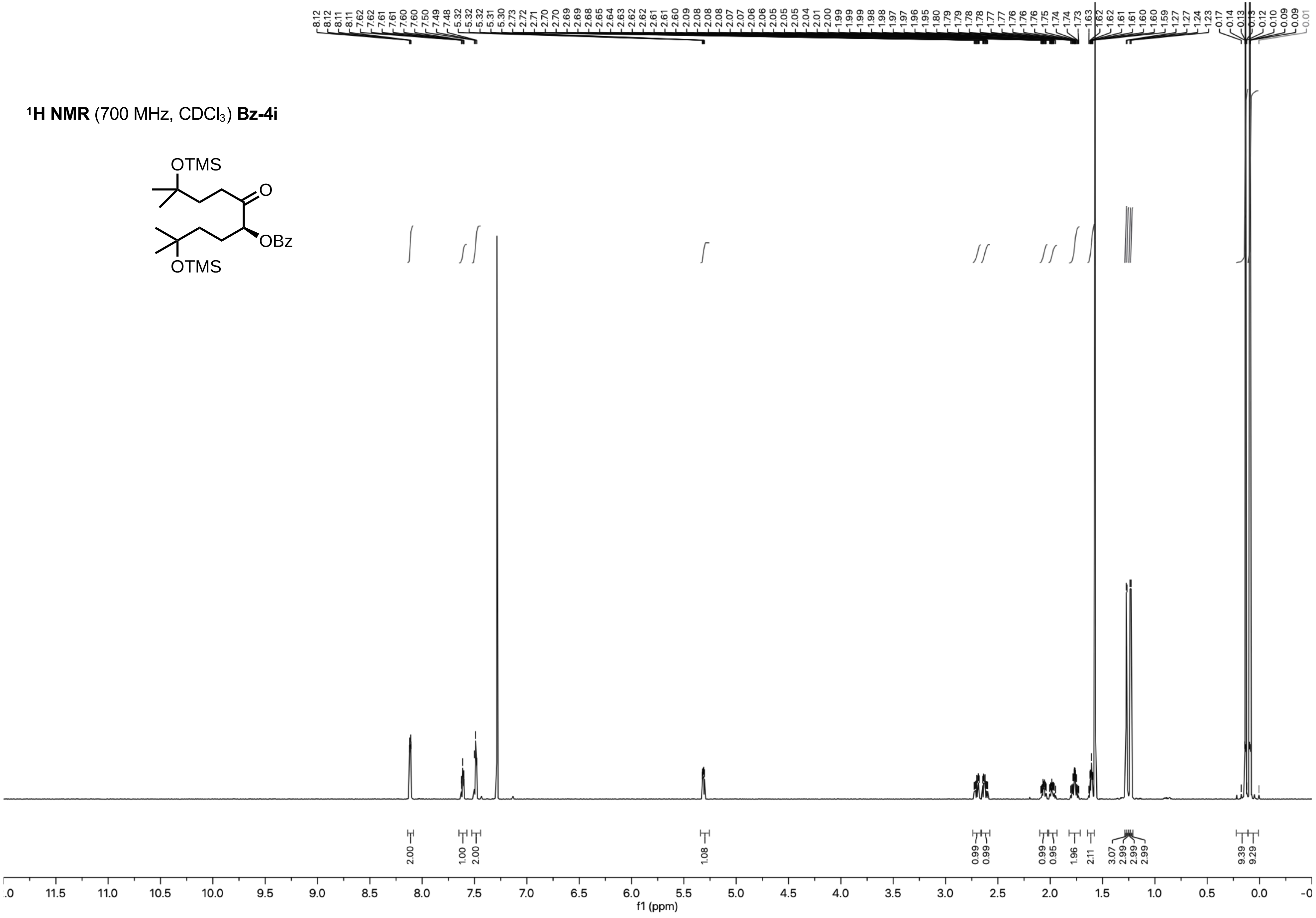

^{13}C NMR (176 MHz, $CDCl_3$) **Bz-4i**

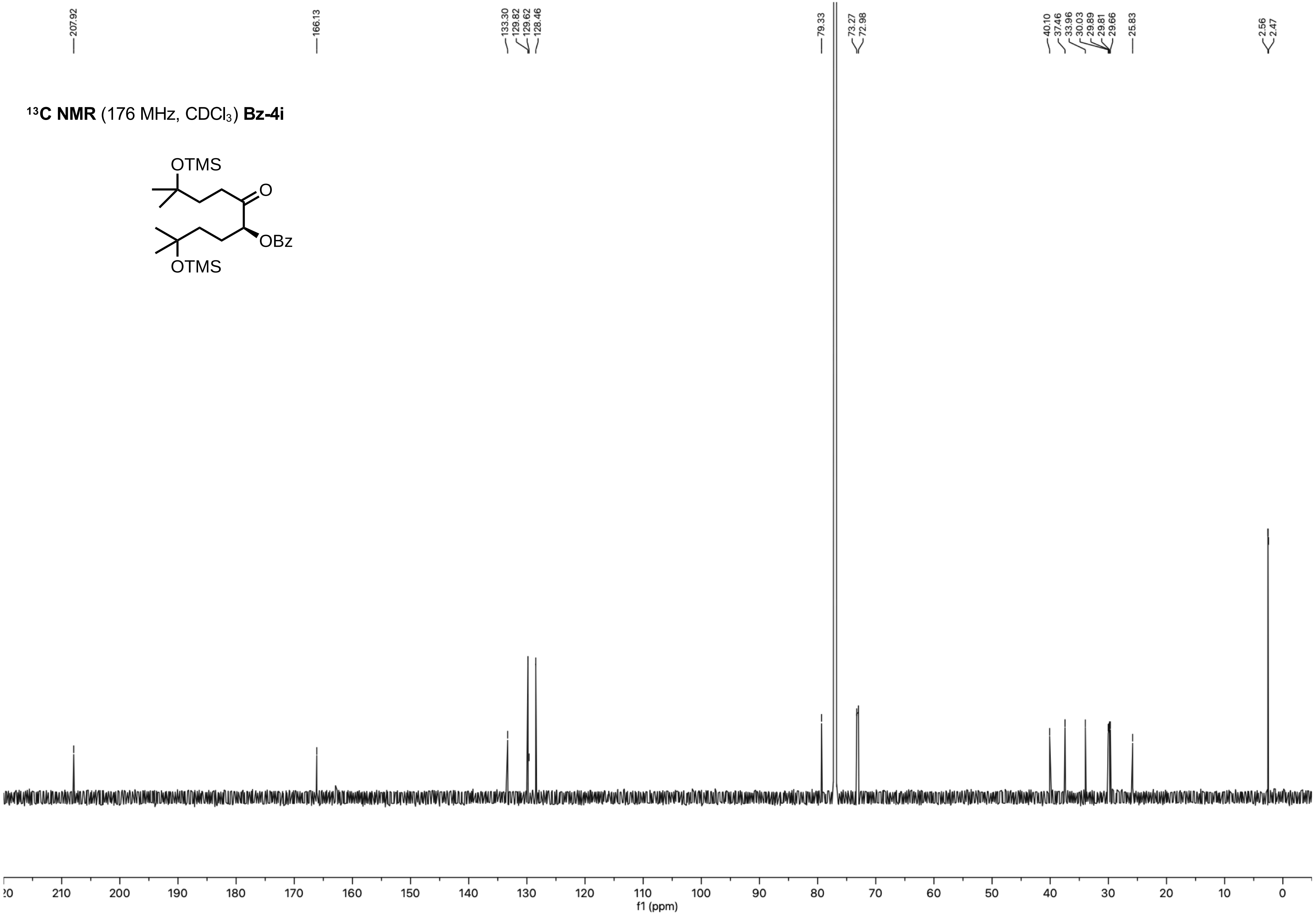

^{1}H NMR (700 MHz, $CDCl_3$) **Bz-4k**

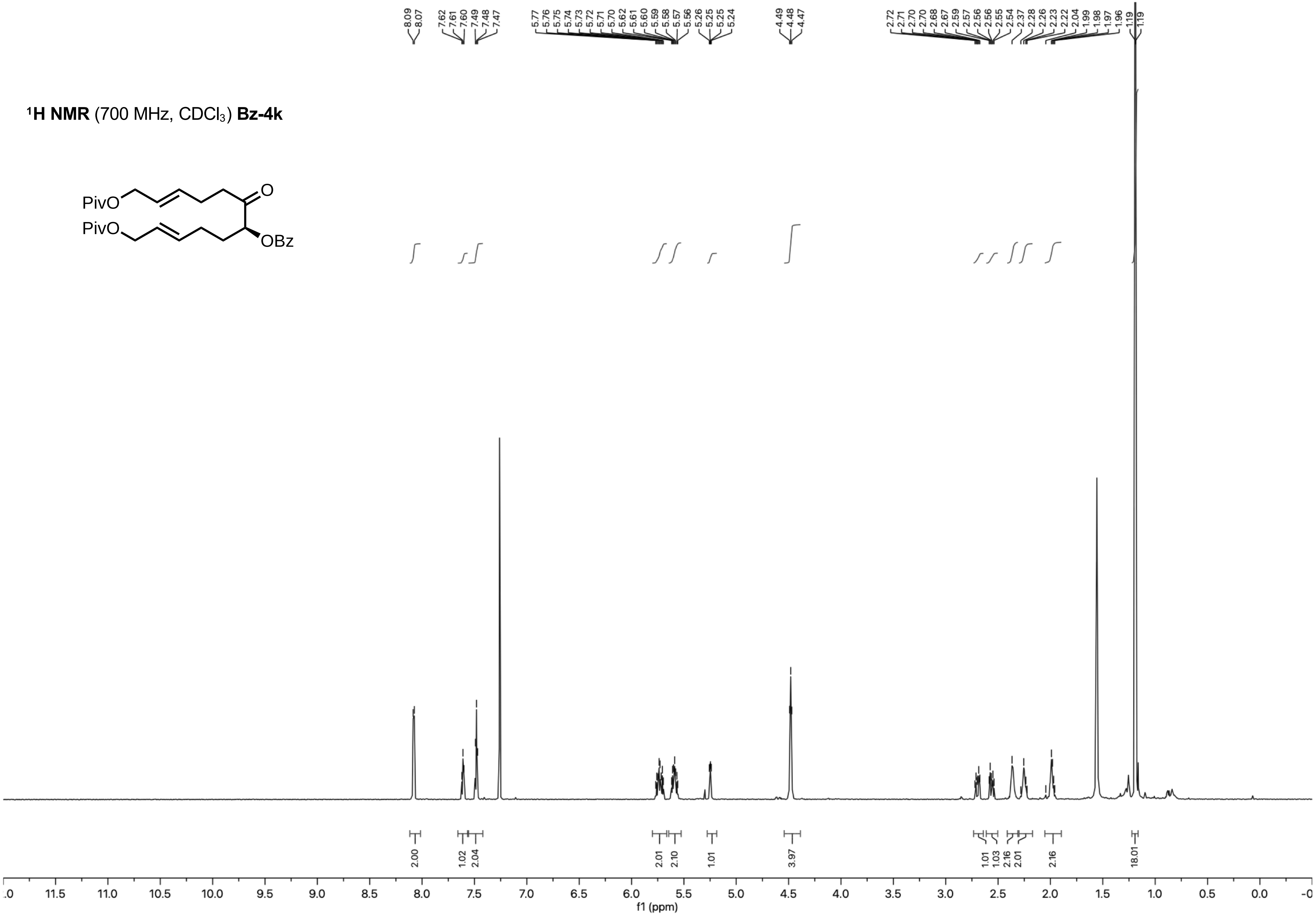

^{13}C NMR (176 MHz, $CDCl_3$) **Bz-4k**

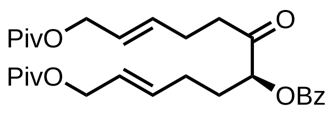

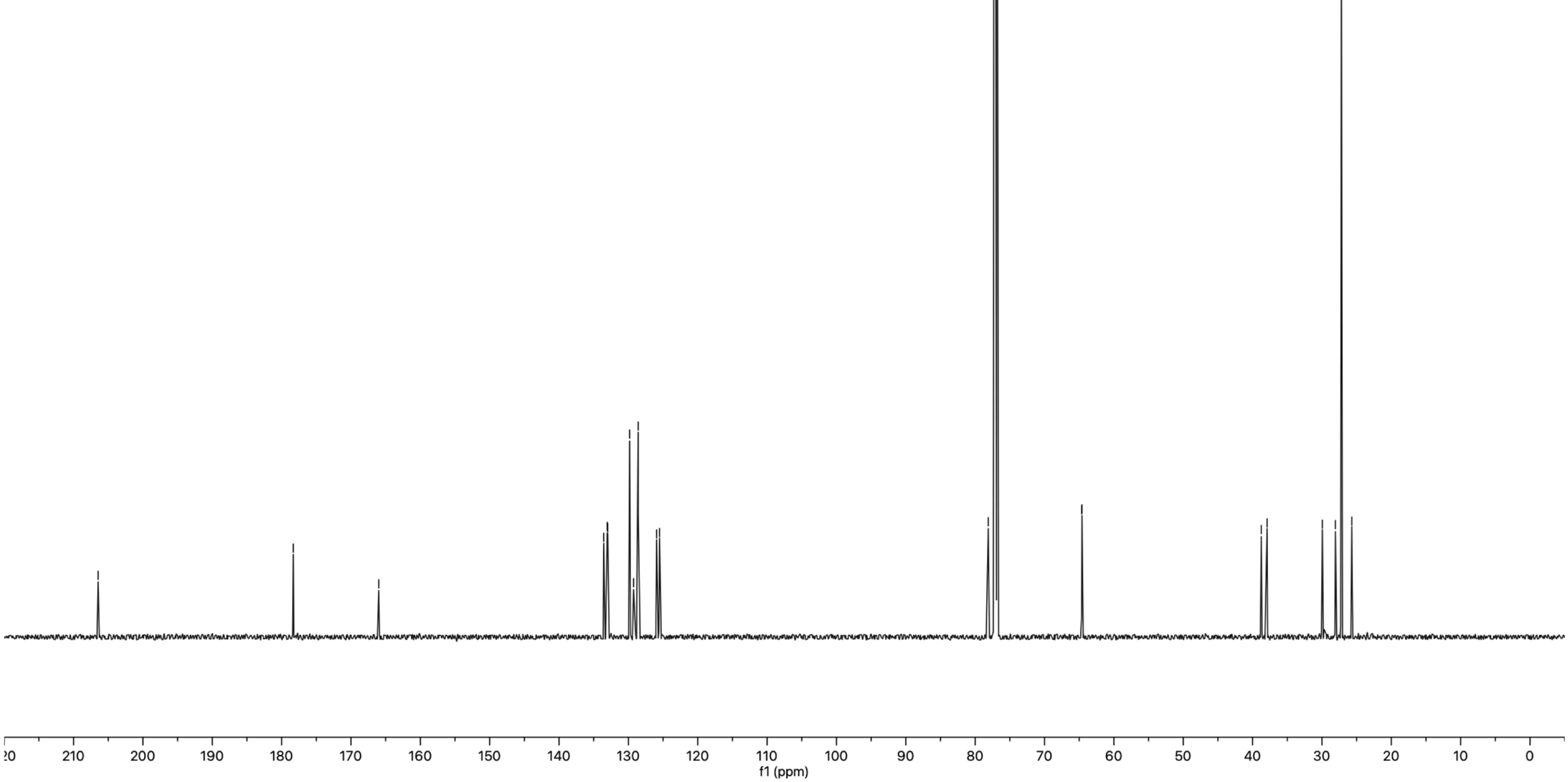

Supplement: Supplementary file 1 — ja4c13919_si_001.pdf [file ja4c13919_si_001.pdf]
